# Extracellular vesicle-associated miR-515-5p from adipose tissue regulates placental metabolism and fetal growth in gestational diabetes mellitus

**DOI:** 10.1186/s12933-025-02739-z

**Published:** 2025-05-14

**Authors:** Nanthini Jayabalan, Soumyalekshmi Nair, Andrew Lai, Katherin Scholz-Romero, Melissa Razo-Azamar, Valeska Ormazabal, Ratana Lim, Flavio Carrion, Dominic Guanzon, Gregory E. Rice, Harold David McIntyre, Martha Lappas, Carlos Salomon

**Affiliations:** 1 Translational Extracellular Vesicles in Obstetrics and Gynae-Oncology Group, University of Queensland Centre for Clinical Research, Faculty of Medicine, The University of Queensland, Brisbane, QLD 4029 Australia; 2 UQ Centre for Extracellular Vesicle nanomedicine, The University of Queensland, Brisbane, QLD 4029 Australia; 3 Faculty of Biological Sciences, Pharmacology Department, University of Concepcion, Concepción, Chile; 4 Obstetrics, Nutrition and Endocrinology Group, Department of Obstetrics and Gynaecology, University of Melbourne, Melbourne, VIC Australia; 5 Departamento de Investigación, Postgrado y Educación Continua (DIPEC), Facultad de Ciencias de la Salud, Universidad del Alba, Santiago, Chile; 6 INOVIQ Ltd, Notting Hill, VIC 3168 Australia; 7 Mater Research, Faculty of Medicine, The University of Queensland, Brisbane, QLD Australia

**Keywords:** Adipose tissue, Placenta, Gestational diabetes, Extracellular vesicles, Glucose uptake, Fetal growth

## Abstract

**Background:**

Gestational diabetes mellitus (GDM) affects 2–20% of pregnant women worldwide and is linked to fetal overgrowth, increased perinatal morbidity, and mortality, as well as a higher risk of developing cardiovascular disease later in life for mother and child. MicroRNAs (miRNAs), which regulate gene expression, can be transported within extracellular vesicles (EVs). Adipose tissue-derived EVs have been associated with changes in placental metabolism in GDM, potentially influencing cardiovascular health outcomes. This study aimed to evaluate the miRNA profile in EVs from omental adipose tissue in GDM and their effect on placental nutrient uptake and fetal growth.

**Methods:**

This case–control study included patients with normal glucose tolerance (NGT) and GDM. We conducted a miRNA expression profiling on omental adipose tissue and its derived EVs from women with NGT (*n* = 20) and GDM (*n* = 36). Trophoblast cells were utilized to assess the effect of EVs on glucose and fatty acid uptake, pro-inflammatory cytokine, and chemokine release. Double-stranded miRNA mimics were used to investigate the effect of selected miRNAs on trophoblast cells. Subsequently, the impact of EVs from NGT and GDM, as well as miR-515-5p, on in vivo glucose tolerance and fetal growth was assessed in pregnant mice.

**Results:**

Fifty-four miRNAs showed significant differences between EVs from the adipose tissue of NGT and GDM groups. EVs from GDM increased glucose uptake in trophoblast cells, whereas EVs from NGT increased the secretion of CXCL8, IL-6, CXCL1, CXCL4, and CXCL5 from trophoblasts compared to the effect without EVs. Specifically, miR-515-5p increased glucose uptake and abolished TNF-α-dependent increase in pro-inflammatory cytokines and chemokines from trophoblast cells. Injection of pregnant mice with EVs from NGT adipose tissue loaded with miR-515-5p resulted in increased fetal weight and glucose levels.

**Conclusion:**

miR-515-5p, specifically encapsulated within EVs from omental adipose tissue in GDM, regulates placental nutrient uptake, glucose homeostasis, and fetal growth.

**Supplementary Information:**

The online version contains supplementary material available at 10.1186/s12933-025-02739-z.

## Research insights

**What is currently known about this topic?**

- Gestational diabetes mellitus (GDM) increases cardiovascular risk for mother and child. Adipose tissue-derived extracellular vesicles (EVs) can influence placental metabolism. microRNAs (miRNAs) play a critical role in gene expression regulation.

**What is the key research question?**

- How do miRNAs in adipose tissue-derived EVs affect placental nutrient uptake and fetal growth in GDM?

**What is new?**

- First study to identify miR-515-5p in adipose tissue-derived EVs as a regulator of placental nutrient uptake, fetal glucose homeostasis, and growth.
- Demonstrates a mechanistic link between maternal adipose tissue EVs and fetal metabolic programming, providing new insights into GDM pathophysiology.
- Suggests miR-515-5p as a potential therapeutic target for managing fetal growth abnormalities in pregnancies complicated by GDM.

**How might this study influence clinical practice?**

- Targeting miR-515-5p may provide a novel therapeutic strategy to improve maternal and fetal outcomes in GDM pregnancies.

## Background

Gestational diabetes mellitus (GDM) is a condition characterized by hyperglycemia that develops during pregnancy, primarily due to insufficient β-cell insulin production and chronic insulin resistance. This condition significantly affects the mother and offspring, contributing to increased risks of adverse perinatal outcomes, including fetal overgrowth (macrosomia), preterm birth, and neonatal complications, such as hypoglycemia, jaundice, and respiratory distress syndrome [1].

Beyond these immediate effects, GDM also poses long-term health risks. Research has demonstrated that women with a history of GDM are at an increased risk of developing cardiovascular diseases later in life. This risk is largely attributable to shared risk factors, such as obesity, insulin resistance, and systemic inflammation, which are commonly observed in women with GDM. Insulin resistance, a hallmark of GDM, predisposes individuals to metabolic disturbances that contribute to the development of atherosclerosis and other cardiovascular conditions [2]. The link between GDM and cardiovascular disease underscores the importance of preventive measure to mitigate future cardiovascular risk in this population. However, the specific pathophysiological mechanisms underlying GDM remain poorly understood, hindering the development of effective preventive and therapeutic measures.

Adipose tissue, the body’s largest energy storage organ, composed of adipocytes and a stromal vascular fraction (SVF), which includes preadipocytes, macrophages, endothelial cells, and fibroblasts [3]. This tissue plays a critical role in regulating energy balance and metabolism. There are two primary types of adipose tissue: white adipose tissue (WAT) and brown adipose tissue (BAT). BAT is primarily involved in thermogenesis and energy metabolism [4], while WAT is primarily involved in energy storage [5]. Hypertrophic WAT is particularly associated with an increased risk of metabolic diseases, contributing to systemic metabolic dysregulation.

Hypertrophic WAT plays a major role in chronic, low-grade systemic inflammation [6], commonly termed “metainflammation.” This inflammation is characterized by the increased secretion of proinflammatory cytokines and chemokines, such as IL-6 and IL-8 [7, 8], which disrupts glucose homeostasis and insulin sensitivity [9–11]. In conditions such as GDM, WAT hypertrophy and the associated metainflammation have been implicated in the pathogenesis of the disease [12, 13]. However, findings have been inconsistent, with some research finding no significant difference in the circulating inflammatory cytokine levels between women with GDM and those with normal glucose tolerance (NGT) [14, 15]. This discrepancy suggests that other factors, such as adipose tissue-derived secretory products, may play a role in regulating glucose tolerance in GDM pregnancies.

One such secretory product is extracellular vesicles (EVs), which are lipid bilayer particles that facilitate intercellular communication by transferring a diverse range of molecular cargo, including proteins, lipids, and nucleic acids [16]. Adipose tissue-derived EVs have emerged as important mediators of systemic functions, including the regulation of glucose metabolism, insulin sensitivity, and inflammation [17–19]. A key component of these EVs is microRNAs (miRNAs), which are small non-coding RNA molecules that regulate gene expression [20]. Adipose tissue-derived miRNAs represent the largest fraction of circulating EV-miRNAs and play a crucial role in glucose homeostasis. These miRNAs can be transferred to distal tissues, such as the liver [20, 21] and skeletal muscle [21, 22], where they modulate insulin sensitivity and glucose regulation.

Despite the increasing evidence linking adipose tissue-derived EVs and their associated miRNAs to metabolic regulation and inflammation, their role in the pathophysiology of GDM remains underexplored. In this study, we hypothesized that the adipose tissue-derived EVs through their miRNA cargo, play a key role in modulating inflammation and metabolic dysregulation in GDM pregnancies, potentially influencing fetal development and maternal health outcomes. We aimed to investigate the role of adipose tissue-derived EVs and their miRNAs on placental metabolism, inflammation, and fetal growth in in vitro and in vivo models of GDM.

The findings of this study revealed a novel interaction between adipose tissue and the placenta in GDM, where miR-515-5p, a miRNA encapsulated in EVs, regulates placental metabolism and the microenvironment, ultimately affecting fetal outcomes. This research highlights the importance of adipose tissue-derived EVs and their miRNA cargo in GDM, providing new insights into the molecular mechanisms underlying placental dysfunction and fetal growth abnormalities. The identification of specific miRNAs, such as miR-515-5p, involved in these processes introduces potential avenues for developing novel diagnostic and therapeutic strategies. Such strategies could enhance maternal and fetal outcomes in GDM pregnancies, contributing to improved management of this common and potentially harmful condition.

## Methods

### Regulatory environment and data quality assurance

The experimental procedures described in the study were conducted within a research facility accredited by ISO17025. All data generated during the study were recorded within a 21 Code of Federal Regulation, part 11, compliant electronic laboratory notebook (Laboratory Archives, Carlsbad, CA).

## Sample collections

Pregnant women were recruited from the Mercy Hospital for Women (Melbourne, Victoria, Australia). Prior to participation, all participants provided informed written consent. Clinical midwives facilitated participant recruitment and acquired patient records and relevant clinical data. GDM was diagnosed through an Oral Glucose Tolerance Test (OGTT) conducted at approximately 28 weeks of gestation. GDM diagnosis followed the criteria set by the Australasian Diabetes in Pregnancy Society, which defined GDM as the presence of one or more elevated glucose levels following a 75-g oral glucose load: fasting glucose ≥ 5.1 mmol/L, 1-hour glucose ≥ 10.0 mmol/L, or 2-hour glucose ≥ 8.5 mmol/L. Women diagnosed with GDM were categorized into two management groups: those who controlled their glucose levels through diet alone and those who required insulin in addition to dietary management. GDM was considered diet-controlled alone if fasting glucose levels remained below 5.5 mmol/L for two weeks following diagnosis. Women with fasting glucose levels of ≥ 5.5 mmol/L were prescribed insulin for optimal glucose control. Participants were matched for age, weight, body mass index (BMI), and gestational age. Women with NGT were included as a control group, having received a negative diagnosis for GDM. At delivery, omental adipose tissue and placenta samples were obtained from consenting women who delivered healthy, singleton infants via caesarean section at term (defined as > 37 weeks’ gestation). The samples were collected within 10 min of delivery, ensuring the preservation of tissue integrity for subsequent analyses.

## Adipose tissue explant culture

Adipose tissue explant culture was performed as previously described [23]. Briefly, adipose tissue samples were first thoroughly washed with ice-cold PBS to remove any residual blood or contaminants. The tissue was then finely diced and incubated in DMEM at 37 °C in a humidified incubator with 21% O_2_ and 5% CO_2_ for 1 h. After the initial incubation, the adipose tissue was transferred to 24-well tissue culture plates, where it was cultured at 10 mg of tissue per mL of medium. The tissue was then incubated for an additional 24 h in serum-free DMEM. The use of serum-free medium was crucial to prevent contamination from serum-derived EVs and RNAs, which could otherwise co-purify during the isolation of EVs or RNA. After the 24-hour incubation period, the media was collected for the subsequent isolation of EVs. This method allowed for the collection of adipose tissue-derived EVs, which were then further analyzed for their molecular content and role in the study.

## Primary trophoblast cell culture

Placental villous cytotrophoblasts were isolated using a method previously described [24], through DNase/trypsin digestion to dissociate the tissue and the subsequent purification of trophoblast cells by separation on a Percoll gradient. This process enabled the isolation of primary trophoblast cells, which were then plated in a 24-well culture plate at a density of 5 × 10^^5^ cells per well. The cells were cultured at 37 °C in an atmosphere with 8% O_2_ and 5% CO_2_, using DMEM supplemented with 10% fetal bovine serum (FBS) and 1% (v/v) penicillin-streptomycin, as well as 1% (v/v) antibiotic-antimycotic to prevent microbial contamination. After 66 h of culture, the cytotrophoblast cells had differentiated into multinucleated syncytiotrophoblasts, a key step in placental development. At this point, the culture media was replaced with fresh DMEM containing 10% EVs-depleted FBS to prevent contamination from EVs present in the serum. These cells were used to investigate the role of adipose derived- EVs in regulating glucose and fatty acid metabolism and inflammation.

## Isolation and characterization of adipose tissue- derived EVs

To isolate EVs from adipose tissue explant-conditioned media, a series of centrifugation steps were performed to remove whole cells, debris, and larger particles. Initially, the conditioned media was centrifuged at 800 × g for 10 min to remove large cells, followed by 2,000 × g for 10 min to further remove cellular debris. The supernatant was then centrifuged at 12,000 × g for 10 min to eliminate any remaining larger particles. The resulting supernatant was filtered through a 0.22-µm sterile filter (Steritop™; Millipore, Billerica, MA) to remove any residual contaminants before the final EV isolation step. To concentrate the EVs, the filtered supernatant was subjected to ultracentrifugation at 200,000 × g for 120 min (Sorvall, SureSpin™ 630/36, tube angle, 900; Thermo Fisher Scientific Inc., Asheville, NC). The pellet obtained from this high-speed centrifugation, containing the EVs, was then resuspended in 150 µl of PBS and stored at − 80 °C for subsequent analysis. To characterize the isolated EVs, several methods were employed. The size distribution of the EVs was assessed using nanoparticle tracking analysis (NTA), which measures the size and concentration of particles in suspension. The abundance of proteins associated with the EVs was also measured, providing insights into the protein content of the isolated vesicles. Additionally, the morphology of the EVs was examined using electron microscopy, which provides detailed images of the vesicles’ structure. These analyses were conducted according to the guidelines set by the International Society of Extracellular Vesicles (ISEV) [25], ensuring that the EV isolation and characterization methods adhered to established standards in the field.

### Single particle analysis

The distribution and concentration of EVs were analyzed using the ExoView R100 system (Nanoview Biosciences) [26]. This system employs a microarray chip (EV-TETRA-P) coated with antibodies against EV surface markers CD81, CD63, and CD9. These antibodies specifically bind to EVs, allowing the detection of their markers at the single-particle level (~ 50 nm diameter) through single-particle interferometric reflectance imaging. In the procedure, 2.5 µg of EVs were diluted in 500 µl of incubation solution, and a 40 µl aliquot was incubated overnight on the chip. After incubation, the chip was washed three times and incubated for 1 h with diluted CD81, CD63, and CD9 antibodies. Following additional washes, the chip was analyzed using the ExoView R100. The analysis was performed in triplicates with the entire chip scanned for particle assessment.

## RNA isolation and next generation sequencing

miRNA from adipose tissue and adipose tissue-derived EVs was extracted using the miRNeasy kit (QIAGEN, Australia) and QIAzol Lysis Reagent (QIAGEN, Australia) for cells and TRIzol reagent for EVs, as previously described [27]. The quantity and quality of RNA was analyzed using Agilent 2100 Bioanalyser Small RNA kit and Qubit microRNA Assay kit. Sequencing libraries were generated using the TruSeq^®^ SmallRNA Library Prep Kit, according to the manufacturer’s instructions [28]. The final library was quantified using the KAPA Library Quantification Kit (Roche) and library size determined using the Tapestation High Sensitivity D1000. A total of 12 samples was pooled in equimolar quantities, and the final pooled library was sequenced using the MiniSeq and 75 cycles High Output kit (single end for 75 cycles).

## Gene target and gene ontology

For proteins, gene set enrichment analysis (GSEA) was performed using the R package fgsea, interrogating the MSigDB hallmarks and MSigDB gene ontology databases. Subsequently, significantly upregulated (normalized enrichment score (NES) > = 0) and downregulated (NES < 0) GSEA gene ontology results (p-value < 0.05) were clustered using the vissE R package (version 1.10.0) [29] and plotted as word clouds. Statistically significant upregulated and downregulated miRNAs (p-value < 0.05, foldchange > 2) were analyzed for gene ontology over-representation analysis (ORA) using the rbioapi R package (version 0.8.0) [30], accessing the miEAA web service (version 2.1) [31]. Afterwards, differentially regulated ORA results were clustered using the vissE R package and plotted as word clouds. Plotting of volcano and dot graphs (MSigDB hallmarks for proteins; miEAA for miRNAs) were performed using ggplot2 R package (version 3.4.4) [32].

### Cell culture and MiRNA transfection

To determine the effect of adipose tissue-derived EVs and miRNAs on placental metabolism and inflammation, primary trophoblast cells (at 24 h post-essentialization) were treated with 100 µg/ml of NGT or GDM adipose tissue-derived EVs or PBS for 24 h. For miRNA overexpression, the primary syncytiotrophoblast cells were transfected with 25 nM of miRControl (AllStars Negative Control; 1027280) or mimics, Syn-hsa-miR-146a-5p (MSY0000449), Syn-hsa-miR-516b-5p (MSY0002859), Syn-hsa-miR-515-5p (MSY0002826) (QIAGEN, Australia) and Lipofectamine 3000 (ThermoFisher, Australia) in Opti-MEM media (Gibco). After 24 h, the transfection media was replaced with fresh medium. For the inflammation study, the mimics transfected trophoblast cells were treated with 10 ng/ml TNF. Cells were collected for proteomics analysis while the media was collected for cytokines and chemokine immunoassay.

### Glucose and fatty acid uptake assay

The glucose and fatty acid uptake assay were performed using commercially available kit. The glucose uptake was measured using 2-(N-(7-Nitrobenz-2-oxa-1,3-diazol-4-yl)Amino)-2-Deoxyglucose (2-NBDG, Invitrogen™) and fatty acid uptake was measured using 4,4-Difluoro-1,3,5,7,8-Pentamethyl-4-Bora-3a,4a-Diaza-s-Indacene (BODIPY™ 493/503, Invitrogen™) following manufacturer’s protocol. Post-EVs treatment or mimics transfection, the cells were treated with 200 µM of 2-NBDG or 20 μm of BODIPY. After incubation for 30 min, cells were washed twice and fluorescence intensity was measured at 465/540 nm.

### Enzyme linked immunosorbent assay (ELISA)

Media from the EV-treated or miRNA-overexpressed primary trophoblast cell culture was assayed using a sandwich ELISA to determine the protein concentrations of IL6 and CXCL8 (Life Technologies, Mulgrave, Vic, Australia) as well as CXCL1, CXCL5, CCL2 and CCL4 (RnD Systems, Minneapolis, MN, USA) according to the manufacturer’s instructions. The inter- and intraassay coefficients of variations for all assays were < 10%.

### Real- time PCR

The quantitative real time PCR was performed to validate the adipose tissue-derived EVs miRNA sequencing data and to check the trend of the selected miRNA expression in placenta from women with NGT and GDM. Reverse transcription was performed using the miScript Reverse Transcription Kit (QIAGEN, Australia). Real-time PCR was performed using the miScript SYBR Green Kit (QIAGEN, Australia). Primers for miRNAs hsa-miR-146a-5p (MS00003535), hsa-miR-515-5p (MS00007035) and hsa-miR-516b-5p (MS00007749) were purchased from QIAGEN (Australia). The expression of each miRNA was normalized to the recommended housekeeping gene [33, 34], human RNU6-2 (RNU6B; MS00033740) and ΔCT calculated as C_T miRNAtarget_ − C_T RNU6B_, with the data presented as 2^−ΔCT^.

### EV loading with miR-515-5p

For loading EVs with miR-515-5p, an equal quantity (in µg) of purified EVs and Syn-hsa-miR-515-5p miScript miRNA Mimic (MSY0002826, QIAGEN) was combined in Gene Pulser^®^ Electroporation Buffer (Bio-Rad). Electroporation was performed using the Gene Pulser Xcell™ Electroporation System (Bio-Rad, Hercules, CA) with 4 mm cuvettes, applying 3 pulses (20 ms each) at 400 V, before the samples were immediately placed on ice. The excess miRNAs were removed from the EVs by ultrafiltration using an Amicon^®^ Ultra 100 kDa filter (Merck, Darmstadt, Germany), with extensive washing in PBS.

### Mice strain and treatment

Pregnant female C57BL/6 mice, weighing 25–26.7 g and aged 10–12 weeks at gestational day (GD) 12.5, were obtained from the Animal Resource Centre (Western Australia, Australia). The mice were randomly assigned into three groups and housed under standard laboratory conditions, with controlled temperature (19–22 °C), a 12-hour light/dark cycle, and access to a standard rodent maintenance diet and clean drinking water *ad libitum*, except during fasting periods. No significant differences in food intake patterns were observed between groups. From GD 13.5–18.5, the mice were administered daily intraperitoneal injections of 8 µg/kg body weight of NGT (*n* = 4), GDM (*n* = 4) adipose tissue-derived EVs, or miR-515-5p transfected EVs (*n* = 4), in a final volume of 300 µl. Control mice (*n* = 4) received an equal volume of PBS. The daily dose of 8 µg/kg was selected based on previous studies and adjusted for the weight of the lightest pregnant mice in the first cohort, and was used consistently throughout the study. The weight of the pregnant mice was considered an important indicator of fetal growth and number.

### Mice experiments

On gestational day 17.5, the mice were fasted for 4 h, and an OGTT was conducted using a glucose dose of 2 g/kg body weight. Blood samples were collected from the tail vein at 0, 15, 30, 45, 60, and 120 min after glucose administration, and blood glucose levels were measured using an Accu Check glucometer. After the OGTT, the mice received a final dose of either EVs or PBS and were allowed to recover overnight. On gestational day 18.5, the mice were fasted again for 4 h before being euthanized by cervical dislocation. The abdominal cavity was opened, and the fetuses were removed, counted, and weighed. The placenta were collected, weighed, and immediately frozen in liquid nitrogen for storage at − 80 °C until further analysis. Fetal blood glucose levels were also measured using a glucometer.

### Mass spectrometry

The mass spectrometry analysis of the EVs treated and mimics transfected primary trophoblast cells was performed as previously described [23, 35]. Briefly, a local ion library was generated to use in the SWATH mass spectra analysis using protein pools prepared from the samples. For SWATH analysis, 30 µg of protein of individual samples were processed using the Filter Aided Sample Preparation (FASP) method [36].

### Statistical analysis

The small RNA sequencing data was processed using Cutadapt (version 4.3) [37] to remove adapters, then analyzed to identify miRNAs using the miRDeep2 software (version 2.0.1.2) [38]. Subsequently, the identified miRNAs were quantified and normalized (median of ratios method). Differential miRNA expression (Wald test with Benjamini and Hochberg correction) was performed using the DESeq2 package in R (version 1.34.0) [39]. To ensure that the differences observed between the groups were driven primarily by disease condition and not by variations in clinical characteristics, the miRNA analysis was adjusted according to clinical characteristics of the patients including BMI and weight gain during pregnancy. This adjustment helped control for potential confounding factors, such as differences in gestational age at delivery and BMI, which could influence miRNAs. Sample analyses for nutrient uptake assay, ELISA, and RT-PCR were performed using repeated measure one-way ANOVA with Bonferroni post-hoc test for multiple comparisons. Sample analyses for maternal and fetal glucose, AUC, placental and fetal weight, placental efficiency and insulin ELISA were performed using one-way ANOVA with Bonferroni post-hoc test for multiple comparisons. Data were presented as the mean ± SEM. A *p*-value ≤ 0.05 is deemed statistically significant, unless otherwise stated. For proteomics, the data-independent acquisition (DIA) data were analyzed using Scaffold DIA (1.3.1). For each peptide, the five highest quality fragment ions were selected for quantitation. Proteins with a minimum of two identified peptides were threshold to achieve a protein FDR threshold of 1.0%. One-way ANOVA was performed with Bonferroni correction for multiple comparisons. Unpaired t-test was performed for comparison between EV-NGT and EV-GDM treatment groups. Data were presented as the mean ± SEM. A *p*-value of ≤ 0.05 is deemed statistically significant unless otherwise stated.

To evaluate the effect of EVs from normal and GDM pregnancies on fetal growth in vivo, we calculated the average fetal weight per dam, adjusted for litter size, and compared these averages across treatment groups. This approach reduces variability and minimizes the impact of small sample sizes. An Analysis of Covariance (ANCOVA) was conducted to assess whether fetal weight significantly differed between treatment groups while controlling for dam weight. Post-hoc analysis using Tukey’s Honestly Significant Difference (HSD) test identified significant pairwise differences while accounting for multiple comparisons and controlling for Type I error rates. Statistical significance was set at *p* < 0.05, and effect sizes were reported where applicable to highlight biological relevance. For each experiment presented, outliers were identified using the Robust Regression and Outlier Removal (ROUT) method, implemented in GraphPad Prism (version 10.1.0, build 264), with the false discovery rate (Q) set to 1%. This method combines nonlinear regression with false discovery rate control to detect and exclude data points that deviate significantly from the overall trend.

## Results

### Clinical characteristics of study population

This study collected samples from a diverse population, including Caucasians and individuals from various regions of Asia to account for a broad range of genetic, cultural, and environmental factors that may influence the outcomes. Primary trophoblast cultures were prepared using placenta samples from non-obese (BMI < 30 kg/m²) women (*n* = 6) with healthy, singleton pregnancies at term (37–41 weeks), who underwent a caesarean section before labor onset. Women with medical conditions, such as intra-uterine growth restriction, preeclampsia, polycystic ovarian syndrome, type 2 diabetes, or who were smokers, were excluded from the study. For adipose tissue-derived EV miRNA analysis related to GDM, omentum samples were obtained from 56 BMI-matched women (NGT, *n* = 20; GDM, *n* = 36). Among the 36 women with GDM, a similar number of GDM women were treated with diet alone (*n* = 16) or were prescribed insulin (*n* = 20). The larger number of GDM samples compared to NGT samples improves the robustness of the comparison and helps address data variability. No significant differences were observed in maternal weight or BMI at 12 weeks of pregnancy or at delivery, nor in fetal birth weight between women with NGT and GDM (Table 1). However, as expected, significant differences were observed in OGTT values at all time points between women with NGT and GDM. Among the 36 women diagnosed with gestational diabetes mellitus (GDM) in this study, 16 were managed through dietary intervention and 20 required insulin therapy. This distribution allows for exploration of potential differences in disease severity and metabolic impact within the GDM group. Insulin-treated GDM often reflects more severe glycemic dysregulation compared to diet-controlled cases, which may be associated with distinct molecular signatures, including variations in EV-associated miRNA profiles. Although this study did not stratify miRNA expression or functional outcomes based on treatment modality, the inclusion of both subgroups strengthens the representativeness of the GDM cohort and supports the broader applicability of the findings. Future analyses comparing EV-miRNA profiles between diet- and insulin-treated GDM could provide valuable insights into the heterogeneity of GDM pathophysiology and its downstream effects on placental function and fetal development.

**Table 1:** Clinical characteristics of the patients involved in this study

Maternal variables	NGT (*n* = 20)	GDM (*n* = 36)	t-test
Age (years)	30.9 ± 1.1 (23.2–40.9)	33.4 ± 0.7 (24.3–40.0)	0.0686
Height (cm)	165.6 ± 1.6 (152.0- 176.0)	161.5 ± 1.0 (148.0- 175.0)	0.0265*
Pre-pregnancy weight (kg)	81.7 ± 2.8 (60.0- 110.7)	78.8 ± 2.5 (55.5–115.0)	0.4521
Pre- pregnancy BMI (kg/m^2^)	29.7 ± 0.9 (26.2–42.1)	30.0 ± 0.7 (23.4–38.1)	0.7540
Delivery weight (kg)	93.8 ± 3.2 (70.0- 125.3)	86.9 ± 2.8 (67.5- 135.6)	0.1266
Delivery BMI (kg/m^2^)	34.4 ± 1.0 (29.5–46.1)	33.2 ± 0.8 (26.67–45.72)	0.3881
Weight gain	12.1 ± 4.3 (9.6–18.7)	8.1 ± 3.8 (6.8–16.1)	0.0006
Gestational age (weeks)	39.9 ± 0.3 (36.4–41.4)	38.4 4 ± 0.3 (33.0- 40.5)	*0.0003*
Fasting OGTT (mmol L^−1^)	4.6 ± 0.1 (4.0–5.0)	5.3 ± 0.1 (4.1–6.7)	*< 0.0001*
1 h OGTT (mmol L^−1^)	7.6 ± 0.3 (5.9–9.4)	10.5 ± 0.4 (5.0- 15.7)	*< 0.0001*
2 h OGTT (mmol L^−1^)	5.9 ± 0.3 (3.2–7.5)	8.75 ± 0.4 (5.4–17.5)	*< 0.0001*
Fetal variables			
Placental weight (g)	712.4 ± 23.9 (478.0- 990.0)	639.8 ± 25.7 (370.0- 870.0)	*0.0449*
Birth weight (g)	3526.0 ± 106.8 (2616.0- 4230.0)	3449 ± 84.03 (1997.0- 4346.0)	0.5732
Fetal gender (Male/ Female	11/9	19/17	NA

Data presented as mean ± SEM (range). All pregnancies were normotensive, non-smoking, non-alcohol or drug consuming, and without intrauterine infection or any other medical orNAobstetrical complications except GDM. BMI: Body mass index; GDM: gestational diabetes mellitus; NGT: women with normal glucose tolerance test; OGTT: Oral glucose tolerance test. Glucose was measured at fasting, 1- and 2-hour post-glucose challenge (75 g). Unpaired T-test was performed to assess statistical difference between the two groups. NA = not applicable

### GDM is associated with differential expression of MiRNAs in adipose tissue-derived EVs

EVs were isolated from adipose tissue explant-conditioned media and characterized for size, morphology, and phenotype using NTA, cryo-electron microscopy, and fluorescence imaging of single-particle interferometry (Additional file S1). The EVs exhibited a size distribution around 100 nm (mean 105 ± 35 nm) (Additional file S1A), a circular morphology (Additional file S1B), and were positive for surface markers CD63, CD81, and CD9 (Additional file S1C). Notably, EVs derived from adipose tissue of women with GDM showed significantly higher levels of CD9 positivity compared to those from women with NGT, corroborating previous findings [35, 40, 41].

**Fig. 1:**
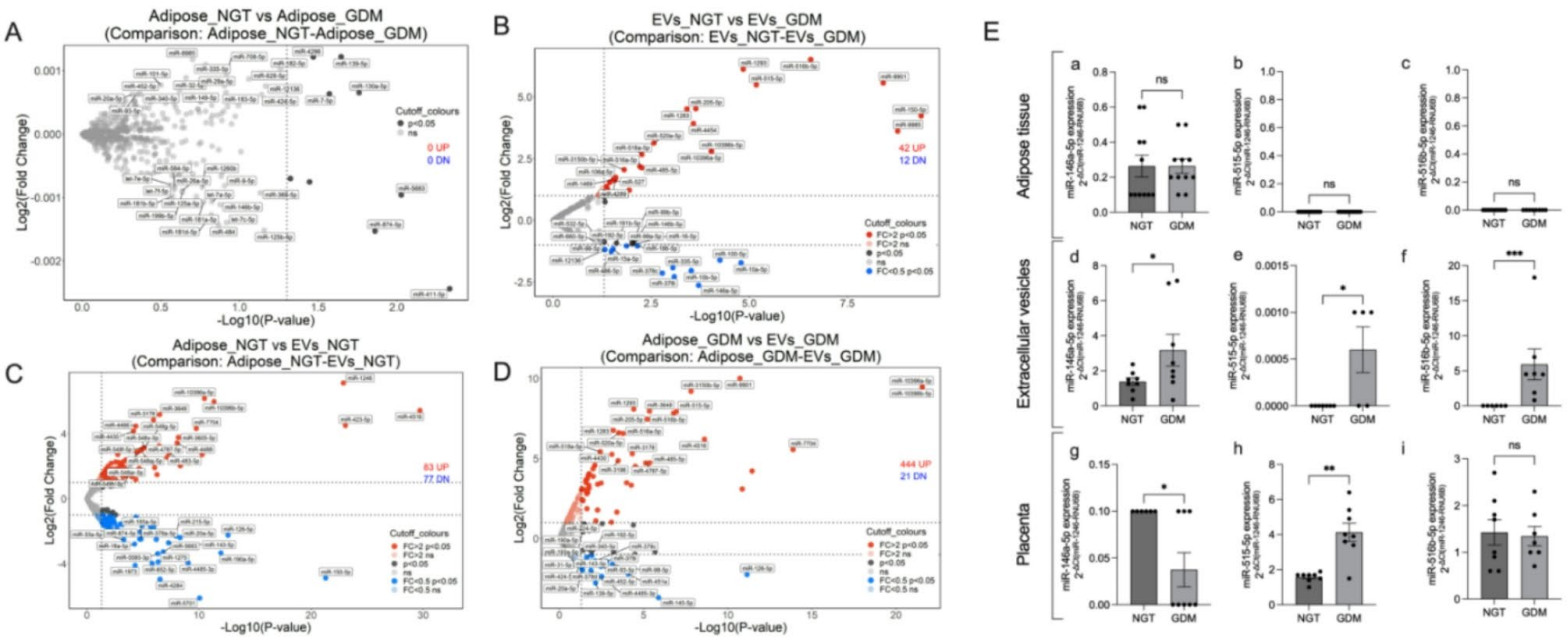
Characterization of miRNA enrichment adipose tissue and EVs in normal and gestational diabetes pregnancy. Volcano plot of miRNA expression in (**A**) NGT adipose compared to GDM adipose, (**B**) NGT EVs compared to GDM EVs, (**C**) NGT adipose compared to NGT EVs, and (**D**) GDM adipose compared to GDM EVs. (**E**) Comparative analysis of miR-146a-5p, miR-515-5p and miR-516-5p in (**a**-**c**) adipose tissue, (**d**-**f**) EVs and (**g**-**i**) placenta from NGT and GDM pregnancy (*n* = 8–12/ sample/condition). The fold change was calculated relative to NGT tissues. Non-parametric Mann–Whitney U test was performed. Data presented as mean ± SEM. *p< 0.05

miRNA expression profile was analyzed using small RNA sequencing for adipose tissue and their secreted EVs from NGT and GDM groups (Fig. 1). The comparison of miRNA profiles between NGT and GDM adipose tissue (Fig. 1A), between NGT and GDM EVs (Fig. 1B), and within groups (NGT and GDM) between adipose tissue and EVs (Fig. 1C and D) showed interesting patterns. A total of 2656 mature miRNAs were analyzed, with 42 miRNAs upregulated and 12 downregulated in GDM adipose tissue-derived EVs relative to NGT. No significant differences were found in the miRNA profiles of adipose tissue between NGT and GDM (Fig. 1A and B). In NGT, 83 miRNAs were upregulated in EVs compared to adipose tissue, while 77 miRNAs were downregulated (Additional file T1-T4). In contrast, major differences were observed in GDM, where 404 miRNAs were upregulated and 21 miRNAs downregulated in EVs compared to adipose tissue (Additional file T1-T4).

Pathway enrichment analysis of genes targeted by the differentially expressed miRNAs revealed enrichment of pathways related to placental metabolism (Additional file S2A). Gene expression data showed further enrichment in metabolic-related pathways, such as TNF-α signaling, inflammatory response, and hexokinase activity (Additional file S2B and T5).

The expression of selected miRNAs—miR-146a-5p, miR-515-5p, and miR-516-5p—was evaluated in adipose tissue, EVs, and placental tissues from women with NGT and GDM. These miRNAs were chosen based on their upregulation in EVs compared with their adipose tissue origin in GDM. Consistent with the RNA sequencing results, no significant differences were found in the expression of these miRNAs in adipose tissue between NGT and GDM (Fig. 1Ea-c). However, all three miRNAs were upregulated in EVs from NGT compared to GDM (Fig. 1Ed-f). In placental tissues, miR-146a-5p was downregulated, while miR-515-5p was upregulated in GDM compared to NGT (Fig. 1E g and h). No difference was observed for miR-516-5p expression in placental tissues between the two groups (Fig. 1Ei).

### Adipose tissue-derived EVs regulates glucose uptake and placental inflammation

**Fig. 2:**
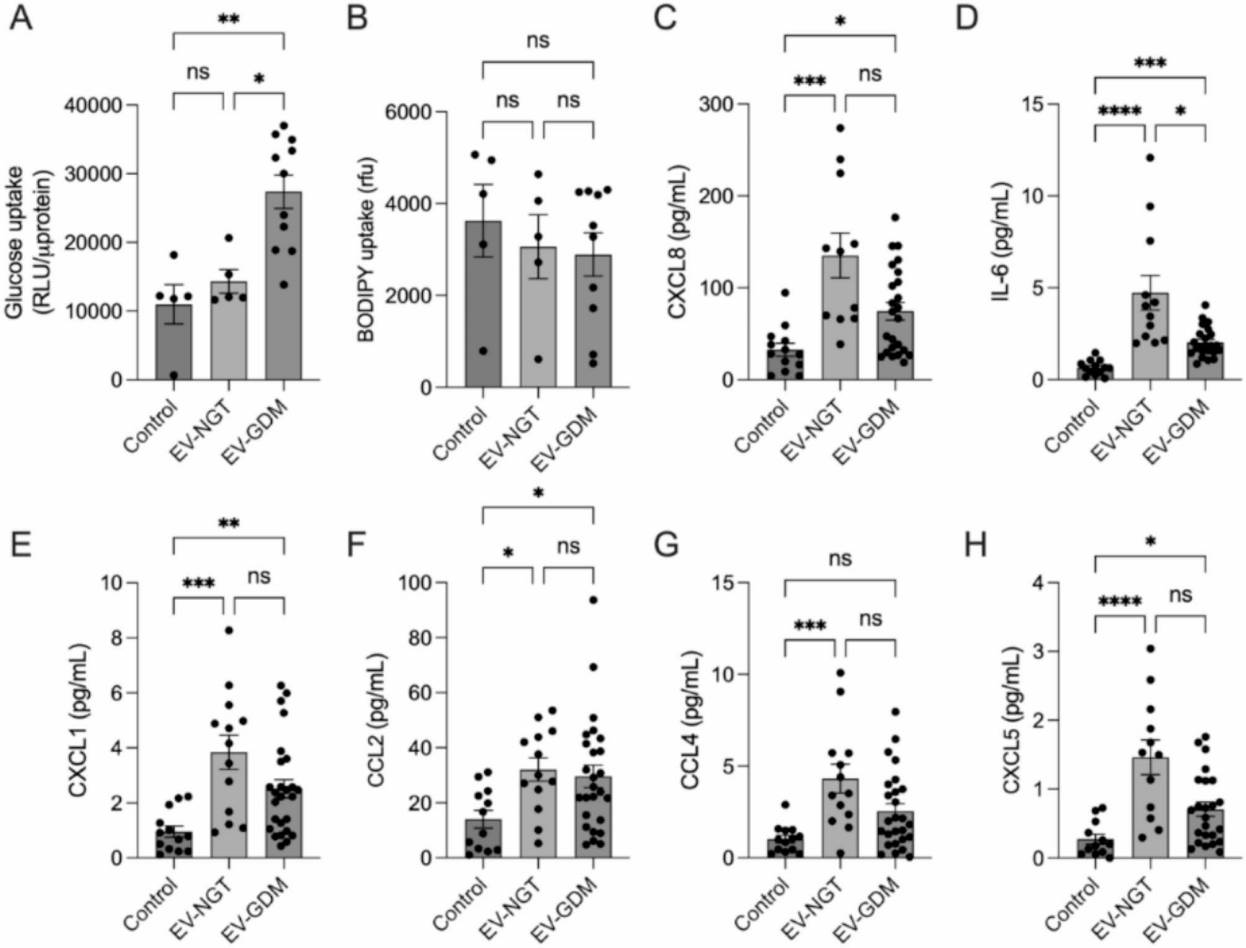
Effects of adipose tissue-derived EVs on placental cells. Effect of adipose tissue-derived EVs on placental (**A**) glucose uptake and (**B**) fatty acid uptake by 2-NBDG and BODIPY staining (*n* = 5–12 patients/condition). Glucose and fatty acid accumulation measured as relative fluorescence unit (rfu). The release of (**C**) CXCL8, (**D**) IL-6, (**E**) CXCL1, (**F**) CCL2, (**G**) CCL4, and (**H**) CCL5 measured using ELISA (*n* = 13 patients/condition). All assays were performed after 24 h of treatment with adipose tissue-derived EV. Repeated measure one-way ANOVA was performed with Bonferroni test. Data presented as mean ± SEM. *p < 0.05 **p < 0.005, ***p < 0.0005, ****p < 0.001

In this study, we examined the effects of adipose tissue-derived EVs from women with NGT and GDM on placental trophoblast cells (Fig. 2). Our results showed that EVs from GDM significantly increased glucose uptake (2.8 times higher) compared to NGT EVs and controls (Fig. 2A). No differences were observed in lipid uptake, as measured by BODIPY, across conditions (Fig. 2B). EVs from women with NGT increased the secretion of several chemokines, including CXCL8 (Fig. 2C), IL-6 (Fig. 2D), CXCL1 (Fig. 2E), CCL2 (Fig. 2F), CCL4 (Fig. 2G), and CXCL5 (Fig. 2H). Meanwhile, GDM-derived EVs did not alter the secretion of CXCL8, CCL2, or CCL4 compared to controls. GDM-derived EVs Increased the levels of IL-6 and CXCL5 compared to control. Additionally, no significant change was observed in GLUT1 mRNA expression in trophoblast cells incubated with either NGT or GDM-derived EVs (Additional file S3).

### miR-146a-5p, miR-515-5p, and miR-516-5p regulate glucose uptake, pro-inflammatory environment, and protein profile in primary trophoblast cells

**Fig. 3:**
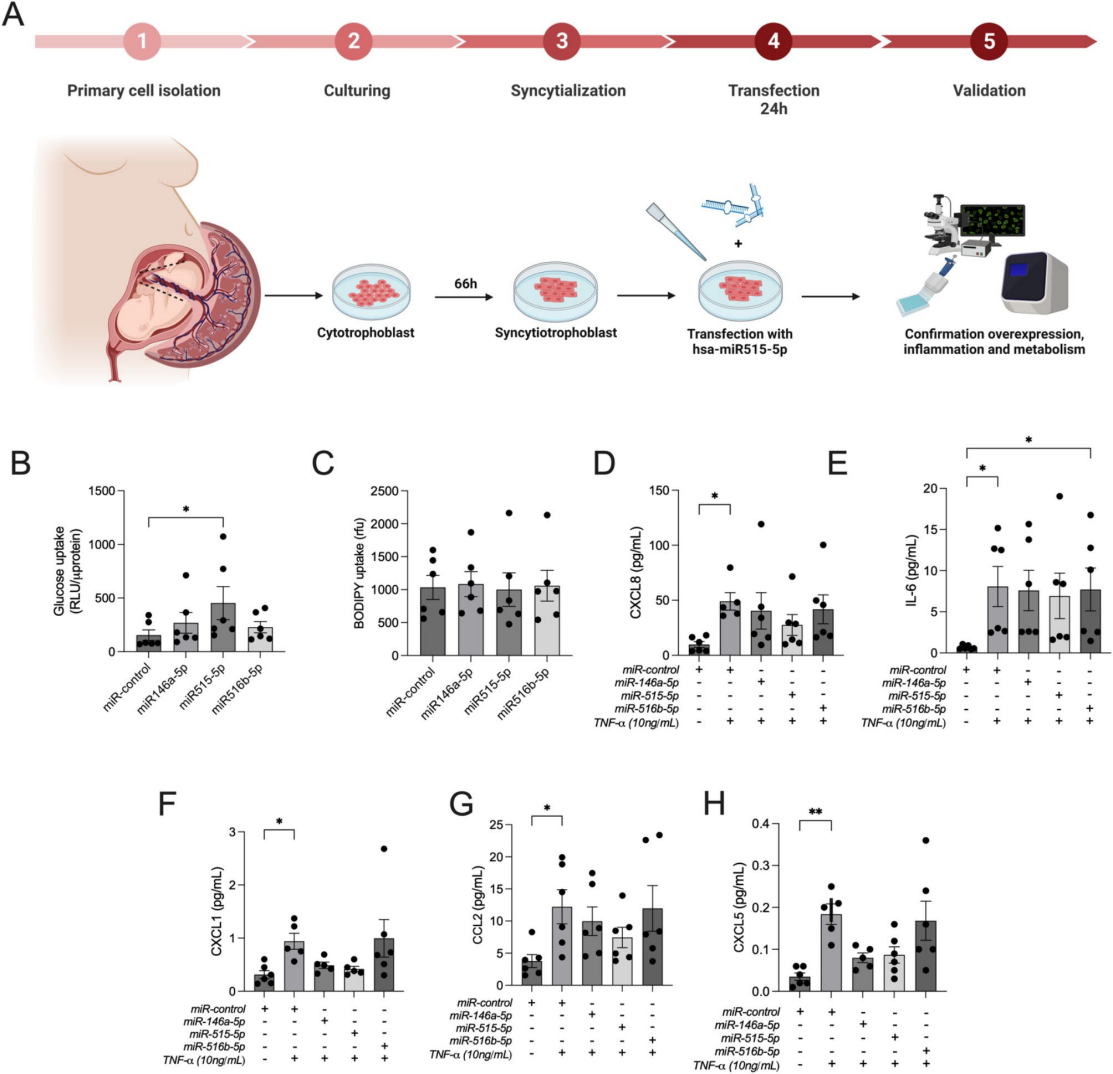
Effects of adipose tissue-derived EVs associated miRNA on placental cells. (**A**) Schematic diagram of miRNA transfection in primary trophoblast experimental design. Effect of adipose tissue-derived EVs associated miRNA, (**B**) glucose uptake, and (**C**) fatty acid uptake by 2-NBDG and BODIPY staining (*n* = 6 patients). Glucose and fatty acid accumulation measured as relative fluorescence unit (rfu). The TNF- induced release of (**D**) CXCL8, (**E**) IL-6, (**F**) CXCL1, (**G**) CCL2, and (**H**) CCL5 measured using ELISA (*n* = 6 patients). All assays were performed after 48 h of transfection with miRControl and miRNA mimics. Repeated measure one-way ANOVA was performed with Bonferroni test. Data presented as mean ± SEM. *p < 0.05 **p < 0.005

To investigate the mechanism underlying the effects of adipose tissue-derived EVs on key biological processes involved in placental nutrient uptake and metabolism, we performed overexpression experiments using miRNA mimics for miRNA-146a-5p, miRNA-515-5p, and miRNA-516-5p. These miRNAs were transfected into primary trophoblast cells isolated from placentae of healthy NGT pregnancies. Subsequently, glucose uptake, fatty acid uptake, and chemokine secretion were assessed (Fig. 3A). Transfection efficiency in trophoblast cells was evaluated using real-time PCR, which confirmed high levels of miRNA expression 24 h post-transfection, without affecting cell viability (Additional file S4 and S5). Overexpression of miR-515-5p resulted in a significant increase in glucose uptake (three-fold higher) compared to miR-control, miR-146a-5p, and miR-516-5p (Fig. 3B). However, no significant differences were observed in fatty acid uptake following overexpression of any of the three miRNAs (Fig. 3C).

In this study, primary trophoblast cells were exposed to TNF-α (10 ng/mL) to simulate the inflammatory environment in GDM [42]. The impact of overexpressing miR-146a-5p, miR-515-5p, and miR-516-5p was evaluated. TNF-α stimulation resulted in increased secretion of various chemokines, including CXCL-8 (Fig. 3D), IL-6 (Fig. 3E), CXCL-1 (Fig. 3F), CCL-2 (Fig. 3G), and CXCL-5 (Fig. 3H), but overexpression of all three miRNAs completely suppressed this response, except for IL-6 and miR-516-5p.

**Fig. 4:**
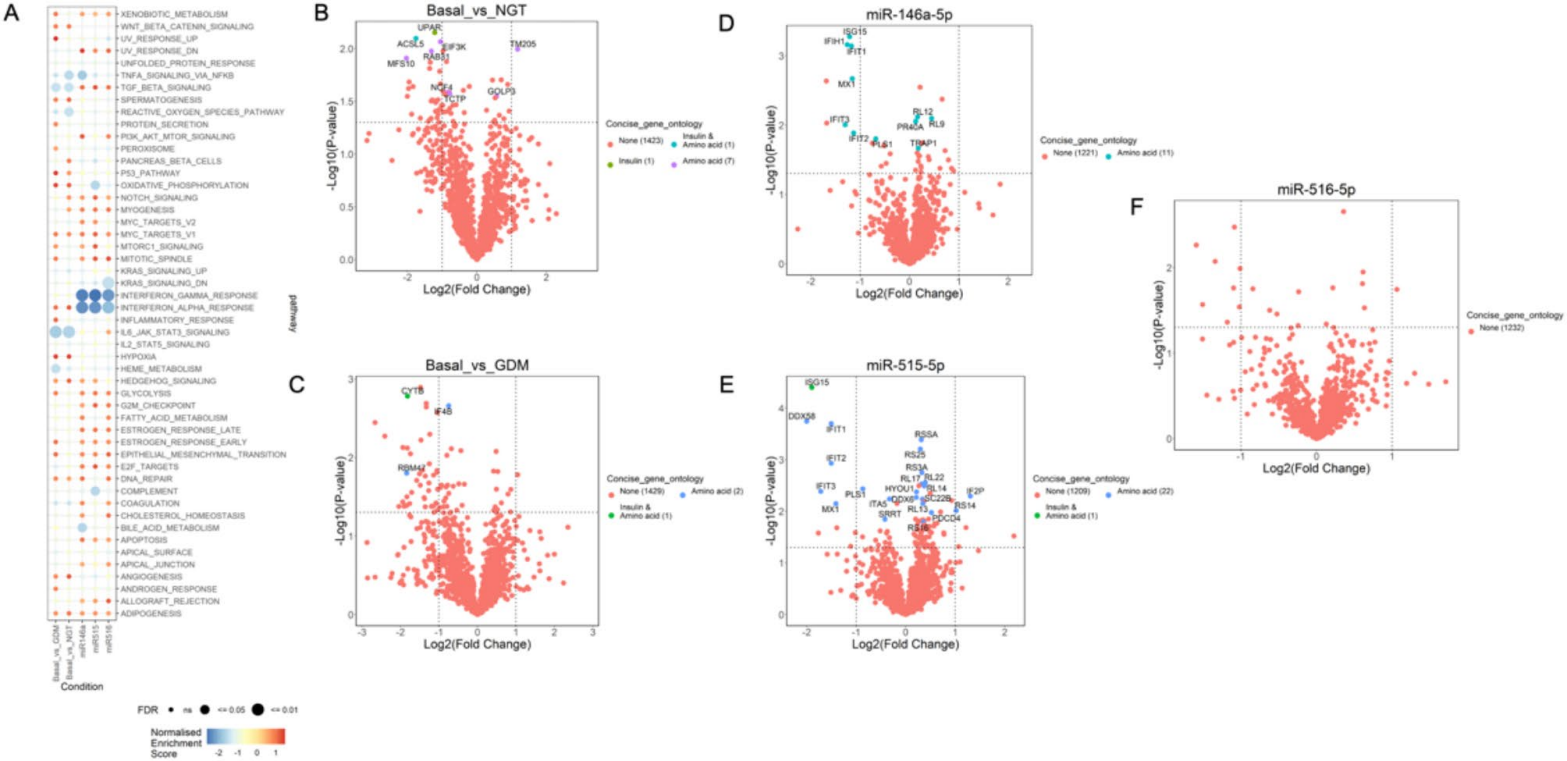
Enrichment of placental pathways associated with metabolism and inflammation. (**A**) Differentially enriched pathways in placental cells transfected with miRNAs. Panel A: miRCont, Panel B: miR-146a-5p, Panel C: miR-515-5p and Panel D: miR-516-5p. Volcano plot gene expression in (**B**) NGT, (**C**) GDM placental tissues, (**D**) miR-146a-5p, (**E**) miR-515-5p, and (**F**) miR-516-5p transfected placental cells (*n* = 5–6 patients)

A quantitative proteomics approach revealed that adipose tissue-derived EVs from GDM (Additional file T6) altered pathways related to IL-6, JAK-STAT3 signaling, TGF-β signaling, and ROS (Fig. 4A and B), while EVs from NGT altered TNF-α signaling via NF-kB (Additional file T7). Overexpressing miR-146a-5p influenced genes linked to interferon responses that might induce the expression of inflammatory mediators (Additional file T8), miR-515-5p impacted oxidative phosphorylation (Additional file T9), and miR-516-5p affected proteins and pathways associated with downregulation of KRAS signaling (Additional file T10). A comparison of the effects of adipose tissue-derived EVs from NGT, GDM, and the overexpression of miR-146a-5p, miR-515-5p, and miR-516-5p on the proteomic profile of trophoblast cells, revealed significant changes across all conditions studied (Fig. 4A–F). Specifically, overexpression of miR-515-5p led to dysregulated expression of genes, such as EMD and IFIH1, which are involved in metabolism (Fig. 4E). This highlights the potential role of miR-515-5p in regulating placental nutrient uptake and metabolism in GDM by affecting genes associated with metabolic processes. Pathway enrichment analysis of proteins dysregulated by miRNAs in GDM revealed significant enrichment in key metabolic pathways, including glycolysis, fatty acid metabolism, and mTORC1 signaling (Additional File S6). These pathways play a critical role in glucose homeostasis, insulin sensitivity, and energy regulation, all of which are disrupted in GDM. Dysregulation of these processes may contribute to altered placental function, fetal overgrowth, and long-term metabolic complications for both the mother and offspring.

### miR-515-5p mimics associated with adipose tissue-derived EVs regulates fetal growth in vivo replicates the effect in GDM

**Fig. 5:**
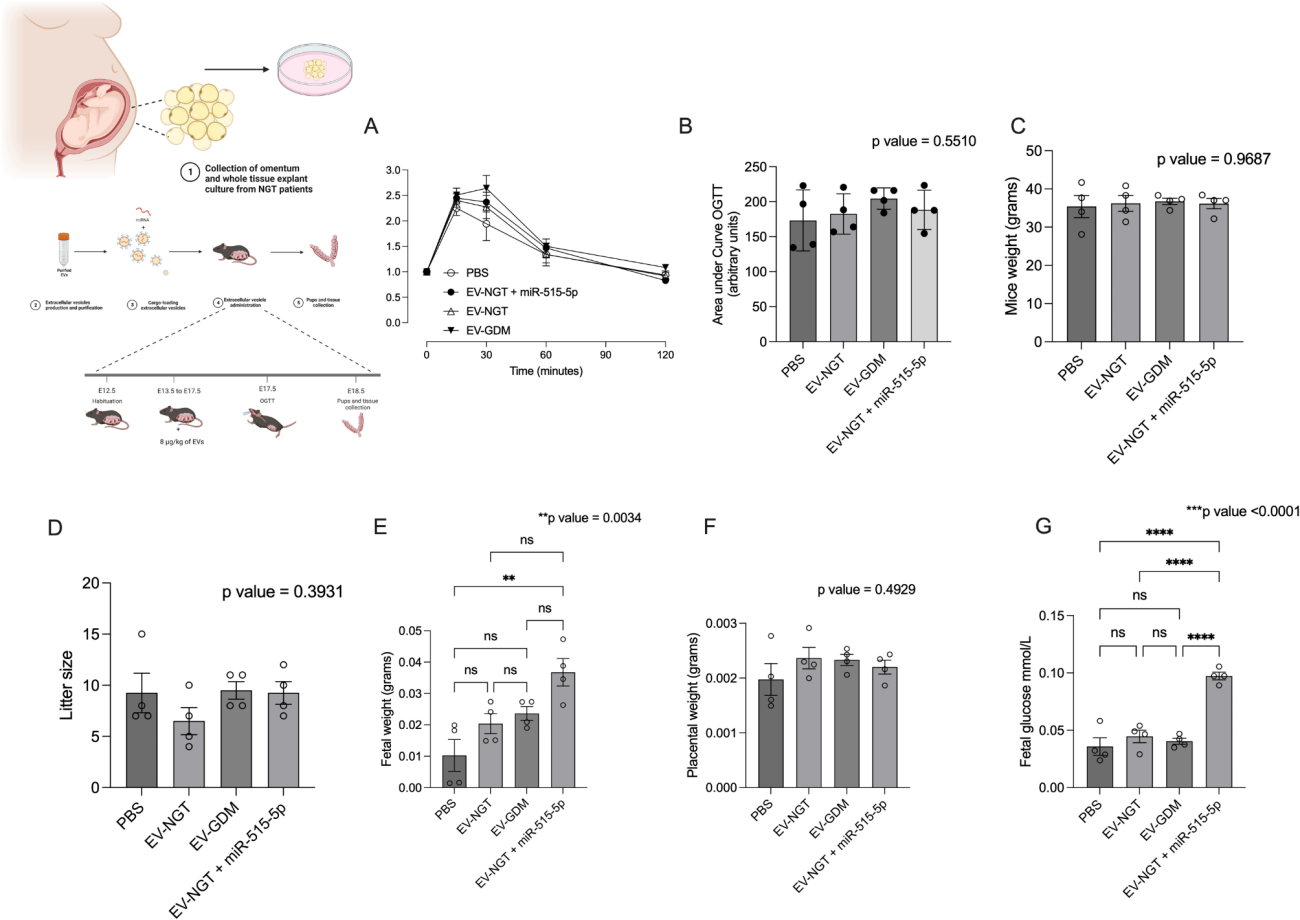
Adipose derived EVs and miRNA regulates glucose metabolism and fetal growth. (Right) Overview of experimental design. Pregnant mice (*n* = 4 per group) received daily doses of 8 µg/kg of EVs from women with normal pregnancy or GDM, EVs-transfected with miR-515-5p or PBS as Control. (**A**) Maternal blood glucose and (**B**) the area under the blood glucose response curve were measured *via* tail bleeding on GD17.5 after 4 days of EVs or PBS injection. (**C**) Mice weight, (**D**) litter size (number of fetuses per pregnant mouse), (**E**) Fetal weight and (**F**) placental weight was measured on post-operative after 5 days of EVs injection. (**G**) Fetal glucose was measured using fetal blood on PO post during fetal and tissue collection. P values in the figures represent the results of a one-way ANOVA, followed by a Bonferroni post hoc test. Data displayed as mean ± SEM. *p < 0.05 **p < 0.005, ***p < 0.0005,****p < 0.001. Fetal weight, placental weight and fetal glucose are presented as the average fetal weight per dam, adjusted for litter size, and compared these averages across treatment groups

Building on the understanding of the molecular mechanisms behind the impact of adipose tissue-derived EVs on placental function in the context of GDM, the next series of experiments explored the effects of EVs and miR-515-5p on fetal growth in vivo. Optimization of miR-515-5p loading into adipose tissue-derived EVs via electroporation determined the optimal loading conditions at 400 volts and three pulses (Additional file S7). Pregnant mice were injected with adipose tissue-derived EVs from women with normal NGT, GDM, and EVs loaded with a miR-515-5p mimic (Fig. 5). No difference on glucose levels following a glucose challenge after fasting was identified (Fig. 5A and B). Moreover, no differences in mice weight (Fig. 5C) or litter size (Fig. 5D) were observed across the different experimental conditions. However, the results showed that pups from pregnant mice injected with NGT-derived EVs loaded with miR-515-5p had significantly higher fetal weight (3.1-fold increase) compared to control (PBS) injections (Fig. 5E). However, no differences in placental weight were observed across the different experimental conditions (Fig. 5F). Interestingly, higher levels of fetal glucose from mice injected with NGT-derived EVs loaded with miR-515-5p compared to all the conditions were observed (Fig. 5G).

## Discussion

Adipose tissue functions as an endocrine organ that secretes adipokines [43, 44], cytokines [45, 46], and EVs [47, 48], which play a significant role in regulating various physiological processes, such as glucose metabolism, inflammation, and angiogenesis. During pregnancy, effective communication between adipose tissue and placenta is crucial for maintaining metabolic balance [49], fetal development [50], and placental function [51].

miRNAs associated with adipose-derived EVs comprised the largest fraction of circulating miRNAs [20] and play a crucial role in regulating inflammation [48] and glucose and lipid metabolism [52] in the recipient cells. The findings of this study highlight the important role of EVs derived from adipose tissue in GDM and its impact on placental function and fetal development. This study makes a significant contribution to understanding the pathophysiology of GDM by identifying specific miRNAs within adipose-derived EVs that may influence maternal and fetal health.

Notably, this study is the first of its kind to characterize and compare the miRNA expression profiles of adipose tissue and adipose tissue-derived EVs in normal and GDM pregnancies. By investigating the effects of these EVs on placental nutrient uptake and inflammation, our findings provide new insights into how adipose tissue communicates with the placenta via EVs, potentially uncovering new regulatory mechanisms of metabolic and inflammatory pathways that influence pregnancy outcomes.

In this study, we observed a significant difference in the miRNA expression between GDM adipose tissue and the corresponding EVs. This finding is not surprising, as EVs can selectively package miRNAs, which may not necessarily reflect the overall miRNA expression in the parental adipose cells [53, 54]. The selective packaging of miRNAs into EVs is a well-documented phenomenon, as EVs can modulate the miRNA content they carry depending on various factors, such as the physiological or pathological state of the donor cells. Therefore, the differences in miRNA expression between adipose tissue and its derived EVs suggest that EVs may play a distinct and important role in mediating signaling between adipose tissue and other tissues, in this context, the placenta.

We identified a distinct set of miRNAs with elevated expression levels in EVs derived from GDM adipose tissue compared to those from NGT. Notably, miR-146a-5p, miR-515-5p, and miR-516-5p were specifically enriched and upregulated in EVs, reflecting the selective packaging of miRNAs, which may vary depending on the pathological condition. The increased expression of these three miRNAs in GDM adipose tissue-derived EVs suggests a selective packaging mechanism specific to GDM. These miRNAs target genes involved in cell metabolism and nutrient uptake, and their expression patterns closely resemble those found in placental tissues from NGT and GDM pregnancies. The targeting of metabolic pathways by these differentially expressed miRNAs supports the idea that adipose tissue actively responds to the maternal diabetic environment during pregnancy, with this response mediated by miRNAs within EVs. Overall, these findings suggest that EVs and their miRNA content may interact with the placenta, potentially regulating key processes related to placental nutrient uptake and metabolism.

Trophoblast cells, forming the outer layer of the placenta, crucially regulate nutrient transport, including glucose and fatty acids, from the maternal bloodstream to the developing fetus [55], while also potentially exhibiting altered patterns of chemokine secretion, resulting in dysregulated immune cell recruitment and function within the placenta [56]. Increased placental nutrient uptake and heightened inflammation are among the central features of GDM pregnancy. We found that adipose tissue- derived EVs from GDM increased placental glucose uptake, suggesting a potential role for these EVs in modulating placental metabolism, particularly glucose uptake, highlighting the complex interplay between adipose tissue and placenta, in GDM [23]. However, the lack of change in GLUT1 mRNA suggests that EVs may enhance GLUT1 activity and transport capacity without influencing mRNA expression, as demonstrated in other studies [57].

Insulin is a first-line pharmacological treatment for gestational diabetes mellitus (GDM) when lifestyle modifications such as diet and exercise fail to maintain optimal blood glucose levels. Insulin has immunomodulatory effects, inhibiting production of inflammatory cytokines and enhances the release of anti-inflammatory cytokines [58]. It is plausible that the exogenously administrated insulin acts as an agent to reduce inflammation in adipose tissue which conveys signals to other maternal tissues, such as skeletal muscle and placenta *via* EVs to inhibit inflammation. This also reflects that the exogenously administrated insulin in women with GDM may not inhibit the excessive glucose transfer to fetus via placenta, and enhances fetal growth. Pregnancy per se is an inflammatory condition as compared to the non-pregnant state where tightly regulated Th1/Th2 paradigm is an important prerequisite for a successful pregnancy [59]. Thus, the observed increase in inflammatory cytokines and chemokines production in cells treated with EVs derived from NGT adipose tissue is probably reflecting the normal pregnancy physiology.

Interestingly, all the miRNAs of interest in this study downregulated or eliminated the secretion of inflammatory cytokines and chemokines. Previous studies have reported similar observation where miR-146a-5p in mesenchymal cell-derived EVs from amniotic fluid attenuated inflammation in lipopolysaccharide treated placental cell [60] and the overexpression of this miRNA in human endothelial cells inhibited the high glucose- induced inflammation [61]. Although the overexpression of miR-515-5p and miR-516b-5p downregulated the TNF-α induced inflammation, the role of these miRNAs have not been widely studied in relation to inflammation. Thus, detecting the inflammatory target genes of these miRNAs and analyzing their expression in miR-515-5p and miR-516b-5p overexpressed placental cells and in GDM placenta will be essential.

The observed STAT3 activation in GDM placental cells treated with adipose tissue-derived EVs may contribute to insulin resistance and inflammation within the placenta, impacting placental function and nutrient transport to the fetus in GDM [62]. Abnormal TGF-β signaling may lead to impaired trophoblast proliferation and invasion, compromising nutrient and oxygen transport to the fetus and contributing to pregnancy complications [63]. Elevated ROS levels can lead to oxidative damage, impair trophoblast function, and disrupt placental development and nutrient transport, ultimately affecting fetal growth and development in GDM pregnancies [64]. The enrichment of these pathways implicates the role of GDM adipose tissue- derived EVs in regulating GDM pathophysiology. The changes in oxidative phosphorylation associated protein in miR-515-5p treated cells might induce mitochondrial dysfunction and impaired energy production [65]. This can affect trophoblast function and placental development, contributing to adverse pregnancy outcomes, such as fetal growth restriction [66]. Similarly, the observed increased in KRAS signaling in miR-516-5p overexpressed placental cells implicates the potential role of miR-516-5p in impairing trophoblast proliferation and invasion, affecting placental development and function [67]. Overall, the findings suggest that adipose tissue- derived EVs and associated miRNAs play crucial roles in modulating placental function, particularly in the context of nutrient uptake.

In this study, we identified miR-515-5p as a key mediator of the effects observed, as its mimicry replicated the impact of adipose tissue-derived extracellular vesicles Evs from GDM on glucose uptake in primary trophoblast cells. miR-515-5p is a primate-specific microRNA belonging to the C19MC family, and it plays a crucial role in reproduction, development, and differentiation. Previous research has highlighted its differential expression in conditions, such as fetal growth restriction [68] and preterm delivery [69]. The increased expression of miR-515-5p in GDM pregnancies compared to those with NGT raises intriguing questions about its role in various pathological contexts and its divergent effects on metabolic regulation and disease pathophysiology. In preeclampsia, overexpression of miR-515-5p suppresses XIAP (X-linked inhibitor of apoptosis protein) expression [70], promotes apoptosis, and reduces trophoblast migration, suggesting its involvement in placental dysfunction and disease development [68, 71, 72]. Our findings indicate that elevated levels of miR-515-5p correlate with increased fetal size and higher fetal plasma glucose levels, pointing to its potential role in promoting fetal growth and elevated blood glucose levels in GDM.

Although mouse models have been instrumental in advancing our understanding of pregnancy and its outcomes, it is important to acknowledge the differences between human and mouse models as limitations of this study. We found that in the mouse model, placental weight remained unchanged while fetal weight was affected. In contrast, the human cohort showed altered placental weight without a corresponding change in fetal weight. We hypothesize that this discrepancy may be due to species-specific biological mechanisms. In mouse models, the placenta may be less capable of adapting to environmental disruptions, such as glucose intolerance or nutrient deprivation, leading to direct maternal influences on fetal growth without sufficient compensation by the placenta. Conversely, in humans, the placenta seems to possess more robust adaptive mechanisms, allowing it to adjust its size and function in response to changes in the maternal environment. This adaptability may help preserve fetal growth, albeit the altered placental size, suggesting a protective mechanism that prioritizes fetal development. Therefore, the human placenta’s ability to adjust its growth in response to adverse conditions may explain why fetal weight remains unaffected even when placental size changes, a phenomenon less commonly observed in mouse models.

Notably, we observed an increase in glucose uptake by trophoblasts in the presence of miR-515-5p. Our bioinformatic analyses suggest that miR-515-5p may regulate placental glucose uptake by targeting the EMD gene or inhibiting interferon-induced helicase C domain-containing protein-1 (IFIH-1). EMD encodes the protein Emerin, which is involved in regulating insulin-like growth factor (IGF) pathway [73], a key regulator of metabolism [74]. This is in consistent with reported previous study where miR-515-5p regulates IGF1 receptor (IGF1R) function, thereby stimulating glucose absorption [75]. Similarly, a relationship has been found between miR-515-5p expression and maternal BMI during the first trimester [76]. Conversely, IFIF-1 encodes melanoma differentiation-associated protein 5 (MDA5). A previous study reported reduced IFIH-1 expression in the placenta of patients with GDM as compared to that of controls [77]. In this study, the transfection if miR-515-5p significantly downregulated the expression of IFH-1 (-0.3 FC). However, further functional studies are warranted to confirm this finding.

Our in vivo experiments demonstrated that adipose tissue-derived EVs from NGT loaded with miR-515-5p induce fetal overgrowth, reflecting the clinical manifestations of GDM. The observed fetal overgrowth further supports the involvement of adipose tissue-derived EVs and miR-515-5p in regulating placental nutrient uptake during GDM pregnancies.

While the results of this study excitingly demonstrate the potential role of adipose tissue-derived EVs in regulating placental function in in vitro and in vivo pregnancy models, several limitations exist that need to be addressed. This study provides valuable insights into the potential effects of adipose tissue-derived EVs on inflammation, nutrient metabolism in the placenta, and fetal growth. However, it is possible that these EVs may also influence nutrient metabolism and inflammation in other metabolic tissues, such as the liver or skeletal muscle, which could contribute to the cascade of events commonly observed in GDM. Additionally, the specific mechanistic pathways by which miR-515-5p regulates glucose uptake and fetal growth need further investigation. Future studies exploring the molecular targets of these miRNAs in placental and fetal tissues would be beneficial. We are also uncertain whether the injected EVs cross the placenta and directly affect the fetus, suggesting the need for biodistribution studies to determine the precise action of EVs on metabolic tissues, placenta, and fetus. Lastly, the relatively weak effects of miR-515-5p observed in vivo may be attributed to its primate-specific nature, which could limit its relevance in non-primate models and complicate the understanding of its biological role. To address this, future studies could employ alternative models, such as microfluidic technology, which could greatly enhance our understanding of this primate-specific miRNA’s functions and its potential therapeutic applications. Another limitation of this study is the absence of placental area measurements, which would have provided additional insight into placental morphology and its relationship with the observed molecular and functional changes. Future studies should incorporate detailed placental phenotyping to further elucidate the impact of adipose tissue-derived EVs on placental development and function. We acknowledge that one of the limitations of this study is the variability observed in the control group of the in vivo experiments, where two out of four litters exhibited notably reduced fetal weights, despite fetuses being alive and undergoing standard collection procedures. While ROUT analysis was employed to detect outliers and data were adjusted for litter size, the presence of two low-weight litters may have skewed the overall control group mean. As such, it is possible that the observed differences between the control and miR-515-treated groups may, in part, reflect this variability rather than a direct effect of the miRNA itself.

## Electronic supplementary material

Below is the link to the electronic supplementary material.

Supplementary Material 1

Supplementary Material 2

Supplementary Material 3

Supplementary Material 4

Supplementary Material 5

Supplementary Material 6

Supplementary Material 7

Supplementary Material 8

Supplementary Material 9

Supplementary Material 10

Supplementary Material 11

## Data Availability

No datasets were generated or analysed during the current study.

## Abbreviations

BAT: Brown adipose tissue
BMI: Body mass index
EVs: Extracellular vesicles
FBS: Fetal bovine serum
GDM: Gestational diabetes mellitus
miRNAs: MicroRNAs
NGT: Normal glucose tolerance
NTA: Nanoparticle tracking analysis
OGTT: Oral Glucose Tolerance Test
WAT: White adipose tissue
