# Supplementary material for: Extracellular vesicle-associated miR-515-5p from adipose tissue regulates placental metabolism and fetal growth in gestational diabetes mellitus
Source: Cardiovasc Diabetol. 2025 May 14;24:205. doi: 10.1186/s12933-025-02739-z (PMC12080180; doi:10.1186/s12933-025-02739-z)
Supplement: Supplementary file 1 — Supplementary Material 1 [file 12933_2025_2739_MOESM1_ESM.pdf]

Additional information Figures

Extracellular Vesicle-Associated miR-515-5p from Adipose Tissue Regulates Placental metabolism and Fetal Growth in Gestational Diabetes Mellitus

Nanthini Jayabalan*[1], Soumyalekshmi Nair[1,2], Andrew Lai[1,2], Katherin Scholz-Romero[1,2], Melissa Razo-Azamar[1,2], Valeska Ormazabal[3], Ratana Lim[4], Flavio Carrion[5], Dominic Guanzon[1,2], Gregory E Rice[1,6], Harold David McIntyre[7], Martha Lappas[4], Carlos Salomon*[1,2]

[1]Translational Extracellular Vesicles in Obstetrics and Gynae-Oncology Group, University of Queensland Centre for Clinical Research, Faculty of Medicine, The University of Queensland, Brisbane, Queensland 4029, Australia
[2]UQ Centre for Extracellular Vesicle nanomedicine, The University of Queensland, Brisbane, Queensland 4029, Australia
[3]Faculty of Biological Sciences, Pharmacology Department, University of Concepcion, Concepción, Chile
[4]Obstetrics, Nutrition and Endocrinology Group, Department of Obstetrics and Gynaecology, University of Melbourne, Victoria, Australia
[5]Departamento de Investigación, Postgrado y Educación Continua (DIPEC), Facultad de Ciencias de la Salud, Universidad del Alba, Santiago, Chile.
[6]INOVIQ Ltd., Notting Hill, VIC 3168, Australia
[7]Mater Research, Faculty of Medicine, The University of Queensland, Brisbane, Queensland, Australia

***Correspondence:**
Professor Carlos Salomon, PhD, DMedSc, MSc
Director, UQ Centre for Extracellular Vesicle Nanomedicine
Head, Translational Extracellular Vesicles in Obstetrics and Gynae-Oncology Group
UQ Centre for Clinical Research, Faculty of Medicine, The University of Queensland
Phone: +61 7 33465500 | Fax: +61 7 3346 5509 |
e-mail: c.salomongallo@uq.edu.au | Web: www.uqccr.uq.edu.au/

Nanthini Jayabalan, PhD
Translational Extracellular Vesicles in Obstetrics and Gynae-Oncology Group
UQ Centre for Clinical Research, Faculty of Medicine, The University of Queensland
e-mail: n.jayabalan@uq.edu.au

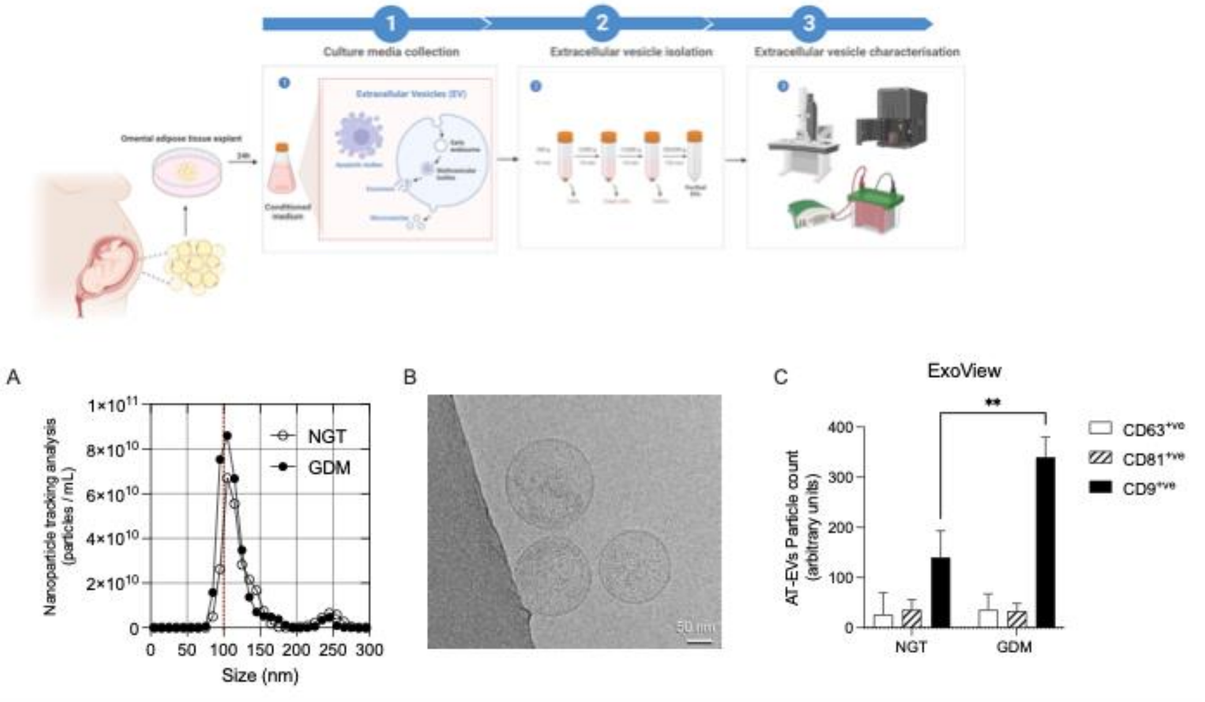

Figure S1. Isolation and characterization of EVs. EVs were isolated from adipose tissue explant media. Omental adipose tissue was obtained from NGT and GDM women and cultured for 24h. EVs were purified from conditioned media and characterised using (A) size distribution, (B) morphology and (C) protein expression using Nanoparticle Tracking analysis (NTA), cryo-electron microscope and ExoView. The NTA analysis shows presence of particles with size distribution of around 100nm with a circular morphology expressing the EV markers, CD63, CD81 and CD9.

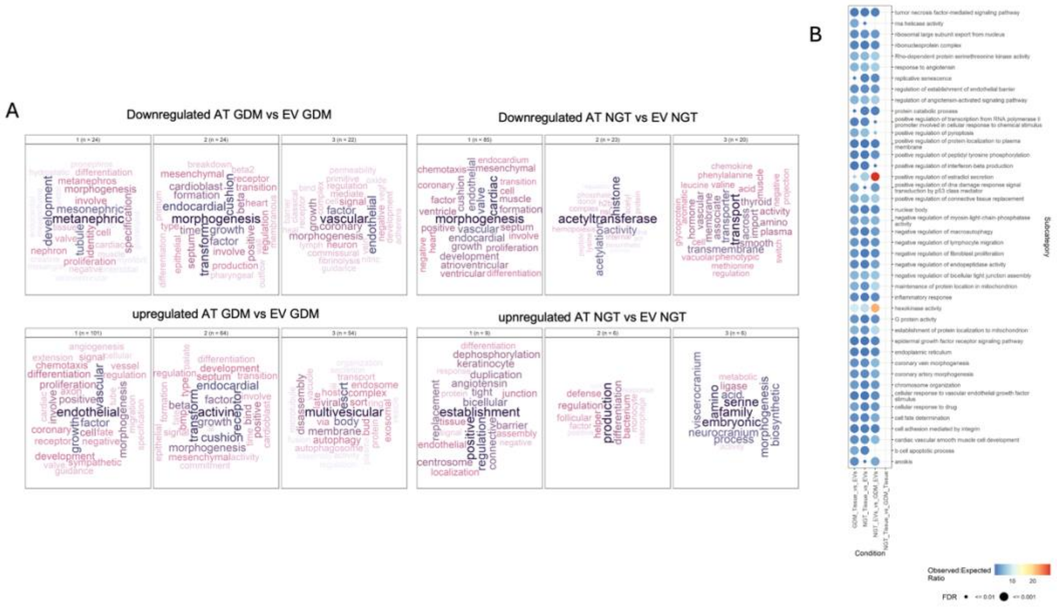

Figure S2. Ontology analysis of miRNA enrichment adipose tissue and EVs in normal and gestational diabetes pregnancy. (A) Comparative analysis of enriched gene ontology biological processes (GO-BP) from the Molecular Signatures Database (MsigDb) generated using vissE for miRNA with 2- fold changes in NGT and GDM adipose tissue and Evs. (B) Differentially enriched pathways by miRNAs. Colour indicates enrichment and the size indicates significance.

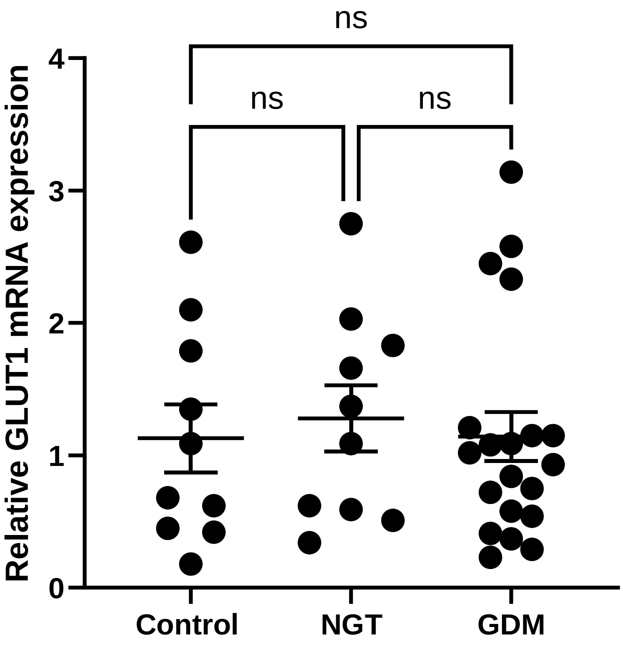

Figure S3. Effects of adipose-tissue derived EVs on placental glucose transporter, GLUT1. No changes were observed in the expression of GLUT1 mRNA between primary human trophoblast cells treated with EVs from NGT or GDM women (n=10- 20 patients). The fold change was calculated relative to Control. Repeated measure one way- ANOVA was performed with Bonferroni's test. Data presented as mean ± SEM.

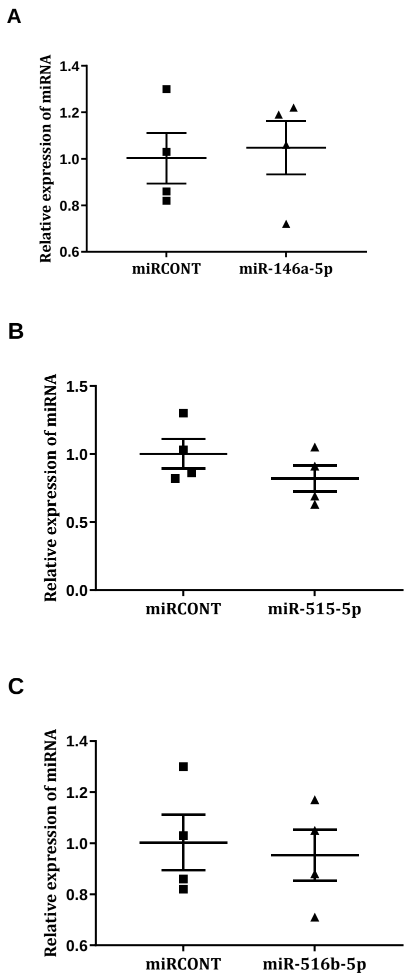

Figure S4. Effects of adipose-tissue derived EVs mimics transfection on placental cell viability. The effect of transfection of mimics A)miR-146a-5p, B) miR-515-5p and C) miR-516b-5p on primary trophoblast cell viability was assessed using MTT assay (n= 4 patients) and the fold change was calculated relative to the miRCONT-transfected cells. Paired T-test was performed. Data are displayed as mean ± SEM.

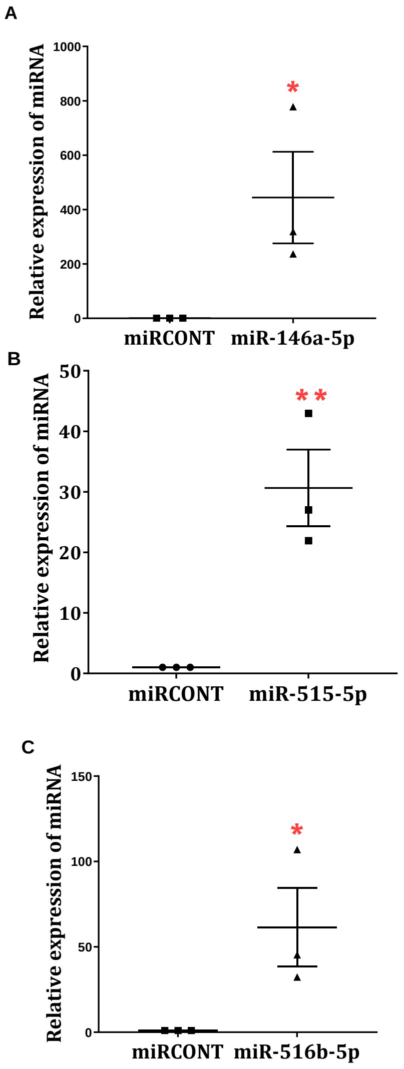

Figure S5. Efficiency of mimics transfection in placental cells. The expression level of A) miR-146a-5p, B) miR-515-5p and C) miR-516b-5p in primary human trophoblast cells transfected with mimic was detected by QRT-PCR (n= 3 patients) and the fold change was calculated relative to the miRCONT-transfected cells. Paired T- test was performed. change was calculated relative to the miRCONT-transfected cells. Paired T-test was performed. Data are displayed as mean ± SEM.

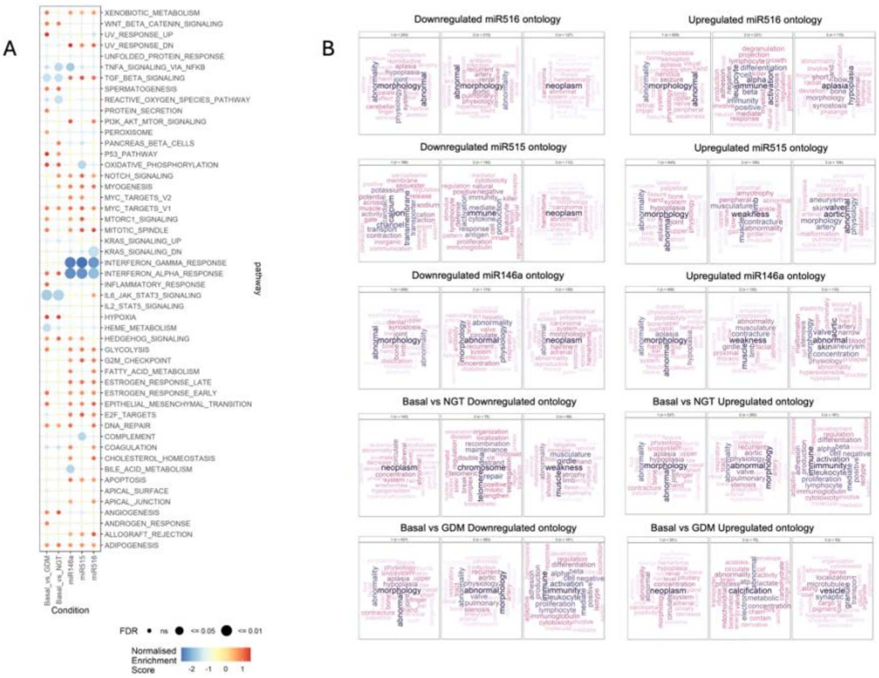

Figure S6. Enrichment of placental pathways associated with metabolism and inflamamtion. (A) Differentially enriched pathways analysis. Colour indicates enrichment and the size indicates significance. (B) Enriched gene ontology biological processes (GO-BP) pathway clusters from the Molecular Signatures Database (MSigDb) generated using vissE in NGT and GDM placental tissues and placental cells transfected with miRNA.

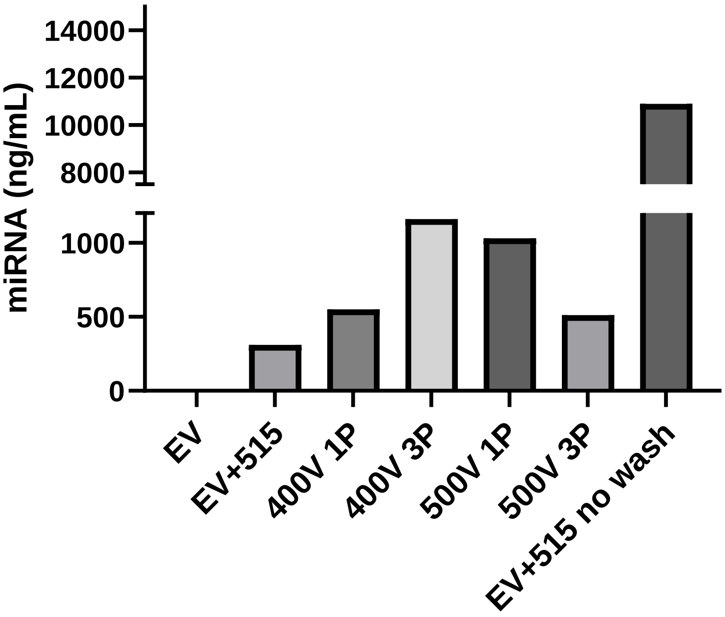

Figure S7. Selection of 400 V 3 pulses as the optimal condition for the loading of EVs with miR-515-5p. EVs alone (EV), mixed with miR-515-5p (EV+515), and subjected to electroporation at various voltages and pulse settings. The miRNA not incorporated into the EVs was removed by washing, followed by small RNA extraction. The amount of miRNA associated with the EVs was quantified using the Qubit microRNA assay.
