# Supplementary material for: Extracellular vesicle-associated miR-515-5p from adipose tissue regulates placental metabolism and fetal growth in gestational diabetes mellitus
Source: Cardiovasc Diabetol. 2025 May 14;24:205. doi: 10.1186/s12933-025-02739-z (PMC12080180; doi:10.1186/s12933-025-02739-z)
Supplement: Supplementary file 2 — Supplementary Material 2 [file 12933_2025_2739_MOESM2_ESM.docx]

**Supplementary Table 1: Table below shows the miRNA differently regulated in EVs derived from GDM adipose tissue compared to EVs derived from NGT adipose tissue.**

| **miRNA** | **log2FoldChange** | **pvalue** |
| --- | --- | --- |
| hsa-miR-145-5p | -3.685587 | 1.30356270095063e-06 |
| hsa-miR-139-5p | -2.773208 | 0.0072 |
| hsa-miR-4485-3p | -2.369849 | 0.0031 |
| hsa-miR-126-5p | -2.249607 | 7.25044702145834e-12 |
| hsa-miR-451a | -2.208677 | 8.2507658313436e-05 |
| hsa-miR-452-5p | -1.991385 | 0.0136 |
| hsa-miR-378d | -1.959951 | 0.0421 |
| hsa-miR-20a-5p | -1.872458 | 0.0478 |
| hsa-miR-93-5p | -1.747312 | 0.0035 |
| hsa-miR-424-5p | -1.623473 | 0.0779 |
| hsa-miR-98-5p | -1.612690 | 0.0007 |
| hsa-miR-31-5p | -1.542036 | 0.1183 |
| hsa-miR-193a-5p | -1.512359 | 0.0930 |
| hsa-miR-143-5p | -1.510844 | 0.0920 |
| hsa-miR-378i | -1.437767 | 0.0016 |
| hsa-miR-378c | -1.405122 | 0.0076 |
| hsa-miR-190a-5p | -1.277300 | 0.1379 |
| hsa-miR-340-5p | -1.168102 | 0.0242 |
| hsa-miR-224-5p | -1.099979 | 0.0988 |
| hsa-miR-192-5p | -1.068742 | 0.0119 |
| hsa-miR-4286 | -1.041666 | 0.2282 |
| hsa-miR-26b-5p | -0.976939 | 3.24210116359851e-05 |
| hsa-miR-106b-5p | -0.938118 | 0.1973 |
| hsa-miR-5701 | -0.885179 | 0.2979 |
| hsa-miR-374b-5p | -0.868036 | 0.2121 |
| hsa-miR-30a-5p | -0.843977 | 2.08632565547857e-06 |
| hsa-let-7g-5p | -0.840349 | 0.0006 |
| hsa-miR-181d-5p | -0.781238 | 0.3187 |
| hsa-miR-10b-5p | -0.754417 | 0.0137 |
| hsa-miR-5683 | -0.738416 | 0.3298 |
| hsa-miR-195-5p | -0.730703 | 0.0381 |
| hsa-miR-140-5p | -0.708526 | 0.2873 |
| hsa-miR-144-5p | -0.701378 | 0.2045 |
| hsa-miR-34a-5p | -0.687342 | 0.2958 |
| hsa-miR-22-5p | -0.680792 | 0.3465 |
| hsa-miR-30e-5p | -0.649955 | 0.0176 |
| hsa-miR-136-5p | -0.631762 | 0.3282 |
| hsa-miR-16-5p | -0.612661 | 0.0215 |
| hsa-miR-142-5p | -0.597899 | 0.1726 |
| hsa-miR-378a-5p | -0.589511 | 0.4568 |
| hsa-miR-181c-5p | -0.561727 | 0.1332 |
| hsa-miR-339-5p | -0.555255 | 0.4818 |
| hsa-miR-15a-5p | -0.531561 | 0.1852 |
| hsa-miR-146a-5p | -0.524115 | 0.2516 |
| hsa-miR-361-5p | -0.518215 | 0.2928 |
| hsa-miR-30b-5p | -0.503082 | 0.1483 |
| hsa-miR-194-5p | -0.502313 | 0.5152 |
| hsa-miR-34c-5p | -0.488683 | 0.5402 |
| hsa-miR-374a-5p | -0.480882 | 0.3745 |
| hsa-miR-15b-5p | -0.475745 | 0.5103 |
| hsa-miR-196b-5p | -0.463563 | 0.4610 |
| hsa-miR-215-5p | -0.444646 | 0.3553 |
| hsa-miR-5585-3p | -0.425772 | 0.6162 |
| hsa-miR-574-5p | -0.414235 | 0.5073 |
| hsa-miR-4284 | -0.407136 | 0.6052 |
| hsa-miR-335-5p | -0.391601 | 0.3288 |
| hsa-miR-324-5p | -0.370313 | 0.6384 |
| hsa-miR-33b-5p | -0.368291 | 0.6414 |
| hsa-miR-12136 | -0.357241 | 0.5443 |
| hsa-miR-497-5p | -0.355693 | 0.2736 |
| hsa-miR-29c-5p | -0.326992 | 0.6796 |
| hsa-miR-32-5p | -0.322937 | 0.5928 |
| hsa-miR-17-5p | -0.310450 | 0.5935 |
| hsa-miR-769-5p | -0.295003 | 0.4407 |
| hsa-miR-589-5p | -0.282137 | 0.7226 |
| hsa-miR-148b-5p | -0.261210 | 0.7406 |
| hsa-miR-532-5p | -0.248388 | 0.5627 |
| hsa-miR-486-5p | -0.223981 | 0.5483 |
| hsa-miR-27b-5p | -0.208088 | 0.7282 |
| hsa-miR-493-5p | -0.192678 | 0.8170 |
| hsa-miR-150-5p | -0.119174 | 0.7027 |
| hsa-miR-101-5p | -0.117483 | 0.8439 |
| hsa-miR-484 | -0.117276 | 0.7816 |
| hsa-miR-30c-5p | -0.093340 | 0.6368 |
| hsa-miR-582-5p | -0.079157 | 0.9239 |
| hsa-miR-185-5p | -0.076483 | 0.9261 |
| hsa-miR-186-5p | -0.051516 | 0.8137 |
| hsa-miR-660-5p | -0.046959 | 0.9156 |
| hsa-miR-548k | -0.045375 | 0.9569 |
| hsa-miR-455-5p | -0.029892 | 0.9514 |
| hsa-miR-3613-5p | -0.026679 | 0.9747 |
| hsa-miR-652-5p | -0.008666 | 0.9919 |
| hsa-let-7b-5p | -0.008236 | 0.9657 |
| hsa-miR-148a-5p | 0.001696 | 0.9969 |
| hsa-miR-28-5p | 0.025568 | 0.9519 |
| hsa-miR-181b-5p | 0.030947 | 0.9076 |
| hsa-miR-218-5p | 0.032205 | 0.9430 |
| hsa-miR-30d-5p | 0.035451 | 0.8322 |
| hsa-miR-628-5p | 0.045790 | 0.9585 |
| hsa-miR-874-5p | 0.053441 | 0.9509 |
| hsa-miR-425-5p | 0.054940 | 0.9019 |
| hsa-miR-33a-5p | 0.078428 | 0.9281 |
| hsa-miR-191-5p | 0.081137 | 0.6967 |
| hsa-miR-653-5p | 0.112787 | 0.8981 |
| hsa-miR-584-5p | 0.129183 | 0.8196 |
| hsa-let-7i-5p | 0.135383 | 0.6601 |
| hsa-miR-152-5p | 0.157623 | 0.8603 |
| hsa-miR-183-5p | 0.158104 | 0.8208 |
| hsa-miR-548ba | 0.167382 | 0.8524 |
| hsa-miR-26a-5p | 0.167873 | 0.3731 |
| hsa-miR-345-5p | 0.175702 | 0.7179 |
| hsa-let-7d-5p | 0.178259 | 0.6219 |
| hsa-miR-21-5p | 0.186991 | 0.5585 |
| hsa-miR-29a-5p | 0.197216 | 0.8282 |
| hsa-miR-338-5p | 0.210981 | 0.8297 |
| hsa-miR-1275 | 0.214598 | 0.7331 |
| hsa-miR-504-5p | 0.223621 | 0.8105 |
| hsa-miR-1296-5p | 0.231794 | 0.7316 |
| hsa-miR-204-5p | 0.235393 | 0.6119 |
| hsa-miR-330-5p | 0.236213 | 0.7982 |
| hsa-miR-511-5p | 0.247672 | 0.7921 |
| hsa-miR-1277-5p | 0.257152 | 0.7842 |
| hsa-miR-377-5p | 0.261655 | 0.7816 |
| hsa-miR-182-5p | 0.265614 | 0.5420 |
| hsa-miR-155-5p | 0.285368 | 0.5248 |
| hsa-miR-1973 | 0.286609 | 0.7631 |
| hsa-miR-18a-5p | 0.290837 | 0.6741 |
| hsa-miR-99b-5p | 0.298244 | 0.2459 |
| hsa-miR-10a-5p | 0.302004 | 0.1810 |
| hsa-miR-561-5p | 0.302013 | 0.7512 |
| hsa-miR-7-5p | 0.307119 | 0.7638 |
| hsa-miR-1179 | 0.313016 | 0.7525 |
| hsa-miR-214-5p | 0.318710 | 0.5304 |
| hsa-miR-1271-5p | 0.332392 | 0.7306 |
| hsa-miR-9-5p | 0.336848 | 0.7264 |
| hsa-miR-10399-3p | 0.347270 | 0.7185 |
| hsa-miR-20b-5p | 0.350025 | 0.7253 |
| hsa-miR-1287-5p | 0.350062 | 0.7211 |
| hsa-miR-450a-5p | 0.382545 | 0.5463 |
| hsa-let-7a-5p | 0.387307 | 0.1851 |
| hsa-miR-188-5p | 0.392014 | 0.6898 |
| hsa-miR-130a-5p | 0.403088 | 0.6979 |
| hsa-miR-29b-2-5p | 0.408591 | 0.6814 |
| hsa-miR-450b-5p | 0.413701 | 0.4689 |
| hsa-miR-154-5p | 0.414774 | 0.6844 |
| hsa-miR-7977 | 0.415468 | 0.4633 |
| hsa-miR-125a-5p | 0.420396 | 0.0388 |
| hsa-miR-223-5p | 0.422705 | 0.6737 |
| hsa-miR-891a-5p | 0.424873 | 0.6856 |
| hsa-miR-134-5p | 0.451007 | 0.4884 |
| hsa-miR-130b-5p | 0.454876 | 0.6506 |
| hsa-miR-2355-5p | 0.463757 | 0.6460 |
| hsa-miR-196a-5p | 0.476797 | 0.5337 |
| hsa-miR-379-5p | 0.484525 | 0.6338 |
| hsa-miR-96-5p | 0.497467 | 0.6374 |
| hsa-miR-181a-5p | 0.498195 | 0.0173 |
| hsa-miR-10399-5p | 0.498633 | 0.6260 |
| hsa-miR-127-5p | 0.499103 | 0.4628 |
| hsa-miR-432-5p | 0.502216 | 0.5219 |
| hsa-miR-24-2-5p | 0.516970 | 0.4159 |
| hsa-miR-376a-5p | 0.519360 | 0.4512 |
| hsa-miR-1260a | 0.528991 | 0.1973 |
| hsa-miR-2114-5p | 0.535851 | 0.6112 |
| hsa-miR-382-5p | 0.540855 | 0.6029 |
| hsa-miR-378g | 0.542756 | 0.6011 |
| hsa-miR-151a-5p | 0.544438 | 0.0856 |
| hsa-miR-199a-5p | 0.548014 | 0.1329 |
| hsa-miR-1291 | 0.549648 | 0.6019 |
| hsa-miR-299-3p | 0.550352 | 0.4431 |
| hsa-miR-651-5p | 0.554248 | 0.5953 |
| hsa-miR-422a | 0.565524 | 0.5903 |
| hsa-miR-629-5p | 0.573990 | 0.5905 |
| hsa-miR-542-5p | 0.600043 | 0.5839 |
| hsa-miR-3913-5p | 0.611683 | 0.5755 |
| hsa-miR-7705 | 0.629818 | 0.5582 |
| hsa-miR-4521 | 0.637458 | 0.5547 |
| hsa-miR-135b-5p | 0.639209 | 0.5593 |
| hsa-miR-99a-5p | 0.652741 | 0.0613 |
| hsa-miR-6504-5p | 0.657384 | 0.5466 |
| hsa-miR-548d-5p | 0.657982 | 0.5451 |
| hsa-miR-873-5p | 0.669559 | 0.5431 |
| hsa-miR-24-1-5p | 0.670836 | 0.5372 |
| hsa-miR-641 | 0.674124 | 0.5393 |
| hsa-miR-2467-5p | 0.674520 | 0.5382 |
| hsa-miR-221-5p | 0.678231 | 0.2366 |
| hsa-miR-671-5p | 0.680306 | 0.5378 |
| hsa-miR-331-5p | 0.681499 | 0.5333 |
| hsa-miR-1260b | 0.688844 | 0.0992 |
| hsa-miR-411-5p | 0.690259 | 0.0822 |
| hsa-miR-1268a | 0.693411 | 0.5353 |
| hsa-miR-501-5p | 0.704945 | 0.5286 |
| hsa-miR-2110 | 0.710520 | 0.5198 |
| hsa-miR-1268b | 0.718522 | 0.5241 |
| hsa-miR-4488 | 0.723201 | 0.5149 |
| hsa-miR-296-5p | 0.726881 | 0.5151 |
| hsa-miR-548c-5p | 0.727991 | 0.5142 |
| hsa-miR-548o-5p | 0.727991 | 0.5142 |
| hsa-miR-365a-5p | 0.732521 | 0.5192 |
| hsa-miR-548am-5p | 0.736094 | 0.5116 |
| hsa-miR-34b-5p | 0.740711 | 0.5060 |
| hsa-miR-503-5p | 0.745326 | 0.5146 |
| hsa-miR-1185-5p | 0.763711 | 0.4968 |
| hsa-miR-210-5p | 0.770648 | 0.5017 |
| hsa-miR-149-5p | 0.780027 | 0.1749 |
| hsa-miR-1468-5p | 0.787937 | 0.3338 |
| hsa-miR-548ay-5p | 0.793550 | 0.4844 |
| hsa-miR-190b-5p | 0.795351 | 0.4845 |
| hsa-miR-25-5p | 0.821457 | 0.4738 |
| hsa-miR-409-5p | 0.823156 | 0.4735 |
| hsa-miR-654-5p | 0.826717 | 0.4715 |
| hsa-miR-211-5p | 0.837034 | 0.4667 |
| hsa-miR-548ae-5p | 0.837566 | 0.4665 |
| hsa-miR-1306-5p | 0.839936 | 0.4665 |
| hsa-miR-100-5p | 0.843557 | 4.0667013980317e-05 |
| hsa-miR-548au-5p | 0.845691 | 0.4647 |
| hsa-miR-642a-5p | 0.848478 | 0.4621 |
| hsa-miR-199b-5p | 0.854738 | 0.0303 |
| hsa-miR-4510 | 0.858669 | 0.4577 |
| hsa-miR-95-5p | 0.871533 | 0.4522 |
| hsa-miR-18b-5p | 0.876513 | 0.4506 |
| hsa-miR-454-5p | 0.878966 | 0.4491 |
| hsa-miR-627-5p | 0.879449 | 0.4492 |
| hsa-miR-499a-5p | 0.880150 | 0.4490 |
| hsa-miR-103a-2-5p | 0.886115 | 0.4462 |
| hsa-miR-659-5p | 0.893396 | 0.4429 |
| hsa-miR-212-5p | 0.894663 | 0.4423 |
| hsa-miR-6761-5p | 0.902067 | 0.4390 |
| hsa-miR-3195 | 0.906855 | 0.1853 |
| hsa-miR-4326 | 0.906967 | 0.4368 |
| hsa-miR-6503-5p | 0.909680 | 0.4356 |
| hsa-miR-675-5p | 0.912230 | 0.4345 |
| hsa-miR-135a-5p | 0.917290 | 0.4322 |
| hsa-miR-125b-5p | 0.920765 | 0.0339 |
| hsa-miR-4531 | 0.924016 | 0.4292 |
| hsa-miR-146b-5p | 0.924139 | 0.0029 |
| hsa-miR-548ad-5p | 0.933332 | 0.4252 |
| hsa-miR-337-5p | 0.934594 | 0.4246 |
| hsa-miR-576-5p | 0.935270 | 0.2611 |
| hsa-miR-299-5p | 0.937247 | 0.4234 |
| hsa-miR-1270 | 0.945436 | 0.4199 |
| hsa-miR-3605-5p | 0.955376 | 0.4156 |
| hsa-miR-500a-5p | 0.966739 | 0.4108 |
| hsa-miR-193b-5p | 0.974000 | 0.2350 |
| hsa-miR-942-5p | 0.974148 | 0.4077 |
| hsa-miR-138-5p | 0.978764 | 0.4057 |
| hsa-miR-29b-1-5p | 0.981523 | 0.4046 |
| hsa-miR-548h-5p | 0.990751 | 0.4008 |
| hsa-miR-502-5p | 0.998607 | 0.3975 |
| hsa-miR-6511b-5p | 1.008422 | 0.3935 |
| hsa-miR-4662a-5p | 1.020137 | 0.2181 |
| hsa-let-7f-5p | 1.020627 | 0.0008 |
| hsa-miR-3065-5p | 1.023001 | 0.3876 |
| hsa-miR-369-5p | 1.030049 | 0.2155 |
| hsa-miR-23a-5p | 1.035829 | 0.3825 |
| hsa-miR-378b | 1.039753 | 0.3810 |
| hsa-miR-92a-1-5p | 1.039753 | 0.3810 |
| hsa-miR-545-5p | 1.041550 | 0.3803 |
| hsa-miR-548e-5p | 1.052352 | 0.3760 |
| hsa-miR-548aj-5p | 1.053559 | 0.3756 |
| hsa-miR-23b-5p | 1.055628 | 0.3748 |
| hsa-miR-1908-5p | 1.060812 | 0.3727 |
| hsa-miR-1261 | 1.062738 | 0.3720 |
| hsa-miR-4485-5p | 1.079719 | 0.3655 |
| hsa-miR-219a-5p | 1.081777 | 0.3647 |
| hsa-miR-548f-5p | 1.084952 | 0.3635 |
| hsa-miR-616-5p | 1.086851 | 0.3628 |
| hsa-miR-585-5p | 1.097033 | 0.3590 |
| hsa-let-7c-5p | 1.107566 | 0.0069 |
| hsa-miR-548ar-5p | 1.118770 | 0.3510 |
| hsa-miR-3928-3p | 1.123664 | 0.3492 |
| hsa-miR-548w | 1.149251 | 0.3400 |
| hsa-miR-618 | 1.149251 | 0.3400 |
| hsa-miR-3622a-5p | 1.155390 | 0.3379 |
| hsa-miR-491-5p | 1.160711 | 0.3360 |
| hsa-miR-92b-5p | 1.164266 | 0.3348 |
| hsa-miR-664a-5p | 1.172333 | 0.1693 |
| hsa-miR-500b-5p | 1.173311 | 0.3316 |
| hsa-miR-4767 | 1.185609 | 0.3274 |
| hsa-miR-624-5p | 1.204972 | 0.3209 |
| hsa-miR-346 | 1.211505 | 0.3187 |
| hsa-miR-5699-5p | 1.211683 | 0.3187 |
| hsa-miR-4455 | 1.214859 | 0.3176 |
| hsa-miR-4772-5p | 1.218258 | 0.3165 |
| hsa-miR-548az-5p | 1.218258 | 0.3165 |
| hsa-miR-548b-5p | 1.218258 | 0.3165 |
| hsa-miR-548i | 1.224955 | 0.3143 |
| hsa-miR-708-5p | 1.226880 | 0.0596 |
| hsa-miR-10396b-3p | 1.231839 | 0.3120 |
| hsa-miR-412-5p | 1.235437 | 0.3109 |
| hsa-miR-548ab | 1.249700 | 0.3063 |
| hsa-miR-548ag | 1.253322 | 0.3051 |
| hsa-miR-548q | 1.253486 | 0.3051 |
| hsa-miR-200a-5p | 1.267744 | 0.3006 |
| hsa-miR-371a-5p | 1.268316 | 0.3004 |
| hsa-miR-362-5p | 1.271487 | 0.1729 |
| hsa-miR-3059-5p | 1.271726 | 0.2993 |
| hsa-miR-548aq-3p | 1.271726 | 0.2993 |
| hsa-miR-550a-3-5p | 1.275617 | 0.2981 |
| hsa-miR-550a-5p | 1.275617 | 0.2981 |
| hsa-miR-216a-5p | 1.275772 | 0.2981 |
| hsa-miR-378h | 1.275772 | 0.2981 |
| hsa-miR-4524a-5p | 1.275772 | 0.2981 |
| hsa-miR-5690 | 1.306873 | 0.2886 |
| hsa-miR-1273h-5p | 1.311366 | 0.2872 |
| hsa-miR-431-5p | 1.315912 | 0.1640 |
| hsa-miR-4497 | 1.336598 | 0.2797 |
| hsa-miR-370-5p | 1.349632 | 0.2760 |
| hsa-miR-132-5p | 1.350565 | 0.1534 |
| hsa-miR-3934-5p | 1.354655 | 0.2745 |
| hsa-miR-625-5p | 1.366133 | 0.1642 |
| hsa-miR-129-5p | 1.373708 | 0.2691 |
| hsa-miR-505-5p | 1.376829 | 0.2683 |
| hsa-miR-548n | 1.396655 | 0.2628 |
| hsa-miR-663a | 1.396718 | 0.2627 |
| hsa-miR-1262 | 1.411462 | 0.2587 |
| hsa-miR-10527-5p | 1.417656 | 0.2571 |
| hsa-miR-605-5p | 1.433115 | 0.2530 |
| hsa-miR-539-5p | 1.442258 | 0.2506 |
| hsa-miR-3677-3p | 1.449046 | 0.2488 |
| hsa-miR-590-5p | 1.449046 | 0.2488 |
| hsa-miR-548j-5p | 1.456035 | 0.2470 |
| hsa-miR-1248 | 1.458340 | 0.2464 |
| hsa-miR-217-5p | 1.465448 | 0.2446 |
| hsa-miR-3199 | 1.465448 | 0.2446 |
| hsa-miR-6724-5p | 1.482373 | 0.2403 |
| hsa-miR-525-5p | 1.490133 | 0.2384 |
| hsa-miR-3157-5p | 1.508050 | 0.2340 |
| hsa-miR-153-5p | 1.517781 | 0.2316 |
| hsa-miR-3612 | 1.526446 | 0.2295 |
| hsa-miR-6501-5p | 1.526446 | 0.2295 |
| hsa-miR-888-5p | 1.526446 | 0.2295 |
| hsa-miR-4661-5p | 1.536569 | 0.2271 |
| hsa-miR-1266-5p | 1.545596 | 0.2250 |
| hsa-miR-6126 | 1.545596 | 0.2250 |
| hsa-miR-3174 | 1.565208 | 0.2204 |
| hsa-miR-4636 | 1.565208 | 0.2204 |
| hsa-miR-200b-5p | 1.565437 | 0.2204 |
| hsa-miR-365b-5p | 1.571212 | 0.1028 |
| hsa-miR-5002-5p | 1.575368 | 0.2181 |
| hsa-miR-548an | 1.575368 | 0.2181 |
| hsa-miR-3690 | 1.575631 | 0.2181 |
| hsa-miR-6882-5p | 1.585448 | 0.2159 |
| hsa-miR-1276 | 1.585729 | 0.2158 |
| hsa-miR-885-5p | 1.585729 | 0.2158 |
| hsa-miR-6868-3p | 1.606680 | 0.2111 |
| hsa-miR-9902 | 1.606680 | 0.2111 |
| hsa-miR-1843 | 1.606826 | 0.0536 |
| hsa-miR-19a-5p | 1.607021 | 0.2111 |
| hsa-miR-3127-5p | 1.607021 | 0.2111 |
| hsa-miR-3142 | 1.607021 | 0.2111 |
| hsa-miR-4999-5p | 1.607021 | 0.2111 |
| hsa-miR-3136-5p | 1.618219 | 0.2086 |
| hsa-miR-526b-5p | 1.618219 | 0.2086 |
| hsa-miR-577 | 1.618219 | 0.2086 |
| hsa-miR-6514-5p | 1.618219 | 0.2086 |
| hsa-miR-4791 | 1.641078 | 0.2037 |
| hsa-let-7e-5p | 1.651033 | 0.0004 |
| hsa-miR-6859-5p | 1.664887 | 0.1987 |
| hsa-miR-197-5p | 1.675452 | 0.1966 |
| hsa-miR-3147 | 1.689072 | 0.1938 |
| hsa-miR-5696 | 1.689072 | 0.1938 |
| hsa-miR-6852-5p | 1.689072 | 0.1938 |
| hsa-miR-6866-5p | 1.689072 | 0.1938 |
| hsa-miR-889-5p | 1.689072 | 0.1938 |
| hsa-miR-6516-5p | 1.700247 | 0.1916 |
| hsa-miR-378j | 1.714123 | 0.1888 |
| hsa-miR-1228-5p | 1.714871 | 0.1887 |
| hsa-miR-3130-5p | 1.714871 | 0.1887 |
| hsa-miR-4746-5p | 1.714871 | 0.1887 |
| hsa-miR-6892-5p | 1.741815 | 0.1835 |
| hsa-miR-10396a-3p | 1.758602 | 0.1803 |
| hsa-miR-5588-5p | 1.758602 | 0.1803 |
| hsa-miR-3202 | 1.768962 | 0.1784 |
| hsa-miR-4508 | 1.780851 | 0.0608 |
| hsa-miR-744-5p | 1.783755 | 0.0054 |
| hsa-miR-3152-5p | 1.788187 | 0.1749 |
| hsa-miR-6499-5p | 1.788187 | 0.1749 |
| hsa-miR-6820-5p | 1.788187 | 0.1749 |
| hsa-miR-320d | 1.793991 | 0.0846 |
| hsa-miR-3163 | 1.798270 | 0.1731 |
| hsa-miR-3194-5p | 1.798270 | 0.1731 |
| hsa-miR-4638-3p | 1.798270 | 0.1731 |
| hsa-miR-487a-5p | 1.798270 | 0.1731 |
| hsa-miR-10393-3p | 1.817917 | 0.1696 |
| hsa-miR-1292-5p | 1.817917 | 0.1696 |
| hsa-miR-548aw | 1.817917 | 0.1696 |
| hsa-miR-222-5p | 1.825106 | 0.0798 |
| hsa-miR-106a-5p | 1.827573 | 0.0856 |
| hsa-miR-6513-5p | 1.828949 | 0.1677 |
| hsa-miR-11400 | 1.850374 | 0.1640 |
| hsa-miR-301a-5p | 1.850374 | 0.1640 |
| hsa-miR-4665-5p | 1.850374 | 0.1640 |
| hsa-miR-5009-5p | 1.850374 | 0.1640 |
| hsa-miR-6847-5p | 1.850374 | 0.1640 |
| hsa-miR-877-5p | 1.864243 | 0.0233 |
| hsa-miR-4443 | 1.882706 | 0.1586 |
| hsa-miR-1226-5p | 1.884526 | 0.1583 |
| hsa-miR-202-5p | 1.884526 | 0.1583 |
| hsa-miR-3129-5p | 1.884526 | 0.1583 |
| hsa-miR-371b-5p | 1.884526 | 0.1583 |
| hsa-miR-548at-5p | 1.884526 | 0.1583 |
| hsa-miR-6770-3p | 1.884526 | 0.1583 |
| hsa-miR-6894-3p | 1.884526 | 0.1583 |
| hsa-miR-3620-5p | 1.918373 | 0.1528 |
| hsa-miR-3661 | 1.918373 | 0.1528 |
| hsa-miR-4768-5p | 1.918373 | 0.1528 |
| hsa-miR-4800-5p | 1.918373 | 0.1528 |
| hsa-miR-548t-5p | 1.918373 | 0.1528 |
| hsa-miR-3180 | 1.949121 | 0.1481 |
| hsa-miR-3180-3p | 1.949121 | 0.1481 |
| hsa-miR-4705 | 1.949121 | 0.1481 |
| hsa-miR-4732-5p | 1.949121 | 0.1481 |
| hsa-miR-548ak | 1.949121 | 0.1481 |
| hsa-miR-5706 | 1.949121 | 0.1481 |
| hsa-miR-6839-5p | 1.949121 | 0.1481 |
| hsa-miR-1252-5p | 1.955936 | 0.1470 |
| hsa-miR-1537-5p | 1.955936 | 0.1470 |
| hsa-miR-548ai | 1.955936 | 0.1470 |
| hsa-miR-551b-5p | 1.955936 | 0.1470 |
| hsa-miR-570-5p | 1.955936 | 0.1470 |
| hsa-miR-664b-5p | 1.955936 | 0.1470 |
| hsa-miR-6734-5p | 1.955936 | 0.1470 |
| hsa-miR-6813-5p | 1.955936 | 0.1470 |
| hsa-miR-4786-5p | 1.995521 | 0.1411 |
| hsa-miR-921 | 1.995521 | 0.1411 |
| hsa-miR-10392-5p | 2.030553 | 0.1361 |
| hsa-miR-1233-5p | 2.030553 | 0.1361 |
| hsa-miR-19b-1-5p | 2.030553 | 0.1361 |
| hsa-miR-216b-5p | 2.030553 | 0.1361 |
| hsa-miR-3200-5p | 2.030553 | 0.1361 |
| hsa-miR-449b-5p | 2.030553 | 0.1361 |
| hsa-miR-4794 | 2.030553 | 0.1361 |
| hsa-miR-5584-5p | 2.030553 | 0.1361 |
| hsa-miR-6508-3p | 2.030553 | 0.1361 |
| hsa-miR-6838-5p | 2.030553 | 0.1361 |
| hsa-miR-6858-5p | 2.030553 | 0.1361 |
| hsa-miR-8059 | 2.030553 | 0.1361 |
| hsa-miR-141-5p | 2.076473 | 0.1298 |
| hsa-miR-3133 | 2.076473 | 0.1298 |
| hsa-miR-380-5p | 2.076473 | 0.1298 |
| hsa-miR-4424 | 2.076473 | 0.1298 |
| hsa-miR-4435 | 2.076473 | 0.1298 |
| hsa-miR-4634 | 2.076473 | 0.1298 |
| hsa-miR-4709-5p | 2.076473 | 0.1298 |
| hsa-miR-5187-5p | 2.076473 | 0.1298 |
| hsa-miR-548l | 2.076473 | 0.1298 |
| hsa-miR-376a-2-5p | 2.117210 | 0.1245 |
| hsa-miR-3926 | 2.117210 | 0.1245 |
| hsa-miR-4478 | 2.117210 | 0.1245 |
| hsa-miR-4770 | 2.117210 | 0.1245 |
| hsa-miR-5001-5p | 2.117210 | 0.1245 |
| hsa-miR-5091 | 2.117210 | 0.1245 |
| hsa-miR-5708 | 2.117210 | 0.1245 |
| hsa-miR-6894-5p | 2.117210 | 0.1245 |
| hsa-miR-103a-1-5p | 2.160412 | 0.1190 |
| hsa-miR-2116-5p | 2.160412 | 0.1190 |
| hsa-miR-3679-5p | 2.160412 | 0.1190 |
| hsa-miR-3916 | 2.160412 | 0.1190 |
| hsa-miR-433-5p | 2.160412 | 0.1190 |
| hsa-miR-449a | 2.160412 | 0.1190 |
| hsa-miR-4647 | 2.160412 | 0.1190 |
| hsa-miR-4677-5p | 2.160412 | 0.1190 |
| hsa-miR-4740-5p | 2.160412 | 0.1190 |
| hsa-miR-4749-5p | 2.160412 | 0.1190 |
| hsa-miR-5700 | 2.160412 | 0.1190 |
| hsa-miR-6511a-5p | 2.160412 | 0.1190 |
| hsa-miR-6735-5p | 2.160412 | 0.1190 |
| hsa-miR-6818-5p | 2.160412 | 0.1190 |
| hsa-miR-6829-5p | 2.160412 | 0.1190 |
| hsa-miR-1243 | 2.206674 | 0.1135 |
| hsa-miR-2681-5p | 2.206674 | 0.1135 |
| hsa-miR-3173-5p | 2.206674 | 0.1135 |
| hsa-miR-3175 | 2.206674 | 0.1135 |
| hsa-miR-383-5p | 2.206674 | 0.1135 |
| hsa-miR-4317 | 2.206674 | 0.1135 |
| hsa-miR-6505-5p | 2.206674 | 0.1135 |
| hsa-miR-939-5p | 2.206674 | 0.1135 |
| hsa-miR-2277-5p | 2.230465 | 0.0576 |
| hsa-miR-1285-5p | 2.369859 | 0.0397 |
| hsa-miR-887-5p | 2.395555 | 0.0413 |
| hsa-miR-128-1-5p | 2.428089 | 0.0452 |
| hsa-miR-122b-5p | 2.508631 | 0.0831 |
| hsa-miR-1269b | 2.508631 | 0.0831 |
| hsa-miR-1302 | 2.508631 | 0.0831 |
| hsa-miR-1909-5p | 2.508631 | 0.0831 |
| hsa-miR-219b-5p | 2.508631 | 0.0831 |
| hsa-miR-3120-5p | 2.508631 | 0.0831 |
| hsa-miR-3124-5p | 2.508631 | 0.0831 |
| hsa-miR-3135b | 2.508631 | 0.0831 |
| hsa-miR-323a-5p | 2.508631 | 0.0831 |
| hsa-miR-329-5p | 2.508631 | 0.0831 |
| hsa-miR-3680-5p | 2.508631 | 0.0831 |
| hsa-miR-3685 | 2.508631 | 0.0831 |
| hsa-miR-3942-5p | 2.508631 | 0.0831 |
| hsa-miR-4288 | 2.508631 | 0.0831 |
| hsa-miR-4423-5p | 2.508631 | 0.0831 |
| hsa-miR-4446-5p | 2.508631 | 0.0831 |
| hsa-miR-4463 | 2.508631 | 0.0831 |
| hsa-miR-4517 | 2.508631 | 0.0831 |
| hsa-miR-4529-5p | 2.508631 | 0.0831 |
| hsa-miR-4649-5p | 2.508631 | 0.0831 |
| hsa-miR-4667-5p | 2.508631 | 0.0831 |
| hsa-miR-4680-5p | 2.508631 | 0.0831 |
| hsa-miR-4750-5p | 2.508631 | 0.0831 |
| hsa-miR-4776-5p | 2.508631 | 0.0831 |
| hsa-miR-5010-5p | 2.508631 | 0.0831 |
| hsa-miR-508-5p | 2.508631 | 0.0831 |
| hsa-miR-510-5p | 2.508631 | 0.0831 |
| hsa-miR-513c-5p | 2.508631 | 0.0831 |
| hsa-miR-518c-5p | 2.508631 | 0.0831 |
| hsa-miR-520d-5p | 2.508631 | 0.0831 |
| hsa-miR-524-5p | 2.508631 | 0.0831 |
| hsa-miR-5581-3p | 2.508631 | 0.0831 |
| hsa-miR-567 | 2.508631 | 0.0831 |
| hsa-miR-579-5p | 2.508631 | 0.0831 |
| hsa-miR-597-5p | 2.508631 | 0.0831 |
| hsa-miR-610 | 2.508631 | 0.0831 |
| hsa-miR-6506-5p | 2.508631 | 0.0831 |
| hsa-miR-6730-5p | 2.508631 | 0.0831 |
| hsa-miR-6811-3p | 2.508631 | 0.0831 |
| hsa-miR-6815-5p | 2.508631 | 0.0831 |
| hsa-miR-6852-3p | 2.508631 | 0.0831 |
| hsa-miR-6877-5p | 2.508631 | 0.0831 |
| hsa-miR-7151-3p | 2.508631 | 0.0831 |
| hsa-miR-758-5p | 2.508631 | 0.0831 |
| hsa-miR-7845-5p | 2.508631 | 0.0831 |
| hsa-miR-7976 | 2.508631 | 0.0831 |
| hsa-miR-101-2-5p | 2.508631 | 0.0831 |
| hsa-miR-1256 | 2.508631 | 0.0831 |
| hsa-miR-1284 | 2.508631 | 0.0831 |
| hsa-miR-1298-5p | 2.508631 | 0.0831 |
| hsa-miR-133a-5p | 2.508631 | 0.0831 |
| hsa-miR-1914-5p | 2.508631 | 0.0831 |
| hsa-miR-1915-5p | 2.508631 | 0.0831 |
| hsa-miR-3135a | 2.508631 | 0.0831 |
| hsa-miR-3150a-5p | 2.508631 | 0.0831 |
| hsa-miR-320a-5p | 2.508631 | 0.0831 |
| hsa-miR-3616-5p | 2.508631 | 0.0831 |
| hsa-miR-3674 | 2.508631 | 0.0831 |
| hsa-miR-376b-5p | 2.508631 | 0.0831 |
| hsa-miR-376c-5p | 2.508631 | 0.0831 |
| hsa-miR-3976 | 2.508631 | 0.0831 |
| hsa-miR-4280 | 2.508631 | 0.0831 |
| hsa-miR-4426 | 2.508631 | 0.0831 |
| hsa-miR-4433b-5p | 2.508631 | 0.0831 |
| hsa-miR-4474-5p | 2.508631 | 0.0831 |
| hsa-miR-4505 | 2.508631 | 0.0831 |
| hsa-miR-4679 | 2.508631 | 0.0831 |
| hsa-miR-4722-5p | 2.508631 | 0.0831 |
| hsa-miR-4731-5p | 2.508631 | 0.0831 |
| hsa-miR-4755-5p | 2.508631 | 0.0831 |
| hsa-miR-4758-5p | 2.508631 | 0.0831 |
| hsa-miR-4796-5p | 2.508631 | 0.0831 |
| hsa-miR-4804-5p | 2.508631 | 0.0831 |
| hsa-miR-518e-5p | 2.508631 | 0.0831 |
| hsa-miR-519a-5p | 2.508631 | 0.0831 |
| hsa-miR-519b-5p | 2.508631 | 0.0831 |
| hsa-miR-519c-5p | 2.508631 | 0.0831 |
| hsa-miR-522-5p | 2.508631 | 0.0831 |
| hsa-miR-523-5p | 2.508631 | 0.0831 |
| hsa-miR-548ar-3p | 2.508631 | 0.0831 |
| hsa-miR-580-5p | 2.508631 | 0.0831 |
| hsa-miR-6069 | 2.508631 | 0.0831 |
| hsa-miR-612 | 2.508631 | 0.0831 |
| hsa-miR-6510-5p | 2.508631 | 0.0831 |
| hsa-miR-656-5p | 2.508631 | 0.0831 |
| hsa-miR-6720-5p | 2.508631 | 0.0831 |
| hsa-miR-6741-5p | 2.508631 | 0.0831 |
| hsa-miR-6780a-5p | 2.508631 | 0.0831 |
| hsa-miR-6809-5p | 2.508631 | 0.0831 |
| hsa-miR-6822-3p | 2.508631 | 0.0831 |
| hsa-miR-6826-5p | 2.508631 | 0.0831 |
| hsa-miR-6834-5p | 2.508631 | 0.0831 |
| hsa-miR-6875-5p | 2.508631 | 0.0831 |
| hsa-miR-6888-5p | 2.508631 | 0.0831 |
| hsa-miR-7107-5p | 2.508631 | 0.0831 |
| hsa-miR-7162-5p | 2.508631 | 0.0831 |
| hsa-miR-10400-3p | 2.508631 | 0.0831 |
| hsa-miR-12116 | 2.508631 | 0.0831 |
| hsa-miR-12121 | 2.508631 | 0.0831 |
| hsa-miR-1245b-5p | 2.508631 | 0.0831 |
| hsa-miR-1255a | 2.508631 | 0.0831 |
| hsa-miR-1304-5p | 2.508631 | 0.0831 |
| hsa-miR-3126-5p | 2.508631 | 0.0831 |
| hsa-miR-3159 | 2.508631 | 0.0831 |
| hsa-miR-3161 | 2.508631 | 0.0831 |
| hsa-miR-3193 | 2.508631 | 0.0831 |
| hsa-miR-3681-5p | 2.508631 | 0.0831 |
| hsa-miR-3691-5p | 2.508631 | 0.0831 |
| hsa-miR-374c-5p | 2.508631 | 0.0831 |
| hsa-miR-3918 | 2.508631 | 0.0831 |
| hsa-miR-4270 | 2.508631 | 0.0831 |
| hsa-miR-4297 | 2.508631 | 0.0831 |
| hsa-miR-4298 | 2.508631 | 0.0831 |
| hsa-miR-4328 | 2.508631 | 0.0831 |
| hsa-miR-4458 | 2.508631 | 0.0831 |
| hsa-miR-4536-5p | 2.508631 | 0.0831 |
| hsa-miR-4668-5p | 2.508631 | 0.0831 |
| hsa-miR-4681 | 2.508631 | 0.0831 |
| hsa-miR-4686 | 2.508631 | 0.0831 |
| hsa-miR-4690-3p | 2.508631 | 0.0831 |
| hsa-miR-4701-5p | 2.508631 | 0.0831 |
| hsa-miR-4725-5p | 2.508631 | 0.0831 |
| hsa-miR-4745-3p | 2.508631 | 0.0831 |
| hsa-miR-4745-5p | 2.508631 | 0.0831 |
| hsa-miR-4757-5p | 2.508631 | 0.0831 |
| hsa-miR-4762-5p | 2.508631 | 0.0831 |
| hsa-miR-494-5p | 2.508631 | 0.0831 |
| hsa-miR-509-3-5p | 2.508631 | 0.0831 |
| hsa-miR-513b-5p | 2.508631 | 0.0831 |
| hsa-miR-514a-5p | 2.508631 | 0.0831 |
| hsa-miR-548ap-5p | 2.508631 | 0.0831 |
| hsa-miR-548ax | 2.508631 | 0.0831 |
| hsa-miR-5580-5p | 2.508631 | 0.0831 |
| hsa-miR-592 | 2.508631 | 0.0831 |
| hsa-miR-6500-5p | 2.508631 | 0.0831 |
| hsa-miR-6515-5p | 2.508631 | 0.0831 |
| hsa-miR-6718-5p | 2.508631 | 0.0831 |
| hsa-miR-6733-5p | 2.508631 | 0.0831 |
| hsa-miR-6740-5p | 2.508631 | 0.0831 |
| hsa-miR-6774-5p | 2.508631 | 0.0831 |
| hsa-miR-6827-3p | 2.508631 | 0.0831 |
| hsa-miR-6836-5p | 2.508631 | 0.0831 |
| hsa-miR-6840-5p | 2.508631 | 0.0831 |
| hsa-miR-6842-5p | 2.508631 | 0.0831 |
| hsa-miR-6862-5p | 2.508631 | 0.0831 |
| hsa-miR-6877-3p | 2.508631 | 0.0831 |
| hsa-miR-6886-3p | 2.508631 | 0.0831 |
| hsa-miR-7113-3p | 2.508631 | 0.0831 |
| hsa-miR-556-5p | 2.703249 | 0.0633 |
| hsa-miR-4436b-5p | 2.706782 | 0.0680 |
| hsa-miR-6502-5p | 2.747178 | 0.0569 |
| hsa-miR-4775 | 2.800750 | 0.0235 |
| hsa-miR-3614-5p | 2.897090 | 0.0211 |
| hsa-miR-548g-5p | 2.913275 | 0.0238 |
| hsa-miR-1250-5p | 3.039638 | 0.0484 |
| hsa-miR-6746-5p | 3.039638 | 0.0484 |
| hsa-miR-10401-3p | 3.044829 | 0.0258 |
| hsa-miR-9985 | 3.096953 | 1.38630298133006e-11 |
| hsa-miR-1910-5p | 3.106356 | 0.0452 |
| hsa-miR-4511 | 3.106356 | 0.0452 |
| hsa-miR-548x-5p | 3.151668 | 0.0173 |
| hsa-miR-3074-5p | 3.333684 | 0.0178 |
| hsa-miR-342-5p | 3.335358 | 5.35684808675366e-05 |
| hsa-miR-4454 | 3.396264 | 0.0054 |
| hsa-miR-483-5p | 3.471082 | 0.0033 |
| hsa-miR-5689 | 3.483092 | 0.0265 |
| hsa-miR-27a-5p | 3.488114 | 0.0001 |
| hsa-miR-3160-5p | 3.607464 | 0.0270 |
| hsa-miR-3168 | 3.607464 | 0.0270 |
| hsa-miR-3668 | 3.607464 | 0.0270 |
| hsa-miR-6767-5p | 3.607464 | 0.0270 |
| hsa-miR-1246 | 3.627442 | 0.0270 |
| hsa-miR-6773-5p | 3.680403 | 0.0251 |
| hsa-miR-770-5p | 3.768250 | 0.0213 |
| hsa-miR-1323 | 3.886910 | 0.0098 |
| hsa-miR-4289 | 3.972051 | 0.0190 |
| hsa-miR-1469 | 3.996898 | 0.0181 |
| hsa-miR-4296 | 3.996898 | 0.0181 |
| hsa-miR-4539 | 3.996898 | 0.0181 |
| hsa-miR-519d-5p | 3.996898 | 0.0181 |
| hsa-miR-519e-5p | 3.996898 | 0.0181 |
| hsa-miR-527 | 3.996898 | 0.0181 |
| hsa-miR-6787-5p | 3.996898 | 0.0181 |
| hsa-miR-6869-5p | 4.009844 | 0.0182 |
| hsa-miR-4718 | 4.053584 | 0.0170 |
| hsa-miR-423-5p | 4.218023 | 3.60033061080536e-12 |
| hsa-miR-619-5p | 4.351879 | 5.30130494526577e-06 |
| hsa-miR-4466 | 4.359311 | 0.0099 |
| hsa-miR-10400-5p | 4.516660 | 3.11787179046756e-05 |
| hsa-miR-519a-2-5p | 4.574152 | 0.0078 |
| hsa-miR-520b-5p | 4.574152 | 0.0078 |
| hsa-miR-4787-5p | 4.719672 | 7.5371512685418e-06 |
| hsa-miR-485-5p | 4.729870 | 4.36029102670569e-06 |
| hsa-miR-3196 | 4.861810 | 0.0003 |
| hsa-miR-4430 | 5.256911 | 0.0008 |
| hsa-miR-3178 | 5.295959 | 4.70368895208695e-05 |
| hsa-miR-518a-5p | 5.424237 | 0.0038 |
| hsa-miR-7704 | 5.556931 | 1.31839981669068e-14 |
| hsa-miR-4516 | 6.204732 | 2.45188141817117e-09 |
| hsa-miR-516a-5p | 6.568527 | 0.0002 |
| hsa-miR-520a-5p | 6.601334 | 0.0003 |
| hsa-miR-1283 | 6.754410 | 0.0006 |
| hsa-miR-205-5p | 7.456382 | 5.98938092423428e-06 |
| hsa-miR-516b-5p | 7.832622 | 1.63257846486656e-07 |
| hsa-miR-515-5p | 7.932070 | 1.1320132976681e-07 |
| hsa-miR-3648 | 7.959782 | 4.44984741989771e-06 |
| hsa-miR-1293 | 8.087553 | 3.8867988090603e-05 |
| hsa-miR-3150b-5p | 9.200499 | 1.53483621240369e-08 |
| hsa-miR-10396a-5p | 9.467601 | 2.7547834597426e-22 |
| hsa-miR-10396b-5p | 9.467601 | 2.7547834597426e-22 |
| hsa-miR-9901 | 10.005167 | 1.91996337144509e-11 |
