# Supplementary material for: Extracellular vesicle-associated miR-515-5p from adipose tissue regulates placental metabolism and fetal growth in gestational diabetes mellitus
Source: Cardiovasc Diabetol. 2025 May 14;24:205. doi: 10.1186/s12933-025-02739-z (PMC12080180; doi:10.1186/s12933-025-02739-z)
Supplement: Supplementary file 3 — Supplementary Material 3 [file 12933_2025_2739_MOESM3_ESM.docx]

**Supplementary Table 2: Table below shows the miRNA identified in adipose tissue from NGT and GDM with no significant difference in the miRNA profile**

| miRNA | log2FoldChange | pvalue |
| --- | --- | --- |
| hsa-miR-411-5p | -0.00244 | 0.0046 |
| hsa-miR-874-5p | -0.00153 | 0.0138 |
| hsa-miR-125b-5p | -0.00152 | 0.0696 |
| hsa-miR-484 | -0.00144 | 0.1358 |
| hsa-let-7c-5p | -0.00140 | 0.1208 |
| hsa-miR-181d-5p | -0.00127 | 0.1511 |
| hsa-miR-146b-5p | -0.00111 | 0.1326 |
| hsa-let-7a-5p | -0.00107 | 0.1499 |
| hsa-miR-181a-5p | -0.00107 | 0.1683 |
| hsa-miR-125a-5p | -0.00098 | 0.1881 |
| hsa-miR-181b-5p | -0.00098 | 0.2655 |
| hsa-miR-5683 | -0.00096 | 0.0094 |
| hsa-miR-369-5p | -0.00093 | 0.0737 |
| hsa-miR-199b-5p | -0.00092 | 0.2306 |
| hsa-miR-26a-5p | -0.00089 | 0.2313 |
| hsa-let-7f-5p | -0.00088 | 0.2618 |
| hsa-miR-1260b | -0.00084 | 0.1718 |
| hsa-miR-584-5p | -0.00084 | 0.2582 |
| hsa-miR-9-5p | -0.00081 | 0.1243 |
| hsa-let-7e-5p | -0.00080 | 0.2723 |
| hsa-miR-454-5p | -0.00075 | 0.0359 |
| hsa-miR-362-5p | -0.00074 | 0.0887 |
| hsa-miR-99a-5p | -0.00074 | 0.3859 |
| hsa-miR-744-5p | -0.00073 | 0.2373 |
| hsa-miR-299-3p | -0.00072 | 0.1935 |
| hsa-miR-873-5p | -0.00070 | 0.0475 |
| hsa-miR-1260a | -0.00069 | 0.2518 |
| hsa-miR-382-5p | -0.00067 | 0.1308 |
| hsa-miR-151a-5p | -0.00066 | 0.2684 |
| hsa-miR-652-5p | -0.00065 | 0.2448 |
| hsa-miR-34a-5p | -0.00065 | 0.3734 |
| hsa-miR-100-5p | -0.00064 | 0.5378 |
| hsa-miR-561-5p | -0.00063 | 0.2407 |
| hsa-miR-204-5p | -0.00063 | 0.1980 |
| hsa-miR-28-5p | -0.00062 | 0.3847 |
| hsa-miR-136-5p | -0.00061 | 0.4210 |
| hsa-miR-24-2-5p | -0.00061 | 0.3507 |
| hsa-miR-154-5p | -0.00060 | 0.1286 |
| hsa-miR-127-5p | -0.00059 | 0.3252 |
| hsa-miR-3613-5p | -0.00059 | 0.3415 |
| hsa-let-7b-5p | -0.00058 | 0.5162 |
| hsa-let-7g-5p | -0.00056 | 0.5624 |
| hsa-miR-181c-5p | -0.00052 | 0.6054 |
| hsa-miR-299-5p | -0.00052 | 0.1053 |
| hsa-miR-199a-5p | -0.00051 | 0.5372 |
| hsa-miR-7977 | -0.00050 | 0.3458 |
| hsa-miR-5585-3p | -0.00047 | 0.1886 |
| hsa-miR-296-5p | -0.00046 | 0.2056 |
| hsa-miR-214-5p | -0.00044 | 0.5280 |
| hsa-miR-1271-5p | -0.00044 | 0.3856 |
| hsa-miR-432-5p | -0.00043 | 0.3224 |
| hsa-miR-195-5p | -0.00042 | 0.6075 |
| hsa-miR-1468-5p | -0.00042 | 0.2921 |
| hsa-miR-26b-5p | -0.00042 | 0.6880 |
| hsa-miR-887-5p | -0.00042 | 0.1693 |
| hsa-miR-190a-5p | -0.00040 | 0.5343 |
| hsa-miR-3195 | -0.00039 | 0.4190 |
| hsa-miR-134-5p | -0.00039 | 0.4690 |
| hsa-miR-186-5p | -0.00039 | 0.6863 |
| hsa-miR-185-5p | -0.00038 | 0.5851 |
| hsa-miR-361-5p | -0.00037 | 0.6810 |
| hsa-miR-5701 | -0.00037 | 0.4694 |
| hsa-miR-15a-5p | -0.00036 | 0.6745 |
| hsa-miR-4775 | -0.00036 | 0.2632 |
| hsa-miR-150-5p | -0.00035 | 0.5761 |
| hsa-miR-10b-5p | -0.00035 | 0.6734 |
| hsa-miR-619-5p | -0.00034 | 0.3487 |
| hsa-miR-497-5p | -0.00032 | 0.7212 |
| hsa-miR-654-5p | -0.00032 | 0.3453 |
| hsa-miR-337-5p | -0.00031 | 0.2828 |
| hsa-miR-95-5p | -0.00031 | 0.3410 |
| hsa-let-7d-5p | -0.00031 | 0.6988 |
| hsa-miR-146a-5p | -0.00030 | 0.7150 |
| hsa-miR-4516 | -0.00029 | 0.3843 |
| hsa-miR-10400-5p | -0.00028 | 0.4309 |
| hsa-miR-132-5p | -0.00028 | 0.5504 |
| hsa-miR-5588-5p | -0.00028 | 0.0979 |
| hsa-miR-431-5p | -0.00027 | 0.4092 |
| hsa-miR-1275 | -0.00026 | 0.6872 |
| hsa-miR-30d-5p | -0.00026 | 0.8086 |
| hsa-miR-664a-5p | -0.00025 | 0.6030 |
| hsa-miR-3196 | -0.00025 | 0.2840 |
| hsa-miR-202-5p | -0.00025 | 0.1354 |
| hsa-miR-193a-5p | -0.00024 | 0.5374 |
| hsa-miR-29c-5p | -0.00022 | 0.7240 |
| hsa-miR-221-5p | -0.00022 | 0.6921 |
| hsa-miR-769-5p | -0.00022 | 0.8249 |
| hsa-miR-2277-5p | -0.00022 | 0.4985 |
| hsa-miR-18a-5p | -0.00021 | 0.5846 |
| hsa-miR-625-5p | -0.00021 | 0.5538 |
| hsa-miR-152-5p | -0.00021 | 0.7295 |
| hsa-miR-616-5p | -0.00021 | 0.3452 |
| hsa-miR-15b-5p | -0.00021 | 0.7585 |
| hsa-miR-1285-5p | -0.00021 | 0.5684 |
| hsa-miR-320d | -0.00020 | 0.5965 |
| hsa-miR-576-5p | -0.00020 | 0.6613 |
| hsa-miR-409-5p | -0.00020 | 0.5383 |
| hsa-miR-6724-5p | -0.00019 | 0.3444 |
| hsa-miR-9901 | -0.00019 | 0.1493 |
| hsa-miR-370-5p | -0.00019 | 0.3964 |
| hsa-miR-423-5p | -0.00018 | 0.7077 |
| hsa-miR-212-5p | -0.00018 | 0.5717 |
| hsa-miR-148a-5p | -0.00018 | 0.8215 |
| hsa-miR-3152-5p | -0.00018 | 0.2672 |
| hsa-miR-504-5p | -0.00017 | 0.7383 |
| hsa-miR-548k | -0.00017 | 0.7840 |
| hsa-miR-4508 | -0.00016 | 0.7379 |
| hsa-miR-4455 | -0.00016 | 0.4928 |
| hsa-miR-130b-5p | -0.00016 | 0.7335 |
| hsa-miR-3942-5p | -0.00016 | 0.1804 |
| hsa-miR-345-5p | -0.00016 | 0.8305 |
| hsa-miR-10401-3p | -0.00015 | 0.5236 |
| hsa-miR-4767 | -0.00015 | 0.5240 |
| hsa-miR-4485-3p | -0.00015 | 0.7289 |
| hsa-miR-148b-5p | -0.00014 | 0.8438 |
| hsa-miR-1306-5p | -0.00014 | 0.6487 |
| hsa-miR-4284 | -0.00014 | 0.7995 |
| hsa-miR-3147 | -0.00013 | 0.4135 |
| hsa-miR-19b-1-5p | -0.00013 | 0.3181 |
| hsa-miR-449a | -0.00013 | 0.3014 |
| hsa-miR-6854-5p | -0.00013 | 0.1760 |
| hsa-miR-1910-5p | -0.00013 | 0.2323 |
| hsa-miR-548i | -0.00013 | 0.4935 |
| hsa-miR-6866-5p | -0.00013 | 0.4387 |
| hsa-miR-379-5p | -0.00013 | 0.7833 |
| hsa-miR-5690 | -0.00013 | 0.5562 |
| hsa-miR-493-5p | -0.00012 | 0.7857 |
| hsa-miR-550a-3-5p | -0.00012 | 0.5322 |
| hsa-miR-671-5p | -0.00012 | 0.7214 |
| hsa-miR-627-5p | -0.00012 | 0.6919 |
| hsa-miR-500a-5p | -0.00012 | 0.6530 |
| hsa-miR-4463 | -0.00012 | 0.2882 |
| hsa-miR-3059-5p | -0.00011 | 0.5830 |
| hsa-miR-433-5p | -0.00011 | 0.3690 |
| hsa-miR-135b-5p | -0.00011 | 0.7406 |
| hsa-miR-6735-5p | -0.00011 | 0.3801 |
| hsa-miR-1255a | -0.00011 | 0.3079 |
| hsa-miR-548n | -0.00011 | 0.5276 |
| hsa-miR-383-5p | -0.00011 | 0.3575 |
| hsa-miR-548ar-5p | -0.00011 | 0.6373 |
| hsa-miR-589-5p | -0.00011 | 0.8656 |
| hsa-miR-875-5p | -0.00011 | 0.2197 |
| hsa-miR-3173-5p | -0.00010 | 0.3961 |
| hsa-miR-450b-5p | -0.00010 | 0.8913 |
| hsa-miR-548aq-3p | -0.00010 | 0.6525 |
| hsa-miR-590-5p | -0.00010 | 0.6055 |
| hsa-miR-196b-5p | -0.00010 | 0.8030 |
| hsa-miR-6502-5p | -0.00009 | 0.6023 |
| hsa-miR-548h-5p | -0.00009 | 0.6982 |
| hsa-miR-550a-5p | -0.00009 | 0.6210 |
| hsa-miR-7976 | -0.00009 | 0.3969 |
| hsa-miR-190b-5p | -0.00009 | 0.7788 |
| hsa-miR-500b-5p | -0.00009 | 0.6872 |
| hsa-miR-612 | -0.00009 | 0.4059 |
| hsa-miR-548f-5p | -0.00008 | 0.7143 |
| hsa-miR-485-5p | -0.00008 | 0.8113 |
| hsa-miR-128-1-5p | -0.00008 | 0.7567 |
| hsa-miR-1246 | -0.00008 | 0.5190 |
| hsa-miR-548aj-5p | -0.00008 | 0.7224 |
| hsa-miR-27b-5p | -0.00008 | 0.9113 |
| hsa-miR-450a-5p | -0.00008 | 0.8946 |
| hsa-miR-3620-5p | -0.00008 | 0.5570 |
| hsa-miR-6516-5p | -0.00008 | 0.5757 |
| hsa-miR-301a-5p | -0.00008 | 0.5830 |
| hsa-miR-1248 | -0.00008 | 0.6660 |
| hsa-miR-3064-5p | -0.00008 | 0.3293 |
| hsa-miR-548ag | -0.00008 | 0.7164 |
| hsa-miR-519a-5p | -0.00008 | 0.4271 |
| hsa-miR-4709-5p | -0.00007 | 0.5381 |
| hsa-miR-6894-5p | -0.00007 | 0.5201 |
| hsa-miR-376a-2-5p | -0.00007 | 0.5146 |
| hsa-miR-6754-5p | -0.00007 | 0.3504 |
| hsa-miR-10399-5p | -0.00007 | 0.8724 |
| hsa-miR-6513-5p | -0.00007 | 0.5989 |
| hsa-miR-10394-5p | -0.00007 | 0.3861 |
| hsa-miR-5684 | -0.00007 | 0.3861 |
| hsa-miR-676-5p | -0.00007 | 0.3861 |
| hsa-miR-5699-5p | -0.00007 | 0.7393 |
| hsa-miR-3194-5p | -0.00007 | 0.5959 |
| hsa-miR-4433b-5p | -0.00007 | 0.4581 |
| hsa-miR-6875-5p | -0.00007 | 0.4619 |
| hsa-miR-3199 | -0.00007 | 0.6969 |
| hsa-miR-4661-5p | -0.00007 | 0.6922 |
| hsa-miR-585-5p | -0.00007 | 0.7828 |
| hsa-miR-25-5p | -0.00006 | 0.8409 |
| hsa-miR-548x-5p | -0.00006 | 0.7908 |
| hsa-miR-4687-5p | -0.00006 | 0.4210 |
| hsa-miR-6512-5p | -0.00006 | 0.4210 |
| hsa-miR-3181 | -0.00006 | 0.4235 |
| hsa-miR-4742-5p | -0.00006 | 0.4235 |
| hsa-miR-6796-5p | -0.00006 | 0.4235 |
| hsa-miR-483-5p | -0.00006 | 0.8508 |
| hsa-miR-4662a-5p | -0.00006 | 0.8730 |
| hsa-miR-518a-5p | -0.00006 | 0.4421 |
| hsa-miR-664b-5p | -0.00006 | 0.6740 |
| hsa-miR-7702 | -0.00006 | 0.4474 |
| hsa-miR-502-5p | -0.00005 | 0.8379 |
| hsa-miR-3934-5p | -0.00005 | 0.7470 |
| hsa-miR-487a-5p | -0.00005 | 0.7084 |
| hsa-miR-1283 | -0.00005 | 0.5614 |
| hsa-miR-1180-5p | -0.00005 | 0.4673 |
| hsa-miR-541-5p | -0.00005 | 0.4673 |
| hsa-miR-6886-5p | -0.00005 | 0.4701 |
| hsa-miR-518e-5p | -0.00005 | 0.5676 |
| hsa-miR-519b-5p | -0.00005 | 0.5676 |
| hsa-miR-519c-5p | -0.00005 | 0.5676 |
| hsa-miR-522-5p | -0.00005 | 0.5676 |
| hsa-miR-523-5p | -0.00005 | 0.5676 |
| hsa-miR-548ar-3p | -0.00005 | 0.5700 |
| hsa-miR-99b-5p | -0.00005 | 0.9532 |
| hsa-miR-4667-5p | -0.00005 | 0.5795 |
| hsa-miR-451a | -0.00005 | 0.9252 |
| hsa-miR-3648 | -0.00005 | 0.5844 |
| hsa-miR-6730-5p | -0.00005 | 0.5844 |
| hsa-miR-770-5p | -0.00005 | 0.6804 |
| hsa-miR-539-5p | -0.00005 | 0.7920 |
| hsa-miR-4497 | -0.00005 | 0.8002 |
| hsa-miR-376a-5p | -0.00005 | 0.9359 |
| hsa-miR-1304-5p | -0.00005 | 0.5914 |
| hsa-miR-641 | -0.00005 | 0.8815 |
| hsa-miR-4690-3p | -0.00005 | 0.5964 |
| hsa-miR-4762-5p | -0.00005 | 0.5964 |
| hsa-miR-216a-5p | -0.00004 | 0.8252 |
| hsa-miR-3674 | -0.00004 | 0.6129 |
| hsa-miR-5088-5p | -0.00004 | 0.4891 |
| hsa-miR-4426 | -0.00004 | 0.6155 |
| hsa-miR-532-5p | -0.00004 | 0.9618 |
| hsa-miR-3161 | -0.00004 | 0.6159 |
| hsa-miR-6852-5p | -0.00004 | 0.7627 |
| hsa-miR-3159 | -0.00004 | 0.6185 |
| hsa-miR-6877-3p | -0.00004 | 0.6185 |
| hsa-miR-200a-5p | -0.00004 | 0.8346 |
| hsa-miR-365a-5p | -0.00004 | 0.8866 |
| hsa-miR-4510 | -0.00004 | 0.8909 |
| hsa-miR-92b-5p | -0.00004 | 0.8655 |
| hsa-miR-210-5p | -0.00004 | 0.8746 |
| hsa-miR-324-5p | -0.00004 | 0.9544 |
| hsa-miR-5708 | -0.00004 | 0.7120 |
| hsa-miR-4705 | -0.00004 | 0.7502 |
| hsa-miR-4443 | -0.00003 | 0.7592 |
| hsa-miR-3145-5p | -0.00003 | 0.6001 |
| hsa-miR-3619-5p | -0.00003 | 0.6001 |
| hsa-miR-6872-5p | -0.00003 | 0.6001 |
| hsa-miR-7152-3p | -0.00003 | 0.6001 |
| hsa-miR-767-5p | -0.00003 | 0.6001 |
| hsa-miR-891a-5p | -0.00003 | 0.9075 |
| hsa-miR-5010-5p | -0.00003 | 0.6727 |
| hsa-miR-1261 | -0.00003 | 0.9000 |
| hsa-miR-582-5p | -0.00003 | 0.9406 |
| hsa-miR-3157-5p | -0.00003 | 0.8331 |
| hsa-miR-3652 | -0.00003 | 0.6312 |
| hsa-miR-3665 | -0.00003 | 0.6312 |
| hsa-miR-4515 | -0.00003 | 0.6312 |
| hsa-miR-527 | -0.00003 | 0.6312 |
| hsa-miR-6509-5p | -0.00003 | 0.6312 |
| hsa-miR-7155-3p | -0.00003 | 0.6312 |
| hsa-miR-1469 | -0.00003 | 0.6350 |
| hsa-miR-449c-5p | -0.00003 | 0.6350 |
| hsa-miR-5189-5p | -0.00003 | 0.6350 |
| hsa-miR-6751-5p | -0.00003 | 0.6350 |
| hsa-miR-5002-5p | -0.00003 | 0.8427 |
| hsa-miR-98-5p | -0.00003 | 0.9774 |
| hsa-miR-1243 | -0.00003 | 0.7857 |
| hsa-miR-3163 | -0.00003 | 0.8483 |
| hsa-miR-329-5p | -0.00003 | 0.7552 |
| hsa-miR-4517 | -0.00003 | 0.7552 |
| hsa-miR-597-5p | -0.00003 | 0.7552 |
| hsa-miR-3128 | -0.00002 | 0.6508 |
| hsa-miR-3617-5p | -0.00002 | 0.6508 |
| hsa-miR-548y | -0.00002 | 0.6508 |
| hsa-miR-6727-5p | -0.00002 | 0.6508 |
| hsa-miR-6789-5p | -0.00002 | 0.6508 |
| hsa-miR-1256 | -0.00002 | 0.7620 |
| hsa-miR-4481 | -0.00002 | 0.6576 |
| hsa-miR-4714-5p | -0.00002 | 0.6576 |
| hsa-miR-5000-5p | -0.00002 | 0.6576 |
| hsa-miR-885-5p | -0.00002 | 0.8861 |
| hsa-miR-6892-5p | -0.00002 | 0.8562 |
| hsa-miR-2116-5p | -0.00002 | 0.8251 |
| hsa-miR-219b-5p | -0.00002 | 0.7871 |
| hsa-miR-6746-5p | -0.00002 | 0.7871 |
| hsa-miR-7845-5p | -0.00002 | 0.7871 |
| hsa-miR-1262 | -0.00002 | 0.8932 |
| hsa-miR-1973 | -0.00002 | 0.9578 |
| hsa-miR-339-5p | -0.00002 | 0.9737 |
| hsa-miR-675-5p | -0.00002 | 0.9339 |
| hsa-miR-6508-3p | -0.00002 | 0.8395 |
| hsa-miR-3127-5p | -0.00002 | 0.8955 |
| hsa-miR-1298-5p | -0.00002 | 0.7943 |
| hsa-miR-320a-5p | -0.00002 | 0.7943 |
| hsa-miR-4634 | -0.00002 | 0.8425 |
| hsa-miR-1245b-5p | -0.00002 | 0.7965 |
| hsa-miR-10393-3p | -0.00002 | 0.8706 |
| hsa-miR-4458 | -0.00002 | 0.7856 |
| hsa-miR-4679 | -0.00002 | 0.7897 |
| hsa-miR-6809-5p | -0.00002 | 0.7897 |
| hsa-miR-4746-5p | -0.00002 | 0.8924 |
| hsa-miR-548q | -0.00002 | 0.9260 |
| hsa-miR-548g-5p | -0.00002 | 0.9493 |
| hsa-miR-7151-3p | -0.00001 | 0.8399 |
| hsa-miR-133a-5p | -0.00001 | 0.8473 |
| hsa-miR-18b-5p | -0.00001 | 0.9596 |
| hsa-miR-888-5p | -0.00001 | 0.9349 |
| hsa-miR-4725-5p | -0.00001 | 0.8496 |
| hsa-miR-509-3-5p | -0.00001 | 0.8496 |
| hsa-miR-191-5p | -0.00001 | 0.9893 |
| hsa-miR-10395-5p | -0.00001 | 0.8404 |
| hsa-miR-11399 | -0.00001 | 0.8404 |
| hsa-miR-1225-5p | -0.00001 | 0.8404 |
| hsa-miR-1255b-5p | -0.00001 | 0.8404 |
| hsa-miR-203b-5p | -0.00001 | 0.8404 |
| hsa-miR-2278 | -0.00001 | 0.8404 |
| hsa-miR-3125 | -0.00001 | 0.8404 |
| hsa-miR-3180-5p | -0.00001 | 0.8404 |
| hsa-miR-381-5p | -0.00001 | 0.8404 |
| hsa-miR-3925-5p | -0.00001 | 0.8404 |
| hsa-miR-3929 | -0.00001 | 0.8404 |
| hsa-miR-4429 | -0.00001 | 0.8404 |
| hsa-miR-4642 | -0.00001 | 0.8404 |
| hsa-miR-4706 | -0.00001 | 0.8404 |
| hsa-miR-4707-5p | -0.00001 | 0.8404 |
| hsa-miR-4798-5p | -0.00001 | 0.8404 |
| hsa-miR-487b-5p | -0.00001 | 0.8404 |
| hsa-miR-509-5p | -0.00001 | 0.8404 |
| hsa-miR-512-5p | -0.00001 | 0.8404 |
| hsa-miR-519d-5p | -0.00001 | 0.8404 |
| hsa-miR-5691 | -0.00001 | 0.8404 |
| hsa-miR-588 | -0.00001 | 0.8404 |
| hsa-miR-623 | -0.00001 | 0.8404 |
| hsa-miR-655-5p | -0.00001 | 0.8404 |
| hsa-miR-6729-5p | -0.00001 | 0.8404 |
| hsa-miR-6732-3p | -0.00001 | 0.8404 |
| hsa-miR-6750-5p | -0.00001 | 0.8404 |
| hsa-miR-6795-5p | -0.00001 | 0.8404 |
| hsa-miR-6797-5p | -0.00001 | 0.8404 |
| hsa-miR-6869-3p | -0.00001 | 0.8404 |
| hsa-miR-7155-5p | -0.00001 | 0.8404 |
| hsa-miR-891b | -0.00001 | 0.8404 |
| hsa-miR-1224-5p | -0.00001 | 0.8404 |
| hsa-miR-1267 | -0.00001 | 0.8404 |
| hsa-miR-3131 | -0.00001 | 0.8404 |
| hsa-miR-3165 | -0.00001 | 0.8404 |
| hsa-miR-3169 | -0.00001 | 0.8404 |
| hsa-miR-3183 | -0.00001 | 0.8404 |
| hsa-miR-3677-5p | -0.00001 | 0.8404 |
| hsa-miR-374c-3p | -0.00001 | 0.8404 |
| hsa-miR-3944-5p | -0.00001 | 0.8404 |
| hsa-miR-4301 | -0.00001 | 0.8404 |
| hsa-miR-4323 | -0.00001 | 0.8404 |
| hsa-miR-4445-5p | -0.00001 | 0.8404 |
| hsa-miR-4538 | -0.00001 | 0.8404 |
| hsa-miR-4651 | -0.00001 | 0.8404 |
| hsa-miR-4670-5p | -0.00001 | 0.8404 |
| hsa-miR-4676-5p | -0.00001 | 0.8404 |
| hsa-miR-4729 | -0.00001 | 0.8404 |
| hsa-miR-4802-5p | -0.00001 | 0.8404 |
| hsa-miR-488-5p | -0.00001 | 0.8404 |
| hsa-miR-5008-5p | -0.00001 | 0.8404 |
| hsa-miR-517-5p | -0.00001 | 0.8404 |
| hsa-miR-548a-5p | -0.00001 | 0.8404 |
| hsa-miR-548as-5p | -0.00001 | 0.8404 |
| hsa-miR-549a-5p | -0.00001 | 0.8404 |
| hsa-miR-6132 | -0.00001 | 0.8404 |
| hsa-miR-6507-5p | -0.00001 | 0.8404 |
| hsa-miR-6803-5p | -0.00001 | 0.8404 |
| hsa-miR-6850-3p | -0.00001 | 0.8404 |
| hsa-miR-7109-3p | -0.00001 | 0.8404 |
| hsa-miR-7111-5p | -0.00001 | 0.8404 |
| hsa-miR-7151-5p | -0.00001 | 0.8404 |
| hsa-miR-10397-5p | -0.00001 | 0.8404 |
| hsa-miR-11401 | -0.00001 | 0.8404 |
| hsa-miR-124-5p | -0.00001 | 0.8404 |
| hsa-miR-1249-5p | -0.00001 | 0.8404 |
| hsa-miR-1265 | -0.00001 | 0.8404 |
| hsa-miR-2115-5p | -0.00001 | 0.8404 |
| hsa-miR-3144-5p | -0.00001 | 0.8404 |
| hsa-miR-3171 | -0.00001 | 0.8404 |
| hsa-miR-3182 | -0.00001 | 0.8404 |
| hsa-miR-372-5p | -0.00001 | 0.8404 |
| hsa-miR-4327 | -0.00001 | 0.8404 |
| hsa-miR-4482-5p | -0.00001 | 0.8404 |
| hsa-miR-4498 | -0.00001 | 0.8404 |
| hsa-miR-4639-5p | -0.00001 | 0.8404 |
| hsa-miR-4648 | -0.00001 | 0.8404 |
| hsa-miR-4657 | -0.00001 | 0.8404 |
| hsa-miR-4663 | -0.00001 | 0.8404 |
| hsa-miR-4690-5p | -0.00001 | 0.8404 |
| hsa-miR-4720-5p | -0.00001 | 0.8404 |
| hsa-miR-4753-5p | -0.00001 | 0.8404 |
| hsa-miR-4782-5p | -0.00001 | 0.8404 |
| hsa-miR-5090 | -0.00001 | 0.8404 |
| hsa-miR-552-5p | -0.00001 | 0.8404 |
| hsa-miR-5579-5p | -0.00001 | 0.8404 |
| hsa-miR-5586-3p | -0.00001 | 0.8404 |
| hsa-miR-5587-5p | -0.00001 | 0.8404 |
| hsa-miR-573 | -0.00001 | 0.8404 |
| hsa-miR-601 | -0.00001 | 0.8404 |
| hsa-miR-642b-5p | -0.00001 | 0.8404 |
| hsa-miR-650 | -0.00001 | 0.8404 |
| hsa-miR-6508-5p | -0.00001 | 0.8404 |
| hsa-miR-6721-5p | -0.00001 | 0.8404 |
| hsa-miR-6755-5p | -0.00001 | 0.8404 |
| hsa-miR-6772-5p | -0.00001 | 0.8404 |
| hsa-miR-6807-5p | -0.00001 | 0.8404 |
| hsa-miR-6827-5p | -0.00001 | 0.8404 |
| hsa-miR-6874-5p | -0.00001 | 0.8404 |
| hsa-miR-7150 | -0.00001 | 0.8404 |
| hsa-miR-7152-5p | -0.00001 | 0.8404 |
| hsa-miR-7853-5p | -0.00001 | 0.8404 |
| hsa-miR-802 | -0.00001 | 0.8404 |
| hsa-miR-205-5p | -0.00001 | 0.9398 |
| hsa-miR-3133 | -0.00001 | 0.9110 |
| hsa-miR-17-5p | -0.00001 | 0.9910 |
| hsa-miR-551b-5p | 0.00000 | 0.9785 |
| hsa-miR-5689 | 0.00000 | 0.9954 |
| hsa-miR-2681-5p | 1.9658263558811e-07 | 0.9982 |
| hsa-miR-3130-5p | 1.75088424004857e-06 | 0.9884 |
| hsa-miR-4524a-5p | 2.12125417454905e-06 | 0.9916 |
| hsa-miR-4536-5p | 2.27663369061356e-06 | 0.9696 |
| hsa-miR-4668-5p | 2.27663369061356e-06 | 0.9696 |
| hsa-miR-494-5p | 2.27663369061356e-06 | 0.9696 |
| hsa-miR-6774-5p | 2.27663369061356e-06 | 0.9696 |
| hsa-miR-101-2-5p | 2.37475425629947e-06 | 0.9690 |
| hsa-miR-1284 | 2.37475425629947e-06 | 0.9690 |
| hsa-miR-1914-5p | 2.37475425629947e-06 | 0.9690 |
| hsa-miR-376b-5p | 2.37475425629947e-06 | 0.9690 |
| hsa-miR-376c-5p | 2.37475425629947e-06 | 0.9690 |
| hsa-miR-3976 | 2.37475425629947e-06 | 0.9690 |
| hsa-miR-4731-5p | 2.37475425629947e-06 | 0.9690 |
| hsa-miR-580-5p | 2.37475425629947e-06 | 0.9690 |
| hsa-miR-6780a-5p | 2.37475425629947e-06 | 0.9690 |
| hsa-miR-3681-5p | 2.42946573651436e-06 | 0.9686 |
| hsa-miR-4745-5p | 2.42946573651436e-06 | 0.9686 |
| hsa-miR-548ap-5p | 2.42946573651436e-06 | 0.9686 |
| hsa-miR-3126-5p | 2.44746250990873e-06 | 0.9685 |
| hsa-miR-374c-5p | 2.44746250990873e-06 | 0.9685 |
| hsa-miR-4757-5p | 2.44746250990873e-06 | 0.9685 |
| hsa-miR-548ax | 2.44746250990873e-06 | 0.9685 |
| hsa-miR-6829-5p | 2.4603369230391e-06 | 0.9792 |
| hsa-miR-3135a | 2.52329171755219e-06 | 0.9680 |
| hsa-miR-6510-5p | 2.52329171755219e-06 | 0.9680 |
| hsa-miR-3150a-5p | 2.54079784530582e-06 | 0.9679 |
| hsa-miR-4505 | 2.54079784530582e-06 | 0.9679 |
| hsa-miR-4796-5p | 2.54079784530582e-06 | 0.9679 |
| hsa-miR-3120-5p | 2.69476976453519e-06 | 0.9669 |
| hsa-miR-508-5p | 2.69476976453519e-06 | 0.9669 |
| hsa-miR-510-5p | 2.69476976453519e-06 | 0.9669 |
| hsa-miR-513c-5p | 2.69476976453519e-06 | 0.9669 |
| hsa-miR-1302 | 2.82994858911302e-06 | 0.9661 |
| hsa-miR-1909-5p | 2.82994858911302e-06 | 0.9661 |
| hsa-miR-4289 | 2.82994858911302e-06 | 0.9661 |
| hsa-miR-5581-3p | 2.82994858911302e-06 | 0.9661 |
| hsa-miR-323a-5p | 2.84591833678954e-06 | 0.9660 |
| hsa-miR-579-5p | 2.84591833678954e-06 | 0.9660 |
| hsa-miR-758-5p | 2.84591833678954e-06 | 0.9660 |
| hsa-miR-3661 | 2.98401092593554e-06 | 0.9799 |
| hsa-miR-4768-5p | 2.98401092593554e-06 | 0.9799 |
| hsa-miR-380-5p | 4.02860023926377e-06 | 0.9639 |
| hsa-miR-4794 | 5.99799034253338e-06 | 0.9465 |
| hsa-miR-6858-5p | 6.67912390291439e-06 | 0.9403 |
| hsa-miR-505-5p | 6.81140194218535e-06 | 0.9686 |
| hsa-miR-6770-3p | 9.66981242796884e-06 | 0.9303 |
| hsa-miR-548e-5p | 1.05613076605685e-05 | 0.9604 |
| hsa-miR-216b-5p | 1.05854168151323e-05 | 0.9129 |
| hsa-miR-200b-5p | 1.12798852081907e-05 | 0.9389 |
| hsa-miR-10396b-3p | 1.13531777627977e-05 | 0.9524 |
| hsa-miR-1292-5p | 1.14807231582717e-05 | 0.9237 |
| hsa-miR-4531 | 1.16528621456987e-05 | 0.9646 |
| hsa-miR-548ad-5p | 1.2214092651588e-05 | 0.9610 |
| hsa-miR-548w | 1.28461989963949e-05 | 0.9532 |
| hsa-miR-4636 | 1.42166976503006e-05 | 0.9267 |
| hsa-miR-122b-5p | 1.45231702338755e-05 | 0.7872 |
| hsa-miR-1269b | 1.45231702338755e-05 | 0.7872 |
| hsa-miR-3124-5p | 1.45231702338755e-05 | 0.7872 |
| hsa-miR-3135b | 1.45231702338755e-05 | 0.7872 |
| hsa-miR-3680-5p | 1.45231702338755e-05 | 0.7872 |
| hsa-miR-3685 | 1.45231702338755e-05 | 0.7872 |
| hsa-miR-4288 | 1.45231702338755e-05 | 0.7872 |
| hsa-miR-4423-5p | 1.45231702338755e-05 | 0.7872 |
| hsa-miR-4446-5p | 1.45231702338755e-05 | 0.7872 |
| hsa-miR-4529-5p | 1.45231702338755e-05 | 0.7872 |
| hsa-miR-4649-5p | 1.45231702338755e-05 | 0.7872 |
| hsa-miR-4680-5p | 1.45231702338755e-05 | 0.7872 |
| hsa-miR-4750-5p | 1.45231702338755e-05 | 0.7872 |
| hsa-miR-4776-5p | 1.45231702338755e-05 | 0.7872 |
| hsa-miR-518c-5p | 1.45231702338755e-05 | 0.7872 |
| hsa-miR-520a-5p | 1.45231702338755e-05 | 0.7872 |
| hsa-miR-520d-5p | 1.45231702338755e-05 | 0.7872 |
| hsa-miR-524-5p | 1.45231702338755e-05 | 0.7872 |
| hsa-miR-567 | 1.45231702338755e-05 | 0.7872 |
| hsa-miR-610 | 1.45231702338755e-05 | 0.7872 |
| hsa-miR-6506-5p | 1.45231702338755e-05 | 0.7872 |
| hsa-miR-6811-3p | 1.45231702338755e-05 | 0.7872 |
| hsa-miR-6815-5p | 1.45231702338755e-05 | 0.7872 |
| hsa-miR-6852-3p | 1.45231702338755e-05 | 0.7872 |
| hsa-miR-6877-5p | 1.45231702338755e-05 | 0.7872 |
| hsa-miR-1250-5p | 1.45231702338755e-05 | 0.7872 |
| hsa-miR-1915-5p | 1.45231702338755e-05 | 0.7872 |
| hsa-miR-3616-5p | 1.45231702338755e-05 | 0.7872 |
| hsa-miR-4280 | 1.45231702338755e-05 | 0.7872 |
| hsa-miR-4474-5p | 1.45231702338755e-05 | 0.7872 |
| hsa-miR-4511 | 1.45231702338755e-05 | 0.7872 |
| hsa-miR-4722-5p | 1.45231702338755e-05 | 0.7872 |
| hsa-miR-4755-5p | 1.45231702338755e-05 | 0.7872 |
| hsa-miR-4758-5p | 1.45231702338755e-05 | 0.7872 |
| hsa-miR-4804-5p | 1.45231702338755e-05 | 0.7872 |
| hsa-miR-6069 | 1.45231702338755e-05 | 0.7872 |
| hsa-miR-656-5p | 1.45231702338755e-05 | 0.7872 |
| hsa-miR-6720-5p | 1.45231702338755e-05 | 0.7872 |
| hsa-miR-6741-5p | 1.45231702338755e-05 | 0.7872 |
| hsa-miR-6822-3p | 1.45231702338755e-05 | 0.7872 |
| hsa-miR-6826-5p | 1.45231702338755e-05 | 0.7872 |
| hsa-miR-6834-5p | 1.45231702338755e-05 | 0.7872 |
| hsa-miR-6888-5p | 1.45231702338755e-05 | 0.7872 |
| hsa-miR-7107-5p | 1.45231702338755e-05 | 0.7872 |
| hsa-miR-7162-5p | 1.45231702338755e-05 | 0.7872 |
| hsa-miR-10400-3p | 1.45231702338755e-05 | 0.7872 |
| hsa-miR-12116 | 1.45231702338755e-05 | 0.7872 |
| hsa-miR-12121 | 1.45231702338755e-05 | 0.7872 |
| hsa-miR-3193 | 1.45231702338755e-05 | 0.7872 |
| hsa-miR-3691-5p | 1.45231702338755e-05 | 0.7872 |
| hsa-miR-3918 | 1.45231702338755e-05 | 0.7872 |
| hsa-miR-4270 | 1.45231702338755e-05 | 0.7872 |
| hsa-miR-4297 | 1.45231702338755e-05 | 0.7872 |
| hsa-miR-4298 | 1.45231702338755e-05 | 0.7872 |
| hsa-miR-4328 | 1.45231702338755e-05 | 0.7872 |
| hsa-miR-4681 | 1.45231702338755e-05 | 0.7872 |
| hsa-miR-4686 | 1.45231702338755e-05 | 0.7872 |
| hsa-miR-4701-5p | 1.45231702338755e-05 | 0.7872 |
| hsa-miR-4745-3p | 1.45231702338755e-05 | 0.7872 |
| hsa-miR-513b-5p | 1.45231702338755e-05 | 0.7872 |
| hsa-miR-514a-5p | 1.45231702338755e-05 | 0.7872 |
| hsa-miR-5580-5p | 1.45231702338755e-05 | 0.7872 |
| hsa-miR-592 | 1.45231702338755e-05 | 0.7872 |
| hsa-miR-6500-5p | 1.45231702338755e-05 | 0.7872 |
| hsa-miR-6515-5p | 1.45231702338755e-05 | 0.7872 |
| hsa-miR-6718-5p | 1.45231702338755e-05 | 0.7872 |
| hsa-miR-6733-5p | 1.45231702338755e-05 | 0.7872 |
| hsa-miR-6740-5p | 1.45231702338755e-05 | 0.7872 |
| hsa-miR-6827-3p | 1.45231702338755e-05 | 0.7872 |
| hsa-miR-6836-5p | 1.45231702338755e-05 | 0.7872 |
| hsa-miR-6840-5p | 1.45231702338755e-05 | 0.7872 |
| hsa-miR-6842-5p | 1.45231702338755e-05 | 0.7872 |
| hsa-miR-6862-5p | 1.45231702338755e-05 | 0.7872 |
| hsa-miR-6869-5p | 1.45231702338755e-05 | 0.7872 |
| hsa-miR-6886-3p | 1.45231702338755e-05 | 0.7872 |
| hsa-miR-7113-3p | 1.45231702338755e-05 | 0.7872 |
| hsa-miR-629-5p | 1.62539466083095e-05 | 0.9654 |
| hsa-let-7i-5p | 1.6665550594352e-05 | 0.9823 |
| hsa-miR-6514-5p | 1.7882081261968e-05 | 0.8948 |
| hsa-miR-193b-5p | 1.8261258403826e-05 | 0.9692 |
| hsa-miR-6505-5p | 1.83893737723008e-05 | 0.7885 |
| hsa-miR-3074-5p | 1.83895601224347e-05 | 0.9292 |
| hsa-miR-577 | 1.89275243178648e-05 | 0.8808 |
| hsa-miR-4317 | 1.92912963864666e-05 | 0.7833 |
| hsa-miR-92a-1-5p | 2.02378780258741e-05 | 0.9342 |
| hsa-miR-548j-5p | 2.05825036690544e-05 | 0.8759 |
| hsa-miR-4786-5p | 2.0978052624087e-05 | 0.8182 |
| hsa-miR-10396b-5p | 2.19044072016027e-05 | 0.8631 |
| hsa-miR-4770 | 2.25079055187633e-05 | 0.7529 |
| hsa-miR-556-5p | 2.39603118650443e-05 | 0.8570 |
| hsa-miR-3175 | 2.41483509728156e-05 | 0.6557 |
| hsa-miR-4436b-5p | 2.41483509728156e-05 | 0.6557 |
| hsa-miR-939-5p | 2.41483509728156e-05 | 0.6557 |
| hsa-miR-3926 | 2.57651733551139e-05 | 0.6349 |
| hsa-miR-4478 | 2.57651733551139e-05 | 0.6349 |
| hsa-miR-5001-5p | 2.57651733551139e-05 | 0.6349 |
| hsa-miR-5091 | 2.57651733551139e-05 | 0.6349 |
| hsa-miR-103a-1-5p | 2.58426517428706e-05 | 0.7419 |
| hsa-miR-3916 | 2.58426517428706e-05 | 0.7419 |
| hsa-miR-4740-5p | 2.58426517428706e-05 | 0.7419 |
| hsa-miR-378b | 2.65414559272474e-05 | 0.9115 |
| hsa-miR-6511a-5p | 2.67754029619154e-05 | 0.7373 |
| hsa-miR-942-5p | 2.79869603393156e-05 | 0.9097 |
| hsa-miR-548aw | 2.97120978358499e-05 | 0.7574 |
| hsa-miR-548at-5p | 3.1722476974934e-05 | 0.7604 |
| hsa-miR-6894-3p | 3.1722476974934e-05 | 0.7604 |
| hsa-miR-141-5p | 3.25404154156593e-05 | 0.6883 |
| hsa-miR-4424 | 3.25404154156593e-05 | 0.6883 |
| hsa-miR-4435 | 3.25404154156593e-05 | 0.6883 |
| hsa-miR-3679-5p | 3.30492275824748e-05 | 0.5957 |
| hsa-miR-4647 | 3.30492275824748e-05 | 0.5957 |
| hsa-miR-4677-5p | 3.30492275824748e-05 | 0.5957 |
| hsa-miR-4749-5p | 3.30492275824748e-05 | 0.5957 |
| hsa-miR-5700 | 3.30492275824748e-05 | 0.5957 |
| hsa-miR-6818-5p | 3.30492275824748e-05 | 0.5957 |
| hsa-miR-548l | 3.34680466929949e-05 | 0.6843 |
| hsa-miR-377-5p | 3.3730679158039e-05 | 0.9429 |
| hsa-miR-10527-5p | 3.38438936201989e-05 | 0.8064 |
| hsa-miR-3180 | 3.39248634266999e-05 | 0.6508 |
| hsa-miR-3180-3p | 3.39248634266999e-05 | 0.6508 |
| hsa-miR-4732-5p | 3.39248634266999e-05 | 0.6508 |
| hsa-miR-548ak | 3.39248634266999e-05 | 0.6508 |
| hsa-miR-5706 | 3.39248634266999e-05 | 0.6508 |
| hsa-miR-23b-5p | 3.3977194971772e-05 | 0.8889 |
| hsa-miR-153-5p | 3.40508885606765e-05 | 0.8169 |
| hsa-miR-5009-5p | 3.40911854026286e-05 | 0.7438 |
| hsa-miR-6499-5p | 3.43357880133296e-05 | 0.7231 |
| hsa-miR-10392-5p | 3.47411726068661e-05 | 0.6710 |
| hsa-miR-6859-5p | 3.5536658193607e-05 | 0.7661 |
| hsa-miR-618 | 3.55381816812936e-05 | 0.8664 |
| hsa-miR-449b-5p | 3.56770046841621e-05 | 0.6671 |
| hsa-miR-3200-5p | 3.57875130967453e-05 | 0.6666 |
| hsa-miR-519a-2-5p | 3.57875130967453e-05 | 0.6666 |
| hsa-miR-520b-5p | 3.57875130967453e-05 | 0.6666 |
| hsa-miR-378a-5p | 3.5916661203181e-05 | 0.9556 |
| hsa-miR-10396a-5p | 3.61361460073264e-05 | 0.7434 |
| hsa-miR-921 | 3.62222005591069e-05 | 0.6303 |
| hsa-miR-3690 | 3.66598189235884e-05 | 0.7757 |
| hsa-miR-499a-5p | 3.81521411339402e-05 | 0.8860 |
| hsa-miR-6839-5p | 3.86309244150404e-05 | 0.5229 |
| hsa-miR-5187-5p | 4.10433392088742e-05 | 0.5348 |
| hsa-miR-1276 | 4.11683695740199e-05 | 0.7713 |
| hsa-miR-3174 | 4.16390907344752e-05 | 0.7648 |
| hsa-miR-4999-5p | 4.16440978079474e-05 | 0.7762 |
| hsa-miR-34b-5p | 4.22784485285416e-05 | 0.9023 |
| hsa-miR-11400 | 4.24327614510394e-05 | 0.7019 |
| hsa-miR-5696 | 4.31717353271438e-05 | 0.7326 |
| hsa-miR-1233-5p | 4.34749281457953e-05 | 0.5170 |
| hsa-miR-5584-5p | 4.34749281457953e-05 | 0.5170 |
| hsa-miR-6838-5p | 4.34749281457953e-05 | 0.5170 |
| hsa-miR-8059 | 4.34749281457953e-05 | 0.5170 |
| hsa-miR-211-5p | 4.44888457002262e-05 | 0.8743 |
| hsa-miR-10396a-3p | 4.51487513181095e-05 | 0.6677 |
| hsa-miR-605-5p | 4.56103212549645e-05 | 0.7642 |
| hsa-miR-4791 | 4.5638762319686e-05 | 0.7206 |
| hsa-miR-491-5p | 4.60721918067355e-05 | 0.8046 |
| hsa-miR-2110 | 4.782987987888e-05 | 0.8950 |
| hsa-miR-6501-5p | 4.91423487965884e-05 | 0.7296 |
| hsa-miR-548ai | 4.97298955780985e-05 | 0.5647 |
| hsa-miR-570-5p | 4.97298955780985e-05 | 0.5647 |
| hsa-miR-6734-5p | 4.97298955780985e-05 | 0.5647 |
| hsa-miR-548t-5p | 5.18524771137203e-05 | 0.5509 |
| hsa-miR-516b-5p | 5.53598245656887e-05 | 0.8325 |
| hsa-miR-1252-5p | 5.65955488846169e-05 | 0.4307 |
| hsa-miR-1537-5p | 5.65955488846169e-05 | 0.4307 |
| hsa-miR-6813-5p | 5.65955488846169e-05 | 0.4307 |
| hsa-miR-3129-5p | 5.66528656228178e-05 | 0.5213 |
| hsa-miR-4800-5p | 5.90647224494991e-05 | 0.4163 |
| hsa-miR-23a-5p | 6.21261853943784e-05 | 0.7643 |
| hsa-miR-378j | 6.2837937097378e-05 | 0.3614 |
| hsa-miR-19a-5p | 6.33769131284948e-05 | 0.6295 |
| hsa-miR-1323 | 6.37684099508095e-05 | 0.6814 |
| hsa-miR-651-5p | 6.43604186613072e-05 | 0.8753 |
| hsa-miR-1226-5p | 6.48977335912031e-05 | 0.3846 |
| hsa-miR-371b-5p | 6.48977335912031e-05 | 0.3846 |
| hsa-miR-548ae-5p | 6.71733183467169e-05 | 0.8085 |
| hsa-miR-4466 | 6.95730799267855e-05 | 0.3602 |
| hsa-miR-6126 | 6.97182290456269e-05 | 0.5703 |
| hsa-miR-6847-5p | 7.13207853177947e-05 | 0.4668 |
| hsa-miR-889-5p | 7.24748344944588e-05 | 0.5380 |
| hsa-miR-4638-3p | 7.33076597333283e-05 | 0.4302 |
| hsa-miR-9902 | 7.49816877096447e-05 | 0.5028 |
| hsa-miR-6820-5p | 7.50931852666709e-05 | 0.3337 |
| hsa-miR-3202 | 7.59950259844038e-05 | 0.4177 |
| hsa-miR-6882-5p | 7.66167301913281e-05 | 0.3730 |
| hsa-miR-3677-3p | 7.81917589627396e-05 | 0.5943 |
| hsa-miR-1266-5p | 8.14386389528353e-05 | 0.5643 |
| hsa-miR-371a-5p | 8.25722997903134e-05 | 0.5887 |
| hsa-miR-624-5p | 8.30875714289351e-05 | 0.6875 |
| hsa-miR-548d-5p | 8.35417674722511e-05 | 0.8152 |
| hsa-miR-659-5p | 8.42883189681132e-05 | 0.7488 |
| hsa-miR-330-5p | 8.46153934236692e-05 | 0.8752 |
| hsa-miR-4665-5p | 8.56083975309436e-05 | 0.3142 |
| hsa-miR-217-5p | 9.18391299566852e-05 | 0.4965 |
| hsa-miR-4772-5p | 9.24524124646298e-05 | 0.5906 |
| hsa-miR-3136-5p | 9.246599133761e-05 | 0.3412 |
| hsa-miR-3612 | 9.2633630187849e-05 | 0.4329 |
| hsa-miR-6503-5p | 9.29272596863409e-05 | 0.7042 |
| hsa-miR-6868-3p | 9.66735848124331e-05 | 0.3191 |
| hsa-miR-548an | 9.6765320645845e-05 | 0.3203 |
| hsa-miR-1228-5p | 9.9016862580589e-05 | 0.3424 |
| hsa-miR-365b-5p | 9.93016015028614e-05 | 0.8049 |
| hsa-miR-197-5p | 0.00010 | 0.2320 |
| hsa-miR-663a | 0.00010 | 0.5225 |
| hsa-miR-548ab | 0.00010 | 0.5619 |
| hsa-miR-4326 | 0.00010 | 0.6064 |
| hsa-miR-526b-5p | 0.00010 | 0.2243 |
| hsa-miR-1296-5p | 0.00011 | 0.8342 |
| hsa-miR-1273h-5p | 0.00011 | 0.5332 |
| hsa-miR-4485-5p | 0.00012 | 0.5325 |
| hsa-miR-219a-5p | 0.00012 | 0.5520 |
| hsa-miR-412-5p | 0.00012 | 0.5228 |
| hsa-miR-223-5p | 0.00012 | 0.7775 |
| hsa-miR-3142 | 0.00012 | 0.2578 |
| hsa-miR-155-5p | 0.00013 | 0.8431 |
| hsa-miR-24-1-5p | 0.00013 | 0.7356 |
| hsa-miR-103a-2-5p | 0.00013 | 0.5434 |
| hsa-miR-331-5p | 0.00013 | 0.7090 |
| hsa-miR-346 | 0.00014 | 0.4576 |
| hsa-miR-515-5p | 0.00014 | 0.4794 |
| hsa-miR-34c-5p | 0.00014 | 0.8180 |
| hsa-miR-422a | 0.00014 | 0.7076 |
| hsa-miR-142-5p | 0.00014 | 0.8050 |
| hsa-miR-378h | 0.00015 | 0.3780 |
| hsa-miR-548b-5p | 0.00015 | 0.3490 |
| hsa-miR-3928-3p | 0.00015 | 0.4095 |
| hsa-miR-542-5p | 0.00015 | 0.5735 |
| hsa-miR-106a-5p | 0.00015 | 0.6554 |
| hsa-miR-525-5p | 0.00016 | 0.1123 |
| hsa-miR-3913-5p | 0.00016 | 0.5948 |
| hsa-miR-29b-1-5p | 0.00016 | 0.3705 |
| hsa-miR-129-5p | 0.00016 | 0.1504 |
| hsa-miR-548ay-5p | 0.00017 | 0.5551 |
| hsa-miR-3614-5p | 0.00017 | 0.4487 |
| hsa-miR-3622a-5p | 0.00017 | 0.3806 |
| hsa-miR-30e-5p | 0.00017 | 0.8668 |
| hsa-miR-516a-5p | 0.00017 | 0.4507 |
| hsa-miR-378g | 0.00017 | 0.6766 |
| hsa-miR-30a-5p | 0.00017 | 0.8615 |
| hsa-miR-574-5p | 0.00018 | 0.8108 |
| hsa-miR-4430 | 0.00018 | 0.1630 |
| hsa-miR-1185-5p | 0.00018 | 0.5528 |
| hsa-miR-144-5p | 0.00018 | 0.7370 |
| hsa-miR-224-5p | 0.00018 | 0.7753 |
| hsa-miR-545-5p | 0.00018 | 0.4046 |
| hsa-miR-1908-5p | 0.00019 | 0.3326 |
| hsa-miR-342-5p | 0.00020 | 0.7275 |
| hsa-miR-31-5p | 0.00020 | 0.6413 |
| hsa-miR-4488 | 0.00020 | 0.5490 |
| hsa-miR-503-5p | 0.00020 | 0.4018 |
| hsa-miR-6511b-5p | 0.00021 | 0.2307 |
| hsa-miR-2467-5p | 0.00021 | 0.5316 |
| hsa-miR-548az-5p | 0.00021 | 0.1294 |
| hsa-miR-222-5p | 0.00022 | 0.5288 |
| hsa-miR-20b-5p | 0.00023 | 0.5553 |
| hsa-miR-548ba | 0.00023 | 0.6127 |
| hsa-miR-30b-5p | 0.00023 | 0.7968 |
| hsa-miR-138-5p | 0.00023 | 0.2878 |
| hsa-miR-660-5p | 0.00023 | 0.7623 |
| hsa-miR-7705 | 0.00024 | 0.5219 |
| hsa-miR-3065-5p | 0.00024 | 0.1516 |
| hsa-miR-7704 | 0.00024 | 0.6923 |
| hsa-miR-3605-5p | 0.00025 | 0.3027 |
| hsa-miR-2355-5p | 0.00025 | 0.5777 |
| hsa-miR-1843 | 0.00026 | 0.6169 |
| hsa-miR-4521 | 0.00026 | 0.4584 |
| hsa-miR-653-5p | 0.00027 | 0.6548 |
| hsa-miR-188-5p | 0.00027 | 0.5319 |
| hsa-miR-16-5p | 0.00028 | 0.7267 |
| hsa-miR-6504-5p | 0.00028 | 0.4022 |
| hsa-miR-3178 | 0.00028 | 0.1065 |
| hsa-miR-135a-5p | 0.00030 | 0.1386 |
| hsa-miR-1270 | 0.00030 | 0.1488 |
| hsa-miR-1291 | 0.00030 | 0.3568 |
| hsa-miR-877-5p | 0.00030 | 0.5984 |
| hsa-miR-511-5p | 0.00030 | 0.5237 |
| hsa-miR-1268b | 0.00031 | 0.2178 |
| hsa-miR-10a-5p | 0.00032 | 0.7577 |
| hsa-miR-425-5p | 0.00032 | 0.7041 |
| hsa-miR-6761-5p | 0.00032 | 0.1303 |
| hsa-miR-30c-5p | 0.00032 | 0.7195 |
| hsa-miR-96-5p | 0.00033 | 0.3076 |
| hsa-miR-1287-5p | 0.00033 | 0.4520 |
| hsa-miR-501-5p | 0.00034 | 0.2036 |
| hsa-miR-642a-5p | 0.00034 | 0.1796 |
| hsa-miR-4787-5p | 0.00035 | 0.2458 |
| hsa-miR-33a-5p | 0.00035 | 0.5575 |
| hsa-miR-1277-5p | 0.00036 | 0.4804 |
| hsa-miR-548au-5p | 0.00036 | 0.1262 |
| hsa-miR-1268a | 0.00036 | 0.1644 |
| hsa-miR-194-5p | 0.00038 | 0.5268 |
| hsa-miR-145-5p | 0.00039 | 0.4916 |
| hsa-miR-548c-5p | 0.00039 | 0.1838 |
| hsa-miR-548o-5p | 0.00039 | 0.1838 |
| hsa-miR-21-5p | 0.00040 | 0.5467 |
| hsa-miR-218-5p | 0.00040 | 0.6046 |
| hsa-miR-10399-3p | 0.00041 | 0.3774 |
| hsa-miR-1179 | 0.00041 | 0.2540 |
| hsa-miR-455-5p | 0.00042 | 0.6125 |
| hsa-miR-374b-5p | 0.00042 | 0.3991 |
| hsa-miR-378i | 0.00042 | 0.6555 |
| hsa-miR-548am-5p | 0.00043 | 0.1190 |
| hsa-miR-143-5p | 0.00044 | 0.4550 |
| hsa-miR-2114-5p | 0.00045 | 0.1017 |
| hsa-miR-338-5p | 0.00045 | 0.1502 |
| hsa-miR-4454 | 0.00046 | 0.4576 |
| hsa-miR-33b-5p | 0.00046 | 0.4664 |
| hsa-miR-27a-5p | 0.00047 | 0.2600 |
| hsa-miR-378d | 0.00048 | 0.5450 |
| hsa-miR-22-5p | 0.00049 | 0.2944 |
| hsa-miR-486-5p | 0.00049 | 0.3674 |
| hsa-miR-196a-5p | 0.00051 | 0.1753 |
| hsa-miR-378c | 0.00051 | 0.5844 |
| hsa-miR-374a-5p | 0.00051 | 0.4616 |
| hsa-miR-29b-2-5p | 0.00052 | 0.1782 |
| hsa-miR-140-5p | 0.00052 | 0.4647 |
| hsa-miR-215-5p | 0.00052 | 0.4659 |
| hsa-miR-126-5p | 0.00052 | 0.5286 |
| hsa-miR-192-5p | 0.00054 | 0.5506 |
| hsa-miR-106b-5p | 0.00054 | 0.3771 |
| hsa-miR-93-5p | 0.00062 | 0.4551 |
| hsa-miR-424-5p | 0.00062 | 0.0724 |
| hsa-miR-20a-5p | 0.00063 | 0.4807 |
| hsa-miR-340-5p | 0.00063 | 0.4256 |
| hsa-miR-452-5p | 0.00063 | 0.4363 |
| hsa-miR-7-5p | 0.00064 | 0.0268 |
| hsa-miR-130a-5p | 0.00065 | 0.0174 |
| hsa-miR-183-5p | 0.00066 | 0.1219 |
| hsa-miR-149-5p | 0.00067 | 0.2405 |
| hsa-miR-12136 | 0.00076 | 0.0685 |
| hsa-miR-29a-5p | 0.00076 | 0.1249 |
| hsa-miR-101-5p | 0.00077 | 0.3154 |
| hsa-miR-628-5p | 0.00087 | 0.0873 |
| hsa-miR-32-5p | 0.00089 | 0.2187 |
| hsa-miR-708-5p | 0.00095 | 0.1211 |
| hsa-miR-182-5p | 0.00103 | 0.0549 |
| hsa-miR-335-5p | 0.00109 | 0.1642 |
| hsa-miR-9985 | 0.00118 | 0.1989 |
| hsa-miR-4286 | 0.00122 | 0.0340 |
| hsa-miR-139-5p | 0.00122 | 0.0226 |
