# Supplementary material for: Extracellular vesicle-associated miR-515-5p from adipose tissue regulates placental metabolism and fetal growth in gestational diabetes mellitus
Source: Cardiovasc Diabetol. 2025 May 14;24:205. doi: 10.1186/s12933-025-02739-z (PMC12080180; doi:10.1186/s12933-025-02739-z)
Supplement: Supplementary file 4 — Supplementary Material 4 [file 12933_2025_2739_MOESM4_ESM.docx]

**Supplementary Table 3: Table below shows the miRNA identified between adipose tissue and EVs from NGT.**

| miRNA | log2FoldChange | pvalue |
| --- | --- | --- |
| hsa-miR-5701 | -6.1072 | 8.82896793975456e-11 |
| hsa-miR-4284 | -4.9545 | 3.01968612026932e-07 |
| hsa-miR-150-5p | -4.8766 | 5.01241865375446e-22 |
| hsa-miR-1973 | -4.0980 | 5.07519930565783e-05 |
| hsa-miR-652-5p | -3.9751 | 1.52018830371948e-06 |
| hsa-miR-4485-3p | -3.9470 | 9.39091378755337e-10 |
| hsa-miR-1275 | -3.8840 | 4.40740939642108e-07 |
| hsa-miR-145-5p | -3.7984 | 1.11674393168291e-32 |
| hsa-miR-5585-3p | -3.3806 | 4.88953948863694e-07 |
| hsa-miR-190a-5p | -3.3340 | 1.13470265941733e-12 |
| hsa-miR-5683 | -3.2684 | 1.60320984422894e-07 |
| hsa-miR-18a-5p | -2.7832 | 5.63137465118878e-05 |
| hsa-miR-143-5p | -2.7127 | 5.38968115298315e-08 |
| hsa-miR-33a-5p | -2.5203 | 0.0005 |
| hsa-miR-378a-5p | -2.5027 | 6.50149476118757e-07 |
| hsa-miR-874-5p | -2.4802 | 1.96688784238073e-05 |
| hsa-miR-193a-5p | -2.4775 | 1.50514556517953e-05 |
| hsa-miR-20a-5p | -2.3897 | 4.91568404983291e-09 |
| hsa-miR-215-5p | -2.2917 | 1.15208579346881e-08 |
| hsa-miR-126-5p | -2.2410 | 2.56276793238264e-13 |
| hsa-miR-140-5p | -2.1977 | 8.7448745742771e-07 |
| hsa-miR-135b-5p | -2.1276 | 0.0081 |
| hsa-miR-4521 | -2.1064 | 0.0100 |
| hsa-miR-29c-5p | -2.0422 | 1.75652239441861e-05 |
| hsa-miR-484 | -2.0284 | 8.6701982352431e-10 |
| hsa-miR-210-5p | -2.0132 | 0.0130 |
| hsa-miR-6504-5p | -1.9638 | 0.0142 |
| hsa-miR-4662a-5p | -1.8118 | 0.0118 |
| hsa-miR-376a-5p | -1.7492 | 0.0022 |
| hsa-miR-576-5p | -1.7473 | 0.0074 |
| hsa-miR-139-5p | -1.7399 | 1.10662770426197e-06 |
| hsa-miR-30b-5p | -1.7081 | 2.16188896199519e-06 |
| hsa-miR-7705 | -1.6825 | 0.0257 |
| hsa-miR-30e-5p | -1.6578 | 1.17879314091598e-05 |
| hsa-miR-130b-5p | -1.6394 | 0.0131 |
| hsa-miR-29a-5p | -1.6318 | 0.0126 |
| hsa-miR-500a-5p | -1.6006 | 0.0343 |
| hsa-miR-324-5p | -1.5902 | 0.0007 |
| hsa-miR-30c-5p | -1.5502 | 0.0011 |
| hsa-miR-548ba | -1.5306 | 0.0130 |
| hsa-miR-1306-5p | -1.5302 | 0.0381 |
| hsa-miR-3613-5p | -1.5007 | 0.0033 |
| hsa-miR-1277-5p | -1.4880 | 0.0136 |
| hsa-miR-4531 | -1.4653 | 0.0470 |
| hsa-miR-504-5p | -1.4522 | 0.0067 |
| hsa-miR-142-5p | -1.4520 | 0.0005 |
| hsa-miR-9-5p | -1.4390 | 0.0086 |
| hsa-miR-125a-5p | -1.4292 | 0.0146 |
| hsa-miR-671-5p | -1.3830 | 0.0479 |
| hsa-miR-340-5p | -1.3761 | 9.66674958892612e-05 |
| hsa-miR-144-5p | -1.3507 | 0.0042 |
| hsa-miR-29b-2-5p | -1.3501 | 0.0493 |
| hsa-miR-26b-5p | -1.2897 | 0.0004 |
| hsa-miR-362-5p | -1.2827 | 0.0175 |
| hsa-miR-106a-5p | -1.2660 | 0.0686 |
| hsa-miR-4455 | -1.2616 | 0.0758 |
| hsa-miR-1260a | -1.2536 | 0.0113 |
| hsa-miR-582-5p | -1.2457 | 0.0315 |
| hsa-miR-4767 | -1.2302 | 0.0814 |
| hsa-miR-22-5p | -1.1995 | 0.0146 |
| hsa-miR-1260b | -1.1987 | 0.0117 |
| hsa-miR-1185-5p | -1.1755 | 0.0925 |
| hsa-miR-24-2-5p | -1.1582 | 0.0099 |
| hsa-miR-891a-5p | -1.1496 | 0.0625 |
| hsa-miR-32-5p | -1.1428 | 0.0044 |
| hsa-miR-24-1-5p | -1.1402 | 0.0864 |
| hsa-miR-195-5p | -1.1251 | 4.21565051367955e-05 |
| hsa-miR-500b-5p | -1.1196 | 0.1045 |
| hsa-miR-5690 | -1.1114 | 0.1074 |
| hsa-miR-2355-5p | -1.0990 | 0.0679 |
| hsa-miR-511-5p | -1.0921 | 0.0927 |
| hsa-miR-377-5p | -1.0764 | 0.0460 |
| hsa-miR-3059-5p | -1.0438 | 0.1245 |
| hsa-miR-330-5p | -1.0393 | 0.0463 |
| hsa-miR-497-5p | -1.0376 | 0.0078 |
| hsa-miR-4454 | -1.0068 | 0.0333 |
| hsa-miR-202-5p | -1.0059 | 0.1367 |
| hsa-miR-7-5p | -0.9863 | 0.1418 |
| hsa-miR-17-5p | -0.9850 | 0.0026 |
| hsa-let-7g-5p | -0.9103 | 0.0063 |
| hsa-miR-1261 | -0.8867 | 0.1835 |
| hsa-miR-31-5p | -0.8634 | 0.0797 |
| hsa-miR-30a-5p | -0.8408 | 0.0054 |
| hsa-miR-18b-5p | -0.8403 | 0.2039 |
| hsa-miR-106b-5p | -0.8357 | 0.0539 |
| hsa-miR-196b-5p | -0.8246 | 0.0808 |
| hsa-miR-93-5p | -0.8242 | 0.0049 |
| hsa-miR-30d-5p | -0.7992 | 0.0498 |
| hsa-miR-1248 | -0.7981 | 0.2170 |
| hsa-miR-299-5p | -0.7943 | 0.1901 |
| hsa-miR-365a-5p | -0.7912 | 0.2039 |
| hsa-miR-374a-5p | -0.7897 | 0.0138 |
| hsa-miR-642a-5p | -0.7875 | 0.2227 |
| hsa-miR-125b-5p | -0.7633 | 0.0823 |
| hsa-miR-345-5p | -0.7547 | 0.0886 |
| hsa-miR-641 | -0.7483 | 0.2159 |
| hsa-miR-101-5p | -0.7395 | 0.0420 |
| hsa-miR-103a-2-5p | -0.7332 | 0.2377 |
| hsa-miR-337-5p | -0.7282 | 0.2516 |
| hsa-miR-4326 | -0.7254 | 0.2408 |
| hsa-miR-3147 | -0.6999 | 0.2669 |
| hsa-miR-4661-5p | -0.6935 | 0.2725 |
| hsa-miR-452-5p | -0.6924 | 0.0306 |
| hsa-miR-211-5p | -0.6909 | 0.2801 |
| hsa-miR-659-5p | -0.6843 | 0.2893 |
| hsa-miR-6516-5p | -0.6723 | 0.2835 |
| hsa-miR-98-5p | -0.6699 | 0.0154 |
| hsa-miR-224-5p | -0.6625 | 0.0663 |
| hsa-miR-10399-5p | -0.6444 | 0.2235 |
| hsa-miR-585-5p | -0.6331 | 0.3247 |
| hsa-miR-33b-5p | -0.6242 | 0.2061 |
| hsa-miR-624-5p | -0.6221 | 0.3169 |
| hsa-miR-653-5p | -0.6210 | 0.2154 |
| hsa-miR-27b-5p | -0.6060 | 0.1047 |
| hsa-miR-1268a | -0.5917 | 0.3548 |
| hsa-miR-20b-5p | -0.5903 | 0.2877 |
| hsa-miR-3152-5p | -0.5769 | 0.3646 |
| hsa-miR-590-5p | -0.5661 | 0.3729 |
| hsa-miR-148b-5p | -0.5632 | 0.2493 |
| hsa-miR-194-5p | -0.5610 | 0.1721 |
| hsa-miR-19b-1-5p | -0.5399 | 0.3752 |
| hsa-miR-1908-5p | -0.5186 | 0.3905 |
| hsa-miR-425-5p | -0.5101 | 0.1259 |
| hsa-miR-136-5p | -0.5097 | 0.1172 |
| hsa-miR-2277-5p | -0.5090 | 0.3816 |
| hsa-miR-28-5p | -0.5013 | 0.1722 |
| hsa-miR-3942-5p | -0.5007 | 0.4046 |
| hsa-miR-26a-5p | -0.5002 | 0.2037 |
| hsa-miR-516a-5p | -0.4973 | 0.4316 |
| hsa-miR-34b-5p | -0.4965 | 0.4083 |
| hsa-miR-6502-5p | -0.4904 | 0.4363 |
| hsa-miR-4286 | -0.4788 | 0.2655 |
| hsa-miR-95-5p | -0.4778 | 0.4099 |
| hsa-miR-374b-5p | -0.4732 | 0.2400 |
| hsa-miR-5588-5p | -0.4716 | 0.4526 |
| hsa-miR-296-5p | -0.4565 | 0.3984 |
| hsa-miR-885-5p | -0.4497 | 0.4478 |
| hsa-miR-6503-5p | -0.4459 | 0.4778 |
| hsa-miR-379-5p | -0.4424 | 0.3802 |
| hsa-miR-548n | -0.4414 | 0.4747 |
| hsa-miR-888-5p | -0.4413 | 0.4533 |
| hsa-miR-3173-5p | -0.4411 | 0.4556 |
| hsa-miR-7977 | -0.4349 | 0.3604 |
| hsa-miR-5699-5p | -0.4346 | 0.4894 |
| hsa-miR-3157-5p | -0.4327 | 0.4468 |
| hsa-miR-3194-5p | -0.4297 | 0.4487 |
| hsa-miR-301a-5p | -0.4270 | 0.4685 |
| hsa-miR-505-5p | -0.4175 | 0.4995 |
| hsa-miR-23a-5p | -0.4120 | 0.5112 |
| hsa-miR-181c-5p | -0.4088 | 0.1356 |
| hsa-miR-9985 | -0.4058 | 0.2463 |
| hsa-miR-21-5p | -0.3913 | 0.2180 |
| hsa-miR-6724-5p | -0.3879 | 0.5356 |
| hsa-miR-616-5p | -0.3872 | 0.5212 |
| hsa-miR-1268b | -0.3811 | 0.5405 |
| hsa-miR-204-5p | -0.3726 | 0.4696 |
| hsa-miR-216a-5p | -0.3630 | 0.5613 |
| hsa-miR-4463 | -0.3595 | 0.5333 |
| hsa-miR-4775 | -0.3594 | 0.5242 |
| hsa-miR-449a | -0.3588 | 0.5600 |
| hsa-let-7a-5p | -0.3581 | 0.3246 |
| hsa-miR-4772-5p | -0.3578 | 0.5175 |
| hsa-miR-376a-2-5p | -0.3547 | 0.5368 |
| hsa-miR-4636 | -0.3473 | 0.5459 |
| hsa-miR-627-5p | -0.3453 | 0.5660 |
| hsa-miR-6854-5p | -0.3445 | 0.5488 |
| hsa-miR-1468-5p | -0.3378 | 0.5319 |
| hsa-miR-135a-5p | -0.3262 | 0.5510 |
| hsa-miR-153-5p | -0.3251 | 0.5681 |
| hsa-miR-548q | -0.3217 | 0.6054 |
| hsa-miR-338-5p | -0.3060 | 0.5847 |
| hsa-miR-3195 | -0.3021 | 0.4654 |
| hsa-miR-346 | -0.2901 | 0.6332 |
| hsa-miR-1910-5p | -0.2896 | 0.6314 |
| hsa-miR-875-5p | -0.2782 | 0.6013 |
| hsa-miR-491-5p | -0.2773 | 0.6550 |
| hsa-miR-556-5p | -0.2616 | 0.6222 |
| hsa-miR-605-5p | -0.2616 | 0.6222 |
| hsa-miR-612 | -0.2596 | 0.6408 |
| hsa-miR-625-5p | -0.2515 | 0.6146 |
| hsa-miR-4443 | -0.2509 | 0.6348 |
| hsa-miR-188-5p | -0.2509 | 0.6341 |
| hsa-miR-378d | -0.2463 | 0.6056 |
| hsa-miR-708-5p | -0.2441 | 0.5239 |
| hsa-miR-4999-5p | -0.2434 | 0.6599 |
| hsa-miR-361-5p | -0.2411 | 0.3954 |
| hsa-miR-887-5p | -0.2387 | 0.6807 |
| hsa-miR-618 | -0.2384 | 0.7000 |
| hsa-miR-10393-3p | -0.2116 | 0.6822 |
| hsa-miR-3690 | -0.2116 | 0.6822 |
| hsa-miR-371a-5p | -0.2114 | 0.7242 |
| hsa-miR-132-5p | -0.2094 | 0.6620 |
| hsa-miR-539-5p | -0.2051 | 0.7395 |
| hsa-miR-10396b-3p | -0.2022 | 0.7437 |
| hsa-miR-450a-5p | -0.2022 | 0.6740 |
| hsa-miR-200b-5p | -0.2012 | 0.7388 |
| hsa-miR-1255a | -0.2006 | 0.7375 |
| hsa-miR-3163 | -0.2006 | 0.7375 |
| hsa-miR-149-5p | -0.1918 | 0.6825 |
| hsa-miR-3064-5p | -0.1893 | 0.7107 |
| hsa-miR-3174 | -0.1893 | 0.7107 |
| hsa-miR-499a-5p | -0.1883 | 0.7556 |
| hsa-miR-299-3p | -0.1852 | 0.6456 |
| hsa-miR-3130-5p | -0.1812 | 0.7131 |
| hsa-miR-152-5p | -0.1771 | 0.6997 |
| hsa-miR-4709-5p | -0.1766 | 0.7692 |
| hsa-miR-5708 | -0.1725 | 0.7317 |
| hsa-miR-3614-5p | -0.1721 | 0.7801 |
| hsa-miR-221-5p | -0.1653 | 0.6832 |
| hsa-miR-550a-3-5p | -0.1646 | 0.7861 |
| hsa-miR-29b-1-5p | -0.1604 | 0.7922 |
| hsa-miR-6513-5p | -0.1582 | 0.7974 |
| hsa-miR-1243 | -0.1542 | 0.7712 |
| hsa-miR-1266-5p | -0.1542 | 0.7712 |
| hsa-miR-1292-5p | -0.1542 | 0.7712 |
| hsa-miR-4768-5p | -0.1542 | 0.7712 |
| hsa-miR-5684 | -0.1542 | 0.7712 |
| hsa-miR-23b-5p | -0.1488 | 0.8058 |
| hsa-miR-5689 | -0.1464 | 0.8120 |
| hsa-miR-545-5p | -0.1442 | 0.8152 |
| hsa-miR-628-5p | -0.1423 | 0.7683 |
| hsa-miR-454-5p | -0.1360 | 0.7850 |
| hsa-miR-450b-5p | -0.1331 | 0.7413 |
| hsa-miR-3181 | -0.1321 | 0.7876 |
| hsa-miR-4742-5p | -0.1321 | 0.7876 |
| hsa-miR-548ar-3p | -0.1321 | 0.7876 |
| hsa-miR-6508-3p | -0.1321 | 0.7876 |
| hsa-miR-6796-5p | -0.1321 | 0.7876 |
| hsa-miR-5002-5p | -0.1284 | 0.8327 |
| hsa-miR-5088-5p | -0.1281 | 0.7938 |
| hsa-miR-15b-5p | -0.1263 | 0.7262 |
| hsa-miR-4690-3p | -0.1232 | 0.8014 |
| hsa-miR-4762-5p | -0.1232 | 0.8014 |
| hsa-miR-7702 | -0.1232 | 0.8014 |
| hsa-miR-19a-5p | -0.1191 | 0.8078 |
| hsa-miR-4634 | -0.1191 | 0.8078 |
| hsa-miR-4791 | -0.1191 | 0.8078 |
| hsa-miR-3159 | -0.1119 | 0.8192 |
| hsa-miR-4426 | -0.1119 | 0.8192 |
| hsa-miR-6877-3p | -0.1119 | 0.8192 |
| hsa-miR-6886-5p | -0.1119 | 0.8192 |
| hsa-miR-1180-5p | -0.1097 | 0.8225 |
| hsa-miR-3674 | -0.1097 | 0.8225 |
| hsa-miR-5696 | -0.1097 | 0.8225 |
| hsa-miR-6852-5p | -0.1095 | 0.8526 |
| hsa-miR-550a-5p | -0.1062 | 0.8617 |
| hsa-miR-219a-5p | -0.0920 | 0.8808 |
| hsa-miR-424-5p | -0.0916 | 0.8203 |
| hsa-miR-873-5p | -0.0909 | 0.8617 |
| hsa-miR-6875-5p | -0.0901 | 0.8737 |
| hsa-miR-34a-5p | -0.0900 | 0.8028 |
| hsa-miR-4433b-5p | -0.0852 | 0.8805 |
| hsa-miR-577 | -0.0751 | 0.8971 |
| hsa-miR-6754-5p | -0.0725 | 0.8975 |
| hsa-miR-34c-5p | -0.0677 | 0.8672 |
| hsa-miR-4481 | -0.0605 | 0.9011 |
| hsa-miR-4679 | -0.0605 | 0.9011 |
| hsa-miR-4714-5p | -0.0605 | 0.9011 |
| hsa-miR-5000-5p | -0.0605 | 0.9011 |
| hsa-miR-5009-5p | -0.0605 | 0.9011 |
| hsa-miR-6499-5p | -0.0605 | 0.9011 |
| hsa-miR-548ab | -0.0600 | 0.9211 |
| hsa-miR-1256 | -0.0578 | 0.9055 |
| hsa-miR-4517 | -0.0578 | 0.9055 |
| hsa-miR-6872-5p | -0.0578 | 0.9055 |
| hsa-miR-6894-3p | -0.0578 | 0.9055 |
| hsa-miR-7152-3p | -0.0578 | 0.9055 |
| hsa-miR-767-5p | -0.0578 | 0.9055 |
| hsa-miR-548i | -0.0571 | 0.9257 |
| hsa-miR-3617-5p | -0.0550 | 0.9100 |
| hsa-miR-380-5p | -0.0550 | 0.9100 |
| hsa-miR-4458 | -0.0550 | 0.9100 |
| hsa-miR-4794 | -0.0550 | 0.9100 |
| hsa-miR-548y | -0.0550 | 0.9100 |
| hsa-miR-6727-5p | -0.0550 | 0.9100 |
| hsa-miR-6789-5p | -0.0550 | 0.9100 |
| hsa-miR-181d-5p | -0.0546 | 0.8649 |
| hsa-miR-501-5p | -0.0518 | 0.9313 |
| hsa-miR-370-5p | -0.0479 | 0.9366 |
| hsa-miR-4524a-5p | -0.0453 | 0.9411 |
| hsa-miR-219b-5p | -0.0452 | 0.9259 |
| hsa-miR-320a-5p | -0.0452 | 0.9259 |
| hsa-miR-6751-5p | -0.0452 | 0.9259 |
| hsa-miR-7845-5p | -0.0452 | 0.9259 |
| hsa-miR-216b-5p | -0.0423 | 0.9306 |
| hsa-miR-3652 | -0.0423 | 0.9306 |
| hsa-miR-4515 | -0.0423 | 0.9306 |
| hsa-miR-6829-5p | -0.0423 | 0.9306 |
| hsa-miR-7155-3p | -0.0423 | 0.9306 |
| hsa-miR-889-5p | -0.0423 | 0.9306 |
| hsa-miR-942-5p | -0.0406 | 0.9460 |
| hsa-miR-185-5p | -0.0379 | 0.9426 |
| hsa-miR-133a-5p | -0.0289 | 0.9524 |
| hsa-miR-4725-5p | -0.0289 | 0.9524 |
| hsa-miR-7151-3p | -0.0289 | 0.9524 |
| hsa-miR-551b-5p | -0.0264 | 0.9621 |
| hsa-miR-4687-5p | -0.0237 | 0.9660 |
| hsa-miR-6512-5p | -0.0237 | 0.9660 |
| hsa-miR-4485-5p | -0.0147 | 0.9805 |
| hsa-miR-10394-5p | -0.0129 | 0.9822 |
| hsa-miR-217-5p | -0.0087 | 0.9875 |
| hsa-miR-192-5p | -0.0042 | 0.9892 |
| hsa-miR-561-5p | -0.0009 | 0.9982 |
| hsa-miR-223-5p | 0.0039 | 0.9942 |
| hsa-miR-6730-5p | 0.0040 | 0.9944 |
| hsa-miR-1304-5p | 0.0097 | 0.9865 |
| hsa-miR-134-5p | 0.0190 | 0.9662 |
| hsa-miR-3161 | 0.0233 | 0.9674 |
| hsa-miR-6761-5p | 0.0242 | 0.9686 |
| hsa-miR-455-5p | 0.0259 | 0.9382 |
| hsa-miR-664b-5p | 0.0273 | 0.9644 |
| hsa-miR-4746-5p | 0.0310 | 0.9591 |
| hsa-miR-3133 | 0.0332 | 0.9507 |
| hsa-miR-151a-5p | 0.0380 | 0.9271 |
| hsa-miR-191-5p | 0.0407 | 0.8951 |
| hsa-miR-3677-3p | 0.0436 | 0.9396 |
| hsa-miR-1267 | 0.0589 | 0.9030 |
| hsa-miR-1302 | 0.0589 | 0.9030 |
| hsa-miR-1909-5p | 0.0589 | 0.9030 |
| hsa-miR-3131 | 0.0589 | 0.9030 |
| hsa-miR-3135a | 0.0589 | 0.9030 |
| hsa-miR-3142 | 0.0589 | 0.9030 |
| hsa-miR-3165 | 0.0589 | 0.9030 |
| hsa-miR-3169 | 0.0589 | 0.9030 |
| hsa-miR-3183 | 0.0589 | 0.9030 |
| hsa-miR-3677-5p | 0.0589 | 0.9030 |
| hsa-miR-3681-5p | 0.0589 | 0.9030 |
| hsa-miR-374c-3p | 0.0589 | 0.9030 |
| hsa-miR-3944-5p | 0.0589 | 0.9030 |
| hsa-miR-4301 | 0.0589 | 0.9030 |
| hsa-miR-4323 | 0.0589 | 0.9030 |
| hsa-miR-4445-5p | 0.0589 | 0.9030 |
| hsa-miR-4538 | 0.0589 | 0.9030 |
| hsa-miR-4651 | 0.0589 | 0.9030 |
| hsa-miR-4676-5p | 0.0589 | 0.9030 |
| hsa-miR-4729 | 0.0589 | 0.9030 |
| hsa-miR-4802-5p | 0.0589 | 0.9030 |
| hsa-miR-5008-5p | 0.0589 | 0.9030 |
| hsa-miR-517-5p | 0.0589 | 0.9030 |
| hsa-miR-548a-5p | 0.0589 | 0.9030 |
| hsa-miR-548as-5p | 0.0589 | 0.9030 |
| hsa-miR-549a-5p | 0.0589 | 0.9030 |
| hsa-miR-5581-3p | 0.0589 | 0.9030 |
| hsa-miR-6803-5p | 0.0589 | 0.9030 |
| hsa-miR-6850-3p | 0.0589 | 0.9030 |
| hsa-miR-7109-3p | 0.0589 | 0.9030 |
| hsa-miR-7111-5p | 0.0589 | 0.9030 |
| hsa-miR-7151-5p | 0.0589 | 0.9030 |
| hsa-miR-101-2-5p | 0.0589 | 0.9030 |
| hsa-miR-10397-5p | 0.0589 | 0.9030 |
| hsa-miR-103a-1-5p | 0.0589 | 0.9030 |
| hsa-miR-124-5p | 0.0589 | 0.9030 |
| hsa-miR-1249-5p | 0.0589 | 0.9030 |
| hsa-miR-1265 | 0.0589 | 0.9030 |
| hsa-miR-1284 | 0.0589 | 0.9030 |
| hsa-miR-141-5p | 0.0589 | 0.9030 |
| hsa-miR-1914-5p | 0.0589 | 0.9030 |
| hsa-miR-2115-5p | 0.0589 | 0.9030 |
| hsa-miR-3120-5p | 0.0589 | 0.9030 |
| hsa-miR-3136-5p | 0.0589 | 0.9030 |
| hsa-miR-3144-5p | 0.0589 | 0.9030 |
| hsa-miR-3171 | 0.0589 | 0.9030 |
| hsa-miR-372-5p | 0.0589 | 0.9030 |
| hsa-miR-376b-5p | 0.0589 | 0.9030 |
| hsa-miR-376c-5p | 0.0589 | 0.9030 |
| hsa-miR-3976 | 0.0589 | 0.9030 |
| hsa-miR-4327 | 0.0589 | 0.9030 |
| hsa-miR-4482-5p | 0.0589 | 0.9030 |
| hsa-miR-4498 | 0.0589 | 0.9030 |
| hsa-miR-4536-5p | 0.0589 | 0.9030 |
| hsa-miR-4639-5p | 0.0589 | 0.9030 |
| hsa-miR-4648 | 0.0589 | 0.9030 |
| hsa-miR-4657 | 0.0589 | 0.9030 |
| hsa-miR-4663 | 0.0589 | 0.9030 |
| hsa-miR-4690-5p | 0.0589 | 0.9030 |
| hsa-miR-4720-5p | 0.0589 | 0.9030 |
| hsa-miR-4731-5p | 0.0589 | 0.9030 |
| hsa-miR-4753-5p | 0.0589 | 0.9030 |
| hsa-miR-494-5p | 0.0589 | 0.9030 |
| hsa-miR-5090 | 0.0589 | 0.9030 |
| hsa-miR-510-5p | 0.0589 | 0.9030 |
| hsa-miR-548t-5p | 0.0589 | 0.9030 |
| hsa-miR-552-5p | 0.0589 | 0.9030 |
| hsa-miR-5579-5p | 0.0589 | 0.9030 |
| hsa-miR-5586-3p | 0.0589 | 0.9030 |
| hsa-miR-5587-5p | 0.0589 | 0.9030 |
| hsa-miR-573 | 0.0589 | 0.9030 |
| hsa-miR-601 | 0.0589 | 0.9030 |
| hsa-miR-642b-5p | 0.0589 | 0.9030 |
| hsa-miR-650 | 0.0589 | 0.9030 |
| hsa-miR-6508-5p | 0.0589 | 0.9030 |
| hsa-miR-6721-5p | 0.0589 | 0.9030 |
| hsa-miR-6734-5p | 0.0589 | 0.9030 |
| hsa-miR-6772-5p | 0.0589 | 0.9030 |
| hsa-miR-6774-5p | 0.0589 | 0.9030 |
| hsa-miR-6827-5p | 0.0589 | 0.9030 |
| hsa-miR-6868-3p | 0.0589 | 0.9030 |
| hsa-miR-6874-5p | 0.0589 | 0.9030 |
| hsa-miR-6882-5p | 0.0589 | 0.9030 |
| hsa-miR-7150 | 0.0589 | 0.9030 |
| hsa-miR-7853-5p | 0.0589 | 0.9030 |
| hsa-miR-802 | 0.0589 | 0.9030 |
| hsa-miR-921 | 0.0589 | 0.9030 |
| hsa-miR-10395-5p | 0.0589 | 0.9030 |
| hsa-miR-1225-5p | 0.0589 | 0.9030 |
| hsa-miR-203b-5p | 0.0589 | 0.9030 |
| hsa-miR-3150a-5p | 0.0589 | 0.9030 |
| hsa-miR-3180-5p | 0.0589 | 0.9030 |
| hsa-miR-323a-5p | 0.0589 | 0.9030 |
| hsa-miR-374c-5p | 0.0589 | 0.9030 |
| hsa-miR-381-5p | 0.0589 | 0.9030 |
| hsa-miR-3925-5p | 0.0589 | 0.9030 |
| hsa-miR-3929 | 0.0589 | 0.9030 |
| hsa-miR-4317 | 0.0589 | 0.9030 |
| hsa-miR-4505 | 0.0589 | 0.9030 |
| hsa-miR-4642 | 0.0589 | 0.9030 |
| hsa-miR-4706 | 0.0589 | 0.9030 |
| hsa-miR-4707-5p | 0.0589 | 0.9030 |
| hsa-miR-4757-5p | 0.0589 | 0.9030 |
| hsa-miR-4796-5p | 0.0589 | 0.9030 |
| hsa-miR-4798-5p | 0.0589 | 0.9030 |
| hsa-miR-487b-5p | 0.0589 | 0.9030 |
| hsa-miR-509-5p | 0.0589 | 0.9030 |
| hsa-miR-519d-5p | 0.0589 | 0.9030 |
| hsa-miR-5691 | 0.0589 | 0.9030 |
| hsa-miR-579-5p | 0.0589 | 0.9030 |
| hsa-miR-588 | 0.0589 | 0.9030 |
| hsa-miR-623 | 0.0589 | 0.9030 |
| hsa-miR-655-5p | 0.0589 | 0.9030 |
| hsa-miR-6732-3p | 0.0589 | 0.9030 |
| hsa-miR-6750-5p | 0.0589 | 0.9030 |
| hsa-miR-6797-5p | 0.0589 | 0.9030 |
| hsa-miR-6847-5p | 0.0589 | 0.9030 |
| hsa-miR-6869-3p | 0.0589 | 0.9030 |
| hsa-miR-7155-5p | 0.0589 | 0.9030 |
| hsa-miR-891b | 0.0589 | 0.9030 |
| hsa-miR-411-5p | 0.0598 | 0.8881 |
| hsa-miR-3612 | 0.0603 | 0.9037 |
| hsa-miR-2467-5p | 0.0605 | 0.9116 |
| hsa-miR-1296-5p | 0.0642 | 0.8797 |
| hsa-miR-218-5p | 0.0671 | 0.8646 |
| hsa-miR-3619-5p | 0.0756 | 0.8887 |
| hsa-miR-597-5p | 0.0756 | 0.8887 |
| hsa-miR-9902 | 0.0756 | 0.8887 |
| hsa-miR-6892-5p | 0.0798 | 0.8953 |
| hsa-miR-509-3-5p | 0.0844 | 0.8637 |
| hsa-miR-548aw | 0.0867 | 0.8682 |
| hsa-miR-548aq-3p | 0.0876 | 0.8845 |
| hsa-miR-1283 | 0.0909 | 0.8737 |
| hsa-miR-3128 | 0.0918 | 0.8630 |
| hsa-miR-527 | 0.0934 | 0.8620 |
| hsa-miR-1276 | 0.1012 | 0.8663 |
| hsa-miR-3145-5p | 0.1042 | 0.8518 |
| hsa-miR-3620-5p | 0.1069 | 0.8623 |
| hsa-miR-382-5p | 0.1083 | 0.8266 |
| hsa-miR-329-5p | 0.1090 | 0.8469 |
| hsa-miR-502-5p | 0.1152 | 0.8409 |
| hsa-miR-770-5p | 0.1159 | 0.8493 |
| hsa-miR-6514-5p | 0.1164 | 0.8489 |
| hsa-miR-2116-5p | 0.1194 | 0.8344 |
| hsa-miR-1298-5p | 0.1205 | 0.8287 |
| hsa-miR-548ag | 0.1209 | 0.8408 |
| hsa-miR-155-5p | 0.1238 | 0.7365 |
| hsa-miR-1245b-5p | 0.1259 | 0.8234 |
| hsa-miR-2114-5p | 0.1277 | 0.8359 |
| hsa-miR-10396a-3p | 0.1295 | 0.8182 |
| hsa-miR-3665 | 0.1295 | 0.8182 |
| hsa-miR-4786-5p | 0.1295 | 0.8182 |
| hsa-miR-433-5p | 0.1298 | 0.8332 |
| hsa-miR-3661 | 0.1303 | 0.8257 |
| hsa-let-7b-5p | 0.1347 | 0.6366 |
| hsa-miR-3928-3p | 0.1561 | 0.8003 |
| hsa-miR-3934-5p | 0.1633 | 0.7910 |
| hsa-miR-130a-5p | 0.1638 | 0.7828 |
| hsa-miR-1224-5p | 0.1691 | 0.7277 |
| hsa-miR-4289 | 0.1691 | 0.7277 |
| hsa-miR-548l | 0.1691 | 0.7277 |
| hsa-miR-6132 | 0.1691 | 0.7277 |
| hsa-miR-6507-5p | 0.1691 | 0.7277 |
| hsa-miR-6510-5p | 0.1691 | 0.7277 |
| hsa-miR-11399 | 0.1691 | 0.7277 |
| hsa-miR-1228-5p | 0.1691 | 0.7277 |
| hsa-miR-2278 | 0.1691 | 0.7277 |
| hsa-miR-4638-3p | 0.1691 | 0.7277 |
| hsa-miR-512-5p | 0.1691 | 0.7277 |
| hsa-miR-6729-5p | 0.1691 | 0.7277 |
| hsa-miR-4782-5p | 0.1691 | 0.7277 |
| hsa-miR-508-5p | 0.1691 | 0.7277 |
| hsa-miR-548ai | 0.1691 | 0.7277 |
| hsa-miR-570-5p | 0.1691 | 0.7277 |
| hsa-miR-7152-5p | 0.1691 | 0.7277 |
| hsa-miR-584-5p | 0.1889 | 0.6552 |
| hsa-let-7d-5p | 0.1900 | 0.5556 |
| hsa-miR-369-5p | 0.1953 | 0.6702 |
| hsa-miR-138-5p | 0.1997 | 0.7461 |
| hsa-miR-200a-5p | 0.2003 | 0.7414 |
| hsa-miR-3199 | 0.2014 | 0.7440 |
| hsa-miR-4435 | 0.2055 | 0.6848 |
| hsa-miR-580-5p | 0.2055 | 0.6848 |
| hsa-miR-422a | 0.2077 | 0.6948 |
| hsa-miR-548ax | 0.2097 | 0.6808 |
| hsa-miR-4670-5p | 0.2106 | 0.6799 |
| hsa-miR-488-5p | 0.2106 | 0.6799 |
| hsa-miR-6770-3p | 0.2119 | 0.7236 |
| hsa-miR-6509-5p | 0.2189 | 0.6977 |
| hsa-miR-769-5p | 0.2194 | 0.4416 |
| hsa-miR-518a-5p | 0.2208 | 0.7064 |
| hsa-miR-11401 | 0.2235 | 0.6678 |
| hsa-miR-3916 | 0.2235 | 0.6678 |
| hsa-miR-4740-5p | 0.2235 | 0.6678 |
| hsa-miR-1287-5p | 0.2240 | 0.6497 |
| hsa-miR-3125 | 0.2271 | 0.6645 |
| hsa-miR-4770 | 0.2279 | 0.6638 |
| hsa-miR-548an | 0.2279 | 0.6638 |
| hsa-miR-3913-5p | 0.2329 | 0.6621 |
| hsa-miR-431-5p | 0.2400 | 0.6278 |
| hsa-miR-190b-5p | 0.2408 | 0.6604 |
| hsa-miR-519a-5p | 0.2475 | 0.6865 |
| hsa-miR-1291 | 0.2482 | 0.6629 |
| hsa-let-7i-5p | 0.2677 | 0.4630 |
| hsa-miR-127-5p | 0.2695 | 0.4736 |
| hsa-miR-3074-5p | 0.2759 | 0.6522 |
| hsa-miR-3127-5p | 0.2764 | 0.6562 |
| hsa-miR-4668-5p | 0.2768 | 0.5915 |
| hsa-miR-513c-5p | 0.2768 | 0.5915 |
| hsa-miR-4705 | 0.2781 | 0.6531 |
| hsa-miR-6859-5p | 0.2799 | 0.6445 |
| hsa-miR-548k | 0.2845 | 0.5219 |
| hsa-miR-10526-3p | 0.2861 | 0.5590 |
| hsa-miR-1587 | 0.2861 | 0.5590 |
| hsa-miR-3132 | 0.2861 | 0.5590 |
| hsa-miR-3135b | 0.2861 | 0.5590 |
| hsa-miR-3940-5p | 0.2861 | 0.5590 |
| hsa-miR-4309 | 0.2861 | 0.5590 |
| hsa-miR-4467 | 0.2861 | 0.5590 |
| hsa-miR-4478 | 0.2861 | 0.5590 |
| hsa-miR-4523 | 0.2861 | 0.5590 |
| hsa-miR-4776-5p | 0.2861 | 0.5590 |
| hsa-miR-495-5p | 0.2861 | 0.5590 |
| hsa-miR-5001-5p | 0.2861 | 0.5590 |
| hsa-miR-518d-5p | 0.2861 | 0.5590 |
| hsa-miR-520c-5p | 0.2861 | 0.5590 |
| hsa-miR-526a-5p | 0.2861 | 0.5590 |
| hsa-miR-548av-5p | 0.2861 | 0.5590 |
| hsa-miR-5703 | 0.2861 | 0.5590 |
| hsa-miR-6756-5p | 0.2861 | 0.5590 |
| hsa-miR-6834-5p | 0.2861 | 0.5590 |
| hsa-miR-6876-5p | 0.2861 | 0.5590 |
| hsa-miR-6888-5p | 0.2861 | 0.5590 |
| hsa-miR-766-5p | 0.2861 | 0.5590 |
| hsa-miR-7973 | 0.2861 | 0.5590 |
| hsa-miR-939-5p | 0.2861 | 0.5590 |
| hsa-miR-548ae-5p | 0.2988 | 0.6093 |
| hsa-miR-1229-5p | 0.3011 | 0.5393 |
| hsa-miR-1273c | 0.3011 | 0.5393 |
| hsa-miR-1293 | 0.3011 | 0.5393 |
| hsa-miR-3616-5p | 0.3011 | 0.5393 |
| hsa-miR-3679-5p | 0.3011 | 0.5393 |
| hsa-miR-4487 | 0.3011 | 0.5393 |
| hsa-miR-4638-5p | 0.3011 | 0.5393 |
| hsa-miR-4646-5p | 0.3011 | 0.5393 |
| hsa-miR-4647 | 0.3011 | 0.5393 |
| hsa-miR-4711-5p | 0.3011 | 0.5393 |
| hsa-miR-4726-5p | 0.3011 | 0.5393 |
| hsa-miR-4739 | 0.3011 | 0.5393 |
| hsa-miR-4766-5p | 0.3011 | 0.5393 |
| hsa-miR-5094 | 0.3011 | 0.5393 |
| hsa-miR-5581-5p | 0.3011 | 0.5393 |
| hsa-miR-6770-5p | 0.3011 | 0.5393 |
| hsa-miR-6855-5p | 0.3011 | 0.5393 |
| hsa-miR-6861-3p | 0.3011 | 0.5393 |
| hsa-miR-6861-5p | 0.3011 | 0.5393 |
| hsa-miR-6879-5p | 0.3011 | 0.5393 |
| hsa-miR-7160-5p | 0.3011 | 0.5393 |
| hsa-miR-1281 | 0.3011 | 0.5393 |
| hsa-miR-4288 | 0.3011 | 0.5393 |
| hsa-miR-4700-5p | 0.3011 | 0.5393 |
| hsa-miR-4763-5p | 0.3011 | 0.5393 |
| hsa-miR-5089-5p | 0.3011 | 0.5393 |
| hsa-miR-6821-5p | 0.3011 | 0.5393 |
| hsa-miR-6838-5p | 0.3011 | 0.5393 |
| hsa-miR-6880-5p | 0.3011 | 0.5393 |
| hsa-miR-8071 | 0.3011 | 0.5393 |
| hsa-miR-2110 | 0.3058 | 0.5508 |
| hsa-miR-487a-5p | 0.3222 | 0.6050 |
| hsa-let-7e-5p | 0.3377 | 0.3475 |
| hsa-miR-2681-5p | 0.3391 | 0.5708 |
| hsa-miR-3918 | 0.3560 | 0.4711 |
| hsa-miR-4658 | 0.3560 | 0.4711 |
| hsa-miR-525-5p | 0.3560 | 0.4711 |
| hsa-miR-6732-5p | 0.3560 | 0.4711 |
| hsa-miR-6773-5p | 0.3560 | 0.4711 |
| hsa-miR-182-5p | 0.3595 | 0.4591 |
| hsa-miR-541-5p | 0.3635 | 0.5536 |
| hsa-miR-548w | 0.3652 | 0.5430 |
| hsa-miR-212-5p | 0.3682 | 0.4916 |
| hsa-miR-331-5p | 0.3781 | 0.4678 |
| hsa-miR-574-5p | 0.3784 | 0.3767 |
| hsa-miR-10399-3p | 0.3991 | 0.3972 |
| hsa-miR-4507 | 0.4104 | 0.4102 |
| hsa-miR-548ad-5p | 0.4161 | 0.4842 |
| hsa-miR-16-5p | 0.4335 | 0.2659 |
| hsa-miR-4424 | 0.4371 | 0.4331 |
| hsa-miR-10226 | 0.4496 | 0.3765 |
| hsa-miR-378b | 0.4496 | 0.4626 |
| hsa-miR-6755-5p | 0.4499 | 0.4367 |
| hsa-miR-6795-5p | 0.4549 | 0.4331 |
| hsa-miR-1271-5p | 0.4582 | 0.3132 |
| hsa-miR-548j-5p | 0.4586 | 0.4666 |
| hsa-miR-199a-5p | 0.4645 | 0.1872 |
| hsa-miR-516b-5p | 0.4653 | 0.4238 |
| hsa-miR-1273h-5p | 0.4849 | 0.4429 |
| hsa-miR-10392-5p | 0.4868 | 0.4069 |
| hsa-miR-5706 | 0.4868 | 0.4069 |
| hsa-miR-378h | 0.4877 | 0.4380 |
| hsa-miR-518e-5p | 0.4891 | 0.4350 |
| hsa-miR-519b-5p | 0.4891 | 0.4350 |
| hsa-miR-519c-5p | 0.4891 | 0.4350 |
| hsa-miR-522-5p | 0.4891 | 0.4350 |
| hsa-miR-523-5p | 0.4891 | 0.4350 |
| hsa-miR-1469 | 0.4943 | 0.4234 |
| hsa-miR-10401-5p | 0.4963 | 0.3589 |
| hsa-miR-3691-5p | 0.4963 | 0.3589 |
| hsa-miR-6845-5p | 0.4963 | 0.3589 |
| hsa-miR-8061 | 0.4963 | 0.3589 |
| hsa-miR-876-5p | 0.4963 | 0.3589 |
| hsa-miR-378g | 0.5177 | 0.3232 |
| hsa-miR-148a-5p | 0.5191 | 0.1481 |
| hsa-miR-4732-5p | 0.5261 | 0.3601 |
| hsa-miR-548ay-5p | 0.5268 | 0.3691 |
| hsa-miR-3200-5p | 0.5299 | 0.3577 |
| hsa-miR-548az-5p | 0.5327 | 0.3918 |
| hsa-miR-663a | 0.5494 | 0.3880 |
| hsa-miR-1226-5p | 0.5503 | 0.3226 |
| hsa-miR-4743-5p | 0.5503 | 0.3226 |
| hsa-miR-518f-5p | 0.5503 | 0.3226 |
| hsa-miR-520d-5p | 0.5503 | 0.3226 |
| hsa-miR-5585-5p | 0.5503 | 0.3226 |
| hsa-miR-6740-5p | 0.5503 | 0.3226 |
| hsa-miR-6757-5p | 0.5503 | 0.3226 |
| hsa-miR-6865-5p | 0.5503 | 0.3226 |
| hsa-miR-3155b | 0.5522 | 0.3016 |
| hsa-miR-3170 | 0.5522 | 0.3016 |
| hsa-miR-4747-5p | 0.5522 | 0.3016 |
| hsa-miR-4769-5p | 0.5522 | 0.3016 |
| hsa-miR-181a-5p | 0.5537 | 0.0733 |
| hsa-miR-651-5p | 0.5580 | 0.2501 |
| hsa-miR-154-5p | 0.5615 | 0.1969 |
| hsa-miR-6501-5p | 0.5704 | 0.3712 |
| hsa-let-7f-5p | 0.5844 | 0.0667 |
| hsa-miR-92b-5p | 0.6028 | 0.3145 |
| hsa-miR-7976 | 0.6101 | 0.3395 |
| hsa-miR-744-5p | 0.6192 | 0.1521 |
| hsa-miR-548d-5p | 0.6222 | 0.2267 |
| hsa-miR-214-5p | 0.6313 | 0.0883 |
| hsa-miR-449c-5p | 0.6399 | 0.3128 |
| hsa-miR-548ak | 0.6638 | 0.2806 |
| hsa-miR-4649-5p | 0.6639 | 0.2515 |
| hsa-miR-6779-5p | 0.6639 | 0.2515 |
| hsa-miR-7856-5p | 0.6639 | 0.2515 |
| hsa-miR-383-5p | 0.6642 | 0.3016 |
| hsa-miR-5189-5p | 0.6655 | 0.2995 |
| hsa-miR-664a-5p | 0.6666 | 0.1365 |
| hsa-miR-519a-2-5p | 0.6686 | 0.2781 |
| hsa-miR-520b-5p | 0.6686 | 0.2781 |
| hsa-miR-449b-5p | 0.6697 | 0.2775 |
| hsa-miR-3926 | 0.6757 | 0.2457 |
| hsa-miR-4667-5p | 0.6854 | 0.2876 |
| hsa-miR-183-5p | 0.6881 | 0.2220 |
| hsa-miR-6735-5p | 0.6897 | 0.2807 |
| hsa-miR-205-5p | 0.6940 | 0.2840 |
| hsa-miR-6505-5p | 0.7021 | 0.2579 |
| hsa-miR-339-5p | 0.7036 | 0.1905 |
| hsa-miR-15a-5p | 0.7051 | 0.0958 |
| hsa-miR-4429 | 0.7067 | 0.2556 |
| hsa-miR-10527-5p | 0.7092 | 0.2747 |
| hsa-miR-181b-5p | 0.7171 | 0.0383 |
| hsa-miR-6891-5p | 0.7391 | 0.2143 |
| hsa-miR-676-5p | 0.7429 | 0.2547 |
| hsa-miR-409-5p | 0.7451 | 0.1682 |
| hsa-miR-1323 | 0.7494 | 0.2511 |
| hsa-miR-532-5p | 0.7619 | 0.0077 |
| hsa-miR-548at-5p | 0.7727 | 0.2348 |
| hsa-miR-4497 | 0.7796 | 0.2083 |
| hsa-miR-6746-5p | 0.7893 | 0.2264 |
| hsa-miR-6780a-5p | 0.7902 | 0.2127 |
| hsa-miR-371b-5p | 0.7907 | 0.1770 |
| hsa-miR-6511b-5p | 0.8012 | 0.2190 |
| hsa-miR-186-5p | 0.8093 | 0.0039 |
| hsa-miR-3202 | 0.8124 | 0.2029 |
| hsa-miR-758-5p | 0.8173 | 0.2009 |
| hsa-miR-128-1-5p | 0.8420 | 0.1492 |
| hsa-miR-25-5p | 0.8563 | 0.1082 |
| hsa-miR-5010-5p | 0.8567 | 0.1948 |
| hsa-miR-6511a-5p | 0.8638 | 0.1859 |
| hsa-miR-3180 | 0.8648 | 0.1798 |
| hsa-miR-3180-3p | 0.8648 | 0.1798 |
| hsa-miR-129-5p | 0.8685 | 0.1785 |
| hsa-miR-4745-5p | 0.8752 | 0.1773 |
| hsa-miR-6809-5p | 0.8798 | 0.1825 |
| hsa-miR-365b-5p | 0.9048 | 0.0610 |
| hsa-miR-378c | 0.9064 | 0.0597 |
| hsa-miR-11400 | 0.9087 | 0.1726 |
| hsa-let-7c-5p | 0.9088 | 0.0114 |
| hsa-miR-1179 | 0.9228 | 0.0982 |
| hsa-miR-3065-5p | 0.9483 | 0.1553 |
| hsa-miR-10b-5p | 0.9619 | 0.0438 |
| hsa-miR-493-5p | 0.9737 | 0.0469 |
| hsa-miR-6866-5p | 1.0147 | 0.1086 |
| hsa-miR-378i | 1.0253 | 0.0346 |
| hsa-miR-4510 | 1.0261 | 0.0708 |
| hsa-miR-1285-5p | 1.0320 | 0.0248 |
| hsa-miR-4511 | 1.0414 | 0.1155 |
| hsa-miR-199b-5p | 1.0466 | 0.0029 |
| hsa-miR-1262 | 1.0505 | 0.1130 |
| hsa-miR-485-5p | 1.0538 | 0.0317 |
| hsa-miR-10400-5p | 1.1227 | 0.0377 |
| hsa-miR-3126-5p | 1.1248 | 0.1030 |
| hsa-miR-92a-1-5p | 1.1411 | 0.0625 |
| hsa-miR-1843 | 1.1666 | 0.0069 |
| hsa-miR-877-5p | 1.1679 | 0.0027 |
| hsa-miR-99b-5p | 1.1849 | 4.43049574142627e-05 |
| hsa-miR-4298 | 1.1873 | 0.0790 |
| hsa-miR-4508 | 1.2086 | 0.0107 |
| hsa-miR-4800-5p | 1.2657 | 0.0662 |
| hsa-miR-503-5p | 1.2774 | 0.0325 |
| hsa-miR-542-5p | 1.2896 | 0.0187 |
| hsa-miR-654-5p | 1.3120 | 0.0182 |
| hsa-miR-548b-5p | 1.3124 | 0.0610 |
| hsa-miR-589-5p | 1.3329 | 0.0006 |
| hsa-miR-548ap-5p | 1.3399 | 0.0620 |
| hsa-miR-146b-5p | 1.3546 | 0.0001 |
| hsa-miR-3622a-5p | 1.3712 | 0.0419 |
| hsa-miR-660-5p | 1.3871 | 0.0003 |
| hsa-miR-1255b-5p | 1.4109 | 0.0526 |
| hsa-miR-196a-5p | 1.4459 | 0.0046 |
| hsa-miR-3129-5p | 1.4744 | 0.0461 |
| hsa-miR-99a-5p | 1.4836 | 5.65189366185928e-07 |
| hsa-miR-486-5p | 1.4930 | 0.0156 |
| hsa-miR-1270 | 1.5496 | 0.0253 |
| hsa-miR-675-5p | 1.6037 | 0.0042 |
| hsa-miR-193b-5p | 1.6090 | 0.0026 |
| hsa-miR-6807-5p | 1.6210 | 0.0328 |
| hsa-miR-432-5p | 1.6402 | 6.42194891502486e-05 |
| hsa-miR-515-5p | 1.6987 | 0.0149 |
| hsa-miR-619-5p | 1.7160 | 0.0031 |
| hsa-miR-412-5p | 1.7470 | 0.0117 |
| hsa-miR-3150b-5p | 1.7861 | 0.0195 |
| hsa-miR-629-5p | 1.7969 | 0.0015 |
| hsa-miR-9901 | 1.8232 | 0.0055 |
| hsa-miR-197-5p | 1.8277 | 0.0205 |
| hsa-miR-10401-3p | 1.8419 | 0.0027 |
| hsa-miR-335-5p | 1.8584 | 2.17259431283305e-05 |
| hsa-miR-146a-5p | 1.9121 | 9.15883238974555e-07 |
| hsa-miR-12136 | 1.9310 | 0.0005 |
| hsa-miR-3196 | 1.9483 | 0.0012 |
| hsa-miR-3182 | 1.9996 | 0.0135 |
| hsa-miR-10a-5p | 2.0242 | 4.6656465337647e-09 |
| hsa-miR-548c-5p | 2.1132 | 0.0008 |
| hsa-miR-548o-5p | 2.1132 | 0.0008 |
| hsa-miR-6126 | 2.1788 | 0.0072 |
| hsa-miR-100-5p | 2.2192 | 8.06295148081774e-10 |
| hsa-miR-548au-5p | 2.2416 | 0.0015 |
| hsa-miR-6894-5p | 2.2586 | 0.0029 |
| hsa-miR-548am-5p | 2.2782 | 0.0006 |
| hsa-miR-96-5p | 2.3844 | NA |
| hsa-miR-27a-5p | 2.3929 | 4.26168138169312e-06 |
| hsa-miR-548e-5p | 2.3942 | 0.0003 |
| hsa-miR-222-5p | 2.4810 | 5.80611715111734e-06 |
| hsa-miR-320d | 2.5460 | 7.08510538254772e-08 |
| hsa-miR-342-5p | 2.7159 | 5.29215981975745e-11 |
| hsa-miR-6858-5p | 2.7230 | 0.0018 |
| hsa-miR-483-5p | 2.8145 | 4.7832652826935e-08 |
| hsa-miR-548h-5p | 2.8682 | 3.70257381415078e-05 |
| hsa-miR-548ar-5p | 2.9484 | 1.86988663259862e-05 |
| hsa-miR-548aj-5p | 3.0524 | 1.29005117791591e-05 |
| hsa-miR-548f-5p | 3.1225 | 8.42990389802947e-06 |
| hsa-miR-548g-5p | 3.1418 | 7.99199982468635e-06 |
| hsa-miR-548x-5p | 3.1418 | 7.99199982468635e-06 |
| hsa-miR-4787-5p | 3.2496 | 3.64578361230073e-07 |
| hsa-miR-4488 | 3.4396 | 9.04608863117046e-09 |
| hsa-miR-3605-5p | 3.7738 | 6.99696422347182e-09 |
| hsa-miR-4430 | 4.1722 | 6.89416527373073e-05 |
| hsa-miR-7704 | 4.3288 | 1.76696950421882e-10 |
| hsa-miR-4466 | 4.4682 | 4.4031376608375e-05 |
| hsa-miR-423-5p | 4.5035 | 8.89540024206751e-24 |
| hsa-miR-3178 | 4.8446 | 1.17377945068722e-06 |
| hsa-miR-3648 | 5.1766 | 3.29124718264936e-07 |
| hsa-miR-4516 | 5.4300 | 1.96938758248897e-30 |
| hsa-miR-10396b-5p | 5.9798 | 4.63361920335766e-12 |
| hsa-miR-10396a-5p | 6.1741 | 3.27932305325682e-11 |
| hsa-miR-1246 | 7.1220 | 1.30020696927748e-23 |
