# Supplementary material for: Extracellular vesicle-associated miR-515-5p from adipose tissue regulates placental metabolism and fetal growth in gestational diabetes mellitus
Source: Cardiovasc Diabetol. 2025 May 14;24:205. doi: 10.1186/s12933-025-02739-z (PMC12080180; doi:10.1186/s12933-025-02739-z)
Supplement: Supplementary file 5 — Supplementary Material 5 [file 12933_2025_2739_MOESM5_ESM.docx]

**Supplementary Table 4: Table below shows the miRNA identified between adipose tissue and EVs from GDM.**

| miRNA | log2FoldChange | pvalue |
| --- | --- | --- |
| hsa-miR-146a-5p | -2.63016 | 0.00020 |
| hsa-miR-378i | -2.28290 | 0.00082 |
| hsa-miR-378c | -2.14000 | 0.00164 |
| hsa-miR-10b-5p | -2.03851 | 0.00030 |
| hsa-miR-335-5p | -1.92210 | 0.00088 |
| hsa-miR-10a-5p | -1.72207 | 1.62148944116654e-05 |
| hsa-miR-100-5p | -1.61339 | 5.60302309038731e-05 |
| hsa-miR-486-5p | -1.25150 | 0.03273 |
| hsa-miR-12136 | -1.18742 | 0.04794 |
| hsa-miR-15a-5p | -1.15455 | 0.02976 |
| hsa-miR-186-5p | -1.03845 | 0.00714 |
| hsa-miR-16-5p | -1.03645 | 0.01350 |
| hsa-miR-99a-5p | -0.95448 | 0.00954 |
| hsa-miR-181b-5p | -0.92672 | 0.02428 |
| hsa-miR-99b-5p | -0.90409 | 0.00969 |
| hsa-miR-146b-5p | -0.90023 | 0.00898 |
| hsa-miR-98-5p | -0.87835 | 0.04936 |
| hsa-miR-192-5p | -0.87100 | 0.05791 |
| hsa-miR-532-5p | -0.86404 | 0.06471 |
| hsa-miR-660-5p | -0.85389 | 0.08608 |
| hsa-miR-589-5p | -0.73553 | 0.18651 |
| hsa-miR-452-5p | -0.72566 | 0.16031 |
| hsa-miR-378d | -0.68563 | 0.21301 |
| hsa-miR-424-5p | -0.67089 | 0.18069 |
| hsa-miR-339-5p | -0.63076 | 0.24378 |
| hsa-miR-93-5p | -0.62007 | 0.16945 |
| hsa-miR-34a-5p | -0.57597 | 0.26345 |
| hsa-miR-181d-5p | -0.56190 | 0.28491 |
| hsa-miR-769-5p | -0.55784 | 0.18564 |
| hsa-miR-181a-5p | -0.50802 | 0.06948 |
| hsa-miR-493-5p | -0.48755 | 0.34339 |
| hsa-miR-574-5p | -0.46283 | 0.35569 |
| hsa-miR-423-5p | -0.44877 | 0.22775 |
| hsa-miR-224-5p | -0.44454 | 0.35302 |
| hsa-let-7b-5p | -0.43157 | 0.06667 |
| hsa-miR-199b-5p | -0.39992 | 0.23548 |
| hsa-miR-361-5p | -0.38448 | 0.38108 |
| hsa-miR-432-5p | -0.38120 | 0.43353 |
| hsa-miR-15b-5p | -0.35543 | 0.46366 |
| hsa-miR-148a-5p | -0.35061 | 0.42076 |
| hsa-miR-136-5p | -0.33245 | 0.48869 |
| hsa-miR-31-5p | -0.32298 | 0.51091 |
| hsa-miR-181c-5p | -0.31682 | 0.38532 |
| hsa-miR-1246 | -0.27030 | 0.55468 |
| hsa-miR-4488 | -0.25650 | 0.59576 |
| hsa-miR-34c-5p | -0.22338 | 0.64178 |
| hsa-miR-374b-5p | -0.22319 | 0.62853 |
| hsa-miR-139-5p | -0.21226 | 0.63950 |
| hsa-let-7i-5p | -0.17701 | 0.54334 |
| hsa-miR-106b-5p | -0.17569 | 0.70888 |
| hsa-miR-584-5p | -0.16814 | 0.71734 |
| hsa-miR-214-5p | -0.15966 | 0.71668 |
| hsa-let-7d-5p | -0.15787 | 0.64484 |
| hsa-miR-548h-5p | -0.15700 | 0.73991 |
| hsa-let-7g-5p | -0.15282 | 0.53994 |
| hsa-miR-548aj-5p | -0.14847 | 0.75317 |
| hsa-miR-548k | -0.14826 | 0.75377 |
| hsa-miR-4286 | -0.14699 | 0.75582 |
| hsa-miR-548f-5p | -0.14386 | 0.76040 |
| hsa-let-7c-5p | -0.12541 | 0.70086 |
| hsa-miR-629-5p | -0.11297 | 0.80953 |
| hsa-miR-548ar-5p | -0.11029 | 0.81400 |
| hsa-miR-185-5p | -0.09422 | 0.83920 |
| hsa-miR-191-5p | -0.09159 | 0.68788 |
| hsa-miR-199a-5p | -0.08791 | 0.78410 |
| hsa-miR-196b-5p | -0.08380 | 0.85281 |
| hsa-miR-320d | -0.08242 | 0.85952 |
| hsa-miR-194-5p | -0.07865 | 0.86642 |
| hsa-miR-154-5p | -0.07830 | 0.86684 |
| hsa-miR-193b-5p | -0.06220 | 0.89419 |
| hsa-miR-411-5p | -0.05819 | 0.88056 |
| hsa-miR-455-5p | -0.05654 | 0.89737 |
| hsa-miR-20a-5p | -0.05224 | 0.91048 |
| hsa-miR-3605-5p | -0.04889 | 0.91621 |
| hsa-miR-30a-5p | -0.03976 | 0.85411 |
| hsa-miR-193a-5p | -0.03806 | 0.93491 |
| hsa-miR-1271-5p | -0.03549 | 0.93887 |
| hsa-miR-196a-5p | -0.02741 | 0.95320 |
| hsa-let-7f-5p | -0.02099 | 0.92889 |
| hsa-miR-374a-5p | -0.01980 | 0.96400 |
| hsa-miR-148b-5p | -0.01600 | 0.97214 |
| hsa-miR-218-5p | -0.01199 | 0.97768 |
| hsa-miR-195-5p | -0.00722 | 0.98063 |
| hsa-miR-33b-5p | -0.00635 | 0.98890 |
| hsa-miR-183-5p | -0.00562 | 0.99043 |
| hsa-miR-340-5p | -0.00436 | 0.99128 |
| hsa-miR-126-5p | 0.00793 | 0.96590 |
| hsa-miR-1179 | 0.00814 | 0.98569 |
| hsa-miR-151a-5p | 0.01423 | 0.96215 |
| hsa-miR-561-5p | 0.02117 | 0.96295 |
| hsa-miR-654-5p | 0.02832 | 0.95018 |
| hsa-miR-27b-5p | 0.02989 | 0.94862 |
| hsa-miR-548e-5p | 0.03850 | 0.93185 |
| hsa-miR-548c-5p | 0.04285 | 0.92419 |
| hsa-miR-548o-5p | 0.04285 | 0.92419 |
| hsa-miR-548g-5p | 0.04374 | 0.92541 |
| hsa-miR-548x-5p | 0.04374 | 0.92541 |
| hsa-miR-548am-5p | 0.04668 | 0.91710 |
| hsa-miR-152-5p | 0.05041 | 0.91065 |
| hsa-miR-4516 | 0.05076 | 0.89780 |
| hsa-miR-542-5p | 0.06336 | 0.88784 |
| hsa-miR-382-5p | 0.06561 | 0.88259 |
| hsa-miR-143-5p | 0.06656 | 0.88199 |
| hsa-miR-22-5p | 0.07168 | 0.87871 |
| hsa-miR-675-5p | 0.07258 | 0.87077 |
| hsa-miR-26b-5p | 0.07405 | 0.76922 |
| hsa-miR-145-5p | 0.07548 | 0.84422 |
| hsa-miR-127-5p | 0.07832 | 0.86632 |
| hsa-miR-28-5p | 0.07955 | 0.83648 |
| hsa-miR-10399-3p | 0.08109 | 0.85521 |
| hsa-miR-1296-5p | 0.08322 | 0.85926 |
| hsa-miR-222-5p | 0.08494 | 0.85632 |
| hsa-miR-651-5p | 0.08685 | 0.84435 |
| hsa-miR-324-5p | 0.08935 | 0.83973 |
| hsa-miR-190a-5p | 0.09125 | 0.83638 |
| hsa-miR-873-5p | 0.09590 | 0.82824 |
| hsa-miR-144-5p | 0.09611 | 0.82803 |
| hsa-miR-17-5p | 0.09831 | 0.82700 |
| hsa-miR-5683 | 0.10085 | 0.83010 |
| hsa-miR-409-5p | 0.10248 | 0.81673 |
| hsa-miR-378g | 0.10295 | 0.81592 |
| hsa-miR-628-5p | 0.10739 | 0.80821 |
| hsa-miR-548au-5p | 0.10985 | 0.80393 |
| hsa-miR-1287-5p | 0.11006 | 0.80356 |
| hsa-miR-155-5p | 0.11420 | 0.76799 |
| hsa-miR-25-5p | 0.11650 | 0.79245 |
| hsa-miR-653-5p | 0.11651 | 0.79243 |
| hsa-miR-223-5p | 0.12288 | 0.78148 |
| hsa-miR-4510 | 0.12715 | 0.77417 |
| hsa-miR-548d-5p | 0.13115 | 0.76737 |
| hsa-miR-29c-5p | 0.13117 | 0.76734 |
| hsa-miR-503-5p | 0.13284 | 0.76450 |
| hsa-miR-204-5p | 0.13611 | 0.74859 |
| hsa-miR-582-5p | 0.13922 | 0.75371 |
| hsa-miR-3613-5p | 0.14008 | 0.75225 |
| hsa-miR-6894-5p | 0.14422 | 0.74529 |
| hsa-miR-26a-5p | 0.14425 | 0.57333 |
| hsa-miR-378a-5p | 0.14939 | 0.73663 |
| hsa-miR-299-3p | 0.15053 | 0.75043 |
| hsa-let-7a-5p | 0.15837 | 0.56979 |
| hsa-miR-422a | 0.16388 | 0.71265 |
| hsa-miR-6858-5p | 0.16647 | 0.70840 |
| hsa-miR-134-5p | 0.17278 | 0.71219 |
| hsa-miR-6126 | 0.17653 | 0.69203 |
| hsa-miR-92a-1-5p | 0.18057 | 0.68551 |
| hsa-miR-454-5p | 0.18137 | 0.68423 |
| hsa-miR-101-5p | 0.18171 | 0.70072 |
| hsa-miR-331-5p | 0.18205 | 0.68314 |
| hsa-miR-2110 | 0.18634 | 0.67627 |
| hsa-miR-497-5p | 0.19003 | 0.60146 |
| hsa-miR-188-5p | 0.19294 | 0.66577 |
| hsa-miR-1291 | 0.19753 | 0.65853 |
| hsa-miR-425-5p | 0.19801 | 0.64845 |
| hsa-miR-190b-5p | 0.19895 | 0.65631 |
| hsa-miR-379-5p | 0.20239 | 0.65091 |
| hsa-miR-3913-5p | 0.20253 | 0.65070 |
| hsa-miR-140-5p | 0.20352 | 0.67020 |
| hsa-miR-212-5p | 0.20460 | 0.64747 |
| hsa-miR-412-5p | 0.20489 | 0.64703 |
| hsa-miR-5585-3p | 0.20727 | 0.64332 |
| hsa-miR-338-5p | 0.20840 | 0.64157 |
| hsa-miR-1270 | 0.20884 | 0.64089 |
| hsa-miR-4508 | 0.20951 | 0.65852 |
| hsa-miR-483-5p | 0.20963 | 0.64335 |
| hsa-miR-32-5p | 0.21273 | 0.65507 |
| hsa-miR-1262 | 0.21395 | 0.63301 |
| hsa-miR-342-5p | 0.21666 | 0.61393 |
| hsa-miR-330-5p | 0.22089 | 0.62241 |
| hsa-miR-369-5p | 0.22189 | 0.64287 |
| hsa-miR-9-5p | 0.22371 | 0.61813 |
| hsa-miR-548ay-5p | 0.22615 | 0.61444 |
| hsa-miR-20b-5p | 0.22758 | 0.61228 |
| hsa-miR-504-5p | 0.23129 | 0.60672 |
| hsa-miR-377-5p | 0.23178 | 0.60598 |
| hsa-miR-10399-5p | 0.23716 | 0.59798 |
| hsa-miR-664a-5p | 0.23959 | 0.61731 |
| hsa-miR-296-5p | 0.24129 | 0.59188 |
| hsa-miR-874-5p | 0.24502 | 0.58640 |
| hsa-miR-3622a-5p | 0.25010 | 0.57901 |
| hsa-miR-6866-5p | 0.25010 | 0.57901 |
| hsa-miR-2467-5p | 0.25406 | 0.57327 |
| hsa-miR-2114-5p | 0.25878 | 0.56650 |
| hsa-miR-182-5p | 0.25879 | 0.54103 |
| hsa-miR-548ad-5p | 0.26044 | 0.56414 |
| hsa-miR-548ae-5p | 0.26044 | 0.56414 |
| hsa-miR-10401-3p | 0.26077 | 0.59010 |
| hsa-miR-1468-5p | 0.26301 | 0.58646 |
| hsa-miR-450a-5p | 0.26633 | 0.57936 |
| hsa-miR-548b-5p | 0.26712 | 0.55466 |
| hsa-miR-4497 | 0.26785 | 0.55364 |
| hsa-miR-92b-5p | 0.27126 | 0.54886 |
| hsa-miR-378b | 0.27874 | 0.53847 |
| hsa-miR-7977 | 0.27998 | 0.55016 |
| hsa-miR-431-5p | 0.29132 | 0.54988 |
| hsa-miR-548ba | 0.29538 | 0.51585 |
| hsa-miR-7704 | 0.30166 | 0.47914 |
| hsa-miR-10527-5p | 0.30354 | 0.50503 |
| hsa-miR-3182 | 0.30479 | 0.50338 |
| hsa-miR-1843 | 0.30713 | 0.51648 |
| hsa-miR-142-5p | 0.30944 | 0.41019 |
| hsa-miR-502-5p | 0.31404 | 0.49134 |
| hsa-miR-345-5p | 0.31935 | 0.48355 |
| hsa-miR-130a-5p | 0.31979 | 0.48398 |
| hsa-miR-511-5p | 0.32068 | 0.48283 |
| hsa-miR-1255b-5p | 0.32336 | 0.47944 |
| hsa-miR-891a-5p | 0.32942 | 0.47181 |
| hsa-miR-450b-5p | 0.32968 | 0.47986 |
| hsa-miR-383-5p | 0.33451 | 0.46548 |
| hsa-miR-1277-5p | 0.34017 | 0.45852 |
| hsa-miR-548w | 0.34017 | 0.45852 |
| hsa-miR-6511b-5p | 0.34090 | 0.45762 |
| hsa-miR-548ap-5p | 0.34570 | 0.45179 |
| hsa-miR-95-5p | 0.34590 | 0.45154 |
| hsa-miR-641 | 0.34644 | 0.45089 |
| hsa-miR-6735-5p | 0.34718 | 0.45000 |
| hsa-miR-3065-5p | 0.35236 | 0.44379 |
| hsa-miR-6807-5p | 0.35236 | 0.44379 |
| hsa-miR-744-5p | 0.35277 | 0.41126 |
| hsa-miR-7976 | 0.35836 | 0.43669 |
| hsa-miR-2355-5p | 0.35838 | 0.43667 |
| hsa-miR-548i | 0.35914 | 0.43577 |
| hsa-miR-365b-5p | 0.35946 | 0.46744 |
| hsa-miR-221-5p | 0.36502 | 0.43293 |
| hsa-miR-5010-5p | 0.36538 | 0.42849 |
| hsa-miR-548aq-3p | 0.37117 | 0.42182 |
| hsa-miR-6809-5p | 0.37117 | 0.42182 |
| hsa-miR-197-5p | 0.37195 | 0.42093 |
| hsa-miR-3129-5p | 0.37195 | 0.42093 |
| hsa-miR-4800-5p | 0.37219 | 0.42065 |
| hsa-miR-34b-5p | 0.37272 | 0.42005 |
| hsa-miR-627-5p | 0.37775 | 0.41434 |
| hsa-miR-200a-5p | 0.37852 | 0.41347 |
| hsa-miR-3934-5p | 0.37891 | 0.41304 |
| hsa-miR-501-5p | 0.37969 | 0.41216 |
| hsa-miR-548ag | 0.37969 | 0.41216 |
| hsa-miR-877-5p | 0.38003 | 0.42196 |
| hsa-miR-942-5p | 0.38574 | 0.40542 |
| hsa-miR-1273h-5p | 0.38630 | 0.40479 |
| hsa-miR-33a-5p | 0.38630 | 0.40479 |
| hsa-miR-4298 | 0.38630 | 0.40479 |
| hsa-miR-4667-5p | 0.38630 | 0.40479 |
| hsa-miR-299-5p | 0.38652 | 0.40455 |
| hsa-miR-616-5p | 0.38652 | 0.40455 |
| hsa-miR-11400 | 0.38710 | 0.40391 |
| hsa-miR-3199 | 0.39341 | 0.39698 |
| hsa-miR-548j-5p | 0.39341 | 0.39698 |
| hsa-miR-370-5p | 0.40141 | 0.38835 |
| hsa-miR-499a-5p | 0.40141 | 0.38835 |
| hsa-miR-365a-5p | 0.40243 | 0.38726 |
| hsa-miR-550a-3-5p | 0.40243 | 0.38726 |
| hsa-miR-550a-5p | 0.40243 | 0.38726 |
| hsa-miR-676-5p | 0.40323 | 0.38641 |
| hsa-miR-29a-5p | 0.40996 | 0.37930 |
| hsa-miR-130b-5p | 0.41074 | 0.37849 |
| hsa-miR-371b-5p | 0.41129 | 0.37790 |
| hsa-miR-3126-5p | 0.41996 | 0.36894 |
| hsa-miR-6501-5p | 0.41996 | 0.36894 |
| hsa-miR-138-5p | 0.42714 | 0.36165 |
| hsa-miR-548at-5p | 0.42714 | 0.36165 |
| hsa-miR-663a | 0.42714 | 0.36165 |
| hsa-miR-129-5p | 0.42999 | 0.35879 |
| hsa-miR-3180 | 0.42999 | 0.35879 |
| hsa-miR-3180-3p | 0.42999 | 0.35879 |
| hsa-miR-378h | 0.43515 | 0.35365 |
| hsa-miR-3178 | 0.43732 | 0.37913 |
| hsa-miR-23b-5p | 0.43832 | 0.35053 |
| hsa-miR-487a-5p | 0.43906 | 0.34980 |
| hsa-miR-449c-5p | 0.44979 | 0.33943 |
| hsa-miR-6780a-5p | 0.44979 | 0.33943 |
| hsa-miR-4485-3p | 0.45328 | 0.33668 |
| hsa-miR-1285-5p | 0.45721 | 0.36302 |
| hsa-miR-3202 | 0.45859 | 0.33112 |
| hsa-miR-518e-5p | 0.45859 | 0.33112 |
| hsa-miR-519b-5p | 0.45859 | 0.33112 |
| hsa-miR-519c-5p | 0.45859 | 0.33112 |
| hsa-miR-522-5p | 0.45859 | 0.33112 |
| hsa-miR-523-5p | 0.45859 | 0.33112 |
| hsa-miR-758-5p | 0.45859 | 0.33112 |
| hsa-miR-3127-5p | 0.45925 | 0.33049 |
| hsa-miR-4745-5p | 0.45925 | 0.33049 |
| hsa-miR-128-1-5p | 0.46106 | 0.36713 |
| hsa-miR-21-5p | 0.46271 | 0.13213 |
| hsa-miR-24-2-5p | 0.46479 | 0.36126 |
| hsa-miR-337-5p | 0.46735 | 0.32301 |
| hsa-miR-3195 | 0.46887 | 0.33697 |
| hsa-miR-4524a-5p | 0.47250 | 0.31834 |
| hsa-miR-5189-5p | 0.47250 | 0.31834 |
| hsa-miR-6511a-5p | 0.47250 | 0.31834 |
| hsa-miR-219a-5p | 0.48752 | 0.30500 |
| hsa-miR-3155b | 0.48752 | 0.30500 |
| hsa-miR-3170 | 0.48752 | 0.30500 |
| hsa-miR-3200-5p | 0.48752 | 0.30500 |
| hsa-miR-4732-5p | 0.48752 | 0.30500 |
| hsa-miR-4747-5p | 0.48752 | 0.30500 |
| hsa-miR-4769-5p | 0.48752 | 0.30500 |
| hsa-miR-5701 | 0.48752 | 0.30500 |
| hsa-miR-1268b | 0.49718 | 0.29669 |
| hsa-miR-211-5p | 0.49718 | 0.29669 |
| hsa-miR-24-1-5p | 0.49718 | 0.29669 |
| hsa-miR-3620-5p | 0.49718 | 0.29669 |
| hsa-miR-449b-5p | 0.49718 | 0.29669 |
| hsa-miR-519a-5p | 0.49718 | 0.29669 |
| hsa-miR-548ak | 0.49718 | 0.29669 |
| hsa-miR-548az-5p | 0.49718 | 0.29669 |
| hsa-miR-6891-5p | 0.49718 | 0.29669 |
| hsa-miR-3928-3p | 0.49759 | 0.29634 |
| hsa-miR-433-5p | 0.49759 | 0.29634 |
| hsa-miR-4429 | 0.49759 | 0.29634 |
| hsa-miR-6505-5p | 0.49759 | 0.29634 |
| hsa-miR-652-5p | 0.49759 | 0.29634 |
| hsa-miR-625-5p | 0.50450 | 0.32986 |
| hsa-miR-887-5p | 0.50493 | 0.31871 |
| hsa-miR-4485-5p | 0.50658 | 0.28878 |
| hsa-miR-4430 | 0.51342 | 0.32287 |
| hsa-miR-10396b-3p | 0.51547 | 0.28148 |
| hsa-miR-23a-5p | 0.51547 | 0.28148 |
| hsa-miR-4649-5p | 0.51547 | 0.28148 |
| hsa-miR-545-5p | 0.51547 | 0.28148 |
| hsa-miR-6779-5p | 0.51547 | 0.28148 |
| hsa-miR-6859-5p | 0.51547 | 0.28148 |
| hsa-miR-6892-5p | 0.51547 | 0.28148 |
| hsa-miR-7856-5p | 0.51547 | 0.28148 |
| hsa-miR-4466 | 0.51725 | 0.32043 |
| hsa-miR-29b-2-5p | 0.52517 | 0.27368 |
| hsa-miR-3926 | 0.52517 | 0.27368 |
| hsa-miR-4705 | 0.52517 | 0.27368 |
| hsa-miR-541-5p | 0.52517 | 0.27368 |
| hsa-miR-5588-5p | 0.52517 | 0.27368 |
| hsa-miR-6503-5p | 0.52517 | 0.27368 |
| hsa-miR-6761-5p | 0.52517 | 0.27368 |
| hsa-miR-30d-5p | 0.53343 | 0.12267 |
| hsa-miR-10226 | 0.54438 | 0.25894 |
| hsa-miR-4424 | 0.54438 | 0.25894 |
| hsa-miR-539-5p | 0.54438 | 0.25894 |
| hsa-miR-7-5p | 0.54438 | 0.25894 |
| hsa-miR-4507 | 0.55267 | 0.25285 |
| hsa-miR-5699-5p | 0.55267 | 0.25285 |
| hsa-miR-618 | 0.55267 | 0.25285 |
| hsa-miR-671-5p | 0.55267 | 0.25285 |
| hsa-miR-6724-5p | 0.55267 | 0.25285 |
| hsa-miR-10392-5p | 0.55453 | 0.25150 |
| hsa-miR-1226-5p | 0.55453 | 0.25150 |
| hsa-miR-216a-5p | 0.55453 | 0.25150 |
| hsa-miR-4743-5p | 0.55453 | 0.25150 |
| hsa-miR-4746-5p | 0.55453 | 0.25150 |
| hsa-miR-518f-5p | 0.55453 | 0.25150 |
| hsa-miR-520d-5p | 0.55453 | 0.25150 |
| hsa-miR-548q | 0.55453 | 0.25150 |
| hsa-miR-5585-5p | 0.55453 | 0.25150 |
| hsa-miR-5706 | 0.55453 | 0.25150 |
| hsa-miR-6514-5p | 0.55453 | 0.25150 |
| hsa-miR-664b-5p | 0.55453 | 0.25150 |
| hsa-miR-6740-5p | 0.55453 | 0.25150 |
| hsa-miR-6757-5p | 0.55453 | 0.25150 |
| hsa-miR-6865-5p | 0.55453 | 0.25150 |
| hsa-miR-10401-5p | 0.57475 | 0.23724 |
| hsa-miR-1276 | 0.57475 | 0.23724 |
| hsa-miR-2681-5p | 0.57475 | 0.23724 |
| hsa-miR-29b-1-5p | 0.57475 | 0.23724 |
| hsa-miR-3691-5p | 0.57475 | 0.23724 |
| hsa-miR-491-5p | 0.57475 | 0.23724 |
| hsa-miR-548ab | 0.57475 | 0.23724 |
| hsa-miR-6513-5p | 0.57475 | 0.23724 |
| hsa-miR-659-5p | 0.57475 | 0.23724 |
| hsa-miR-6755-5p | 0.57475 | 0.23724 |
| hsa-miR-6770-3p | 0.57475 | 0.23724 |
| hsa-miR-6795-5p | 0.57475 | 0.23724 |
| hsa-miR-6845-5p | 0.57475 | 0.23724 |
| hsa-miR-8061 | 0.57475 | 0.23724 |
| hsa-miR-876-5p | 0.57475 | 0.23724 |
| hsa-let-7e-5p | 0.57640 | 0.13365 |
| hsa-miR-576-5p | 0.57783 | 0.25008 |
| hsa-miR-3074-5p | 0.58819 | 0.24581 |
| hsa-miR-3196 | 0.58871 | 0.25950 |
| hsa-miR-27a-5p | 0.59635 | 0.20538 |
| hsa-miR-1268a | 0.60970 | 0.21442 |
| hsa-miR-18b-5p | 0.60970 | 0.21442 |
| hsa-miR-2116-5p | 0.60970 | 0.21442 |
| hsa-miR-3661 | 0.60970 | 0.21442 |
| hsa-miR-3677-3p | 0.60970 | 0.21442 |
| hsa-miR-3918 | 0.60970 | 0.21442 |
| hsa-miR-4284 | 0.60970 | 0.21442 |
| hsa-miR-4658 | 0.60970 | 0.21442 |
| hsa-miR-4668-5p | 0.60970 | 0.21442 |
| hsa-miR-5002-5p | 0.60970 | 0.21442 |
| hsa-miR-513c-5p | 0.60970 | 0.21442 |
| hsa-miR-525-5p | 0.60970 | 0.21442 |
| hsa-miR-548n | 0.60970 | 0.21442 |
| hsa-miR-585-5p | 0.60970 | 0.21442 |
| hsa-miR-6509-5p | 0.60970 | 0.21442 |
| hsa-miR-6732-5p | 0.60970 | 0.21442 |
| hsa-miR-6852-5p | 0.60970 | 0.21442 |
| hsa-miR-6746-5p | 0.61382 | 0.22694 |
| hsa-miR-3648 | 0.63032 | 0.23111 |
| hsa-miR-132-5p | 0.65068 | 0.22972 |
| hsa-miR-1281 | 0.67229 | 0.17924 |
| hsa-miR-1298-5p | 0.67229 | 0.17924 |
| hsa-miR-200b-5p | 0.67229 | 0.17924 |
| hsa-miR-3145-5p | 0.67229 | 0.17924 |
| hsa-miR-3152-5p | 0.67229 | 0.17924 |
| hsa-miR-4288 | 0.67229 | 0.17924 |
| hsa-miR-4435 | 0.67229 | 0.17924 |
| hsa-miR-4670-5p | 0.67229 | 0.17924 |
| hsa-miR-4700-5p | 0.67229 | 0.17924 |
| hsa-miR-4763-5p | 0.67229 | 0.17924 |
| hsa-miR-488-5p | 0.67229 | 0.17924 |
| hsa-miR-5089-5p | 0.67229 | 0.17924 |
| hsa-miR-548aw | 0.67229 | 0.17924 |
| hsa-miR-548ax | 0.67229 | 0.17924 |
| hsa-miR-580-5p | 0.67229 | 0.17924 |
| hsa-miR-6730-5p | 0.67229 | 0.17924 |
| hsa-miR-6821-5p | 0.67229 | 0.17924 |
| hsa-miR-6838-5p | 0.67229 | 0.17924 |
| hsa-miR-6880-5p | 0.67229 | 0.17924 |
| hsa-miR-8071 | 0.67229 | 0.17924 |
| hsa-miR-10396a-3p | 0.67229 | 0.17924 |
| hsa-miR-11401 | 0.67229 | 0.17924 |
| hsa-miR-1229-5p | 0.67229 | 0.17924 |
| hsa-miR-1245b-5p | 0.67229 | 0.17924 |
| hsa-miR-1261 | 0.67229 | 0.17924 |
| hsa-miR-1273c | 0.67229 | 0.17924 |
| hsa-miR-1304-5p | 0.67229 | 0.17924 |
| hsa-miR-135b-5p | 0.67229 | 0.17924 |
| hsa-miR-3125 | 0.67229 | 0.17924 |
| hsa-miR-3128 | 0.67229 | 0.17924 |
| hsa-miR-3133 | 0.67229 | 0.17924 |
| hsa-miR-3161 | 0.67229 | 0.17924 |
| hsa-miR-329-5p | 0.67229 | 0.17924 |
| hsa-miR-3616-5p | 0.67229 | 0.17924 |
| hsa-miR-3665 | 0.67229 | 0.17924 |
| hsa-miR-3679-5p | 0.67229 | 0.17924 |
| hsa-miR-3916 | 0.67229 | 0.17924 |
| hsa-miR-4487 | 0.67229 | 0.17924 |
| hsa-miR-449a | 0.67229 | 0.17924 |
| hsa-miR-4638-5p | 0.67229 | 0.17924 |
| hsa-miR-4646-5p | 0.67229 | 0.17924 |
| hsa-miR-4647 | 0.67229 | 0.17924 |
| hsa-miR-4709-5p | 0.67229 | 0.17924 |
| hsa-miR-4711-5p | 0.67229 | 0.17924 |
| hsa-miR-4726-5p | 0.67229 | 0.17924 |
| hsa-miR-4739 | 0.67229 | 0.17924 |
| hsa-miR-4740-5p | 0.67229 | 0.17924 |
| hsa-miR-4766-5p | 0.67229 | 0.17924 |
| hsa-miR-4770 | 0.67229 | 0.17924 |
| hsa-miR-4786-5p | 0.67229 | 0.17924 |
| hsa-miR-5094 | 0.67229 | 0.17924 |
| hsa-miR-548an | 0.67229 | 0.17924 |
| hsa-miR-5581-5p | 0.67229 | 0.17924 |
| hsa-miR-577 | 0.67229 | 0.17924 |
| hsa-miR-590-5p | 0.67229 | 0.17924 |
| hsa-miR-6770-5p | 0.67229 | 0.17924 |
| hsa-miR-6855-5p | 0.67229 | 0.17924 |
| hsa-miR-6861-3p | 0.67229 | 0.17924 |
| hsa-miR-6861-5p | 0.67229 | 0.17924 |
| hsa-miR-6879-5p | 0.67229 | 0.17924 |
| hsa-miR-7160-5p | 0.67229 | 0.17924 |
| hsa-miR-619-5p | 0.67520 | 0.16320 |
| hsa-miR-10394-5p | 0.68927 | 0.17072 |
| hsa-miR-10526-3p | 0.68927 | 0.17072 |
| hsa-miR-11399 | 0.68927 | 0.17072 |
| hsa-miR-1185-5p | 0.68927 | 0.17072 |
| hsa-miR-1224-5p | 0.68927 | 0.17072 |
| hsa-miR-1228-5p | 0.68927 | 0.17072 |
| hsa-miR-1255a | 0.68927 | 0.17072 |
| hsa-miR-1306-5p | 0.68927 | 0.17072 |
| hsa-miR-1587 | 0.68927 | 0.17072 |
| hsa-miR-217-5p | 0.68927 | 0.17072 |
| hsa-miR-2278 | 0.68927 | 0.17072 |
| hsa-miR-3132 | 0.68927 | 0.17072 |
| hsa-miR-3135b | 0.68927 | 0.17072 |
| hsa-miR-3163 | 0.68927 | 0.17072 |
| hsa-miR-346 | 0.68927 | 0.17072 |
| hsa-miR-3612 | 0.68927 | 0.17072 |
| hsa-miR-3619-5p | 0.68927 | 0.17072 |
| hsa-miR-371a-5p | 0.68927 | 0.17072 |
| hsa-miR-3940-5p | 0.68927 | 0.17072 |
| hsa-miR-4309 | 0.68927 | 0.17072 |
| hsa-miR-4433b-5p | 0.68927 | 0.17072 |
| hsa-miR-4467 | 0.68927 | 0.17072 |
| hsa-miR-4478 | 0.68927 | 0.17072 |
| hsa-miR-4523 | 0.68927 | 0.17072 |
| hsa-miR-4638-3p | 0.68927 | 0.17072 |
| hsa-miR-4687-5p | 0.68927 | 0.17072 |
| hsa-miR-4776-5p | 0.68927 | 0.17072 |
| hsa-miR-4782-5p | 0.68927 | 0.17072 |
| hsa-miR-495-5p | 0.68927 | 0.17072 |
| hsa-miR-5001-5p | 0.68927 | 0.17072 |
| hsa-miR-505-5p | 0.68927 | 0.17072 |
| hsa-miR-508-5p | 0.68927 | 0.17072 |
| hsa-miR-509-3-5p | 0.68927 | 0.17072 |
| hsa-miR-512-5p | 0.68927 | 0.17072 |
| hsa-miR-518d-5p | 0.68927 | 0.17072 |
| hsa-miR-520c-5p | 0.68927 | 0.17072 |
| hsa-miR-526a-5p | 0.68927 | 0.17072 |
| hsa-miR-548ai | 0.68927 | 0.17072 |
| hsa-miR-548av-5p | 0.68927 | 0.17072 |
| hsa-miR-548l | 0.68927 | 0.17072 |
| hsa-miR-551b-5p | 0.68927 | 0.17072 |
| hsa-miR-570-5p | 0.68927 | 0.17072 |
| hsa-miR-5703 | 0.68927 | 0.17072 |
| hsa-miR-597-5p | 0.68927 | 0.17072 |
| hsa-miR-6132 | 0.68927 | 0.17072 |
| hsa-miR-6507-5p | 0.68927 | 0.17072 |
| hsa-miR-6510-5p | 0.68927 | 0.17072 |
| hsa-miR-6512-5p | 0.68927 | 0.17072 |
| hsa-miR-6729-5p | 0.68927 | 0.17072 |
| hsa-miR-6754-5p | 0.68927 | 0.17072 |
| hsa-miR-6756-5p | 0.68927 | 0.17072 |
| hsa-miR-6834-5p | 0.68927 | 0.17072 |
| hsa-miR-6875-5p | 0.68927 | 0.17072 |
| hsa-miR-6876-5p | 0.68927 | 0.17072 |
| hsa-miR-6888-5p | 0.68927 | 0.17072 |
| hsa-miR-7152-5p | 0.68927 | 0.17072 |
| hsa-miR-766-5p | 0.68927 | 0.17072 |
| hsa-miR-7705 | 0.68927 | 0.17072 |
| hsa-miR-7973 | 0.68927 | 0.17072 |
| hsa-miR-939-5p | 0.68927 | 0.17072 |
| hsa-miR-9902 | 0.68927 | 0.17072 |
| hsa-miR-2277-5p | 0.69949 | 0.19548 |
| hsa-miR-4787-5p | 0.71043 | 0.17755 |
| hsa-miR-149-5p | 0.72965 | 0.16473 |
| hsa-miR-30b-5p | 0.73152 | 0.07089 |
| hsa-miR-18a-5p | 0.73248 | 0.18844 |
| hsa-miR-484 | 0.74036 | 0.13486 |
| hsa-miR-362-5p | 0.74488 | 0.18655 |
| hsa-miR-30e-5p | 0.74522 | 0.04759 |
| hsa-miR-376a-5p | 0.75727 | 0.18117 |
| hsa-miR-6502-5p | 0.80857 | 0.12413 |
| hsa-miR-6773-5p | 0.80857 | 0.12413 |
| hsa-miR-215-5p | 0.81420 | 0.11911 |
| hsa-miR-4662a-5p | 0.82714 | 0.14009 |
| hsa-miR-770-5p | 0.82760 | 0.12811 |
| hsa-miR-1910-5p | 0.90265 | 0.09574 |
| hsa-miR-1323 | 0.90847 | 0.12712 |
| hsa-miR-4775 | 0.92360 | 0.12418 |
| hsa-miR-1260a | 0.92508 | 0.06836 |
| hsa-miR-1275 | 0.93874 | 0.10864 |
| hsa-miR-125b-5p | 0.94894 | 0.04909 |
| hsa-miR-3614-5p | 0.95603 | 0.10341 |
| hsa-miR-1260b | 0.99699 | 0.05015 |
| hsa-miR-1250-5p | 1.01749 | 0.07016 |
| hsa-miR-3160-5p | 1.01749 | 0.07016 |
| hsa-miR-3168 | 1.01749 | 0.07016 |
| hsa-miR-3668 | 1.01749 | 0.07016 |
| hsa-miR-4436b-5p | 1.01749 | 0.07016 |
| hsa-miR-556-5p | 1.01749 | 0.07016 |
| hsa-miR-6767-5p | 1.01749 | 0.07016 |
| hsa-miR-125a-5p | 1.09870 | 0.03671 |
| hsa-miR-10400-5p | 1.12434 | 0.06381 |
| hsa-miR-4718 | 1.16514 | 0.04877 |
| hsa-miR-708-5p | 1.17527 | 0.04853 |
| hsa-miR-30c-5p | 1.21995 | 0.01148 |
| hsa-miR-4296 | 1.25876 | 0.04151 |
| hsa-miR-4539 | 1.25876 | 0.04151 |
| hsa-miR-519d-5p | 1.25876 | 0.04151 |
| hsa-miR-519e-5p | 1.25876 | 0.04151 |
| hsa-miR-6787-5p | 1.25876 | 0.04151 |
| hsa-miR-6869-5p | 1.25876 | 0.04151 |
| hsa-miR-4511 | 1.30412 | 0.05539 |
| hsa-miR-5689 | 1.37790 | 0.04347 |
| hsa-miR-519a-2-5p | 1.54203 | 0.03588 |
| hsa-miR-520b-5p | 1.54203 | 0.03588 |
| hsa-miR-4289 | 1.62370 | 0.02676 |
| hsa-miR-527 | 1.62370 | 0.02676 |
| hsa-miR-1469 | 1.66563 | 0.02892 |
| hsa-miR-106a-5p | 1.73568 | 0.02522 |
| hsa-miR-3150b-5p | 2.04912 | 0.01548 |
| hsa-miR-485-5p | 2.13132 | 0.00547 |
| hsa-miR-516a-5p | 2.17038 | 0.00615 |
| hsa-miR-518a-5p | 2.66357 | 0.00544 |
| hsa-miR-10396a-5p | 2.79537 | 9.23433682826843e-05 |
| hsa-miR-10396b-5p | 2.79537 | 9.23433682826843e-05 |
| hsa-miR-520a-5p | 3.13089 | 0.00268 |
| hsa-miR-9985 | 3.61506 | 1.66514824202134e-09 |
| hsa-miR-4454 | 3.92036 | 0.00027 |
| hsa-miR-150-5p | 4.22691 | 4.21097665416653e-10 |
| hsa-miR-1283 | 4.50894 | 0.00039 |
| hsa-miR-205-5p | 4.52057 | 0.00023 |
| hsa-miR-515-5p | 5.48678 | 6.66321397032352e-06 |
| hsa-miR-9901 | 5.56149 | 3.88783237117153e-09 |
| hsa-miR-1293 | 6.11962 | 1.42516322703381e-05 |
| hsa-miR-516b-5p | 6.50858 | 2.72713381944166e-07 |
