# Supplementary material for: Extracellular vesicle-associated miR-515-5p from adipose tissue regulates placental metabolism and fetal growth in gestational diabetes mellitus
Source: Cardiovasc Diabetol. 2025 May 14;24:205. doi: 10.1186/s12933-025-02739-z (PMC12080180; doi:10.1186/s12933-025-02739-z)
Supplement: Supplementary file 6 — Supplementary Material 6 [file 12933_2025_2739_MOESM6_ESM.docx]

**Supplementary table 5: Table below shows enriched gene ontology biological processes (GO-BP) associated with the miRNAs identified in adipose tissue EVs using vissE**

| **Pathway** | **p-value** | **Log (fold change)** |
| --- | --- | --- |
| Interferon γ response | 2.61E-06 | 0.62725674 |
| KRAS signalling DN | 0.000205 | 0.518848078 |
| Interferon α resposne | 0.000959 | 0.477270815 |
| UV response UP | 0.067797 | 0.271288555 |
| Mitotic spindle | 0.134021 | 0.157402899 |
| Oxidative phosphorylation | 0.149184 | 0.175204048 |
| HEME metabolism | 0.151515 | 0.173747839 |
| Peroxisome | 0.193396 | 0.153158809 |
| Unfolded Protein response | 0.201439 | 0.151148761 |
| Allograft rejection | 0.218213 | 0.119887845 |
| TNFα signalling via NFKβ | 0.219955 | 0.139599673 |
| Complement | 0.257908 | 0.132846301 |
| Apical surface | 0.285714 | 0.114266502 |
| Epithelial mesenchymal transition | 0.308219 | 0.097544922 |
| Permatignesis | 0.321721 | 0.105920293 |
| Hypoxia | 0.332547 | 0.112843356 |
| Protein secretion | 0.334135 | 0.113787262 |
| Androgen response | 0.403263 | 0.099927697 |
| UV response DN | 0.413357 | 0.083836106 |
| Cholesterol Homeostasis | 0.482569 | 0.076476705 |
| TGFβ signalling | 0.49711 | 0.077476755 |
| DNA repair | 0.502618 | 0.071827629 |
| Myogenesis | 0.506195 | 0.072179797 |
| IL2/STAT5 signalling | 0.528395 | 0.087569712 |
| G2M checkpoint | 0.544674 | 0.067051258 |
| Estrogen responses late | 0.549915 | 0.065831196 |
| Reactive oxygen species checkpoint | 0.555556 | 0.080202344 |
| Bile acid metabolism | 0.567686 | 0.077073673 |
| Glycolysis | 0.634021 | 0.059731798 |
| PI3K/AKT/MTOR signalling | 0.650624 | 0.060248409 |
| Inflammatory response | 0.673469 | 0.070281284 |
| Fatty acid metabolism | 0.680203 | 0.055710423 |
| MYC targets V2 | 0.707589 | 0.067051258 |
| E2F targets | 0.72028 | 0.054568065 |
| Estrogen responses early | 0.766488 | 0.052589895 |
| Pancreas β-cells | 0.814004 | 0.059348768 |
| Angiogenesis | 0.828157 | 0.055942859 |
| P53 pathway | 0.83293 | 0.063079041 |
| MYC targets V1 | 0.84743 | 0.04468544 |
| KRAS signalling UP | 0.87965 | 0.055710423 |
| Apical junction | 0.883562 | 0.044240742 |
| Xenobiotic metabolism | 0.895189 | 0.043800203 |
| Coagulation | 0.898778 | 0.044329345 |
| Hedgehog signalling | 0.908297 | 0.054120057 |
| Apoptosis | 0.924399 | 0.042331839 |
| Adipogenesis | 0.928328 | 0.041823869 |
| IL6/JAK/STAT3 sigballing | 0.930147 | 0.045134422 |
| NOTCH signalling | 0.95183 | 0.046230252 |
| MTORC1 signalling | 0.976744 | 0.05367696 |
