# Supplementary material for: Extracellular vesicle-associated miR-515-5p from adipose tissue regulates placental metabolism and fetal growth in gestational diabetes mellitus
Source: Cardiovasc Diabetol. 2025 May 14;24:205. doi: 10.1186/s12933-025-02739-z (PMC12080180; doi:10.1186/s12933-025-02739-z)
Supplement: Supplementary file 7 — Supplementary Material 7 [file 12933_2025_2739_MOESM7_ESM.docx]

**Supplementary Table 6: Table below shows the comparative analysis of enriched gene ontology biological processes (GO-BP) in GDM AT derived EVs treated placental cells generated using vissE**

| **Protein ID** | **Mapped Gene** | **p-value** | **Log (Fold Change)** |
| --- | --- | --- | --- |
| Q9Y230 | RUVB2_HUMAN | 0.00128 | -0.446000119 |
| P04080 | CYTB_HUMAN | 0.00165 | -0.547471445 |
| P67809 | YBOX1_HUMAN | 0.00204 | -0.40063747 |
| P23588 | IF4B_HUMAN | 0.00219 | -0.225454787 |
| Q9UHA4 | LTOR3_HUMAN | 0.00227 | -0.400250825 |
| P02787 | TRFE_HUMAN | 0.00266 | -0.313484236 |
| Q8WWP7 | GIMA1_HUMAN | 0.00358 | -0.800861531 |
| Q03405 | UPAR_HUMAN | 0.00505 | -0.37304992 |
| P20702 | ITAX_HUMAN | 0.00533 | -0.724587529 |
| P27348 | 1433T_HUMAN | 0.00598 | -0.442269811 |
| Q14956 | GPNMB_HUMAN | 0.00745 | -0.584249597 |
| Q14728 | MFS10_HUMAN | 0.00757 | -0.548621497 |
| P08670 | VIME_HUMAN | 0.00771 | -0.204137253 |
| Q13510 | ASAH1_HUMAN | 0.00817 | -0.129309492 |
| P45880 | VDAC2_HUMAN | 0.00834 | 0.145858368 |
| Q92499 | DDX1_HUMAN | 0.00895 | -0.523777544 |
| P13693 | TCTP_HUMAN | 0.00937 | -0.238297275 |
| P30622-2 | CLIP1_HUMAN | 0.01199 | -0.376755594 |
| P02774 | VTDB_HUMAN | 0.01442 | -0.428955838 |
| Q15080 | NCF4_HUMAN | 0.01467 | -0.316870982 |
| P34932 | HSP74_HUMAN | 0.01484 | 0.1264061 |
| P36957 | ODO2_HUMAN | 0.01505 | 0.158490468 |
| Q5K4L6 | S27A3_HUMAN | 0.01541 | -0.317202456 |
| A0AV96 | RBM47_HUMAN | 0.01578 | -0.554189651 |
| P19823 | ITIH2_HUMAN | 0.01612 | -0.453274475 |
| A1L0T0 | ILVBL_HUMAN | 0.01657 | 0.314436636 |
| P18510 | IL1RA_HUMAN | 0.01676 | -0.343477452 |
| P02042 | HBD_HUMAN | 0.01719 | -0.40819496 |
| Q6IBS0 | TWF2_HUMAN | 0.01951 | -0.475715652 |
| P33241 | LSP1_HUMAN | 0.02108 | -0.379069646 |
| P50552 | VASP_HUMAN | 0.0216 | -0.146513554 |
| Q9H8S9 | MOB1A_HUMAN | 0.02183 | -0.102171775 |
| O75489 | NDUS3_HUMAN | 0.02245 | -0.233869423 |
| O60711 | LPXN_HUMAN | 0.02245 | -0.281362858 |
| P78527 | PRKDC_HUMAN | 0.02254 | -0.448069134 |
| Q9UBR2 | CATZ_HUMAN | 0.02318 | -0.190324311 |
| P33176 | KINH_HUMAN | 0.02362 | -0.24551567 |
| P15144 | AMPN_HUMAN | 0.02403 | -0.288514072 |
| Q00325-2 | MPCP_HUMAN | 0.02528 | 0.148767003 |
| Q9C002 | NMES1_HUMAN | 0.02529 | -0.311816257 |
| Q00839 | HNRPU_HUMAN | 0.02545 | 0.261636837 |
| Q9BYT8 | NEUL_HUMAN | 0.02637 | -0.381720602 |
| O96000 | NDUBA_HUMAN | 0.02779 | -0.514655183 |
| P0DP25 | CALM3_HUMAN | 0.02857 | -0.224847658 |
| Q6UW68 | TM205_HUMAN | 0.02921 | 0.239184853 |
| O75964 | ATP5L_HUMAN | 0.03164 | -0.552507745 |
| P05543 | THBG_HUMAN | 0.03238 | -0.417788598 |
| O76003 | GLRX3_HUMAN | 0.03265 | -0.247566494 |
| O00154 | BACH_HUMAN | 0.03333 | -0.413913489 |
| Q9UBQ5 | EIF3K_HUMAN | 0.03591 | -0.261596549 |
| Q15904 | VAS1_HUMAN | 0.03678 | -0.584708414 |
| Q15582 | BGH3_HUMAN | 0.03777 | 0.186614779 |
| O43493 | TGON2_HUMAN | 0.0379 | -0.324514716 |
| P83731 | RL24_HUMAN | 0.03861 | -0.530620718 |
| P05387 | RLA2_HUMAN | 0.03872 | -0.323797775 |
| P08865 | RSSA_HUMAN | 0.04051 | -0.120579893 |
| P02511 | CRYAB_HUMAN | 0.04069 | -0.552601318 |
| P13284 | GILT_HUMAN | 0.04162 | -0.57247967 |
| Q14213 | IL27B_HUMAN | 0.04167 | -0.347268794 |
| Q9ULC5-3 | ACSL5_HUMAN | 0.04177 | -0.359723703 |
| P02788 | TRFL_HUMAN | 0.04184 | -0.238398276 |
| Q9H3Z4 | DNJC5_HUMAN | 0.04201 | -0.614798 |
| P08727 | K1C19_HUMAN | 0.04232 | -0.264117308 |
| P54802 | ANAG_HUMAN | 0.04236 | -0.408054861 |
| P49448 | DHE4_HUMAN | 0.04238 | 0.138382656 |
| P08575 | PTPRC_HUMAN | 0.04314 | -0.292053536 |
| P53621 | COPA_HUMAN | 0.04582 | 0.15588184 |
| Q13636 | RAB31_HUMAN | 0.04643 | -0.311288701 |
| Q9BT09 | CNPY3_HUMAN | 0.04671 | -0.16081636 |
| Q9UJS0 | CMC2_HUMAN | 0.04695 | 0.12795158 |
| Q96HY6 | DDRGK_HUMAN | 0.04909 | 0.258806542 |
| P30084 | ECHM_HUMAN | 0.04959 | -0.209349867 |
| P02771 | FETA_HUMAN | 0.04986 | -0.340373689 |
| Q9NZM1 | MYOF_HUMAN | 0.05023 | 0.150285138 |
| Q6IAA8 | LTOR1_HUMAN | 0.0505 | -0.264126201 |
| Q9BTZ2 | DHRS4_HUMAN | 0.05128 | -0.214425679 |
| P51659 | DHB4_HUMAN | 0.05399 | -0.531913324 |
| O75534-4 | CSDE1_HUMAN | 0.05494 | 0.184331084 |
| Q13287 | NMI_HUMAN | 0.05621 | 0.324503851 |
| P52630 | STAT2_HUMAN | 0.0571 | -0.521286837 |
| P12814 | ACTN1_HUMAN | 0.05811 | -0.077947318 |
| P37108 | SRP14_HUMAN | 0.05813 | -0.178843394 |
| P62750 | RL23A_HUMAN | 0.05844 | 0.160033996 |
| P61619 | S61A1_HUMAN | 0.059 | -0.241112772 |
| O43143 | DHX15_HUMAN | 0.05985 | 0.153637036 |
| Q9UQ80 | PA2G4_HUMAN | 0.06099 | -0.258968952 |
| P48960 | CD97_HUMAN | 0.06121 | -0.202509473 |
| P60228 | EIF3E_HUMAN | 0.0627 | 0.127908667 |
| O75915 | PRAF3_HUMAN | 0.06339 | 0.291172162 |
| O00410 | IPO5_HUMAN | 0.06352 | -0.245971371 |
| Q9H299 | SH3L3_HUMAN | 0.06476 | -0.402866706 |
| P18621 | RL17_HUMAN | 0.06482 | 0.229777794 |
| Q14839 | CHD4_HUMAN | 0.06546 | -0.29971203 |
| Q9H832 | UBE2Z_HUMAN | 0.06571 | -0.39792539 |
| Q9HC38 | GLOD4_HUMAN | 0.06601 | -0.18479168 |
| P62753 | RS6_HUMAN | 0.06698 | -0.385960017 |
| Q96SQ9 | CP2S1_HUMAN | 0.06717 | -0.437234399 |
| Q96PP9 | GBP4_HUMAN | 0.06798 | -0.284422005 |
| P30520 | PURA2_HUMAN | 0.06847 | 0.107019261 |
| P23469-2 | PTPRE_HUMAN | 0.0692 | -0.239779011 |
| Q13724 | MOGS_HUMAN | 0.06934 | -0.344620246 |
| P13796 | PLSL_HUMAN | 0.06948 | -0.120563571 |
| Q16778 | H2B2E_HUMAN | 0.0702 | 0.213199566 |
| Q92572 | AP3S1_HUMAN | 0.07039 | 0.21733853 |
| P35610 | SOAT1_HUMAN | 0.07097 | 0.065132658 |
| Q15084-5 | PDIA6_HUMAN | 0.07115 | -0.185769414 |
| P29692-2 | EF1D_HUMAN | 0.07191 | -0.166123901 |
| Q9Y3A6 | TMED5_HUMAN | 0.07321 | -0.254399933 |
| P13798 | ACPH_HUMAN | 0.07332 | 0.134169837 |
| P20701 | ITAL_HUMAN | 0.07375 | -0.215199599 |
| P31040 | SDHA_HUMAN | 0.07389 | 0.199715956 |
| P10599 | THIO_HUMAN | 0.07522 | -0.138442395 |
| Q15637 | SF01_HUMAN | 0.07531 | 0.303254062 |
| P83111 | LACTB_HUMAN | 0.07696 | -0.384363412 |
| Q96TA1 | NIBL1_HUMAN | 0.07705 | 0.708599079 |
| Q14914-2 | PTGR1_HUMAN | 0.07797 | -0.447505098 |
| P05161 | ISG15_HUMAN | 0.0796 | 0.247369974 |
| Q96Q11 | TRNT1_HUMAN | 0.08024 | -0.473471534 |
| Q99653 | CHP1_HUMAN | 0.0808 | -0.387987553 |
| P13473 | LAMP2_HUMAN | 0.08181 | -0.218890164 |
| O15511 | ARPC5_HUMAN | 0.08302 | -0.130189634 |
| Q04637-8 | IF4G1_HUMAN | 0.08323 | -0.071337415 |
| P62834 | RAP1A_HUMAN | 0.08512 | 0.411084577 |
| P40227 | TCPZ_HUMAN | 0.08536 | -0.336615793 |
| Q16576 | RBBP7_HUMAN | 0.08604 | -0.216733204 |
| Q8N766 | EMC1_HUMAN | 0.08628 | -0.228480399 |
| P21926 | CD9_HUMAN | 0.08632 | -0.373909499 |
| Q86TU7 | SETD3_HUMAN | 0.08644 | -0.358985114 |
| P47914 | RL29_HUMAN | 0.08678 | 0.216598758 |
| P15121 | ALDR_HUMAN | 0.08817 | 0.488358793 |
| P27105 | STOM_HUMAN | 0.08819 | 0.134279199 |
| Q9Y4P3 | TBL2_HUMAN | 0.09054 | -0.223720902 |
| P15311 | EZRI_HUMAN | 0.09061 | 0.112390085 |
| P61923 | COPZ1_HUMAN | 0.09256 | -0.232113097 |
| O43252 | PAPS1_HUMAN | 0.09403 | -0.256712601 |
| P02768 | ALBU_HUMAN | 0.0946 | -0.417681058 |
| Q969X1 | LFG3_HUMAN | 0.09479 | 0.243318428 |
| P30049 | ATPD_HUMAN | 0.09551 | -0.190013811 |
| P40763 | STAT3_HUMAN | 0.09578 | -0.195774903 |
| P10619 | PPGB_HUMAN | 0.09724 | -0.345586492 |
| P13010 | XRCC5_HUMAN | 0.09758 | 0.104586021 |
| P16070 | CD44_HUMAN | 0.09775 | -0.124464261 |
| Q8TAT6 | NPL4_HUMAN | 0.09819 | -0.183773753 |
| P35222 | CTNB1_HUMAN | 0.09893 | 0.234579768 |
| P17813 | EGLN_HUMAN | 0.09965 | -0.241568316 |
| Q9UHB9 | SRP68_HUMAN | 0.1 | 0.173951796 |
| P62993 | GRB2_HUMAN | 0.10158 | -0.26790623 |
| P11047 | LAMC1_HUMAN | 0.10158 | -0.318847397 |
| P15153 | RAC2_HUMAN | 0.10222 | -0.226127207 |
| Q9UGQ3 | GTR6_HUMAN | 0.10423 | -0.206022264 |
| P00846 | ATP6_HUMAN | 0.10425 | -0.41008913 |
| O95373 | IPO7_HUMAN | 0.10551 | -0.191226544 |
| Q7Z4W1 | DCXR_HUMAN | 0.10582 | 0.118019453 |
| Q9Y5S9 | RBM8A_HUMAN | 0.10615 | -0.209295506 |
| P00441 | SODC_HUMAN | 0.10674 | -0.203917369 |
| Q8IZ83 | A16A1_HUMAN | 0.10676 | 0.243225287 |
| Q9BYU5 | KRA21_HUMAN | 0.10686 | 0.498628911 |
| P39656 | OST48_HUMAN | 0.10722 | 0.083317814 |
| P49207 | RL34_HUMAN | 0.10752 | 0.269795454 |
| Q14240 | IF4A2_HUMAN | 0.1077 | 0.222449248 |
| P51572 | BAP31_HUMAN | 0.10789 | 0.291640795 |
| Q15758 | AAAT_HUMAN | 0.1081 | -0.218307412 |
| Q96HC4 | PDLI5_HUMAN | 0.10908 | 0.20293733 |
| Q16543 | CDC37_HUMAN | 0.1093 | -0.178311836 |
| P20160 | CAP7_HUMAN | 0.10932 | -0.216702007 |
| Q9UBI6 | GBG12_HUMAN | 0.11002 | -0.252148823 |
| P08754 | GNAI3_HUMAN | 0.11179 | 0.120962842 |
| P29350 | PTN6_HUMAN | 0.11299 | -0.112739532 |
| P04843 | RPN1_HUMAN | 0.11327 | 0.06222849 |
| Q8IY17 | PLPL6_HUMAN | 0.11364 | 0.315871228 |
| P50990 | TCPQ_HUMAN | 0.11372 | 0.140195744 |
| O15127 | SCAM2_HUMAN | 0.11407 | 0.144780481 |
| O15400 | STX7_HUMAN | 0.1144 | -0.139285908 |
| Q8TD55 | PKHO2_HUMAN | 0.11668 | -0.166341533 |
| P05165 | PCCA_HUMAN | 0.11726 | -0.15761921 |
| Q15435 | PP1R7_HUMAN | 0.11732 | -0.519546478 |
| P20036 | DPA1_HUMAN | 0.11832 | -0.250566857 |
| P49411 | EFTU_HUMAN | 0.11903 | -0.414052989 |
| Q03519 | TAP2_HUMAN | 0.11991 | 0.203078497 |
| P09917 | LOX5_HUMAN | 0.12005 | -0.214961308 |
| Q9Y5P6-2 | GMPPB_HUMAN | 0.12057 | -0.863856028 |
| Q9NUQ9 | FA49B_HUMAN | 0.12084 | -0.07080204 |
| Q9ULZ3 | ASC_HUMAN | 0.12096 | -0.150377719 |
| Q02790 | FKBP4_HUMAN | 0.1225 | 0.104578397 |
| Q9H4G4 | GAPR1_HUMAN | 0.1228 | 0.351616097 |
| O75477 | ERLN1_HUMAN | 0.12298 | 0.192125261 |
| Q99805 | TM9S2_HUMAN | 0.12319 | -0.134915352 |
| Q9UHD8 | SEPT9_HUMAN | 0.12453 | 0.129843869 |
| P08842 | STS_HUMAN | 0.12464 | 0.282885805 |
| O15118 | NPC1_HUMAN | 0.1249 | 0.087418389 |
| P63104 | 1433Z_HUMAN | 0.12535 | -0.225404554 |
| Q9Y5X3 | SNX5_HUMAN | 0.12581 | -0.218839609 |
| P13489 | RINI_HUMAN | 0.12616 | -0.075054436 |
| Q9H4M9 | EHD1_HUMAN | 0.12635 | -0.217557793 |
| P62318 | SMD3_HUMAN | 0.1265 | 0.155050989 |
| P48643 | TCPE_HUMAN | 0.12723 | -0.158997853 |
| P24534 | EF1B_HUMAN | 0.12846 | -0.203731007 |
| P61026 | RAB10_HUMAN | 0.1295 | -0.124217766 |
| P07858 | CATB_HUMAN | 0.13001 | -0.132556088 |
| P50897 | PPT1_HUMAN | 0.13035 | -0.350715029 |
| P58546 | MTPN_HUMAN | 0.13106 | -0.224765973 |
| P35270 | SPRE_HUMAN | 0.1326 | -0.14472834 |
| P67812 | SC11A_HUMAN | 0.13273 | -0.197394456 |
| P49354 | FNTA_HUMAN | 0.13304 | -0.188389312 |
| Q14008 | CKAP5_HUMAN | 0.13355 | -0.187066519 |
| P61353 | RL27_HUMAN | 0.1341 | 0.256828722 |
| Q16775 | GLO2_HUMAN | 0.13531 | -0.208063496 |
| P08962 | CD63_HUMAN | 0.13548 | -0.196226353 |
| Q8TDB6 | DTX3L_HUMAN | 0.13652 | -0.207087559 |
| P12110 | CO6A2_HUMAN | 0.13792 | -0.354930198 |
| O60271 | JIP4_HUMAN | 0.13918 | -0.574533056 |
| Q92522 | H1X_HUMAN | 0.14043 | 0.172396813 |
| P60660-2 | MYL6_HUMAN | 0.1407 | -0.117475812 |
| P35580 | MYH10_HUMAN | 0.14183 | 0.189741154 |
| Q06033 | ITIH3_HUMAN | 0.14284 | -0.125684853 |
| Q01469 | FABP5_HUMAN | 0.14403 | -0.35504217 |
| Q9UBV8 | PEF1_HUMAN | 0.14495 | -0.387106026 |
| P10644 | KAP0_HUMAN | 0.14512 | -0.188600274 |
| Q9H939 | PPIP2_HUMAN | 0.14514 | 0.221335056 |
| O43399-7 | TPD54_HUMAN | 0.14559 | -0.169270514 |
| Q15907 | RB11B_HUMAN | 0.14665 | -0.056432053 |
| P24539 | AT5F1_HUMAN | 0.14671 | 0.133712613 |
| P19878 | NCF2_HUMAN | 0.1469 | 0.153777398 |
| Q04837 | SSBP_HUMAN | 0.14728 | 0.231093067 |
| P53680 | AP2S1_HUMAN | 0.14844 | 0.132394133 |
| P35232 | PHB_HUMAN | 0.14888 | 0.104394395 |
| P57053 | H2BFS_HUMAN | 0.14941 | 0.223561077 |
| Q9NY33 | DPP3_HUMAN | 0.14957 | 0.191712841 |
| Q96CW1 | AP2M1_HUMAN | 0.15072 | 0.366381768 |
| P06702 | S10A9_HUMAN | 0.15135 | -0.151073421 |
| Q92973 | TNPO1_HUMAN | 0.15175 | 0.235306027 |
| P04792 | HSPB1_HUMAN | 0.15261 | -0.145495972 |
| P12111 | CO6A3_HUMAN | 0.15379 | -0.340686895 |
| P23396 | RS3_HUMAN | 0.15506 | 0.172180103 |
| Q00796 | DHSO_HUMAN | 0.15524 | -0.226206671 |
| P61513 | RL37A_HUMAN | 0.15599 | 0.249988272 |
| Q14165 | MLEC_HUMAN | 0.15673 | 0.118344576 |
| P16671 | CD36_HUMAN | 0.15687 | -0.170668282 |
| P23284 | PPIB_HUMAN | 0.15697 | -0.061113798 |
| P62277 | RS13_HUMAN | 0.15749 | 0.209154986 |
| P05107 | ITB2_HUMAN | 0.15759 | -0.13934099 |
| Q9H0W9 | CK054_HUMAN | 0.15766 | -0.160896849 |
| Q92945 | FUBP2_HUMAN | 0.15802 | 0.080546771 |
| P15924 | DESP_HUMAN | 0.15851 | 0.278644977 |
| P36543 | VATE1_HUMAN | 0.15908 | -0.112172451 |
| P12956 | XRCC6_HUMAN | 0.15934 | 0.101006551 |
| Q8TC12 | RDH11_HUMAN | 0.16082 | -0.196553715 |
| P63010 | AP2B1_HUMAN | 0.16243 | 0.139020117 |
| Q9UNF0 | PACN2_HUMAN | 0.16307 | -0.175674407 |
| P27487 | DPP4_HUMAN | 0.1636 | 0.084573429 |
| Q7Z5R6 | AB1IP_HUMAN | 0.16468 | 0.136431279 |
| P61225 | RAP2B_HUMAN | 0.16601 | 0.210539574 |
| O00487 | PSDE_HUMAN | 0.16615 | 0.080963362 |
| Q5SSJ5 | HP1B3_HUMAN | 0.16665 | -0.215393653 |
| P07195 | LDHB_HUMAN | 0.16681 | 0.047653431 |
| P51606 | RENBP_HUMAN | 0.1682 | 0.204089343 |
| P50213 | IDH3A_HUMAN | 0.16937 | -0.177224849 |
| P00505 | AATM_HUMAN | 0.17014 | -0.18969631 |
| Q9Y5L0 | TNPO3_HUMAN | 0.17035 | 0.601648705 |
| Q9Y224 | RTRAF_HUMAN | 0.17059 | -0.230989003 |
| P62917 | RL8_HUMAN | 0.17087 | 0.173723657 |
| Q01995 | TAGL_HUMAN | 0.1714 | -0.621000628 |
| P43003 | EAA1_HUMAN | 0.17325 | -0.227617193 |
| P37837 | TALDO_HUMAN | 0.17367 | -0.127873705 |
| P61758 | PFD3_HUMAN | 0.17412 | -0.34537619 |
| P05413 | FABPH_HUMAN | 0.17479 | -0.624239035 |
| P50416 | CPT1A_HUMAN | 0.17513 | 0.548360357 |
| P30041 | PRDX6_HUMAN | 0.17624 | -0.181638865 |
| Q13011 | ECH1_HUMAN | 0.17624 | 0.161674037 |
| Q86Y39 | NDUAB_HUMAN | 0.17638 | -0.291393819 |
| P48047 | ATPO_HUMAN | 0.17677 | 0.157643507 |
| Q9BWS9 | CHID1_HUMAN | 0.17683 | -0.264240097 |
| P36551 | HEM6_HUMAN | 0.17928 | -0.220107606 |
| Q01082 | SPTB2_HUMAN | 0.17938 | 0.149430331 |
| P01023 | A2MG_HUMAN | 0.17954 | -0.269536962 |
| P19801 | AOC1_HUMAN | 0.18094 | -0.382213122 |
| Q13303-3 | KCAB2_HUMAN | 0.18138 | -0.19106946 |
| P46776 | RL27A_HUMAN | 0.18142 | 0.173201252 |
| P62314 | SMD1_HUMAN | 0.1822 | 0.106960451 |
| Q27J81 | INF2_HUMAN | 0.18233 | -0.128587853 |
| O95379 | TFIP8_HUMAN | 0.18243 | -0.206673019 |
| Q15006 | EMC2_HUMAN | 0.186 | -0.140908762 |
| P62899 | RL31_HUMAN | 0.18667 | 0.134476326 |
| P30048 | PRDX3_HUMAN | 0.18699 | -0.215077797 |
| P48163 | MAOX_HUMAN | 0.18783 | 0.282041142 |
| Q9UBQ7 | GRHPR_HUMAN | 0.18929 | 0.10337116 |
| Q8IWB7 | WDFY1_HUMAN | 0.18953 | -0.330820594 |
| Q9BRX8 | PXL2A_HUMAN | 0.18954 | -0.273642389 |
| Q92597 | NDRG1_HUMAN | 0.19002 | -0.105409975 |
| Q9Y696 | CLIC4_HUMAN | 0.19026 | -0.208308477 |
| O95777 | LSM8_HUMAN | 0.19134 | -0.320012437 |
| Q8TEM1 | PO210_HUMAN | 0.19206 | 0.200483532 |
| Q9Y6C9 | MTCH2_HUMAN | 0.19288 | 0.094776936 |
| Q9H008 | LHPP_HUMAN | 0.19342 | -0.251770882 |
| P09012 | SNRPA_HUMAN | 0.19398 | -0.293327168 |
| Q15393 | SF3B3_HUMAN | 0.19536 | 0.072000994 |
| O95197 | RTN3_HUMAN | 0.1962 | -0.173898262 |
| O15427 | MOT4_HUMAN | 0.19643 | -0.206254297 |
| O95486 | SC24A_HUMAN | 0.19649 | 0.391453823 |
| P36578 | RL4_HUMAN | 0.19717 | 0.161244432 |
| P17931 | LEG3_HUMAN | 0.19806 | -0.1044361 |
| Q9NQW7 | XPP1_HUMAN | 0.19854 | 0.079742552 |
| P31939 | PUR9_HUMAN | 0.19885 | -0.148924558 |
| Q96GA7 | SDSL_HUMAN | 0.19886 | -0.093521767 |
| Q9P2R7 | SUCB1_HUMAN | 0.19891 | -0.18296372 |
| Q03135 | CAV1_HUMAN | 0.19899 | -0.205883385 |
| Q9P035 | HACD3_HUMAN | 0.19955 | 0.17302329 |
| P06737 | PYGL_HUMAN | 0.19976 | 0.205688872 |
| O60826 | CCD22_HUMAN | 0.20024 | -0.373963821 |
| P12109 | CO6A1_HUMAN | 0.2003 | -0.379811728 |
| P35573 | GDE_HUMAN | 0.20037 | 0.201044083 |
| Q92930 | RAB8B_HUMAN | 0.20046 | -0.154316389 |
| Q03169 | TNAP2_HUMAN | 0.20279 | -0.341594575 |
| Q9Y262 | EIF3L_HUMAN | 0.2036 | 0.114169001 |
| P21283 | VATC1_HUMAN | 0.20525 | -0.209292551 |
| Q13488 | VPP3_HUMAN | 0.20587 | -0.125687647 |
| P35579 | MYH9_HUMAN | 0.20617 | 0.105515425 |
| P14061 | DHB1_HUMAN | 0.20708 | -0.414909608 |
| P54652 | HSP72_HUMAN | 0.20825 | 0.129533619 |
| P62873 | GBB1_HUMAN | 0.20926 | 0.120445652 |
| Q9Y2B0 | CNPY2_HUMAN | 0.20955 | -0.214453281 |
| P15880 | RS2_HUMAN | 0.20985 | 0.130770587 |
| P11387 | TOP1_HUMAN | 0.21124 | -0.325943893 |
| Q08722 | CD47_HUMAN | 0.21143 | 0.085459413 |
| Q9Y2S2 | CRYL1_HUMAN | 0.21232 | -0.280104275 |
| P30153 | 2AAA_HUMAN | 0.21241 | -0.15767924 |
| P67936 | TPM4_HUMAN | 0.21285 | 0.076997569 |
| O75306 | NDUS2_HUMAN | 0.21367 | -0.310217421 |
| Q96D96 | HVCN1_HUMAN | 0.2147 | 0.154423852 |
| Q15029 | U5S1_HUMAN | 0.21488 | 0.087080743 |
| Q8IXB1 | DJC10_HUMAN | 0.21573 | -0.249022361 |
| P55084 | ECHB_HUMAN | 0.21588 | -0.252788712 |
| Q6P996 | PDXD1_HUMAN | 0.22027 | -0.175233507 |
| P14550 | AK1A1_HUMAN | 0.22046 | 0.127686792 |
| O15143 | ARC1B_HUMAN | 0.22211 | 0.092724563 |
| Q15019 | SEPT2_HUMAN | 0.22253 | 0.171869388 |
| P21333 | FLNA_HUMAN | 0.22324 | -0.076171775 |
| P21589 | 5NTD_HUMAN | 0.22378 | -0.263786211 |
| Q08J23 | NSUN2_HUMAN | 0.22445 | -0.222580772 |
| P02792 | FRIL_HUMAN | 0.22482 | -0.26806584 |
| P39687 | AN32A_HUMAN | 0.22521 | 0.484788376 |
| O96005 | CLPT1_HUMAN | 0.22678 | 0.148897333 |
| Q99798 | ACON_HUMAN | 0.2277 | -0.074453573 |
| P09110 | THIK_HUMAN | 0.22774 | 0.216287682 |
| P46782 | RS5_HUMAN | 0.22802 | -0.079825634 |
| Q53GQ0 | DHB12_HUMAN | 0.22848 | -0.189608007 |
| O43684 | BUB3_HUMAN | 0.22937 | 0.255430934 |
| P29966 | MARCS_HUMAN | 0.23004 | -0.136369336 |
| O14744-5 | ANM5_HUMAN | 0.23046 | -0.261933256 |
| Q8TCU6 | PREX1_HUMAN | 0.23075 | -0.40460094 |
| Q9P2E9 | RRBP1_HUMAN | 0.23116 | 0.158530952 |
| Q86Y82 | STX12_HUMAN | 0.23156 | -0.116358659 |
| P54886 | P5CS_HUMAN | 0.23186 | -0.320444577 |
| P52790 | HXK3_HUMAN | 0.23204 | -0.138281003 |
| Q13283 | G3BP1_HUMAN | 0.23418 | -0.214006811 |
| P62851 | RS25_HUMAN | 0.23483 | 0.165143235 |
| Q13200 | PSMD2_HUMAN | 0.23529 | 0.142503594 |
| P0DOX5 | IGG1_HUMAN | 0.23669 | -0.258406648 |
| P50135 | HNMT_HUMAN | 0.23722 | -0.136165491 |
| P07384 | CAN1_HUMAN | 0.23765 | 0.165357669 |
| P20292 | AL5AP_HUMAN | 0.23889 | -0.141965008 |
| Q68CZ2 | TENS3_HUMAN | 0.24001 | 0.271218933 |
| Q7L7X3 | TAOK1_HUMAN | 0.24056 | 0.154327667 |
| P28067 | DMA_HUMAN | 0.24226 | 0.151571162 |
| P62937 | PPIA_HUMAN | 0.24262 | -0.143173638 |
| P11166 | GTR1_HUMAN | 0.24276 | 0.174426471 |
| P00918 | CAH2_HUMAN | 0.24282 | -0.129543746 |
| P30484 | 1B46_HUMAN | 0.24309 | -0.40198043 |
| P62826 | RAN_HUMAN | 0.24342 | 0.071145579 |
| Q14152 | EIF3A_HUMAN | 0.24419 | 0.097703537 |
| O94979 | SC31A_HUMAN | 0.24442 | 0.133741481 |
| P07900-2 | HS90A_HUMAN | 0.24471 | 0.0529422 |
| P14780 | MMP9_HUMAN | 0.24569 | -0.294079962 |
| Q96N66 | MBOA7_HUMAN | 0.24597 | 0.265607329 |
| P40939 | ECHA_HUMAN | 0.24639 | 0.190908173 |
| P46940 | IQGA1_HUMAN | 0.24668 | 0.047645875 |
| P21980 | TGM2_HUMAN | 0.24699 | 0.199344441 |
| O43776 | SYNC_HUMAN | 0.2471 | -0.107985588 |
| P30626 | SORCN_HUMAN | 0.24713 | -0.162426445 |
| P32969 | RL9_HUMAN | 0.24728 | 0.102082672 |
| P69891 | HBG1_HUMAN | 0.24861 | -0.181660245 |
| P52566 | GDIR2_HUMAN | 0.24884 | -0.04049065 |
| P08195 | 4F2_HUMAN | 0.25335 | 0.122221454 |
| P55160 | NCKPL_HUMAN | 0.25504 | -0.133988757 |
| Q9Y639 | NPTN_HUMAN | 0.25554 | -0.189501413 |
| O95674 | CDS2_HUMAN | 0.25747 | 0.121652856 |
| Q01518 | CAP1_HUMAN | 0.25814 | -0.051690866 |
| O00299 | CLIC1_HUMAN | 0.26005 | -0.135049039 |
| Q3V6T2 | GRDN_HUMAN | 0.2603 | 0.182690516 |
| Q9NQR4 | NIT2_HUMAN | 0.26078 | -0.544365827 |
| P35754 | GLRX1_HUMAN | 0.26194 | -0.171796208 |
| P28331 | NDUS1_HUMAN | 0.2627 | -0.152453582 |
| Q12979 | ABR_HUMAN | 0.26288 | 0.17651072 |
| P48426 | PI42A_HUMAN | 0.26406 | -0.320800052 |
| P05198 | IF2A_HUMAN | 0.26439 | 0.069158134 |
| P34910-2 | EVI2B_HUMAN | 0.26473 | 0.105259946 |
| Q05209 | PTN12_HUMAN | 0.26502 | -0.181242924 |
| P05187 | PPB1_HUMAN | 0.26506 | -0.236187035 |
| P31689 | DNJA1_HUMAN | 0.26646 | 0.065311326 |
| P27797 | CALR_HUMAN | 0.26766 | -0.070753825 |
| P27986 | P85A_HUMAN | 0.26811 | -0.495107668 |
| O75368 | SH3L1_HUMAN | 0.26822 | -0.230290865 |
| P05455 | LA_HUMAN | 0.26909 | -0.221370148 |
| Q9HB71 | CYBP_HUMAN | 0.26954 | 0.102057577 |
| Q13885 | TBB2A_HUMAN | 0.26961 | -0.210026421 |
| P48556 | PSMD8_HUMAN | 0.27021 | 0.200360323 |
| Q9NP72 | RAB18_HUMAN | 0.27054 | 0.056166122 |
| P07954 | FUMH_HUMAN | 0.27153 | 0.08563028 |
| P50502 | F10A1_HUMAN | 0.27166 | -0.14168801 |
| Q96IJ6 | GMPPA_HUMAN | 0.27171 | 0.224745179 |
| Q99829 | CPNE1_HUMAN | 0.27258 | -0.193310664 |
| P31153 | METK2_HUMAN | 0.27282 | 0.113328585 |
| Q86VP6 | CAND1_HUMAN | 0.27332 | 0.061483134 |
| O15533 | TPSN_HUMAN | 0.27458 | -0.166154655 |
| P35606 | COPB2_HUMAN | 0.27469 | 0.148571971 |
| Q658Y4 | F91A1_HUMAN | 0.27488 | -0.471054806 |
| P04075 | ALDOA_HUMAN | 0.27493 | 0.065620466 |
| Q9H1C4 | UN93B_HUMAN | 0.27527 | -0.470525904 |
| Q8IXQ6 | PARP9_HUMAN | 0.27529 | -0.320836026 |
| Q6P587 | FAHD1_HUMAN | 0.27532 | -0.155442621 |
| P20618 | PSB1_HUMAN | 0.27537 | 0.060344276 |
| Q01813 | PFKAP_HUMAN | 0.27654 | 0.488692419 |
| Q9NX14 | NDUBB_HUMAN | 0.27681 | 0.155408043 |
| P68104 | EF1A1_HUMAN | 0.27722 | 0.075334113 |
| P42126 | ECI1_HUMAN | 0.2776 | -0.154873609 |
| P41091 | IF2G_HUMAN | 0.27821 | 0.183359645 |
| Q5EB52 | MEST_HUMAN | 0.27825 | 0.23998419 |
| P38117 | ETFB_HUMAN | 0.27851 | -0.078778342 |
| Q8NCW5 | NNRE_HUMAN | 0.27899 | -0.298086884 |
| P04040 | CATA_HUMAN | 0.28067 | 0.202776169 |
| P12830 | CADH1_HUMAN | 0.28098 | 0.123334122 |
| O95782 | AP2A1_HUMAN | 0.28105 | 0.194242073 |
| P35241 | RADI_HUMAN | 0.28399 | -0.086248587 |
| P12724 | ECP_HUMAN | 0.28452 | -0.22098653 |
| P35268 | RL22_HUMAN | 0.28638 | 0.107582415 |
| P02647 | APOA1_HUMAN | 0.28953 | -0.233308658 |
| P54577 | SYYC_HUMAN | 0.29071 | -0.139923424 |
| Q9UHD1 | CHRD1_HUMAN | 0.29156 | 0.159814968 |
| Q16853 | AOC3_HUMAN | 0.29206 | -0.227194791 |
| P07711 | CATL1_HUMAN | 0.29356 | -0.169710374 |
| Q5BJH7 | YIF1B_HUMAN | 0.29432 | -0.195878945 |
| Q13459 | MYO9B_HUMAN | 0.29479 | -0.147631785 |
| P61313 | RL15_HUMAN | 0.29479 | 0.123561539 |
| P39019 | RS19_HUMAN | 0.2968 | -0.115566064 |
| Q9UBE0 | SAE1_HUMAN | 0.29713 | -0.199377106 |
| Q16836 | HCDH_HUMAN | 0.29814 | -0.667757898 |
| P04216 | THY1_HUMAN | 0.29883 | -0.205554634 |
| Q02750 | MP2K1_HUMAN | 0.29959 | -0.17757562 |
| Q460N5 | PAR14_HUMAN | 0.29974 | 0.182595561 |
| Q86UX7 | URP2_HUMAN | 0.30012 | -0.083586991 |
| P62081 | RS7_HUMAN | 0.30215 | -0.357992829 |
| P57740 | NU107_HUMAN | 0.30264 | -0.102774779 |
| P14927 | QCR7_HUMAN | 0.30444 | -0.143794255 |
| A0MZ66 | SHOT1_HUMAN | 0.30482 | 0.137014792 |
| P08567 | PLEK_HUMAN | 0.30504 | -0.190595186 |
| P46783 | RS10_HUMAN | 0.30529 | -0.166035433 |
| P47756-2 | CAPZB_HUMAN | 0.30645 | 0.057884954 |
| P41567 | EIF1_HUMAN | 0.30709 | -0.168891738 |
| P08571 | CD14_HUMAN | 0.30725 | -0.11471241 |
| Q5T447 | HECD3_HUMAN | 0.30757 | -0.469972831 |
| O00159 | MYO1C_HUMAN | 0.30803 | 0.114369879 |
| O43390 | HNRPR_HUMAN | 0.30807 | 0.13299534 |
| O94766 | B3GA3_HUMAN | 0.30903 | 0.207219846 |
| Q30134 | 2B18_HUMAN | 0.30918 | 0.223910803 |
| P26885 | FKBP2_HUMAN | 0.30965 | 0.080173177 |
| P55769 | NH2L1_HUMAN | 0.31035 | 0.174316316 |
| O94903 | PLPHP_HUMAN | 0.31044 | -0.121886956 |
| Q92882 | OSTF1_HUMAN | 0.31094 | -0.114837582 |
| O60506 | HNRPQ_HUMAN | 0.3117 | 0.078752152 |
| O14828 | SCAM3_HUMAN | 0.31175 | -0.075409181 |
| P04179 | SODM_HUMAN | 0.31256 | -0.112620915 |
| Q96AG4 | LRC59_HUMAN | 0.31275 | -0.158827676 |
| P35237 | SPB6_HUMAN | 0.3137 | -0.110566234 |
| Q9P0J0 | NDUAD_HUMAN | 0.31387 | -0.156524384 |
| P10253 | LYAG_HUMAN | 0.31408 | -0.173954355 |
| P04899 | GNAI2_HUMAN | 0.31504 | 0.043063627 |
| P25685 | DNJB1_HUMAN | 0.31555 | -0.099259409 |
| Q7Z6Z7 | HUWE1_HUMAN | 0.3156 | -0.580218826 |
| P42766 | RL35_HUMAN | 0.31578 | 0.095282731 |
| P62979 | RS27A_HUMAN | 0.3163 | 0.033144678 |
| P20674 | COX5A_HUMAN | 0.31685 | -0.1257366 |
| Q16630 | CPSF6_HUMAN | 0.31719 | -0.117534756 |
| P32119 | PRDX2_HUMAN | 0.31727 | -0.108814764 |
| P51665 | PSMD7_HUMAN | 0.31756 | -0.148238221 |
| O43707 | ACTN4_HUMAN | 0.31891 | 0.044319363 |
| Q13492 | PICAL_HUMAN | 0.32042 | -0.27929813 |
| Q92616 | GCN1_HUMAN | 0.32046 | 0.318892024 |
| Q9Y4L1 | HYOU1_HUMAN | 0.32107 | 0.081975029 |
| P13639 | EF2_HUMAN | 0.3211 | 0.061937123 |
| Q15185 | TEBP_HUMAN | 0.3211 | -0.16858492 |
| P61289 | PSME3_HUMAN | 0.32183 | 0.147551005 |
| Q99541 | PLIN2_HUMAN | 0.32198 | -0.161778159 |
| Q9NXW2 | DJB12_HUMAN | 0.32247 | -0.475857759 |
| Q9Y6W5 | WASF2_HUMAN | 0.32355 | 0.069809357 |
| P84074 | HPCA_HUMAN | 0.32401 | -0.294530332 |
| P78324 | SHPS1_HUMAN | 0.32755 | -0.173891823 |
| P02747 | C1QC_HUMAN | 0.32802 | 0.111415257 |
| O60568 | PLOD3_HUMAN | 0.32818 | 0.086056991 |
| P67775 | PP2AA_HUMAN | 0.3288 | -0.246042725 |
| Q13162 | PRDX4_HUMAN | 0.33076 | -0.125186964 |
| Q15363 | TMED2_HUMAN | 0.3311 | -0.14781366 |
| Q9P0L0 | VAPA_HUMAN | 0.33152 | -0.04561419 |
| P02746 | C1QB_HUMAN | 0.33198 | 0.164655586 |
| O14874-2 | BCKD_HUMAN | 0.33286 | -0.801675734 |
| Q16186 | ADRM1_HUMAN | 0.33315 | 0.138408871 |
| Q9Y570 | PPME1_HUMAN | 0.33373 | 0.442463535 |
| O00625 | PIR_HUMAN | 0.33468 | -0.374457561 |
| P02679 | FIBG_HUMAN | 0.33497 | -0.129695007 |
| Q9Y4D7 | PLXD1_HUMAN | 0.33555 | 0.213828253 |
| O60664 | PLIN3_HUMAN | 0.33567 | -0.140973742 |
| P62424 | RL7A_HUMAN | 0.33576 | 0.082135346 |
| P30273 | FCERG_HUMAN | 0.33669 | -0.062137731 |
| P23434 | GCSH_HUMAN | 0.33705 | 0.2773241 |
| P12004 | PCNA_HUMAN | 0.3373 | 0.104025805 |
| P06748 | NPM_HUMAN | 0.33733 | -0.34293295 |
| O75907 | DGAT1_HUMAN | 0.33745 | -0.677414714 |
| P46459 | NSF_HUMAN | 0.3375 | 0.128212624 |
| P13987-2 | CD59_HUMAN | 0.33775 | 0.09128456 |
| O43760 | SNG2_HUMAN | 0.33912 | -0.088528342 |
| A6NHR9 | SMHD1_HUMAN | 0.34035 | -0.227663828 |
| P17987 | TCPA_HUMAN | 0.34134 | 0.065033994 |
| P51148 | RAB5C_HUMAN | 0.3415 | 0.182673718 |
| P28072 | PSB6_HUMAN | 0.34154 | 0.071604165 |
| P55072 | TERA_HUMAN | 0.34156 | -0.088454851 |
| Q6YP21 | KAT3_HUMAN | 0.34177 | -0.125222614 |
| P49721 | PSB2_HUMAN | 0.34193 | -0.86012702 |
| Q16822 | PCKGM_HUMAN | 0.34233 | 0.14343423 |
| O43852 | CALU_HUMAN | 0.34237 | -0.138948576 |
| P60842 | IF4A1_HUMAN | 0.34353 | -0.118142648 |
| O95236 | APOL3_HUMAN | 0.34431 | -0.476766855 |
| Q9UM00 | TMCO1_HUMAN | 0.34542 | -0.199831818 |
| P30457 | 1A66_HUMAN | 0.34671 | -0.225067697 |
| Q9NSB4 | KRT82_HUMAN | 0.34819 | 0.560693721 |
| P16278 | BGAL_HUMAN | 0.34915 | 0.127206177 |
| P25705 | ATPA_HUMAN | 0.34959 | 0.052774839 |
| Q92609 | TBCD5_HUMAN | 0.34962 | -0.351994469 |
| Q12907 | LMAN2_HUMAN | 0.34995 | -0.10095747 |
| P52565 | GDIR1_HUMAN | 0.35011 | 0.055376281 |
| O15258 | RER1_HUMAN | 0.35042 | -0.117697905 |
| P38646 | GRP75_HUMAN | 0.35094 | -0.037734716 |
| Q9Y6N5 | SQOR_HUMAN | 0.3528 | 0.098599044 |
| P52657 | T2AG_HUMAN | 0.35483 | 0.065081181 |
| Q9Y6K5 | OAS3_HUMAN | 0.35538 | -0.218651555 |
| Q9BS26 | ERP44_HUMAN | 0.35675 | -0.063955139 |
| Q13404 | UB2V1_HUMAN | 0.35686 | -0.118291543 |
| O75298 | RTN2_HUMAN | 0.35689 | 0.232712585 |
| P26038 | MOES_HUMAN | 0.35761 | 0.071178221 |
| A5YKK6 | CNOT1_HUMAN | 0.35761 | 0.248103351 |
| Q6YN16 | HSDL2_HUMAN | 0.35776 | -0.157719804 |
| O75874 | IDHC_HUMAN | 0.35868 | -0.079274688 |
| O95292 | VAPB_HUMAN | 0.35973 | 0.089255967 |
| P53992 | SC24C_HUMAN | 0.36017 | -0.19045733 |
| P49720 | PSB3_HUMAN | 0.36026 | -0.107007313 |
| P80723 | BASP1_HUMAN | 0.36029 | 0.070588594 |
| Q9NZ45 | CISD1_HUMAN | 0.3604 | 0.154930456 |
| Q9NZ08 | ERAP1_HUMAN | 0.36051 | -0.094751443 |
| P52789 | HXK2_HUMAN | 0.36351 | -0.098514504 |
| P16152 | CBR1_HUMAN | 0.36367 | -0.079318683 |
| P18124 | RL7_HUMAN | 0.36374 | 0.072707045 |
| O60610 | DIAP1_HUMAN | 0.36437 | -0.111664694 |
| P62195 | PRS8_HUMAN | 0.36525 | -0.455378948 |
| Q09161 | NCBP1_HUMAN | 0.36581 | -0.128423916 |
| Q9BZF1 | OSBL8_HUMAN | 0.36593 | 0.203826564 |
| Q99439 | CNN2_HUMAN | 0.36644 | -0.084619465 |
| Q9HC35 | EMAL4_HUMAN | 0.36709 | 0.103178746 |
| Q9NPH2 | INO1_HUMAN | 0.36731 | 0.147107692 |
| P69892 | HBG2_HUMAN | 0.36798 | -0.143899993 |
| P01903 | DRA_HUMAN | 0.36893 | -0.123515952 |
| P30536 | TSPO_HUMAN | 0.36907 | -0.592886015 |
| P68371 | TBB4B_HUMAN | 0.36922 | 0.052458324 |
| Q14166 | TTL12_HUMAN | 0.36945 | -0.148630062 |
| P28065 | PSB9_HUMAN | 0.36994 | 0.092006666 |
| P30044 | PRDX5_HUMAN | 0.37016 | 0.121356452 |
| Q7L2H7 | EIF3M_HUMAN | 0.37123 | -0.155035742 |
| P53007 | TXTP_HUMAN | 0.37155 | 0.225802165 |
| P15531 | NDKA_HUMAN | 0.37215 | 0.162322736 |
| P28066 | PSA5_HUMAN | 0.37258 | -0.089906399 |
| Q5VT79 | AXA81_HUMAN | 0.37316 | -0.151922668 |
| O14880 | MGST3_HUMAN | 0.37362 | -0.156732519 |
| Q13596 | SNX1_HUMAN | 0.37413 | 0.200600974 |
| O43681 | ASNA_HUMAN | 0.37666 | -0.086381303 |
| P31946 | 1433B_HUMAN | 0.37854 | -0.073570523 |
| Q15365 | PCBP1_HUMAN | 0.37854 | 0.140825022 |
| P62879 | GBB2_HUMAN | 0.3796 | 0.074076215 |
| Q8N6T3 | ARFG1_HUMAN | 0.37963 | 0.167029022 |
| P53999 | TCP4_HUMAN | 0.38108 | -0.181845684 |
| P11177 | ODPB_HUMAN | 0.38141 | 0.093895243 |
| Q7KZF4 | SND1_HUMAN | 0.38269 | 0.090778628 |
| Q99832 | TCPH_HUMAN | 0.38424 | -0.142485684 |
| P05787 | K2C8_HUMAN | 0.3867 | -0.078100801 |
| Q14669 | TRIPC_HUMAN | 0.3867 | -0.427643014 |
| P18031 | PTN1_HUMAN | 0.3875 | 0.080307365 |
| P51858 | HDGF_HUMAN | 0.38801 | -0.121612301 |
| Q86VS8 | HOOK3_HUMAN | 0.38823 | -0.45757018 |
| Q96HE7 | ERO1A_HUMAN | 0.38906 | -0.090361646 |
| Q9BRF8 | CPPED_HUMAN | 0.38924 | -0.074033 |
| Q15691 | MARE1_HUMAN | 0.38966 | 0.157991801 |
| P05141 | ADT2_HUMAN | 0.38967 | 0.044712955 |
| P04114 | APOB_HUMAN | 0.39021 | -0.116035289 |
| Q96QK1 | VPS35_HUMAN | 0.39232 | 0.049972649 |
| P84095 | RHOG_HUMAN | 0.39389 | 0.04226139 |
| O60763 | USO1_HUMAN | 0.39397 | -0.029808132 |
| O15173 | PGRC2_HUMAN | 0.39523 | -0.094299958 |
| A6NCN2 | KR87P_HUMAN | 0.39523 | 0.673817646 |
| Q9UHY7 | ENOPH_HUMAN | 0.39579 | -0.606971888 |
| P08240 | SRPRA_HUMAN | 0.39613 | -0.46795322 |
| Q8WVM8 | SCFD1_HUMAN | 0.39623 | -0.066346617 |
| Q13561 | DCTN2_HUMAN | 0.39634 | 0.078330999 |
| Q9BXP5 | SRRT_HUMAN | 0.39642 | -0.177742641 |
| P07099 | HYEP_HUMAN | 0.39653 | -0.137548134 |
| E9PAV3 | NACAM_HUMAN | 0.39668 | -0.06795973 |
| Q9BPW8 | NIPS1_HUMAN | 0.39843 | -0.187232661 |
| Q9Y3D6 | FIS1_HUMAN | 0.39897 | -0.131805904 |
| Q9BV40 | VAMP8_HUMAN | 0.39907 | 0.106681668 |
| Q15717 | ELAV1_HUMAN | 0.39948 | 0.081628252 |
| P09429 | HMGB1_HUMAN | 0.40039 | -0.109961214 |
| P00338 | LDHA_HUMAN | 0.4011 | 0.057681644 |
| Q00341 | VIGLN_HUMAN | 0.40113 | 0.059250145 |
| P27708 | PYR1_HUMAN | 0.40258 | -0.268144578 |
| O75396 | SC22B_HUMAN | 0.4046 | -0.062982434 |
| Q53T59 | H1BP3_HUMAN | 0.40558 | -0.102169794 |
| P61158 | ARP3_HUMAN | 0.40561 | -0.059145957 |
| P36871 | PGM1_HUMAN | 0.40629 | 0.113549446 |
| Q9UH65 | SWP70_HUMAN | 0.40727 | -0.073304367 |
| Q00610 | CLH1_HUMAN | 0.40731 | 0.067342308 |
| O00571 | DDX3X_HUMAN | 0.40824 | -0.141539852 |
| Q9P2I0 | CPSF2_HUMAN | 0.4091 | -0.473018259 |
| P62249 | RS16_HUMAN | 0.40918 | 0.10129821 |
| Q9Y3B3 | TMED7_HUMAN | 0.40958 | 0.050931958 |
| P00558 | PGK1_HUMAN | 0.41159 | -0.044810792 |
| Q15075 | EEA1_HUMAN | 0.41214 | -0.120560362 |
| P55209 | NP1L1_HUMAN | 0.41355 | -0.082911353 |
| P62310 | LSM3_HUMAN | 0.41392 | -0.159528259 |
| Q9H0D6 | XRN2_HUMAN | 0.41508 | -0.284490735 |
| Q14103-4 | HNRPD_HUMAN | 0.4152 | 0.053759734 |
| Q13232 | NDK3_HUMAN | 0.41552 | -0.128306897 |
| P07355 | ANXA2_HUMAN | 0.41582 | -0.086085638 |
| P40429 | RL13A_HUMAN | 0.41599 | 0.083610233 |
| P55145 | MANF_HUMAN | 0.41647 | -0.185751432 |
| O60613 | SEP15_HUMAN | 0.41832 | -0.112057952 |
| Q96P70 | IPO9_HUMAN | 0.41859 | -0.578059342 |
| O60749 | SNX2_HUMAN | 0.41895 | -0.161667129 |
| P63241 | IF5A1_HUMAN | 0.42121 | -0.125134191 |
| P24752 | THIL_HUMAN | 0.42238 | -0.092795596 |
| P06744 | G6PI_HUMAN | 0.42321 | -0.122719196 |
| Q13263 | TIF1B_HUMAN | 0.42403 | 0.207765688 |
| Q9Y295 | DRG1_HUMAN | 0.42452 | -0.07938433 |
| O43920 | NDUS5_HUMAN | 0.4251 | 0.250870452 |
| P32455 | GBP1_HUMAN | 0.42618 | 0.132146687 |
| P46779 | RL28_HUMAN | 0.42733 | 0.173999131 |
| O43488 | ARK72_HUMAN | 0.42782 | -0.089547339 |
| Q08211 | DHX9_HUMAN | 0.42832 | 0.043129346 |
| O43765 | SGTA_HUMAN | 0.42848 | -0.096485052 |
| Q86YN1 | DOPP1_HUMAN | 0.42849 | -0.216269051 |
| O00232 | PSD12_HUMAN | 0.42856 | -0.108074949 |
| Q9NR45 | SIAS_HUMAN | 0.42883 | -0.069846823 |
| Q12904 | AIMP1_HUMAN | 0.42902 | -0.111648021 |
| Q99729-3 | ROAA_HUMAN | 0.42992 | -0.407584645 |
| Q92769 | HDAC2_HUMAN | 0.43148 | 0.119134614 |
| Q16795 | NDUA9_HUMAN | 0.43161 | -0.231476429 |
| P62942 | FKB1A_HUMAN | 0.43326 | -0.047940204 |
| Q9UIJ7 | KAD3_HUMAN | 0.43375 | -0.149777461 |
| P29466 | CASP1_HUMAN | 0.4343 | -0.050714321 |
| P14625 | ENPL_HUMAN | 0.43438 | -0.026547081 |
| Q70UQ0-4 | IKIP_HUMAN | 0.43487 | -0.109084482 |
| P42224 | STAT1_HUMAN | 0.43708 | 0.062927792 |
| Q6P179 | ERAP2_HUMAN | 0.43817 | 0.138520668 |
| Q8NBQ5 | DHB11_HUMAN | 0.43868 | 0.074115872 |
| P78386 | KRT85_HUMAN | 0.4392 | 0.303411227 |
| P43686 | PRS6B_HUMAN | 0.44016 | -0.177242479 |
| Q71UM5 | RS27L_HUMAN | 0.44016 | 0.087109724 |
| O00231 | PSD11_HUMAN | 0.44103 | -0.080982464 |
| P14060 | 3BHS1_HUMAN | 0.44175 | -0.133548228 |
| P62263 | RS14_HUMAN | 0.44181 | 0.067177393 |
| Q96A26 | F162A_HUMAN | 0.44291 | 0.145381184 |
| Q15181 | IPYR_HUMAN | 0.44329 | 0.06801579 |
| Q86U42 | PABP2_HUMAN | 0.44467 | 0.074713775 |
| Q8NBS9 | TXND5_HUMAN | 0.44532 | -0.144233715 |
| P07237 | PDIA1_HUMAN | 0.44663 | 0.058516442 |
| P00403 | COX2_HUMAN | 0.44703 | 0.095905964 |
| P04839 | CY24B_HUMAN | 0.44717 | -0.099746727 |
| Q99613 | EIF3C_HUMAN | 0.44767 | 0.07463288 |
| P51003 | PAPOA_HUMAN | 0.44922 | 0.389360018 |
| P30043 | BLVRB_HUMAN | 0.44952 | -0.129129283 |
| P31937 | 3HIDH_HUMAN | 0.4504 | -0.091475858 |
| P62913 | RL11_HUMAN | 0.45069 | 0.065947477 |
| Q15642 | CIP4_HUMAN | 0.45133 | -0.229424089 |
| Q07021 | C1QBP_HUMAN | 0.45201 | -0.09760267 |
| Q9Y490 | TLN1_HUMAN | 0.45295 | -0.056342614 |
| P62701 | RS4X_HUMAN | 0.45315 | -0.071501341 |
| Q9UBS4 | DJB11_HUMAN | 0.45316 | -0.235026008 |
| P23528 | COF1_HUMAN | 0.45413 | -0.057941843 |
| P45974 | UBP5_HUMAN | 0.45416 | 0.073757716 |
| P40306 | PSB10_HUMAN | 0.45481 | -0.076216059 |
| Q9Y5Z4 | HEBP2_HUMAN | 0.45482 | -0.15629877 |
| P19404 | NDUV2_HUMAN | 0.45505 | -0.142083639 |
| P60033 | CD81_HUMAN | 0.45553 | 0.201233852 |
| P14314 | GLU2B_HUMAN | 0.45575 | -0.061080551 |
| P61163 | ACTZ_HUMAN | 0.45756 | -0.200177074 |
| P38571 | LICH_HUMAN | 0.45765 | -0.127404424 |
| Q8N423 | LIRB2_HUMAN | 0.45829 | 0.195953212 |
| Q13155 | AIMP2_HUMAN | 0.45836 | -0.175580786 |
| Q7Z3J2 | VP35L_HUMAN | 0.45845 | -0.139192073 |
| Q13423 | NNTM_HUMAN | 0.45936 | 0.046562226 |
| P30101 | PDIA3_HUMAN | 0.45969 | -0.142120934 |
| P41240 | CSK_HUMAN | 0.46068 | 0.084183531 |
| O75844 | FACE1_HUMAN | 0.46253 | -0.0957761 |
| Q9Y2R0 | COA3_HUMAN | 0.46261 | -0.113248628 |
| P14618 | KPYM_HUMAN | 0.46534 | 0.04710442 |
| P52209 | 6PGD_HUMAN | 0.46557 | 0.056895064 |
| P51571 | SSRD_HUMAN | 0.46586 | -0.088985208 |
| P30740 | ILEU_HUMAN | 0.46611 | 0.089774279 |
| P47985 | UCRI_HUMAN | 0.46682 | -0.103807692 |
| Q9Y4K1 | CRBG1_HUMAN | 0.46691 | 0.152913662 |
| O43592 | XPOT_HUMAN | 0.46745 | -0.166998564 |
| O43790 | KRT86_HUMAN | 0.46748 | 0.197947802 |
| P29401 | TKT_HUMAN | 0.46774 | 0.039058903 |
| Q6NUK1 | SCMC1_HUMAN | 0.46778 | -0.139621173 |
| P05556 | ITB1_HUMAN | 0.4681 | -0.106247707 |
| Q13045 | FLII_HUMAN | 0.46839 | -0.076090162 |
| Q92556 | ELMO1_HUMAN | 0.46874 | 0.139077818 |
| O00400 | ACATN_HUMAN | 0.47019 | 0.334648676 |
| Q7Z7H5 | TMED4_HUMAN | 0.47056 | -0.082040833 |
| Q15323 | K1H1_HUMAN | 0.47127 | 0.40046138 |
| Q9Y584 | TIM22_HUMAN | 0.47157 | 0.21519382 |
| Q13177 | PAK2_HUMAN | 0.47314 | -0.078986016 |
| P23368 | MAOM_HUMAN | 0.47323 | 0.092770887 |
| Q9NWV8 | BABA1_HUMAN | 0.47374 | -0.161709677 |
| P55786 | PSA_HUMAN | 0.47448 | -0.09373757 |
| Q9NT62 | ATG3_HUMAN | 0.47504 | -0.123429937 |
| Q99497 | PARK7_HUMAN | 0.47642 | -0.033467357 |
| P19105 | ML12A_HUMAN | 0.47698 | -0.05149755 |
| Q8NCN5 | PDPR_HUMAN | 0.47723 | -0.159490631 |
| P22234 | PUR6_HUMAN | 0.47787 | 0.101121293 |
| Q02878 | RL6_HUMAN | 0.47921 | 0.093967166 |
| Q9Y276 | BCS1_HUMAN | 0.47924 | -0.156720703 |
| P17661 | DESM_HUMAN | 0.48017 | 0.090525665 |
| Q31610 | 1B81_HUMAN | 0.48252 | -0.112557025 |
| P09104 | ENOG_HUMAN | 0.48263 | -0.09170647 |
| P04229 | 2B11_HUMAN | 0.4835 | -0.176840986 |
| Q93050 | VPP1_HUMAN | 0.48366 | 0.129203223 |
| P57088 | TMM33_HUMAN | 0.48408 | 0.363414035 |
| Q13347 | EIF3I_HUMAN | 0.48441 | 0.058348769 |
| Q00169 | PIPNA_HUMAN | 0.48514 | -0.117725931 |
| P61604 | CH10_HUMAN | 0.48518 | -0.085016021 |
| P01040 | CYTA_HUMAN | 0.48594 | 0.119498817 |
| Q9NZL9 | MAT2B_HUMAN | 0.48612 | 0.11260391 |
| Q9NR28 | DBLOH_HUMAN | 0.48668 | 0.054867225 |
| O00754 | MA2B1_HUMAN | 0.48877 | 0.245664071 |
| Q9BT78 | CSN4_HUMAN | 0.48975 | 0.085107508 |
| Q8TBQ9 | KISHA_HUMAN | 0.48988 | -0.229174372 |
| P11169 | GTR3_HUMAN | 0.49086 | -0.072735068 |
| P11021 | BIP_HUMAN | 0.49102 | -0.029223102 |
| Q9NX63 | MIC19_HUMAN | 0.49284 | 0.096687621 |
| Q7L1Q6-2 | BZW1_HUMAN | 0.49304 | -0.193776756 |
| Q14974 | IMB1_HUMAN | 0.49338 | 0.028187893 |
| P68871 | HBB_HUMAN | 0.49349 | -0.165891075 |
| Q16563-2 | SYPL1_HUMAN | 0.49455 | -0.113948144 |
| Q9Y6G9 | DC1L1_HUMAN | 0.49526 | 0.116544621 |
| P51991 | ROA3_HUMAN | 0.49587 | -0.077432449 |
| Q9UFN0 | NPS3A_HUMAN | 0.49593 | -0.1045425 |
| P63173 | RL38_HUMAN | 0.49629 | 0.082164714 |
| Q9HCU5 | PREB_HUMAN | 0.49634 | 0.165072497 |
| Q08380 | LG3BP_HUMAN | 0.49747 | -0.046878481 |
| Q9BVK6 | TMED9_HUMAN | 0.49896 | 0.067972336 |
| Q9NSD9 | SYFB_HUMAN | 0.49945 | 0.169444833 |
| P46108 | CRK_HUMAN | 0.49962 | -0.28947818 |
| Q8NBJ5 | GT251_HUMAN | 0.50176 | 0.118397919 |
| P55036 | PSMD4_HUMAN | 0.50225 | -0.105995616 |
| Q9Y241 | HIG1A_HUMAN | 0.5023 | -0.316614883 |
| P00352 | AL1A1_HUMAN | 0.50303 | 0.14148079 |
| Q08752 | PPID_HUMAN | 0.50315 | -0.076842746 |
| P61088 | UBE2N_HUMAN | 0.50327 | -0.036586395 |
| P02794 | FRIH_HUMAN | 0.50344 | -0.054763825 |
| P13647 | K2C5_HUMAN | 0.5055 | -0.274029696 |
| O60488 | ACSL4_HUMAN | 0.50562 | -0.071550361 |
| P50914 | RL14_HUMAN | 0.50586 | 0.070071852 |
| Q99436 | PSB7_HUMAN | 0.50656 | 0.051919491 |
| P59998 | ARPC4_HUMAN | 0.50719 | 0.033488069 |
| Q04760 | LGUL_HUMAN | 0.5081 | -0.315982386 |
| P62495 | ERF1_HUMAN | 0.50839 | 0.125702469 |
| Q9BRA2 | TXD17_HUMAN | 0.50867 | 0.072768408 |
| Q9UID3 | VPS51_HUMAN | 0.50867 | -0.254993425 |
| P78417 | GSTO1_HUMAN | 0.5087 | -0.11768188 |
| Q8N0X7 | SPART_HUMAN | 0.50976 | -0.066760339 |
| O60884 | DNJA2_HUMAN | 0.51042 | 0.092648176 |
| P21912 | SDHB_HUMAN | 0.51049 | -0.131106049 |
| O43676 | NDUB3_HUMAN | 0.51293 | 0.112343651 |
| P62241 | RS8_HUMAN | 0.51468 | 0.077493514 |
| P19525 | E2AK2_HUMAN | 0.51487 | -0.132445649 |
| O75688 | PPM1B_HUMAN | 0.51496 | -0.136324166 |
| Q9P0S9 | TM14C_HUMAN | 0.51634 | -0.239900588 |
| Q9UMY4-2 | SNX12_HUMAN | 0.51718 | 0.056172113 |
| Q8TCT9 | HM13_HUMAN | 0.51747 | -0.195865487 |
| Q92734 | TFG_HUMAN | 0.51752 | -0.123163946 |
| Q9H223 | EHD4_HUMAN | 0.51853 | -0.060946704 |
| Q9NVJ2 | ARL8B_HUMAN | 0.51861 | -0.093158625 |
| Q9HDC9 | APMAP_HUMAN | 0.51865 | 0.096383183 |
| P12277 | KCRB_HUMAN | 0.51967 | 0.122128734 |
| Q5JTV8 | TOIP1_HUMAN | 0.52012 | -0.126014765 |
| Q01105 | SET_HUMAN | 0.5207 | -0.048971278 |
| O15260 | SURF4_HUMAN | 0.52113 | -0.116090255 |
| O95340 | PAPS2_HUMAN | 0.52128 | 0.178157277 |
| Q9BUL8 | PDC10_HUMAN | 0.52146 | -0.063853308 |
| Q9H2U2 | IPYR2_HUMAN | 0.52282 | -0.052697979 |
| P21964 | COMT_HUMAN | 0.52479 | -0.105753431 |
| Q06210 | GFPT1_HUMAN | 0.52512 | 0.115654401 |
| Q9Y3B4 | SF3B6_HUMAN | 0.52544 | -0.102718744 |
| O75369-8 | FLNB_HUMAN | 0.52557 | 0.112861439 |
| Q9NX40 | OCAD1_HUMAN | 0.52572 | -0.175197671 |
| Q9UKK9 | NUDT5_HUMAN | 0.52595 | -0.07648147 |
| O75165 | DJC13_HUMAN | 0.52618 | 0.101622791 |
| P24557 | THAS_HUMAN | 0.52633 | -0.138451999 |
| Q9UJ70-2 | NAGK_HUMAN | 0.52645 | -0.073916752 |
| P14317 | HCLS1_HUMAN | 0.52679 | -0.097531576 |
| O00764 | PDXK_HUMAN | 0.52718 | -0.062307501 |
| Q15008 | PSMD6_HUMAN | 0.52726 | -0.115898882 |
| Q9UNM6 | PSD13_HUMAN | 0.52764 | 0.179467352 |
| P61019 | RAB2A_HUMAN | 0.52773 | 0.028505523 |
| Q9UNZ2 | NSF1C_HUMAN | 0.52929 | -0.123195652 |
| Q9H4A4 | AMPB_HUMAN | 0.52933 | -0.080344457 |
| P54727 | RD23B_HUMAN | 0.52937 | 0.081934027 |
| Q7L5L3 | GDPD3_HUMAN | 0.52949 | -0.102310568 |
| P43490 | NAMPT_HUMAN | 0.52988 | -0.058656021 |
| P26641 | EF1G_HUMAN | 0.53092 | 0.028656153 |
| Q96I99 | SUCB2_HUMAN | 0.53311 | -0.07634273 |
| Q9H3N1 | TMX1_HUMAN | 0.53347 | -0.034827411 |
| Q8TD19 | NEK9_HUMAN | 0.53429 | -0.089466708 |
| Q6P2Q9 | PRP8_HUMAN | 0.53454 | 0.111007901 |
| P61916 | NPC2_HUMAN | 0.53479 | 0.093222088 |
| P02751 | FINC_HUMAN | 0.5356 | -0.089902511 |
| P04632 | CPNS1_HUMAN | 0.53813 | -0.075970223 |
| Q96T76-8 | MMS19_HUMAN | 0.53825 | -0.322101571 |
| P09525 | ANXA4_HUMAN | 0.53881 | -0.109911435 |
| Q9BQA1 | MEP50_HUMAN | 0.54327 | 0.127486378 |
| Q92643 | GPI8_HUMAN | 0.54364 | -0.129564064 |
| Q9BXS5 | AP1M1_HUMAN | 0.54631 | 0.055490453 |
| P61160 | ARP2_HUMAN | 0.54646 | 0.071258385 |
| P09382 | LEG1_HUMAN | 0.54718 | -0.098924437 |
| P07602 | SAP_HUMAN | 0.54942 | -0.065980223 |
| P35908 | K22E_HUMAN | 0.55016 | -0.249115218 |
| Q92900 | RENT1_HUMAN | 0.5508 | 0.090655223 |
| P61086 | UBE2K_HUMAN | 0.55139 | 0.107992246 |
| Q9BWM7 | SFXN3_HUMAN | 0.55157 | 0.064836645 |
| Q9BSJ8 | ESYT1_HUMAN | 0.55164 | -0.079148038 |
| O15371 | EIF3D_HUMAN | 0.55255 | 0.08813701 |
| P53396 | ACLY_HUMAN | 0.55378 | 0.032779402 |
| P62805 | H4_HUMAN | 0.55384 | 0.103000396 |
| P51692 | STA5B_HUMAN | 0.55763 | 0.108412475 |
| Q15366 | PCBP2_HUMAN | 0.55841 | 0.10966739 |
| P25787 | PSA2_HUMAN | 0.55845 | -0.048180215 |
| P16401 | H15_HUMAN | 0.55943 | 0.107306159 |
| Q07960 | RHG01_HUMAN | 0.5597 | -0.056528184 |
| Q9P2J5 | SYLC_HUMAN | 0.56003 | 0.13273719 |
| P33897 | ABCD1_HUMAN | 0.56085 | -0.074261038 |
| O76009 | KT33A_HUMAN | 0.56117 | 0.210200759 |
| Q96A72 | MGN2_HUMAN | 0.56188 | -0.13075602 |
| Q5VYY1 | ANR22_HUMAN | 0.5625 | 0.123310338 |
| Q14203 | DCTN1_HUMAN | 0.56289 | -0.058732691 |
| Q00765 | REEP5_HUMAN | 0.56365 | 0.061499203 |
| Q9HBL7 | PLRKT_HUMAN | 0.5643 | -0.234320506 |
| Q7Z3C6 | ATG9A_HUMAN | 0.56556 | 0.143139828 |
| P15529 | MCP_HUMAN | 0.56608 | 0.178846439 |
| Q05655 | KPCD_HUMAN | 0.57002 | 0.270188946 |
| P62269 | RS18_HUMAN | 0.57048 | -0.051160099 |
| P35221 | CTNA1_HUMAN | 0.57285 | -0.104295715 |
| P37802 | TAGL2_HUMAN | 0.57286 | -0.076140708 |
| P43034 | LIS1_HUMAN | 0.5733 | 0.060953453 |
| P62280 | RS11_HUMAN | 0.57418 | 0.072295936 |
| P00492 | HPRT_HUMAN | 0.57444 | -0.15239926 |
| Q86UE4 | LYRIC_HUMAN | 0.57639 | 0.116932629 |
| O15162 | PLS1_HUMAN | 0.57721 | 0.151154452 |
| P21397 | AOFA_HUMAN | 0.57857 | -0.087872439 |
| P40121 | CAPG_HUMAN | 0.57915 | -0.058461063 |
| Q8N1F7 | NUP93_HUMAN | 0.5792 | 0.050250201 |
| P19338 | NUCL_HUMAN | 0.57949 | 0.03579545 |
| Q15149-4 | PLEC_HUMAN | 0.57987 | 0.106150952 |
| Q96RQ9 | OXLA_HUMAN | 0.58001 | -0.063293027 |
| P11215 | ITAM_HUMAN | 0.58026 | 0.075659195 |
| O14980 | XPO1_HUMAN | 0.58322 | -0.171671976 |
| P21796 | VDAC1_HUMAN | 0.58325 | 0.075463441 |
| P04264 | K2C1_HUMAN | 0.5837 | -0.250013756 |
| P07305 | H10_HUMAN | 0.5857 | 0.120008328 |
| Q9Y305 | ACOT9_HUMAN | 0.58725 | -0.165975515 |
| O75608 | LYPA1_HUMAN | 0.58737 | 0.082120887 |
| Q99538 | LGMN_HUMAN | 0.58762 | -0.108859991 |
| Q9Y266 | NUDC_HUMAN | 0.58766 | -0.054697159 |
| P54687 | BCAT1_HUMAN | 0.58814 | -0.081029534 |
| P22626 | ROA2_HUMAN | 0.58822 | 0.090163408 |
| P07203 | GPX1_HUMAN | 0.58986 | 0.056667068 |
| Q5EBM0 | CMPK2_HUMAN | 0.59218 | 0.082384272 |
| Q9NTX5 | ECHD1_HUMAN | 0.59443 | -0.058683463 |
| Q9Y6M9 | NDUB9_HUMAN | 0.59482 | -0.108953474 |
| Q71UI9 | H2AV_HUMAN | 0.59622 | 0.06816417 |
| Q92598 | HS105_HUMAN | 0.59625 | 0.048925542 |
| P61009 | SPCS3_HUMAN | 0.5973 | -0.05402335 |
| P99999 | CYC_HUMAN | 0.59845 | 0.028593203 |
| P61970 | NTF2_HUMAN | 0.59862 | -0.073291369 |
| P29218 | IMPA1_HUMAN | 0.60001 | -0.0624869 |
| P51812 | KS6A3_HUMAN | 0.60366 | 0.083319622 |
| P13073 | COX41_HUMAN | 0.60379 | 0.084604015 |
| P98082 | DAB2_HUMAN | 0.60596 | -0.115204704 |
| P13804 | ETFA_HUMAN | 0.60654 | -0.081942514 |
| Q9Y3E0 | GOT1B_HUMAN | 0.60673 | -0.218028156 |
| Q9NVD7-2 | PARVA_HUMAN | 0.60728 | -0.11191263 |
| P62888 | RL30_HUMAN | 0.60755 | 0.03028992 |
| P16930 | FAAA_HUMAN | 0.60796 | 0.07301251 |
| P13667 | PDIA4_HUMAN | 0.60831 | -0.033248452 |
| P55957 | BID_HUMAN | 0.60881 | -0.214813151 |
| P06396-2 | GELS_HUMAN | 0.61004 | -0.026197591 |
| O60762 | DPM1_HUMAN | 0.61128 | 0.114201805 |
| P39023 | RL3_HUMAN | 0.61249 | 0.137601501 |
| Q13642 | FHL1_HUMAN | 0.61266 | 0.216282157 |
| Q9Y3F4 | STRAP_HUMAN | 0.61482 | 0.115636878 |
| Q6PIU2 | NCEH1_HUMAN | 0.61532 | 0.040777261 |
| O75390 | CISY_HUMAN | 0.61629 | -0.032718487 |
| P00387-3 | NB5R3_HUMAN | 0.61759 | 0.056041257 |
| P06753-2 | TPM3_HUMAN | 0.61771 | -0.038982841 |
| P31943 | HNRH1_HUMAN | 0.61872 | 0.051803405 |
| O95298 | NDUC2_HUMAN | 0.61964 | 0.147919801 |
| Q9Y2Q3 | GSTK1_HUMAN | 0.61987 | 0.02997386 |
| Q14444 | CAPR1_HUMAN | 0.62037 | -0.040767418 |
| O75643 | U520_HUMAN | 0.62141 | 0.080981386 |
| P50452 | SPB8_HUMAN | 0.62144 | -0.097850445 |
| Q13740 | CD166_HUMAN | 0.62435 | 0.112439274 |
| P41218 | MNDA_HUMAN | 0.6258 | -0.052726342 |
| P28074 | PSB5_HUMAN | 0.6259 | 0.078130369 |
| Q96BN8 | OTUL_HUMAN | 0.62679 | -0.191109956 |
| P08133 | ANXA6_HUMAN | 0.62706 | 0.057394728 |
| P13498 | CY24A_HUMAN | 0.62709 | 0.099876876 |
| O00160 | MYO1F_HUMAN | 0.62843 | -0.07077974 |
| P62316 | SMD2_HUMAN | 0.62975 | 0.042136245 |
| Q96CX2 | KCD12_HUMAN | 0.62981 | -0.037696743 |
| Q9NZ01 | TECR_HUMAN | 0.62996 | -0.115249827 |
| P08729 | K2C7_HUMAN | 0.63006 | -0.057047935 |
| Q9H8H3 | MET7A_HUMAN | 0.63009 | -0.070900519 |
| Q9UNH7 | SNX6_HUMAN | 0.63041 | -0.049790526 |
| P53597 | SUCA_HUMAN | 0.6314 | -0.060427282 |
| P40926 | MDHM_HUMAN | 0.63237 | -0.058429111 |
| Q16799 | RTN1_HUMAN | 0.63333 | -0.071737934 |
| P49368 | TCPG_HUMAN | 0.63335 | -0.029182661 |
| P13716 | HEM2_HUMAN | 0.63488 | 0.051410936 |
| P55008 | AIF1_HUMAN | 0.63545 | 0.077583985 |
| P05121 | PAI1_HUMAN | 0.63631 | 0.089163979 |
| Q1KMD3 | HNRL2_HUMAN | 0.63717 | 0.064130127 |
| O94856 | NFASC_HUMAN | 0.63836 | 0.234096198 |
| P02545 | LMNA_HUMAN | 0.63936 | 0.046584832 |
| O75083 | WDR1_HUMAN | 0.64087 | 0.029774471 |
| O75323 | NIPS2_HUMAN | 0.64108 | 0.075435159 |
| Q02978 | M2OM_HUMAN | 0.64113 | -0.107525466 |
| Q99426 | TBCB_HUMAN | 0.64135 | 0.086669413 |
| P05362 | ICAM1_HUMAN | 0.64233 | -0.039315618 |
| P13797-2 | PLST_HUMAN | 0.64291 | -0.077612196 |
| P55265 | DSRAD_HUMAN | 0.64553 | 0.060847991 |
| P13674-3 | P4HA1_HUMAN | 0.64565 | 0.068361217 |
| P04844 | RPN2_HUMAN | 0.64574 | 0.02862975 |
| Q9Y371-2 | SHLB1_HUMAN | 0.64615 | 0.080768669 |
| P10809 | CH60_HUMAN | 0.64652 | 0.047885128 |
| Q68EM7 | RHG17_HUMAN | 0.64741 | -0.078131981 |
| P16219 | ACADS_HUMAN | 0.64906 | 0.105765991 |
| P09455 | RET1_HUMAN | 0.65012 | -0.038154157 |
| P62258 | 1433E_HUMAN | 0.65027 | 0.0201515 |
| P09669 | COX6C_HUMAN | 0.65201 | 0.049967082 |
| Q05682 | CALD1_HUMAN | 0.65268 | 0.079214788 |
| Q16629 | SRSF7_HUMAN | 0.65317 | -0.065288369 |
| Q8NE71 | ABCF1_HUMAN | 0.65407 | 0.04189039 |
| P09960 | LKHA4_HUMAN | 0.65489 | -0.070413176 |
| P07814 | SYEP_HUMAN | 0.65581 | 0.070597312 |
| Q16134 | ETFD_HUMAN | 0.65585 | 0.043189118 |
| Q8N163 | CCAR2_HUMAN | 0.65634 | 0.058236612 |
| P31949 | S10AB_HUMAN | 0.65788 | -0.065425936 |
| Q8N6L1 | KTAP2_HUMAN | 0.65805 | 0.043537054 |
| Q8TCT8 | SPP2A_HUMAN | 0.65817 | 0.068103395 |
| P46977 | STT3A_HUMAN | 0.65842 | 0.070341996 |
| P15170-3 | ERF3A_HUMAN | 0.65842 | 0.051554828 |
| Q99735 | MGST2_HUMAN | 0.65858 | 0.071767489 |
| Q8N0U8 | VKORL_HUMAN | 0.65874 | -0.160168041 |
| Q13185 | CBX3_HUMAN | 0.65881 | -0.04768075 |
| Q99536 | VAT1_HUMAN | 0.65909 | -0.047859296 |
| P04062 | GLCM_HUMAN | 0.6592 | -0.062449048 |
| Q8IUE6 | H2A2B_HUMAN | 0.65924 | 0.060667425 |
| Q96C19 | EFHD2_HUMAN | 0.65925 | -0.033735641 |
| Q13838-2 | DX39B_HUMAN | 0.65987 | 0.0552125 |
| Q14764 | MVP_HUMAN | 0.65996 | 0.050081176 |
| P55039 | DRG2_HUMAN | 0.66087 | 0.098438604 |
| P80217 | IN35_HUMAN | 0.66104 | -0.058617509 |
| Q96AE4 | FUBP1_HUMAN | 0.66147 | -0.026207148 |
| P08246 | ELNE_HUMAN | 0.66231 | -0.073078339 |
| P26373 | RL13_HUMAN | 0.66402 | 0.069944575 |
| Q15388 | TOM20_HUMAN | 0.66488 | 0.09028225 |
| O94973 | AP2A2_HUMAN | 0.66566 | -0.123270928 |
| P53618 | COPB_HUMAN | 0.66596 | -0.083604406 |
| O94905 | ERLN2_HUMAN | 0.6665 | 0.029344799 |
| P20340-2 | RAB6A_HUMAN | 0.66697 | -0.076140943 |
| Q15836 | VAMP3_HUMAN | 0.66849 | 0.025914662 |
| O95831 | AIFM1_HUMAN | 0.67025 | -0.064832421 |
| O00217 | NDUS8_HUMAN | 0.67169 | -0.102613556 |
| O96019 | ACL6A_HUMAN | 0.6734 | 0.035233146 |
| Q6PI78 | TMM65_HUMAN | 0.67362 | -0.118217411 |
| Q9Y333 | LSM2_HUMAN | 0.67371 | 0.070902072 |
| P59666 | DEF3_HUMAN | 0.67444 | -0.120171355 |
| P33121 | ACSL1_HUMAN | 0.67459 | -0.064242736 |
| P38919 | IF4A3_HUMAN | 0.67507 | -0.047297326 |
| Q14344 | GNA13_HUMAN | 0.67657 | 0.067950759 |
| P53634 | CATC_HUMAN | 0.67705 | -0.044324492 |
| Q9H9B4 | SFXN1_HUMAN | 0.67766 | 0.044899944 |
| P50991 | TCPD_HUMAN | 0.67804 | -0.043702972 |
| P12429 | ANXA3_HUMAN | 0.67807 | 0.070797024 |
| Q9BQB6 | VKOR1_HUMAN | 0.68028 | -0.101923307 |
| Q15459 | SF3A1_HUMAN | 0.68149 | 0.040939242 |
| P22695 | QCR2_HUMAN | 0.68158 | 0.064186038 |
| P61981 | 1433G_HUMAN | 0.68175 | 0.028243753 |
| O75367 | H2AY_HUMAN | 0.68374 | 0.09361383 |
| P07910 | HNRPC_HUMAN | 0.68379 | 0.138460196 |
| P20339 | RAB5A_HUMAN | 0.68501 | 0.045688862 |
| P47755 | CAZA2_HUMAN | 0.68543 | -0.059438294 |
| Q5TEJ8 | THMS2_HUMAN | 0.68604 | 0.12163401 |
| P61224 | RAP1B_HUMAN | 0.68621 | -0.072475112 |
| O60716 | CTND1_HUMAN | 0.68627 | -0.095945457 |
| Q96AC1-3 | FERM2_HUMAN | 0.68692 | -0.058945734 |
| P40925 | MDHC_HUMAN | 0.6876 | -0.019548111 |
| P60953 | CDC42_HUMAN | 0.68828 | 0.030705606 |
| Q12797 | ASPH_HUMAN | 0.68838 | -0.060901358 |
| P48059-5 | LIMS1_HUMAN | 0.68849 | -0.097623346 |
| Q15738 | NSDHL_HUMAN | 0.6896 | 0.095994162 |
| Q14108 | SCRB2_HUMAN | 0.68983 | 0.037795276 |
| P52788 | SPSY_HUMAN | 0.68986 | 0.061178689 |
| P20591 | MX1_HUMAN | 0.6905 | 0.070468063 |
| P67870 | CSK2B_HUMAN | 0.69064 | 0.075487391 |
| Q96FQ6 | S10AG_HUMAN | 0.69109 | -0.080630535 |
| P23381 | SYWC_HUMAN | 0.69193 | 0.047622269 |
| Q02543 | RL18A_HUMAN | 0.69268 | 0.061882213 |
| P42167 | LAP2B_HUMAN | 0.69279 | 0.036468765 |
| P52907 | CAZA1_HUMAN | 0.69337 | -0.043133929 |
| Q15274 | NADC_HUMAN | 0.69418 | -0.130741854 |
| P05120 | PAI2_HUMAN | 0.69491 | -0.06552559 |
| P60709 | ACTB_HUMAN | 0.69533 | 0.025507852 |
| Q9NRV9 | HEBP1_HUMAN | 0.69552 | -0.048664088 |
| Q6EEV6 | SUMO4_HUMAN | 0.69731 | -0.041105731 |
| Q9UIQ6 | LCAP_HUMAN | 0.69745 | 0.07450017 |
| P61106 | RAB14_HUMAN | 0.6991 | 0.070585162 |
| Q9NQ88 | TIGAR_HUMAN | 0.70069 | 0.05005371 |
| P02786 | TFR1_HUMAN | 0.70307 | 0.054478818 |
| P63172 | DYLT1_HUMAN | 0.70355 | -0.052168238 |
| Q04941 | PLP2_HUMAN | 0.70377 | 0.07619352 |
| P47897 | SYQ_HUMAN | 0.70759 | 0.07698036 |
| P61077 | UB2D3_HUMAN | 0.708 | -0.102406992 |
| O75116 | ROCK2_HUMAN | 0.70974 | 0.044762422 |
| Q13617 | CUL2_HUMAN | 0.71023 | -0.106231109 |
| Q96G03 | PGM2_HUMAN | 0.71025 | 0.029324063 |
| Q02218 | ODO1_HUMAN | 0.71031 | 0.047679411 |
| Q9NVI7 | ATD3A_HUMAN | 0.71111 | -0.101819024 |
| P33947 | ERD22_HUMAN | 0.71173 | 0.084049465 |
| P02538 | K2C6A_HUMAN | 0.71256 | 0.07430158 |
| Q16698 | DECR_HUMAN | 0.71281 | -0.047635289 |
| P14866 | HNRPL_HUMAN | 0.71335 | 0.040303202 |
| Q16851 | UGPA_HUMAN | 0.71361 | -0.042713841 |
| O00186 | STXB3_HUMAN | 0.7139 | -0.066993988 |
| Q16666-3 | IF16_HUMAN | 0.71478 | 0.038590135 |
| O43914 | TYOBP_HUMAN | 0.71538 | -0.057511562 |
| Q9NZT2 | OGFR_HUMAN | 0.71631 | -0.068248881 |
| P02533 | K1C14_HUMAN | 0.71739 | -0.098838896 |
| P12532 | KCRU_HUMAN | 0.71744 | 0.064727775 |
| P51452 | DUS3_HUMAN | 0.71765 | 0.047826003 |
| P10620 | MGST1_HUMAN | 0.72023 | -0.068681541 |
| O14818 | PSA7_HUMAN | 0.72117 | 0.020774913 |
| Q969H8 | MYDGF_HUMAN | 0.72169 | 0.042557352 |
| P80297 | MT1X_HUMAN | 0.72195 | -0.077256014 |
| Q15369 | ELOC_HUMAN | 0.72265 | 0.046660531 |
| Q13576 | IQGA2_HUMAN | 0.72283 | 0.037388275 |
| P11216 | PYGB_HUMAN | 0.72289 | -0.032544717 |
| P04406 | G3P_HUMAN | 0.723 | -0.021394164 |
| Q71U36 | TBA1A_HUMAN | 0.72329 | -0.147006909 |
| Q9HD20 | AT131_HUMAN | 0.72359 | -0.050197689 |
| P43307 | SSRA_HUMAN | 0.7249 | -0.035059203 |
| P56199 | ITA1_HUMAN | 0.72534 | 0.080877116 |
| P20936 | RASA1_HUMAN | 0.72555 | 0.093690259 |
| P62244 | RS15A_HUMAN | 0.72614 | -0.032222135 |
| Q06323 | PSME1_HUMAN | 0.72616 | -0.062514036 |
| P08134 | RHOC_HUMAN | 0.72627 | -0.047306055 |
| Q93008 | USP9X_HUMAN | 0.72637 | 0.036371837 |
| P05106 | ITB3_HUMAN | 0.72672 | 0.051579364 |
| P08473 | NEP_HUMAN | 0.72716 | 0.070660162 |
| Q9UL25 | RAB21_HUMAN | 0.72912 | 0.023132677 |
| O43324 | MCA3_HUMAN | 0.72933 | 0.039549127 |
| Q9NSA0 | S22AB_HUMAN | 0.72951 | -0.101725792 |
| Q99873 | ANM1_HUMAN | 0.73148 | 0.052968958 |
| Q9BZQ8 | NIBAN_HUMAN | 0.73174 | -0.031489 |
| Q5JWF2 | GNAS1_HUMAN | 0.73194 | 0.046677399 |
| Q14019 | COTL1_HUMAN | 0.73249 | -0.016911024 |
| Q9UNL2 | SSRG_HUMAN | 0.73286 | 0.043028111 |
| P06730-2 | IF4E_HUMAN | 0.73343 | -0.032081099 |
| P26639 | SYTC_HUMAN | 0.73352 | -0.020764254 |
| P13928 | ANXA8_HUMAN | 0.7339 | -0.069643035 |
| P78537 | BL1S1_HUMAN | 0.73393 | -0.065290702 |
| P11586 | C1TC_HUMAN | 0.73413 | 0.062767437 |
| P31930 | QCR1_HUMAN | 0.73602 | 0.034306278 |
| P16615 | AT2A2_HUMAN | 0.73731 | -0.027429621 |
| O15144 | ARPC2_HUMAN | 0.73817 | 0.034922255 |
| O14737 | PDCD5_HUMAN | 0.73821 | -0.046695341 |
| Q99460 | PSMD1_HUMAN | 0.73825 | -0.031925514 |
| P16083 | NQO2_HUMAN | 0.73825 | 0.039893088 |
| O60234 | GMFG_HUMAN | 0.73827 | -0.031020115 |
| Q13571 | LAPM5_HUMAN | 0.73986 | 0.099180698 |
| P05023 | AT1A1_HUMAN | 0.73991 | -0.045543885 |
| P04233-2 | HG2A_HUMAN | 0.7408 | -0.063393088 |
| P08648 | ITA5_HUMAN | 0.74233 | -0.047293667 |
| O94826 | TOM70_HUMAN | 0.74382 | -0.074708504 |
| Q9NSE4 | SYIM_HUMAN | 0.74406 | -0.050576618 |
| P51159 | RB27A_HUMAN | 0.74517 | 0.036381222 |
| P60981 | DEST_HUMAN | 0.74534 | 0.097055851 |
| P49748 | ACADV_HUMAN | 0.74605 | -0.059700923 |
| P61978 | HNRPK_HUMAN | 0.74652 | -0.055854379 |
| Q15041 | AR6P1_HUMAN | 0.7471 | -0.050490813 |
| P25788 | PSA3_HUMAN | 0.74858 | -0.027990834 |
| Q15833 | STXB2_HUMAN | 0.75098 | 0.022446836 |
| Q96KP4 | CNDP2_HUMAN | 0.75236 | 0.020847704 |
| P20645 | MPRD_HUMAN | 0.75245 | 0.043872301 |
| P18669 | PGAM1_HUMAN | 0.75339 | 0.025196251 |
| Q9Y3C8 | UFC1_HUMAN | 0.75616 | 0.039280111 |
| P16144 | ITB4_HUMAN | 0.75715 | 0.069437736 |
| Q4V328 | GRAP1_HUMAN | 0.75814 | 0.042760517 |
| P07437 | TBB5_HUMAN | 0.7584 | -0.052198786 |
| P35613-2 | BASI_HUMAN | 0.75883 | 0.016902215 |
| P49327 | FAS_HUMAN | 0.75949 | 0.044435047 |
| Q99714 | HCD2_HUMAN | 0.76027 | 0.023716121 |
| Q7RTV0 | PHF5A_HUMAN | 0.7634 | -0.049369937 |
| Q16891 | MIC60_HUMAN | 0.76405 | 0.111533717 |
| P60983 | GMFB_HUMAN | 0.76406 | -0.043089022 |
| P25815 | S100P_HUMAN | 0.76415 | -0.076091964 |
| P28482 | MK01_HUMAN | 0.76441 | -0.028330163 |
| Q7LG56 | RIR2B_HUMAN | 0.76717 | 0.105980006 |
| Q00577 | PURA_HUMAN | 0.76956 | -0.049214179 |
| Q00653 | NFKB2_HUMAN | 0.77169 | 0.06919494 |
| Q96TC7 | RMD3_HUMAN | 0.77338 | -0.074075009 |
| P62330 | ARF6_HUMAN | 0.77443 | -0.028532982 |
| Q09666 | AHNK_HUMAN | 0.775 | -0.032925269 |
| O95433 | AHSA1_HUMAN | 0.77658 | -0.027405424 |
| Q08257 | QOR_HUMAN | 0.77747 | 0.047316602 |
| P25786 | PSA1_HUMAN | 0.77776 | 0.046008423 |
| P36776 | LONM_HUMAN | 0.77811 | 0.03101597 |
| P48444 | COPD_HUMAN | 0.77822 | 0.048665318 |
| P61247 | RS3A_HUMAN | 0.78103 | 0.044719588 |
| Q15661 | TRYB1_HUMAN | 0.78371 | -0.046368606 |
| O15145 | ARPC3_HUMAN | 0.78437 | -0.01501531 |
| Q9Y678 | COPG1_HUMAN | 0.78449 | 0.016883021 |
| Q13616 | CUL1_HUMAN | 0.78454 | -0.076051771 |
| Q9NX46 | ARHL2_HUMAN | 0.78754 | -0.019515303 |
| P11310 | ACADM_HUMAN | 0.78876 | -0.046780995 |
| Q9NUV9 | GIMA4_HUMAN | 0.78975 | 0.026010086 |
| Q10567 | AP1B1_HUMAN | 0.79055 | -0.053322403 |
| O96008 | TOM40_HUMAN | 0.79164 | -0.027869712 |
| O43169 | CYB5B_HUMAN | 0.79374 | -0.028062884 |
| P28070 | PSB4_HUMAN | 0.79405 | 0.035581366 |
| Q7Z434 | MAVS_HUMAN | 0.79521 | -0.038363374 |
| P04440 | DPB1_HUMAN | 0.79749 | -0.056543424 |
| O15551 | CLD3_HUMAN | 0.79795 | -0.053273018 |
| Q9UHQ9 | NB5R1_HUMAN | 0.79799 | -0.041426831 |
| Q9Y2Q5 | LTOR2_HUMAN | 0.79813 | 0.052003612 |
| P61421 | VA0D1_HUMAN | 0.79892 | 0.014448453 |
| P08708 | RS17_HUMAN | 0.79928 | -0.025802364 |
| P63244 | RACK1_HUMAN | 0.80009 | -0.066088987 |
| Q14254 | FLOT2_HUMAN | 0.80163 | 0.03841411 |
| Q92575 | UBXN4_HUMAN | 0.803 | -0.047678747 |
| P43243 | MATR3_HUMAN | 0.80439 | 0.06068086 |
| P23246 | SFPQ_HUMAN | 0.80509 | -0.039957378 |
| Q07065 | CKAP4_HUMAN | 0.80591 | -0.033616001 |
| P46926 | GNPI1_HUMAN | 0.80682 | 0.026306422 |
| Q9UI12 | VATH_HUMAN | 0.80703 | -0.010763888 |
| Q9H488 | OFUT1_HUMAN | 0.80717 | 0.0338369 |
| Q13620 | CUL4B_HUMAN | 0.80874 | -0.041164071 |
| Q8N2K0 | ABD12_HUMAN | 0.8088 | -0.03384639 |
| P56385 | ATP5I_HUMAN | 0.80947 | 0.058324453 |
| O14579 | COPE_HUMAN | 0.81118 | -0.063450805 |
| P60174 | TPIS_HUMAN | 0.81273 | 0.06457206 |
| O75821 | EIF3G_HUMAN | 0.81363 | -0.035308593 |
| Q9Y6U3 | ADSV_HUMAN | 0.81388 | 0.048689109 |
| Q13637 | RAB32_HUMAN | 0.81437 | 0.036443747 |
| P06576 | ATPB_HUMAN | 0.81492 | 0.008852446 |
| O95168-2 | NDUB4_HUMAN | 0.81845 | 0.025757605 |
| P78344 | IF4G2_HUMAN | 0.81943 | -0.018389347 |
| Q8IVH4 | MMAA_HUMAN | 0.82037 | -0.058624509 |
| P18077 | RL35A_HUMAN | 0.82078 | 0.03017817 |
| P40616 | ARL1_HUMAN | 0.82288 | -0.030084728 |
| P62333 | PRS10_HUMAN | 0.82309 | -0.032662404 |
| Q12905 | ILF2_HUMAN | 0.82311 | 0.031562337 |
| Q9H4A6 | GOLP3_HUMAN | 0.82382 | 0.018079307 |
| Q86VB7 | C163A_HUMAN | 0.82519 | 0.050539274 |
| P00488 | F13A_HUMAN | 0.82532 | -0.057242179 |
| P62266 | RS23_HUMAN | 0.82658 | -0.026710503 |
| P36222 | CH3L1_HUMAN | 0.82728 | 0.034762612 |
| P09661 | RU2A_HUMAN | 0.82834 | 0.024866606 |
| P46781 | RS9_HUMAN | 0.82847 | 0.032808497 |
| Q07812 | BAX_HUMAN | 0.82847 | -0.034244069 |
| P00167 | CYB5_HUMAN | 0.82901 | -0.029318132 |
| P27694 | RFA1_HUMAN | 0.8301 | 0.032976746 |
| P61006 | RAB8A_HUMAN | 0.83031 | -0.033003728 |
| P23141 | EST1_HUMAN | 0.8341 | -0.038408461 |
| Q8WUM4 | PDC6I_HUMAN | 0.83422 | -0.020942128 |
| P25789 | PSA4_HUMAN | 0.83427 | -0.006169218 |
| P17980 | PRS6A_HUMAN | 0.83432 | -0.032927014 |
| P18206 | VINC_HUMAN | 0.83484 | 0.024999652 |
| Q9NTK5 | OLA1_HUMAN | 0.83492 | 0.02182863 |
| Q04917 | 1433F_HUMAN | 0.83541 | -0.012957088 |
| Q9HD45 | TM9S3_HUMAN | 0.83632 | 0.016504929 |
| P69905 | HBA_HUMAN | 0.83718 | -0.044665805 |
| P14902 | I23O1_HUMAN | 0.83784 | 0.046193689 |
| P08311 | CATG_HUMAN | 0.83813 | -0.044545278 |
| P09467 | F16P1_HUMAN | 0.83831 | 0.029470726 |
| P48147 | PPCE_HUMAN | 0.84062 | -0.015202834 |
| Q9UKM9 | RALY_HUMAN | 0.84083 | 0.071337185 |
| O76013 | KRT36_HUMAN | 0.84093 | -0.085837474 |
| P06733 | ENOA_HUMAN | 0.8428 | 0.019720997 |
| P09417 | DHPR_HUMAN | 0.84342 | 0.01917947 |
| P49755 | TMEDA_HUMAN | 0.8443 | 0.027669458 |
| A0AVT1 | UBA6_HUMAN | 0.84542 | -0.028477057 |
| Q99961 | SH3G1_HUMAN | 0.84594 | -0.022104045 |
| Q9BVC6 | TM109_HUMAN | 0.847 | 0.012867592 |
| Q14258 | TRI25_HUMAN | 0.84753 | 0.027170395 |
| P22314 | UBA1_HUMAN | 0.84951 | 0.01006777 |
| Q9NYL9 | TMOD3_HUMAN | 0.85012 | -0.030338292 |
| O75695 | XRP2_HUMAN | 0.8502 | -0.029196553 |
| Q9UJZ1 | STML2_HUMAN | 0.8511 | -0.041698721 |
| P63000 | RAC1_HUMAN | 0.85232 | -0.015943922 |
| Q9Y2Z0 | SGT1_HUMAN | 0.85361 | 0.0287351 |
| P50570-2 | DYN2_HUMAN | 0.85375 | -0.050893256 |
| P02545-2 | LMNA_HUMAN | 0.85438 | -0.027304932 |
| Q969E2 | SCAM4_HUMAN | 0.85611 | 0.043399628 |
| P05164 | PERM_HUMAN | 0.85828 | 0.024182083 |
| Q9BXJ9 | NAA15_HUMAN | 0.8586 | -0.039405564 |
| Q9Y2V2 | CHSP1_HUMAN | 0.86078 | 0.037479526 |
| P07339 | CATD_HUMAN | 0.86083 | -0.013106869 |
| P68402 | PA1B2_HUMAN | 0.86273 | -0.033644641 |
| Q16658 | FSCN1_HUMAN | 0.86362 | -0.043292767 |
| P20042 | IF2B_HUMAN | 0.86511 | -0.034852673 |
| P79483 | DRB3_HUMAN | 0.86583 | -0.021700646 |
| P46777 | RL5_HUMAN | 0.86706 | 0.020180633 |
| P38606 | VATA_HUMAN | 0.86735 | -0.011520985 |
| Q14315 | FLNC_HUMAN | 0.8695 | 0.025328917 |
| P22897 | MRC1_HUMAN | 0.86957 | -0.026658619 |
| P50395 | GDIB_HUMAN | 0.87044 | -0.017853863 |
| P84243 | H33_HUMAN | 0.87158 | -0.047524835 |
| O95352 | ATG7_HUMAN | 0.87179 | 0.022018731 |
| P63162 | RSMN_HUMAN | 0.87338 | 0.026403727 |
| Q9Y5M8 | SRPRB_HUMAN | 0.87506 | -0.035249881 |
| O00182 | LEG9_HUMAN | 0.87514 | -0.014313637 |
| P04066 | FUCO_HUMAN | 0.87523 | 0.011670986 |
| Q15631 | TSN_HUMAN | 0.87638 | -0.010417061 |
| P09543 | CN37_HUMAN | 0.87646 | -0.02565415 |
| B0I1T2 | MYO1G_HUMAN | 0.8771 | 0.012808704 |
| O95573 | ACSL3_HUMAN | 0.8773 | 0.015928331 |
| P51149 | RAB7A_HUMAN | 0.87746 | 0.005462404 |
| Q9Y277 | VDAC3_HUMAN | 0.87852 | -0.033015248 |
| Q16181 | SEPT7_HUMAN | 0.87856 | 0.034455197 |
| P31146 | COR1A_HUMAN | 0.87875 | -0.024982844 |
| Q13445 | TMED1_HUMAN | 0.88205 | -0.032165302 |
| Q92841 | DDX17_HUMAN | 0.8825 | -0.017498592 |
| P35998 | PRS7_HUMAN | 0.88325 | -0.013317198 |
| Q9UKK3 | PARP4_HUMAN | 0.88331 | 0.01268612 |
| P01892 | 1A02_HUMAN | 0.88371 | -0.020280503 |
| P07737 | PROF1_HUMAN | 0.88371 | 0.015911681 |
| Q5JPE7 | NOMO2_HUMAN | 0.88381 | -0.022322699 |
| Q13363 | CTBP1_HUMAN | 0.88561 | -0.025139042 |
| P53004 | BIEA_HUMAN | 0.88719 | -0.018639775 |
| Q12906-7 | ILF3_HUMAN | 0.88831 | -0.016855004 |
| O75955 | FLOT1_HUMAN | 0.88876 | 0.025524305 |
| P08631 | HCK_HUMAN | 0.88918 | -0.013167763 |
| Q14376 | GALE_HUMAN | 0.8898 | 0.026142279 |
| Q0VD83 | APOBR_HUMAN | 0.89029 | 0.030760582 |
| P11279 | LAMP1_HUMAN | 0.89096 | -0.017416888 |
| P30050 | RL12_HUMAN | 0.8925 | -0.009464137 |
| Q07020 | RL18_HUMAN | 0.89257 | -0.016339741 |
| Q96FW1 | OTUB1_HUMAN | 0.89275 | 0.01285466 |
| Q9UHD9 | UBQL2_HUMAN | 0.89311 | -0.022443973 |
| Q15942 | ZYX_HUMAN | 0.89345 | 0.037439111 |
| Q15437 | SC23B_HUMAN | 0.89417 | 0.015523797 |
| Q13126 | MTAP_HUMAN | 0.8956 | -0.011659389 |
| Q14697-2 | GANAB_HUMAN | 0.89588 | 0.01771568 |
| O14683 | P5I11_HUMAN | 0.89641 | 0.014972794 |
| O95336 | 6PGL_HUMAN | 0.89738 | -0.007446425 |
| P56556 | NDUA6_HUMAN | 0.89765 | 0.027493171 |
| Q92542 | NICA_HUMAN | 0.899 | -0.006680134 |
| P38159 | RBMX_HUMAN | 0.90021 | 0.032300413 |
| P56537 | IF6_HUMAN | 0.90123 | 0.008703763 |
| Q5RI15 | COX20_HUMAN | 0.9026 | 0.022267959 |
| Q01085 | TIAR_HUMAN | 0.90282 | -0.024794301 |
| Q9ULV4 | COR1C_HUMAN | 0.90301 | -0.021234576 |
| Q96AQ6 | PBIP1_HUMAN | 0.90377 | -0.026884907 |
| P01111 | RASN_HUMAN | 0.90431 | 0.018846123 |
| Q9UH99 | SUN2_HUMAN | 0.90509 | 0.013091996 |
| P41250 | GARS_HUMAN | 0.90566 | -0.014567871 |
| Q8IV08 | PLD3_HUMAN | 0.90609 | -0.007036991 |
| P16435 | NCPR_HUMAN | 0.9077 | -0.01354109 |
| Q9Y315 | DEOC_HUMAN | 0.90792 | -0.006926665 |
| Q13148 | TADBP_HUMAN | 0.90988 | -0.044361022 |
| P48735 | IDHP_HUMAN | 0.90989 | -0.020048719 |
| P54819 | KAD2_HUMAN | 0.91004 | 0.041732181 |
| P19367 | HXK1_HUMAN | 0.91026 | 0.025725603 |
| Q9NR30 | DDX21_HUMAN | 0.91091 | 0.02606304 |
| O60493 | SNX3_HUMAN | 0.91321 | -0.012449236 |
| Q6NYC8 | PPR18_HUMAN | 0.91396 | -0.027123784 |
| P55060 | XPO2_HUMAN | 0.91409 | 0.019021042 |
| O14773 | TPP1_HUMAN | 0.91712 | -0.015027954 |
| Q15293 | RCN1_HUMAN | 0.91748 | 0.019676052 |
| O95479 | G6PE_HUMAN | 0.91762 | 0.024076245 |
| P61586 | RHOA_HUMAN | 0.91878 | -0.007064619 |
| O15347 | HMGB3_HUMAN | 0.91898 | 0.020820739 |
| P0DMV9 | HS71B_HUMAN | 0.92126 | -0.00328319 |
| P54136 | SYRC_HUMAN | 0.92157 | -0.010102681 |
| Q9Y394 | DHRS7_HUMAN | 0.92168 | 0.007680642 |
| Q16555 | DPYL2_HUMAN | 0.9218 | -0.004594866 |
| P07093 | GDN_HUMAN | 0.92257 | 0.017430663 |
| P31948 | STIP1_HUMAN | 0.92262 | 0.004030603 |
| P09972 | ALDOC_HUMAN | 0.92387 | 0.015273366 |
| P13727 | PRG2_HUMAN | 0.92466 | 0.017966248 |
| P20810-9 | ICAL_HUMAN | 0.92481 | -0.017377225 |
| Q32P28 | P3H1_HUMAN | 0.92657 | 0.029057118 |
| P35527 | K1C9_HUMAN | 0.9289 | -0.02967811 |
| P45877 | PPIC_HUMAN | 0.92986 | 0.014646752 |
| O14684 | PTGES_HUMAN | 0.93014 | -0.00957487 |
| P08238 | HS90B_HUMAN | 0.93019 | 0.009075758 |
| P05534 | 1A24_HUMAN | 0.9302 | 0.009241222 |
| P30040 | ERP29_HUMAN | 0.93134 | -0.012860197 |
| P35914 | HMGCL_HUMAN | 0.93184 | 0.025105005 |
| Q03518 | TAP1_HUMAN | 0.93241 | 0.009659738 |
| P18085 | ARF4_HUMAN | 0.93397 | -0.003597151 |
| P43304 | GPDM_HUMAN | 0.93456 | -0.005256555 |
| P68363 | TBA1B_HUMAN | 0.9349 | 0.015612287 |
| O43747 | AP1G1_HUMAN | 0.93515 | 0.009892396 |
| P61803 | DAD1_HUMAN | 0.93536 | -0.008772304 |
| P27824 | CALX_HUMAN | 0.9356 | -0.011459598 |
| P09211 | GSTP1_HUMAN | 0.93629 | 0.013733933 |
| P02649 | APOE_HUMAN | 0.93724 | -0.008845726 |
| P17900 | SAP3_HUMAN | 0.93806 | -0.014187649 |
| O60603 | TLR2_HUMAN | 0.93905 | 0.017221493 |
| Q8WYA6 | CTBL1_HUMAN | 0.93906 | 0.011955174 |
| Q9BTV4 | TMM43_HUMAN | 0.93915 | 0.012149772 |
| P60866 | RS20_HUMAN | 0.94016 | 0.004601844 |
| P08574 | CY1_HUMAN | 0.94126 | 0.011036165 |
| P61626 | LYSC_HUMAN | 0.94136 | -0.011448151 |
| P14174 | MIF_HUMAN | 0.94173 | 0.012400376 |
| P05109 | S10A8_HUMAN | 0.94268 | 0.005303055 |
| P30086 | PEBP1_HUMAN | 0.94274 | 0.004558536 |
| P61254 | RL26_HUMAN | 0.94287 | 0.012854877 |
| P35625 | TIMP3_HUMAN | 0.94289 | -0.016086346 |
| Q06830 | PRDX1_HUMAN | 0.94328 | -0.008766406 |
| P11233 | RALA_HUMAN | 0.94496 | -0.006974983 |
| P28838 | AMPL_HUMAN | 0.9454 | -0.006780096 |
| P11940 | PABP1_HUMAN | 0.94542 | -0.008055452 |
| Q96A65 | EXOC4_HUMAN | 0.94568 | 0.013408106 |
| P32456 | GBP2_HUMAN | 0.94646 | -0.00536398 |
| Q15417 | CNN3_HUMAN | 0.94701 | 0.010187489 |
| P49189-3 | AL9A1_HUMAN | 0.94729 | 0.015822211 |
| P30533 | AMRP_HUMAN | 0.94814 | 0.030915215 |
| O00469 | PLOD2_HUMAN | 0.94845 | -0.022919499 |
| P30481 | 1B44_HUMAN | 0.9528 | 0.008691314 |
| Q96CN7 | ISOC1_HUMAN | 0.9529 | -0.007897355 |
| P62857 | RS28_HUMAN | 0.95356 | 0.00580957 |
| P14598 | NCF1_HUMAN | 0.95362 | 0.008015242 |
| P49591 | SYSC_HUMAN | 0.95438 | -0.00356528 |
| P48668 | K2C6C_HUMAN | 0.95457 | 0.013725532 |
| O43402 | EMC8_HUMAN | 0.95474 | -0.008275558 |
| Q14677 | EPN4_HUMAN | 0.95539 | 0.009060999 |
| O60784 | TOM1_HUMAN | 0.95721 | 0.013177723 |
| Q16531 | DDB1_HUMAN | 0.95727 | 0.007624685 |
| P62820 | RAB1A_HUMAN | 0.95785 | 0.007094004 |
| P05783 | K1C18_HUMAN | 0.95823 | 0.005491973 |
| O75436 | VP26A_HUMAN | 0.95911 | 0.00796413 |
| O95833 | CLIC3_HUMAN | 0.95914 | -0.011256181 |
| P62854 | RS26_HUMAN | 0.95964 | 0.009599226 |
| O75352 | MPU1_HUMAN | 0.96002 | 0.008518121 |
| Q15057 | ACAP2_HUMAN | 0.96133 | -0.006783475 |
| Q96PP8 | GBP5_HUMAN | 0.96202 | -0.012576739 |
| P29590 | PML_HUMAN | 0.96225 | 0.004488165 |
| Q12931 | TRAP1_HUMAN | 0.96227 | -0.006639023 |
| P35611 | ADDA_HUMAN | 0.96232 | 0.006337857 |
| P55884-2 | EIF3B_HUMAN | 0.96256 | -0.005192553 |
| P22102 | PUR2_HUMAN | 0.96271 | 0.006953854 |
| P10768 | ESTD_HUMAN | 0.96295 | -0.003925379 |
| Q9HB90 | RRAGC_HUMAN | 0.96306 | -0.006398967 |
| Q96C23 | GALM_HUMAN | 0.96377 | -0.013842772 |
| Q9H3P7 | GCP60_HUMAN | 0.96381 | 0.007735905 |
| P50454 | SERPH_HUMAN | 0.96419 | 0.007819916 |
| P50995 | ANX11_HUMAN | 0.96547 | -0.011907116 |
| P09651 | ROA1_HUMAN | 0.96772 | 0.007615679 |
| P49588 | SYAC_HUMAN | 0.96807 | -0.004572542 |
| P07686 | HEXB_HUMAN | 0.96818 | 0.007212775 |
| P08779 | K1C16_HUMAN | 0.97002 | 0.010275439 |
| O75533 | SF3B1_HUMAN | 0.97002 | -0.009290198 |
| P50440 | GATM_HUMAN | 0.97032 | 0.008376607 |
| P17693-5 | HLAG_HUMAN | 0.97093 | -0.009277019 |
| Q92820 | GGH_HUMAN | 0.97105 | -0.006006789 |
| Q92974 | ARHG2_HUMAN | 0.97108 | 0.003812744 |
| P52597 | HNRPF_HUMAN | 0.97215 | 0.003128177 |
| O43396 | TXNL1_HUMAN | 0.97314 | 0.001587267 |
| P84098 | RL19_HUMAN | 0.97471 | -0.005321974 |
| P25774 | CATS_HUMAN | 0.97577 | 0.002939191 |
| Q9Y2J2 | E41L3_HUMAN | 0.97605 | 0.001957582 |
| Q6RW13 | ATRAP_HUMAN | 0.97632 | 0.002866902 |
| Q8N5K1 | CISD2_HUMAN | 0.97656 | 0.004946138 |
| O00203 | AP3B1_HUMAN | 0.97703 | -0.003238008 |
| O15498 | YKT6_HUMAN | 0.97756 | 0.001792997 |
| Q9BUJ2 | HNRL1_HUMAN | 0.97804 | 0.003744505 |
| P53990 | IST1_HUMAN | 0.97923 | -0.00236547 |
| Q14204 | DYHC1_HUMAN | 0.97938 | -0.001803256 |
| P52272 | HNRPM_HUMAN | 0.98078 | -0.002270623 |
| Q9NQC3-2 | RTN4_HUMAN | 0.98084 | -0.003753529 |
| P08758 | ANXA5_HUMAN | 0.98117 | 0.0018381 |
| P62829 | RL23_HUMAN | 0.98305 | 0.003769235 |
| Q96C86 | DCPS_HUMAN | 0.9831 | 0.002636054 |
| Q9Y3Z3 | SAMH1_HUMAN | 0.98325 | 0.004522976 |
| Q0JRZ9 | FCHO2_HUMAN | 0.98448 | 0.003145201 |
| Q05086 | UBE3A_HUMAN | 0.98494 | -0.002606377 |
| P60900 | PSA6_HUMAN | 0.98551 | -0.001869947 |
| Q13813 | SPTN1_HUMAN | 0.98608 | -0.003962682 |
| P10515 | ODP2_HUMAN | 0.98613 | -0.001920726 |
| Q15165 | PON2_HUMAN | 0.98709 | 0.004426347 |
| P05388 | RLA0_HUMAN | 0.98724 | 0.00118173 |
| Q9UBW5 | BIN2_HUMAN | 0.98974 | 0.002391229 |
| P13645 | K1C10_HUMAN | 0.9902 | 0.003824532 |
| Q9NYU2 | UGGG1_HUMAN | 0.99039 | 0.001242082 |
| P19971 | TYPH_HUMAN | 0.9908 | -0.002002173 |
| Q9Y6Q1 | CAN6_HUMAN | 0.99133 | -0.002397005 |
| P04083 | ANXA1_HUMAN | 0.99198 | -0.001485251 |
| Q9P0S3 | ORML1_HUMAN | 0.99216 | -0.001322733 |
| Q9HAV0 | GBB4_HUMAN | 0.9928 | 0.001150643 |
| P56377 | AP1S2_HUMAN | 0.99309 | 0.001488772 |
| Q9P0V8 | SLAF8_HUMAN | 0.99343 | -0.002156541 |
| P27338 | AOFB_HUMAN | 0.99345 | 0.001387042 |
| P20700 | LMNB1_HUMAN | 0.99373 | -0.000794027 |
| P11142 | HSP7C_HUMAN | 0.99419 | 0.000201553 |
| P01834 | IGKC_HUMAN | 0.99657 | 0.00076957 |
