# Supplementary material for: Extracellular vesicle-associated miR-515-5p from adipose tissue regulates placental metabolism and fetal growth in gestational diabetes mellitus
Source: Cardiovasc Diabetol. 2025 May 14;24:205. doi: 10.1186/s12933-025-02739-z (PMC12080180; doi:10.1186/s12933-025-02739-z)
Supplement: Supplementary file 8 — Supplementary Material 8 [file 12933_2025_2739_MOESM8_ESM.docx]

**Supplementary Table 7: Table below shows the comparative analysis of enriched gene ontology biological processes (GO-BP) in NGT AT derived EVs treated placental cells generated using vissE**

| **Protein ID** | **Mapped ID** | **p-value** | **Log (Fold Change)** |
| --- | --- | --- | --- |
| Q03405 | UPAR_HUMAN | 0.00704 | -0.365852329 |
| Q9ULC5-3 | ACSL5_HUMAN | 0.00799 | -0.528982291 |
| Q9UBQ5 | EIF3K_HUMAN | 0.00859 | -0.315440031 |
| Q6UW68 | TM205_HUMAN | 0.01013 | 0.353924979 |
| Q658Y4 | F91A1_HUMAN | 0.01054 | -0.292985659 |
| Q13636 | RAB31_HUMAN | 0.01055 | -0.393788862 |
| Q14728 | MFS10_HUMAN | 0.0123 | -0.611106134 |
| P30622-2 | CLIP1_HUMAN | 0.01325 | -0.263417425 |
| P05413 | FABPH_HUMAN | 0.01346 | -0.404988447 |
| Q9UBI6 | GBG12_HUMAN | 0.01537 | -0.409433402 |
| P20160 | CAP7_HUMAN | 0.01636 | -0.323335704 |
| P62750 | RL23A_HUMAN | 0.01973 | 0.211283076 |
| P45880 | VDAC2_HUMAN | 0.01986 | 0.135760379 |
| A0AV96 | RBM47_HUMAN | 0.02072 | -0.58722158 |
| P15153 | RAC2_HUMAN | 0.02153 | -0.315326149 |
| P46977 | STT3A_HUMAN | 0.02182 | 0.267468437 |
| P14625 | ENPL_HUMAN | 0.0228 | -0.066251187 |
| P19823 | ITIH2_HUMAN | 0.02284 | -0.552840729 |
| Q9H299 | SH3L3_HUMAN | 0.02386 | -0.59239207 |
| P08865 | RSSA_HUMAN | 0.02486 | -0.118005439 |
| P40763 | STAT3_HUMAN | 0.02544 | -0.294456352 |
| P33176 | KINH_HUMAN | 0.0259 | -0.241628148 |
| Q15080 | NCF4_HUMAN | 0.02605 | -0.257573049 |
| P13693 | TCTP_HUMAN | 0.02652 | -0.235466887 |
| Q13404 | UB2V1_HUMAN | 0.02717 | -0.273821787 |
| P20702 | ITAX_HUMAN | 0.02831 | -0.602992493 |
| Q9H4A6 | GOLP3_HUMAN | 0.02836 | 0.169649808 |
| P36957 | ODO2_HUMAN | 0.02929 | 0.158036699 |
| Q9H3Z4 | DNJC5_HUMAN | 0.03054 | -0.336282951 |
| P83731 | RL24_HUMAN | 0.0307 | -0.387069885 |
| P63241 | IF5A1_HUMAN | 0.03118 | -0.35319424 |
| Q9Y230 | RUVB2_HUMAN | 0.03137 | -0.396533869 |
| P08962 | CD63_HUMAN | 0.03247 | -0.357176575 |
| P61026 | RAB10_HUMAN | 0.03406 | -0.180512596 |
| Q07960 | RHG01_HUMAN | 0.03447 | -0.20917849 |
| P67809 | YBOX1_HUMAN | 0.03451 | -0.295128766 |
| P02788 | TRFL_HUMAN | 0.03463 | -0.306953732 |
| Q9UBR2 | CATZ_HUMAN | 0.03691 | -0.236329933 |
| O43252 | PAPS1_HUMAN | 0.03731 | -0.320389647 |
| O15427 | MOT4_HUMAN | 0.03789 | -0.316058534 |
| P62979 | RS27A_HUMAN | 0.03838 | 0.084321597 |
| Q13459 | MYO9B_HUMAN | 0.03888 | -0.342806129 |
| O95674 | CDS2_HUMAN | 0.03908 | 0.279478918 |
| Q14839 | CHD4_HUMAN | 0.03924 | -0.359494622 |
| Q14008 | CKAP5_HUMAN | 0.0393 | -0.244252779 |
| P36551 | HEM6_HUMAN | 0.03954 | -0.26888969 |
| P15144 | AMPN_HUMAN | 0.03986 | -0.290107104 |
| P23284 | PPIB_HUMAN | 0.04042 | -0.109423833 |
| O43399-7 | TPD54_HUMAN | 0.04111 | -0.223915471 |
| O75964 | ATP5L_HUMAN | 0.04176 | -0.565228443 |
| Q9UHA4 | LTOR3_HUMAN | 0.04201 | -0.336432631 |
| Q14152 | EIF3A_HUMAN | 0.04282 | 0.194820405 |
| P30484 | 1B46_HUMAN | 0.0452 | -0.344527212 |
| O95373 | IPO7_HUMAN | 0.04557 | -0.490431529 |
| P62753 | RS6_HUMAN | 0.0458 | -0.395833546 |
| A1L0T0 | ILVBL_HUMAN | 0.04611 | 0.234224577 |
| P00441 | SODC_HUMAN | 0.04671 | -0.404758159 |
| Q08722 | CD47_HUMAN | 0.04756 | 0.155615448 |
| P02647 | APOA1_HUMAN | 0.0488 | -0.420870698 |
| Q9HC38 | GLOD4_HUMAN | 0.04881 | -0.205322606 |
| Q7Z4W1 | DCXR_HUMAN | 0.04931 | 0.144041941 |
| P69891 | HBG1_HUMAN | 0.05006 | -0.327329695 |
| P52657 | T2AG_HUMAN | 0.05143 | 0.163486506 |
| Q13510 | ASAH1_HUMAN | 0.05196 | -0.107158032 |
| O95433 | AHSA1_HUMAN | 0.05271 | -0.184772842 |
| P18510 | IL1RA_HUMAN | 0.05371 | -0.282892025 |
| P0DP25 | CALM3_HUMAN | 0.0541 | -0.197950933 |
| O00154 | BACH_HUMAN | 0.05434 | -0.359707817 |
| P62937 | PPIA_HUMAN | 0.05473 | -0.190404968 |
| P18621 | RL17_HUMAN | 0.05482 | 0.245744229 |
| P04843 | RPN1_HUMAN | 0.05505 | 0.118714713 |
| P26885 | FKBP2_HUMAN | 0.05506 | 0.197616191 |
| P05387 | RLA2_HUMAN | 0.05608 | -0.387917485 |
| P02787 | TRFE_HUMAN | 0.05622 | -0.430116518 |
| Q8IZ83 | A16A1_HUMAN | 0.05715 | 0.338729665 |
| P62993 | GRB2_HUMAN | 0.0575 | -0.313348927 |
| P49411 | EFTU_HUMAN | 0.05785 | -0.506753535 |
| O75477 | ERLN1_HUMAN | 0.05796 | 0.258319743 |
| P13284 | GILT_HUMAN | 0.05881 | -0.676072524 |
| Q7Z6Z7 | HUWE1_HUMAN | 0.05883 | -0.346390793 |
| P02774 | VTDB_HUMAN | 0.0594 | -0.446554288 |
| P46782 | RS5_HUMAN | 0.0604 | -0.12681205 |
| P41218 | MNDA_HUMAN | 0.06046 | -0.229131986 |
| Q969X1 | LFG3_HUMAN | 0.06106 | 0.297934988 |
| Q7L1Q6-2 | BZW1_HUMAN | 0.06238 | -0.42217795 |
| P34910-2 | EVI2B_HUMAN | 0.06343 | 0.194941005 |
| P05543 | THBG_HUMAN | 0.0637 | -0.550750067 |
| P51659 | DHB4_HUMAN | 0.06372 | -0.935180676 |
| P60228 | EIF3E_HUMAN | 0.06379 | 0.143776303 |
| P30520 | PURA2_HUMAN | 0.06429 | 0.118155287 |
| Q6YP21 | KAT3_HUMAN | 0.0645 | -0.326093948 |
| O95379 | TFIP8_HUMAN | 0.06503 | -0.271120401 |
| Q8WWP7 | GIMA1_HUMAN | 0.06579 | -0.558307071 |
| P14780 | MMP9_HUMAN | 0.06653 | -0.454786978 |
| P61619 | S61A1_HUMAN | 0.0666 | -0.245438655 |
| Q92973 | TNPO1_HUMAN | 0.06665 | 0.370548024 |
| P35579 | MYH9_HUMAN | 0.06699 | 0.168649463 |
| P04839 | CY24B_HUMAN | 0.06742 | -0.232282949 |
| P08670 | VIME_HUMAN | 0.06887 | -0.138451461 |
| Q96A26 | F162A_HUMAN | 0.06975 | 0.475075089 |
| P47914 | RL29_HUMAN | 0.07006 | 0.235362455 |
| Q9NZM1 | MYOF_HUMAN | 0.07131 | 0.139042909 |
| Q9ULZ3 | ASC_HUMAN | 0.07193 | -0.179516036 |
| Q14240 | IF4A2_HUMAN | 0.07225 | 0.271309024 |
| Q96Q11 | TRNT1_HUMAN | 0.07314 | -0.484233817 |
| O76003 | GLRX3_HUMAN | 0.07326 | -0.204691003 |
| P62834 | RAP1A_HUMAN | 0.07335 | 0.453877653 |
| Q15084-5 | PDIA6_HUMAN | 0.07339 | -0.186707788 |
| P31949 | S10AB_HUMAN | 0.07362 | -0.264652998 |
| P09917 | LOX5_HUMAN | 0.07379 | -0.186387115 |
| P46776 | RL27A_HUMAN | 0.07387 | 0.259449787 |
| P78527 | PRKDC_HUMAN | 0.07399 | -0.346865986 |
| P33241 | LSP1_HUMAN | 0.07417 | -0.406603919 |
| Q9Y5P6-2 | GMPPB_HUMAN | 0.0742 | -0.953403661 |
| Q00325-2 | MPCP_HUMAN | 0.07434 | 0.114402441 |
| P08727 | K1C19_HUMAN | 0.07502 | -0.267574104 |
| P43003 | EAA1_HUMAN | 0.0751 | -0.303901713 |
| P35270 | SPRE_HUMAN | 0.07631 | -0.270291667 |
| O96000 | NDUBA_HUMAN | 0.07754 | -0.501771839 |
| P36578 | RL4_HUMAN | 0.07835 | 0.260430855 |
| P31040 | SDHA_HUMAN | 0.07888 | 0.221700106 |
| P39656 | OST48_HUMAN | 0.07924 | 0.087354853 |
| P50552 | VASP_HUMAN | 0.07944 | -0.115345472 |
| P54687 | BCAT1_HUMAN | 0.08012 | -0.35481191 |
| P15121 | ALDR_HUMAN | 0.08014 | 0.533314146 |
| Q92572 | AP3S1_HUMAN | 0.08105 | 0.219385968 |
| P58546 | MTPN_HUMAN | 0.08174 | -0.271424684 |
| P40227 | TCPZ_HUMAN | 0.08213 | -0.378397584 |
| Q14165 | MLEC_HUMAN | 0.08254 | 0.158477786 |
| Q9NY33 | DPP3_HUMAN | 0.0829 | 0.262418547 |
| P49354 | FNTA_HUMAN | 0.08342 | -0.229163774 |
| P05161 | ISG15_HUMAN | 0.08375 | 0.239410979 |
| Q9H8S9 | MOB1A_HUMAN | 0.0844 | -0.073402113 |
| Q14956 | GPNMB_HUMAN | 0.08597 | -0.440141541 |
| O60271 | JIP4_HUMAN | 0.08651 | -0.311382096 |
| Q12907 | LMAN2_HUMAN | 0.08669 | -0.18023738 |
| O43143 | DHX15_HUMAN | 0.08778 | 0.132418183 |
| Q13185 | CBX3_HUMAN | 0.08828 | -0.177305592 |
| O60763 | USO1_HUMAN | 0.08948 | -0.055600063 |
| P41091 | IF2G_HUMAN | 0.09014 | 0.369584248 |
| Q8N0X7 | SPART_HUMAN | 0.09039 | -0.147281382 |
| P21912 | SDHB_HUMAN | 0.09064 | -0.330027731 |
| Q99653 | CHP1_HUMAN | 0.09093 | -0.318423441 |
| Q9UGQ3 | GTR6_HUMAN | 0.09116 | -0.216039977 |
| P19878 | NCF2_HUMAN | 0.09125 | 0.196552912 |
| O00299 | CLIC1_HUMAN | 0.09191 | -0.206948947 |
| P12109 | CO6A1_HUMAN | 0.09195 | -0.416713093 |
| P49207 | RL34_HUMAN | 0.09238 | 0.287592628 |
| Q9Y2S2 | CRYL1_HUMAN | 0.09427 | -0.381430045 |
| O00764 | PDXK_HUMAN | 0.09433 | -0.192193852 |
| O00410 | IPO5_HUMAN | 0.09442 | -0.236543305 |
| P04792 | HSPB1_HUMAN | 0.09757 | -0.179064738 |
| Q96SQ9 | CP2S1_HUMAN | 0.09836 | -0.289665394 |
| P36543 | VATE1_HUMAN | 0.09924 | -0.13129658 |
| Q13347 | EIF3I_HUMAN | 0.10002 | 0.188246282 |
| P46940 | IQGA1_HUMAN | 0.1006 | 0.082988923 |
| P67812 | SC11A_HUMAN | 0.10146 | -0.213615172 |
| P05107 | ITB2_HUMAN | 0.10199 | -0.171961629 |
| P30041 | PRDX6_HUMAN | 0.10224 | -0.234308714 |
| Q96HY6 | DDRGK_HUMAN | 0.10307 | 0.19083047 |
| Q8IWB7 | WDFY1_HUMAN | 0.10312 | -0.388365332 |
| Q7RTV0 | PHF5A_HUMAN | 0.1032 | 0.255479574 |
| P51991 | ROA3_HUMAN | 0.10354 | -0.191481388 |
| Q9Y4P3 | TBL2_HUMAN | 0.10433 | -0.1477563 |
| O94903 | PLPHP_HUMAN | 0.10467 | -0.223347635 |
| P00558 | PGK1_HUMAN | 0.10528 | -0.096362228 |
| P10644 | KAP0_HUMAN | 0.10546 | -0.211761979 |
| Q6NUK1 | SCMC1_HUMAN | 0.10566 | -0.297147736 |
| P14927 | QCR7_HUMAN | 0.10612 | -0.186719504 |
| Q15642 | CIP4_HUMAN | 0.10651 | -0.329516747 |
| P52907 | CAZA1_HUMAN | 0.10704 | -0.177369275 |
| P49748 | ACADV_HUMAN | 0.10876 | -0.392879509 |
| Q9BTZ2 | DHRS4_HUMAN | 0.10916 | -0.193219352 |
| P12111 | CO6A3_HUMAN | 0.10941 | -0.389365968 |
| Q969E2 | SCAM4_HUMAN | 0.11 | 0.26090319 |
| P24534 | EF1B_HUMAN | 0.11007 | -0.224441171 |
| P30044 | PRDX5_HUMAN | 0.11185 | 0.279527653 |
| P61758 | PFD3_HUMAN | 0.11186 | -0.195942857 |
| P02771 | FETA_HUMAN | 0.11373 | -0.346875705 |
| Q9UJS0 | CMC2_HUMAN | 0.11403 | 0.122883752 |
| P54802 | ANAG_HUMAN | 0.11425 | -0.23940423 |
| O95777 | LSM8_HUMAN | 0.11463 | -0.735680322 |
| Q03169 | TNAP2_HUMAN | 0.11518 | -0.274668937 |
| P23396 | RS3_HUMAN | 0.11527 | 0.196853392 |
| P15880 | RS2_HUMAN | 0.11585 | 0.18349748 |
| P35754 | GLRX1_HUMAN | 0.11639 | -0.248534504 |
| P07858 | CATB_HUMAN | 0.11716 | -0.174278942 |
| P30049 | ATPD_HUMAN | 0.1174 | -0.175252868 |
| Q15029 | U5S1_HUMAN | 0.11799 | 0.126466596 |
| P13796 | PLSL_HUMAN | 0.1182 | -0.096151958 |
| P31946 | 1433B_HUMAN | 0.11951 | -0.115615708 |
| P30101 | PDIA3_HUMAN | 0.12049 | -0.308465014 |
| O60884 | DNJA2_HUMAN | 0.12061 | 0.320111384 |
| Q7Z5R6 | AB1IP_HUMAN | 0.12171 | 0.155483446 |
| Q13287 | NMI_HUMAN | 0.12225 | 0.242296535 |
| P63010 | AP2B1_HUMAN | 0.12236 | 0.15799602 |
| Q13283 | G3BP1_HUMAN | 0.12247 | -0.283756304 |
| Q92499 | DDX1_HUMAN | 0.12265 | -0.360141905 |
| Q9NSD9 | SYFB_HUMAN | 0.12298 | 0.632453349 |
| P28331 | NDUS1_HUMAN | 0.12374 | -0.183254712 |
| P51572 | BAP31_HUMAN | 0.1239 | 0.265009387 |
| O60234 | GMFG_HUMAN | 0.12406 | -0.164065548 |
| P55145 | MANF_HUMAN | 0.1242 | -0.325456845 |
| P39019 | RS19_HUMAN | 0.12439 | -0.166193381 |
| Q9H4M9 | EHD1_HUMAN | 0.12493 | -0.219358339 |
| P08754 | GNAI3_HUMAN | 0.12542 | -0.071989728 |
| O75306 | NDUS2_HUMAN | 0.12559 | -0.310907488 |
| P11021 | BIP_HUMAN | 0.12628 | -0.044892952 |
| P61353 | RL27_HUMAN | 0.12674 | 0.263474249 |
| Q8TDB6 | DTX3L_HUMAN | 0.12681 | -0.381512622 |
| P04040 | CATA_HUMAN | 0.12726 | 0.356400265 |
| P32119 | PRDX2_HUMAN | 0.12799 | -0.201250267 |
| Q03519 | TAP2_HUMAN | 0.12819 | 0.201564586 |
| Q15907 | RB11B_HUMAN | 0.1284 | -0.060099182 |
| Q8N6L1 | KTAP2_HUMAN | 0.12866 | 0.152966148 |
| Q12979 | ABR_HUMAN | 0.12872 | 0.262959726 |
| Q8TD19 | NEK9_HUMAN | 0.12882 | -0.212791403 |
| O15511 | ARPC5_HUMAN | 0.13002 | -0.124495652 |
| Q6IAA8 | LTOR1_HUMAN | 0.1306 | -0.203005257 |
| E9PAV3 | NACAM_HUMAN | 0.13171 | -0.114368535 |
| P54577 | SYYC_HUMAN | 0.13235 | -0.204207418 |
| P21926 | CD9_HUMAN | 0.13274 | -0.31041374 |
| O43681 | ASNA_HUMAN | 0.13336 | -0.156452441 |
| P12814 | ACTN1_HUMAN | 0.1349 | -0.061324196 |
| P26038 | MOES_HUMAN | 0.13543 | 0.124477967 |
| Q99829 | CPNE1_HUMAN | 0.13553 | -0.2187147 |
| P40306 | PSB10_HUMAN | 0.13577 | -0.162008715 |
| O75533 | SF3B1_HUMAN | 0.13633 | 0.236135071 |
| P18124 | RL7_HUMAN | 0.13652 | 0.129197697 |
| P30043 | BLVRB_HUMAN | 0.13812 | -0.250368063 |
| O75368 | SH3L1_HUMAN | 0.13816 | -0.447513778 |
| P69892 | HBG2_HUMAN | 0.13818 | -0.253693124 |
| P07602 | SAP_HUMAN | 0.14127 | -0.319476903 |
| P12110 | CO6A2_HUMAN | 0.14147 | -0.329466015 |
| P05388 | RLA0_HUMAN | 0.14212 | -0.100643028 |
| P30048 | PRDX3_HUMAN | 0.14215 | -0.240664976 |
| Q96CW1 | AP2M1_HUMAN | 0.14254 | 0.381234762 |
| Q9NR28 | DBLOH_HUMAN | 0.14278 | 0.162751256 |
| Q03518 | TAP1_HUMAN | 0.14432 | -0.179285741 |
| P30273 | FCERG_HUMAN | 0.14462 | -0.100303987 |
| P62899 | RL31_HUMAN | 0.14469 | 0.151125501 |
| P55957 | BID_HUMAN | 0.14627 | 0.236012149 |
| Q15904 | VAS1_HUMAN | 0.14673 | -0.485390319 |
| P23588 | IF4B_HUMAN | 0.14745 | -0.389337929 |
| Q9Y696 | CLIC4_HUMAN | 0.14809 | -0.23392061 |
| O43493 | TGON2_HUMAN | 0.14864 | -0.307069963 |
| P60660-2 | MYL6_HUMAN | 0.14912 | -0.131851408 |
| P55265 | DSRAD_HUMAN | 0.14915 | 0.238615296 |
| P14061 | DHB1_HUMAN | 0.14936 | -0.424081614 |
| P29692-2 | EF1D_HUMAN | 0.1495 | -0.13270663 |
| P02768 | ALBU_HUMAN | 0.15142 | -0.522699215 |
| Q9C002 | NMES1_HUMAN | 0.15144 | -0.287902651 |
| P61586 | RHOA_HUMAN | 0.1521 | -0.102192534 |
| P19801 | AOC1_HUMAN | 0.15215 | -0.310689542 |
| P05198 | IF2A_HUMAN | 0.15253 | 0.106465224 |
| P40926 | MDHM_HUMAN | 0.15259 | -0.178692675 |
| Q16576 | RBBP7_HUMAN | 0.1532 | -0.184383404 |
| Q15459 | SF3A1_HUMAN | 0.15339 | 0.193592555 |
| P08842 | STS_HUMAN | 0.15347 | 0.256445229 |
| Q9UM00 | TMCO1_HUMAN | 0.15368 | -0.286140162 |
| P37837 | TALDO_HUMAN | 0.154 | -0.13078045 |
| P13010 | XRCC5_HUMAN | 0.15468 | 0.094033363 |
| P50395 | GDIB_HUMAN | 0.15517 | -0.161436099 |
| Q96HC4 | PDLI5_HUMAN | 0.1552 | 0.176375944 |
| Q9Y639 | NPTN_HUMAN | 0.15739 | -0.226696467 |
| P02042 | HBD_HUMAN | 0.1574 | -0.394897973 |
| P50897 | PPT1_HUMAN | 0.15857 | -0.246793642 |
| P28072 | PSB6_HUMAN | 0.15877 | 0.109475037 |
| Q8TEM1 | PO210_HUMAN | 0.15882 | 0.221027029 |
| Q9Y3A6 | TMED5_HUMAN | 0.15891 | -0.191068868 |
| Q01995 | TAGL_HUMAN | 0.15973 | -0.426574784 |
| Q9Y277 | VDAC3_HUMAN | 0.16026 | 0.329866831 |
| Q15758 | AAAT_HUMAN | 0.16045 | -0.19961765 |
| Q96PP9 | GBP4_HUMAN | 0.16154 | -0.223815355 |
| P34932 | HSP74_HUMAN | 0.16217 | 0.065892817 |
| P21796 | VDAC1_HUMAN | 0.16343 | 0.247383292 |
| P54652 | HSP72_HUMAN | 0.16367 | 0.175636445 |
| P40429 | RL13A_HUMAN | 0.16507 | 0.153401222 |
| Q14315 | FLNC_HUMAN | 0.16513 | 0.159679274 |
| O75489 | NDUS3_HUMAN | 0.1688 | -0.14437718 |
| P54727 | RD23B_HUMAN | 0.16892 | 0.207348743 |
| P38606 | VATA_HUMAN | 0.1691 | -0.10536498 |
| Q8N0U8 | VKORL_HUMAN | 0.17017 | 0.341073304 |
| Q14213 | IL27B_HUMAN | 0.17042 | -0.362231793 |
| P00918 | CAH2_HUMAN | 0.17137 | -0.143619402 |
| O15118 | NPC1_HUMAN | 0.17193 | 0.10244284 |
| P06702 | S10A9_HUMAN | 0.17209 | -0.146212144 |
| Q96TA1 | NIBL1_HUMAN | 0.17297 | 0.417354468 |
| O15258 | RER1_HUMAN | 0.17377 | -0.177781691 |
| P46779 | RL28_HUMAN | 0.17465 | 0.321008748 |
| P27487 | DPP4_HUMAN | 0.17506 | 0.103778456 |
| O75821 | EIF3G_HUMAN | 0.17508 | 0.177751038 |
| Q9Y2B0 | CNPY2_HUMAN | 0.17682 | -0.245546268 |
| P0DMV9 | HS71B_HUMAN | 0.17692 | -0.043238635 |
| Q8NCW5 | NNRE_HUMAN | 0.17714 | -0.237624264 |
| Q15435 | PP1R7_HUMAN | 0.17788 | -0.358931518 |
| Q96T76-8 | MMS19_HUMAN | 0.17813 | 0.438246231 |
| P50452 | SPB8_HUMAN | 0.17924 | -0.263175525 |
| Q13488 | VPP3_HUMAN | 0.17944 | -0.134967865 |
| P60174 | TPIS_HUMAN | 0.18019 | -0.402313592 |
| O00203 | AP3B1_HUMAN | 0.18117 | -0.153953646 |
| Q9NP72 | RAB18_HUMAN | 0.18138 | 0.065996515 |
| Q9UH65 | SWP70_HUMAN | 0.18193 | -0.082121608 |
| Q13363 | CTBP1_HUMAN | 0.18228 | -0.362105105 |
| P00846 | ATP6_HUMAN | 0.18237 | -0.44257565 |
| Q92882 | OSTF1_HUMAN | 0.18238 | -0.151177562 |
| Q9Y295 | DRG1_HUMAN | 0.183 | -0.088535419 |
| P13727 | PRG2_HUMAN | 0.18301 | 0.380221081 |
| P48960 | CD97_HUMAN | 0.18374 | -0.146647645 |
| P16152 | CBR1_HUMAN | 0.18424 | -0.117098522 |
| P18669 | PGAM1_HUMAN | 0.18497 | 0.158473883 |
| Q96AG4 | LRC59_HUMAN | 0.18543 | -0.20504254 |
| P56537 | IF6_HUMAN | 0.18624 | -0.097434773 |
| P51606 | RENBP_HUMAN | 0.18685 | 0.192836311 |
| Q96D96 | HVCN1_HUMAN | 0.18699 | 0.167598519 |
| P04080 | CYTB_HUMAN | 0.18794 | -0.378278742 |
| Q8IY17 | PLPL6_HUMAN | 0.1881 | 0.232697485 |
| P16930 | FAAA_HUMAN | 0.18871 | 0.220566815 |
| Q8IXB1 | DJC10_HUMAN | 0.18899 | -0.165798381 |
| P48163 | MAOX_HUMAN | 0.18919 | 0.286579736 |
| Q9Y224 | RTRAF_HUMAN | 0.1894 | -0.205422753 |
| Q9Y6C9 | MTCH2_HUMAN | 0.18977 | 0.091301303 |
| Q8TC12 | RDH11_HUMAN | 0.1904 | -0.189881501 |
| P04179 | SODM_HUMAN | 0.19126 | -0.139698604 |
| Q9HCU5 | PREB_HUMAN | 0.19156 | 0.412946886 |
| O75323 | NIPS2_HUMAN | 0.19223 | 0.277336074 |
| Q08J23 | NSUN2_HUMAN | 0.1938 | -0.197387974 |
| Q9Y5L0 | TNPO3_HUMAN | 0.19392 | 0.53453645 |
| Q13263 | TIF1B_HUMAN | 0.19418 | -0.288076082 |
| Q70UQ0-4 | IKIP_HUMAN | 0.19455 | -0.177635546 |
| Q9NXW2 | DJB12_HUMAN | 0.19585 | -0.291739933 |
| P50990 | TCPQ_HUMAN | 0.19675 | 0.106192845 |
| Q96IJ6 | GMPPA_HUMAN | 0.19732 | 0.27160281 |
| Q27J81 | INF2_HUMAN | 0.19733 | -0.155597973 |
| Q3V6T2 | GRDN_HUMAN | 0.19763 | 0.213730475 |
| P40616 | ARL1_HUMAN | 0.19788 | -0.161399691 |
| P33947 | ERD22_HUMAN | 0.19805 | 0.376384696 |
| Q9BYU5 | KRA21_HUMAN | 0.19833 | 0.381146963 |
| P60709 | ACTB_HUMAN | 0.19887 | 0.080977289 |
| Q9Y3D6 | FIS1_HUMAN | 0.19946 | -0.188899789 |
| P13489 | RINI_HUMAN | 0.1996 | -0.033199228 |
| Q16851 | UGPA_HUMAN | 0.20078 | -0.147197737 |
| Q9Y6N5 | SQOR_HUMAN | 0.20094 | 0.145032473 |
| O60826 | CCD22_HUMAN | 0.20099 | -0.207257942 |
| P37802 | TAGL2_HUMAN | 0.20129 | -0.178514094 |
| P24539 | AT5F1_HUMAN | 0.20165 | 0.103468524 |
| Q14444 | CAPR1_HUMAN | 0.20301 | -0.105126102 |
| P55209 | NP1L1_HUMAN | 0.20319 | -0.130203379 |
| Q9NX14 | NDUBB_HUMAN | 0.20354 | 0.195990336 |
| O00232 | PSD12_HUMAN | 0.20383 | -0.171484005 |
| Q06033 | ITIH3_HUMAN | 0.20412 | -0.130748417 |
| P25815 | S100P_HUMAN | 0.20416 | -0.289324015 |
| P04440 | DPB1_HUMAN | 0.2043 | 0.206747106 |
| Q02978 | M2OM_HUMAN | 0.20446 | 0.168186011 |
| P21964 | COMT_HUMAN | 0.2067 | -0.20187076 |
| Q15637 | SF01_HUMAN | 0.20684 | 0.192066271 |
| Q96CX2 | KCD12_HUMAN | 0.20703 | -0.103740404 |
| P32969 | RL9_HUMAN | 0.20735 | 0.115150626 |
| P62917 | RL8_HUMAN | 0.20737 | 0.153108921 |
| Q9HC35 | EMAL4_HUMAN | 0.20752 | 0.155725029 |
| Q460N5 | PAR14_HUMAN | 0.20963 | 0.18031415 |
| P04406 | G3P_HUMAN | 0.20995 | -0.08175996 |
| Q9BUL8 | PDC10_HUMAN | 0.21048 | -0.133913758 |
| O75608 | LYPA1_HUMAN | 0.21057 | 0.182989379 |
| Q13617 | CUL2_HUMAN | 0.21243 | -0.309194744 |
| O15143 | ARC1B_HUMAN | 0.21257 | 0.093184646 |
| Q9NR45 | SIAS_HUMAN | 0.21313 | -0.111221243 |
| P04075 | ALDOA_HUMAN | 0.21319 | 0.077164447 |
| Q86UX7 | URP2_HUMAN | 0.21362 | -0.101205252 |
| P35580 | MYH10_HUMAN | 0.21373 | 0.157969398 |
| P62195 | PRS8_HUMAN | 0.21393 | 0.11098076 |
| P55072 | TERA_HUMAN | 0.21412 | -0.114814614 |
| O00625 | PIR_HUMAN | 0.21514 | -0.123579291 |
| P16070 | CD44_HUMAN | 0.21677 | -0.115064634 |
| P67775 | PP2AA_HUMAN | 0.21733 | -0.432347046 |
| P30153 | 2AAA_HUMAN | 0.218 | -0.173192544 |
| Q9NTX5 | ECHD1_HUMAN | 0.2193 | -0.132468798 |
| Q92930 | RAB8B_HUMAN | 0.21945 | -0.155328756 |
| P05362 | ICAM1_HUMAN | 0.21994 | -0.107116363 |
| P52630 | STAT2_HUMAN | 0.22132 | -0.220636867 |
| P27348 | 1433T_HUMAN | 0.22242 | -0.24839456 |
| P31948 | STIP1_HUMAN | 0.22276 | -0.057883351 |
| Q9P0S9 | TM14C_HUMAN | 0.22301 | 0.162934352 |
| P68104 | EF1A1_HUMAN | 0.22353 | 0.082321651 |
| Q9NZ01 | TECR_HUMAN | 0.2238 | 0.422343103 |
| O60711 | LPXN_HUMAN | 0.22384 | -0.147728173 |
| P02511 | CRYAB_HUMAN | 0.22408 | -0.391685367 |
| A0MZ66 | SHOT1_HUMAN | 0.2241 | 0.172085251 |
| P61225 | RAP2B_HUMAN | 0.22557 | 0.178016995 |
| O95336 | 6PGL_HUMAN | 0.22627 | -0.063015792 |
| Q6IBS0 | TWF2_HUMAN | 0.22634 | -0.169251821 |
| P62318 | SMD3_HUMAN | 0.22634 | 0.115065854 |
| P62249 | RS16_HUMAN | 0.22657 | 0.166260974 |
| P08571 | CD14_HUMAN | 0.22707 | -0.138607019 |
| Q9UNH7 | SNX6_HUMAN | 0.22714 | -0.124551478 |
| Q96AQ6 | PBIP1_HUMAN | 0.22796 | 0.175069655 |
| Q96HE7 | ERO1A_HUMAN | 0.22845 | -0.130248684 |
| Q13177 | PAK2_HUMAN | 0.22854 | -0.131742227 |
| P21333 | FLNA_HUMAN | 0.23036 | -0.075242571 |
| P54886 | P5CS_HUMAN | 0.23056 | -0.218454458 |
| P55786 | PSA_HUMAN | 0.231 | -0.156455267 |
| P63173 | RL38_HUMAN | 0.23137 | 0.165498221 |
| P23469-2 | PTPRE_HUMAN | 0.23323 | -0.137724514 |
| Q8N1F7 | NUP93_HUMAN | 0.2333 | 0.119391326 |
| P61158 | ARP3_HUMAN | 0.23454 | -0.08207456 |
| P25788 | PSA3_HUMAN | 0.23537 | -0.237681224 |
| Q14697-2 | GANAB_HUMAN | 0.23636 | -0.14191998 |
| P20292 | AL5AP_HUMAN | 0.23665 | -0.122233152 |
| P08133 | ANXA6_HUMAN | 0.23697 | 0.175899304 |
| P09543 | CN37_HUMAN | 0.23788 | -0.246297408 |
| O00182 | LEG9_HUMAN | 0.2381 | -0.148847084 |
| O75352 | MPU1_HUMAN | 0.23834 | -0.18345206 |
| Q96CN7 | ISOC1_HUMAN | 0.23929 | -0.155301989 |
| P08631 | HCK_HUMAN | 0.24022 | -0.118232444 |
| Q92820 | GGH_HUMAN | 0.24024 | -0.18099201 |
| P36871 | PGM1_HUMAN | 0.24147 | 0.177778008 |
| P23528 | COF1_HUMAN | 0.2415 | -0.104374552 |
| Q9BPW8 | NIPS1_HUMAN | 0.24163 | 0.190911575 |
| P62277 | RS13_HUMAN | 0.24217 | 0.170494335 |
| Q00796 | DHSO_HUMAN | 0.24265 | -0.183110007 |
| Q9NZL9 | MAT2B_HUMAN | 0.24348 | 0.215090572 |
| Q9UNF0 | PACN2_HUMAN | 0.24371 | -0.170843747 |
| P47755 | CAZA2_HUMAN | 0.24451 | -0.179447838 |
| Q9H4G4 | GAPR1_HUMAN | 0.24472 | 0.230781756 |
| P24557 | THAS_HUMAN | 0.24532 | -0.24918539 |
| Q99497 | PARK7_HUMAN | 0.24623 | -0.087331912 |
| Q86Y82 | STX12_HUMAN | 0.2467 | -0.119005797 |
| Q96P70 | IPO9_HUMAN | 0.24691 | 0.599430682 |
| P62314 | SMD1_HUMAN | 0.24697 | 0.08981968 |
| Q09161 | NCBP1_HUMAN | 0.2475 | -0.185540254 |
| Q9BYT8 | NEUL_HUMAN | 0.2481 | -0.157736156 |
| Q9NVJ2 | ARL8B_HUMAN | 0.24871 | -0.167074045 |
| Q92556 | ELMO1_HUMAN | 0.24912 | 0.245652355 |
| Q99798 | ACON_HUMAN | 0.24921 | -0.101385539 |
| P10599 | THIO_HUMAN | 0.24981 | -0.105147418 |
| Q9Y3F4 | STRAP_HUMAN | 0.25195 | 0.319128226 |
| P25787 | PSA2_HUMAN | 0.25223 | -0.092987048 |
| P62081 | RS7_HUMAN | 0.25383 | -0.179419349 |
| Q9P035 | HACD3_HUMAN | 0.25414 | 0.143538841 |
| P38646 | GRP75_HUMAN | 0.25573 | -0.040094734 |
| P11387 | TOP1_HUMAN | 0.256 | -0.217228307 |
| P43243 | MATR3_HUMAN | 0.25634 | 0.269748145 |
| P30626 | SORCN_HUMAN | 0.25683 | -0.16085059 |
| Q9BXS5 | AP1M1_HUMAN | 0.25696 | 0.111537854 |
| P07093 | GDN_HUMAN | 0.25809 | 0.285605021 |
| P62873 | GBB1_HUMAN | 0.25824 | 0.106963663 |
| P14618 | KPYM_HUMAN | 0.25912 | 0.074345343 |
| Q13423 | NNTM_HUMAN | 0.25947 | 0.071174247 |
| Q08211 | DHX9_HUMAN | 0.25965 | 0.06250672 |
| P18085 | ARF4_HUMAN | 0.25979 | -0.04796776 |
| P61289 | PSME3_HUMAN | 0.25989 | 0.175335786 |
| P02538 | K2C6A_HUMAN | 0.2604 | 0.219302954 |
| P13798 | ACPH_HUMAN | 0.26168 | 0.07174316 |
| O75915 | PRAF3_HUMAN | 0.2629 | 0.165094928 |
| O75298 | RTN2_HUMAN | 0.26546 | 0.308013493 |
| P05534 | 1A24_HUMAN | 0.26599 | 0.135568318 |
| P98082 | DAB2_HUMAN | 0.26659 | -0.1943336 |
| P21589 | 5NTD_HUMAN | 0.26668 | 0.210427337 |
| P52789 | HXK2_HUMAN | 0.26857 | -0.130337126 |
| Q9HBL7 | PLRKT_HUMAN | 0.26998 | 0.315068748 |
| Q9H488 | OFUT1_HUMAN | 0.27001 | -0.132283483 |
| P46108 | CRK_HUMAN | 0.27115 | -0.232381705 |
| P16401 | H15_HUMAN | 0.27256 | 0.247732049 |
| P02533 | K1C14_HUMAN | 0.27281 | 0.184466121 |
| P62263 | RS14_HUMAN | 0.27312 | 0.094469184 |
| Q9H0W9 | CK054_HUMAN | 0.27331 | -0.109946497 |
| P04264 | K2C1_HUMAN | 0.27495 | 0.435143225 |
| Q8TD55 | PKHO2_HUMAN | 0.27588 | -0.111963061 |
| P13473 | LAMP2_HUMAN | 0.27686 | -0.109594319 |
| P39687 | AN32A_HUMAN | 0.27725 | 0.400781476 |
| Q9BXP5 | SRRT_HUMAN | 0.27761 | -0.177353657 |
| P07954 | FUMH_HUMAN | 0.2789 | 0.088744845 |
| P46783 | RS10_HUMAN | 0.28048 | -0.184919418 |
| P25789 | PSA4_HUMAN | 0.28102 | -0.041036736 |
| Q01518 | CAP1_HUMAN | 0.28126 | -0.047906788 |
| Q15582 | BGH3_HUMAN | 0.28339 | -0.149322635 |
| P61088 | UBE2N_HUMAN | 0.28446 | 0.067800508 |
| Q12797 | ASPH_HUMAN | 0.28739 | -0.156226541 |
| P11047 | LAMC1_HUMAN | 0.28751 | -0.228560829 |
| Q16543 | CDC37_HUMAN | 0.28805 | -0.108962955 |
| P15311 | EZRI_HUMAN | 0.28899 | 0.063765589 |
| Q99729-3 | ROAA_HUMAN | 0.28903 | 0.215116747 |
| P61077 | UB2D3_HUMAN | 0.2903 | -0.219097278 |
| O14737 | PDCD5_HUMAN | 0.29228 | -0.124572872 |
| Q13838-2 | DX39B_HUMAN | 0.29246 | -0.12099791 |
| P13674-3 | P4HA1_HUMAN | 0.2927 | -0.12251944 |
| P62280 | RS11_HUMAN | 0.29278 | 0.149405292 |
| O95236 | APOL3_HUMAN | 0.29291 | -0.235943135 |
| P11166 | GTR1_HUMAN | 0.29317 | 0.158526838 |
| Q8NE71 | ABCF1_HUMAN | 0.29338 | -0.09478928 |
| P43686 | PRS6B_HUMAN | 0.29518 | -0.276585172 |
| Q9H4A4 | AMPB_HUMAN | 0.29556 | -0.111406591 |
| Q9P0J0 | NDUAD_HUMAN | 0.29585 | -0.12084604 |
| P53621 | COPA_HUMAN | 0.29712 | 0.062433665 |
| Q99538 | LGMN_HUMAN | 0.29754 | -0.227298044 |
| P16278 | BGAL_HUMAN | 0.29808 | 0.16007526 |
| P07814 | SYEP_HUMAN | 0.29843 | 0.193971868 |
| P11940 | PABP1_HUMAN | 0.29843 | -0.115744173 |
| Q9Y6G9 | DC1L1_HUMAN | 0.29881 | 0.203830709 |
| P35237 | SPB6_HUMAN | 0.29891 | -0.121492029 |
| P62826 | RAN_HUMAN | 0.30106 | 0.058540957 |
| Q9UQ80 | PA2G4_HUMAN | 0.30134 | -0.140845455 |
| O96008 | TOM40_HUMAN | 0.30227 | 0.108293499 |
| P40925 | MDHC_HUMAN | 0.30229 | -0.056374922 |
| O43760 | SNG2_HUMAN | 0.30249 | -0.103909445 |
| O00487 | PSDE_HUMAN | 0.30299 | 0.061771168 |
| P57053 | H2BFS_HUMAN | 0.30306 | 0.151528211 |
| P35527 | K1C9_HUMAN | 0.30326 | 0.357329194 |
| Q92597 | NDRG1_HUMAN | 0.30351 | -0.099347412 |
| Q9HDC9 | APMAP_HUMAN | 0.30369 | 0.178971215 |
| P47897 | SYQ_HUMAN | 0.30434 | 0.243536937 |
| P61803 | DAD1_HUMAN | 0.30592 | -0.11746854 |
| P61313 | RL15_HUMAN | 0.30603 | 0.126007756 |
| Q9NSA0 | S22AB_HUMAN | 0.3064 | 0.256487879 |
| P0DOX5 | IGG1_HUMAN | 0.30725 | -0.136908712 |
| P41567 | EIF1_HUMAN | 0.30814 | -0.123042106 |
| P50570-2 | DYN2_HUMAN | 0.30839 | -0.221203012 |
| Q00341 | VIGLN_HUMAN | 0.31101 | 0.090426997 |
| Q9BWM7 | SFXN3_HUMAN | 0.31115 | 0.116655245 |
| P61086 | UBE2K_HUMAN | 0.31182 | 0.207505755 |
| Q92734 | TFG_HUMAN | 0.31223 | -0.181498662 |
| P01834 | IGKC_HUMAN | 0.31234 | -0.180902576 |
| P05556 | ITB1_HUMAN | 0.31457 | -0.142382717 |
| P06744 | G6PI_HUMAN | 0.3149 | -0.18426406 |
| Q5K4L6 | S27A3_HUMAN | 0.31806 | -0.156225725 |
| Q13571 | LAPM5_HUMAN | 0.32035 | 0.426326292 |
| Q9H008 | LHPP_HUMAN | 0.32059 | -0.182697691 |
| P68871 | HBB_HUMAN | 0.32065 | -0.272064297 |
| Q04760 | LGUL_HUMAN | 0.32083 | 0.203970016 |
| Q02750 | MP2K1_HUMAN | 0.32105 | -0.235642089 |
| P78344 | IF4G2_HUMAN | 0.32197 | -0.078671384 |
| P55884-2 | EIF3B_HUMAN | 0.32227 | -0.108152963 |
| P18031 | PTN1_HUMAN | 0.32261 | 0.105763504 |
| P51571 | SSRD_HUMAN | 0.3231 | -0.118515063 |
| Q13492 | PICAL_HUMAN | 0.32323 | -0.119412444 |
| P14550 | AK1A1_HUMAN | 0.32336 | 0.099501405 |
| O15533 | TPSN_HUMAN | 0.32463 | -0.181381462 |
| O75534-4 | CSDE1_HUMAN | 0.3258 | 0.098203 |
| Q6PIU2 | NCEH1_HUMAN | 0.32599 | 0.079392612 |
| Q6P587 | FAHD1_HUMAN | 0.32631 | -0.161500068 |
| P50416 | CPT1A_HUMAN | 0.32737 | 0.304832081 |
| P01023 | A2MG_HUMAN | 0.32751 | -0.205606717 |
| Q96C19 | EFHD2_HUMAN | 0.32788 | -0.087983687 |
| Q9H832 | UBE2Z_HUMAN | 0.3285 | -0.1985975 |
| Q8IXQ6 | PARP9_HUMAN | 0.32929 | -0.211536262 |
| Q9P2E9 | RRBP1_HUMAN | 0.32952 | 0.12308836 |
| O60613 | SEP15_HUMAN | 0.3303 | -0.153374046 |
| Q30134 | 2B18_HUMAN | 0.33069 | 0.217801515 |
| Q14019 | COTL1_HUMAN | 0.33092 | -0.054247378 |
| P68363 | TBA1B_HUMAN | 0.33127 | -0.233813237 |
| Q8N2K0 | ABD12_HUMAN | 0.33362 | -0.133713754 |
| P16219 | ACADS_HUMAN | 0.33385 | 0.288199904 |
| P61006 | RAB8A_HUMAN | 0.3344 | -0.143248515 |
| P09661 | RU2A_HUMAN | 0.33457 | -0.097405095 |
| P61923 | COPZ1_HUMAN | 0.33465 | -0.120420839 |
| P52566 | GDIR2_HUMAN | 0.33472 | -0.036697509 |
| P33897 | ABCD1_HUMAN | 0.33506 | 0.159379126 |
| Q8NCN5 | PDPR_HUMAN | 0.33516 | -0.166051469 |
| P05187 | PPB1_HUMAN | 0.33534 | -0.218065673 |
| P04632 | CPNS1_HUMAN | 0.3355 | -0.124704917 |
| O14818 | PSA7_HUMAN | 0.33551 | 0.068404349 |
| P50213 | IDH3A_HUMAN | 0.33552 | -0.119690618 |
| Q16658 | FSCN1_HUMAN | 0.33588 | -0.214320724 |
| P22102 | PUR2_HUMAN | 0.33622 | -0.115332378 |
| Q15363 | TMED2_HUMAN | 0.33707 | -0.145498247 |
| P55084 | ECHB_HUMAN | 0.33734 | -0.193184166 |
| P51003 | PAPOA_HUMAN | 0.33779 | 0.624956864 |
| P63104 | 1433Z_HUMAN | 0.33819 | -0.162917835 |
| P01892 | 1A02_HUMAN | 0.33958 | 0.118612252 |
| Q9H2U2 | IPYR2_HUMAN | 0.34004 | -0.075637631 |
| P06396-2 | GELS_HUMAN | 0.34228 | -0.05442218 |
| O14744-5 | ANM5_HUMAN | 0.34282 | -0.237323228 |
| Q16181 | SEPT7_HUMAN | 0.34285 | 0.249073741 |
| P78537 | BL1S1_HUMAN | 0.34381 | -0.178516237 |
| Q0JRZ9 | FCHO2_HUMAN | 0.3443 | 0.147469074 |
| Q9UHD1 | CHRD1_HUMAN | 0.34462 | 0.143135337 |
| Q16778 | H2B2E_HUMAN | 0.34515 | 0.098555113 |
| P28065 | PSB9_HUMAN | 0.34536 | 0.097796756 |
| P15170-3 | ERF3A_HUMAN | 0.34654 | 0.132430913 |
| O60488 | ACSL4_HUMAN | 0.34682 | -0.123654272 |
| Q16186 | ADRM1_HUMAN | 0.34739 | 0.141735076 |
| Q9UBV8 | PEF1_HUMAN | 0.34804 | -0.493614377 |
| P50995 | ANX11_HUMAN | 0.3486 | 0.272776104 |
| P08575 | PTPRC_HUMAN | 0.34887 | -0.202387714 |
| Q53T59 | H1BP3_HUMAN | 0.34892 | -0.124363413 |
| Q13620 | CUL4B_HUMAN | 0.34997 | -0.154158138 |
| O43920 | NDUS5_HUMAN | 0.35006 | 0.318360072 |
| P42766 | RL35_HUMAN | 0.35006 | 0.086811959 |
| Q9HB71 | CYBP_HUMAN | 0.35008 | 0.08629868 |
| Q92522 | H1X_HUMAN | 0.35133 | 0.105508306 |
| Q14108 | SCRB2_HUMAN | 0.3515 | 0.093973474 |
| Q14254 | FLOT2_HUMAN | 0.35156 | 0.142400051 |
| Q15185 | TEBP_HUMAN | 0.35228 | -0.182864221 |
| Q9Y678 | COPG1_HUMAN | 0.35263 | 0.058261249 |
| P05455 | LA_HUMAN | 0.35327 | -0.17935649 |
| P48047 | ATPO_HUMAN | 0.3561 | 0.114534191 |
| P17980 | PRS6A_HUMAN | 0.35616 | -0.138616796 |
| Q14669 | TRIPC_HUMAN | 0.3566 | 0.163439657 |
| P55769 | NH2L1_HUMAN | 0.35906 | 0.16989538 |
| Q13162 | PRDX4_HUMAN | 0.36068 | -0.124427812 |
| P35268 | RL22_HUMAN | 0.36307 | 0.08675834 |
| Q6P179 | ERAP2_HUMAN | 0.36339 | 0.138462398 |
| P42224 | STAT1_HUMAN | 0.36427 | 0.081018948 |
| Q9UHB9 | SRP68_HUMAN | 0.36553 | 0.081709385 |
| P23434 | GCSH_HUMAN | 0.36595 | 0.256942921 |
| P61970 | NTF2_HUMAN | 0.36623 | -0.124032453 |
| O15260 | SURF4_HUMAN | 0.367 | -0.178453489 |
| Q15833 | STXB2_HUMAN | 0.36711 | 0.076610268 |
| O76013 | KRT36_HUMAN | 0.36711 | 0.688624351 |
| Q7LG56 | RIR2B_HUMAN | 0.36903 | 0.305689013 |
| Q9Y3E0 | GOT1B_HUMAN | 0.36985 | 0.27390231 |
| Q9Y266 | NUDC_HUMAN | 0.37115 | -0.083514092 |
| O00217 | NDUS8_HUMAN | 0.37232 | 0.181075267 |
| B0I1T2 | MYO1G_HUMAN | 0.37354 | 0.082752789 |
| P57740 | NU107_HUMAN | 0.3741 | -0.095758275 |
| Q15691 | MARE1_HUMAN | 0.37454 | 0.167322085 |
| P50991 | TCPD_HUMAN | 0.37503 | -0.093854891 |
| Q6PI78 | TMM65_HUMAN | 0.37588 | -0.135222891 |
| O95197 | RTN3_HUMAN | 0.37768 | -0.125225521 |
| Q06830 | PRDX1_HUMAN | 0.37791 | -0.110227588 |
| P48643 | TCPE_HUMAN | 0.38018 | -0.08857722 |
| Q13576 | IQGA2_HUMAN | 0.38047 | -0.077724062 |
| P32456 | GBP2_HUMAN | 0.38047 | 0.08991594 |
| Q13200 | PSMD2_HUMAN | 0.38082 | 0.099278238 |
| Q9UI12 | VATH_HUMAN | 0.38102 | -0.046407494 |
| Q8TCU6 | PREX1_HUMAN | 0.38211 | -0.205856528 |
| P61224 | RAP1B_HUMAN | 0.38325 | -0.150562545 |
| P62258 | 1433E_HUMAN | 0.38408 | -0.034412133 |
| P63000 | RAC1_HUMAN | 0.38436 | -0.075842397 |
| P06737 | PYGL_HUMAN | 0.38677 | 0.127055263 |
| Q01813 | PFKAP_HUMAN | 0.38719 | 0.344468509 |
| Q16799 | RTN1_HUMAN | 0.38737 | -0.119170731 |
| P13645 | K1C10_HUMAN | 0.38778 | 0.252990506 |
| P62310 | LSM3_HUMAN | 0.38778 | -0.1920571 |
| P78324 | SHPS1_HUMAN | 0.38788 | -0.161130309 |
| P13639 | EF2_HUMAN | 0.38809 | 0.048307487 |
| P43490 | NAMPT_HUMAN | 0.38821 | -0.093810675 |
| P51148 | RAB5C_HUMAN | 0.38884 | 0.172775858 |
| P09211 | GSTP1_HUMAN | 0.38919 | -0.161485504 |
| P14866 | HNRPL_HUMAN | 0.3894 | 0.106801497 |
| Q13445 | TMED1_HUMAN | 0.39126 | -0.171771739 |
| Q5JTV8 | TOIP1_HUMAN | 0.39144 | -0.170833361 |
| P11177 | ODPB_HUMAN | 0.39171 | 0.085982297 |
| P09012 | SNRPA_HUMAN | 0.39406 | -0.152313537 |
| Q93050 | VPP1_HUMAN | 0.39462 | 0.16838565 |
| O00159 | MYO1C_HUMAN | 0.39465 | 0.098423893 |
| P07099 | HYEP_HUMAN | 0.39507 | -0.160430692 |
| P27797 | CALR_HUMAN | 0.39551 | -0.04765584 |
| P01111 | RASN_HUMAN | 0.3964 | -0.117618683 |
| Q9BS26 | ERP44_HUMAN | 0.39663 | -0.063571575 |
| Q04637-8 | IF4G1_HUMAN | 0.39672 | 0.032287874 |
| Q9NT62 | ATG3_HUMAN | 0.39709 | -0.143272584 |
| Q13740 | CD166_HUMAN | 0.39717 | 0.217590348 |
| P12956 | XRCC6_HUMAN | 0.39837 | 0.052266359 |
| P17931 | LEG3_HUMAN | 0.39869 | -0.062261078 |
| O15127 | SCAM2_HUMAN | 0.39975 | 0.080910144 |
| P35908 | K22E_HUMAN | 0.40035 | 0.192361015 |
| Q9NTK5 | OLA1_HUMAN | 0.40196 | -0.07590419 |
| O95833 | CLIC3_HUMAN | 0.40199 | -0.182585548 |
| Q9UHQ9 | NB5R1_HUMAN | 0.40231 | -0.135639154 |
| Q9BWS9 | CHID1_HUMAN | 0.40236 | -0.121729775 |
| Q9H939 | PPIP2_HUMAN | 0.40264 | 0.12451406 |
| Q8N5K1 | CISD2_HUMAN | 0.40296 | 0.170825214 |
| P08708 | RS17_HUMAN | 0.40331 | 0.08213237 |
| P27824 | CALX_HUMAN | 0.40352 | -0.107406552 |
| Q8N766 | EMC1_HUMAN | 0.40431 | -0.119295454 |
| Q99460 | PSMD1_HUMAN | 0.40498 | 0.098721701 |
| P07384 | CAN1_HUMAN | 0.40589 | 0.108718727 |
| P48147 | PPCE_HUMAN | 0.40613 | -0.066427408 |
| Q9NZ08 | ERAP1_HUMAN | 0.40638 | -0.095496758 |
| P78417 | GSTO1_HUMAN | 0.40691 | -0.146858509 |
| P13797-2 | PLST_HUMAN | 0.40697 | -0.124247746 |
| P83111 | LACTB_HUMAN | 0.40848 | -0.194604645 |
| Q16822 | PCKGM_HUMAN | 0.40882 | 0.131804307 |
| Q12905 | ILF2_HUMAN | 0.40891 | -0.120359912 |
| P19525 | E2AK2_HUMAN | 0.40892 | -0.154069776 |
| Q9NX46 | ARHL2_HUMAN | 0.40909 | -0.067683144 |
| P52209 | 6PGD_HUMAN | 0.41016 | 0.063318391 |
| A6NCN2 | KR87P_HUMAN | 0.41025 | 0.62112578 |
| P20340-2 | RAB6A_HUMAN | 0.41112 | 0.09190695 |
| O95340 | PAPS2_HUMAN | 0.41173 | 0.258224229 |
| P09382 | LEG1_HUMAN | 0.41191 | -0.156272759 |
| P05023 | AT1A1_HUMAN | 0.41217 | 0.150404931 |
| P60981 | DEST_HUMAN | 0.41222 | 0.322977106 |
| P61421 | VA0D1_HUMAN | 0.41247 | 0.056006502 |
| Q9Y2Z0 | SGT1_HUMAN | 0.41298 | -0.120193555 |
| P41240 | CSK_HUMAN | 0.41414 | 0.098856688 |
| Q9Y3B3 | TMED7_HUMAN | 0.41415 | 0.050189566 |
| P49448 | DHE4_HUMAN | 0.41447 | 0.05194947 |
| Q9Y3B4 | SF3B6_HUMAN | 0.41598 | 0.08357721 |
| Q14203 | DCTN1_HUMAN | 0.41772 | -0.087398946 |
| O00754 | MA2B1_HUMAN | 0.41839 | 0.312561064 |
| Q15075 | EEA1_HUMAN | 0.42047 | -0.130359142 |
| P08134 | RHOC_HUMAN | 0.42183 | -0.107770604 |
| Q02878 | RL6_HUMAN | 0.42313 | 0.111741718 |
| P05787 | K2C8_HUMAN | 0.42381 | -0.078171428 |
| Q13045 | FLII_HUMAN | 0.42438 | -0.072725291 |
| P05120 | PAI2_HUMAN | 0.42439 | -0.161031158 |
| P11169 | GTR3_HUMAN | 0.42582 | -0.087344205 |
| Q13303-3 | KCAB2_HUMAN | 0.42646 | -0.125175983 |
| Q99832 | TCPH_HUMAN | 0.42687 | -0.132717622 |
| Q15323 | K1H1_HUMAN | 0.42695 | 0.479757781 |
| Q9P2J5 | SYLC_HUMAN | 0.42699 | 0.189241781 |
| Q8NBS9 | TXND5_HUMAN | 0.42742 | -0.162978002 |
| Q9BZQ8 | NIBAN_HUMAN | 0.4275 | -0.071558905 |
| P55160 | NCKPL_HUMAN | 0.42898 | -0.108663168 |
| P15531 | NDKA_HUMAN | 0.42963 | 0.152835174 |
| Q15393 | SF3B3_HUMAN | 0.43046 | 0.042712152 |
| Q96TC7 | RMD3_HUMAN | 0.4305 | -0.147720265 |
| Q9Y570 | PPME1_HUMAN | 0.43123 | 0.317319069 |
| Q13724 | MOGS_HUMAN | 0.43169 | -0.154965927 |
| O95352 | ATG7_HUMAN | 0.43189 | -0.102493908 |
| P28066 | PSA5_HUMAN | 0.4319 | -0.078660267 |
| Q68CZ2 | TENS3_HUMAN | 0.43191 | 0.169600337 |
| P28070 | PSB4_HUMAN | 0.43268 | 0.123309914 |
| O43852 | CALU_HUMAN | 0.4327 | -0.12869832 |
| P06576 | ATPB_HUMAN | 0.43443 | -0.026258107 |
| P06733 | ENOA_HUMAN | 0.43456 | -0.084522622 |
| Q03135 | CAV1_HUMAN | 0.43482 | -0.145629617 |
| A5YKK6 | CNOT1_HUMAN | 0.43734 | 0.208277293 |
| P32455 | GBP1_HUMAN | 0.43754 | 0.138393584 |
| Q15149-4 | PLEC_HUMAN | 0.43761 | -0.117400771 |
| P69905 | HBA_HUMAN | 0.43774 | -0.188098395 |
| P29966 | MARCS_HUMAN | 0.43801 | -0.080370506 |
| O14579 | COPE_HUMAN | 0.43939 | 0.140924025 |
| Q9NZ45 | CISD1_HUMAN | 0.4399 | 0.139409834 |
| P48668 | K2C6C_HUMAN | 0.43991 | 0.180120085 |
| O75844 | FACE1_HUMAN | 0.44224 | -0.110079339 |
| Q9H0D6 | XRN2_HUMAN | 0.44282 | 0.121160203 |
| Q9UMY4-2 | SNX12_HUMAN | 0.44339 | -0.08139829 |
| Q99436 | PSB7_HUMAN | 0.44348 | 0.064762086 |
| P11279 | LAMP1_HUMAN | 0.44488 | 0.113919255 |
| P13647 | K2C5_HUMAN | 0.44497 | 0.169701004 |
| P62495 | ERF1_HUMAN | 0.4457 | 0.145153612 |
| P22234 | PUR6_HUMAN | 0.44582 | 0.118510779 |
| Q96AE4 | FUBP1_HUMAN | 0.44616 | -0.047793005 |
| P15924 | DESP_HUMAN | 0.44685 | 0.13274221 |
| Q86UE4 | LYRIC_HUMAN | 0.44887 | 0.17300916 |
| Q6EEV6 | SUMO4_HUMAN | 0.4505 | -0.07639338 |
| P05164 | PERM_HUMAN | 0.45127 | 0.114907144 |
| P05106 | ITB3_HUMAN | 0.45211 | 0.112815545 |
| O95298 | NDUC2_HUMAN | 0.45334 | 0.245196506 |
| O14828 | SCAM3_HUMAN | 0.45361 | 0.065289278 |
| O43707 | ACTN4_HUMAN | 0.45492 | 0.030023775 |
| Q96RQ9 | OXLA_HUMAN | 0.45946 | -0.081179174 |
| Q9BSJ8 | ESYT1_HUMAN | 0.46034 | 0.137224826 |
| Q7Z434 | MAVS_HUMAN | 0.46286 | 0.131632208 |
| P68402 | PA1B2_HUMAN | 0.46463 | -0.144951882 |
| Q13561 | DCTN2_HUMAN | 0.46487 | 0.081922177 |
| P14317 | HCLS1_HUMAN | 0.46493 | 0.107768604 |
| P08567 | PLEK_HUMAN | 0.46534 | -0.107466169 |
| O95486 | SC24A_HUMAN | 0.4654 | 0.180682973 |
| P30740 | ILEU_HUMAN | 0.46593 | 0.104219989 |
| Q13232 | NDK3_HUMAN | 0.46593 | -0.106087085 |
| Q01085 | TIAR_HUMAN | 0.46595 | 0.151008657 |
| Q00169 | PIPNA_HUMAN | 0.46678 | -0.110040885 |
| P16083 | NQO2_HUMAN | 0.467 | -0.073773526 |
| Q96N66 | MBOA7_HUMAN | 0.46705 | 0.144701535 |
| P11216 | PYGB_HUMAN | 0.46731 | 0.078749875 |
| P19367 | HXK1_HUMAN | 0.46868 | -0.137884085 |
| P07900-2 | HS90A_HUMAN | 0.47021 | 0.028161109 |
| P61513 | RL37A_HUMAN | 0.47049 | 0.103953359 |
| P22897 | MRC1_HUMAN | 0.47208 | 0.151840248 |
| P13804 | ETFA_HUMAN | 0.47261 | -0.119626489 |
| O75643 | U520_HUMAN | 0.47317 | 0.12093643 |
| P50454 | SERPH_HUMAN | 0.47361 | -0.121015084 |
| O43592 | XPOT_HUMAN | 0.4737 | -0.16030987 |
| Q15293 | RCN1_HUMAN | 0.47387 | -0.119244631 |
| P62424 | RL7A_HUMAN | 0.47444 | 0.054158757 |
| Q07812 | BAX_HUMAN | 0.47464 | -0.109445485 |
| O00400 | ACATN_HUMAN | 0.47495 | 0.330240153 |
| P48059-5 | LIMS1_HUMAN | 0.47545 | -0.174464009 |
| O95292 | VAPB_HUMAN | 0.47548 | 0.065505565 |
| Q5VT79 | AXA81_HUMAN | 0.47675 | -0.132292969 |
| O96019 | ACL6A_HUMAN | 0.47881 | 0.082545748 |
| O00231 | PSD11_HUMAN | 0.47909 | -0.074300862 |
| P11310 | ACADM_HUMAN | 0.47926 | -0.140311939 |
| P45877 | PPIC_HUMAN | 0.47927 | -0.111318068 |
| Q7L7X3 | TAOK1_HUMAN | 0.47994 | -0.195640339 |
| O60716 | CTND1_HUMAN | 0.48086 | -0.159457578 |
| Q9Y5Z4 | HEBP2_HUMAN | 0.48105 | -0.152058003 |
| Q00839 | HNRPU_HUMAN | 0.48185 | -0.075580563 |
| Q9NRV9 | HEBP1_HUMAN | 0.48477 | 0.094185318 |
| P25685 | DNJB1_HUMAN | 0.48576 | 0.089840107 |
| P11233 | RALA_HUMAN | 0.48643 | -0.071654689 |
| Q9Y4D7 | PLXD1_HUMAN | 0.48703 | 0.146534768 |
| Q92609 | TBCD5_HUMAN | 0.48885 | 0.07143557 |
| Q9P0V8 | SLAF8_HUMAN | 0.48945 | 0.162359983 |
| Q01082 | SPTB2_HUMAN | 0.49026 | 0.065580295 |
| Q15631 | TSN_HUMAN | 0.49176 | -0.053615132 |
| P25705 | ATPA_HUMAN | 0.4942 | 0.03987061 |
| P52790 | HXK3_HUMAN | 0.49428 | -0.087983499 |
| P08779 | K1C16_HUMAN | 0.49513 | 0.19958567 |
| P37108 | SRP14_HUMAN | 0.49613 | -0.084940281 |
| P13716 | HEM2_HUMAN | 0.49633 | 0.081014471 |
| Q9BRF8 | CPPED_HUMAN | 0.49727 | -0.071235377 |
| P21980 | TGM2_HUMAN | 0.50001 | 0.105561873 |
| P38571 | LICH_HUMAN | 0.50013 | -0.143953405 |
| Q09666 | AHNK_HUMAN | 0.50072 | 0.083591382 |
| O14773 | TPP1_HUMAN | 0.50083 | -0.114129259 |
| Q9UL25 | RAB21_HUMAN | 0.50122 | 0.051080404 |
| P02751 | FINC_HUMAN | 0.50167 | -0.110301298 |
| P09429 | HMGB1_HUMAN | 0.50172 | -0.144441819 |
| P12724 | ECP_HUMAN | 0.5019 | -0.162400069 |
| Q96A72 | MGN2_HUMAN | 0.50219 | -0.077032 |
| Q14204 | DYHC1_HUMAN | 0.50332 | 0.050399929 |
| Q9Y490 | TLN1_HUMAN | 0.5039 | -0.049454763 |
| P29466 | CASP1_HUMAN | 0.50397 | -0.045392511 |
| P60033 | CD81_HUMAN | 0.50469 | -0.18030709 |
| P57088 | TMM33_HUMAN | 0.50486 | 0.33333329 |
| Q16630 | CPSF6_HUMAN | 0.50575 | -0.076743397 |
| P07237 | PDIA1_HUMAN | 0.50695 | -0.044932356 |
| Q15417 | CNN3_HUMAN | 0.50906 | -0.12085678 |
| P09104 | ENOG_HUMAN | 0.51209 | -0.095043334 |
| P35222 | CTNB1_HUMAN | 0.51265 | 0.08100557 |
| P51665 | PSMD7_HUMAN | 0.51317 | -0.108811012 |
| Q16629 | SRSF7_HUMAN | 0.51368 | -0.101274436 |
| P11215 | ITAM_HUMAN | 0.51523 | 0.093125155 |
| O60506 | HNRPQ_HUMAN | 0.51539 | 0.050904805 |
| P53396 | ACLY_HUMAN | 0.51556 | -0.033530576 |
| P00387-3 | NB5R3_HUMAN | 0.51586 | 0.085098128 |
| P23368 | MAOM_HUMAN | 0.51662 | 0.080705232 |
| P30050 | RL12_HUMAN | 0.5167 | -0.042509055 |
| P35241 | RADI_HUMAN | 0.51827 | -0.05849624 |
| P51159 | RB27A_HUMAN | 0.51905 | 0.078981611 |
| Q9HB90 | RRAGC_HUMAN | 0.51942 | -0.084197519 |
| P51812 | KS6A3_HUMAN | 0.52018 | 0.114128141 |
| P60900 | PSA6_HUMAN | 0.52027 | -0.06601043 |
| O00571 | DDX3X_HUMAN | 0.52049 | -0.090741492 |
| P35610 | SOAT1_HUMAN | 0.52109 | 0.024088151 |
| P09525 | ANXA4_HUMAN | 0.52122 | -0.124390033 |
| O15144 | ARPC2_HUMAN | 0.52267 | 0.078221637 |
| P19404 | NDUV2_HUMAN | 0.52278 | -0.072279072 |
| Q13642 | FHL1_HUMAN | 0.52279 | 0.306260131 |
| P60953 | CDC42_HUMAN | 0.52378 | 0.049236429 |
| P49720 | PSB3_HUMAN | 0.5238 | -0.086718247 |
| P49721 | PSB2_HUMAN | 0.52432 | -0.109234049 |
| O75083 | WDR1_HUMAN | 0.52645 | -0.052709268 |
| P53004 | BIEA_HUMAN | 0.52652 | -0.08867668 |
| P02786 | TFR1_HUMAN | 0.52776 | 0.100513158 |
| Q13813 | SPTN1_HUMAN | 0.52801 | 0.139362669 |
| Q9UJ70-2 | NAGK_HUMAN | 0.52837 | -0.075565088 |
| P05121 | PAI1_HUMAN | 0.5289 | 0.144947706 |
| Q9UHD8 | SEPT9_HUMAN | 0.52936 | 0.045835357 |
| Q9BV40 | VAMP8_HUMAN | 0.52955 | 0.087702701 |
| P13498 | CY24A_HUMAN | 0.53056 | 0.136120512 |
| P31939 | PUR9_HUMAN | 0.53059 | -0.081267807 |
| P02792 | FRIL_HUMAN | 0.53144 | -0.16940387 |
| Q16563-2 | SYPL1_HUMAN | 0.533 | -0.10325652 |
| P26373 | RL13_HUMAN | 0.53363 | 0.103249828 |
| P35998 | PRS7_HUMAN | 0.53401 | -0.056089773 |
| Q71UM5 | RS27L_HUMAN | 0.53468 | 0.064830007 |
| Q16134 | ETFD_HUMAN | 0.53534 | 0.057027955 |
| P02649 | APOE_HUMAN | 0.53692 | 0.08137306 |
| O14880 | MGST3_HUMAN | 0.53885 | 0.096834762 |
| Q13885 | TBB2A_HUMAN | 0.53918 | -0.089320584 |
| Q16795 | NDUA9_HUMAN | 0.53928 | -0.097987764 |
| P35625 | TIMP3_HUMAN | 0.5393 | -0.152832714 |
| Q7L5L3 | GDPD3_HUMAN | 0.54085 | -0.093954548 |
| O00469 | PLOD2_HUMAN | 0.54124 | 0.278676088 |
| Q9NSB4 | KRT82_HUMAN | 0.54382 | 0.287598729 |
| P04216 | THY1_HUMAN | 0.5448 | -0.112980263 |
| Q92900 | RENT1_HUMAN | 0.54627 | 0.101865947 |
| Q86Y39 | NDUAB_HUMAN | 0.54745 | -0.130512908 |
| P14060 | 3BHS1_HUMAN | 0.54761 | -0.11368007 |
| P22314 | UBA1_HUMAN | 0.54958 | -0.027800475 |
| P50440 | GATM_HUMAN | 0.54962 | -0.133951209 |
| Q9Y241 | HIG1A_HUMAN | 0.54972 | 0.13172914 |
| P52788 | SPSY_HUMAN | 0.54983 | -0.08341464 |
| P48444 | COPD_HUMAN | 0.55014 | 0.113225979 |
| Q8N163 | CCAR2_HUMAN | 0.55015 | -0.074478822 |
| Q9Y2R0 | COA3_HUMAN | 0.55023 | -0.094438975 |
| Q08752 | PPID_HUMAN | 0.55033 | -0.066682345 |
| Q8WVM8 | SCFD1_HUMAN | 0.55151 | 0.06469049 |
| Q9Y5S9 | RBM8A_HUMAN | 0.55179 | -0.082468088 |
| P00505 | AATM_HUMAN | 0.55215 | -0.093365596 |
| Q96FQ6 | S10AG_HUMAN | 0.55373 | 0.136970885 |
| P62851 | RS25_HUMAN | 0.55467 | 0.07159844 |
| P20674 | COX5A_HUMAN | 0.55537 | -0.07351985 |
| Q9H223 | EHD4_HUMAN | 0.55595 | -0.058339534 |
| Q9P0L0 | VAPA_HUMAN | 0.55656 | -0.025079132 |
| O94979 | SC31A_HUMAN | 0.55666 | 0.056279899 |
| Q9UKK9 | NUDT5_HUMAN | 0.55768 | -0.072405553 |
| Q86YN1 | DOPP1_HUMAN | 0.55771 | -0.078557664 |
| P50135 | HNMT_HUMAN | 0.55785 | -0.066991602 |
| Q9NVI7 | ATD3A_HUMAN | 0.55942 | -0.15934193 |
| P27694 | RFA1_HUMAN | 0.56013 | 0.109573984 |
| Q92616 | GCN1_HUMAN | 0.56034 | 0.163519257 |
| P84095 | RHOG_HUMAN | 0.56065 | 0.026103902 |
| P20618 | PSB1_HUMAN | 0.56147 | 0.03128681 |
| O43790 | KRT86_HUMAN | 0.5615 | 0.163844226 |
| P30040 | ERP29_HUMAN | 0.56162 | -0.082808658 |
| O95831 | AIFM1_HUMAN | 0.56283 | -0.067856919 |
| O15145 | ARPC3_HUMAN | 0.56297 | -0.029641122 |
| Q16836 | HCDH_HUMAN | 0.56366 | -0.10497011 |
| Q9NQ88 | TIGAR_HUMAN | 0.56508 | -0.07253401 |
| P14314 | GLU2B_HUMAN | 0.56509 | -0.046680315 |
| P20591 | MX1_HUMAN | 0.56604 | 0.105046083 |
| Q12906-7 | ILF3_HUMAN | 0.56624 | 0.075683471 |
| P02747 | C1QC_HUMAN | 0.56635 | 0.066647769 |
| Q5EB52 | MEST_HUMAN | 0.5665 | 0.12109879 |
| O60784 | TOM1_HUMAN | 0.5666 | -0.132686235 |
| P53990 | IST1_HUMAN | 0.56907 | 0.06921031 |
| P19105 | ML12A_HUMAN | 0.5691 | -0.042434385 |
| P08246 | ELNE_HUMAN | 0.57048 | -0.091036177 |
| Q99961 | SH3G1_HUMAN | 0.57221 | -0.062330491 |
| P17813 | EGLN_HUMAN | 0.5733 | -0.095767283 |
| P27986 | P85A_HUMAN | 0.57338 | -0.105320582 |
| Q14677 | EPN4_HUMAN | 0.57338 | 0.100576236 |
| P38117 | ETFB_HUMAN | 0.57393 | -0.04234108 |
| P19338 | NUCL_HUMAN | 0.57415 | 0.034090657 |
| P12004 | PCNA_HUMAN | 0.57568 | 0.057738482 |
| O43324 | MCA3_HUMAN | 0.57571 | 0.055885747 |
| Q9Y5M8 | SRPRB_HUMAN | 0.57588 | 0.158028785 |
| Q96BN8 | OTUL_HUMAN | 0.57604 | 0.136258084 |
| P12429 | ANXA3_HUMAN | 0.57852 | 0.097111986 |
| Q96C86 | DCPS_HUMAN | 0.57895 | -0.073338741 |
| Q9Y4L1 | HYOU1_HUMAN | 0.57908 | 0.043485976 |
| Q7Z3C6 | ATG9A_HUMAN | 0.58067 | 0.103811133 |
| O15498 | YKT6_HUMAN | 0.58078 | 0.038406594 |
| Q86VS8 | HOOK3_HUMAN | 0.58211 | -0.108527055 |
| P01040 | CYTA_HUMAN | 0.5833 | 0.094923223 |
| O15162 | PLS1_HUMAN | 0.58391 | 0.14978439 |
| Q9UJZ1 | STML2_HUMAN | 0.58524 | 0.130355115 |
| Q14764 | MVP_HUMAN | 0.58564 | 0.060822893 |
| P47985 | UCRI_HUMAN | 0.58564 | -0.075370537 |
| O15551 | CLD3_HUMAN | 0.58616 | -0.114165357 |
| Q04837 | SSBP_HUMAN | 0.5863 | 0.089591069 |
| Q9Y2Q5 | LTOR2_HUMAN | 0.58648 | 0.103980632 |
| P61163 | ACTZ_HUMAN | 0.58668 | -0.140164185 |
| O00160 | MYO1F_HUMAN | 0.58746 | 0.0963468 |
| O60762 | DPM1_HUMAN | 0.58818 | 0.114775815 |
| P09960 | LKHA4_HUMAN | 0.58873 | 0.100533227 |
| P80217 | IN35_HUMAN | 0.58958 | -0.077869995 |
| P09669 | COX6C_HUMAN | 0.58984 | 0.063129067 |
| P55008 | AIF1_HUMAN | 0.59108 | -0.102779772 |
| Q9BRA2 | TXD17_HUMAN | 0.59145 | -0.05308708 |
| O95479 | G6PE_HUMAN | 0.59192 | 0.154127846 |
| P52565 | GDIR1_HUMAN | 0.5929 | 0.030243586 |
| P51452 | DUS3_HUMAN | 0.59494 | -0.063376501 |
| Q31610 | 1B81_HUMAN | 0.59529 | -0.094471691 |
| Q9Y6M9 | NDUB9_HUMAN | 0.59587 | -0.113995162 |
| Q05682 | CALD1_HUMAN | 0.59679 | 0.096447817 |
| P10515 | ODP2_HUMAN | 0.59758 | -0.059038211 |
| P36776 | LONM_HUMAN | 0.59778 | -0.051023937 |
| P25786 | PSA1_HUMAN | 0.59825 | 0.09194729 |
| Q969H8 | MYDGF_HUMAN | 0.59943 | 0.06719632 |
| P02794 | FRIH_HUMAN | 0.59948 | 0.05117116 |
| Q96AC1-3 | FERM2_HUMAN | 0.60033 | -0.075344056 |
| Q9NWV8 | BABA1_HUMAN | 0.60534 | -0.12821454 |
| P49588 | SYAC_HUMAN | 0.6055 | -0.059255383 |
| Q02218 | ODO1_HUMAN | 0.60645 | -0.060530965 |
| P80723 | BASP1_HUMAN | 0.6073 | 0.046647274 |
| P48426 | PI42A_HUMAN | 0.6078 | -0.074932016 |
| P61160 | ARP2_HUMAN | 0.60815 | 0.065695452 |
| Q9H8H3 | MET7A_HUMAN | 0.60832 | 0.081922487 |
| P43304 | GPDM_HUMAN | 0.60833 | 0.026673203 |
| P29218 | IMPA1_HUMAN | 0.60884 | -0.06124167 |
| Q00577 | PURA_HUMAN | 0.60922 | -0.084080272 |
| Q9NX63 | MIC19_HUMAN | 0.61018 | 0.070535916 |
| Q8NBJ5 | GT251_HUMAN | 0.61101 | 0.096805507 |
| P12830 | CADH1_HUMAN | 0.61186 | 0.050405976 |
| O43776 | SYNC_HUMAN | 0.61245 | 0.06226473 |
| P35613-2 | BASI_HUMAN | 0.61261 | 0.025263304 |
| P48735 | IDHP_HUMAN | 0.61297 | -0.091713002 |
| Q15836 | VAMP3_HUMAN | 0.61509 | 0.031783657 |
| P55036 | PSMD4_HUMAN | 0.61567 | 0.059613947 |
| P01903 | DRA_HUMAN | 0.61579 | -0.066872389 |
| Q99873 | ANM1_HUMAN | 0.61627 | 0.077111047 |
| Q6YN16 | HSDL2_HUMAN | 0.61705 | -0.100406848 |
| P00403 | COX2_HUMAN | 0.61744 | 0.063298949 |
| Q92945 | FUBP2_HUMAN | 0.61746 | 0.032514088 |
| P62316 | SMD2_HUMAN | 0.61785 | 0.047450742 |
| P62269 | RS18_HUMAN | 0.61951 | -0.046033143 |
| P07305 | H10_HUMAN | 0.61952 | 0.111237031 |
| Q9NVD7-2 | PARVA_HUMAN | 0.6198 | 0.114180744 |
| O60493 | SNX3_HUMAN | 0.62082 | -0.053424627 |
| Q9NUV9 | GIMA4_HUMAN | 0.6209 | 0.052290222 |
| Q8N423 | LIRB2_HUMAN | 0.62118 | 0.124029114 |
| Q9Y5X3 | SNX5_HUMAN | 0.62145 | -0.085897677 |
| Q15942 | ZYX_HUMAN | 0.62272 | 0.156936522 |
| P62333 | PRS10_HUMAN | 0.6241 | -0.071229259 |
| Q13637 | RAB32_HUMAN | 0.62506 | -0.074633338 |
| P02545 | LMNA_HUMAN | 0.62591 | 0.056007715 |
| O60749 | SNX2_HUMAN | 0.62635 | 0.121666556 |
| Q6NYC8 | PPR18_HUMAN | 0.62793 | 0.143601125 |
| Q9BVK6 | TMED9_HUMAN | 0.62828 | -0.048879578 |
| P62266 | RS23_HUMAN | 0.6288 | -0.062034242 |
| O75390 | CISY_HUMAN | 0.62951 | -0.032924453 |
| P46459 | NSF_HUMAN | 0.62965 | 0.059829441 |
| Q9P2I0 | CPSF2_HUMAN | 0.62968 | 0.082643417 |
| P51858 | HDGF_HUMAN | 0.63062 | -0.063704892 |
| P47756-2 | CAPZB_HUMAN | 0.63087 | 0.025926135 |
| P31153 | METK2_HUMAN | 0.63351 | 0.043572223 |
| Q5JWF2 | GNAS1_HUMAN | 0.63385 | 0.063024403 |
| P10619 | PPGB_HUMAN | 0.63478 | -0.092014269 |
| O43684 | BUB3_HUMAN | 0.63592 | 0.080754393 |
| Q8TBQ9 | KISHA_HUMAN | 0.63593 | 0.05942317 |
| Q06323 | PSME1_HUMAN | 0.63687 | 0.089179069 |
| P10253 | LYAG_HUMAN | 0.63763 | -0.043415628 |
| Q99426 | TBCB_HUMAN | 0.63812 | 0.092716912 |
| Q16853 | AOC3_HUMAN | 0.63865 | -0.091757213 |
| Q15008 | PSMD6_HUMAN | 0.63889 | 0.108253292 |
| Q15388 | TOM20_HUMAN | 0.63893 | 0.101186856 |
| P12532 | KCRU_HUMAN | 0.64013 | 0.094681044 |
| Q15274 | NADC_HUMAN | 0.64107 | 0.116903177 |
| Q99714 | HCD2_HUMAN | 0.64229 | -0.035446157 |
| Q92575 | UBXN4_HUMAN | 0.6426 | -0.087514375 |
| Q05086 | UBE3A_HUMAN | 0.64398 | 0.061923573 |
| P36222 | CH3L1_HUMAN | 0.64483 | 0.084573595 |
| Q9UHY7 | ENOPH_HUMAN | 0.64518 | 0.144868145 |
| P16671 | CD36_HUMAN | 0.64582 | -0.058807871 |
| O75955 | FLOT1_HUMAN | 0.64634 | 0.093432721 |
| P18077 | RL35A_HUMAN | 0.64655 | 0.069666346 |
| P38159 | RBMX_HUMAN | 0.64814 | 0.129232393 |
| P13928 | ANXA8_HUMAN | 0.64894 | -0.093827015 |
| P27105 | STOM_HUMAN | 0.64973 | 0.034109175 |
| Q9UNZ2 | NSF1C_HUMAN | 0.65004 | 0.102290638 |
| Q9NQW7 | XPP1_HUMAN | 0.6516 | 0.023171419 |
| P59998 | ARPC4_HUMAN | 0.65175 | 0.023124937 |
| Q5VYY1 | ANR22_HUMAN | 0.65214 | 0.102703416 |
| P13987-2 | CD59_HUMAN | 0.65264 | 0.03895112 |
| Q15019 | SEPT2_HUMAN | 0.65272 | -0.05454527 |
| P07686 | HEXB_HUMAN | 0.65383 | 0.086426189 |
| O94856 | NFASC_HUMAN | 0.65384 | 0.1944246 |
| Q01469 | FABP5_HUMAN | 0.65395 | 0.064687053 |
| P07437 | TBB5_HUMAN | 0.65408 | -0.077748898 |
| Q9Y4K1 | CRBG1_HUMAN | 0.65454 | 0.088998382 |
| P00492 | HPRT_HUMAN | 0.65471 | 0.082295751 |
| Q00610 | CLH1_HUMAN | 0.65477 | 0.033028185 |
| O43676 | NDUB3_HUMAN | 0.65479 | -0.067374426 |
| P62241 | RS8_HUMAN | 0.65497 | 0.04996137 |
| P04062 | GLCM_HUMAN | 0.65587 | -0.067378818 |
| P07355 | ANXA2_HUMAN | 0.6571 | -0.053038221 |
| Q9UIJ7 | KAD3_HUMAN | 0.65764 | -0.078749514 |
| P62805 | H4_HUMAN | 0.65904 | 0.074176367 |
| Q9BTV4 | TMM43_HUMAN | 0.66199 | -0.071927704 |
| Q9Y2J2 | E41L3_HUMAN | 0.66274 | -0.027548755 |
| P08758 | ANXA5_HUMAN | 0.66326 | 0.034728006 |
| O75396 | SC22B_HUMAN | 0.66332 | -0.031677685 |
| Q9NUQ9 | FA49B_HUMAN | 0.66626 | -0.019079256 |
| Q6RW13 | ATRAP_HUMAN | 0.66772 | 0.040569801 |
| Q05655 | KPCD_HUMAN | 0.66861 | 0.208602038 |
| P63162 | RSMN_HUMAN | 0.66898 | -0.072594144 |
| P43034 | LIS1_HUMAN | 0.66995 | 0.049463407 |
| P10809 | CH60_HUMAN | 0.6701 | 0.045812651 |
| P00338 | LDHA_HUMAN | 0.67016 | 0.02651937 |
| P60842 | IF4A1_HUMAN | 0.6724 | -0.059187506 |
| P05783 | K1C18_HUMAN | 0.67371 | -0.045117637 |
| Q9Y276 | BCS1_HUMAN | 0.67413 | 0.077889417 |
| P18206 | VINC_HUMAN | 0.67624 | 0.052870226 |
| P06753-2 | TPM3_HUMAN | 0.67693 | -0.038551265 |
| P09110 | THIK_HUMAN | 0.67844 | 0.066794163 |
| P22626 | ROA2_HUMAN | 0.67868 | 0.068007424 |
| P59666 | DEF3_HUMAN | 0.67888 | -0.135187618 |
| O60664 | PLIN3_HUMAN | 0.67933 | -0.070573397 |
| P19971 | TYPH_HUMAN | 0.67974 | -0.071988076 |
| P22695 | QCR2_HUMAN | 0.68117 | 0.067022162 |
| P79483 | DRB3_HUMAN | 0.68122 | -0.055410443 |
| Q9Y2V2 | CHSP1_HUMAN | 0.68124 | -0.080892226 |
| P06730-2 | IF4E_HUMAN | 0.68156 | -0.039120179 |
| Q5SSJ5 | HP1B3_HUMAN | 0.68158 | -0.057584121 |
| P20339 | RAB5A_HUMAN | 0.68176 | 0.052847548 |
| Q9Y2Q3 | GSTK1_HUMAN | 0.68186 | 0.02874341 |
| P00488 | F13A_HUMAN | 0.68187 | -0.109162791 |
| Q0VD83 | APOBR_HUMAN | 0.68219 | 0.103705275 |
| P09417 | DHPR_HUMAN | 0.68231 | -0.036389911 |
| P20700 | LMNB1_HUMAN | 0.68254 | 0.043720563 |
| P04114 | APOB_HUMAN | 0.68257 | -0.059848263 |
| Q15006 | EMC2_HUMAN | 0.68427 | 0.039858249 |
| Q9UIQ6 | LCAP_HUMAN | 0.68816 | 0.082918569 |
| Q15366 | PCBP2_HUMAN | 0.69025 | -0.073945487 |
| A6NHR9 | SMHD1_HUMAN | 0.6923 | -0.066373303 |
| Q02790 | FKBP4_HUMAN | 0.69253 | -0.042930216 |
| P61247 | RS3A_HUMAN | 0.69303 | 0.065676452 |
| O75165 | DJC13_HUMAN | 0.6944 | 0.069978953 |
| P53007 | TXTP_HUMAN | 0.69465 | 0.090763972 |
| P05165 | PCCA_HUMAN | 0.69475 | -0.045830775 |
| P49591 | SYSC_HUMAN | 0.69498 | -0.025041912 |
| Q15717 | ELAV1_HUMAN | 0.69854 | 0.035972766 |
| P49755 | TMEDA_HUMAN | 0.69863 | 0.057929341 |
| P20810-9 | ICAL_HUMAN | 0.69945 | -0.070866297 |
| Q5RI15 | COX20_HUMAN | 0.69958 | -0.067850758 |
| Q9NPH2 | INO1_HUMAN | 0.70001 | 0.061369288 |
| Q8TCT9 | HM13_HUMAN | 0.70097 | -0.128270097 |
| P62913 | RL11_HUMAN | 0.70248 | -0.031692633 |
| O76009 | KT33A_HUMAN | 0.70662 | 0.125860173 |
| Q9Y371-2 | SHLB1_HUMAN | 0.70846 | 0.055477839 |
| O14980 | XPO1_HUMAN | 0.70909 | -0.056293884 |
| P84243 | H33_HUMAN | 0.71118 | 0.079597384 |
| P25774 | CATS_HUMAN | 0.71127 | -0.037107543 |
| P35221 | CTNA1_HUMAN | 0.71213 | -0.073242598 |
| P53999 | TCP4_HUMAN | 0.71316 | 0.05301621 |
| P60983 | GMFB_HUMAN | 0.71338 | -0.060361116 |
| P30457 | 1A66_HUMAN | 0.71393 | -0.100360427 |
| Q9UKK3 | PARP4_HUMAN | 0.71404 | 0.030309269 |
| P17987 | TCPA_HUMAN | 0.71505 | 0.021481222 |
| Q14974 | IMB1_HUMAN | 0.71529 | -0.014307163 |
| Q8IV08 | PLD3_HUMAN | 0.7153 | 0.035011295 |
| P16435 | NCPR_HUMAN | 0.71747 | -0.041922261 |
| P21397 | AOFA_HUMAN | 0.71967 | -0.058519754 |
| O60568 | PLOD3_HUMAN | 0.72061 | 0.029153321 |
| O15173 | PGRC2_HUMAN | 0.72185 | 0.053078019 |
| P30481 | 1B44_HUMAN | 0.72294 | 0.060056523 |
| Q9H1C4 | UN93B_HUMAN | 0.72306 | -0.07660794 |
| Q9Y584 | TIM22_HUMAN | 0.72354 | 0.097457264 |
| P08729 | K2C7_HUMAN | 0.72598 | -0.047699909 |
| O15400 | STX7_HUMAN | 0.72709 | -0.034930827 |
| P63244 | RACK1_HUMAN | 0.72813 | -0.087791602 |
| O94826 | TOM70_HUMAN | 0.72828 | -0.067397196 |
| Q15181 | IPYR_HUMAN | 0.72854 | -0.032720379 |
| P02679 | FIBG_HUMAN | 0.72862 | -0.053594556 |
| P35232 | PHB_HUMAN | 0.72913 | 0.021794865 |
| P78386 | KRT85_HUMAN | 0.73096 | 0.120459813 |
| Q9UHD9 | UBQL2_HUMAN | 0.73132 | 0.065257164 |
| P61981 | 1433G_HUMAN | 0.73329 | 0.021765241 |
| P31930 | QCR1_HUMAN | 0.73351 | 0.034375154 |
| P14174 | MIF_HUMAN | 0.73424 | -0.059110735 |
| P56199 | ITA1_HUMAN | 0.7355 | 0.078143403 |
| P62701 | RS4X_HUMAN | 0.73627 | -0.031837394 |
| P67870 | CSK2B_HUMAN | 0.73799 | 0.052880777 |
| Q10567 | AP1B1_HUMAN | 0.73891 | -0.070980493 |
| P60866 | RS20_HUMAN | 0.74038 | 0.018315498 |
| Q7Z7H5 | TMED4_HUMAN | 0.7421 | -0.039249911 |
| Q9UBE0 | SAE1_HUMAN | 0.74246 | -0.078255933 |
| P35611 | ADDA_HUMAN | 0.74266 | 0.043471972 |
| Q00765 | REEP5_HUMAN | 0.74466 | 0.032674916 |
| P42167 | LAP2B_HUMAN | 0.74486 | -0.031257575 |
| Q71UI9 | H2AV_HUMAN | 0.74521 | -0.037633798 |
| Q9BT09 | CNPY3_HUMAN | 0.7463 | -0.028080266 |
| Q9Y6K5 | OAS3_HUMAN | 0.74844 | 0.055626802 |
| Q93008 | USP9X_HUMAN | 0.74863 | 0.033883877 |
| O43169 | CYB5B_HUMAN | 0.7505 | -0.034005606 |
| Q06210 | GFPT1_HUMAN | 0.75209 | 0.059076862 |
| P20042 | IF2B_HUMAN | 0.75229 | -0.066255944 |
| Q04917 | 1433F_HUMAN | 0.75252 | 0.02080084 |
| Q14914-2 | PTGR1_HUMAN | 0.75306 | -0.062633188 |
| P31943 | HNRH1_HUMAN | 0.75332 | 0.031403445 |
| P52272 | HNRPM_HUMAN | 0.75503 | -0.030154412 |
| P07339 | CATD_HUMAN | 0.75551 | -0.028422458 |
| Q96PP8 | GBP5_HUMAN | 0.7559 | 0.096322221 |
| P21283 | VATC1_HUMAN | 0.757 | -0.042308592 |
| Q96A65 | EXOC4_HUMAN | 0.75715 | -0.059979065 |
| P04844 | RPN2_HUMAN | 0.7574 | -0.017242484 |
| Q15041 | AR6P1_HUMAN | 0.75744 | -0.049292889 |
| P53634 | CATC_HUMAN | 0.75785 | -0.033456407 |
| O43488 | ARK72_HUMAN | 0.75825 | -0.04774439 |
| Q9UFN0 | NPS3A_HUMAN | 0.75877 | -0.043079221 |
| Q14258 | TRI25_HUMAN | 0.75903 | 0.056149653 |
| Q13155 | AIMP2_HUMAN | 0.75976 | -0.072500992 |
| Q15165 | PON2_HUMAN | 0.76012 | -0.082475732 |
| Q15661 | TRYB1_HUMAN | 0.76217 | -0.052094455 |
| P39023 | RL3_HUMAN | 0.76266 | 0.077241973 |
| P35573 | GDE_HUMAN | 0.76311 | 0.040828206 |
| P04229 | 2B11_HUMAN | 0.76354 | 0.070307571 |
| O75695 | XRP2_HUMAN | 0.76407 | 0.048425585 |
| Q8WUM4 | PDC6I_HUMAN | 0.76413 | -0.03196889 |
| Q05209 | PTN12_HUMAN | 0.7645 | -0.033706775 |
| P99999 | CYC_HUMAN | 0.76535 | -0.017591327 |
| P09651 | ROA1_HUMAN | 0.76753 | -0.053278092 |
| Q5JPE7 | NOMO2_HUMAN | 0.76846 | -0.044323248 |
| P12277 | KCRB_HUMAN | 0.77012 | -0.047261779 |
| P41250 | GARS_HUMAN | 0.77154 | 0.039720708 |
| P42126 | ECI1_HUMAN | 0.77189 | -0.048454027 |
| P09455 | RET1_HUMAN | 0.77303 | 0.03205846 |
| P84098 | RL19_HUMAN | 0.77383 | 0.051070955 |
| P62879 | GBB2_HUMAN | 0.77534 | 0.022366082 |
| P17693-5 | HLAG_HUMAN | 0.77556 | 0.082381137 |
| P31689 | DNJA1_HUMAN | 0.77562 | -0.015069855 |
| P10620 | MGST1_HUMAN | 0.77566 | 0.049441196 |
| P29350 | PTN6_HUMAN | 0.77608 | -0.02307445 |
| O94973 | AP2A2_HUMAN | 0.77675 | 0.052627846 |
| Q9ULV4 | COR1C_HUMAN | 0.77714 | -0.050426416 |
| P53680 | AP2S1_HUMAN | 0.77785 | 0.020163874 |
| Q86VP6 | CAND1_HUMAN | 0.77898 | 0.01247702 |
| Q5BJH7 | YIF1B_HUMAN | 0.7793 | -0.053183659 |
| P61019 | RAB2A_HUMAN | 0.78124 | 0.010813586 |
| Q6P2Q9 | PRP8_HUMAN | 0.78151 | 0.046095553 |
| Q9HD45 | TM9S3_HUMAN | 0.782 | -0.020593591 |
| P61916 | NPC2_HUMAN | 0.78215 | -0.043234748 |
| P84074 | HPCA_HUMAN | 0.78729 | 0.055901483 |
| O75116 | ROCK2_HUMAN | 0.78807 | -0.031677606 |
| Q13126 | MTAP_HUMAN | 0.78958 | -0.023681301 |
| P35914 | HMGCL_HUMAN | 0.78975 | -0.076800789 |
| Q9BUJ2 | HNRL1_HUMAN | 0.79132 | -0.02778198 |
| Q9UNL2 | SSRG_HUMAN | 0.79262 | -0.028464763 |
| Q53GQ0 | DHB12_HUMAN | 0.79508 | 0.036668365 |
| Q99541 | PLIN2_HUMAN | 0.79518 | -0.04854093 |
| Q9Y3Z3 | SAMH1_HUMAN | 0.79764 | 0.060281377 |
| P05109 | S10A8_HUMAN | 0.79828 | -0.018850861 |
| P23246 | SFPQ_HUMAN | 0.7983 | 0.041707763 |
| P13073 | COX41_HUMAN | 0.79906 | 0.039634277 |
| P08574 | CY1_HUMAN | 0.79913 | 0.039922219 |
| Q99735 | MGST2_HUMAN | 0.79978 | -0.037700207 |
| P62820 | RAB1A_HUMAN | 0.7999 | -0.032804587 |
| P54819 | KAD2_HUMAN | 0.8 | 0.112811108 |
| Q9BZF1 | OSBL8_HUMAN | 0.80017 | 0.050446445 |
| Q9BVC6 | TM109_HUMAN | 0.80115 | 0.017410685 |
| Q9BQA1 | MEP50_HUMAN | 0.80148 | -0.041510554 |
| P80297 | MT1X_HUMAN | 0.80154 | 0.062371929 |
| P23141 | EST1_HUMAN | 0.802 | 0.050831477 |
| Q16698 | DECR_HUMAN | 0.80305 | -0.035624641 |
| Q15738 | NSDHL_HUMAN | 0.80406 | 0.047982868 |
| P45974 | UBP5_HUMAN | 0.80553 | 0.022898433 |
| P55039 | DRG2_HUMAN | 0.80604 | -0.04547448 |
| P27708 | PYR1_HUMAN | 0.80621 | -0.03691832 |
| Q08257 | QOR_HUMAN | 0.80666 | -0.038237211 |
| P30084 | ECHM_HUMAN | 0.80775 | -0.022853049 |
| O43396 | TXNL1_HUMAN | 0.80874 | 0.013509454 |
| Q9UKM9 | RALY_HUMAN | 0.80932 | 0.083113491 |
| Q9Y3C8 | UFC1_HUMAN | 0.81018 | 0.029471164 |
| P68371 | TBB4B_HUMAN | 0.81244 | 0.011733316 |
| P29401 | TKT_HUMAN | 0.81304 | -0.013020435 |
| P08648 | ITA5_HUMAN | 0.81372 | 0.038514716 |
| Q9NYU2 | UGGG1_HUMAN | 0.81419 | -0.023947377 |
| Q9BQB6 | VKOR1_HUMAN | 0.81461 | -0.059384978 |
| P35606 | COPB2_HUMAN | 0.81476 | 0.027131885 |
| P30086 | PEBP1_HUMAN | 0.81618 | -0.015822736 |
| Q04941 | PLP2_HUMAN | 0.81866 | 0.046951455 |
| Q9UNM6 | PSD13_HUMAN | 0.81923 | 0.059635469 |
| P20036 | DPA1_HUMAN | 0.82083 | -0.030946823 |
| Q07021 | C1QBP_HUMAN | 0.82099 | -0.028888701 |
| P33121 | ACSL1_HUMAN | 0.82107 | -0.035970467 |
| Q14166 | TTL12_HUMAN | 0.82189 | 0.048757582 |
| Q12904 | AIMP1_HUMAN | 0.82225 | -0.038104239 |
| Q9BRX8 | PXL2A_HUMAN | 0.82438 | -0.042061685 |
| P00352 | AL1A1_HUMAN | 0.82497 | -0.047600054 |
| P07910 | HNRPC_HUMAN | 0.82502 | -0.064581608 |
| Q07065 | CKAP4_HUMAN | 0.82503 | -0.03026326 |
| P63172 | DYLT1_HUMAN | 0.8253 | 0.028511392 |
| Q9HD20 | AT131_HUMAN | 0.82618 | -0.031903691 |
| P53597 | SUCA_HUMAN | 0.82634 | 0.037259333 |
| O96005 | CLPT1_HUMAN | 0.8274 | -0.022467808 |
| Q16531 | DDB1_HUMAN | 0.82815 | -0.0321641 |
| Q96C23 | GALM_HUMAN | 0.82875 | -0.063792622 |
| Q00653 | NFKB2_HUMAN | 0.82926 | 0.052911787 |
| P29590 | PML_HUMAN | 0.83047 | 0.021101201 |
| Q15369 | ELOC_HUMAN | 0.83062 | 0.02896653 |
| O94766 | B3GA3_HUMAN | 0.83074 | 0.037107032 |
| Q9H3N1 | TMX1_HUMAN | 0.83164 | -0.010511973 |
| Q07020 | RL18_HUMAN | 0.83255 | -0.025389159 |
| Q86VB7 | C163A_HUMAN | 0.83273 | 0.04896392 |
| P08195 | 4F2_HUMAN | 0.83395 | 0.019509825 |
| P04233-2 | HG2A_HUMAN | 0.83395 | -0.041713209 |
| Q9NSE4 | SYIM_HUMAN | 0.83574 | -0.023253277 |
| Q9NX40 | OCAD1_HUMAN | 0.83619 | -0.064464909 |
| P04899 | GNAI2_HUMAN | 0.83626 | 0.0084534 |
| Q9NZT2 | OGFR_HUMAN | 0.83733 | 0.041136664 |
| O95782 | AP2A1_HUMAN | 0.83755 | -0.033583776 |
| P40939 | ECHA_HUMAN | 0.83774 | -0.029113677 |
| Q9NQC3-2 | RTN4_HUMAN | 0.8399 | -0.031865633 |
| Q96FW1 | OTUB1_HUMAN | 0.84057 | -0.019381226 |
| P50502 | F10A1_HUMAN | 0.84112 | -0.027857864 |
| Q01105 | SET_HUMAN | 0.84145 | -0.018246716 |
| Q32P28 | P3H1_HUMAN | 0.84291 | -0.062504724 |
| Q16555 | DPYL2_HUMAN | 0.84484 | -0.008591407 |
| P28074 | PSB5_HUMAN | 0.84496 | 0.033499303 |
| Q7Z3J2 | VP35L_HUMAN | 0.84507 | -0.036868983 |
| P20936 | RASA1_HUMAN | 0.84604 | 0.05019466 |
| O15347 | HMGB3_HUMAN | 0.84715 | -0.041974883 |
| O60603 | TLR2_HUMAN | 0.84747 | -0.040845058 |
| Q5T447 | HECD3_HUMAN | 0.84777 | -0.028007937 |
| P28067 | DMA_HUMAN | 0.84915 | 0.022672868 |
| P62330 | ARF6_HUMAN | 0.84983 | 0.021104946 |
| Q99439 | CNN2_HUMAN | 0.85064 | 0.019974749 |
| P46777 | RL5_HUMAN | 0.85291 | 0.022240902 |
| P54136 | SYRC_HUMAN | 0.8542 | -0.017519415 |
| P38919 | IF4A3_HUMAN | 0.85647 | -0.021002089 |
| O75688 | PPM1B_HUMAN | 0.85686 | -0.045288984 |
| P62854 | RS26_HUMAN | 0.85695 | -0.034157898 |
| Q86TU7 | SETD3_HUMAN | 0.85763 | 0.02485065 |
| P28838 | AMPL_HUMAN | 0.85854 | -0.019163905 |
| O95573 | ACSL3_HUMAN | 0.85901 | -0.018163563 |
| Q9H3P7 | GCP60_HUMAN | 0.85928 | 0.030785269 |
| Q9NR30 | DDX21_HUMAN | 0.8624 | 0.040759378 |
| Q15365 | PCBP1_HUMAN | 0.86298 | 0.028166247 |
| Q8TCT8 | SPP2A_HUMAN | 0.86437 | -0.029230112 |
| P49327 | FAS_HUMAN | 0.86471 | -0.023488313 |
| P08311 | CATG_HUMAN | 0.86582 | -0.03854543 |
| Q96KP4 | CNDP2_HUMAN | 0.86601 | -0.013001381 |
| P49368 | TCPG_HUMAN | 0.86749 | 0.010633946 |
| Q8NBQ5 | DHB11_HUMAN | 0.86827 | 0.015582062 |
| O94905 | ERLN2_HUMAN | 0.87013 | 0.011430129 |
| P09467 | F16P1_HUMAN | 0.87049 | -0.023261839 |
| Q02543 | RL18A_HUMAN | 0.87642 | -0.021362516 |
| P61626 | LYSC_HUMAN | 0.87737 | 0.030203058 |
| P07203 | GPX1_HUMAN | 0.87772 | 0.015306629 |
| Q9Y6Q1 | CAN6_HUMAN | 0.87867 | -0.034348886 |
| P04066 | FUCO_HUMAN | 0.87927 | -0.011472238 |
| P53618 | COPB_HUMAN | 0.87995 | 0.03486699 |
| P62857 | RS28_HUMAN | 0.88 | -0.015381202 |
| Q13596 | SNX1_HUMAN | 0.8819 | -0.026609482 |
| Q9UH99 | SUN2_HUMAN | 0.88365 | -0.024813585 |
| P56556 | NDUA6_HUMAN | 0.88474 | -0.029297568 |
| Q9H9B4 | SFXN1_HUMAN | 0.88515 | 0.015482874 |
| Q9Y6U3 | ADSV_HUMAN | 0.88516 | 0.03044197 |
| Q13011 | ECH1_HUMAN | 0.8855 | -0.014831665 |
| P61604 | CH10_HUMAN | 0.88679 | -0.017900932 |
| Q9UID3 | VPS51_HUMAN | 0.88721 | -0.032061617 |
| Q86U42 | PABP2_HUMAN | 0.88908 | 0.014279284 |
| Q5TEJ8 | THMS2_HUMAN | 0.8898 | -0.043808034 |
| Q92769 | HDAC2_HUMAN | 0.89181 | 0.021404258 |
| P52597 | HNRPF_HUMAN | 0.89227 | -0.013078339 |
| O95168-2 | NDUB4_HUMAN | 0.89246 | 0.016286471 |
| P10768 | ESTD_HUMAN | 0.89329 | -0.012675562 |
| P08240 | SRPRA_HUMAN | 0.89333 | 0.046664134 |
| Q4V328 | GRAP1_HUMAN | 0.89337 | -0.020071582 |
| Q14376 | GALE_HUMAN | 0.89405 | -0.025958883 |
| Q16891 | MIC60_HUMAN | 0.89406 | 0.049056903 |
| P16615 | AT2A2_HUMAN | 0.89447 | -0.011075211 |
| Q9Y394 | DHRS7_HUMAN | 0.89481 | -0.009866708 |
| Q14344 | GNA13_HUMAN | 0.89578 | 0.021990726 |
| P51692 | STA5B_HUMAN | 0.89728 | -0.02045251 |
| Q16775 | GLO2_HUMAN | 0.89822 | -0.021762943 |
| P26641 | EF1G_HUMAN | 0.89901 | 0.009207095 |
| O14874-2 | BCKD_HUMAN | 0.89978 | 0.026364514 |
| P67936 | TPM4_HUMAN | 0.90054 | 0.008304768 |
| P53992 | SC24C_HUMAN | 0.90096 | 0.033061077 |
| P62942 | FKB1A_HUMAN | 0.9013 | -0.007983193 |
| O43402 | EMC8_HUMAN | 0.90271 | -0.017143947 |
| Q68EM7 | RHG17_HUMAN | 0.90379 | -0.023049279 |
| P11142 | HSP7C_HUMAN | 0.90415 | 0.002340146 |
| P15529 | MCP_HUMAN | 0.90497 | 0.032942273 |
| Q8IUE6 | H2A2B_HUMAN | 0.9077 | -0.015772829 |
| P09972 | ALDOC_HUMAN | 0.9093 | -0.018270929 |
| O75436 | VP26A_HUMAN | 0.90963 | -0.018701609 |
| O75907 | DGAT1_HUMAN | 0.91204 | -0.022862584 |
| Q96QK1 | VPS35_HUMAN | 0.91401 | 0.005657208 |
| Q8WYA6 | CTBL1_HUMAN | 0.91416 | 0.016545629 |
| P27338 | AOFB_HUMAN | 0.91694 | 0.018426067 |
| P11586 | C1TC_HUMAN | 0.91842 | -0.018025544 |
| P16144 | ITB4_HUMAN | 0.91906 | 0.022297187 |
| Q96I99 | SUCB2_HUMAN | 0.92296 | 0.013638009 |
| P08238 | HS90B_HUMAN | 0.92471 | 0.009697046 |
| P61978 | HNRPK_HUMAN | 0.92501 | 0.0118514 |
| P24752 | THIL_HUMAN | 0.92577 | -0.011778394 |
| P00167 | CYB5_HUMAN | 0.92588 | 0.012700004 |
| Q9Y305 | ACOT9_HUMAN | 0.92603 | 0.016371731 |
| Q92598 | HS105_HUMAN | 0.92619 | 0.007947578 |
| O15371 | EIF3D_HUMAN | 0.92685 | 0.018482538 |
| Q71U36 | TBA1A_HUMAN | 0.92724 | -0.02560697 |
| P06748 | NPM_HUMAN | 0.92754 | -0.01433303 |
| Q9NQR4 | NIT2_HUMAN | 0.92765 | -0.015557944 |
| P30536 | TSPO_HUMAN | 0.92776 | 0.017669161 |
| P56377 | AP1S2_HUMAN | 0.92788 | 0.015820305 |
| Q9UBQ7 | GRHPR_HUMAN | 0.92789 | -0.007943108 |
| P30533 | AMRP_HUMAN | 0.93143 | -0.037427001 |
| Q92974 | ARHG2_HUMAN | 0.93284 | 0.008879718 |
| Q08380 | LG3BP_HUMAN | 0.93612 | -0.006192514 |
| Q8TAT6 | NPL4_HUMAN | 0.93716 | -0.013529608 |
| Q7KZF4 | SND1_HUMAN | 0.93724 | 0.007314102 |
| O43747 | AP1G1_HUMAN | 0.93998 | 0.009296126 |
| A0AVT1 | UBA6_HUMAN | 0.94085 | 0.007958771 |
| Q9UBW5 | BIN2_HUMAN | 0.94206 | -0.013354127 |
| P51149 | RAB7A_HUMAN | 0.94219 | 0.002370991 |
| P17661 | DESM_HUMAN | 0.94319 | 0.010678223 |
| P20645 | MPRD_HUMAN | 0.9435 | 0.010331573 |
| Q9Y333 | LSM2_HUMAN | 0.94468 | -0.012306683 |
| O14684 | PTGES_HUMAN | 0.94606 | -0.00708826 |
| P07737 | PROF1_HUMAN | 0.94737 | -0.007626183 |
| P61254 | RL26_HUMAN | 0.94743 | 0.012084119 |
| O75367 | H2AY_HUMAN | 0.94878 | 0.013398519 |
| O75874 | IDHC_HUMAN | 0.95031 | 0.006456235 |
| P55060 | XPO2_HUMAN | 0.95108 | 0.011243388 |
| O60610 | DIAP1_HUMAN | 0.95294 | -0.005512767 |
| Q99805 | TM9S2_HUMAN | 0.95335 | 0.005936598 |
| Q9Y262 | EIF3L_HUMAN | 0.95373 | 0.004690241 |
| P46781 | RS9_HUMAN | 0.95396 | 0.009098701 |
| Q9UBS4 | DJB11_HUMAN | 0.95412 | 0.012397346 |
| Q1KMD3 | HNRL2_HUMAN | 0.95829 | -0.007389643 |
| P48556 | PSMD8_HUMAN | 0.96113 | 0.007707214 |
| O75369-8 | FLNB_HUMAN | 0.96114 | 0.00826147 |
| P26639 | SYTC_HUMAN | 0.96134 | 0.003930085 |
| P23381 | SYWC_HUMAN | 0.96278 | -0.005859482 |
| Q13148 | TADBP_HUMAN | 0.96324 | 0.013604358 |
| P28482 | MK01_HUMAN | 0.9653 | 0.004453657 |
| P31146 | COR1A_HUMAN | 0.96534 | 0.0078277 |
| P08473 | NEP_HUMAN | 0.96559 | -0.009017903 |
| Q7L2H7 | EIF3M_HUMAN | 0.96592 | -0.007982262 |
| Q9P2R7 | SUCB1_HUMAN | 0.96643 | -0.004399159 |
| Q15057 | ACAP2_HUMAN | 0.96684 | 0.005331772 |
| Q9P0S3 | ORML1_HUMAN | 0.96735 | 0.00541019 |
| P49189-3 | AL9A1_HUMAN | 0.96778 | 0.009755399 |
| O43914 | TYOBP_HUMAN | 0.96806 | -0.006728705 |
| Q8IVH4 | MMAA_HUMAN | 0.9686 | -0.009472012 |
| Q99536 | VAT1_HUMAN | 0.97017 | -0.004390917 |
| P62888 | RL30_HUMAN | 0.97034 | 0.002098095 |
| Q9Y6W5 | WASF2_HUMAN | 0.97094 | 0.002276417 |
| Q96G03 | PGM2_HUMAN | 0.97188 | -0.00297662 |
| Q9BXJ9 | NAA15_HUMAN | 0.97323 | 0.007185711 |
| Q92542 | NICA_HUMAN | 0.97325 | -0.002373771 |
| P46926 | GNPI1_HUMAN | 0.97542 | -0.003286432 |
| O43765 | SGTA_HUMAN | 0.97545 | 0.00402463 |
| O14683 | P5I11_HUMAN | 0.97717 | 0.003616191 |
| Q9Y315 | DEOC_HUMAN | 0.97731 | -0.001715946 |
| P61106 | RAB14_HUMAN | 0.97818 | -0.005071841 |
| P40121 | CAPG_HUMAN | 0.97907 | 0.002961017 |
| P13667 | PDIA4_HUMAN | 0.97987 | -0.001590252 |
| P07195 | LDHB_HUMAN | 0.98376 | 0.000768523 |
| Q5EBM0 | CMPK2_HUMAN | 0.98379 | 0.003030894 |
| Q99613 | EIF3C_HUMAN | 0.98445 | -0.001643489 |
| Q9HAV0 | GBB4_HUMAN | 0.98452 | -0.002537231 |
| P61009 | SPCS3_HUMAN | 0.98562 | -0.00188437 |
| P50914 | RL14_HUMAN | 0.98632 | 0.001612958 |
| P14598 | NCF1_HUMAN | 0.9864 | -0.002283237 |
| Q15437 | SC23B_HUMAN | 0.98733 | -0.001728446 |
| P62829 | RL23_HUMAN | 0.98827 | 0.002728423 |
| Q12931 | TRAP1_HUMAN | 0.98861 | -0.002248444 |
| P14902 | I23O1_HUMAN | 0.98871 | 0.003399478 |
| P05141 | ADT2_HUMAN | 0.98897 | -0.000778065 |
| O00186 | STXB3_HUMAN | 0.99077 | -0.0013142 |
| Q8N6T3 | ARFG1_HUMAN | 0.99122 | -0.001875975 |
| Q6P996 | PDXD1_HUMAN | 0.99163 | -0.001897423 |
| Q92841 | DDX17_HUMAN | 0.99189 | -0.001235408 |
| O43390 | HNRPR_HUMAN | 0.99319 | -0.000929863 |
| Q92643 | GPI8_HUMAN | 0.99341 | -0.001409912 |
| P62244 | RS15A_HUMAN | 0.9935 | 0.000808523 |
| Q9BT78 | CSN4_HUMAN | 0.99378 | 0.000956808 |
| P31937 | 3HIDH_HUMAN | 0.99389 | -0.001033629 |
| P20701 | ITAL_HUMAN | 0.99444 | -0.000844368 |
| Q16666-3 | IF16_HUMAN | 0.99461 | -0.000708127 |
| P02545-2 | LMNA_HUMAN | 0.99613 | 0.000766192 |
| Q9NYL9 | TMOD3_HUMAN | 0.99629 | -0.000831137 |
| Q14103-4 | HNRPD_HUMAN | 0.99696 | -0.00022271 |
| P43307 | SSRA_HUMAN | 0.99699 | 0.000444373 |
| P02746 | C1QB_HUMAN | 0.99701 | 0.00060233 |
| Q13616 | CUL1_HUMAN | 0.99742 | -0.001025885 |
| P04083 | ANXA1_HUMAN | 0.99758 | -0.000443612 |
| P17900 | SAP3_HUMAN | 0.9977 | -0.000581155 |
| Q96GA7 | SDSL_HUMAN | 0.99793 | 0.000196506 |
| P56385 | ATP5I_HUMAN | 0.99909 | -0.000272395 |
| P07711 | CATL1_HUMAN | 0.99976 | 6.59E-05 |
