# Supplementary material for: Extracellular vesicle-associated miR-515-5p from adipose tissue regulates placental metabolism and fetal growth in gestational diabetes mellitus
Source: Cardiovasc Diabetol. 2025 May 14;24:205. doi: 10.1186/s12933-025-02739-z (PMC12080180; doi:10.1186/s12933-025-02739-z)
Supplement: Supplementary file 9 — Supplementary Material 9 [file 12933_2025_2739_MOESM9_ESM.docx]

**Supplementary Table 8: Table below shows the pathways regulated by miR-146a-5p in the placental cells identified using Gene Ontology analysis**

| **Protein ID** | **Mapped ID** | **Group** | **p-value** | **Log (FC)** |
| --- | --- | --- | --- | --- |
| P05161 | ISG15_HUMAN | Ubiquitin-like protein ISG15 | 0.00053 | -0.365312191 |
| Q9BYX4 | IFIH1_HUMAN | Interferon-induced helicase C domain-containing protein 1 | 0.00069 | -0.380175231 |
| P09914 | IFIT1_HUMAN | Interferon-induced protein with tetratricopeptide repeats 1 | 0.00072 | -0.354061517 |
| P20591 | MX1_HUMAN | Interferon-induced GTP-binding protein Mx1 | 0.00213 | -0.348783259 |
| O95786 | DDX58_HUMAN | Probable ATP-dependent RNA helicase DDX58 | 0.00231 | -0.507387672 |
| P08865 | RSSA_HUMAN | 40S ribosomal protein SA | 0.00283 | 0.0647738 |
| Q16531 | DDB1_HUMAN | DNA damage-binding protein 1 | 0.00422 | 0.199570174 |
| P30050 | RL12_HUMAN | 60S ribosomal protein L12 | 0.0076 | 0.049619761 |
| P32969 | RL9_HUMAN | 60S ribosomal protein L9 | 0.00797 | 0.135633836 |
| O75400 | PR40A_HUMAN | Pre-mRNA-processing factor 40 homolog A | 0.00881 | 0.038061007 |
| Q9UBW5 | BIN2_HUMAN | Bridging integrator 2 | 0.00932 | -0.505335983 |
| O14879 | IFIT3_HUMAN | Interferon-induced protein with tetratricopeptide repeats 3 | 0.00985 | -0.392159493 |
| P09913 | IFIT2_HUMAN | Interferon-induced protein with tetratricopeptide repeats 2 | 0.01296 | -0.34081484 |
| O15162 | PLS1_HUMAN | Phospholipid scramblase 1 | 0.0157 | -0.205274617 |
| O75396 | SC22B_HUMAN | Vesicle-trafficking protein SEC22b | 0.01789 | 0.077757875 |
| P20674 | COX5A_HUMAN | Cytochrome c oxidase subunit 5A, mitochondrial | 0.01817 | -0.226762294 |
| P07919 | QCR6_HUMAN | Cytochrome b-c1 complex subunit 6, mitochondrial | 0.01968 | -0.156398075 |
| Q12931 | TRAP1_HUMAN | Heat shock protein 75 kDa, mitochondrial | 0.02162 | 0.053774485 |
| P27694 | RFA1_HUMAN | Replication protein A 70 kDa DNA-binding subunit | 0.02331 | 0.217670663 |
| P62314 | SMD1_HUMAN | Small nuclear ribonucleoprotein Sm D1 | 0.02516 | 0.047125254 |
| Q14258 | TRI25_HUMAN | E3 ubiquitin/ISG15 ligase TRIM25 | 0.02595 | -0.117723276 |
| P61247 | RS3A_HUMAN | 40S ribosomal protein S3a | 0.02769 | 0.061287229 |
| P26373 | RL13_HUMAN | 60S ribosomal protein L13 | 0.02827 | 0.057394773 |
| P27695 | APEX1_HUMAN | DNA-(apurinic or apyrimidinic site) lyase | 0.02853 | 0.115248614 |
| Q14669 | TRIPC_HUMAN | E3 ubiquitin-protein ligase TRIP12 | 0.03142 | 0.120462685 |
| Q9UNF0 | PACN2_HUMAN | Protein kinase C and casein kinase substrate in neurons protein 2 | 0.03163 | 0.238009556 |
| P50914 | RL14_HUMAN | 60S ribosomal protein L14 | 0.03339 | 0.053110056 |
| P19525 | E2AK2_HUMAN | Interferon-induced, double-stranded RNA-activated protein kinase | 0.03556 | -0.14241872 |
| P05388 | RLA0_HUMAN | 60S acidic ribosomal protein P0 | 0.03872 | 0.069337305 |
| P52907 | CAZA1_HUMAN | F-actin-capping protein subunit alpha-1 | 0.03904 | -0.17238444 |
| Q13418 | ILK_HUMAN | Integrin-linked protein kinase | 0.04062 | 0.071511789 |
| P06744-2 | G6PI_HUMAN | Isoform 2 of Glucose-6-phosphate isomerase | 0.04186 | 0.18607502 |
| O15355 | PPM1G_HUMAN | Protein phosphatase 1G | 0.04186 | 0.256943021 |
| Q9Y4L1 | HYOU1_HUMAN | Hypoxia up-regulated protein 1 | 0.04459 | 0.037497486 |
| P27635 | RL10_HUMAN | 60S ribosomal protein L10 | 0.04558 | 0.269407107 |
| Q02543 | RL18A_HUMAN | 60S ribosomal protein L18a | 0.04767 | 0.069980189 |
| P29728 | OAS2_HUMAN | 2'-5'-oligoadenylate synthase 2 | 0.05202 | -0.242746849 |
| O75390 | CISY_HUMAN | Citrate synthase, mitochondrial | 0.05459 | 0.124879141 |
| Q9Y383 | LC7L2_HUMAN | Putative RNA-binding protein Luc7-like 2 | 0.05587 | 0.177785721 |
| P18621-3 | RL17_HUMAN | Isoform 3 of 60S ribosomal protein L17 | 0.05626 | 0.056731575 |
| P54577 | SYYC_HUMAN | Tyrosine--tRNA ligase, cytoplasmic | 0.05682 | -0.115405542 |
| P23396 | RS3_HUMAN | 40S ribosomal protein S3 | 0.05703 | 0.06634655 |
| Q9Y6E0 | STK24_HUMAN | Serine/threonine-protein kinase 24 | 0.05804 | 0.139302563 |
| P39023 | RL3_HUMAN | 60S ribosomal protein L3 | 0.0596 | 0.056044679 |
| Q15717-2 | ELAV1_HUMAN | Isoform 2 of ELAV-like protein 1 | 0.05995 | 0.14499195 |
| P25786 | PSA1_HUMAN | Proteasome subunit alpha type-1 | 0.06021 | -0.02525356 |
| P02774 | VTDB_HUMAN | Vitamin D-binding protein | 0.06324 | -0.167546253 |
| Q13287 | NMI_HUMAN | N-myc-interactor | 0.0651 | -0.20101684 |
| O94919 | ENDD1_HUMAN | Endonuclease domain-containing 1 protein | 0.06538 | -0.407696089 |
| Q09666 | AHNK_HUMAN | Neuroblast differentiation-associated protein AHNAK | 0.06632 | 0.167373296 |
| P13489 | RINI_HUMAN | Ribonuclease inhibitor | 0.06678 | 0.129298268 |
| P55265 | DSRAD_HUMAN | Double-stranded RNA-specific adenosine deaminase | 0.06908 | -0.169695921 |
| Q5JTV8 | TOIP1_HUMAN | Torsin-1A-interacting protein 1 | 0.06918 | -0.168528201 |
| P51970 | NDUA8_HUMAN | NADH dehydrogenase [ubiquinone] 1 alpha subcomplex subunit 8 | 0.07119 | 0.553329972 |
| O00764 | PDXK_HUMAN | Pyridoxal kinase | 0.07154 | 0.248678378 |
| P35268 | RL22_HUMAN | 60S ribosomal protein L22 | 0.07553 | 0.052933966 |
| Q16891 | MIC60_HUMAN | MIC | 0.07665 | 0.150284618 |
| O60884 | DNJA2_HUMAN | DnaJ homolog subfamily A member 2 | 0.0785 | 0.064201837 |
| Q9Y265 | RUVB1_HUMAN | RuvB-like 1 | 0.08042 | -0.19865306 |
| Q14152 | EIF3A_HUMAN | Eukaryotic translation initiation factor 3 subunit A | 0.0811 | 0.044423908 |
| Q96FW1 | OTUB1_HUMAN | Ubiquitin thioesterase | 0.08161 | 0.155593424 |
| P14868 | SYDC_HUMAN | Aspartate--tRNA ligase, cytoplasmic | 0.08188 | 0.05640109 |
| P02768 | ALBU_HUMAN | Serum albumin | 0.08443 | -0.1352501 |
| P62851 | RS25_HUMAN | 40S ribosomal protein S25 | 0.08524 | 0.101761265 |
| P02788 | TRFL_HUMAN | Lactotransferrin | 0.08662 | -0.193614751 |
| Q27J81 | INF2_HUMAN | Inverted formin-2 | 0.0873 | -0.484200941 |
| Q9NYU2 | UGGG1_HUMAN | UDP-glucose:glycoprotein glucosyltransferase 1 | 0.08736 | -0.129853023 |
| Q07020 | RL18_HUMAN | 60S ribosomal protein L18 | 0.08832 | 0.059198074 |
| P42677 | RS27_HUMAN | 40S ribosomal protein S27 | 0.08894 | 0.149065187 |
| O75955 | FLOT1_HUMAN | Flotillin-1 | 0.08965 | 0.182853353 |
| Q9NVA2 | SEP11_HUMAN | Septin-11 | 0.09034 | -0.332705791 |
| P49411 | EFTU_HUMAN | Elongation factor Tu, mitochondrial | 0.09172 | 0.039791132 |
| A0AV96 | RBM47_HUMAN | RNA-binding protein 47 | 0.09203 | 0.063505901 |
| P62263 | RS14_HUMAN | 40S ribosomal protein S14 | 0.09232 | 0.12869231 |
| O43865 | SAHH2_HUMAN | S-adenosylhomocysteine hydrolase-like protein 1 | 0.09296 | 0.337648487 |
| Q9UDY2 | ZO2_HUMAN | Tight junction protein ZO-2 | 0.09503 | 0.211568537 |
| P61966 | AP1S1_HUMAN | AP-1 complex subunit sigma-1A | 0.09515 | 0.170283064 |
| Q86Y82 | STX12_HUMAN | Syntaxin-12 | 0.09525 | -0.101501531 |
| P17813 | EGLN_HUMAN | Endoglin | 0.0953 | -0.189102641 |
| P60900 | PSA6_HUMAN | Proteasome subunit alpha type-6 | 0.09623 | -0.034465559 |
| O00299 | CLIC1_HUMAN | Chloride intracellular channel protein 1 | 0.09903 | -0.098627059 |
| P27348 | 1433T_HUMAN | 14-3-3 protein theta | 0.09945 | -0.324265462 |
| Q00169 | PIPNA_HUMAN | Phosphatidylinositol transfer protein alpha isoform | 0.10199 | -0.149096723 |
| P11234 | RALB_HUMAN | Ras-related protein Ral-B | 0.10242 | 0.210289506 |
| Q96M27 | PRRC1_HUMAN | Protein PRRC1 | 0.10258 | 0.135375871 |
| P26196 | DDX6_HUMAN | Probable ATP-dependent RNA helicase DDX6 | 0.10301 | 0.031705829 |
| Q9NX14 | NDUBB_HUMAN | NADH dehydrogenase [ubiquinone] 1 beta subcomplex subunit 11, mitochondrial | 0.10366 | -0.055484535 |
| P83731 | RL24_HUMAN | 60S ribosomal protein L24 | 0.10414 | 0.121834588 |
| O60506 | HNRPQ_HUMAN | Heterogeneous nuclear ribonucleoprotein Q | 0.10735 | 0.043990004 |
| P08174-7 | DAF_HUMAN | Isoform 7 of Complement decay-accelerating factor | 0.10941 | -0.160430271 |
| P60468 | SC61B_HUMAN | Protein transport protein Sec61 subunit beta | 0.11138 | 0.08073021 |
| P07355-2 | ANXA2_HUMAN | Isoform 2 of Annexin A2 | 0.1125 | 0.130352687 |
| P62829 | RL23_HUMAN | 60S ribosomal protein L23 | 0.1147 | 0.053653092 |
| Q15907 | RB11B_HUMAN | Ras-related protein Rab-11B | 0.11622 | 0.035205986 |
| Q9GZT6 | CC90B_HUMAN | Coiled-coil domain-containing protein 90B, mitochondrial | 0.11629 | -0.034208672 |
| O75351 | VPS4B_HUMAN | Vacuolar protein sorting-associated protein 4B | 0.11734 | -0.151477956 |
| P62249 | RS16_HUMAN | 40S ribosomal protein S16 | 0.11768 | 0.058334532 |
| Q16706 | MA2A1_HUMAN | Alpha-mannosidase 2 | 0.11879 | 0.150029732 |
| P04066 | FUCO_HUMAN | Tissue alpha-L-fucosidase | 0.11976 | -0.247642907 |
| P09543 | CN37_HUMAN | 2',3'-cyclic-nucleotide 3'-phosphodiesterase | 0.11995 | -0.074481074 |
| P46777 | RL5_HUMAN | 60S ribosomal protein L5 | 0.12293 | 0.056106323 |
| P00441 | SODC_HUMAN | Superoxide dismutase [Cu-Zn] | 0.12301 | -0.240277989 |
| Q00013 | EM55_HUMAN | 55 kDa erythrocyte membrane protein | 0.12378 | 0.175922521 |
| Q9UKV3-5 | ACINU_HUMAN | Isoform 4 of Apoptotic chromatin condensation inducer in the nucleus | 0.12481 | 0.091329156 |
| P08708 | RS17_HUMAN | 40S ribosomal protein S17 | 0.12575 | 0.097753093 |
| O75083 | WDR1_HUMAN | WD repeat-containing protein 1 | 0.12626 | 0.045449009 |
| P62280 | RS11_HUMAN | 40S ribosomal protein S11 | 0.12692 | 0.176123286 |
| Q7L576 | CYFP1_HUMAN | Cytoplasmic FMR1-interacting protein 1 | 0.12877 | 0.058232528 |
| Q9NVJ2 | ARL8B_HUMAN | ADP-ribosylation factor-like protein 8B | 0.13188 | -0.20644172 |
| O75694 | NU155_HUMAN | Nuclear pore complex protein Nup155 | 0.13198 | 0.145746335 |
| P02765 | FETUA_HUMAN | Alpha-2-HS-glycoprotein | 0.13276 | -0.097116486 |
| P11498 | PYC_HUMAN | Pyruvate carboxylase, mitochondrial | 0.13306 | 0.098715992 |
| P18754 | RCC1_HUMAN | Regulator of chromosome condensation | 0.13354 | 0.058378093 |
| P62269 | RS18_HUMAN | 40S ribosomal protein S18 | 0.13387 | 0.060035814 |
| P35606 | COPB2_HUMAN | Coatomer subunit beta' | 0.13401 | 0.077359667 |
| Q92572 | AP3S1_HUMAN | AP-3 complex subunit sigma-1 | 0.13509 | 0.175834961 |
| Q13636 | RAB31_HUMAN | Ras-related protein Rab-31 | 0.13521 | 0.063340209 |
| Q02978 | M2OM_HUMAN | Mitochondrial 2-oxoglutarate/malate carrier protein | 0.13571 | 0.422377979 |
| P46781 | RS9_HUMAN | 40S ribosomal protein S9 | 0.13742 | 0.069204986 |
| Q99961 | SH3G1_HUMAN | Endophilin-A2 | 0.13812 | 0.076551327 |
| Q96CS3 | FAF2_HUMAN | FAS-associated factor 2 | 0.13831 | 0.042043698 |
| P46778 | RL21_HUMAN | 60S ribosomal protein L21 | 0.13896 | 0.05348558 |
| Q99798 | ACON_HUMAN | Aconitate hydratase, mitochondrial | 0.13986 | -0.066883363 |
| P63167 | DYL1_HUMAN | Dynein light chain 1, cytoplasmic | 0.1399 | 0.031513097 |
| Q8TBC4 | UBA3_HUMAN | NEDD8-activating enzyme E1 catalytic subunit | 0.14027 | -0.055410061 |
| P62753 | RS6_HUMAN | 40S ribosomal protein S6 | 0.14101 | 0.176546255 |
| Q16181 | SEPT7_HUMAN | Septin-7 | 0.14198 | 0.077519903 |
| Q08380 | LG3BP_HUMAN | Galectin-3-binding protein | 0.14206 | -0.08718746 |
| Q9BTV4 | TMM43_HUMAN | Transmembrane protein 43 | 0.14298 | -0.183621546 |
| P13798 | ACPH_HUMAN | Acylamino-acid-releasing enzyme | 0.14574 | 0.10861724 |
| Q13564 | ULA1_HUMAN | NEDD8-activating enzyme E1 regulatory subunit | 0.14584 | -0.353028137 |
| P55084 | ECHB_HUMAN | Trifunctional enzyme subunit beta, mitochondrial | 0.14955 | 0.089925019 |
| P60842 | IF4A1_HUMAN | Eukaryotic initiation factor 4A-I | 0.15162 | 0.065716104 |
| Q14134 | TRI29_HUMAN | Tripartite motif-containing protein 29 | 0.1567 | 0.426853305 |
| Q9UBE0 | SAE1_HUMAN | SUMO- activating enzyme subunit 1 | 0.15703 | 0.10842967 |
| P28066 | PSA5_HUMAN | Proteasome subunit alpha type-5 | 0.15722 | -0.058799707 |
| Q00059 | TFAM_HUMAN | Transcription factor A, mitochondrial | 0.15898 | -0.13305493 |
| P09429 | HMGB1_HUMAN | High mobility group protein B1 | 0.15991 | 0.077985102 |
| P98082 | DAB2_HUMAN | Disabled homolog 2 | 0.16084 | 0.095868347 |
| Q9NR31 | SAR1A_HUMAN | GTP-binding protein SAR1a | 0.1613 | 0.117102167 |
| P61254 | RL26_HUMAN | 60S ribosomal protein L26 | 0.16166 | 0.081812088 |
| P54886 | P5CS_HUMAN | Delta-1-pyrroline-5-carboxylate synthase | 0.1618 | 0.223372298 |
| P26640 | SYVC_HUMAN | Valine--tRNA ligase | 0.16229 | 0.126617487 |
| P51149 | RAB7A_HUMAN | Ras-related protein Rab-7a | 0.16299 | 0.070472271 |
| P51571 | SSRD_HUMAN | Translocon-associated protein subunit delta | 0.16621 | -0.032928192 |
| P00747 | PLMN_HUMAN | Plasminogen | 0.16707 | -0.134731976 |
| P62879 | GBB2_HUMAN | Guanine nucleotide-binding protein G(I)/G(S)/G(T) subunit beta-2 | 0.16743 | 0.225343012 |
| Q99714 | HCD2_HUMAN | 3-hydroxyacyl-CoA dehydrogenase type-2 | 0.16769 | 0.163326959 |
| P42766 | RL35_HUMAN | 60S ribosomal protein L35 | 0.16877 | 0.053350462 |
| P61289 | PSME3_HUMAN | Proteasome activator complex subunit 3 | 0.16953 | -0.068369421 |
| P43034 | LIS1_HUMAN | Platelet-activating factor acetylhydrolase IB subunit alpha | 0.1701 | 0.211903192 |
| P84098 | RL19_HUMAN | 60S ribosomal protein L19 | 0.17311 | 0.048963972 |
| Q86XR7-2 | TCAM2_HUMAN | Isoform 2 of TIR domain-containing adapter molecule 2 | 0.17356 | -0.0638041 |
| Q13423 | NNTM_HUMAN | NAD(P) transhydrogenase, mitochondrial | 0.17369 | 0.10181797 |
| P67775 | PP2AA_HUMAN | Serine/threonine-protein phosphatase 2A catalytic subunit alpha isoform | 0.17692 | -0.055683178 |
| P46940 | IQGA1_HUMAN | Ras GTPase-activating-like protein IQGAP1 | 0.17747 | 0.063856925 |
| O00571 | DDX3X_HUMAN | ATP-dependent RNA helicase DDX3X | 0.17917 | 0.050651572 |
| P35659 | DEK_HUMAN | Protein DEK | 0.18521 | 0.046522789 |
| P40429 | RL13A_HUMAN | 60S ribosomal protein L13a | 0.18551 | 0.058932231 |
| Q04837 | SSBP_HUMAN | Single-stranded DNA-binding protein, mitochondrial | 0.18681 | -0.160885606 |
| Q96NB2 | SFXN2_HUMAN | Sideroflexin-2 | 0.18709 | -0.103861708 |
| O15371 | EIF3D_HUMAN | Eukaryotic translation initiation factor 3 subunit D | 0.18737 | -0.144799555 |
| Q14257 | RCN2_HUMAN | Reticulocalbin-2 | 0.18805 | 0.117927573 |
| Q9H4A4 | AMPB_HUMAN | Aminopeptidase B | 0.18847 | 0.104378976 |
| P07814 | SYEP_HUMAN | Bifunctional glutamate/proline--tRNA ligase | 0.18872 | -0.048481112 |
| Q9Y2A7 | NCKP1_HUMAN | Nck-associated protein 1 | 0.18887 | 0.25262329 |
| Q07960 | RHG01_HUMAN | Rho GTPase-activating protein 1 | 0.18924 | -0.053576676 |
| P30153 | 2AAA_HUMAN | Serine/threonine-protein phosphatase 2A 65 kDa regulatory subunit A alpha isoform | 0.19475 | 0.029023616 |
| P00387-3 | NB5R3_HUMAN | Isoform 3 of NADH-cytochrome b5 reductase 3 | 0.19518 | 0.061273876 |
| P11413-2 | G6PD_HUMAN | Isoform Long of Glucose-6-phosphate 1-dehydrogenase | 0.1972 | 0.131903859 |
| P27338 | AOFB_HUMAN | Amine oxidase [flavin-containing] B | 0.19765 | 0.509756726 |
| P60228 | EIF3E_HUMAN | Eukaryotic translation initiation factor 3 subunit E | 0.19823 | 0.126145317 |
| P49368 | TCPG_HUMAN | T-complex protein 1 subunit gamma | 0.19899 | 0.024278123 |
| P61086 | UBE2K_HUMAN | Ubiquitin-conjugating enzyme E2 K | 0.20156 | 0.152894941 |
| Q07021 | C1QBP_HUMAN | Complement component 1 Q subcomponent-binding protein, mitochondrial | 0.20295 | 0.148088823 |
| Q9NTK5 | OLA1_HUMAN | Obg like ATPase 1 | 0.20386 | -0.047188509 |
| Q15363 | TMED2_HUMAN | Transmembrane emp24 domain-containing protein 2 | 0.20393 | -0.044599841 |
| Q9NZQ7 | PD1L1_HUMAN | Programmed cell death 1 ligand 1 | 0.2051 | -0.147908829 |
| P26599 | PTBP1_HUMAN | Polypyrimidine tract-binding protein 1 | 0.20679 | 0.059650038 |
| P26572 | MGAT1_HUMAN | Alpha-1,3-mannosyl-glycoprotein 2-beta-N-acetylglucosaminyltransferase | 0.20743 | -0.050009634 |
| P17858 | PFKAL_HUMAN | ATP-dependent 6-phosphofructokinase, liver type | 0.20767 | 0.111972902 |
| Q16543 | CDC37_HUMAN | Hsp90 co-chaperone Cdc37 | 0.20778 | 0.082815607 |
| Q16666 | IF16_HUMAN | Gamma-interferon-inducible protein 16 | 0.20792 | -0.103954192 |
| Q04695 | K1C17_HUMAN | Keratin, type I cytoskeletal 17 | 0.2083 | 0.15472497 |
| Q02818 | NUCB1_HUMAN | Nucleobindin-1 | 0.20943 | -0.105785289 |
| P08648 | ITA5_HUMAN | Integrin alpha-5 | 0.20977 | -0.052187028 |
| P35754 | GLRX1_HUMAN | Glutaredoxin-1 | 0.21115 | -0.237405216 |
| P62330 | ARF6_HUMAN | ADP-ribosylation factor 6 | 0.2116 | 0.077393683 |
| P18124 | RL7_HUMAN | 60S ribosomal protein L7 | 0.21302 | 0.091283648 |
| Q70UQ0 | IKIP_HUMAN | Inhibitor of nuclear factor kappa-B kinase-interacting protein | 0.21394 | -0.110223804 |
| Q06830 | PRDX1_HUMAN | Peroxiredoxin-1 | 0.21408 | 0.03529655 |
| O00303 | EIF3F_HUMAN | Eukaryotic translation initiation factor 3 subunit F | 0.2152 | 0.027977614 |
| Q9UHX1 | PUF60_HUMAN | Poly(U)-binding-splicing factor PUF60 | 0.21534 | 0.208705317 |
| P25705 | ATPA_HUMAN | ATP synthase subunit alpha, mitochondrial | 0.21607 | 0.033318447 |
| Q9Y6A9 | SPCS1_HUMAN | Signal peptidase complex subunit 1 | 0.21725 | -0.076125526 |
| P50453 | SPB9_HUMAN | Serpin B9 | 0.21894 | -0.108835904 |
| Q96A33 | CCD47_HUMAN | Coiled-coil domain-containing protein 47 | 0.21906 | 0.050252399 |
| P62917 | RL8_HUMAN | 60S ribosomal protein L8 | 0.21944 | 0.033748747 |
| P02760 | AMBP_HUMAN | Protein AMBP | 0.22059 | -0.117158863 |
| P09758 | TACD2_HUMAN | Tumor-associated calcium signal transducer 2 | 0.22175 | -0.062801096 |
| P53621 | COPA_HUMAN | Coatomer subunit alpha | 0.22374 | 0.068103935 |
| Q96FJ2 | DYL2_HUMAN | Dynein light chain 2, cytoplasmic | 0.22652 | -0.212056039 |
| Q15257 | PTPA_HUMAN | Serine/threonine-protein phosphatase 2A activator | 0.22669 | -0.214625966 |
| Q9NZJ7 | MTCH1_HUMAN | Mitochondrial carrier homolog 1 | 0.22794 | 0.047238025 |
| Q08211 | DHX9_HUMAN | ATP-dependent RNA helicase A | 0.22886 | 0.099591647 |
| Q16795 | NDUA9_HUMAN | NADH dehydrogenase [ubiquinone] 1 alpha subcomplex subunit 9, mitochondrial | 0.22909 | 0.134248708 |
| P47985 | UCRI_HUMAN | Cytochrome b-c1 complex subunit Rieske, mitochondrial | 0.23006 | -0.185219817 |
| P61421 | VA0D1_HUMAN | V-type proton ATPase subunit d 1 | 0.23103 | 0.09235014 |
| Q13155 | AIMP2_HUMAN | Aminoacyl tRNA synthase complex-interacting multifunctional protein 2 | 0.23153 | 0.03515177 |
| P04264 | K2C1_HUMAN | Keratin, type II cytoskeletal 1 | 0.23181 | 0.201523069 |
| O95571 | ETHE1_HUMAN | Persulfide dioxygenase ETHE1, mitochondrial | 0.23303 | 0.101407592 |
| Q15185 | TEBP_HUMAN | Prostaglandin E synthase 3 | 0.23359 | -0.055553416 |
| P11233 | RALA_HUMAN | Ras-related protein Ral-A | 0.23402 | 0.106481076 |
| Q12905 | ILF2_HUMAN | Interleukin enhancer-binding factor 2 | 0.23514 | 0.093795512 |
| Q15833 | STXB2_HUMAN | Syntaxin-binding protein 2 | 0.23542 | 0.06858984 |
| P55884-2 | EIF3B_HUMAN | Isoform 2 of Eukaryotic translation initiation factor 3 subunit B | 0.24005 | -0.017703421 |
| Q14728 | MFS10_HUMAN | Major facilitator superfamily domain-containing protein 10 | 0.24011 | -0.101336596 |
| P35908 | K22E_HUMAN | Keratin, type II cytoskeletal 2 epidermal | 0.24051 | 0.094297948 |
| Q9UQ35 | SRRM2_HUMAN | Serine/arginine repetitive matrix protein 2 | 0.2419 | 0.15368636 |
| P47756-2 | CAPZB_HUMAN | Isoform 2 of F-actin-capping protein subunit beta | 0.24238 | 0.108153679 |
| O43852-3 | CALU_HUMAN | Isoform 3 of Calumenin | 0.24661 | 0.106320978 |
| P62906 | RL10A_HUMAN | 60S ribosomal protein L10a | 0.24803 | 0.050939318 |
| P62910 | RL32_HUMAN | 60S ribosomal protein L32 | 0.24892 | 0.13925546 |
| P07339 | CATD_HUMAN | Cathepsin D | 0.24993 | 0.110871627 |
| Q9BXJ9 | NAA15_HUMAN | N-alpha-acetyltransferase 15, NatA auxiliary subunit | 0.25114 | 0.11985414 |
| Q14204 | DYHC1_HUMAN | Cytoplasmic dynein 1 heavy chain 1 | 0.25206 | 0.055316356 |
| Q9NQG5 | RPR1B_HUMAN | Regulation of nuclear pre-mRNA domain-containing protein 1B | 0.25329 | -0.184835341 |
| Q96CV9-2 | OPTN_HUMAN | Isoform 2 of Optineurin | 0.25447 | -0.079182893 |
| P35613-2 | BASI_HUMAN | Isoform 2 of Basigin | 0.25575 | -0.019994829 |
| P01024 | CO3_HUMAN | Complement C3 | 0.25678 | -0.107985391 |
| P13639 | EF2_HUMAN | Elongation factor 2 | 0.25727 | 0.033537478 |
| P62310 | LSM3_HUMAN | U6 snRNA-associated Sm-like protein LSm3 | 0.25951 | -0.234215187 |
| P35232 | PHB_HUMAN | Prohibitin | 0.26034 | -0.041004522 |
| Q9Y2B0 | CNPY2_HUMAN | Protein canopy homolog 2 | 0.26044 | -0.098485743 |
| P06737 | PYGL_HUMAN | Glycogen phosphorylase, liver form | 0.26066 | 0.126301799 |
| Q53EL6 | PDCD4_HUMAN | Programmed cell death protein 4 | 0.26159 | 0.083241999 |
| Q14980-2 | NUMA1_HUMAN | Isoform 2 of Nuclear mitotic apparatus protein 1 | 0.26288 | -0.226363412 |
| P07996 | TSP1_HUMAN | Thrombospondin-1 | 0.26362 | -0.121721965 |
| Q7KZF4 | SND1_HUMAN | Staphylococcal nuclease domain-containing protein 1 | 0.26363 | 0.039180299 |
| Q15149-4 | PLEC_HUMAN | Isoform 4 of Plectin | 0.26466 | 0.179066167 |
| Q92575 | UBXN4_HUMAN | UBX domain-containing protein 4 | 0.26468 | -0.154382571 |
| P50402 | EMD_HUMAN | Emerin | 0.26478 | 0.115561742 |
| Q8N5K1 | CISD2_HUMAN | CDGSH iron-sulfur domain-containing protein 2 | 0.26593 | -0.082520122 |
| Q9GZU2 | PEG3_HUMAN | Paternally-expressed gene 3 protein | 0.26929 | -0.222782142 |
| Q13283 | G3BP1_HUMAN | Ras GTPase-activating protein-binding protein 1 | 0.26952 | 0.144682202 |
| P20936 | RASA1_HUMAN | Ras GTPase-activating protein 1 | 0.26957 | 0.092854837 |
| Q9HCC0-2 | MCCB_HUMAN | Isoform 2 of Methylcrotonoyl-CoA carboxylase beta chain, mitochondrial | 0.2703 | 0.112175228 |
| P42574 | CASP3_HUMAN | Caspase-3 | 0.27227 | -0.10335585 |
| P02649 | APOE_HUMAN | Apolipoprotein E | 0.2734 | 0.164862471 |
| P61026 | RAB10_HUMAN | Ras-related protein Rab-10 | 0.27349 | 0.037970868 |
| P50395 | GDIB_HUMAN | Rab GDP dissociation inhibitor beta | 0.27614 | -0.077660993 |
| Q14157 | UBP2L_HUMAN | Ubiquitin-associated protein 2-like | 0.27646 | 0.06985714 |
| Q5JPE7 | NOMO2_HUMAN | Nodal modulator 2 | 0.27718 | -0.228683957 |
| Q96CM8 | ACSF2_HUMAN | Acyl-CoA synthetase family member 2, mitochondrial | 0.27801 | -0.222362923 |
| Q14554 | PDIA5_HUMAN | Protein disulfide-isomerase A5 | 0.27807 | 0.061704571 |
| P30084 | ECHM_HUMAN | Enoyl-CoA hydratase, mitochondrial | 0.27902 | 0.032308701 |
| P0DP25 | CALM3_HUMAN | Calmodulin-3 | 0.283 | -0.063891063 |
| O15127 | SCAM2_HUMAN | Secretory carrier-associated membrane protein 2 | 0.28588 | -0.023841881 |
| Q15008 | PSMD6_HUMAN | 26S proteasome non-ATPase regulatory subunit 6 | 0.28672 | 0.066331483 |
| P62913 | RL11_HUMAN | 60S ribosomal protein L11 | 0.28852 | 0.03888062 |
| P46459 | NSF_HUMAN | Vesicle-fusing ATPase | 0.28955 | 0.086548111 |
| P40939 | ECHA_HUMAN | Trifunctional enzyme subunit alpha, mitochondrial | 0.29118 | -0.095815591 |
| P18085 | ARF4_HUMAN | ADP-ribosylation factor 4 | 0.2915 | 0.06170442 |
| P05204 | HMGN2_HUMAN | Non-histone chromosomal protein HMG-17 | 0.29176 | 0.169448776 |
| Q15046 | SYK_HUMAN | Lysine--tRNA ligase | 0.29193 | 0.131760208 |
| P12830 | CADH1_HUMAN | Cadherin-1 | 0.29505 | -0.11484381 |
| P06730-2 | IF4E_HUMAN | Isoform 2 of Eukaryotic translation initiation factor 4E | 0.29641 | -0.036919004 |
| P45880 | VDAC2_HUMAN | Voltage-dependent anion-selective channel protein 2 | 0.29731 | 0.045648488 |
| O60829 | PAGE4_HUMAN | P antigen family member 4 | 0.29774 | -0.140220704 |
| Q71UI9 | H2AV_HUMAN | Histone H2A.V | 0.29859 | 0.137581224 |
| O43242 | PSMD3_HUMAN | 26S proteasome non-ATPase regulatory subunit 3 | 0.30099 | -0.266834853 |
| Q96KP4 | CNDP2_HUMAN | Cytosolic non-specific dipeptidase | 0.30259 | -0.091092799 |
| P62424 | RL7A_HUMAN | 60S ribosomal protein L7a | 0.30421 | 0.088137267 |
| O15400 | STX7_HUMAN | Syntaxin-7 | 0.30438 | -0.108469676 |
| Q99571 | P2RX4_HUMAN | P2X purinoceptor 4 | 0.30565 | -0.116216901 |
| P38919 | IF4A3_HUMAN | Eukaryotic initiation factor 4A-III | 0.30592 | 0.149157967 |
| Q96HC4 | PDLI5_HUMAN | PDZ and LIM domain protein 5 | 0.3063 | 0.055673365 |
| Q5T4S7 | UBR4_HUMAN | E3 ubiquitin-protein ligase UBR4 | 0.30678 | -0.037523595 |
| O60716-11 | CTND1_HUMAN | Isoform 2AC of Catenin delta-1 | 0.30814 | 0.1133962 |
| Q16629 | SRSF7_HUMAN | Serine/arginine-rich splicing factor 7 | 0.3082 | 0.054328923 |
| Q15181 | IPYR_HUMAN | Inorganic pyrophosphatase | 0.30857 | -0.060969152 |
| P09467 | F16P1_HUMAN | Fructose-1,6-bisphosphatase 1 | 0.30918 | 0.22028356 |
| Q92597 | NDRG1_HUMAN | Protein NDRG1 | 0.31054 | -0.09846091 |
| P61224 | RAP1B_HUMAN | Ras-related protein Rap-1b | 0.31057 | 0.047517203 |
| P53990 | IST1_HUMAN | IST1 homolog | 0.31103 | -0.027912351 |
| P41091 | IF2G_HUMAN | Eukaryotic translation initiation factor 2 subunit 3 | 0.31272 | -0.083915564 |
| Q96TC7 | RMD3_HUMAN | Regulator of microtubule dynamics protein 3 | 0.31291 | 0.234314243 |
| Q6UXV4 | MIC27_HUMAN | MICOS complex subunit MIC27 | 0.31292 | 0.10500684 |
| P26639 | SYTC_HUMAN | Threonine--tRNA ligase, cytoplasmic | 0.3132 | -0.109436764 |
| Q99832 | TCPH_HUMAN | T-complex protein 1 subunit eta | 0.3137 | 0.0272788 |
| Q9P258 | RCC2_HUMAN | Protein RCC2 | 0.31474 | 0.039254792 |
| Q5EBM0 | CMPK2_HUMAN | UMP-CMP kinase 2, mitochondrial | 0.31497 | -0.681810515 |
| P55735 | SEC13_HUMAN | Protein SEC13 homolog | 0.31551 | 0.291960285 |
| Q9H0U4 | RAB1B_HUMAN | Ras-related protein Rab-1B | 0.31594 | 0.015111533 |
| Q15233 | NONO_HUMAN | Non-POU domain- containing octamer- binding protein | 0.31621 | 0.195720593 |
| O15372 | EIF3H_HUMAN | Eukaryotic translation initiation factor 3 subunit H | 0.3167 | 0.039956507 |
| P19823 | ITIH2_HUMAN | Inter-alpha-trypsin inhibitor heavy chain H2 | 0.31712 | -0.179775464 |
| P23381 | SYWC_HUMAN | Tryptophan--tRNA ligase, cytoplasmic | 0.3204 | -0.134688097 |
| O94905 | ERLN2_HUMAN | Erlin-2 | 0.32129 | 0.035195148 |
| P13284 | GILT_HUMAN | Gamma-interferon-inducible lysosomal thiol reductase | 0.32187 | 0.131349951 |
| P52790 | HXK3_HUMAN | Hexokinase-3 | 0.32364 | -0.105657567 |
| O43681 | ASNA_HUMAN | ATPase ASNA1 | 0.32438 | 0.080769512 |
| P35573 | GDE_HUMAN | Glycogen debranching enzyme | 0.3247 | 0.178552484 |
| Q99856 | ARI3A_HUMAN | AT-rich interactive domain-containing protein 3A | 0.325 | 0.109199381 |
| O75643 | U520_HUMAN | U5 small nuclear ribonucleoprotein 200 kDa helicase | 0.32523 | 0.122805376 |
| Q53SF7 | COBL1_HUMAN | Cordon-bleu protein-like 1 | 0.32653 | -0.118336886 |
| Q12907 | LMAN2_HUMAN | Vesicular integral-membrane protein VIP36 | 0.3281 | -0.024121321 |
| O00567 | NOP56_HUMAN | Nucleolar protein 56 | 0.33055 | 0.095881768 |
| Q96CN7 | ISOC1_HUMAN | Isochorismatase domain-containing protein 1 | 0.33063 | -0.043258977 |
| P17096 | HMGA1_HUMAN | High mobility group protein HMG-I/HMG-Y | 0.331 | 0.114589072 |
| P31153 | METK2_HUMAN | S-adenosylmethionine synthase isoform type-2 | 0.33413 | 0.099393599 |
| P09874 | PARP1_HUMAN | Poly [ADP-ribose] polymerase 1 | 0.33585 | 0.111681434 |
| Q15121 | PEA15_HUMAN | Astrocytic phosphoprotein PEA-15 | 0.3385 | -0.07389688 |
| Q99816 | TS101_HUMAN | Tumor susceptibility gene 101 protein | 0.33995 | 0.029928627 |
| P62273 | RS29_HUMAN | 40S ribosomal protein S29 | 0.34002 | 0.08097281 |
| Q5ZPR3 | CD276_HUMAN | CD276 antigen | 0.34022 | -0.111126686 |
| Q9NUB1 | ACS2L_HUMAN | Acetyl-coenzyme A synthetase 2-like, mitochondrial | 0.34082 | 0.083378888 |
| Q9BXK5 | B2L13_HUMAN | Bcl-2-like protein 13 | 0.34143 | -0.158080335 |
| Q9BRK5 | CAB45_HUMAN | 45 kDa calcium-binding protein | 0.34306 | 0.139004878 |
| P08195 | 4F2_HUMAN | 4F2 cell-surface antigen heavy chain | 0.34309 | -0.039325226 |
| P21980 | TGM2_HUMAN | Protein-glutamine gamma-glutamyltransferase 2 | 0.34374 | -0.109803772 |
| P82979 | SARNP_HUMAN | SAP domain-containing ribonucleoprotein | 0.34544 | -0.216959435 |
| P61978-3 | HNRPK_HUMAN | Isoform 3 of Heterogeneous nuclear ribonucleoprotein K | 0.3494 | 0.051436576 |
| P29590-9 | PML_HUMAN | Isoform PML-3 of Protein PML | 0.34996 | -0.073504658 |
| P11177 | ODPB_HUMAN | Pyruvate dehydrogenase E1 component subunit beta, mitochondrial | 0.34999 | 0.07522548 |
| P12270 | TPR_HUMAN | Nucleoprotein TPR | 0.35019 | 0.207578804 |
| Q15436 | SC23A_HUMAN | Protein transport protein Sec23A | 0.35575 | -0.124252402 |
| O43278 | SPIT1_HUMAN | Kunitz-type protease inhibitor 1 | 0.35608 | -0.050754213 |
| O15260 | SURF4_HUMAN | Surfeit locus protein 4 | 0.35629 | 0.033997253 |
| P37837 | TALDO_HUMAN | Transaldolase | 0.35833 | 0.035023333 |
| Q14683 | SMC1A_HUMAN | Structural maintenance of chromosomes protein 1A | 0.35894 | -0.136025624 |
| Q13445 | TMED1_HUMAN | Transmembrane emp24 domain-containing protein 1 | 0.35898 | -0.100919683 |
| P33176 | KINH_HUMAN | Kinesin-1 heavy chain | 0.36016 | -0.311456047 |
| Q9H5X1 | CIA2A_HUMAN | Cytosolic iron-sulfur assembly component 2A | 0.36045 | 0.104241941 |
| Q9Y3I0 | RTCB_HUMAN | tRNA-splicing ligase RtcB homolog | 0.36047 | -0.04983508 |
| P10809 | CH60_HUMAN | 60 kDa heat shock protein, mitochondrial | 0.36097 | -0.035221349 |
| P06454 | PTMA_HUMAN | Prothymosin alpha | 0.3614 | -0.071064534 |
| P49748 | ACADV_HUMAN | Very long-chain specific acyl-CoA dehydrogenase, mitochondrial | 0.36145 | 0.046462676 |
| Q15029 | U5S1_HUMAN | 116 kDa U5 small nuclear ribonucleoprotein component | 0.36149 | 0.046149052 |
| Q86U42 | PABP2_HUMAN | Polyadenylate-binding protein 2 | 0.36176 | 0.039851027 |
| P11940 | PABP1_HUMAN | Polyadenylate-binding protein 1 | 0.36283 | 0.040778171 |
| P61923 | COPZ1_HUMAN | Coatomer subunit zeta-1 | 0.36372 | 0.027552757 |
| Q01105 | SET_HUMAN | Protein SET | 0.36475 | -0.070070148 |
| P42167 | LAP2B_HUMAN | Lamina-associated polypeptide 2, isoforms beta/gamma | 0.36582 | 0.08331915 |
| P13804 | ETFA_HUMAN | Electron transfer flavoprotein subunit alpha, mitochondrial | 0.36611 | 0.04923766 |
| Q9H6S3 | ES8L2_HUMAN | Epidermal growth factor receptor kinase substrate 8-like protein 2 | 0.36688 | 0.117583052 |
| Q93077 | H2A1C_HUMAN | Histone H2A type 1-C | 0.36772 | 0.095385132 |
| Q92499 | DDX1_HUMAN | ATP-dependent RNA helicase DDX1 | 0.36917 | 0.029649733 |
| P62873 | GBB1_HUMAN | Guanine nucleotide-binding protein G(I)/G(S)/G(T) subunit beta-1 | 0.37024 | 0.031434771 |
| Q3LXA3 | TKFC_HUMAN | Triokinase/FMN cyclase | 0.3706 | 0.080908474 |
| Q8NBN7 | RDH13_HUMAN | Retinol dehydrogenase 13 | 0.37182 | 0.128032632 |
| O95484 | CLD9_HUMAN | Claudin-9 | 0.37199 | -0.076011687 |
| Q15691 | MARE1_HUMAN | Microtubule-associated protein RP/EB family member 1 | 0.37298 | 0.067376531 |
| Q15067 | ACOX1_HUMAN | Peroxisomal acyl-coenzyme A oxidase 1 | 0.37302 | 0.116767585 |
| O14818 | PSA7_HUMAN | Proteasome subunit alpha type-7 | 0.37412 | -0.073181046 |
| P46926 | GNPI1_HUMAN | Glucosamine-6-phosphate isomerase 1 | 0.37485 | -0.032924472 |
| P29692 | EF1D_HUMAN | Elongation factor 1-delta | 0.37573 | -0.136568581 |
| O75438 | NDUB1_HUMAN | NADH dehydrogenase [ubiquinone] 1 beta subcomplex subunit 1 | 0.37611 | -0.243322252 |
| O43143 | DHX15_HUMAN | Pre-mRNA-splicing factor ATP-dependent RNA helicase DHX15 | 0.37662 | 0.041501351 |
| Q5VT79 | AXA81_HUMAN | Annexin A8-like protein 1 | 0.37749 | -0.241035284 |
| P62081 | RS7_HUMAN | 40S ribosomal protein S7 | 0.3799 | 0.040563419 |
| O75695 | XRP2_HUMAN | Protein XRP2 | 0.38117 | 0.047002533 |
| P27487 | DPP4_HUMAN | Dipeptidyl peptidase 4 | 0.38376 | -0.033955348 |
| Q9Y5U9 | IR3IP_HUMAN | Immediate early response 3-interacting protein 1 | 0.38417 | 0.087022784 |
| Q9H8Y8-3 | GORS2_HUMAN | Isoform 3 of Golgi reassembly-stacking protein 2 | 0.38422 | 0.073717172 |
| P62841 | RS15_HUMAN | 40S ribosomal protein S15 | 0.38434 | 0.027414623 |
| Q9Y6E2 | BZW2_HUMAN | Basic leucine zipper and W2 domain-containing protein 2 | 0.38476 | 0.14825024 |
| P15121 | ALDR_HUMAN | Aldo-keto reductase family 1 member B1 | 0.38477 | 0.064193713 |
| Q9NX02 | NALP2_HUMAN | NACHT, LRR and PYD domains-containing protein 2 | 0.38591 | -0.102665094 |
| P08758 | ANXA5_HUMAN | Annexin A5 | 0.38596 | -0.025904857 |
| O14773 | TPP1_HUMAN | Tripeptidyl-peptidase 1 | 0.38633 | 0.044897797 |
| O95298 | NDUC2_HUMAN | NADH dehydrogenase [ubiquinone] 1 subunit C2 | 0.38765 | -0.126115268 |
| Q16881 | TRXR1_HUMAN | Thioredoxin reductase 1, cytoplasmic | 0.38911 | 0.054937688 |
| P20645 | MPRD_HUMAN | Cation-dependent mannose-6-phosphate receptor | 0.38917 | 0.058601571 |
| Q02218 | ODO1_HUMAN | 2-oxoglutarate dehydrogenase, mitochondrial | 0.39058 | 0.079539671 |
| Q7L5L3 | GDPD3_HUMAN | Lysophospholipase D GDPD3 | 0.39191 | -0.111864194 |
| P49207 | RL34_HUMAN | 60S ribosomal protein L34 | 0.39437 | 0.088169057 |
| P63104 | 1433Z_HUMAN | 14-3-3 protein zeta/delta | 0.39558 | -0.133808211 |
| Q14126 | DSG2_HUMAN | Desmoglein-2 | 0.39714 | 0.151186931 |
| Q8NBJ5 | GT251_HUMAN | Procollagen galactosyltransferase 1 | 0.39751 | 0.09300817 |
| P42224 | STAT1_HUMAN | Signal transducer and activator of transcription 1-alpha/beta | 0.39829 | -0.075737221 |
| Q92973 | TNPO1_HUMAN | Transportin-1 | 0.4033 | -0.066750061 |
| P13473 | LAMP2_HUMAN | Lysosome-associated membrane glycoprotein 2 | 0.40398 | 0.06047722 |
| P13645 | K1C10_HUMAN | Keratin, type I cytoskeletal 10 | 0.40455 | 0.070397232 |
| Q9BR76 | COR1B_HUMAN | Coronin-1B | 0.40455 | -0.081040131 |
| O43615 | TIM44_HUMAN | Mitochondrial import inner membrane translocase subunit TIM44 | 0.40485 | 0.103451971 |
| Q9UL25 | RAB21_HUMAN | Ras-related protein Rab-21 | 0.40604 | 0.02727296 |
| P30533 | AMRP_HUMAN | Alpha-2-macroglobulin receptor-associated protein | 0.40628 | 0.082647077 |
| Q15642 | CIP4_HUMAN | Cdc42-interacting protein 4 | 0.40745 | 0.026517897 |
| Q15428 | SF3A2_HUMAN | Splicing factor 3A subunit 2 | 0.41023 | 0.041093218 |
| P22234 | PUR6_HUMAN | Multifunctional protein ADE2 | 0.41064 | 0.059193711 |
| P62195 | PRS8_HUMAN | 26S proteasome regulatory subunit 8 | 0.41171 | 0.049009533 |
| Q13561 | DCTN2_HUMAN | Dynactin subunit 2 | 0.41246 | 0.092188527 |
| Q7Z7H5 | TMED4_HUMAN | Transmembrane emp24 domain-containing protein 4 | 0.41272 | -0.050547178 |
| Q12792 | TWF1_HUMAN | Twinfilin-1 | 0.41318 | -0.055069004 |
| O95573 | ACSL3_HUMAN | Long-chain-fatty-acid--CoA ligase 3 | 0.41562 | 0.052926277 |
| P35998 | PRS7_HUMAN | 26S proteasome regulatory subunit 7 | 0.41692 | 0.094032363 |
| O00232 | PSD12_HUMAN | 26S proteasome non-ATPase regulatory subunit 12 | 0.41758 | 0.124624075 |
| P31689 | DNJA1_HUMAN | DnaJ homolog subfamily A member 1 | 0.41812 | -0.068381034 |
| P62888 | RL30_HUMAN | 60S ribosomal protein L30 | 0.41924 | 0.02298133 |
| Q8TE68 | ES8L1_HUMAN | Epidermal growth factor receptor kinase substrate 8-like protein 1 | 0.42049 | -0.137346636 |
| O75822 | EIF3J_HUMAN | Eukaryotic translation initiation factor 3 subunit J | 0.42092 | 0.032032019 |
| Q9NUU7 | DD19A_HUMAN | ATP-dependent RNA helicase DDX19A | 0.42109 | 0.069391273 |
| Q9UQ80 | PA2G4_HUMAN | Proliferation-associated protein 2G4 | 0.42243 | -0.127305258 |
| Q9H8H3 | MET7A_HUMAN | Methyltransferase-like protein 7A | 0.42282 | 0.089493203 |
| O60443 | GSDME_HUMAN | Gasdermin-E | 0.42374 | -0.030448761 |
| Q9BTM1 | H2AJ_HUMAN | Histone H2A.J | 0.42433 | 0.120307839 |
| P78417 | GSTO1_HUMAN | Glutathione S-transferase omega-1 | 0.42605 | 0.070208812 |
| P13010 | XRCC5_HUMAN | X-ray repair cross-complementing protein 5 | 0.42786 | 0.041271673 |
| Q9HB71 | CYBP_HUMAN | Calcyclin-binding protein | 0.42859 | -0.029037312 |
| O96008 | TOM40_HUMAN | Mitochondrial import receptor subunit TOM40 homolog | 0.42929 | 0.107252082 |
| P04040 | CATA_HUMAN | Catalase | 0.42938 | -0.048143746 |
| O95433 | AHSA1_HUMAN | Activator of 90 kDa heat shock protein ATPase homolog 1 | 0.42977 | 0.050513741 |
| P13716 | HEM2_HUMAN | Delta-aminolevulinic acid dehydratase | 0.43004 | -0.142162214 |
| O00429 | DNM1L_HUMAN | Dynamin-1-like protein | 0.43017 | 0.037831113 |
| Q15637-5 | SF01_HUMAN | Isoform 5 of Splicing factor 1 | 0.4303 | 0.11050891 |
| Q99536 | VAT1_HUMAN | Synaptic vesicle membrane protein VAT-1 homolog | 0.4304 | 0.069374975 |
| P61981 | 1433G_HUMAN | 14-3-3 protein gamma | 0.43052 | -0.05649874 |
| O00273 | DFFA_HUMAN | DNA fragmentation factor subunit alpha | 0.43171 | 0.102802742 |
| Q13263 | TIF1B_HUMAN | Transcription intermediary factor 1-beta | 0.43253 | -0.044640219 |
| Q12874 | SF3A3_HUMAN | Splicing factor 3A subunit 3 | 0.4333 | 0.028454907 |
| P55060 | XPO2_HUMAN | Exportin-2 | 0.43367 | 0.028078724 |
| P51114 | FXR1_HUMAN | Fragile X mental retardation syndrome-related protein 1 | 0.43375 | -0.07766258 |
| P25788 | PSA3_HUMAN | Proteasome subunit alpha type-3 | 0.43514 | -0.025728609 |
| P36578 | RL4_HUMAN | 60S ribosomal protein L4 | 0.43563 | 0.194144929 |
| P20810-9 | ICAL_HUMAN | Isoform 9 of Calpastatin | 0.43595 | -0.039454628 |
| P53680 | AP2S1_HUMAN | AP-2 complex subunit sigma | 0.4363 | 0.041359824 |
| P25789 | PSA4_HUMAN | Proteasome subunit alpha type-4 | 0.43797 | 0.065245678 |
| Q13576 | IQGA2_HUMAN | Ras GTPase-activating-like protein IQGAP2 | 0.43827 | 0.048300499 |
| Q9UBS4 | DJB11_HUMAN | DnaJ homolog subfamily B member 11 | 0.43873 | -0.075396142 |
| P12004 | PCNA_HUMAN | Proliferating cell nuclear antigen | 0.43927 | 0.02858567 |
| P48444 | COPD_HUMAN | Coatomer subunit delta | 0.43928 | 0.043228481 |
| P05107 | ITB2_HUMAN | Integrin beta-2 | 0.43968 | 0.135390155 |
| P20073 | ANXA7_HUMAN | Annexin A7 | 0.44001 | -0.022606308 |
| Q92598 | HS105_HUMAN | Heat shock protein 105 kDa | 0.44028 | -0.030676909 |
| P49247 | RPIA_HUMAN | Ribose-5-phosphate isomerase | 0.44105 | 0.044992948 |
| Q8WUM4 | PDC6I_HUMAN | Programmed cell death 6-interacting protein | 0.44129 | -0.020409122 |
| P07858 | CATB_HUMAN | Cathepsin B | 0.4417 | 0.083578343 |
| Q12959 | DLG1_HUMAN | Disks large homolog 1 | 0.44191 | 0.096921919 |
| P02545 | LMNA_HUMAN | Prelamin-A/C | 0.44423 | 0.043843269 |
| P50990 | TCPQ_HUMAN | T-complex protein 1 subunit theta | 0.44475 | -0.085856291 |
| P20339 | RAB5A_HUMAN | Ras-related protein Rab-5A | 0.44478 | 0.031463563 |
| P04632 | CPNS1_HUMAN | Calpain small subunit 1 | 0.44668 | -0.068581337 |
| Q9UH65 | SWP70_HUMAN | Switch-associated protein 70 | 0.44671 | 0.024578189 |
| O14828 | SCAM3_HUMAN | Secretory carrier-associated membrane protein 3 | 0.44743 | 0.025549633 |
| Q9UHQ9 | NB5R1_HUMAN | NADH-cytochrome b5 reductase 1 | 0.44763 | 0.091202872 |
| Q9HC35 | EMAL4_HUMAN | Echinoderm microtubule-associated protein-like 4 | 0.44781 | 0.04528866 |
| Q9BUF5 | TBB6_HUMAN | Tubulin beta-6 chain | 0.44844 | 0.083938094 |
| Q15424-4 | SAFB1_HUMAN | Isoform 4 of Scaffold attachment factor B1 | 0.44887 | 0.069550863 |
| Q86VP6 | CAND1_HUMAN | Cullin-associated NEDD8-dissociated protein 1 | 0.44959 | 0.071342027 |
| Q15366 | PCBP2_HUMAN | Poly(rC)-binding protein 2 | 0.45128 | 0.036396457 |
| P08133 | ANXA6_HUMAN | Annexin A6 | 0.45144 | 0.030279453 |
| O75367 | H2AY_HUMAN | Core histone macro-H2A.1 | 0.45273 | 0.110482567 |
| Q9H7M9 | VISTA_HUMAN | V-type immunoglobulin domain-containing suppressor of T-cell activation | 0.45441 | -0.076080458 |
| Q03518 | TAP1_HUMAN | Antigen peptide transporter 1 | 0.455 | 0.129160651 |
| Q16831 | UPP1_HUMAN | Uridine phosphorylase 1 | 0.45532 | -0.062618883 |
| Q9Y3A6 | TMED5_HUMAN | Transmembrane emp24 domain-containing protein 5 | 0.45532 | 0.150404078 |
| Q92520 | FAM3C_HUMAN | Protein FAM3C | 0.45555 | -0.039151682 |
| P49755 | TMEDA_HUMAN | Transmembrane emp24 domain-containing protein 10 | 0.45565 | -0.026923538 |
| Q8TCJ2 | STT3B_HUMAN | Dolichyl-diphosphooligosaccharide--protein glycosyltransferase subunit STT3B | 0.45574 | 0.03145495 |
| P09651 | ROA1_HUMAN | Heterogeneous nuclear ribonucleoprotein A1 | 0.4567 | 0.08667636 |
| P49023 | PAXI_HUMAN | Paxillin | 0.45672 | -0.059508211 |
| P52566 | GDIR2_HUMAN | Rho GDP-dissociation inhibitor 2 | 0.45746 | 0.12549281 |
| Q14974 | IMB1_HUMAN | Importin subunit beta-1 | 0.45772 | -0.058668179 |
| Q9NZ01 | TECR_HUMAN | Very-long-chain enoyl-CoA reductase | 0.45934 | 0.033314184 |
| P18669 | PGAM1_HUMAN | Phosphoglycerate mutase 1 | 0.45957 | -0.02773454 |
| P67809 | YBOX1_HUMAN | Nuclease-sensitive element-binding protein 1 | 0.4598 | 0.058944717 |
| O14975 | S27A2_HUMAN | Very long-chain acyl-CoA synthetase | 0.46066 | -0.125230888 |
| Q07065 | CKAP4_HUMAN | Cytoskeleton-associated protein 4 | 0.46128 | -0.046226628 |
| P17174 | AATC_HUMAN | Aspartate aminotransferase, cytoplasmic | 0.46239 | 0.089639934 |
| Q8NE71 | ABCF1_HUMAN | ATP-binding cassette sub-family F member 1 | 0.46454 | 0.042414744 |
| O60684 | IMA7_HUMAN | Importin subunit alpha-7 | 0.46518 | 0.027009264 |
| P00367 | DHE3_HUMAN | Glutamate dehydrogenase 1, mitochondrial | 0.4652 | -0.034053291 |
| P62807 | H2B1C_HUMAN | Histone H2B type 1-C/E/F/G/I | 0.46599 | 0.089712867 |
| P11216 | PYGB_HUMAN | Glycogen phosphorylase, brain form | 0.46651 | -0.061103931 |
| P31943 | HNRH1_HUMAN | Heterogeneous nuclear ribonucleoprotein H | 0.46751 | 0.083853947 |
| P11586 | C1TC_HUMAN | C-1-tetrahydrofolate synthase, cytoplasmic | 0.46831 | 0.057787794 |
| P22087 | FBRL_HUMAN | rRNA 2'- | 0.4692 | 0.134405548 |
| P53004 | BIEA_HUMAN | Biliverdin reductase A | 0.46959 | -0.09057656 |
| P53384 | NUBP1_HUMAN | Cytosolic Fe-S cluster assembly factor NUBP1 | 0.46968 | 0.046284316 |
| Q9UNZ2 | NSF1C_HUMAN | NSFL1 cofactor p47 | 0.47083 | -0.064261927 |
| P62995 | TRA2B_HUMAN | Transformer-2 protein homolog beta | 0.47106 | 0.059498105 |
| P63241 | IF5A1_HUMAN | Eukaryotic translation initiation factor 5A-1 | 0.47109 | -0.110298082 |
| Q99829 | CPNE1_HUMAN | Copine-1 | 0.47129 | 0.070261525 |
| Q15274 | NADC_HUMAN | Nicotinate-nucleotide pyrophosphorylase [carboxylating] | 0.47168 | -0.043937399 |
| P52272 | HNRPM_HUMAN | Heterogeneous nuclear ribonucleoprotein M | 0.47177 | 0.047737205 |
| Q9NT62 | ATG3_HUMAN | Ubiquitin-like-conjugating enzyme ATG3 | 0.47243 | -0.041091943 |
| Q96HD1 | CREL1_HUMAN | Cysteine-rich with EGF-like domain protein 1 | 0.47519 | -0.090380983 |
| P07900 | HS90A_HUMAN | Heat shock protein HSP 90-alpha | 0.47567 | -0.01598886 |
| Q93009 | UBP7_HUMAN | Ubiquitin carboxyl-terminal hydrolase 7 | 0.47593 | 0.09180162 |
| Q9HDC9 | APMAP_HUMAN | Adipocyte plasma membrane-associated protein | 0.47756 | -0.041720381 |
| P36776 | LONM_HUMAN | Lon protease homolog, mitochondrial | 0.4778 | 0.062455644 |
| Q14444 | CAPR1_HUMAN | Caprin-1 | 0.47783 | 0.045753261 |
| Q9P2E9 | RRBP1_HUMAN | Ribosome-binding protein 1 | 0.47798 | -0.016570699 |
| Q9NUQ9 | FA49B_HUMAN | Protein FAM49B | 0.47838 | -0.085830089 |
| P45974 | UBP5_HUMAN | Ubiquitin carboxyl-terminal hydrolase 5 | 0.48083 | 0.042085562 |
| P21796 | VDAC1_HUMAN | Voltage-dependent anion-selective channel protein 1 | 0.48097 | 0.023183968 |
| P48047 | ATPO_HUMAN | ATP synthase subunit | 0.48123 | 0.053197346 |
| Q92544 | TM9S4_HUMAN | Transmembrane 9 superfamily member 4 | 0.48196 | 0.040112997 |
| Q7L5N7 | PCAT2_HUMAN | Lysophosphatidylcholine acyltransferase 2 | 0.48202 | 0.142459701 |
| P32119 | PRDX2_HUMAN | Peroxiredoxin-2 | 0.48258 | -0.115364666 |
| Q92841 | DDX17_HUMAN | Probable ATP-dependent RNA helicase DDX17 | 0.48433 | 0.032190438 |
| Q05519 | SRS11_HUMAN | Serine/arginine-rich splicing factor 11 | 0.4844 | -0.099744894 |
| O43493 | TGON2_HUMAN | Trans-Golgi network integral membrane protein 2 | 0.48531 | 0.028785351 |
| P00390 | GSHR_HUMAN | Glutathione reductase, mitochondrial | 0.4863 | 0.016884265 |
| P49419 | AL7A1_HUMAN | Alpha-aminoadipic semialdehyde dehydrogenase | 0.48684 | 0.055478062 |
| P00966 | ASSY_HUMAN | Argininosuccinate synthase | 0.48736 | 0.066892066 |
| P08134 | RHOC_HUMAN | Rho-related GTP-binding protein RhoC | 0.4885 | 0.022503887 |
| Q13596 | SNX1_HUMAN | Sorting nexin-1 | 0.48868 | 0.023422093 |
| Q99623 | PHB2_HUMAN | Prohibitin-2 | 0.49037 | -0.031064859 |
| Q9H1B7 | I2BPL_HUMAN | Probable E3 ubiquitin-protein ligase IRF2BPL | 0.49081 | 0.100657137 |
| Q9NS69 | TOM22_HUMAN | Mitochondrial import receptor subunit TOM22 homolog | 0.49138 | -0.050641081 |
| O15347 | HMGB3_HUMAN | High mobility group protein B3 | 0.49227 | 0.038325575 |
| Q9H223 | EHD4_HUMAN | EH domain-containing protein 4 | 0.49263 | -0.060862041 |
| Q00610 | CLH1_HUMAN | Clathrin heavy chain 1 | 0.49442 | 0.023694898 |
| P43686 | PRS6B_HUMAN | 26S proteasome regulatory subunit 6B | 0.49506 | 0.023321355 |
| P08779 | K1C16_HUMAN | Keratin, type I cytoskeletal 16 | 0.4951 | -0.035553631 |
| Q14498 | RBM39_HUMAN | RNA-binding protein 39 | 0.49521 | 0.044256123 |
| P17655 | CAN2_HUMAN | Calpain-2 catalytic subunit | 0.49522 | 0.060716539 |
| P07384 | CAN1_HUMAN | Calpain-1 catalytic subunit | 0.4956 | 0.015129762 |
| O94979 | SC31A_HUMAN | Protein transport protein Sec31A | 0.49574 | 0.02938649 |
| Q71DI3 | H32_HUMAN | Histone H3.2 | 0.49641 | 0.134536966 |
| Q9H3P7 | GCP60_HUMAN | Golgi resident protein GCP60 | 0.49664 | 0.030622749 |
| O95236 | APOL3_HUMAN | Apolipoprotein L3 | 0.49952 | -0.071279756 |
| P49720 | PSB3_HUMAN | Proteasome subunit beta type-3 | 0.49995 | 0.055340669 |
| P55327-4 | TPD52_HUMAN | Isoform 4 of Tumor protein D52 | 0.50046 | -0.058688926 |
| P22695 | QCR2_HUMAN | Cytochrome b-c1 complex subunit 2, mitochondrial | 0.5013 | -0.041625012 |
| Q9UHD8-7 | SEPT9_HUMAN | Isoform 7 of Septin-9 | 0.5018 | 0.022821428 |
| P32455 | GBP1_HUMAN | Guanylate-binding protein 1 | 0.50192 | -0.067048228 |
| P47914 | RL29_HUMAN | 60S ribosomal protein L29 | 0.50243 | 0.045700769 |
| Q68CZ2 | TENS3_HUMAN | Tensin-3 | 0.50258 | 0.055360897 |
| P20340 | RAB6A_HUMAN | Ras-related protein Rab-6A | 0.50367 | 0.039090918 |
| P22626 | ROA2_HUMAN | Heterogeneous nuclear ribonucleoproteins A2/B1 | 0.50649 | 0.033237386 |
| P69891 | HBG1_HUMAN | Hemoglobin subunit gamma-1 | 0.50661 | -0.099787153 |
| Q969V3 | NCLN_HUMAN | Nicalin | 0.50786 | -0.149477422 |
| O15498 | YKT6_HUMAN | Synaptobrevin homolog YKT6 | 0.50883 | 0.090957828 |
| Q06323 | PSME1_HUMAN | Proteasome activator complex subunit 1 | 0.51095 | -0.065488918 |
| P33241 | LSP1_HUMAN | Lymphocyte-specific protein 1 | 0.51116 | 0.202297659 |
| P50281 | MMP14_HUMAN | Matrix metalloproteinase-14 | 0.51116 | -0.134296434 |
| Q15005 | SPCS2_HUMAN | Signal peptidase complex subunit 2 | 0.51372 | -0.068385356 |
| P67936 | TPM4_HUMAN | Tropomyosin alpha-4 chain | 0.51381 | -0.037901011 |
| Q00765 | REEP5_HUMAN | Receptor expression-enhancing protein 5 | 0.51433 | -0.067798865 |
| P31150 | GDIA_HUMAN | Rab GDP dissociation inhibitor alpha | 0.51514 | -0.105893635 |
| P67812-3 | SC11A_HUMAN | Isoform 3 of Signal peptidase complex catalytic subunit SEC11A | 0.51689 | -0.017913596 |
| O60271 | JIP4_HUMAN | C-Jun-amino-terminal kinase-interacting protein 4 | 0.51734 | -0.04585334 |
| Q14624-4 | ITIH4_HUMAN | Isoform 4 of Inter-alpha-trypsin inhibitor heavy chain H4 | 0.51751 | -0.110106806 |
| Q9Y266 | NUDC_HUMAN | Nuclear migration protein nudC | 0.51772 | 0.022375572 |
| P51858 | HDGF_HUMAN | Hepatoma-derived growth factor | 0.51929 | 0.069068611 |
| Q16836 | HCDH_HUMAN | Hydroxyacyl-coenzyme A dehydrogenase, mitochondrial | 0.51976 | -0.028848628 |
| P07910 | HNRPC_HUMAN | Heterogeneous nuclear ribonucleoproteins C1/C2 | 0.51997 | 0.065746618 |
| Q29RF7 | PDS5A_HUMAN | Sister chromatid cohesion protein PDS5 homolog A | 0.52025 | 0.0839316 |
| Q14254 | FLOT2_HUMAN | Flotillin-2 | 0.52071 | -0.028991027 |
| P01920 | DQB1_HUMAN | HLA class II histocompatibility antigen, DQ beta 1 chain | 0.52146 | 0.127284488 |
| P55010 | IF5_HUMAN | Eukaryotic translation initiation factor 5 | 0.52273 | 0.052056022 |
| P17987 | TCPA_HUMAN | T-complex protein 1 subunit alpha | 0.52292 | 0.020831152 |
| P50995 | ANX11_HUMAN | Annexin A11 | 0.52487 | 0.034818465 |
| P05198 | IF2A_HUMAN | Eukaryotic translation initiation factor 2 subunit 1 | 0.52819 | 0.019204545 |
| P12277 | KCRB_HUMAN | Creatine kinase B-type | 0.52842 | 0.076152978 |
| O14617 | AP3D1_HUMAN | AP-3 complex subunit delta-1 | 0.52896 | 0.053373563 |
| P12268 | IMDH2_HUMAN | Inosine-5'-monophosphate dehydrogenase 2 | 0.52973 | 0.031388547 |
| P51572 | BAP31_HUMAN | B-cell receptor-associated protein 31 | 0.53076 | 0.036843671 |
| P16070 | CD44_HUMAN | CD44 antigen | 0.53086 | 0.098327004 |
| Q9UNH7 | SNX6_HUMAN | Sorting nexin-6 | 0.53098 | -0.029708221 |
| P14406 | CX7A2_HUMAN | Cytochrome c oxidase subunit 7A2, mitochondrial | 0.53163 | -0.018072384 |
| P69905 | HBA_HUMAN | Hemoglobin subunit alpha | 0.5324 | -0.094086965 |
| P23229-9 | ITA6_HUMAN | Isoform 9 of Integrin alpha-6 | 0.53367 | 0.046186564 |
| P61006 | RAB8A_HUMAN | Ras-related protein Rab-8A | 0.53479 | -0.081393867 |
| P00403 | COX2_HUMAN | Cytochrome c oxidase subunit 2 | 0.53527 | -0.0257818 |
| P63244 | RACK1_HUMAN | Receptor of activated protein C kinase 1 | 0.53572 | 0.031501979 |
| Q15056 | IF4H_HUMAN | Eukaryotic translation initiation factor 4H | 0.53613 | -0.080589112 |
| Q13838-2 | DX39B_HUMAN | Isoform 2 of Spliceosome RNA helicase DDX39B | 0.53765 | 0.013036158 |
| P14060 | 3BHS1_HUMAN | 3 beta-hydroxysteroid dehydrogenase/Delta 5-->4-isomerase type 1 | 0.53778 | 0.075180851 |
| O95782 | AP2A1_HUMAN | AP-2 complex subunit alpha-1 | 0.53788 | 0.04797542 |
| P49591 | SYSC_HUMAN | Serine--tRNA ligase, cytoplasmic | 0.53853 | 0.050858647 |
| Q5EB52 | MEST_HUMAN | Mesoderm-specific transcript homolog protein | 0.53914 | 0.068645825 |
| P11021 | BIP_HUMAN | Endoplasmic reticulum chaperone BiP | 0.53948 | 0.015294977 |
| P07437 | TBB5_HUMAN | Tubulin beta chain | 0.53954 | 0.062981513 |
| P26641 | EF1G_HUMAN | Elongation factor 1-gamma | 0.53981 | 0.020982315 |
| Q9H9B4 | SFXN1_HUMAN | Sideroflexin-1 | 0.54088 | -0.078412008 |
| P61313 | RL15_HUMAN | 60S ribosomal protein L15 | 0.54114 | 0.032524664 |
| P11166 | GTR1_HUMAN | Solute carrier family 2, facilitated glucose transporter member 1 | 0.54134 | -0.02708338 |
| Q08379 | GOGA2_HUMAN | Golgin subfamily A member 2 | 0.54161 | 0.078318158 |
| P20700 | LMNB1_HUMAN | Lamin-B1 | 0.54374 | 0.035309369 |
| Q29963 | 1C06_HUMAN | HLA class I histocompatibility antigen, Cw-6 alpha chain | 0.54443 | -0.086156823 |
| P04075 | ALDOA_HUMAN | Fructose-bisphosphate aldolase A | 0.54596 | -0.018120447 |
| P31930 | QCR1_HUMAN | Cytochrome b-c1 complex subunit 1, mitochondrial | 0.54649 | -0.054323405 |
| Q8N163 | CCAR2_HUMAN | Cell cycle and apoptosis regulator protein 2 | 0.54666 | 0.038589211 |
| O60664 | PLIN3_HUMAN | Perilipin-3 | 0.54714 | 0.027233675 |
| P30041 | PRDX6_HUMAN | Peroxiredoxin-6 | 0.54733 | -0.024495807 |
| Q9Y6W5 | WASF2_HUMAN | Wiskott-Aldrich syndrome protein family member 2 | 0.54872 | 0.066680788 |
| P61019 | RAB2A_HUMAN | Ras-related protein Rab-2A | 0.55 | -0.075663648 |
| P39687 | AN32A_HUMAN | Acidic leucine-rich nuclear phosphoprotein 32 family member A | 0.55013 | -0.026326582 |
| O95373 | IPO7_HUMAN | Importin-7 | 0.55052 | 0.02835571 |
| Q16698 | DECR_HUMAN | 2,4-dienoyl-CoA reductase, mitochondrial | 0.5522 | -0.100431896 |
| O95747 | OXSR1_HUMAN | Serine/threonine-protein kinase | 0.55642 | -0.039080293 |
| Q99733 | NP1L4_HUMAN | Nucleosome assembly protein 1-like 4 | 0.55651 | 0.067354045 |
| Q8WVM8 | SCFD1_HUMAN | Sec1 family domain-containing protein 1 | 0.5571 | 0.022571581 |
| O14745 | NHRF1_HUMAN | Na(+)/H(+) exchange regulatory cofactor NHE-RF1 | 0.55762 | -0.074470562 |
| P61160 | ARP2_HUMAN | Actin-related protein 2 | 0.55803 | 0.035233605 |
| P54920 | SNAA_HUMAN | Alpha-soluble NSF attachment protein | 0.55811 | -0.038539399 |
| Q96GT9 | XAGE2_HUMAN | X antigen family member 2 | 0.56024 | -0.088127073 |
| P49821 | NDUV1_HUMAN | NADH dehydrogenase [ubiquinone] flavoprotein 1, mitochondrial | 0.56048 | -0.049779564 |
| P05386 | RLA1_HUMAN | 60S acidic ribosomal protein P1 | 0.561 | -0.064275378 |
| Q9NUV9 | GIMA4_HUMAN | GTPase IMAP family member 4 | 0.56181 | 0.131656988 |
| P15924 | DESP_HUMAN | Desmoplakin | 0.56238 | 0.051803499 |
| P15428-5 | PGDH_HUMAN | Isoform 5 of 15-hydroxyprostaglandin dehydrogenase [NAD(+)] | 0.56243 | 0.050103211 |
| P22392 | NDKB_HUMAN | Nucleoside diphosphate kinase B | 0.56325 | 0.025452009 |
| P09960 | LKHA4_HUMAN | Leukotriene A-4 hydrolase | 0.5637 | 0.105646834 |
| P36957 | ODO2_HUMAN | Dihydrolipoyllysine-residue succinyltransferase component of 2-oxoglutarate dehydrogenase complex, mitochondrial | 0.56396 | 0.044194773 |
| P14902 | I23O1_HUMAN | Indoleamine 2,3-dioxygenase 1 | 0.56401 | -0.083804967 |
| P29966 | MARCS_HUMAN | Myristoylated alanine-rich C-kinase substrate | 0.56403 | 0.067282819 |
| Q15293 | RCN1_HUMAN | Reticulocalbin-1 | 0.56556 | -0.037885584 |
| P09493-3 | TPM1_HUMAN | Isoform 3 of Tropomyosin alpha-1 chain | 0.56906 | 0.103733833 |
| P0DMV9 | HS71B_HUMAN | Heat shock 70 kDa protein 1B | 0.57019 | -0.032712165 |
| Q15041 | AR6P1_HUMAN | ADP-ribosylation factor-like protein 6-interacting protein 1 | 0.5702 | 0.01981431 |
| O15144 | ARPC2_HUMAN | Actin-related protein 2/3 complex subunit 2 | 0.57222 | 0.030323602 |
| Q99541 | PLIN2_HUMAN | Perilipin-2 | 0.57225 | 0.038040329 |
| P02786 | TFR1_HUMAN | Transferrin receptor protein 1 | 0.57234 | 0.040353953 |
| P05387 | RLA2_HUMAN | 60S acidic ribosomal protein P2 | 0.57318 | -0.048118389 |
| Q9BXP5 | SRRT_HUMAN | Serrate RNA effector molecule homolog | 0.57373 | -0.037728618 |
| O75874 | IDHC_HUMAN | Isocitrate dehydrogenase [NADP] cytoplasmic | 0.57425 | 0.042892254 |
| P13667 | PDIA4_HUMAN | Protein disulfide-isomerase A4 | 0.57605 | -0.01463981 |
| Q06787 | FMR1_HUMAN | Synaptic functional regulator FMR1 | 0.57737 | 0.047582949 |
| O15143 | ARC1B_HUMAN | Actin-related protein 2/3 complex subunit 1B | 0.57745 | -0.076626893 |
| P14314 | GLU2B_HUMAN | Glucosidase 2 subunit beta | 0.57772 | -0.016777909 |
| P23434 | GCSH_HUMAN | Glycine cleavage system H protein, mitochondrial | 0.57802 | 0.165077795 |
| P53634 | CATC_HUMAN | Dipeptidyl peptidase 1 | 0.5796 | 0.095751946 |
| Q96G03 | PGM2_HUMAN | Phosphoglucomutase-2 | 0.58093 | 0.01730253 |
| P62701 | RS4X_HUMAN | 40S ribosomal protein S4, X isoform | 0.58139 | 0.026225985 |
| Q9NPH2 | INO1_HUMAN | Inositol-3-phosphate synthase 1 | 0.58227 | 0.069340173 |
| Q9BS26 | ERP44_HUMAN | Endoplasmic reticulum resident protein 44 | 0.58273 | -0.019515134 |
| Q63HN8 | RN213_HUMAN | E3 ubiquitin-protein ligase RNF213 | 0.5837 | -0.077956059 |
| Q9ULZ3 | ASC_HUMAN | Apoptosis-associated speck-like protein containing a CARD | 0.58428 | -0.082114675 |
| P07686 | HEXB_HUMAN | Beta-hexosaminidase subunit beta | 0.58525 | 0.036004687 |
| P63151 | 2ABA_HUMAN | Serine/threonine-protein phosphatase 2A 55 kDa regulatory subunit B alpha isoform | 0.58537 | -0.038082093 |
| Q99460 | PSMD1_HUMAN | 26S proteasome non-ATPase regulatory subunit 1 | 0.58577 | -0.032663123 |
| P36542 | ATPG_HUMAN | ATP synthase subunit gamma, mitochondrial | 0.58651 | 0.044433164 |
| P09012 | SNRPA_HUMAN | U1 small nuclear ribonucleoprotein A | 0.5867 | 0.015000215 |
| P40306 | PSB10_HUMAN | Proteasome subunit beta type-10 | 0.58842 | -0.066644798 |
| P00505 | AATM_HUMAN | Aspartate aminotransferase, mitochondrial | 0.59029 | -0.039471083 |
| P02749 | APOH_HUMAN | Beta-2-glycoprotein 1 | 0.59147 | -0.033320635 |
| Q99442 | SEC62_HUMAN | Translocation protein SEC62 | 0.5925 | -0.044970126 |
| Q9BRP8 | PYM1_HUMAN | Partner of Y14 and mago | 0.59297 | 0.07558199 |
| Q01082 | SPTB2_HUMAN | Spectrin beta chain, non-erythrocytic 1 | 0.59458 | -0.020923868 |
| P49327 | FAS_HUMAN | Fatty acid synthase | 0.59524 | 0.050841948 |
| P16615 | AT2A2_HUMAN | Sarcoplasmic/endoplasmic reticulum calcium ATPase 2 | 0.59591 | 0.030307965 |
| A0AVT1 | UBA6_HUMAN | Ubiquitin-like modifier-activating enzyme 6 | 0.59678 | 0.016151755 |
| Q5RI15 | COX20_HUMAN | Cytochrome c oxidase assembly protein COX20, mitochondrial | 0.59776 | -0.01971055 |
| Q14764 | MVP_HUMAN | Major vault protein | 0.59806 | -0.049363657 |
| Q16576 | RBBP7_HUMAN | Histone-binding protein RBBP7 | 0.5985 | 0.031011482 |
| P21964 | COMT_HUMAN | Catechol | 0.59911 | 0.022183183 |
| Q13510 | ASAH1_HUMAN | Acid ceramidase | 0.60006 | 0.060542331 |
| P54652 | HSP72_HUMAN | Heat shock-related 70 kDa protein 2 | 0.60203 | 0.033321773 |
| P68400 | CSK21_HUMAN | Casein kinase II subunit alpha | 0.60228 | -0.081488007 |
| P59998 | ARPC4_HUMAN | Actin-related protein 2/3 complex subunit 4 | 0.6049 | 0.016215084 |
| P30626 | SORCN_HUMAN | Sorcin | 0.60565 | -0.034035886 |
| P55072 | TERA_HUMAN | Transitional endoplasmic reticulum ATPase | 0.6057 | 0.013322485 |
| Q96CW1 | AP2M1_HUMAN | AP-2 complex subunit mu | 0.60594 | 0.052085494 |
| Q16630-2 | CPSF6_HUMAN | Isoform 2 of Cleavage and polyadenylation specificity factor subunit 6 | 0.60617 | -0.042244701 |
| O60313 | OPA1_HUMAN | Dynamin-like 120 kDa protein, mitochondrial | 0.60622 | -0.054050711 |
| Q15084 | PDIA6_HUMAN | Protein disulfide-isomerase A6 | 0.60783 | -0.018750854 |
| O43399 | TPD54_HUMAN | Tumor protein D54 | 0.60793 | -0.045197234 |
| P49257 | LMAN1_HUMAN | Protein ERGIC-53 | 0.6092 | -0.01802861 |
| P55209 | NP1L1_HUMAN | Nucleosome assembly protein 1-like 1 | 0.61066 | 0.027831451 |
| P61586 | RHOA_HUMAN | Transforming protein RhoA | 0.61187 | 0.065767368 |
| O00264 | PGRC1_HUMAN | Membrane-associated progesterone receptor component 1 | 0.61187 | 0.047789462 |
| P68871 | HBB_HUMAN | Hemoglobin subunit beta | 0.61201 | -0.10675359 |
| Q14978 | NOLC1_HUMAN | Nucleolar and coiled-body phosphoprotein 1 | 0.61233 | 0.107810465 |
| Q02878 | RL6_HUMAN | 60S ribosomal protein L6 | 0.61296 | -0.04595209 |
| P62979 | RS27A_HUMAN | Ubiquitin-40S ribosomal protein S27a | 0.61604 | 0.017310305 |
| P62820 | RAB1A_HUMAN | Ras-related protein Rab-1A | 0.61638 | -0.016743233 |
| P50213 | IDH3A_HUMAN | Isocitrate dehydrogenase [NAD] subunit alpha, mitochondrial | 0.61678 | 0.050884704 |
| Q06033 | ITIH3_HUMAN | Inter-alpha-trypsin inhibitor heavy chain H3 | 0.61695 | -0.061322031 |
| Q9C0C2 | TB182_HUMAN | 182 kDa tankyrase-1-binding protein | 0.61774 | -0.03773856 |
| P54578 | UBP14_HUMAN | Ubiquitin carboxyl-terminal hydrolase 14 | 0.61787 | 0.03136205 |
| P23246 | SFPQ_HUMAN | Splicing factor, proline- and glutamine-rich | 0.61828 | 0.049120206 |
| Q9UHB6 | LIMA1_HUMAN | LIM domain and actin-binding protein 1 | 0.6188 | 0.053303795 |
| O43670 | ZN207_HUMAN | BUB3-interacting and GLEBS motif-containing protein ZNF207 | 0.61935 | 0.041067444 |
| O95359 | TACC2_HUMAN | Transforming acidic coiled-coil-containing protein 2 | 0.61969 | -0.054947685 |
| P26583 | HMGB2_HUMAN | High mobility group protein B2 | 0.62122 | 0.042572513 |
| Q8IZP0 | ABI1_HUMAN | Abl interactor 1 | 0.62124 | 0.049558774 |
| P53801 | PTTG_HUMAN | Pituitary tumor-transforming gene 1 protein-interacting protein | 0.62184 | 0.035018408 |
| P14618 | KPYM_HUMAN | Pyruvate kinase PKM | 0.62246 | 0.029692419 |
| Q9Y696 | CLIC4_HUMAN | Chloride intracellular channel protein 4 | 0.62286 | -0.023442315 |
| Q96AG4 | LRC59_HUMAN | Leucine-rich repeat-containing protein 59 | 0.62288 | 0.048799361 |
| Q13185 | CBX3_HUMAN | Chromobox protein homolog 3 | 0.62295 | -0.046903239 |
| Q9UJ70 | NAGK_HUMAN | N-acetyl-D-glucosamine kinase | 0.62337 | -0.085600379 |
| P52788 | SPSY_HUMAN | Spermine synthase | 0.62483 | -0.050204327 |
| P04179 | SODM_HUMAN | Superoxide dismutase [Mn], mitochondrial | 0.62635 | 0.073991201 |
| Q9H2G2 | SLK_HUMAN | STE20-like serine/threonine-protein kinase | 0.62705 | -0.047798675 |
| P08621 | RU17_HUMAN | U1 small nuclear ribonucleoprotein 70 kDa | 0.6276 | 0.029436721 |
| P01111 | RASN_HUMAN | GTPase NRas | 0.62778 | -0.011794333 |
| P12814 | ACTN1_HUMAN | Alpha-actinin-1 | 0.62784 | -0.009282639 |
| Q16539 | MK14_HUMAN | Mitogen-activated protein kinase 14 | 0.62844 | 0.020450865 |
| O43776 | SYNC_HUMAN | Asparagine--tRNA ligase, cytoplasmic | 0.62915 | 0.013781031 |
| Q13162 | PRDX4_HUMAN | Peroxiredoxin-4 | 0.62949 | -0.045346803 |
| Q9Y6U3 | ADSV_HUMAN | Adseverin | 0.62985 | -0.116157245 |
| P21912 | SDHB_HUMAN | Succinate dehydrogenase [ubiquinone] iron-sulfur subunit, mitochondrial | 0.63067 | -0.04017563 |
| O60841 | IF2P_HUMAN | Eukaryotic translation initiation factor 5B | 0.63082 | 0.038485696 |
| Q9Y2J2 | E41L3_HUMAN | Band 4.1-like protein 3 | 0.63086 | -0.022364535 |
| Q9Y6N5 | SQOR_HUMAN | Sulfide:quinone oxidoreductase, mitochondrial | 0.63126 | 0.06752629 |
| P00488 | F13A_HUMAN | Coagulation factor XIII A chain | 0.63276 | 0.089423403 |
| P25787 | PSA2_HUMAN | Proteasome subunit alpha type-2 | 0.63287 | -0.04182521 |
| P35555 | FBN1_HUMAN | Fibrillin-1 | 0.63314 | 0.104052981 |
| P46783 | RS10_HUMAN | 40S ribosomal protein S10 | 0.63619 | 0.044216377 |
| Q9NQC3 | RTN4_HUMAN | Reticulon-4 | 0.63645 | -0.013187381 |
| P27824 | CALX_HUMAN | Calnexin | 0.63715 | 0.014443464 |
| Q9H6K4 | OPA3_HUMAN | Optic atrophy 3 protein | 0.63902 | -0.014646272 |
| O75915 | PRAF3_HUMAN | PRA1 family protein 3 | 0.63991 | 0.02188581 |
| P62937 | PPIA_HUMAN | Peptidyl-prolyl cis-trans isomerase A | 0.64021 | 0.017299932 |
| P08754 | GNAI3_HUMAN | Guanine nucleotide-binding protein G(i) subunit alpha | 0.64041 | 0.064578389 |
| P51153 | RAB13_HUMAN | Ras-related protein Rab-13 | 0.64044 | 0.07272546 |
| P11215 | ITAM_HUMAN | Integrin alpha-M | 0.64044 | 0.069356311 |
| Q93050 | VPP1_HUMAN | V-type proton ATPase 116 kDa subunit a isoform 1 | 0.64205 | -0.025843997 |
| P18206 | VINC_HUMAN | Vinculin | 0.64315 | -0.027135465 |
| P38606 | VATA_HUMAN | V-type proton ATPase catalytic subunit A | 0.64448 | 0.038516964 |
| P62241 | RS8_HUMAN | 40S ribosomal protein S8 | 0.64494 | 0.030979561 |
| Q15365 | PCBP1_HUMAN | Poly(rC)-binding protein 1 | 0.64512 | 0.025688076 |
| P06753-2 | TPM3_HUMAN | Isoform 2 of Tropomyosin alpha-3 chain | 0.64562 | 0.05272374 |
| P04839 | CY24B_HUMAN | Cytochrome b-245 heavy chain | 0.64581 | 0.065085371 |
| Q96TA1 | NIBL1_HUMAN | Niban-like protein 1 | 0.64624 | -0.02900205 |
| Q9C075 | K1C23_HUMAN | Keratin, type I cytoskeletal 23 | 0.64699 | 0.055203035 |
| O00217 | NDUS8_HUMAN | NADH dehydrogenase [ubiquinone] iron-sulfur protein 8, mitochondrial | 0.65013 | -0.024096547 |
| Q14108 | SCRB2_HUMAN | Lysosome membrane protein 2 | 0.65079 | 0.08671088 |
| P16435 | NCPR_HUMAN | NADPH--cytochrome P450 reductase | 0.65225 | 0.01012587 |
| P27105 | STOM_HUMAN | Erythrocyte band 7 integral membrane protein | 0.65347 | -0.032154233 |
| P47755 | CAZA2_HUMAN | F-actin-capping protein subunit alpha-2 | 0.65383 | -0.01415966 |
| P56537 | IF6_HUMAN | Eukaryotic translation initiation factor 6 | 0.65561 | 0.043534207 |
| P00491 | PNPH_HUMAN | Purine nucleoside phosphorylase | 0.65666 | -0.019348673 |
| O00186 | STXB3_HUMAN | Syntaxin-binding protein 3 | 0.65738 | -0.055283622 |
| P51665 | PSMD7_HUMAN | 26S proteasome non-ATPase regulatory subunit 7 | 0.65785 | -0.053046265 |
| P13861 | KAP2_HUMAN | cAMP-dependent protein kinase type II-alpha regulatory subunit | 0.65792 | -0.057237197 |
| Q92896 | GSLG1_HUMAN | Golgi apparatus protein 1 | 0.65843 | 0.0232583 |
| P13987 | CD59_HUMAN | CD59 glycoprotein | 0.65885 | 0.020131024 |
| P13727 | PRG2_HUMAN | Bone marrow proteoglycan | 0.66011 | -0.059762471 |
| Q8NC51 | PAIRB_HUMAN | Plasminogen activator inhibitor 1 RNA-binding protein | 0.66042 | 0.04886618 |
| P09497-2 | CLCB_HUMAN | Isoform Non-brain of Clathrin light chain B | 0.66113 | -0.022122708 |
| P14923 | PLAK_HUMAN | Junction plakoglobin | 0.66153 | 0.046062714 |
| P46937 | YAP1_HUMAN | Transcriptional coactivator YAP1 | 0.66268 | 0.057977734 |
| Q7L2H7 | EIF3M_HUMAN | Eukaryotic translation initiation factor 3 subunit M | 0.6627 | -0.022572052 |
| Q969E2 | SCAM4_HUMAN | Secretory carrier-associated membrane protein 4 | 0.66285 | -0.045971608 |
| P46779 | RL28_HUMAN | 60S ribosomal protein L28 | 0.6631 | -0.038847592 |
| O15173 | PGRC2_HUMAN | Membrane-associated progesterone receptor component 2 | 0.66379 | -0.026275596 |
| P13073 | COX41_HUMAN | Cytochrome c oxidase subunit 4 isoform 1, mitochondrial | 0.66389 | -0.021750763 |
| P31948 | STIP1_HUMAN | Stress-induced-phosphoprotein 1 | 0.66536 | -0.030512131 |
| P49321 | NASP_HUMAN | Nuclear autoantigenic sperm protein | 0.66603 | -0.015194139 |
| P56385 | ATP5I_HUMAN | ATP synthase subunit e, mitochondrial | 0.66648 | 0.094526181 |
| P00915 | CAH1_HUMAN | Carbonic anhydrase 1 | 0.66826 | -0.077228984 |
| Q13813 | SPTN1_HUMAN | Spectrin alpha chain, non-erythrocytic 1 | 0.66874 | -0.035552795 |
| P62140 | PP1B_HUMAN | Serine/threonine-protein phosphatase PP1-beta catalytic subunit | 0.66917 | 0.031376864 |
| P08567 | PLEK_HUMAN | Pleckstrin | 0.66947 | 0.080473858 |
| P28838 | AMPL_HUMAN | Cytosol aminopeptidase | 0.67003 | -0.047660384 |
| P05165 | PCCA_HUMAN | Propionyl-CoA carboxylase alpha chain, mitochondrial | 0.67025 | -0.079097078 |
| P30740 | ILEU_HUMAN | Leukocyte elastase inhibitor | 0.67029 | -0.051014782 |
| Q9NP72 | RAB18_HUMAN | Ras-related protein Rab-18 | 0.6705 | 0.087394147 |
| Q7Z3D6 | GLUCM_HUMAN | D-glutamate cyclase, mitochondrial | 0.67306 | 0.026719913 |
| Q8IV08 | PLD3_HUMAN | Phospholipase D3 | 0.67345 | 0.082914354 |
| P06733 | ENOA_HUMAN | Alpha-enolase | 0.67417 | 0.016849415 |
| P55036 | PSMD4_HUMAN | 26S proteasome non-ATPase regulatory subunit 4 | 0.67526 | -0.029557526 |
| P25815 | S100P_HUMAN | Protein S100-P | 0.67585 | -0.051538 |
| O43707 | ACTN4_HUMAN | Alpha-actinin-4 | 0.67688 | -0.023931752 |
| O43617 | TPPC3_HUMAN | Trafficking protein particle complex subunit 3 | 0.67748 | -0.03923136 |
| Q04917 | 1433F_HUMAN | 14-3-3 protein eta | 0.6778 | -0.021925892 |
| P62070 | RRAS2_HUMAN | Ras-related protein R-Ras2 | 0.67831 | -0.044766786 |
| P13693 | TCTP_HUMAN | Translationally-controlled tumor protein | 0.67859 | 0.013431197 |
| P68104 | EF1A1_HUMAN | Elongation factor 1-alpha 1 | 0.67889 | 0.014321792 |
| P43490 | NAMPT_HUMAN | Nicotinamide phosphoribosyltransferase | 0.67893 | 0.044069236 |
| P13928 | ANXA8_HUMAN | Annexin A8 | 0.67916 | -0.055716189 |
| Q86SG5 | S1A7A_HUMAN | Protein S100-A7A | 0.67954 | 0.111137512 |
| Q92616 | GCN1_HUMAN | eIF-2-alpha kinase activator GCN1 | 0.68109 | 0.071376775 |
| Q92542 | NICA_HUMAN | Nicastrin | 0.68196 | -0.063138073 |
| O43390 | HNRPR_HUMAN | Heterogeneous nuclear ribonucleoprotein R | 0.68241 | 0.041125522 |
| P61077 | UB2D3_HUMAN | Ubiquitin-conjugating enzyme E2 D3 | 0.68355 | 0.034967635 |
| O76021 | RL1D1_HUMAN | Ribosomal L1 domain-containing protein 1 | 0.68475 | 0.061755602 |
| P08579 | RU2B_HUMAN | U2 small nuclear ribonucleoprotein B'' | 0.68477 | 0.0285003 |
| Q9UBI6 | GBG12_HUMAN | Guanine nucleotide-binding protein G(I)/G(S)/G(O) subunit gamma- 12 | 0.68599 | 0.05147206 |
| P23141 | EST1_HUMAN | Liver carboxylesterase 1 | 0.68769 | -0.077759657 |
| Q9HD45 | TM9S3_HUMAN | Transmembrane 9 superfamily member 3 | 0.6887 | -0.043320157 |
| P21281 | VATB2_HUMAN | V-type proton ATPase subunit B, brain isoform | 0.68974 | 0.037376705 |
| P42126 | ECI1_HUMAN | Enoyl-CoA delta isomerase 1, mitochondrial | 0.69058 | 0.027781354 |
| P00338 | LDHA_HUMAN | L-lactate dehydrogenase A chain | 0.69215 | 0.010335958 |
| O94776 | MTA2_HUMAN | Metastasis-associated protein MTA2 | 0.69312 | 0.044807712 |
| P07195 | LDHB_HUMAN | L-lactate dehydrogenase B chain | 0.69322 | -0.033190187 |
| P31946-2 | 1433B_HUMAN | Isoform Short of 14-3-3 protein beta/alpha | 0.69337 | -0.022404677 |
| P06396-2 | GELS_HUMAN | Isoform 2 of Gelsolin | 0.69347 | 0.032174869 |
| Q99735 | MGST2_HUMAN | Microsomal glutathione S-transferase 2 | 0.69443 | 0.041814928 |
| Q00839 | HNRPU_HUMAN | Heterogeneous nuclear ribonucleoprotein U | 0.69563 | 0.028037913 |
| P62805 | H4_HUMAN | Histone H4 | 0.69603 | 0.048403687 |
| P06748 | NPM_HUMAN | Nucleophosmin | 0.69626 | -0.046956185 |
| P53618 | COPB_HUMAN | Coatomer subunit beta | 0.69855 | -0.040783198 |
| P30040 | ERP29_HUMAN | Endoplasmic reticulum resident protein 29 | 0.69858 | -0.035112093 |
| O00159 | MYO1C_HUMAN | Unconventional myosin-Ic | 0.7001 | 0.027816775 |
| Q86V81 | THOC4_HUMAN | THO complex subunit 14 | 0.70072 | 0.037436762 |
| O00231 | PSD11_HUMAN | 26S proteasome non-ATPase regulatory subunit 11 | 0.70165 | 0.013899009 |
| P62244 | RS15A_HUMAN | 40S ribosomal protein S15a | 0.70212 | -0.046880829 |
| P60953 | CDC42_HUMAN | Cell division control protein 42 homolog | 0.70334 | 0.015915676 |
| P59666 | DEF3_HUMAN | Neutrophil defensin 3 | 0.70496 | 0.148815157 |
| Q6DD88 | ATLA3_HUMAN | Atlastin-3 | 0.70582 | 0.019193992 |
| P36871 | PGM1_HUMAN | Phosphoglucomutase-1 | 0.70702 | 0.020009007 |
| Q9BRF8 | CPPED_HUMAN | Serine/threonine-protein phosphatase CPPED1 | 0.7071 | -0.019619383 |
| P38646 | GRP75_HUMAN | Stress-70 protein, mitochondrial | 0.70753 | 0.035162603 |
| O95466 | FMNL1_HUMAN | Formin-like protein 1 | 0.70791 | 0.049507752 |
| Q9HC38-2 | GLOD4_HUMAN | Isoform 2 of Glyoxalase domain-containing protein 4 | 0.70892 | -0.096422203 |
| P08238 | HS90B_HUMAN | Heat shock protein HSP 90-beta | 0.70974 | -0.009181485 |
| Q7Z6Z7 | HUWE1_HUMAN | E3 ubiquitin-protein ligase HUWE1 | 0.70988 | 0.079341039 |
| P68371 | TBB4B_HUMAN | Tubulin beta-4B chain | 0.71041 | 0.032351003 |
| Q14697 | GANAB_HUMAN | Neutral alpha-glucosidase AB | 0.71087 | 0.014786737 |
| P28331-2 | NDUS1_HUMAN | Isoform 2 of NADH-ubiquinone oxidoreductase 75 kDa subunit, mitochondrial | 0.71137 | -0.020720137 |
| P30419 | NMT1_HUMAN | Glycylpeptide N-tetradecanoyltransferase 1 | 0.71187 | 0.056859157 |
| P51659 | DHB4_HUMAN | Peroxisomal multifunctional enzyme type 2 | 0.71247 | 0.03513064 |
| P49589 | SYCC_HUMAN | Cysteine--tRNA ligase, cytoplasmic | 0.71259 | 0.027382723 |
| P62256 | UBE2H_HUMAN | Ubiquitin-conjugating enzyme E2 H | 0.71259 | -0.026302445 |
| Q8WTP9 | XAGE3_HUMAN | X antigen family member 3 | 0.71287 | -0.083184401 |
| Q9UHG3 | PCYOX_HUMAN | Prenylcysteine oxidase 1 | 0.71295 | 0.027491316 |
| O95833 | CLIC3_HUMAN | Chloride intracellular channel protein 3 | 0.71307 | -0.037029743 |
| Q9UJS0 | CMC2_HUMAN | Calcium-binding mitochondrial carrier protein Aralar2 | 0.7139 | -0.019956946 |
| Q9P035 | HACD3_HUMAN | Very-long-chain (3R)-3-hydroxyacyl-CoA dehydratase 3 | 0.71393 | 0.039409008 |
| Q9BQE5 | APOL2_HUMAN | Apolipoprotein L2 | 0.714 | -0.028894374 |
| Q14696 | MESD_HUMAN | LRP chaperone MESD | 0.71433 | -0.033993604 |
| P08575 | PTPRC_HUMAN | Receptor-type tyrosine-protein phosphatase C | 0.71504 | -0.079022174 |
| Q567U6 | CCD93_HUMAN | Coiled-coil domain-containing protein 93 | 0.71601 | -0.078177634 |
| Q9UN86 | G3BP2_HUMAN | Ras GTPase-activating protein-binding protein 2 | 0.71605 | 0.009482336 |
| P37802 | TAGL2_HUMAN | Transgelin-2 | 0.71697 | 0.026367538 |
| P23193 | TCEA1_HUMAN | Transcription elongation factor A protein 1 | 0.71799 | 0.016966268 |
| Q13435 | SF3B2_HUMAN | Splicing factor 3B subunit 2 | 0.71834 | 0.014926747 |
| Q99497 | PARK7_HUMAN | Protein/nucleic acid deglycase DJ-1 | 0.71913 | -0.049327264 |
| P04844 | RPN2_HUMAN | Dolichyl-diphosphooligosaccharide--protein glycosyltransferase subunit 2 | 0.71949 | -0.009825239 |
| P60033 | CD81_HUMAN | CD81 antigen | 0.7199 | -0.038702973 |
| Q96AE4 | FUBP1_HUMAN | Far upstream element-binding protein 1 | 0.72108 | 0.041984655 |
| Q9BVK6 | TMED9_HUMAN | Transmembrane emp24 domain-containing protein 9 | 0.72226 | -0.02994384 |
| P14598 | NCF1_HUMAN | Neutrophil cytosol factor 1 | 0.72379 | -0.058520339 |
| Q13126 | MTAP_HUMAN | S-methyl-5'-thioadenosine phosphorylase | 0.72385 | 0.047142451 |
| P68363 | TBA1B_HUMAN | Tubulin alpha-1B chain | 0.72469 | 0.017203947 |
| Q9UNS2 | CSN3_HUMAN | COP9 signalasome complex sununit 3 | 0.72529 | -0.068120534 |
| P09669 | COX6C_HUMAN | Cytochrome c oxidase subunit 6C | 0.72635 | -0.026092546 |
| Q9Y678 | COPG1_HUMAN | Coatomer subunit gamma-1 | 0.72682 | 0.024149425 |
| P62899 | RL31_HUMAN | 60S ribosomal protein L31 | 0.72686 | 0.025367869 |
| P05120 | PAI2_HUMAN | Plasminogen activator inhibitor 2 | 0.7279 | 0.047370785 |
| Q01518 | CAP1_HUMAN | Adenylyl cyclase-associated protein 1 | 0.72969 | -0.023978257 |
| Q52LJ0 | FA98B_HUMAN | Protein FAM98B | 0.73124 | -0.019913059 |
| O95831 | AIFM1_HUMAN | Apoptosis-inducing factor 1, mitochondrial | 0.73297 | 0.02190037 |
| P54727 | RD23B_HUMAN | UV excision repair protein RAD23 homolog B | 0.73313 | 0.030088873 |
| P28074 | PSB5_HUMAN | Proteasome subunit beta type-5 | 0.73417 | 0.075965358 |
| P49407 | ARRB1_HUMAN | Beta-arrestin-1 | 0.73431 | 0.02098385 |
| P02792 | FRIL_HUMAN | Ferritin light chain | 0.73632 | 0.075646543 |
| Q9NR28 | DBLOH_HUMAN | Diablo homolog, mitochondrial | 0.73636 | 0.015238292 |
| P37108 | SRP14_HUMAN | Signal recognition particle 14 kDa protein | 0.73773 | -0.033216572 |
| Q8WXF1 | PSPC1_HUMAN | Paraspeckle component 1 | 0.73843 | -0.021999701 |
| Q9BT09 | CNPY3_HUMAN | Protein canopy homolog 3 | 0.73868 | 0.03648068 |
| O75131 | CPNE3_HUMAN | Copine-3 | 0.7387 | -0.032350152 |
| O76094 | SRP72_HUMAN | Signal recognition particle subunit SRP72 | 0.73894 | 0.024479571 |
| P02751 | FINC_HUMAN | Fibronectin | 0.74007 | 0.024769097 |
| P15954 | COX7C_HUMAN | Cytochrome c oxidase subunit 7C, mitochondrial | 0.74099 | -0.026407047 |
| Q86UE4 | LYRIC_HUMAN | Protein LYRIC | 0.7418 | 0.069002852 |
| P30048 | PRDX3_HUMAN | Thioredoxin-dependent peroxide reductase, mitochondrial | 0.74201 | -0.032516469 |
| P11310-2 | ACADM_HUMAN | Isoform 2 of Medium-chain specific acyl-CoA dehydrogenase, mitochondrial | 0.74337 | -0.017704958 |
| P14550 | AK1A1_HUMAN | Aldo-keto reductase family 1 member A1 | 0.74382 | 0.018354507 |
| P07741 | APT_HUMAN | Adenine phosphoribosyltransferase | 0.74385 | -0.012430529 |
| P21399 | ACOC_HUMAN | Cytoplasmic aconitate hydratase | 0.74388 | -0.036077911 |
| P61626 | LYSC_HUMAN | Lysozyme C | 0.74425 | 0.082813324 |
| Q15459 | SF3A1_HUMAN | Splicing factor 3A subunit 1 | 0.74464 | -0.018715092 |
| Q00325-2 | MPCP_HUMAN | Isoform B of Phosphate carrier protein, mitochondrial | 0.74478 | -0.013756814 |
| Q8N1F7 | NUP93_HUMAN | Nuclear pore complex protein Nup93 | 0.74508 | -0.015669026 |
| P21333 | FLNA_HUMAN | Filamin-A | 0.74533 | 0.037958581 |
| Q96B49 | TOM6_HUMAN | Mitochondrial import receptor subunit TOM6 homolog | 0.74553 | -0.030855826 |
| Q92882 | OSTF1_HUMAN | Osteoclast- stimulating factor 1 | 0.74661 | 0.038190898 |
| P04899 | GNAI2_HUMAN | Guanine nucleotide-binding protein G(i) subunit alpha-2 | 0.74722 | 0.023545261 |
| P61225 | RAP2B_HUMAN | Ras-related protein Rap-2b | 0.74753 | 0.011771496 |
| P50454 | SERPH_HUMAN | Serpin H1 | 0.74821 | -0.026533774 |
| Q9ULA0 | DNPEP_HUMAN | Aspartyl aminopeptidase | 0.74912 | 0.045671504 |
| P36955 | PEDF_HUMAN | Pigment epithelium-derived factor | 0.74928 | -0.043386212 |
| P30038 | AL4A1_HUMAN | Delta-1-pyrroline-5-carboxylate dehydrogenase, mitochondrial | 0.74999 | 0.032336619 |
| O60784 | TOM1_HUMAN | Target of Myb protein 1 | 0.75073 | -0.01410247 |
| P01023 | A2MG_HUMAN | Alpha-2-macroglobulin | 0.75205 | -0.062458171 |
| P48739-2 | PIPNB_HUMAN | Isoform 2 of Phosphatidylinositol transfer protein beta isoform | 0.75275 | -0.051241502 |
| A1L0T0 | ILVBL_HUMAN | Acetolactate synthase-like protein | 0.75286 | -0.012125673 |
| Q9Y262 | EIF3L_HUMAN | Eukaryotic translation initiation factor 3 subunit L | 0.75324 | 0.034354918 |
| Q1KMD3 | HNRL2_HUMAN | Heterogeneous nuclear ribonucleoprotein U-like protein 2 | 0.75415 | -0.03864293 |
| Q9Y5Z4 | HEBP2_HUMAN | Heme-binding protein 2 | 0.75558 | -0.035097972 |
| Q07955 | SRSF1_HUMAN | Serine/arginine-rich splicing factor 1 | 0.75611 | 0.029708017 |
| P40121 | CAPG_HUMAN | Macrophage-capping protein | 0.75626 | 0.047574016 |
| Q9HA64 | KT3K_HUMAN | Ketosamine-3-kinase | 0.75736 | -0.024584578 |
| Q12906-7 | ILF3_HUMAN | Isoform 7 of Interleukin enhancer-binding factor 3 | 0.75837 | 0.01349329 |
| Q96QR8 | PURB_HUMAN | Transcriptional activator protein Pur-beta | 0.75976 | 0.080877643 |
| P30046 | DOPD_HUMAN | D-dopachrome decarboxylase | 0.76168 | 0.053883835 |
| P20042 | IF2B_HUMAN | Eukaryotic translation initiation factor 2 subunit 2 | 0.76287 | -0.040408597 |
| P46776 | RL27A_HUMAN | 60S ribosomal protein L27a | 0.76354 | -0.053499363 |
| P32456 | GBP2_HUMAN | Guanylate-binding protein 2 | 0.76373 | 0.01910346 |
| P16144 | ITB4_HUMAN | Integrin beta-4 | 0.76486 | 0.023822534 |
| P15170 | ERF3A_HUMAN | Eukaryotic peptide chain release factor GTP-binding subunit ERF3A | 0.76581 | -0.02145834 |
| Q14103 | HNRPD_HUMAN | Heterogeneous nuclear ribonucleoprotein D0 | 0.76645 | 0.062426205 |
| Q8NFQ8 | TOIP2_HUMAN | Torsin-1A-interacting protein 2 | 0.76734 | 0.052148638 |
| P16401 | H15_HUMAN | Histone H1.5 | 0.7681 | 0.024296843 |
| P08729 | K2C7_HUMAN | Keratin, type II cytoskeletal 7 | 0.76859 | 0.021352735 |
| P38117 | ETFB_HUMAN | Electron transfer flavoprotein subunit beta | 0.76863 | 0.045617748 |
| Q01130 | SRSF2_HUMAN | Serine/arginine-rich splicing factor 2 | 0.77081 | 0.012030233 |
| P24534 | EF1B_HUMAN | Elongation factor 1-beta | 0.77178 | -0.009338082 |
| Q6P9B6 | MEAK7_HUMAN | MTOR associated protein MEAK7 | 0.77178 | 0.04633921 |
| O75954 | TSN9_HUMAN | Tetraspanin-9 | 0.77211 | -0.029869421 |
| P80723 | BASP1_HUMAN | Brain acid soluble protein 1 | 0.7722 | -0.009277127 |
| P78371 | TCPB_HUMAN | T-complex protein 1 subunit beta | 0.77266 | 0.005543218 |
| P52209 | 6PGD_HUMAN | 6-phosphogluconate dehydrogenase, decarboxylating | 0.77276 | 0.025688378 |
| Q8IZ83 | A16A1_HUMAN | Aldehyde dehydrogenase family 16 member A1 | 0.77301 | 0.034169758 |
| P61106 | RAB14_HUMAN | Ras-related protein Rab-14 | 0.77436 | -0.02443462 |
| Q86UP2 | KTN1_HUMAN | Kinectin | 0.77496 | 0.024228532 |
| Q9Y6Q1 | CAN6_HUMAN | Calpain-6 | 0.77586 | -0.035699162 |
| Q9NSE4 | SYIM_HUMAN | Isoleucine--tRNA ligase, mitochondrial | 0.77915 | -0.037877613 |
| P09211 | GSTP1_HUMAN | Glutathione S-transferase P | 0.77922 | -0.023104415 |
| Q9P2J5 | SYLC_HUMAN | Leucine--tRNA ligase, cytoplasmic | 0.78074 | 0.031853227 |
| Q8TCT9 | HM13_HUMAN | Minor histocompatibility antigen H13 | 0.78146 | 0.04877766 |
| Q7Z4W1 | DCXR_HUMAN | L-xylulose reductase | 0.78164 | -0.021660356 |
| P50502 | F10A1_HUMAN | Hsc70-interacting protein | 0.7824 | -0.00996605 |
| P08574 | CY1_HUMAN | Cytochrome c1, heme protein, mitochondrial | 0.78272 | -0.016545296 |
| Q6ZT62 | BGIN_HUMAN | Bargin | 0.78402 | -0.022567009 |
| P20742 | PZP_HUMAN | Pregnancy zone protein | 0.78471 | -0.035817242 |
| Q9NX40 | OCAD1_HUMAN | OCIA domain-containing protein 1 | 0.78486 | 0.01947524 |
| Q15417 | CNN3_HUMAN | Calponin-3 | 0.78526 | 0.034995432 |
| P08473 | NEP_HUMAN | Neprilysin | 0.7854 | -0.015230211 |
| A0FGR8 | ESYT2_HUMAN | Extended synaptotagmin-2 | 0.78688 | 0.016397231 |
| Q86UX7 | URP2_HUMAN | Fermitin family homolog 3 | 0.78723 | 0.047866454 |
| Q96CX2 | KCD12_HUMAN | BTB/POZ domain- containing protein KCTD12 | 0.78865 | 0.037519603 |
| P62258 | 1433E_HUMAN | 14-3-3 protein epsilon | 0.78914 | -0.008703485 |
| P13647 | K2C5_HUMAN | Keratin, type II cytoskeletal 5 | 0.79012 | 0.013140037 |
| P35222 | CTNB1_HUMAN | Catenin beta-1 | 0.79047 | -0.018756426 |
| P13796 | PLSL_HUMAN | Plastin-2 | 0.79136 | 0.047357196 |
| P11142 | HSP7C_HUMAN | Heat shock cognate 71 kDa protein | 0.79217 | -0.013784458 |
| P12956 | XRCC6_HUMAN | X-ray repair cross-complementing protein 6 | 0.79219 | 0.01454346 |
| P41218 | MNDA_HUMAN | Myeloid cell nuclear differentiation antigen | 0.79369 | -0.039084997 |
| P53396 | ACLY_HUMAN | ATP-citrate synthase | 0.79441 | 0.020132461 |
| P40616 | ARL1_HUMAN | ADP-ribosylation factor-like protein 1 | 0.79521 | 0.046179927 |
| P10316 | 1A69_HUMAN | HLA class I histocompatibility antigen, A-69 alpha chain | 0.7961 | -0.032802177 |
| Q9H444 | CHM4B_HUMAN | Charged multivesicular body protein 4b | 0.79673 | 0.013438028 |
| O15533 | TPSN_HUMAN | Tapasin | 0.79697 | -0.022232145 |
| P02794 | FRIH_HUMAN | Ferritin heavy chain | 0.7971 | 0.040657144 |
| P49189 | AL9A1_HUMAN | 4-trimethylaminobutyraldehyde dehydrogenase | 0.79881 | -0.014660813 |
| P13674-3 | P4HA1_HUMAN | Isoform 3 of Prolyl 4-hydroxylase subunit alpha-1 | 0.79907 | -0.032459624 |
| P00167 | CYB5_HUMAN | Cytochrome b5 | 0.79964 | 0.017119606 |
| P43304 | GPDM_HUMAN | Glycerol-3-phosphate dehydrogenase, mitochondrial | 0.80123 | 0.019462017 |
| Q14694 | UBP10_HUMAN | Ubiquitin carboxyl-terminal hydrolase 10 | 0.80152 | 0.026803713 |
| Q6PIU2 | NCEH1_HUMAN | Neutral cholesterol ester hydrolase 1 | 0.80265 | 0.018184825 |
| P05109 | S10A8_HUMAN | Protein S100-A8 | 0.80279 | 0.040959682 |
| Q16851 | UGPA_HUMAN | UTP--glucose-1-phosphate uridylyltransferase | 0.80303 | -0.014719966 |
| P15328 | FOLR1_HUMAN | Folate receptor alpha | 0.80425 | 0.023914701 |
| P19367 | HXK1_HUMAN | Hexokinase-1 | 0.80437 | 0.007178342 |
| Q8IYB3 | SRRM1_HUMAN | Serine/arginine repetitive matrix protein 1 | 0.80447 | -0.032990169 |
| P62333 | PRS10_HUMAN | 26S proteasome regulatory subunit 10B | 0.80461 | 0.036591716 |
| Q14956 | GPNMB_HUMAN | Transmembrane glycoprotein NMB | 0.80554 | 0.056639178 |
| O00442 | RTCA_HUMAN | RNA 3'-terminal phosphate cyclase | 0.80641 | -0.026219965 |
| P80303 | NUCB2_HUMAN | Nucleobindin-2 | 0.80652 | -0.024006627 |
| P40926 | MDHM_HUMAN | Malate dehydrogenase, mitochondrial | 0.80658 | -0.015631534 |
| P49588 | SYAC_HUMAN | Alanine--tRNA ligase, cytoplasmic | 0.80704 | -0.050910913 |
| P15153 | RAC2_HUMAN | Ras-related C3 botulinum toxin substrate 2 | 0.80724 | -0.052757325 |
| Q16134 | ETFD_HUMAN | Electron transfer flavoprotein-ubiquinone oxidoreductase, mitochondrial | 0.80797 | -0.013568711 |
| P02042 | HBD_HUMAN | Hemoglobin subunit delta | 0.80908 | -0.045866117 |
| P56192 | SYMC_HUMAN | Methionine--tRNA ligase, cytoplasmic | 0.80953 | 0.007705119 |
| P62277 | RS13_HUMAN | 40S ribosomal protein S13 | 0.81026 | 0.018233588 |
| P12236 | ADT3_HUMAN | ADP/ATP translocase 3 | 0.81138 | 0.010292484 |
| P35579 | MYH9_HUMAN | Myosin-9 | 0.81207 | -0.017944783 |
| Q9BTT0 | AN32E_HUMAN | Acidic leucine-rich nuclear phosphoprotein 32 family member E | 0.81279 | 0.014948483 |
| O43684 | BUB3_HUMAN | Mitotic checkpoint protein BUB3 | 0.81411 | -0.016248533 |
| Q9UNM6 | PSD13_HUMAN | 26S proteasome non-ATPase regulatory subunit 13 | 0.81412 | -0.027910027 |
| P27797 | CALR_HUMAN | Calreticulin | 0.81448 | -0.008737277 |
| O14980 | XPO1_HUMAN | Exportin-1 | 0.81621 | -0.023215493 |
| Q32P28-3 | P3H1_HUMAN | Isoform 3 of Prolyl 3-hydroxylase 1 | 0.81812 | -0.017223993 |
| Q9UQE7 | SMC3_HUMAN | Structural maintenance of chromosomes protein 3 | 0.81926 | 0.031682692 |
| P04406 | G3P_HUMAN | Glyceraldehyde-3-phosphate dehydrogenase | 0.82058 | 0.010216717 |
| Q9NNW7-2 | TRXR2_HUMAN | Isoform 2 of Thioredoxin reductase 2, mitochondrial | 0.82223 | -0.021034515 |
| P39656 | OST48_HUMAN | Dolichyl-diphosphooligosaccharide--protein glycosyltransferase 48 kDa subunit | 0.82272 | -0.007834076 |
| O94874 | UFL1_HUMAN | E3 UFM1-protein ligase 1 | 0.82286 | 0.025683513 |
| Q9Y490 | TLN1_HUMAN | Talin-1 | 0.82348 | 0.015786262 |
| P35580-4 | MYH10_HUMAN | Isoform 4 of Myosin-10 | 0.8243 | 0.061802088 |
| P13747 | HLAE_HUMAN | HLA class I histocompatibility antigen, alpha chain E | 0.82432 | 0.021242404 |
| P51148 | RAB5C_HUMAN | Ras-related protein Rab-5C | 0.82576 | 0.014729481 |
| P43487 | RANG_HUMAN | Ran-specific GTPase-activating protein | 0.82607 | 0.03528447 |
| Q04637-8 | IF4G1_HUMAN | Isoform 8 of Eukaryotic translation initiation factor 4 gamma 1 | 0.82794 | -0.022605839 |
| P11766 | ADHX_HUMAN | Alcohol dehydrogenase class-3 | 0.82866 | 0.060148291 |
| P31040 | SDHA_HUMAN | Succinate dehydrogenase [ubiquinone] flavoprotein subunit, mitochondrial | 0.82887 | 0.016149324 |
| O60610 | DIAP1_HUMAN | Protein diaphanous homolog 1 | 0.83048 | 0.010581216 |
| Q8N1P7 | CRBG2_HUMAN | Beta/gamma crystallin domain-containing protein 2 | 0.83053 | -0.040069506 |
| Q14019 | COTL1_HUMAN | Coactosin-like protein | 0.83077 | -0.027158186 |
| P04083 | ANXA1_HUMAN | Annexin A1 | 0.83241 | -0.014135349 |
| Q9NZ08 | ERAP1_HUMAN | Endoplasmic reticulum aminopeptidase 1 | 0.83251 | 0.015792505 |
| P31146 | COR1A_HUMAN | Coronin-1A | 0.83367 | 0.032819713 |
| P62191 | PRS4_HUMAN | 26S proteasome regulatory subunit 4 | 0.8337 | -0.020891384 |
| Q9Y584 | TIM22_HUMAN | Mitochondrial import inner membrane translocase subunit Tim22 | 0.83403 | -0.03879566 |
| P23528 | COF1_HUMAN | Cofilin-1 | 0.83458 | -0.020447851 |
| P13760 | 2B14_HUMAN | HLA class II histocompatibility antigen, DRB1-4 beta chain | 0.8346 | -0.056174946 |
| Q02790 | FKBP4_HUMAN | Peptidyl-prolyl cis-trans isomerase FKBP4 | 0.83518 | 0.007037305 |
| Q9NZZ3 | CHMP5_HUMAN | Charged multivesicular body protein 5 | 0.83519 | 0.024916873 |
| P15311 | EZRI_HUMAN | Ezrin | 0.83566 | -0.017450077 |
| P05556 | ITB1_HUMAN | Integrin beta-1 | 0.83679 | -0.007479182 |
| P09382 | LEG1_HUMAN | Galectin-1 | 0.8375 | -0.043940421 |
| P22314 | UBA1_HUMAN | Ubiquitin-like modifier-activating enzyme 1 | 0.83756 | 0.009222344 |
| Q9Y285 | SYFA_HUMAN | Phenylalanine--tRNA ligase alpha subunit | 0.84101 | 0.00586446 |
| O75489 | NDUS3_HUMAN | NADH dehydrogenase [ubiquinone] iron-sulfur protein 3, mitochondrial | 0.84195 | -0.011732722 |
| P34897 | GLYM_HUMAN | Serine hydroxymethyltransferase, mitochondrial | 0.84298 | 0.016958943 |
| Q9UHB9 | SRP68_HUMAN | Signal recognition particle subunit SRP68 | 0.84301 | 0.014446828 |
| P23284 | PPIB_HUMAN | Peptidyl-prolyl cis-trans isomerase B | 0.84306 | -0.014165508 |
| P07602 | SAP_HUMAN | Prosaposin | 0.8432 | -0.025066172 |
| Q13409 | DC1I2_HUMAN | Cytoplasmic dynein 1 intermediate chain 2 | 0.84459 | -0.033056479 |
| Q96NY8 | NECT4_HUMAN | Nectin-4 | 0.8447 | 0.027795132 |
| P10155 | RO60_HUMAN | 60 kDa SS-A/Ro ribonucleoprotein | 0.84522 | -0.01381352 |
| Q9UP95 | S12A4_HUMAN | Solute carrier family 12 member 4 | 0.84528 | 0.013976826 |
| O43169 | CYB5B_HUMAN | Cytochrome b5 type B | 0.84753 | -0.007783152 |
| Q9NZM1 | MYOF_HUMAN | Myoferlin | 0.84764 | 0.010838043 |
| Q9BV40 | VAMP8_HUMAN | Vesicle-associated membrane protein 8 | 0.84827 | -0.012549082 |
| Q9Y2P4 | S27A6_HUMAN | Long-chain fatty acid transport protein 6 | 0.84828 | 0.030008434 |
| P11279 | LAMP1_HUMAN | Lysosome-associated membrane glycoprotein 1 | 0.84948 | -0.009012347 |
| O75964 | ATP5L_HUMAN | ATP synthase subunit g, mitochondrial | 0.84948 | -0.016136928 |
| Q9H299 | SH3L3_HUMAN | SH3 domain-binding glutamic acid-rich-like protein 3 | 0.84951 | -0.053038588 |
| Q86TM6 | SYVN1_HUMAN | E3 ubiquitin-protein ligase synoviolin | 0.85245 | 0.008787881 |
| P19878 | NCF2_HUMAN | Neutrophil cytosol factor 2 | 0.85331 | 0.026584927 |
| P11387 | TOP1_HUMAN | DNA topoisomerase 1 | 0.85384 | -0.013700308 |
| P04843 | RPN1_HUMAN | Dolichyl-diphosphooligosaccharide--protein glycosyltransferase subunit 1 | 0.85429 | 0.003357902 |
| P05787 | K2C8_HUMAN | Keratin, type II cytoskeletal 8 | 0.85459 | -0.006332168 |
| P19338 | NUCL_HUMAN | Nucleolin | 0.8547 | 0.011580461 |
| O75223 | GGCT_HUMAN | Gamma-glutamylcyclotransferase | 0.85526 | -0.017527582 |
| P28070 | PSB4_HUMAN | Proteasome subunit beta type-4 | 0.8557 | -0.012385318 |
| P48147 | PPCE_HUMAN | Prolyl endopeptidase | 0.85585 | 0.016766979 |
| Q6NUK1 | SCMC1_HUMAN | Calcium-binding mitochondrial carrier protein SCaMC-1 | 0.856 | 0.016634854 |
| P78527 | PRKDC_HUMAN | DNA-dependent protein kinase catalytic subunit | 0.8562 | 0.023323202 |
| P04792 | HSPB1_HUMAN | Heat shock protein beta-1 | 0.85651 | 0.018958595 |
| Q9NR45 | SIAS_HUMAN | Sialic acid synthase | 0.85777 | -0.010938272 |
| Q9P0L0 | VAPA_HUMAN | Vesicle-associated membrane protein-associated protein A | 0.85803 | 0.013439987 |
| P09622 | DLDH_HUMAN | Dihydrolipoyl dehydrogenase, mitochondrial | 0.85984 | -0.006361758 |
| P23142 | FBLN1_HUMAN | Fibulin-1 | 0.86001 | -0.007980623 |
| Q4VCS5 | AMOT_HUMAN | Angiomotin | 0.86013 | 0.023246522 |
| O14735 | CDIPT_HUMAN | CDP-diacylglycerol--inositol 3-phosphatidyltransferase | 0.8612 | 0.007499249 |
| P48556 | PSMD8_HUMAN | 26S proteasome non-ATPase regulatory subunit 8 | 0.86154 | -0.010950654 |
| P07237 | PDIA1_HUMAN | Protein disulfide-isomerase | 0.8621 | -0.004853323 |
| Q13200 | PSMD2_HUMAN | 26S proteasome non-ATPase regulatory subunit 2 | 0.86374 | 0.007933854 |
| Q9NRP0 | OSTC_HUMAN | Oligosaccharyltransferase complex subunit | 0.86407 | -0.006454803 |
| Q07075 | AMPE_HUMAN | Glutamyl aminopeptidase | 0.8644 | 0.01658874 |
| P08243 | ASNS_HUMAN | Asparagine synthetase [glutamine-hydrolyzing] | 0.86468 | -0.01284311 |
| P55786 | PSA_HUMAN | Puromycin-sensitive aminopeptidase | 0.86527 | 0.007333729 |
| Q6NZI2 | CAVN1_HUMAN | Caveolae-associated protein 1 | 0.86554 | -0.014835743 |
| Q92945 | FUBP2_HUMAN | Far upstream element-binding protein 2 | 0.86576 | 0.013148133 |
| O94826 | TOM70_HUMAN | Mitochondrial import receptor subunit TOM70 | 0.86585 | 0.008884099 |
| Q15758 | AAAT_HUMAN | Neutral amino acid transporter B(0) | 0.86607 | 0.020739205 |
| Q07812-5 | BAX_HUMAN | Isoform Epsilon of Apoptosis regulator BAX | 0.86667 | -0.00788234 |
| P35241 | RADI_HUMAN | Radixin | 0.86818 | 0.012065453 |
| O75352 | MPU1_HUMAN | Mannose-P-dolichol utilization defect 1 protein | 0.86847 | -0.021462227 |
| P84095 | RHOG_HUMAN | Rho-related GTP-binding protein RhoG | 0.86857 | -0.042010963 |
| Q8NBS9 | TXND5_HUMAN | Thioredoxin domain-containing protein 5 | 0.8687 | -0.021452909 |
| P53041 | PPP5_HUMAN | Serine/threonine-protein phosphatase 5 | 0.86875 | 0.033487099 |
| P17980 | PRS6A_HUMAN | 26S proteasome regulatory subunit 6A | 0.86894 | -0.017862979 |
| Q96AC1 | FERM2_HUMAN | Fermitin family homolog 2 | 0.86898 | 0.010310744 |
| Q99959 | PKP2_HUMAN | Plakophilin-2 | 0.86987 | -0.020941138 |
| Q96QK1 | VPS35_HUMAN | Vacuolar protein sorting-associated protein 35 | 0.86992 | 0.008239723 |
| Q15393 | SF3B3_HUMAN | Splicing factor 3B subunit 3 | 0.87034 | 0.012634295 |
| P05141 | ADT2_HUMAN | ADP/ATP translocase 2 | 0.87081 | -0.00692496 |
| P50570 | DYN2_HUMAN | Dynamin-2 | 0.87205 | 0.006236535 |
| Q9BZZ5 | API5_HUMAN | Apoptosis inhibitor 5 | 0.8721 | -0.007814292 |
| P61158 | ARP3_HUMAN | Actin-related protein 3 | 0.87279 | 0.006229121 |
| Q32MZ4 | LRRF1_HUMAN | Leucine-rich repeat flightless-interacting protein 1 | 0.87299 | 0.015575519 |
| Q9NW15 | ANO10_HUMAN | Anoctamin-10 | 0.87372 | -0.025764461 |
| P05166 | PCCB_HUMAN | Propionyl-CoA carboxylase beta chain, mitochondrial | 0.87453 | 0.028990767 |
| P39019 | RS19_HUMAN | 40S ribosomal protein S19 | 0.87489 | -0.010753753 |
| P07942 | LAMB1_HUMAN | Laminin subunit beta-1 | 0.87572 | -0.087985946 |
| P83105 | HTRA4_HUMAN | Serine protease HTRA4 | 0.87614 | 0.016196315 |
| O00151 | PDLI1_HUMAN | PDZ and LIM domain protein 1 | 0.87673 | -0.029694859 |
| P55957 | BID_HUMAN | BH3-interacting domain death agonist | 0.87697 | 0.018201466 |
| Q9Y3Z3 | SAMH1_HUMAN | Deoxynucleoside triphosphate triphosphohydrolase SAMHD1 | 0.87746 | 0.021725549 |
| Q9BQE3 | TBA1C_HUMAN | Tubulin alpha-1C chain | 0.87868 | 0.012220315 |
| P28072 | PSB6_HUMAN | Proteasome subunit beta type-6 | 0.87918 | -0.010375408 |
| Q9BSJ8 | ESYT1_HUMAN | Extended synaptotagmin-1 | 0.88202 | -0.010710902 |
| Q07666 | KHDR1_HUMAN | KH domain-containing, RNA-binding, signal transduction-associated protein 1 | 0.88202 | -0.01109592 |
| P35527 | K1C9_HUMAN | Keratin, type I cytoskeletal 9 | 0.88257 | 0.008781996 |
| P50991 | TCPD_HUMAN | T-complex protein 1 subunit delta | 0.88357 | -0.01564514 |
| Q13148 | TADBP_HUMAN | TAR DNA-binding protein 43 | 0.88375 | 0.012585955 |
| P05362 | ICAM1_HUMAN | Intercellular adhesion molecule 1 | 0.88417 | -0.022469149 |
| O43175 | SERA_HUMAN | D-3-phosphoglycerate dehydrogenase | 0.88513 | -0.012850734 |
| Q03519 | TAP2_HUMAN | Antigen peptide transporter 2 | 0.88543 | 0.010501368 |
| P08670 | VIME_HUMAN | Vimentin | 0.88641 | 0.016304179 |
| P07737 | PROF1_HUMAN | Profilin-1 | 0.88715 | -0.008676982 |
| P41252 | SYIC_HUMAN | Isoleucine--tRNA ligase, cytoplasmic | 0.88796 | 0.007647483 |
| P50895 | BCAM_HUMAN | Basal cell adhesion molecule | 0.89215 | 0.015902423 |
| P01903 | DRA_HUMAN | HLA class II histocompatibility antigen, DR alpha chain | 0.8927 | -0.027254116 |
| P40227 | TCPZ_HUMAN | T-complex protein 1 subunit zeta | 0.89286 | 0.003798309 |
| Q13724 | MOGS_HUMAN | Mannosyl-oligosaccharide glucosidase | 0.89423 | 0.020098087 |
| P43243 | MATR3_HUMAN | Matrin-3 | 0.89479 | 0.022168348 |
| Q9UJZ1 | STML2_HUMAN | Stomatin-like protein 2, mitochondrial | 0.89498 | 0.007500733 |
| Q13011 | ECH1_HUMAN | Delta(3,5)-Delta(2,4)-dienoyl-CoA isomerase, mitochondrial | 0.89635 | -0.012596499 |
| Q06203 | PUR1_HUMAN | Amidophosphoribosyltransferase | 0.8974 | -0.011753576 |
| P21291 | CSRP1_HUMAN | Cysteine and glycine-rich protein 1 | 0.89745 | 0.005550046 |
| Q9UNL2 | SSRG_HUMAN | Translocon-associated protein subunit gamma | 0.89872 | -0.004401338 |
| P14061 | DHB1_HUMAN | Estradiol 17-beta-dehydrogenase 1 | 0.89908 | -0.011475937 |
| P30622 | CLIP1_HUMAN | CAP-Gly domain-containing linker protein 1 | 0.90009 | 0.012639727 |
| Q13188 | STK3_HUMAN | Serine/threonine-protein kinase 3 | 0.90144 | -0.003526053 |
| O15145 | ARPC3_HUMAN | Actin-related protein 2/3 complex subunit 3 | 0.90156 | 0.011006192 |
| Q03135 | CAV1_HUMAN | Caveolin-1 | 0.90269 | -0.006582596 |
| P27816-6 | MAP4_HUMAN | Isoform 6 of Microtubule-associated protein 4 | 0.90293 | -0.005624585 |
| O60701 | UGDH_HUMAN | UDP-glucose 6-dehydrogenase | 0.90318 | -0.009130167 |
| Q9H4M9 | EHD1_HUMAN | EH domain-containing protein 1 | 0.90408 | 0.005965049 |
| P00558 | PGK1_HUMAN | Phosphoglycerate kinase 1 | 0.90474 | -0.005956294 |
| Q9P246-2 | STIM2_HUMAN | Isoform 2 of Stromal interaction molecule 2 | 0.90519 | -0.013183309 |
| P22059 | OSBP1_HUMAN | Oxysterol- binding protein 1 | 0.90536 | 0.004789464 |
| Q12797 | ASPH_HUMAN | Aspartyl/asparaginyl beta-hydroxylase | 0.90562 | 0.003867315 |
| Q8NC56 | LEMD2_HUMAN | LEM domain-containing protein 2 | 0.90573 | 0.004559847 |
| O60763 | USO1_HUMAN | General vesicular transport factor p115 | 0.90633 | 0.011214634 |
| P42765 | THIM_HUMAN | 3-ketoacyl-CoA thiolase, mitochondrial | 0.90754 | 0.010175551 |
| Q16555 | DPYL2_HUMAN | Dihydropyrimidinase-related protein 2 | 0.90793 | 0.007199911 |
| P60174 | TPIS_HUMAN | Triosephosphate isomerase | 0.9082 | -0.005044634 |
| P09525 | ANXA4_HUMAN | Annexin A4 | 0.91005 | -0.008115588 |
| P05023 | AT1A1_HUMAN | Sodium/potassium-transporting ATPase subunit alpha-1 | 0.91146 | 0.005472309 |
| P00533 | EGFR_HUMAN | Epidermal growth factor receptor | 0.91173 | 0.004445051 |
| Q06210 | GFPT1_HUMAN | Glutamine--fructose-6-phosphate aminotransferase [isomerizing] 1 | 0.91195 | 0.006607309 |
| O14949 | QCR8_HUMAN | Cytochrome b-c1 complex subunit 8 | 0.9127 | 0.007430943 |
| P30101 | PDIA3_HUMAN | Protein disulfide-isomerase A3 | 0.9159 | 0.003830534 |
| P62826 | RAN_HUMAN | GTP-binding nuclear protein Ran | 0.91605 | 0.004315888 |
| P23588 | IF4B_HUMAN | Eukaryotic translation initiation factor 4B | 0.91619 | -0.036802194 |
| Q9ULV4 | COR1C_HUMAN | Coronin-1C | 0.91696 | 0.005252798 |
| Q9Y4E8 | UBP15_HUMAN | Ubiquitin carboxyl-terminal hydrolase 15 | 0.9176 | -0.004238017 |
| P47897 | SYQ_HUMAN | Glutamine--tRNA ligase | 0.91858 | -0.009444284 |
| P53007 | TXTP_HUMAN | Tricarboxylate transport protein, mitochondrial | 0.9199 | -0.012188658 |
| P21283 | VATC1_HUMAN | V-type proton ATPase subunit C 1 | 0.92 | -0.010413362 |
| P63000 | RAC1_HUMAN | Ras-related C3 botulinum toxin substrate 1 | 0.92039 | 0.004972237 |
| Q99805 | TM9S2_HUMAN | Transmembrane 9 superfamily member 2 | 0.92059 | -0.008303976 |
| P29466 | CASP1_HUMAN | Caspase-1 | 0.92077 | -0.014047062 |
| P14866 | HNRPL_HUMAN | Heterogeneous nuclear ribonucleoprotein L | 0.92124 | 0.014806094 |
| O14684 | PTGES_HUMAN | Prostaglandin E synthase | 0.92152 | -0.004976535 |
| P31939 | PUR9_HUMAN | Bifunctional purine biosynthesis protein PURH | 0.92159 | -0.005064387 |
| O95292 | VAPB_HUMAN | Vesicle-associated membrane protein-associated protein B/C | 0.92438 | -0.003136717 |
| Q15075 | EEA1_HUMAN | Early endosome antigen 1 | 0.92464 | -0.017703162 |
| P16152 | CBR1_HUMAN | Carbonyl reductase [NADPH] 1 | 0.92551 | -0.00840568 |
| P30566 | PUR8_HUMAN | Adenylosuccinate lyase | 0.92568 | 0.011031067 |
| P26038 | MOES_HUMAN | Moesin | 0.92618 | 0.012339174 |
| Q92688 | AN32B_HUMAN | Acidic leucine-rich nuclear phosphoprotein 32 family member B | 0.92768 | 0.004805288 |
| P84077 | ARF1_HUMAN | ADP-ribosylation factor 1 | 0.92914 | 0.010551644 |
| Q9C002 | NMES1_HUMAN | Normal mucosa of esophagus-specific gene 1 protein | 0.92968 | -0.014134222 |
| P46782 | RS5_HUMAN | 40S ribosomal protein S5 | 0.93029 | 0.007235629 |
| P15144 | AMPN_HUMAN | Aminopeptidase N | 0.93041 | -0.023499711 |
| P08842 | STS_HUMAN | Steryl-sulfatase | 0.93095 | 0.004063591 |
| P41250 | GARS_HUMAN | Glycine--tRNA ligase | 0.93134 | -0.002803011 |
| O95379 | TFIP8_HUMAN | Tumor necrosis factor alpha-induced protein 8 | 0.93384 | 0.003455622 |
| P30086 | PEBP1_HUMAN | Phosphatidylethanolamine-binding protein 1 | 0.93672 | 0.009455486 |
| Q8IY17 | PLPL6_HUMAN | Neuropathy target esterase | 0.93673 | -0.005378748 |
| P06702 | S10A9_HUMAN | Protein S100-A9 | 0.93786 | -0.012982994 |
| Q9NTX5 | ECHD1_HUMAN | Ethylmalonyl-CoA decarboxylase | 0.93821 | 0.025504079 |
| P34932 | HSP74_HUMAN | Heat shock 70 kDa protein 4 | 0.93858 | -0.001498733 |
| P29401 | TKT_HUMAN | Transketolase | 0.93903 | 0.003204551 |
| O43488 | ARK72_HUMAN | Aflatoxin B1 aldehyde reductase member 2 | 0.93956 | -0.005493522 |
| Q15836 | VAMP3_HUMAN | Vesicle-associated membrane protein 3 | 0.93986 | -0.003759582 |
| P04080 | CYTB_HUMAN | Cystatin-B | 0.94047 | -0.009457504 |
| P10253 | LYAG_HUMAN | Lysosomal alpha-glucosidase | 0.9406 | -0.011503072 |
| Q92599-2 | SEPT8_HUMAN | Isoform 2 of Septin-8 | 0.94116 | -0.004382767 |
| P61163 | ACTZ_HUMAN | Alpha-centractin | 0.94141 | 0.007288573 |
| Q8TD55 | PKHO2_HUMAN | Pleckstrin homology domain-containing family | 0.94247 | 0.007603378 |
| Q99436 | PSB7_HUMAN | Proteasome subunit beta type-7 | 0.94253 | 0.004970752 |
| P07954 | FUMH_HUMAN | Fumarate hydratase, mitochondrial | 0.94285 | 0.004288427 |
| Q16527 | CSRP2_HUMAN | Cysteine and glycine-rich protein 2 | 0.94294 | 0.008060516 |
| P50552 | VASP_HUMAN | Vasodilator-stimulated phosphoprotein | 0.94535 | -0.007383311 |
| P40925 | MDHC_HUMAN | Malate dehydrogenase, cytoplasmic | 0.94636 | -0.001560839 |
| Q8WWP7 | GIMA1_HUMAN | GTPase IMAP family member 1 | 0.9474 | 0.012123079 |
| P02533 | K1C14_HUMAN | Keratin, type I cytoskeletal 14 | 0.94851 | -0.00567823 |
| P19971 | TYPH_HUMAN | Thymidine phosphorylase | 0.94997 | 0.008615379 |
| P36543 | VATE1_HUMAN | V-type proton ATPase subunit E 1 | 0.95022 | 0.00587729 |
| Q92734 | TFG_HUMAN | Protein TFG | 0.95089 | 0.005185632 |
| Q9Y2Q3 | GSTK1_HUMAN | Glutathione S-transferase kappa 1 | 0.95168 | 0.00372612 |
| Q8TAT6 | NPL4_HUMAN | Nuclear protein localization protein 4 homolog | 0.952 | -0.002175862 |
| P63208 | SKP1_HUMAN | S-phase kinase-associated protein 1 | 0.95266 | 0.009181763 |
| P19105 | ML12A_HUMAN | Myosin regulatory light chain 12A | 0.95387 | -0.005739921 |
| O94973 | AP2A2_HUMAN | AP-2 complex subunit alpha-2 | 0.95688 | 0.002235003 |
| Q9UGI8-2 | TES_HUMAN | Isoform 2 of Testin | 0.95839 | -0.002001304 |
| P98164 | LRP2_HUMAN | Low-density lipoprotein receptor-related protein 2 | 0.9584 | 0.006559029 |
| P05091 | ALDH2_HUMAN | Aldehyde dehydrogenase, mitochondrial | 0.95843 | -0.011128074 |
| P04004 | VTNC_HUMAN | Vitronectin | 0.95895 | -0.003640749 |
| Q9NZ45 | CISD1_HUMAN | CDGSH iron-sulfur domain-containing protein 1 | 0.9592 | -0.003062106 |
| P62304 | RUXE_HUMAN | Small nuclear ribonucleoprotein E | 0.95957 | -0.006251987 |
| P14317 | HCLS1_HUMAN | Hematopoietic lineage cell-specific protein | 0.95966 | 0.005119508 |
| Q0P6H9 | TMM62_HUMAN | Transmembrane protein 62 | 0.95989 | -0.009814475 |
| O00410 | IPO5_HUMAN | Importin-5 | 0.96048 | -0.004950763 |
| Q9NYL9 | TMOD3_HUMAN | Tropomodulin-3 | 0.9617 | 0.004320358 |
| P31949 | S10AB_HUMAN | Protein S100-A11 | 0.96191 | -0.00746803 |
| Q15165 | PON2_HUMAN | Serum paraoxonase/arylesterase 2 | 0.96196 | 0.005204257 |
| P55795 | HNRH2_HUMAN | Heterogeneous nuclear ribonucleoprotein H2 | 0.96406 | 0.004262491 |
| Q13492 | PICAL_HUMAN | Phosphatidylinositol-binding clathrin assembly protein | 0.96434 | -0.005686029 |
| Q99613 | EIF3C_HUMAN | Eukaryotic translation initiation factor 3 subunit C | 0.96457 | -0.000984216 |
| P14625 | ENPL_HUMAN | Endoplasmin | 0.96511 | 0.001691119 |
| P30044 | PRDX5_HUMAN | Peroxiredoxin-5, mitochondrial | 0.96602 | -0.00239309 |
| O14579 | COPE_HUMAN | Coatomer subunit epsilon | 0.96753 | -0.002736242 |
| O75369 | FLNB_HUMAN | Filamin-B | 0.96805 | 0.001369705 |
| Q08170 | SRSF4_HUMAN | Serine/arginine-rich splicing factor 4 | 0.96854 | -0.001771361 |
| Q15286 | RAB35_HUMAN | Ras-related protein Rab-35 | 0.96888 | -0.005416167 |
| P21397 | AOFA_HUMAN | Amine oxidase [flavin-containing] A | 0.96954 | -0.004016449 |
| P05114 | HMGN1_HUMAN | Non-histone chromosomal protein HMG-14 | 0.97144 | 0.011186222 |
| Q9UL46 | PSME2_HUMAN | Proteasome activator complex subunit 2 | 0.97401 | 0.003063607 |
| P48643 | TCPE_HUMAN | T-complex protein 1 subunit epsilon | 0.9741 | 0.004360317 |
| E9PAV3 | NACAM_HUMAN | Nascent polypeptide-associated complex subunit alpha, muscle-specific form | 0.97443 | -0.001213399 |
| P23634 | AT2B4_HUMAN | Plasma membrane calcium-transporting ATPase 4 | 0.97579 | -0.002219845 |
| O75821 | EIF3G_HUMAN | Eukaryotic translation initiation factor 3 subunit G | 0.9763 | 0.001071688 |
| Q04446 | GLGB_HUMAN | 1,4-alpha-glucan-branching enzyme | 0.97708 | -0.004766019 |
| Q13045 | FLII_HUMAN | Protein flightless-1 homolog | 0.9771 | -0.001314043 |
| Q9Y365 | STA10_HUMAN | START domain-containing protein 10 | 0.97786 | -0.002780165 |
| P12429 | ANXA3_HUMAN | Annexin A3 | 0.97802 | -0.00238778 |
| Q01650 | LAT1_HUMAN | Large neutral amino acids transporter small subunit 1 | 0.97854 | -0.00286682 |
| P43307 | SSRA_HUMAN | Translocon-associated protein subunit alpha | 0.97911 | 0.00115559 |
| Q9BWM7 | SFXN3_HUMAN | Sideroflexin-3 | 0.9803 | -0.002246107 |
| Q9H4A3-7 | WNK1_HUMAN | Isoform 6 of Serine/threonine-protein kinase WNK1 | 0.98043 | 0.004204696 |
| Q7Z2K6 | ERMP1_HUMAN | Endoplasmic reticulum metallopeptidase 1 | 0.98129 | 0.001844024 |
| P05783 | K1C18_HUMAN | Keratin, type I cytoskeletal 18 | 0.98288 | -0.000875715 |
| Q96HE7 | ERO1A_HUMAN | ERO1- likeprotein alpha | 0.98332 | -0.001187462 |
| P05164 | PERM_HUMAN | Myeloperoxidase | 0.98365 | 0.005734268 |
| P24539 | AT5F1_HUMAN | ATP synthase F(0) complex subunit B1, mitochondrial | 0.98387 | 0.000865718 |
| P05455 | LA_HUMAN | Lupus La protein | 0.98399 | 0.003127708 |
| O95336 | 6PGL_HUMAN | 6-phosphogluconolactonase | 0.98401 | -0.001184908 |
| O95817 | BAG3_HUMAN | BAG family molecular chaperone regulator 3 | 0.98413 | 0.001896925 |
| P09601 | HMOX1_HUMAN | Heme oxygenase 1 | 0.98436 | -0.001477225 |
| P68402 | PA1B2_HUMAN | Platelet-activating factor acetylhydrolase IB subunit beta | 0.98571 | -0.001616119 |
| P52565 | GDIR1_HUMAN | Rho GDP-dissociation inhibitor 1 | 0.98611 | -0.001030368 |
| Q14165 | MLEC_HUMAN | Malectin | 0.98778 | 0.002794255 |
| Q9H8S9 | MOB1A_HUMAN | MOB kinase activator 1A | 0.98809 | 0.001825758 |
| Q05682-5 | CALD1_HUMAN | Isoform 5 of Caldesmon | 0.98945 | 0.001352893 |
| P61803 | DAD1_HUMAN | Dolichyl-diphosphooligosaccharide--protein glycosyltransferase subunit DAD1 | 0.98966 | -0.00114233 |
| P10599 | THIO_HUMAN | Thioredoxin | 0.99063 | -0.00201965 |
| P61088 | UBE2N_HUMAN | Ubiquitin-conjugating enzyme E2 N | 0.99118 | 0.001046403 |
| Q99459 | CDC5L_HUMAN | Cell division cycle 5-like protein | 0.99118 | 0.000665705 |
| Q08722 | CD47_HUMAN | Leukocyte surface antigen CD47 | 0.99154 | -0.001449629 |
| P60981 | DEST_HUMAN | Destrin | 0.99175 | 0.001434974 |
| A6NCS6 | CB072_HUMAN | Uncharacterized protein C2orf72 | 0.99227 | 0.001279121 |
| Q9H3N1 | TMX1_HUMAN | Thioredoxin-related transmembrane protein 1 | 0.99261 | -0.000978258 |
| P12532 | KCRU_HUMAN | Creatine kinase U-type, mitochondrial | 0.99274 | -0.002137745 |
| P60660 | MYL6_HUMAN | Myosin light polypeptide 6 | 0.99284 | -0.00126597 |
| P35221 | CTNA1_HUMAN | Catenin alpha-1 | 0.9945 | -0.000477467 |
| Q03252 | LMNB2_HUMAN | Lamin-B2 | 0.99458 | -0.000304412 |
| P48735 | IDHP_HUMAN | Isocitrate dehydrogenase [NADP], mitochondrial | 0.9947 | 0.000580113 |
| P08727 | K1C19_HUMAN | Keratin, type I cytoskeletal 19 | 0.99473 | -0.000360121 |
| Q16658 | FSCN1_HUMAN | Fascin | 0.99623 | 0.0006135 |
| P62318 | SMD3_HUMAN | Small nuclear ribonucleoprotein Sm D3 | 0.99644 | -0.000233159 |
| P06576 | ATPB_HUMAN | ATP synthase subunit beta, mitochondrial | 0.99654 | 0.000162207 |
| P21589 | 5NTD_HUMAN | 5'-nucleotidase | 0.99664 | -0.001008175 |
| Q9BVC6 | TM109_HUMAN | Transmembrane protein 109 | 0.99751 | 0.000108645 |
