# Supplementary material for: Extracellular vesicle-associated miR-515-5p from adipose tissue regulates placental metabolism and fetal growth in gestational diabetes mellitus
Source: Cardiovasc Diabetol. 2025 May 14;24:205. doi: 10.1186/s12933-025-02739-z (PMC12080180; doi:10.1186/s12933-025-02739-z)
Supplement: Supplementary file 11 — Supplementary Material 11 [file 12933_2025_2739_MOESM11_ESM.docx]

**Supplementary Table 10: Table below shows the pathway regulated by miR-516-5p in the placental cells identified using Gene Ontology analysis**

| **Protein ID** | **Mapped ID** | **Group** | **p-value** | **Log (Fold Change)** |
| --- | --- | --- | --- | --- |
| P05161 | ISG15_HUMAN | Ubiquitin-like protein ISG15 | 3.98E-05 | -0.57078 |
| O95786 | DDX58_HUMAN | Probable ATP-dependent RNA helicase DDX58 | 0.00018 | -0.60194 |
| P09914 | IFIT1_HUMAN | Interferon-induced protein with tetratricopeptide repeats 1 | 0.0002 | -0.45251 |
| P08865 | RSSA_HUMAN | 40S ribosomal protein SA | 0.00041 | 0.09437 |
| P62851 | RS25_HUMAN | 40S ribosomal protein S25 | 0.00062 | 0.08880 |
| P09913 | IFIT2_HUMAN | Interferon-induced protein with tetratricopeptide repeats 2 | 0.00117 | -0.45305 |
| P61247 | RS3A_HUMAN | 40S ribosomal protein S3a | 0.00178 | 0.09852 |
| P35268 | RL22_HUMAN | 60S ribosomal protein L22 | 0.00277 | 0.11951 |
| P18621-3 | RL17_HUMAN | Isoform 3 of 60S ribosomal protein L17 | 0.00294 | 0.09788 |
| P30050 | RL12_HUMAN | 60S ribosomal protein L12 | 0.00319 | 0.07993 |
| P50914 | RL14_HUMAN | 60S ribosomal protein L14 | 0.00323 | 0.11303 |
| O15162 | PLS1_HUMAN | Phospholipid scramblase 1 | 0.00367 | -0.26123 |
| O14879 | IFIT3_HUMAN | Interferon-induced protein with tetratricopeptide repeats 3 | 0.00409 | -0.51672 |
| Q9Y4L1 | HYOU1_HUMAN | Hypoxia up-regulated protein 1 | 0.00419 | 0.06579 |
| Q02543 | RL18A_HUMAN | 60S ribosomal protein L18a | 0.00449 | 0.14909 |
| O60841 | IF2P_HUMAN | Eukaryotic translation initiation factor 5B | 0.00507 | 0.39297 |
| P26196 | DDX6_HUMAN | Probable ATP-dependent RNA helicase DDX6 | 0.00538 | 0.06451 |
| P08648 | ITA5_HUMAN | Integrin alpha-5 | 0.00576 | -0.09842 |
| O75396 | SC22B_HUMAN | Vesicle-trafficking protein SEC22b | 0.00583 | 0.10167 |
| Q16531 | DDB1_HUMAN | DNA damage-binding protein 1 | 0.00617 | 0.27919 |
| P05388 | RLA0_HUMAN | 60S acidic ribosomal protein P0 | 0.00647 | 0.10976 |
| P26373 | RL13_HUMAN | 60S ribosomal protein L13 | 0.00702 | 0.10492 |
| P20591 | MX1_HUMAN | Interferon-induced GTP-binding protein Mx1 | 0.00708 | -0.42453 |
| P35613-2 | BASI_HUMAN | Isoform 2 of Basigin | 0.0071 | -0.05293 |
| P62263 | RS14_HUMAN | 40S ribosomal protein S14 | 0.00971 | 0.30662 |
| O14617 | AP3D1_HUMAN | AP-3 complex subunit delta-1 | 0.0103 | 0.21184 |
| Q53EL6 | PDCD4_HUMAN | Programmed cell death protein 4 | 0.01056 | 0.15606 |
| P06744-2 | G6PI_HUMAN | Isoform 2 of Glucose-6-phosphate isomerase | 0.01409 | 0.13874 |
| P62314 | SMD1_HUMAN | Small nuclear ribonucleoprotein Sm D1 | 0.01415 | 0.05984 |
| Q9BXP5 | SRRT_HUMAN | Serrate RNA effector molecule homolog | 0.01418 | -0.12647 |
| Q07020 | RL18_HUMAN | 60S ribosomal protein L18 | 0.01456 | 0.10525 |
| P23396 | RS3_HUMAN | 40S ribosomal protein S3 | 0.01507 | 0.09502 |
| A0AV96 | RBM47_HUMAN | RNA-binding protein 47 | 0.01547 | 0.11105 |
| Q13418 | ILK_HUMAN | Integrin-linked protein kinase | 0.01549 | 0.07945 |
| P62249 | RS16_HUMAN | 40S ribosomal protein S16 | 0.01614 | 0.10740 |
| Q96FW1 | OTUB1_HUMAN | Ubiquitin thioesterase | 0.01691 | 0.19006 |
| Q15833 | STXB2_HUMAN | Syntaxin-binding protein 2 | 0.01742 | 0.17752 |
| Q13596 | SNX1_HUMAN | Sorting nexin-1 | 0.01772 | 0.07273 |
| O60610 | DIAP1_HUMAN | Protein diaphanous homolog 1 | 0.01833 | 0.11297 |
| Q00013 | EM55_HUMAN | 55 kDa erythrocyte membrane protein | 0.01923 | 0.17127 |
| Q9UKV3-5 | ACINU_HUMAN | Isoform 4 of Apoptotic chromatin condensation inducer in the nucleus | 0.01956 | 0.14419 |
| P50402 | EMD_HUMAN | Emerin | 0.02065 | 0.36618 |
| Q9UBW5 | BIN2_HUMAN | Bridging integrator 2 | 0.02081 | -0.41870 |
| P61421 | VA0D1_HUMAN | V-type proton ATPase subunit d 1 | 0.0217 | 0.12218 |
| O60506 | HNRPQ_HUMAN | Heterogeneous nuclear ribonucleoprotein Q | 0.0225 | 0.07180 |
| P62888 | RL30_HUMAN | 60S ribosomal protein L30 | 0.02262 | 0.07534 |
| P84098 | RL19_HUMAN | 60S ribosomal protein L19 | 0.02277 | 0.09451 |
| P19525 | E2AK2_HUMAN | Interferon-induced, double-stranded RNA-activated protein kinase | 0.02399 | -0.15516 |
| P62829 | RL23_HUMAN | 60S ribosomal protein L23 | 0.02411 | 0.10006 |
| P13693 | TCTP_HUMAN | Translationally-controlled tumor protein | 0.02428 | 0.14489 |
| Q9NX14 | NDUBB_HUMAN | NADH dehydrogenase [ubiquinone] 1 beta subcomplex subunit 11, mitochondrial | 0.02458 | -0.11178 |
| Q9NZ01 | TECR_HUMAN | Very-long-chain enoyl-CoA reductase | 0.02579 | 0.15601 |
| Q15257 | PTPA_HUMAN | Serine/threonine-protein phosphatase 2A activator | 0.02591 | -0.30634 |
| P07339 | CATD_HUMAN | Cathepsin D | 0.0261 | 0.23151 |
| O75438 | NDUB1_HUMAN | NADH dehydrogenase [ubiquinone] 1 beta subcomplex subunit 1 | 0.02633 | -0.53075 |
| Q92520 | FAM3C_HUMAN | Protein FAM3C | 0.02639 | -0.11701 |
| Q13435 | SF3B2_HUMAN | Splicing factor 3B subunit 2 | 0.02689 | 0.09175 |
| Q13838-2 | DX39B_HUMAN | Isoform 2 of Spliceosome RNA helicase DDX39B | 0.02735 | 0.05074 |
| P98082 | DAB2_HUMAN | Disabled homolog 2 | 0.02798 | 0.23289 |
| P49411 | EFTU_HUMAN | Elongation factor Tu, mitochondrial | 0.03 | 0.06103 |
| Q14019 | COTL1_HUMAN | Coactosin-like protein | 0.03012 | 0.65826 |
| P29728 | OAS2_HUMAN | 2'-5'-oligoadenylate synthase 2 | 0.03114 | -0.26252 |
| P32969 | RL9_HUMAN | 60S ribosomal protein L9 | 0.03128 | 0.14235 |
| Q14204 | DYHC1_HUMAN | Cytoplasmic dynein 1 heavy chain 1 | 0.03138 | 0.12590 |
| P62081 | RS7_HUMAN | 40S ribosomal protein S7 | 0.03188 | 0.10459 |
| P42766 | RL35_HUMAN | 60S ribosomal protein L35 | 0.03233 | 0.09596 |
| Q9GZT6 | CC90B_HUMAN | Coiled-coil domain-containing protein 90B, mitochondrial | 0.03234 | -0.09877 |
| P61923 | COPZ1_HUMAN | Coatomer subunit zeta-1 | 0.03256 | 0.07856 |
| P62917 | RL8_HUMAN | 60S ribosomal protein L8 | 0.03267 | 0.08531 |
| O00571 | DDX3X_HUMAN | ATP-dependent RNA helicase DDX3X | 0.03506 | 0.09498 |
| P40429 | RL13A_HUMAN | 60S ribosomal protein L13a | 0.03568 | 0.11374 |
| Q15005 | SPCS2_HUMAN | Signal peptidase complex subunit 2 | 0.03614 | -0.18268 |
| P46781 | RS9_HUMAN | 40S ribosomal protein S9 | 0.03717 | 0.09877 |
| P63167 | DYL1_HUMAN | Dynein light chain 1, cytoplasmic | 0.03983 | 0.05760 |
| P61803 | DAD1_HUMAN | Dolichyl-diphosphooligosaccharide--protein glycosyltransferase subunit DAD1 | 0.04004 | -0.12493 |
| P49755 | TMEDA_HUMAN | Transmembrane emp24 domain-containing protein 10 | 0.04195 | -0.09563 |
| P62913 | RL11_HUMAN | 60S ribosomal protein L11 | 0.04232 | 0.08334 |
| P42677 | RS27_HUMAN | 40S ribosomal protein S27 | 0.04281 | 0.16609 |
| Q99832 | TCPH_HUMAN | T-complex protein 1 subunit eta | 0.04326 | 0.06187 |
| P08708 | RS17_HUMAN | 40S ribosomal protein S17 | 0.04379 | 0.12967 |
| Q12931 | TRAP1_HUMAN | Heat shock protein 75 kDa, mitochondrial | 0.04399 | 0.06835 |
| P23193 | TCEA1_HUMAN | Transcription elongation factor A protein 1 | 0.04436 | 0.10476 |
| Q9HD45 | TM9S3_HUMAN | Transmembrane 9 superfamily member 3 | 0.04501 | -0.16631 |
| P13639 | EF2_HUMAN | Elongation factor 2 | 0.04526 | 0.07049 |
| P62269 | RS18_HUMAN | 40S ribosomal protein S18 | 0.04585 | 0.06769 |
| Q12905 | ILF2_HUMAN | Interleukin enhancer-binding factor 2 | 0.04594 | 0.15164 |
| P35659 | DEK_HUMAN | Protein DEK | 0.04607 | 0.07155 |
| Q9Y2P4 | S27A6_HUMAN | Long-chain fatty acid transport protein 6 | 0.04755 | -0.33495 |
| P13284 | GILT_HUMAN | Gamma-interferon-inducible lysosomal thiol reductase | 0.04831 | 0.32190 |
| P09429 | HMGB1_HUMAN | High mobility group protein B1 | 0.04869 | 0.11305 |
| P49407 | ARRB1_HUMAN | Beta-arrestin-1 | 0.04987 | 0.14853 |
| Q68CZ2 | TENS3_HUMAN | Tensin-3 | 0.04998 | 0.11696 |
| Q9P246-2 | STIM2_HUMAN | Isoform 2 of Stromal interaction molecule 2 | 0.05109 | -0.12691 |
| P00367 | DHE3_HUMAN | Glutamate dehydrogenase 1, mitochondrial | 0.0512 | -0.11132 |
| Q9NZQ7 | PD1L1_HUMAN | Programmed cell death 1 ligand 1 | 0.05181 | -0.21272 |
| Q15029 | U5S1_HUMAN | 116 kDa U5 small nuclear ribonucleoprotein component | 0.05223 | 0.16683 |
| P22626 | ROA2_HUMAN | Heterogeneous nuclear ribonucleoproteins A2/B1 | 0.05233 | 0.16246 |
| Q86XR7-2 | TCAM2_HUMAN | Isoform 2 of TIR domain-containing adapter molecule 2 | 0.05311 | -0.13237 |
| Q12907 | LMAN2_HUMAN | Vesicular integral-membrane protein VIP36 | 0.05349 | -0.08663 |
| O00159 | MYO1C_HUMAN | Unconventional myosin-Ic | 0.05382 | 0.18121 |
| P08195 | 4F2_HUMAN | 4F2 cell-surface antigen heavy chain | 0.05474 | -0.08037 |
| Q9NZJ7 | MTCH1_HUMAN | Mitochondrial carrier homolog 1 | 0.05494 | 0.16879 |
| P02768 | ALBU_HUMAN | Serum albumin | 0.05565 | -0.20798 |
| P07384 | CAN1_HUMAN | Calpain-1 catalytic subunit | 0.05574 | 0.17095 |
| O60884 | DNJA2_HUMAN | DnaJ homolog subfamily A member 2 | 0.05605 | 0.07856 |
| P32119 | PRDX2_HUMAN | Peroxiredoxin-2 | 0.05687 | -0.24447 |
| Q00169 | PIPNA_HUMAN | Phosphatidylinositol transfer protein alpha isoform | 0.05699 | -0.17113 |
| O43865 | SAHH2_HUMAN | S-adenosylhomocysteine hydrolase-like protein 1 | 0.05773 | 0.44208 |
| Q16881 | TRXR1_HUMAN | Thioredoxin reductase 1, cytoplasmic | 0.05801 | 0.16230 |
| Q15363 | TMED2_HUMAN | Transmembrane emp24 domain-containing protein 2 | 0.05899 | -0.11555 |
| Q9NQC3 | RTN4_HUMAN | Reticulon-4 | 0.06039 | -0.08244 |
| P46940 | IQGA1_HUMAN | Ras GTPase-activating-like protein IQGAP1 | 0.06144 | 0.07096 |
| P49368 | TCPG_HUMAN | T-complex protein 1 subunit gamma | 0.06262 | 0.06189 |
| P09758 | TACD2_HUMAN | Tumor-associated calcium signal transducer 2 | 0.06286 | -0.11739 |
| P49257 | LMAN1_HUMAN | Protein ERGIC-53 | 0.06295 | -0.07542 |
| P46777 | RL5_HUMAN | 60S ribosomal protein L5 | 0.06307 | 0.06645 |
| Q09666 | AHNK_HUMAN | Neuroblast differentiation-associated protein AHNAK | 0.06411 | 0.16630 |
| O15355 | PPM1G_HUMAN | Protein phosphatase 1G | 0.06461 | 0.19524 |
| P27487 | DPP4_HUMAN | Dipeptidyl peptidase 4 | 0.06617 | -0.05679 |
| P62906 | RL10A_HUMAN | 60S ribosomal protein L10a | 0.06683 | 0.09080 |
| P60842 | IF4A1_HUMAN | Eukaryotic initiation factor 4A-I | 0.067 | 0.09088 |
| Q5EBM0 | CMPK2_HUMAN | UMP-CMP kinase 2, mitochondrial | 0.0675 | -0.41581 |
| O94919 | ENDD1_HUMAN | Endonuclease domain-containing 1 protein | 0.06794 | -0.47708 |
| Q7L576 | CYFP1_HUMAN | Cytoplasmic FMR1-interacting protein 1 | 0.06809 | 0.07213 |
| O15127 | SCAM2_HUMAN | Secretory carrier-associated membrane protein 2 | 0.06842 | -0.06314 |
| Q08380 | LG3BP_HUMAN | Galectin-3-binding protein | 0.06857 | -0.14572 |
| P02545 | LMNA_HUMAN | Prelamin-A/C | 0.06916 | 0.18490 |
| Q7Z7H5 | TMED4_HUMAN | Transmembrane emp24 domain-containing protein 4 | 0.06974 | -0.12839 |
| P51571 | SSRD_HUMAN | Translocon-associated protein subunit delta | 0.06999 | -0.07023 |
| P30084 | ECHM_HUMAN | Enoyl-CoA hydratase, mitochondrial | 0.07189 | 0.11105 |
| O43681 | ASNA_HUMAN | ATPase ASNA1 | 0.07282 | 0.18779 |
| P61978-3 | HNRPK_HUMAN | Isoform 3 of Heterogeneous nuclear ribonucleoprotein K | 0.07362 | 0.13777 |
| Q9NQG5 | RPR1B_HUMAN | Regulation of nuclear pre-mRNA domain-containing protein 1B | 0.07388 | -0.30525 |
| O15372 | EIF3H_HUMAN | Eukaryotic translation initiation factor 3 subunit H | 0.07395 | 0.06831 |
| P62273 | RS29_HUMAN | 40S ribosomal protein S29 | 0.07407 | 0.11023 |
| P27635 | RL10_HUMAN | 60S ribosomal protein L10 | 0.07631 | 0.15388 |
| P13489 | RINI_HUMAN | Ribonuclease inhibitor | 0.07638 | 0.17415 |
| P42126 | ECI1_HUMAN | Enoyl-CoA delta isomerase 1, mitochondrial | 0.07658 | -0.06707 |
| Q8NE71 | ABCF1_HUMAN | ATP-binding cassette sub-family F member 1 | 0.07916 | 0.09548 |
| Q9NUQ9 | FA49B_HUMAN | Protein FAM49B | 0.08003 | -0.20026 |
| P46778 | RL21_HUMAN | 60S ribosomal protein L21 | 0.0805 | 0.11130 |
| O14579 | COPE_HUMAN | Coatomer subunit epsilon | 0.08197 | 0.10600 |
| P60900 | PSA6_HUMAN | Proteasome subunit alpha type-6 | 0.08244 | -0.03001 |
| Q9HDC9 | APMAP_HUMAN | Adipocyte plasma membrane-associated protein | 0.08387 | -0.10832 |
| P52907 | CAZA1_HUMAN | F-actin-capping protein subunit alpha-1 | 0.08458 | -0.14286 |
| Q9P258 | RCC2_HUMAN | Protein RCC2 | 0.08698 | 0.10218 |
| P61086 | UBE2K_HUMAN | Ubiquitin-conjugating enzyme E2 K | 0.08712 | 0.15419 |
| P62937 | PPIA_HUMAN | Peptidyl-prolyl cis-trans isomerase A | 0.08785 | 0.08490 |
| P07858 | CATB_HUMAN | Cathepsin B | 0.08799 | 0.19702 |
| Q13636 | RAB31_HUMAN | Ras-related protein Rab-31 | 0.08856 | 0.15317 |
| P07996 | TSP1_HUMAN | Thrombospondin-1 | 0.08917 | -0.18796 |
| P27348 | 1433T_HUMAN | 14-3-3 protein theta | 0.08923 | -0.35755 |
| P18124 | RL7_HUMAN | 60S ribosomal protein L7 | 0.09038 | 0.14316 |
| P26640 | SYVC_HUMAN | Valine--tRNA ligase | 0.09162 | 0.15288 |
| Q7KZF4 | SND1_HUMAN | Staphylococcal nuclease domain-containing protein 1 | 0.09247 | 0.18424 |
| P00747 | PLMN_HUMAN | Plasminogen | 0.09385 | -0.17295 |
| Q9Y266 | NUDC_HUMAN | Nuclear migration protein nudC | 0.09393 | 0.08144 |
| Q96CV9-2 | OPTN_HUMAN | Isoform 2 of Optineurin | 0.09534 | -0.12221 |
| Q5EB52 | MEST_HUMAN | Mesoderm-specific transcript homolog protein | 0.09536 | 0.33045 |
| P54920 | SNAA_HUMAN | Alpha-soluble NSF attachment protein | 0.09543 | -0.11530 |
| P62333 | PRS10_HUMAN | 26S proteasome regulatory subunit 10B | 0.09608 | 0.26898 |
| P53007 | TXTP_HUMAN | Tricarboxylate transport protein, mitochondrial | 0.09629 | -0.14791 |
| P11166 | GTR1_HUMAN | Solute carrier family 2, facilitated glucose transporter member 1 | 0.09761 | -0.06620 |
| Q16666 | IF16_HUMAN | Gamma-interferon-inducible protein 16 | 0.09795 | -0.13953 |
| Q14696 | MESD_HUMAN | LRP chaperone MESD | 0.09818 | 0.30637 |
| Q8NBJ5 | GT251_HUMAN | Procollagen galactosyltransferase 1 | 0.09858 | 0.15420 |
| Q9UJ70 | NAGK_HUMAN | N-acetyl-D-glucosamine kinase | 0.09896 | -0.28196 |
| O75351 | VPS4B_HUMAN | Vacuolar protein sorting-associated protein 4B | 0.09941 | -0.15980 |
| Q86U42 | PABP2_HUMAN | Polyadenylate-binding protein 2 | 0.10003 | 0.07986 |
| O95573 | ACSL3_HUMAN | Long-chain-fatty-acid--CoA ligase 3 | 0.10024 | 0.06791 |
| P62330 | ARF6_HUMAN | ADP-ribosylation factor 6 | 0.10046 | 0.10911 |
| O00299 | CLIC1_HUMAN | Chloride intracellular channel protein 1 | 0.10058 | -0.09809 |
| Q8N5K1 | CISD2_HUMAN | CDGSH iron-sulfur domain-containing protein 2 | 0.10101 | -0.12662 |
| P17813 | EGLN_HUMAN | Endoglin | 0.10114 | -0.19832 |
| P51149 | RAB7A_HUMAN | Ras-related protein Rab-7a | 0.10262 | 0.08767 |
| Q07955 | SRSF1_HUMAN | Serine/arginine-rich splicing factor 1 | 0.10308 | 0.15706 |
| Q99798 | ACON_HUMAN | Aconitate hydratase, mitochondrial | 0.1035 | -0.06738 |
| P62841 | RS15_HUMAN | 40S ribosomal protein S15 | 0.10387 | 0.14247 |
| O95571 | ETHE1_HUMAN | Persulfide dioxygenase ETHE1, mitochondrial | 0.10522 | 0.18295 |
| P83731 | RL24_HUMAN | 60S ribosomal protein L24 | 0.1057 | 0.10836 |
| P53384 | NUBP1_HUMAN | Cytosolic Fe-S cluster assembly factor NUBP1 | 0.10583 | 0.14960 |
| P18754 | RCC1_HUMAN | Regulator of chromosome condensation | 0.10769 | 0.07125 |
| P04066 | FUCO_HUMAN | Tissue alpha-L-fucosidase | 0.10808 | -0.26103 |
| P02774 | VTDB_HUMAN | Vitamin D-binding protein | 0.1092 | -0.15702 |
| P39023 | RL3_HUMAN | 60S ribosomal protein L3 | 0.11029 | 0.07745 |
| P15428-5 | PGDH_HUMAN | Isoform 5 of 15-hydroxyprostaglandin dehydrogenase [NAD(+)] | 0.11067 | 0.15319 |
| Q9Y5U9 | IR3IP_HUMAN | Immediate early response 3-interacting protein 1 | 0.11085 | -0.08102 |
| O43615 | TIM44_HUMAN | Mitochondrial import inner membrane translocase subunit TIM44 | 0.1115 | 0.16946 |
| O60684 | IMA7_HUMAN | Importin subunit alpha-7 | 0.11273 | 0.09860 |
| O00764 | PDXK_HUMAN | Pyridoxal kinase | 0.11357 | 0.24681 |
| P00403 | COX2_HUMAN | Cytochrome c oxidase subunit 2 | 0.11404 | -0.09259 |
| P46459 | NSF_HUMAN | Vesicle-fusing ATPase | 0.1148 | 0.18464 |
| O75822 | EIF3J_HUMAN | Eukaryotic translation initiation factor 3 subunit J | 0.1152 | 0.11186 |
| P09543 | CN37_HUMAN | 2',3'-cyclic-nucleotide 3'-phosphodiesterase | 0.11554 | -0.15797 |
| P53680 | AP2S1_HUMAN | AP-2 complex subunit sigma | 0.11983 | 0.07098 |
| Q9UDY2 | ZO2_HUMAN | Tight junction protein Z | 0.12076 | 0.16247 |
| Q15642 | CIP4_HUMAN | Cdc42-interacting protein 4 | 0.12178 | 0.08297 |
| P49748 | ACADV_HUMAN | Very long-chain specific acyl-CoA dehydrogenase, mitochondrial | 0.12218 | 0.08199 |
| Q14444 | CAPR1_HUMAN | Caprin-1 | 0.12259 | 0.10645 |
| P13073 | COX41_HUMAN | Cytochrome c oxidase subunit 4 isoform 1, mitochondrial | 0.12306 | -0.12560 |
| P02649 | APOE_HUMAN | Apolipoprotein E | 0.12331 | 0.21010 |
| P53801 | PTTG_HUMAN | Pituitary tumor-transforming gene 1 protein-interacting protein | 0.124 | -0.12760 |
| Q99623 | PHB2_HUMAN | Prohibitin-2 | 0.12426 | -0.08860 |
| P08174-7 | DAF_HUMAN | Isoform 7 of Complement decay-accelerating factor | 0.12445 | -0.20688 |
| P62424 | RL7A_HUMAN | 60S ribosomal protein L7a | 0.12475 | 0.13973 |
| P20339 | RAB5A_HUMAN | Ras-related protein Rab-5A | 0.12486 | 0.05077 |
| Q9H9B4 | SFXN1_HUMAN | Sideroflexin-1 | 0.1257 | -0.18692 |
| P62753 | RS6_HUMAN | 40S ribosomal protein S6 | 0.12675 | 0.16095 |
| P61225 | RAP2B_HUMAN | Ras-related protein Rap-2b | 0.12711 | 0.06152 |
| O75390 | CISY_HUMAN | Citrate synthase, mitochondrial | 0.12845 | 0.10332 |
| P24534 | EF1B_HUMAN | Elongation factor 1-beta | 0.12861 | 0.06017 |
| O00264 | PGRC1_HUMAN | Membrane-associated progesterone receptor component 1 | 0.12931 | -0.10741 |
| Q9BYX4 | IFIH1_HUMAN | Interferon-induced helicase C domain-containing protein 1 | 0.12964 | -0.30228 |
| O75083 | WDR1_HUMAN | WD repeat-containing protein 1 | 0.12992 | 0.03512 |
| P04040 | CATA_HUMAN | Catalase | 0.13096 | -0.12314 |
| P54886 | P5CS_HUMAN | Delta-1-pyrroline-5-carboxylate synthase | 0.13123 | 0.24644 |
| Q14728 | MFS10_HUMAN | Major facilitator superfamily domain-containing protein 10 | 0.13135 | -0.14461 |
| P01024 | CO3_HUMAN | Complement C3 | 0.13154 | -0.13294 |
| P54577 | SYYC_HUMAN | Tyrosine--tRNA ligase, cytoplasmic | 0.13176 | -0.10132 |
| Q02818 | NUCB1_HUMAN | Nucleobindin-1 | 0.13242 | -0.12399 |
| Q9UJS0 | CMC2_HUMAN | Calcium-binding mitochondrial carrier protein Aralar2 | 0.13301 | -0.09155 |
| P53041 | PPP5_HUMAN | Serine/threonine-protein phosphatase 5 | 0.13366 | 0.27970 |
| P34932 | HSP74_HUMAN | Heat shock 70 kDa protein 4 | 0.13593 | 0.03436 |
| P35232 | PHB_HUMAN | Prohibitin | 0.13608 | -0.09223 |
| P25786 | PSA1_HUMAN | Proteasome subunit alpha type-1 | 0.13626 | 0.16514 |
| P37837 | TALDO_HUMAN | Transaldolase | 0.13659 | 0.05938 |
| Q99816 | TS101_HUMAN | Tumor susceptibility gene 101 protein | 0.13755 | 0.04074 |
| P49589 | SYCC_HUMAN | Cysteine--tRNA ligase, cytoplasmic | 0.1393 | -0.06697 |
| P02765 | FETUA_HUMAN | Alpha-2-HS-glycoprotein | 0.13989 | -0.10963 |
| Q13445 | TMED1_HUMAN | Transmembrane emp24 domain-containing protein 1 | 0.14141 | -0.14434 |
| Q14152 | EIF3A_HUMAN | Eukaryotic translation initiation factor 3 subunit A | 0.14195 | 0.03524 |
| P05556 | ITB1_HUMAN | Integrin beta-1 | 0.14209 | -0.05384 |
| P52272 | HNRPM_HUMAN | Heterogeneous nuclear ribonucleoprotein M | 0.14255 | 0.11675 |
| P18085 | ARF4_HUMAN | ADP-ribosylation factor 4 | 0.14259 | 0.09145 |
| Q12874 | SF3A3_HUMAN | Splicing factor 3A subunit 3 | 0.14353 | 0.05303 |
| O43278 | SPIT1_HUMAN | Kunitz-type protease inhibitor 1 | 0.14477 | -0.08856 |
| P14902 | I23O1_HUMAN | Indoleamine 2,3-dioxygenase 1 | 0.14521 | -0.35968 |
| Q02978 | M2OM_HUMAN | Mitochondrial 2-oxoglutarate/malate carrier protein | 0.14611 | -0.21615 |
| Q9BSJ8 | ESYT1_HUMAN | Extended synaptotagmin-1 | 0.1465 | 0.10971 |
| P31689 | DNJA1_HUMAN | DnaJ homolog subfamily A member 1 | 0.14666 | -0.13047 |
| P62995 | TRA2B_HUMAN | Transformer-2 protein homolog beta | 0.14684 | 0.12901 |
| P55084 | ECHB_HUMAN | Trifunctional enzyme subunit beta, mitochondrial | 0.14741 | 0.08910 |
| P15954 | COX7C_HUMAN | Cytochrome c oxidase subunit 7C, mitochondrial | 0.14789 | -0.13470 |
| O94905 | ERLN2_HUMAN | Erlin-2 | 0.14804 | 0.06044 |
| Q9H4A4 | AMPB_HUMAN | Aminopeptidase B | 0.1486 | 0.12258 |
| P20936 | RASA1_HUMAN | Ras GTPase-activating protein 1 | 0.15017 | 0.13776 |
| O95292 | VAPB_HUMAN | Vesicle-associated membrane protein-associated protein B/C | 0.15025 | 0.05973 |
| Q15907 | RB11B_HUMAN | Ras-related protein Rab-11B | 0.15097 | 0.02998 |
| P52565 | GDIR1_HUMAN | Rho GDP-dissociation inhibitor 1 | 0.15272 | 0.11778 |
| Q14257 | RCN2_HUMAN | Reticulocalbin-2 | 0.15295 | 0.08617 |
| P47914 | RL29_HUMAN | 60S ribosomal protein L29 | 0.15329 | 0.10226 |
| O43776 | SYNC_HUMAN | Asparagine--tRNA ligase, cytoplasmic | 0.1534 | 0.13025 |
| P05204 | HMGN2_HUMAN | Non-histone chromosomal protein HMG-17 | 0.15351 | 0.23303 |
| P50281 | MMP14_HUMAN | Matrix metalloproteinase-14 | 0.15361 | -0.27030 |
| Q14157 | UBP2L_HUMAN | Ubiquitin-associated protein 2-like | 0.15387 | 0.10603 |
| P52790 | HXK3_HUMAN | Hexokinase-3 | 0.15479 | -0.11252 |
| Q9Y2A7 | NCKP1_HUMAN | Nck-associated protein 1 | 0.155 | 0.27470 |
| Q92841 | DDX17_HUMAN | Probable ATP-dependent RNA helicase DDX17 | 0.15502 | 0.07431 |
| P35573 | GDE_HUMAN | Glycogen debranching enzyme | 0.15516 | 0.28367 |
| P02788 | TRFL_HUMAN | Lactotransferrin | 0.15617 | -0.17905 |
| Q9UH65 | SWP70_HUMAN | Switch-associated protein 70 | 0.15771 | 0.05159 |
| Q00610 | CLH1_HUMAN | Clathrin heavy chain 1 | 0.15775 | 0.05404 |
| Q12906-7 | ILF3_HUMAN | Isoform 7 of Interleukin enhancer-binding factor 3 | 0.15786 | 0.13702 |
| P21964 | COMT_HUMAN | Catechol | 0.15798 | 0.07065 |
| P61026 | RAB10_HUMAN | Ras-related protein Rab-10 | 0.15861 | 0.05134 |
| P02749 | APOH_HUMAN | Beta-2-glycoprotein 1 | 0.16007 | -0.09380 |
| Q15365 | PCBP1_HUMAN | Poly(rC)-binding protein 1 | 0.1609 | 0.10212 |
| P63104 | 1433Z_HUMAN | 14-3-3 protein zeta/delta | 0.16269 | -0.16854 |
| Q16543 | CDC37_HUMAN | Hsp90 co-chaperone Cdc37 | 0.16355 | 0.06317 |
| P02760 | AMBP_HUMAN | Protein AMBP | 0.16421 | -0.12538 |
| Q9H3N1 | TMX1_HUMAN | Thioredoxin-related transmembrane protein 1 | 0.16447 | -0.09836 |
| P04632 | CPNS1_HUMAN | Calpain small subunit 1 | 0.16461 | -0.12301 |
| Q13011 | ECH1_HUMAN | Delta(3,5)-Delta(2,4)-dienoyl-CoA isomerase, mitochondrial | 0.16528 | -0.15147 |
| P11413-2 | G6PD_HUMAN | Isoform Long of Glucose-6-phosphate 1-dehydrogenase | 0.16683 | 0.13170 |
| P17174 | AATC_HUMAN | Aspartate aminotransferase, cytoplasmic | 0.16739 | 0.16892 |
| P22695 | QCR2_HUMAN | Cytochrome b-c1 complex subunit 2, mitochondrial | 0.16836 | -0.11340 |
| Q15428 | SF3A2_HUMAN | Splicing factor 3A subunit 2 | 0.16846 | 0.07181 |
| O15260 | SURF4_HUMAN | Surfeit locus protein 4 | 0.16868 | 0.05021 |
| P61966 | AP1S1_HUMAN | AP-1 complex subunit sigma-1A | 0.1702 | 0.13684 |
| P33176 | KINH_HUMAN | Kinesin-1 heavy chain | 0.17108 | 0.17877 |
| Q16891 | MIC60_HUMAN | MIC | 0.17138 | 0.10764 |
| Q9H223 | EHD4_HUMAN | EH domain-containing protein 4 | 0.172 | -0.12204 |
| Q13283 | G3BP1_HUMAN | Ras GTPase-activating protein-binding protein 1 | 0.1722 | 0.14330 |
| Q06830 | PRDX1_HUMAN | Peroxiredoxin-1 | 0.17287 | 0.03857 |
| P00338 | LDHA_HUMAN | L-lactate dehydrogenase A chain | 0.17301 | 0.03180 |
| Q9NZ45 | CISD1_HUMAN | CDGSH iron-sulfur domain-containing protein 1 | 0.17367 | -0.08605 |
| A0FGR8 | ESYT2_HUMAN | Extended synaptotagmin-2 | 0.17368 | 0.09688 |
| P20042 | IF2B_HUMAN | Eukaryotic translation initiation factor 2 subunit 2 | 0.17443 | -0.13483 |
| Q9BRP8 | PYM1_HUMAN | Partner of Y14 and mago | 0.17618 | 0.23091 |
| Q5RI15 | COX20_HUMAN | Cytochrome c oxidase assembly protein C | 0.17646 | -0.07578 |
| P08758 | ANXA5_HUMAN | Annexin A5 | 0.17649 | -0.04076 |
| Q9H7M9 | VISTA_HUMAN | V-type immunoglobulin domain-containing suppressor of T-cell activation | 0.1772 | 0.14520 |
| P00505 | AATM_HUMAN | Aspartate aminotransferase, mitochondrial | 0.17861 | -0.11813 |
| P51970 | NDUA8_HUMAN | NADH dehydrogenase [ubiquinone] 1 alpha subcomplex subunit 8 | 0.17864 | -0.23282 |
| P05107 | ITB2_HUMAN | Integrin beta-2 | 0.17865 | 0.22132 |
| Q9H6K4 | OPA3_HUMAN | Optic atrophy3 protein | 0.18062 | -0.06009 |
| Q9BVK6 | TMED9_HUMAN | Transmembrane emp24 domain-containing protein 9 | 0.1811 | -0.11477 |
| Q14254 | FLOT2_HUMAN | Flotillin-2 | 0.18117 | -0.10711 |
| P09669 | COX6C_HUMAN | Cytochrome c oxidase subunit 6C | 0.18218 | -0.12670 |
| P08574 | CY1_HUMAN | Cytochrome c1, heme protein, mitochondrial | 0.18297 | -0.10049 |
| P62241 | RS8_HUMAN | 40S ribosomal protein S8 | 0.18534 | 0.11992 |
| P54578 | UBP14_HUMAN | Ubiquitin carboxyl-terminal hydrolase 14 | 0.18596 | 0.09556 |
| P62280 | RS11_HUMAN | 40S ribosomal protein S11 | 0.1869 | 0.14535 |
| P78371 | TCPB_HUMAN | T-complex protein 1 subunit beta | 0.18783 | 0.07377 |
| P56192 | SYMC_HUMAN | Methionine--tRNA ligase, cytoplasmic | 0.18916 | 0.11308 |
| Q9Y6E0 | STK24_HUMAN | Serine/threonine-protein kinase 24 | 0.18919 | 0.05436 |
| Q93077 | H2A1C_HUMAN | Histone H2A type 1-C | 0.18963 | 0.14158 |
| P07910 | HNRPC_HUMAN | Heterogeneous nuclear ribonucleoproteins C1/C2 | 0.19048 | 0.14542 |
| Q9NX40 | OCAD1_HUMAN | OCIA domain-containing protein 1 | 0.19124 | -0.12203 |
| Q9BUF5 | TBB6_HUMAN | Tubulin beta-6 chain | 0.19278 | 0.17312 |
| Q8TCJ2 | STT3B_HUMAN | Dolichyl-diphosphooligosaccharide--protein glycosyltransferase subunit STT3B | 0.19337 | 0.06561 |
| Q15274 | NADC_HUMAN | Nicotinate-nucleotide pyrophosphorylase [carboxylating] | 0.19392 | -0.06436 |
| P29590-9 | PML_HUMAN | Isoform PML-3 of Protein PML | 0.19432 | -0.11364 |
| P49321 | NASP_HUMAN | Nuclear autoantigenic sperm protein | 0.19448 | -0.03867 |
| Q16181 | SEPT7_HUMAN | Septin-7 | 0.19473 | 0.08468 |
| Q9P2J5 | SYLC_HUMAN | Leucine--tRNA ligase, cytoplasmic | 0.19537 | 0.19251 |
| O75643 | U520_HUMAN | U5 small nuclear ribonucleoprotein 200 kDa helicase | 0.19614 | 0.16691 |
| P61313 | RL15_HUMAN | 60S ribosomal protein L15 | 0.19666 | 0.09093 |
| P08779 | K1C16_HUMAN | Keratin, type I cytoskeletal 16 | 0.1969 | -0.07495 |
| Q96CS3 | FAF2_HUMAN | FAS-associated factor 2 | 0.19694 | 0.03896 |
| P60228 | EIF3E_HUMAN | Eukaryotic translation initiation factor 3 subunit E | 0.19798 | 0.13074 |
| Q03135 | CAV1_HUMAN | Caveolin-1 | 0.19911 | -0.07259 |
| Q8WVM8 | SCFD1_HUMAN | Sec1 family domain-containing protein 1 | 0.20145 | 0.11110 |
| P68400 | CSK21_HUMAN | Casein kinase II subunit alpha | 0.20182 | -0.12952 |
| Q07075 | AMPE_HUMAN | Glutamyl aminopeptidase | 0.2024 | -0.09997 |
| P69905 | HBA_HUMAN | Hemoglobin subunit alpha | 0.20343 | -0.14557 |
| P14868 | SYDC_HUMAN | Aspartate--tRNA ligase, cytoplasmic | 0.2036 | 0.03905 |
| P14618 | KPYM_HUMAN | Pyruvate kinase PKM | 0.20366 | 0.07898 |
| Q9Y262 | EIF3L_HUMAN | Eukaryotic translation initiation factor 3 subunit L | 0.20552 | -0.10380 |
| P26641 | EF1G_HUMAN | Elongation factor 1-gamma | 0.20635 | 0.04760 |
| O00303 | EIF3F_HUMAN | Eukaryotic translation initiation factor 3 subunit F | 0.20698 | 0.03201 |
| Q99805 | TM9S2_HUMAN | Transmembrane 9 superfamily member 2 | 0.20791 | -0.08752 |
| Q16629 | SRSF7_HUMAN | Serine/arginine-rich splicing factor 7 | 0.20836 | 0.07736 |
| P20700 | LMNB1_HUMAN | Lamin-B1 | 0.21009 | 0.09133 |
| P55786 | PSA_HUMAN | Puromycin-sensitive aminopeptidase | 0.2101 | 0.04604 |
| O14949 | QCR8_HUMAN | Cytochrome b-c1 complex subunit 8 | 0.21061 | -0.11120 |
| Q7L5L3 | GDPD3_HUMAN | Lysophospholipase D GDPD3 | 0.21086 | -0.15966 |
| P04792 | HSPB1_HUMAN | Heat shock protein beta-1 | 0.21089 | 0.13581 |
| P21397 | AOFA_HUMAN | Amine oxidase [flavin-containing] A | 0.21156 | -0.13094 |
| P25788 | PSA3_HUMAN | Proteasome subunit alpha type-3 | 0.21252 | -0.03521 |
| Q9Y285 | SYFA_HUMAN | Phenylalanine--tRNA ligase alpha subunit | 0.21313 | 0.05259 |
| Q99961 | SH3G1_HUMAN | Endophilin-A2 | 0.21367 | 0.07914 |
| O75821 | EIF3G_HUMAN | Eukaryotic translation initiation factor 3 subunit G | 0.21388 | 0.02388 |
| O14773 | TPP1_HUMAN | Tripeptidyl-peptidase 1 | 0.21392 | 0.07283 |
| P42574 | CASP3_HUMAN | Caspase-3 | 0.21418 | -0.12011 |
| Q7Z2K6 | ERMP1_HUMAN | Endoplasmic reticulum metallopeptidase 1 | 0.21438 | 0.14790 |
| P51665 | PSMD7_HUMAN | 26S proteasome non-ATPase regulatory subunit 7 | 0.21489 | -0.11426 |
| Q92544 | TM9S4_HUMAN | Transmembrane 9 superfamily member 4 | 0.21512 | 0.11537 |
| Q16539 | MK14_HUMAN | Mitogen-activated protein kinase 14 | 0.21512 | 0.05364 |
| P27694 | RFA1_HUMAN | Replication protein A 70 kDa DNA-binding subunit | 0.21609 | 0.04090 |
| Q9H6S3 | ES8L2_HUMAN | Epidermal growth factor receptor kinase substrate 8-like protein 2 | 0.21625 | 0.17163 |
| Q96NY8 | NECT4_HUMAN | Nectin-4 | 0.21695 | -0.10658 |
| Q00325-2 | MPCP_HUMAN | Isoform B of Phosphate carrier protein, mitochondrial | 0.21829 | -0.06688 |
| P09651 | ROA1_HUMAN | Heterogeneous nuclear ribonucleoprotein A1 | 0.22159 | 0.17713 |
| P67809 | YBOX1_HUMAN | Nuclease-sensitive element-binding protein 1 | 0.22207 | 0.09161 |
| P68371 | TBB4B_HUMAN | Tubulin beta-4B chain | 0.22213 | 0.11988 |
| P39656 | OST48_HUMAN | Dolichyl-diphosphooligosaccharide--protein glycosyltransferase 48 kDa subunit | 0.22438 | -0.04152 |
| Q01650 | LAT1_HUMAN | Large neutral amino acids transporter small subunit 1 | 0.22505 | -0.10805 |
| Q99536 | VAT1_HUMAN | Synaptic vesicle membrane protein VAT-1 homolog | 0.22655 | 0.11683 |
| Q92597 | NDRG1_HUMAN | Protein NDRG1 | 0.22683 | -0.09530 |
| P05141 | ADT2_HUMAN | ADP/ATP translocase 2 | 0.22793 | -0.05990 |
| A6NCS6 | CB072_HUMAN | Uncharacterized protein C2orf72 | 0.22874 | 0.17012 |
| P06737 | PYGL_HUMAN | Glycogen phosphorylase, liver form | 0.22919 | 0.11588 |
| Q9NUB1 | ACS2L_HUMAN | Acetyl-coenzyme A synthetase 2-like, mitochondrial | 0.23031 | 0.13276 |
| P17987 | TCPA_HUMAN | T-complex protein 1 subunit alpha | 0.23265 | 0.03909 |
| P30038 | AL4A1_HUMAN | Delta-1-pyrroline-5-carboxylate dehydrogenase, mitochondrial | 0.23342 | 0.19466 |
| Q92598 | HS105_HUMAN | Heat shock protein 105 kDa | 0.23397 | 0.08722 |
| Q9BTM1 | H2AJ_HUMAN | Histone H2A.J | 0.23447 | 0.19776 |
| Q15637-5 | SF01_HUMAN | Isoform 5 of Splicing factor 1 | 0.23481 | 0.16804 |
| Q07812-5 | BAX_HUMAN | Isoform Epsilon of Apoptosis regulator BAX | 0.2349 | -0.07314 |
| Q9UNL2 | SSRG_HUMAN | Translocon-associated protein subunit gamma | 0.23535 | -0.04241 |
| P11233 | RALA_HUMAN | Ras-related protein Ral-A | 0.2359 | 0.10306 |
| P35606 | COPB2_HUMAN | Coatomer subunit beta' | 0.23659 | 0.03632 |
| P30044 | PRDX5_HUMAN | Peroxiredoxin-5, mitochondrial | 0.23664 | 0.09229 |
| P24539 | AT5F1_HUMAN | ATP synthase F(0) complex subunit B1, mitochondrial | 0.23765 | -0.07271 |
| Q96HC4 | PDLI5_HUMAN | PDZ and LIM domain protein 5 | 0.23782 | 0.06294 |
| Q9NUU7 | DD19A_HUMAN | ATP-dependent RNA helicase DDX19A | 0.23808 | 0.15776 |
| P17655 | CAN2_HUMAN | Calpain-2 catalytic subunit | 0.23984 | 0.15401 |
| P11498 | PYC_HUMAN | Pyruvate carboxylase, mitochondrial | 0.24145 | 0.06181 |
| Q29963 | 1C06_HUMAN | HLA class I histocompatibility antigen, Cw-6 alpha chain | 0.2416 | -0.18467 |
| P17096 | HMGA1_HUMAN | High mobility group protein HMG-I/HMG-Y | 0.24171 | 0.14368 |
| P14406 | CX7A2_HUMAN | Cytochrome c oxidase subunit 7A2, mitochondrial | 0.24187 | -0.04461 |
| O00231 | PSD11_HUMAN | 26S proteasome non-ATPase regulatory subunit 11 | 0.2439 | 0.03830 |
| P28331-2 | NDUS1_HUMAN | Isoform 2 of NADH-ubiquinone oxidoreductase 75 kDa subunit, mitochondrial | 0.24396 | -0.07164 |
| Q14126 | DSG2_HUMAN | Desmoglein-2 | 0.24627 | 0.27567 |
| Q5JTV8 | TOIP1_HUMAN | Torsin-1A-interacting protein 1 | 0.24718 | -0.10795 |
| P55060 | XPO2_HUMAN | Exportin-2 | 0.24975 | 0.08509 |
| P30153 | 2AAA_HUMAN | Serine/threonine-protein phosphatase 2A 65 kDa regulatory subunit A alpha isoform | 0.25041 | 0.03927 |
| Q14697 | GANAB_HUMAN | Neutral alpha-glucosidase AB | 0.25314 | 0.04826 |
| Q9Y383 | LC7L2_HUMAN | Putative RNA-binding protein Luc7-like 2 | 0.25429 | 0.08487 |
| Q9BVC6 | TM109_HUMAN | Transmembrane protein 109 | 0.25524 | -0.05419 |
| P36542 | ATPG_HUMAN | ATP synthase subunit gamma, mitochondrial | 0.25596 | -0.02974 |
| P61254 | RL26_HUMAN | 60S ribosomal protein L26 | 0.25599 | 0.09401 |
| Q08211 | DHX9_HUMAN | ATP-dependent RNA helicase A | 0.25755 | 0.08412 |
| P53621 | COPA_HUMAN | Coatomer subunit alpha | 0.25766 | 0.04326 |
| Q9H1B7 | I2BPL_HUMAN | Probable E3 ubiquitin-protein ligase IRF2BPL | 0.25831 | 0.14937 |
| P08621 | RU17_HUMAN | U1 small nuclear ribonucleoprotein 70 kDa | 0.25894 | 0.07012 |
| O43242 | PSMD3_HUMAN | 26S proteasome non-ATPase regulatory subunit 3 | 0.25894 | -0.26858 |
| P54652 | HSP72_HUMAN | Heat shock-related 70 kDa protein 2 | 0.25914 | 0.07966 |
| P25705 | ATPA_HUMAN | ATP synthase subunit alpha, mitochondrial | 0.25932 | 0.02993 |
| Q96B49 | TOM6_HUMAN | Mitochondrial import receptor subunit T | 0.25945 | -0.11223 |
| P19338 | NUCL_HUMAN | Nucleolin | 0.25964 | 0.08221 |
| Q9BXK5 | B2L13_HUMAN | Bcl-2-like protein 13 | 0.26216 | -0.20024 |
| P49207 | RL34_HUMAN | 60S ribosomal protein L34 | 0.26358 | 0.13477 |
| Q9Y6Q1 | CAN6_HUMAN | Calpain-6 | 0.26445 | 0.16737 |
| Q13576 | IQGA2_HUMAN | Ras GTPase-activating-like protein IQGAP2 | 0.26615 | 0.07054 |
| P20073 | ANXA7_HUMAN | Annexin A7 | 0.26696 | -0.04578 |
| Q15233 | NONO_HUMAN | Non-POU domain containing octamer-binding protein | 0.26945 | 0.19649 |
| O14818 | PSA7_HUMAN | Proteasome subunit alpha type-7 | 0.27008 | -0.08849 |
| Q96FJ2 | DYL2_HUMAN | Dynein light chain 2, cytoplasmic | 0.27268 | -0.19278 |
| P47897 | SYQ_HUMAN | Glutamine--tRNA ligase | 0.27289 | 0.12942 |
| Q13564 | ULA1_HUMAN | NEDD8-activating enzyme E1 regulatory subunit | 0.27289 | -0.28627 |
| Q07065 | CKAP4_HUMAN | Cytoskeleton-associated protein 4 | 0.27417 | -0.07871 |
| P55209 | NP1L1_HUMAN | Nucleosome assembly protein 1-like 1 | 0.27748 | 0.06495 |
| Q9Y6A9 | SPCS1_HUMAN | Signal peptidase complex subunit 1 | 0.27766 | -0.08380 |
| P50502 | F10A1_HUMAN | Hsc70-interacting protein | 0.27774 | 0.05592 |
| Q13813 | SPTN1_HUMAN | Spectrin alpha chain, non-erythrocytic 1 | 0.27793 | 0.08486 |
| Q92575 | UBXN4_HUMAN | UBX domain-containing protein 4 | 0.27846 | -0.14939 |
| P69891 | HBG1_HUMAN | Hemoglobin subunit gamma-1 | 0.27863 | -0.29555 |
| P04844 | RPN2_HUMAN | Dolichyl-diphosphooligosaccharide--protein glycosyltransferase subunit 2 | 0.27883 | -0.04095 |
| P16615 | AT2A2_HUMAN | Sarcoplasmic/endoplasmic reticulum calcium ATPase 2 | 0.27947 | 0.06480 |
| Q99733 | NP1L4_HUMAN | Nucleosome assembly protein 1-like 4 | 0.2801 | 0.11302 |
| P13798 | ACPH_HUMAN | Acylamino-acid-releasing enzyme | 0.28041 | 0.05531 |
| Q9UBI6 | GBG12_HUMAN | Guanine nucleotide-binding protein G(I)/G(S)/G( | 0.28158 | 0.15240 |
| O95236 | APOL3_HUMAN | Apolipoprotein L3 | 0.28163 | -0.14677 |
| P26599 | PTBP1_HUMAN | Polypyrimidine tract-binding protein 1 | 0.28177 | 0.04646 |
| P29466 | CASP1_HUMAN | Caspase-1 | 0.28239 | -0.12114 |
| P09622 | DLDH_HUMAN | Dihydrolipoyl dehydrogenase, mitochondrial | 0.28286 | -0.05278 |
| P48444 | COPD_HUMAN | Coatomer subunit delta | 0.28322 | 0.02596 |
| Q00765 | REEP5_HUMAN | Receptor expression-enhancing protein 5 | 0.28336 | -0.10752 |
| P07437 | TBB5_HUMAN | Tubulin beta chain | 0.28446 | 0.12178 |
| P08473 | NEP_HUMAN | Neprilysin | 0.28721 | -0.05625 |
| A0AVT1 | UBA6_HUMAN | Ubiquitin-like modifier-activating enzyme 6 | 0.28992 | 0.03337 |
| P27695 | APEX1_HUMAN | DNA-(apurinic or apyrimidinic site) lyase | 0.29022 | 0.04285 |
| P11940 | PABP1_HUMAN | Polyadenylate-binding protein 1 | 0.29219 | 0.05546 |
| P49247 | RPIA_HUMAN | Ribose-5-phosphate isomerase | 0.29468 | 0.13103 |
| P20674 | COX5A_HUMAN | Cytochrome c oxidase subunit 5A, mitochondrial | 0.29553 | -0.11755 |
| P16435 | NCPR_HUMAN | NADPH--cytochrome P450 reductase | 0.29618 | 0.03532 |
| O60443 | GSDME_HUMAN | Gasdermin-E | 0.2963 | 0.03547 |
| Q96TC7 | RMD3_HUMAN | Regulator of microtubule dynamics protein 3 | 0.2975 | -0.19479 |
| P07355-2 | ANXA2_HUMAN | Isoform 2 of Annexin A2 | 0.2985 | 0.08589 |
| P67812-3 | SC11A_HUMAN | Isoform 3 of Signal peptidase complex catalytic subunit SEC11A | 0.29877 | -0.05299 |
| P50453 | SPB9_HUMAN | Serpin B9 | 0.29899 | -0.08772 |
| P09467 | F16P1_HUMAN | Fructose-1,6-bisphosphatase 1 | 0.30027 | 0.20870 |
| P05120 | PAI2_HUMAN | Plasminogen activator inhibitor 2 | 0.30043 | 0.13570 |
| Q14764 | MVP_HUMAN | Major vault protein | 0.30047 | -0.09541 |
| Q5ZPR3 | CD276_HUMAN | CD276 antigen | 0.30115 | -0.14349 |
| Q9UN86 | G3BP2_HUMAN | Ras GTPase-activating protein-binding protein 2 | 0.3013 | 0.08129 |
| Q8WXF1 | PSPC1_HUMAN | Paraspeckle component 1 | 0.30274 | 0.09820 |
| P02786 | TFR1_HUMAN | Transferrin receptor protein 1 | 0.30329 | 0.07653 |
| E9PAV3 | NACAM_HUMAN | Nascent polypeptide-associated complex subunit alpha, muscle-specific form | 0.30553 | 0.03689 |
| Q86SG5 | S1A7A_HUMAN | Protein S100-A7A | 0.30635 | 0.34284 |
| Q70UQ0 | IKIP_HUMAN | Inhibitor of nuclear factor kappa-B kinase-interacting protein | 0.30766 | -0.12674 |
| P61224 | RAP1B_HUMAN | Ras-related protein Rap-1b | 0.30788 | 0.05041 |
| Q92499 | DDX1_HUMAN | ATP-dependent RNA helicase DDX1 | 0.30844 | 0.03342 |
| Q13045 | FLII_HUMAN | Protein flightless-1 homolog | 0.30949 | 0.04640 |
| P55884-2 | EIF3B_HUMAN | Isoform 2 of Eukaryotic translation initiation factor 3 subunit B | 0.31282 | 0.06330 |
| P0DMV9 | HS71B_HUMAN | Heat shock 70 kDa protein 1B | 0.31401 | -0.06912 |
| P15121 | ALDR_HUMAN | Aldo-keto reductase family 1 member B1 | 0.31533 | 0.08051 |
| Q9UL25 | RAB21_HUMAN | Ras-related protein Rab-21 | 0.3164 | 0.03451 |
| Q13188 | STK3_HUMAN | Serine/threonine-protein kinase 3 | 0.31652 | 0.08356 |
| Q92945 | FUBP2_HUMAN | Far upstream element-binding protein 2 | 0.31959 | 0.10473 |
| P36955 | PEDF_HUMAN | Pigment epithelium-derived factor | 0.31992 | -0.14962 |
| P08579 | RU2B_HUMAN | U2 small nuclear ribonucleoprotein B'' | 0.32014 | 0.07187 |
| Q15084 | PDIA6_HUMAN | Protein disulfide-isomerase A6 | 0.32095 | -0.03718 |
| Q99459 | CDC5L_HUMAN | Cell division cycle 5-like protein | 0.32135 | 0.10391 |
| P05165 | PCCA_HUMAN | Propionyl-CoA carboxylase alpha chain, mitochondrial | 0.32219 | 0.27075 |
| Q9H3P7 | GCP60_HUMAN | Golgi resident protein GCP60 | 0.32254 | 0.03759 |
| O94826 | TOM70_HUMAN | Mitochondrial import receptor subunit TOM70 | 0.32405 | 0.07014 |
| P62140 | PP1B_HUMAN | Serine/threonine-protein phosphatase PP1-beta catalytic subunit | 0.32455 | 0.07500 |
| P43307 | SSRA_HUMAN | Translocon-associated protein subunit alpha | 0.32571 | -0.04600 |
| Q9UHG3 | PCYOX_HUMAN | Prenylcysteine oxidase 1 | 0.32669 | 0.09434 |
| P14923 | PLAK_HUMAN | Junction plakoglobin | 0.32727 | 0.09917 |
| Q14980-2 | NUMA1_HUMAN | Isoform 2 of Nuclear mitotic apparatus protein 1 | 0.32886 | 0.11944 |
| P30533 | AMRP_HUMAN | Alpha-2-macroglobulin receptor-associated protein | 0.32941 | 0.06236 |
| Q9NYU2 | UGGG1_HUMAN | UDP-glucose:glycoprotein glucosyltransferase 1 | 0.33026 | -0.11235 |
| Q9UHB9 | SRP68_HUMAN | Signal recognition particle subunit SRP68 | 0.33056 | 0.10174 |
| Q96CN7 | ISOC1_HUMAN | Isochorismatase domain-containing protein 1 | 0.33098 | -0.05000 |
| P09960 | LKHA4_HUMAN | Leukotriene A-4 hydrolase | 0.33117 | 0.18387 |
| P15328 | FOLR1_HUMAN | Folate receptor alpha | 0.33202 | 0.13390 |
| Q9UP95 | S12A4_HUMAN | Solute carrier family 12 member 4 | 0.33322 | 0.07257 |
| P62310 | LSM3_HUMAN | U6 snRNA-associated Sm-like protein LSm3 | 0.33445 | -0.19707 |
| P12004 | PCNA_HUMAN | Proliferating cell nuclear antigen | 0.33699 | 0.03689 |
| P62873 | GBB1_HUMAN | Guanine nucleotide-binding protein G(I)/G(S)/G(T) subunit beta-1 | 0.33774 | 0.03334 |
| Q15691 | MARE1_HUMAN | Microtubule-associated protein RP/EB family member 1 | 0.33905 | -0.39510 |
| P27105 | STOM_HUMAN | Erythrocyte band 7 integral membrane protein | 0.33908 | -0.08401 |
| Q93009 | UBP7_HUMAN | Ubiquitin carboxyl-terminal hydrolase 7 | 0.33943 | 0.12193 |
| Q99541 | PLIN2_HUMAN | Perilipin-2 | 0.34063 | 0.06426 |
| Q15041 | AR6P1_HUMAN | ADP-ribosylation factor-like protein 6-interacting protein 1 | 0.34142 | -0.05100 |
| Q14165 | MLEC_HUMAN | Malectin | 0.3424 | -0.16152 |
| Q8NFQ8 | TOIP2_HUMAN | Torsin-1A-interacting protein 2 | 0.34248 | 0.19453 |
| P55735 | SEC13_HUMAN | Protein SEC13 homolog | 0.34298 | 0.27265 |
| P43243 | MATR3_HUMAN | Matrin-3 | 0.34352 | 0.17482 |
| P14314 | GLU2B_HUMAN | Glucosidase 2 subunit beta | 0.34399 | -0.02496 |
| P62910 | RL32_HUMAN | 60S ribosomal protein L32 | 0.34436 | 0.08900 |
| P13010 | XRCC5_HUMAN | X-ray repair cross-complementing protein 5 | 0.3458 | 0.05127 |
| O76094 | SRP72_HUMAN | Signal recognition particle subunit SRP72 | 0.3461 | 0.09527 |
| Q9UJZ1 | STML2_HUMAN | Stomatin-like protein 2, mitochondrial | 0.3474 | -0.06552 |
| Q13162 | PRDX4_HUMAN | Peroxiredoxin-4 | 0.3482 | -0.09306 |
| P68871 | HBB_HUMAN | Hemoglobin subunit beta | 0.34991 | -0.21801 |
| Q02790 | FKBP4_HUMAN | Peptidyl-prolyl cis-trans isomerase FKBP4 | 0.35066 | 0.03016 |
| P07741 | APT_HUMAN | Adenine phosphoribosyltransferase | 0.35127 | 0.06873 |
| P12830 | CADH1_HUMAN | Cadherin-1 | 0.35128 | -0.11455 |
| P23381 | SYWC_HUMAN | Tryptophan--tRNA ligase, cytoplasmic | 0.35383 | -0.13201 |
| O15173 | PGRC2_HUMAN | Membrane-associated progesterone receptor component 2 | 0.35384 | -0.05648 |
| O43670 | ZN207_HUMAN | BUB3-interacting and GLEBS motif-containing protein ZNF207 | 0.35501 | 0.07430 |
| P40227 | TCPZ_HUMAN | T-complex protein 1 subunit zeta | 0.35555 | 0.03269 |
| Q9HCC0-2 | MCCB_HUMAN | Isoform 2 of Methylcrotonoyl-CoA carboxylase beta chain, mitochondrial | 0.35599 | -0.09090 |
| P40926 | MDHM_HUMAN | Malate dehydrogenase, mitochondrial | 0.35628 | -0.06048 |
| Q13287 | NMI_HUMAN | N-myc-interactor | 0.3563 | -0.13593 |
| Q15717-2 | ELAV1_HUMAN | Isoform 2 of ELAV-like protein 1 | 0.35658 | 0.04071 |
| Q06323 | PSME1_HUMAN | Proteasome activator complex subunit 1 | 0.35772 | -0.08853 |
| P51114 | FXR1_HUMAN | Fragile X mental retardation syndrome-related protein 1 | 0.35866 | -0.08155 |
| P07737 | PROF1_HUMAN | Profilin-1 | 0.35894 | 0.08586 |
| P14060 | 3BHS1_HUMAN | 3 beta-hydroxysteroid dehydrogenase/Delta 5-->4-isomerase type 1 | 0.36003 | 0.11723 |
| P02042 | HBD_HUMAN | Hemoglobin subunit delta | 0.36041 | -0.23137 |
| Q9BS26 | ERP44_HUMAN | Endoplasmic reticulum resident protein 44 | 0.36131 | -0.03101 |
| Q8IZP0 | ABI1_HUMAN | Abl interactor 1 | 0.36366 | 0.08719 |
| Q96KP4 | CNDP2_HUMAN | Cytosolic non-specific dipeptidase | 0.36535 | -0.08035 |
| P35555 | FBN1_HUMAN | Fibrillin-1 | 0.36614 | 0.20808 |
| Q9H0U4 | RAB1B_HUMAN | Ras-related protein Rab-1B | 0.36641 | 0.01713 |
| P07919 | QCR6_HUMAN | Cytochrome b-c1 complex subunit 6, mitochondrial | 0.36651 | -0.10832 |
| Q8NC51 | PAIRB_HUMAN | Plasminogen activator inhibitor 1 RNA-binding protein | 0.36738 | 0.10421 |
| Q14108 | SCRB2_HUMAN | Lysosome membrane protein 2 | 0.36794 | -0.21448 |
| P49591 | SYSC_HUMAN | Serine--tRNA ligase, cytoplasmic | 0.36921 | 0.08737 |
| P40616 | ARL1_HUMAN | ADP-ribosylation factor-like protein 1 | 0.36933 | 0.15158 |
| Q96CM8 | ACSF2_HUMAN | Acyl-CoA synthetase family member 2, mitochondrial | 0.37178 | -0.45504 |
| Q5T4S7 | UBR4_HUMAN | E3 ubiquitin-protein ligase UBR4 | 0.37334 | 0.07842 |
| Q9Y6W5 | WASF2_HUMAN | Wiskott-Aldrich syndrome protein family member 2 | 0.37423 | 0.07697 |
| Q9BQE5 | APOL2_HUMAN | Apolipoprotein L2 | 0.37452 | -0.06133 |
| Q14498 | RBM39_HUMAN | RNA-binding protein 39 | 0.37532 | 0.06425 |
| Q9BRK5 | CAB45_HUMAN | 45 kDa calcium-binding protein | 0.37697 | 0.11200 |
| Q13510 | ASAH1_HUMAN | Acid ceramidase | 0.37709 | 0.07447 |
| Q15836 | VAMP3_HUMAN | Vesicle-associated membrane protein 3 | 0.3779 | -0.04900 |
| Q06203 | PUR1_HUMAN | Amidophosphoribosyltransferase | 0.37807 | 0.13432 |
| P52566 | GDIR2_HUMAN | Rho GDP-dissociation inhibitor 2 | 0.37808 | 0.17523 |
| P23246 | SFPQ_HUMAN | Splicing factor, proline- and glutamine-rich | 0.37867 | 0.08947 |
| Q96CW1 | AP2M1_HUMAN | AP-2 complex subunit mu | 0.37921 | 0.08774 |
| Q9H8Y8-3 | GORS2_HUMAN | Isoform 3 of Golgi reassembly-stacking protein 2 | 0.38065 | 0.06713 |
| P11279 | LAMP1_HUMAN | Lysosome-associated membrane glycoprotein 1 | 0.38094 | -0.04740 |
| Q9UQE7 | SMC3_HUMAN | Structural maintenance of chromosomes protein 3 | 0.38108 | 0.13211 |
| P61077 | UB2D3_HUMAN | Ubiquitin-conjugating enzyme E2 D3 | 0.38128 | 0.10055 |
| P10809 | CH60_HUMAN | 60 kDa heat shock protein, mitochondrial | 0.38178 | -0.04132 |
| P07954 | FUMH_HUMAN | Fumarate hydratase, mitochondrial | 0.38184 | -0.06103 |
| O94874 | UFL1_HUMAN | E3 UFM1-protein ligase 1 | 0.38223 | 0.10619 |
| Q8NBS9 | TXND5_HUMAN | Thioredoxin domain-containing protein 5 | 0.38351 | -0.10456 |
| Q92542 | NICA_HUMAN | Nicastrin | 0.38432 | -0.11949 |
| P13727 | PRG2_HUMAN | Bone marrow proteoglycan | 0.38437 | -0.10884 |
| P04075 | ALDOA_HUMAN | Fructose-bisphosphate aldolase A | 0.38528 | -0.01913 |
| P31948 | STIP1_HUMAN | Stress-induced-phosphoprotein 1 | 0.38596 | 0.06948 |
| O15498 | YKT6_HUMAN | Synaptobrevin homolog YKT6 | 0.38615 | -0.18321 |
| P31930 | QCR1_HUMAN | Cytochrome b-c1 complex subunit 1, mitochondrial | 0.38626 | -0.07201 |
| P22059 | OSBP1_HUMAN | Oxysterol- inding protein 1 | 0.38688 | 0.02953 |
| P62195 | PRS8_HUMAN | 26S proteasome regulatory subunit 8 | 0.38715 | 0.04188 |
| Q7Z3D6 | GLUCM_HUMAN | D-glutamate cyclase, mitochondrial | 0.38728 | 0.07807 |
| P22087 | FBRL_HUMAN | rRNA 2'- | 0.38758 | 0.14816 |
| Q71UI9 | H2AV_HUMAN | Histone H2A.V | 0.3876 | 0.09834 |
| P11177 | ODPB_HUMAN | Pyruvate dehydrogenase E1 component subunit beta, mitochondrial | 0.38991 | 0.06030 |
| P42224 | STAT1_HUMAN | Signal transducer and activator of transcription 1-alpha/beta | 0.39042 | -0.08090 |
| O14828 | SCAM3_HUMAN | Secretory carrier-associated membrane protein 3 | 0.39059 | 0.02843 |
| O95747 | OXSR1_HUMAN | Serine/threonine-protein kinase | 0.3915 | -0.05651 |
| P11021 | BIP_HUMAN | Endoplasmic reticulum chaperone BiP | 0.39282 | 0.02559 |
| Q9Y365 | STA10_HUMAN | START domain-containing protein 10 | 0.39385 | 0.12440 |
| O95817 | BAG3_HUMAN | BAG family molecular chaperone regulator 3 | 0.39398 | 0.08708 |
| O00410 | IPO5_HUMAN | Importin-5 | 0.39441 | 0.12496 |
| P00387-3 | NB5R3_HUMAN | Isoform 3 of NADH-cytochrome b5 reductase 3 | 0.39558 | 0.04163 |
| P11216 | PYGB_HUMAN | Glycogen phosphorylase, brain form | 0.39615 | 0.10060 |
| Q86TM6 | SYVN1_HUMAN | E3 ubiquitin-protein ligase synoviolin | 0.3962 | 0.02836 |
| O76021 | RL1D1_HUMAN | Ribosomal L1 domain-containing protein 1 | 0.3992 | 0.13732 |
| P04080 | CYTB_HUMAN | Cystatin-B | 0.39927 | 0.12337 |
| P51572 | BAP31_HUMAN | B-cell receptor-associated protein 31 | 0.39933 | -0.03727 |
| P22392 | NDKB_HUMAN | Nucleoside diphosphate kinase B | 0.39959 | 0.03364 |
| Q96A33 | CCD47_HUMAN | Coiled-coil domain-containing protein 47 | 0.39985 | 0.02992 |
| P05387 | RLA2_HUMAN | 60S acidic ribosomal protein P2 | 0.40019 | 0.07280 |
| P09874 | PARP1_HUMAN | Poly [ADP-ribose] polymerase 1 | 0.40098 | 0.10148 |
| Q8IV08 | PLD3_HUMAN | Phospholipase D3 | 0.40341 | 0.16352 |
| Q9BZZ5 | API5_HUMAN | Apoptosis inhibitor 5 | 0.40375 | 0.03389 |
| P15144 | AMPN_HUMAN | Aminopeptidase N | 0.40421 | -0.22327 |
| Q9H4M9 | EHD1_HUMAN | EH domain-containing protein 1 | 0.40552 | 0.04517 |
| P12814 | ACTN1_HUMAN | Alpha-actinin-1 | 0.40753 | -0.01896 |
| P04839 | CY24B_HUMAN | Cytochrome b-245 heavy chain | 0.40762 | 0.11771 |
| O15400 | STX7_HUMAN | Syntaxin-7 | 0.40996 | -0.09676 |
| Q9Y6U3 | ADSV_HUMAN | Adseverin | 0.40999 | -0.15268 |
| O00232 | PSD12_HUMAN | 26S proteasome non-ATPase regulatory subunit 12 | 0.41015 | 0.10398 |
| Q29RF7 | PDS5A_HUMAN | Sister chromatid cohesion protein PDS5 homolog A | 0.41079 | 0.09872 |
| Q9NVJ2 | ARL8B_HUMAN | ADP-ribosylation factor-like protein 8B | 0.4119 | -0.12587 |
| P05166 | PCCB_HUMAN | Propionyl-CoA carboxylase beta chain, mitochondrial | 0.41204 | 0.13958 |
| P21589 | 5NTD_HUMAN | 5'-nucleotidase | 0.41334 | -0.32007 |
| P06733 | ENOA_HUMAN | Alpha-enolase | 0.41342 | 0.02984 |
| O75369 | FLNB_HUMAN | Filamin-B | 0.41356 | 0.03011 |
| Q8N1F7 | NUP93_HUMAN | Nuclear pore complex protein Nup93 | 0.41387 | 0.04790 |
| Q13492 | PICAL_HUMAN | Phosphatidylinositol-binding clathrin assembly protein | 0.41651 | 0.10201 |
| Q15366 | PCBP2_HUMAN | Poly(rC)-binding protein 2 | 0.4172 | 0.03930 |
| P55265 | DSRAD_HUMAN | Double-stranded RNA-specific adenosine deaminase | 0.41847 | 0.12980 |
| Q8N163 | CCAR2_HUMAN | Cell cycle and apoptosis regulator protein 2 | 0.42023 | 0.04755 |
| P04264 | K2C1_HUMAN | Keratin, type II cytoskeletal 1 | 0.42089 | 0.13514 |
| Q92572 | AP3S1_HUMAN | AP-3 complex subunit sigma-1 | 0.4209 | 0.04407 |
| O15347 | HMGB3_HUMAN | High mobility group protein B3 | 0.42091 | 0.05372 |
| O95466 | FMNL1_HUMAN | Formin-like protein 1 | 0.42104 | 0.09771 |
| O15144 | ARPC2_HUMAN | Actin-related protein 2/3 complex subunit 2 | 0.42129 | 0.04639 |
| O43399 | TPD54_HUMAN | Tumor protein D54 | 0.42221 | 0.08262 |
| P28074 | PSB5_HUMAN | Proteasome subunit beta type-5 | 0.42292 | 0.21333 |
| P17858 | PFKAL_HUMAN | ATP-dependent 6-phosphofructokinase, liver type | 0.42425 | 0.04957 |
| P05198 | IF2A_HUMAN | Eukaryotic translation initiation factor 2 subunit 1 | 0.42493 | 0.02463 |
| Q08170 | SRSF4_HUMAN | Serine/arginine-rich splicing factor 4 | 0.42612 | 0.02846 |
| P27816-6 | MAP4_HUMAN | Isoform 6 of Microtubule-associated protein 4 | 0.42711 | 0.04230 |
| P12429 | ANXA3_HUMAN | Annexin A3 | 0.42722 | -0.05838 |
| O75954 | TSN9_HUMAN | Tetraspanin-9 | 0.42934 | -0.06662 |
| P09012 | SNRPA_HUMAN | U1 small nuclear ribonucleoprotein A | 0.43126 | 0.05214 |
| P05362 | ICAM1_HUMAN | Intercellular adhesion molecule 1 | 0.43224 | -0.12253 |
| P10155 | RO60_HUMAN | 60 kDa SS-A/Ro ribonucleoprotein | 0.43256 | 0.04106 |
| P40925 | MDHC_HUMAN | Malate dehydrogenase, cytoplasmic | 0.43292 | 0.01940 |
| Q15056 | IF4H_HUMAN | Eukaryotic translation initiation factor 4H | 0.43327 | 0.14585 |
| P36578 | RL4_HUMAN | 60S ribosomal protein L4 | 0.43544 | 0.19853 |
| Q9H8H3 | MET7A_HUMAN | Methyltransferase-like protein 7A | 0.43568 | 0.08152 |
| P38606 | VATA_HUMAN | V-type proton ATPase catalytic subunit A | 0.43631 | 0.07463 |
| P62701 | RS4X_HUMAN | 40S ribosomal protein S4, X isoform | 0.43709 | 0.04629 |
| P32455 | GBP1_HUMAN | Guanylate-binding protein 1 | 0.43862 | -0.07766 |
| P12956 | XRCC6_HUMAN | X-ray repair cross-complementing protein 6 | 0.43906 | 0.04538 |
| P84095 | RHOG_HUMAN | Rho-related GTP-binding protein RhoG | 0.43957 | -0.17009 |
| Q9NVA2 | SEP11_HUMAN | Septin-11 | 0.44033 | -0.32735 |
| P25787 | PSA2_HUMAN | Proteasome subunit alpha type-2 | 0.44106 | -0.06677 |
| P62277 | RS13_HUMAN | 40S ribosomal protein S13 | 0.44165 | 0.05566 |
| P04004 | VTNC_HUMAN | Vitronectin | 0.44233 | -0.05966 |
| O95298 | NDUC2_HUMAN | NADH dehydrogenase [ubiquinone] 1 subunit C2 | 0.44428 | -0.12359 |
| O00186 | STXB3_HUMAN | Syntaxin-binding protein 3 | 0.44538 | -0.07824 |
| Q9BR76 | COR1B_HUMAN | Coronin-1B | 0.44642 | 0.07364 |
| O94979 | SC31A_HUMAN | Protein transport protein Sec31A | 0.44686 | 0.03282 |
| P10316 | 1A69_HUMAN | HLA class I histocompatibility antigen, A-69 alpha chain | 0.44693 | -0.11093 |
| P31943 | HNRH1_HUMAN | Heterogeneous nuclear ribonucleoprotein H | 0.44826 | 0.09095 |
| P09493-3 | TPM1_HUMAN | Isoform 3 of Tropomyosin alpha-1 chain | 0.44873 | -0.09465 |
| Q9UHD8-7 | SEPT9_HUMAN | Isoform 7 of Septin-9 | 0.44937 | 0.02642 |
| Q8IY17 | PLPL6_HUMAN | Neuropathy target esterase | 0.45212 | 0.05220 |
| Q05519 | SRS11_HUMAN | Serine/arginine-rich splicing factor 11 | 0.45297 | -0.10705 |
| P00966 | ASSY_HUMAN | Argininosuccinate synthase | 0.4531 | 0.09657 |
| P13804 | ETFA_HUMAN | Electron transfer flavoprotein subunit alpha, mitochondrial | 0.45374 | 0.03342 |
| Q92973 | TNPO1_HUMAN | Transportin-1 | 0.45428 | -0.05136 |
| Q9C002 | NMES1_HUMAN | Normal mucosa of esophagus-specific gene 1 protein | 0.45445 | -0.11174 |
| P14550 | AK1A1_HUMAN | Aldo-keto reductase family 1 member A1 | 0.45473 | 0.04374 |
| Q05682-5 | CALD1_HUMAN | Isoform 5 of Caldesmon | 0.45552 | 0.09466 |
| P48739-2 | PIPNB_HUMAN | Isoform 2 of Phosphatidylinositol transfer protein beta isoform | 0.45616 | -0.13187 |
| Q9HC35 | EMAL4_HUMAN | Echinoderm microtubule-associated protein-like 4 | 0.45687 | 0.05002 |
| Q15459 | SF3A1_HUMAN | Splicing factor 3A subunit 1 | 0.45758 | 0.05454 |
| P38117 | ETFB_HUMAN | Electron transfer flavoprotein subunit beta | 0.45792 | 0.13033 |
| P00915 | CAH1_HUMAN | Carbonic anhydrase 1 | 0.45812 | -0.18431 |
| P60660 | MYL6_HUMAN | Myosin light polypeptide 6 | 0.45886 | 0.09541 |
| Q9UHQ9 | NB5R1_HUMAN | NADH-cytochrome b5 reductase 1 | 0.45975 | 0.08908 |
| Q86UP2 | KTN1_HUMAN | Kinectin | 0.46347 | 0.06562 |
| P61289 | PSME3_HUMAN | Proteasome activator complex subunit 3 | 0.46485 | -0.04260 |
| Q96NB2 | SFXN2_HUMAN | Sideroflexin-2 | 0.46495 | -0.06386 |
| P49327 | FAS_HUMAN | Fatty acid synthase | 0.46508 | 0.07157 |
| Q14624-4 | ITIH4_HUMAN | Isoform 4 of Inter-alpha-trypsin inhibitor heavy chain H4 | 0.466 | -0.12335 |
| Q9NYL9 | TMOD3_HUMAN | Tropomodulin-3 | 0.46649 | 0.08714 |
| P46937 | YAP1_HUMAN | Transcriptional coactivator YAP1 | 0.47005 | 0.11381 |
| Q96HE7 | ERO1A_HUMAN | ER1-like protein alpha | 0.4711 | -0.03869 |
| P61626 | LYSC_HUMAN | Lysozyme C | 0.47456 | 0.19096 |
| P59998 | ARPC4_HUMAN | Actin-related protein 2/3 complex subunit 4 | 0.47494 | 0.02718 |
| Q6NUK1 | SCMC1_HUMAN | Calcium-binding mitochondrial carrier protein SCaMC-1 | 0.47506 | -0.06530 |
| Q9UNM6 | PSD13_HUMAN | 26S proteasome non-ATPase regulatory subunit 13 | 0.47622 | -0.06329 |
| P21291 | CSRP1_HUMAN | Cysteine and glycine-rich protein 1 | 0.47649 | 0.03696 |
| Q7Z6Z7 | HUWE1_HUMAN | E3 ubiquitin-protein ligase HUWE1 | 0.47684 | 0.14864 |
| Q16836 | HCDH_HUMAN | Hydroxyacyl-coenzyme A dehydrogenase, mitochondrial | 0.47799 | 0.07794 |
| O15143 | ARC1B_HUMAN | Actin-related protein 2/3 complex subunit 1B | 0.47871 | -0.09172 |
| P31946-2 | 1433B_HUMAN | Isoform Short of 14-3-3 protein beta/alpha | 0.47899 | 0.05455 |
| O75400 | PR40A_HUMAN | Pre-mRNA-processing factor 40 homolog A | 0.47927 | 0.01875 |
| O95373 | IPO7_HUMAN | Importin-7 | 0.47931 | 0.03950 |
| O43390 | HNRPR_HUMAN | Heterogeneous nuclear ribonucleoprotein R | 0.48032 | 0.07427 |
| O00567 | NOP56_HUMAN | Nucleolar protein 56 | 0.48037 | 0.07029 |
| P25789 | PSA4_HUMAN | Proteasome subunit alpha type-4 | 0.48119 | 0.05932 |
| P15924 | DESP_HUMAN | Desmoplakin | 0.48171 | 0.07016 |
| P55072 | TERA_HUMAN | Transitional endoplasmic reticulum ATPase | 0.4838 | 0.01803 |
| P23141 | EST1_HUMAN | Liver carboxylesterase 1 | 0.48408 | -0.15506 |
| Q92734 | TFG_HUMAN | Protein TFG | 0.48497 | 0.07701 |
| Q02878 | RL6_HUMAN | 60S ribosomal protein L6 | 0.48516 | 0.08755 |
| P26639 | SYTC_HUMAN | Threonine--tRNA ligase, cytoplasmic | 0.48521 | -0.30003 |
| Q14956 | GPNMB_HUMAN | Transmembrane glycoprotein NMB | 0.48592 | 0.16579 |
| O15371 | EIF3D_HUMAN | Eukaryotic translation initiation factor 3 subunit D | 0.48635 | 0.06871 |
| Q9BT09 | CNPY3_HUMAN | Protein canopy homolog 3 | 0.48683 | 0.07579 |
| P68104 | EF1A1_HUMAN | Elongation factor 1-alpha 1 | 0.49171 | 0.02475 |
| Q96AG4 | LRC59_HUMAN | Leucine-rich repeat-containing protein 59 | 0.49386 | 0.06582 |
| Q8WWP7 | GIMA1_HUMAN | GTPase IMAP family member 1 | 0.49427 | 0.13825 |
| O15533 | TPSN_HUMAN | Tapasin | 0.49484 | -0.05391 |
| P36957 | ODO2_HUMAN | Dihydrolipoyllysine-residue succinyltransferase component of 2-oxoglutarate dehydrogenase complex, mitochondrial | 0.4968 | -0.05589 |
| P08670 | VIME_HUMAN | Vimentin | 0.4972 | 0.08013 |
| P07602 | SAP_HUMAN | Prosaposin | 0.4974 | 0.10046 |
| P60981 | DEST_HUMAN | Destrin | 0.4974 | -0.09525 |
| Q96HD1 | CREL1_HUMAN | Cysteine-rich with EGF-like domain protein 1 | 0.49989 | -0.07850 |
| P07814 | SYEP_HUMAN | Bifunctional glutamate/proline--tRNA ligase | 0.50087 | -0.02079 |
| P82979 | SARNP_HUMAN | SAP domain-containing ribonucleoprotein | 0.50118 | 0.13384 |
| O75695 | XRP2_HUMAN | Protein XRP2 | 0.50159 | 0.03073 |
| P30046 | DOPD_HUMAN | D-dopachrome decarboxylase | 0.50203 | -0.09736 |
| P43304 | GPDM_HUMAN | Glycerol-3-phosphate dehydrogenase, mitochondrial | 0.50582 | -0.06564 |
| P16070 | CD44_HUMAN | CD44 antigen | 0.50723 | 0.09947 |
| P22234 | PUR6_HUMAN | Multifunctional protein ADE2 | 0.50823 | 0.03660 |
| Q03252 | LMNB2_HUMAN | Lamin-B2 | 0.50847 | 0.03479 |
| O75489 | NDUS3_HUMAN | NADH dehydrogenase [ubiquinone] iron-sulfur protein 3, mitochondrial | 0.50922 | -0.04382 |
| P43686 | PRS6B_HUMAN | 26S proteasome regulatory subunit 6B | 0.50955 | 0.03306 |
| P49821 | NDUV1_HUMAN | NADH dehydrogenase [ubiquinone] flavoprotein 1, mitochondrial | 0.51085 | -0.05619 |
| P19878 | NCF2_HUMAN | Neutrophil cytosol factor 2 | 0.51164 | 0.09451 |
| P21283 | VATC1_HUMAN | V-type proton ATPase subunit C 1 | 0.51204 | 0.07461 |
| Q16576 | RBBP7_HUMAN | Histone-binding protein RBBP7 | 0.51432 | 0.04140 |
| P36776 | LONM_HUMAN | Lon protease homolog, mitochondrial | 0.51533 | 0.05133 |
| O43617 | TPPC3_HUMAN | Trafficking protein particle complex subunit 3 | 0.51609 | -0.05584 |
| O75352 | MPU1_HUMAN | Mannose-P-dolichol utilization defect 1 protein | 0.51715 | -0.09286 |
| O95782 | AP2A1_HUMAN | AP-2 complex subunit alpha-1 | 0.51746 | 0.04410 |
| Q9UNF0 | PACN2_HUMAN | Protein kinase C and casein kinase substrate in neurons protein 2 | 0.51976 | 0.06925 |
| P48047 | ATPO_HUMAN | ATP synthase subunit, mitochondrial | 0.52196 | -0.04730 |
| P27824 | CALX_HUMAN | Calnexin | 0.52224 | 0.02164 |
| Q00059 | TFAM_HUMAN | Transcription factor A, mitochondrial | 0.52233 | -0.07906 |
| O00217 | NDUS8_HUMAN | NADH dehydrogenase [ubiquinone] iron-sulfur protein 8, mitochondrial | 0.52286 | 0.07868 |
| Q52LJ0 | FA98B_HUMAN | Protein FAM98B | 0.52304 | 0.06431 |
| Q9C0C2 | TB182_HUMAN | 182 kDa tankyrase-1-binding protein | 0.52472 | 0.08844 |
| P35527 | K1C9_HUMAN | Keratin, type I cytoskeletal 9 | 0.52508 | -0.03866 |
| P14598 | NCF1_HUMAN | Neutrophil cytosol factor 1 | 0.52587 | -0.11380 |
| P46783 | RS10_HUMAN | 40S ribosomal protein S10 | 0.52694 | 0.06695 |
| Q16630-2 | CPSF6_HUMAN | Isoform 2 of Cleavage and polyadenylation specificity factor subunit 6 | 0.52694 | 0.06516 |
| Q13724 | MOGS_HUMAN | Mannosyl-oligosaccharide glucosidase | 0.52697 | -0.07532 |
| Q9NP72 | RAB18_HUMAN | Ras-related protein Rab-18 | 0.52706 | -0.10562 |
| Q9BRF8 | CPPED_HUMAN | Serine/threonine-protein phosphatase CPPED1 | 0.52741 | 0.03196 |
| Q16698 | DECR_HUMAN | 2,4-dienoyl-CoA reductase, mitochondrial | 0.52756 | -0.10597 |
| Q9NX02 | NALP2_HUMAN | NACHT, LRR and PYD domains-containing protein 2 | 0.52758 | -0.09979 |
| P45880 | VDAC2_HUMAN | Voltage-dependent anion-selective channel protein 2 | 0.52834 | 0.02828 |
| O14735 | CDIPT_HUMAN | CDP-diacylglycerol--inositol 3-phosphatidyltransferase | 0.52935 | -0.03450 |
| Q15121 | PEA15_HUMAN | Astrocytic phosphoprotein PEA-15 | 0.53015 | 0.07169 |
| P30101 | PDIA3_HUMAN | Protein disulfide-isomerase A3 | 0.53018 | 0.02116 |
| P22314 | UBA1_HUMAN | Ubiquitin-like modifier-activating enzyme 1 | 0.53105 | 0.02858 |
| Q01082 | SPTB2_HUMAN | Spectrin beta chain, non-erythrocytic 1 | 0.5315 | -0.02579 |
| Q08379 | GOGA2_HUMAN | Golgin subfamily A member 2 | 0.53179 | 0.07098 |
| Q9Y678 | COPG1_HUMAN | Coatomer subunit gamma-1 | 0.53304 | 0.03903 |
| Q9H4A3-7 | WNK1_HUMAN | Isoform 6 of Serine/threonine-protein kinase WNK1 | 0.53361 | 0.10494 |
| O43852-3 | CALU_HUMAN | Isoform 3 of Calumenin | 0.53377 | 0.03671 |
| P04843 | RPN1_HUMAN | Dolichyl-diphosphooligosaccharide--protein glycosyltransferase subunit 1 | 0.53413 | -0.01403 |
| P01903 | DRA_HUMAN | HLA class II histocompatibility antigen, DR alpha chain | 0.53536 | -0.12077 |
| P12236 | ADT3_HUMAN | ADP/ATP translocase 3 | 0.53819 | -0.02647 |
| Q9UQ35 | SRRM2_HUMAN | Serine/arginine repetitive matrix protein 2 | 0.53976 | 0.08472 |
| P62826 | RAN_HUMAN | GTP-binding nuclear protein Ran | 0.54102 | 0.02188 |
| P29401 | TKT_HUMAN | Transketolase | 0.54225 | 0.02644 |
| Q9Y2J2 | E41L3_HUMAN | Band 4.1-like protein 3 | 0.54507 | -0.02653 |
| Q0P6H9 | TMM62_HUMAN | Transmembrane protein 62 | 0.54609 | -0.09830 |
| Q92688 | AN32B_HUMAN | Acidic leucine-rich nuclear phosphoprotein 32 family member B | 0.54653 | 0.02868 |
| P62258 | 1433E_HUMAN | 14-3-3 protein epsilon | 0.54701 | 0.02864 |
| Q9NR28 | DBLOH_HUMAN | Diablo homolog, mitochondrial | 0.54701 | -0.02864 |
| Q9NRP0 | OSTC_HUMAN | Oligosaccharyltransferase complex subunit | 0.548 | -0.02994 |
| Q13185 | CBX3_HUMAN | Chromobox protein homolog 3 | 0.54818 | 0.06645 |
| P01111 | RASN_HUMAN | GTPase NRas | 0.54843 | 0.01878 |
| P30566 | PUR8_HUMAN | Adenylosuccinate lyase | 0.54876 | -0.04098 |
| O94973 | AP2A2_HUMAN | AP-2 complex subunit alpha-2 | 0.54901 | 0.02536 |
| Q12797 | ASPH_HUMAN | Aspartyl/asparaginyl beta-hydroxylase | 0.54909 | 0.02020 |
| Q9H8S9 | MOB1A_HUMAN | MOB kinase acivator 1A | 0.55009 | -0.06157 |
| Q03519 | TAP2_HUMAN | Antigen peptide transporter 2 | 0.5503 | -0.04434 |
| O14684 | PTGES_HUMAN | Prostaglandin E synthase | 0.55041 | -0.03383 |
| P67936 | TPM4_HUMAN | Tropomyosin alpha-4 chain | 0.55098 | -0.03009 |
| P84077 | ARF1_HUMAN | ADP-ribosylation factor 1 | 0.55358 | -0.05201 |
| Q86Y82 | STX12_HUMAN | Syntaxin-12 | 0.55428 | -0.03756 |
| Q99436 | PSB7_HUMAN | Proteasome subunit beta type-7 | 0.55442 | -0.03347 |
| O00442 | RTCA_HUMAN | RNA 3'-terminal phosphate cyclase | 0.55612 | 0.06170 |
| P11215 | ITAM_HUMAN | Integrin alpha-M | 0.55748 | 0.08347 |
| P60953 | CDC42_HUMAN | Cell division control protein 42 homolog | 0.55797 | 0.02779 |
| O60701 | UGDH_HUMAN | UDP-glucose 6-dehydrogenase | 0.55868 | 0.03316 |
| P33241 | LSP1_HUMAN | Lymphocyte-specific protein 1 | 0.56089 | 0.17586 |
| P40939 | ECHA_HUMAN | Trifunctional enzyme subunit alpha, mitochondrial | 0.56109 | -0.05967 |
| O75964 | ATP5L_HUMAN | ATP synthase subunit g, mitochondrial | 0.56111 | 0.04936 |
| P09497-2 | CLCB_HUMAN | Isoform Non-brain of Clathrin light chain B | 0.5624 | 0.03323 |
| P49419 | AL7A1_HUMAN | Alpha-aminoadipic semialdehyde dehydrogenase | 0.5625 | 0.04239 |
| Q99442 | SEC62_HUMAN | Translocation protein SEC62 | 0.56315 | 0.04543 |
| Q06787 | FMR1_HUMAN | Synaptic functional regulator FMR1 | 0.56417 | 0.02115 |
| O96008 | TOM40_HUMAN | Mitochondrial import receptor subunit T | 0.56417 | -0.06296 |
| Q96AE4 | FUBP1_HUMAN | Far upstream element-binding protein 1 | 0.56577 | 0.07944 |
| O00429 | DNM1L_HUMAN | Dynamin-1-like protein | 0.56639 | 0.02860 |
| P20742 | PZP_HUMAN | Pregnancy zone protein | 0.56708 | -0.06040 |
| Q16706 | MA2A1_HUMAN | Alpha-mannosidase 2 | 0.56747 | 0.08024 |
| Q86UX7 | URP2_HUMAN | Fermitin family homolog 3 | 0.56832 | 0.10272 |
| O60664 | PLIN3_HUMAN | Perilipin-3 | 0.56932 | -0.02663 |
| P50552 | VASP_HUMAN | Vasodilator-stimulated phosphoprotein | 0.56933 | 0.06204 |
| Q13561 | DCTN2_HUMAN | Dynactin subunit 2 | 0.56942 | 0.04331 |
| P48735 | IDHP_HUMAN | Isocitrate dehydrogenase [NADP], mitochondrial | 0.57058 | -0.04390 |
| P06396-2 | GELS_HUMAN | Isoform 2 of Gelsolin | 0.57152 | 0.06440 |
| Q01105 | SET_HUMAN | Protein SET | 0.5721 | -0.04872 |
| P56385 | ATP5I_HUMAN | ATP synthase subunit e, mitochondrial | 0.57216 | 0.10956 |
| P26583 | HMGB2_HUMAN | High mobility group protein B2 | 0.57256 | 0.04668 |
| Q9Y5Z4 | HEBP2_HUMAN | Heme-binding protein 2 | 0.57418 | -0.04179 |
| O75874 | IDHC_HUMAN | Isocitrate dehydrogenase [NADP] cytoplasmic | 0.57435 | 0.03773 |
| P28838 | AMPL_HUMAN | Cytosol aminopeptidase | 0.57452 | -0.06925 |
| P02751 | FINC_HUMAN | Fibronectin | 0.57472 | 0.04296 |
| P62070 | RRAS2_HUMAN | Ras-related protein R-Ras2 | 0.5776 | 0.06674 |
| P47755 | CAZA2_HUMAN | F-actin-capping protein subunit alpha-2 | 0.57801 | 0.02883 |
| Q15008 | PSMD6_HUMAN | 26S proteasome non-ATPase regulatory subunit 6 | 0.57835 | 0.03431 |
| Q15165 | PON2_HUMAN | Serum paraoxonase/arylesterase 2 | 0.5812 | -0.04432 |
| P47985 | UCRI_HUMAN | Cytochrome b-c1 complex subunit Rieske, mitochondrial | 0.58446 | -0.12693 |
| P50991 | TCPD_HUMAN | T-complex protein 1 subunit delta | 0.5857 | 0.07457 |
| P60033 | CD81_HUMAN | CD81 antigen | 0.58616 | -0.06476 |
| P43034 | LIS1_HUMAN | Platelet-activating factor acetylhydrolase IB subunit alpha | 0.58623 | -0.03501 |
| O60313 | OPA1_HUMAN | Dynamin-like 120 kDa protein, mitochondrial | 0.58648 | -0.05808 |
| P30740 | ILEU_HUMAN | Leukocyte elastase inhibitor | 0.58709 | -0.06794 |
| P05164 | PERM_HUMAN | Myeloperoxidase | 0.58745 | -0.18493 |
| Q01130 | SRSF2_HUMAN | Serine/arginine-rich splicing factor 2 | 0.58796 | 0.02032 |
| Q3LXA3 | TKFC_HUMAN | Triokinase/FMN cyclase | 0.58932 | 0.04918 |
| Q99735 | MGST2_HUMAN | Microsomal glutathione S-transferase 2 | 0.58943 | -0.05565 |
| P62899 | RL31_HUMAN | 60S ribosomal protein L31 | 0.58975 | 0.04699 |
| Q9HB71 | CYBP_HUMAN | Calcyclin-binding protein | 0.58987 | -0.02107 |
| P40121 | CAPG_HUMAN | Macrophage-capping protein | 0.59001 | 0.08182 |
| P30040 | ERP29_HUMAN | Endoplasmic reticulum resident protein 29 | 0.59038 | -0.05840 |
| P52209 | 6PGD_HUMAN | 6-phosphogluconate dehydrogenase, decarboxylating | 0.59057 | 0.04865 |
| P61160 | ARP2_HUMAN | Actin-related protein 2 | 0.59062 | 0.03428 |
| P62244 | RS15A_HUMAN | 40S ribosomal protein S15a | 0.59249 | 0.08741 |
| P21333 | FLNA_HUMAN | Filamin-A | 0.59354 | 0.06367 |
| Q9Y265 | RUVB1_HUMAN | RuvB-like 1 | 0.59536 | -0.07782 |
| O95833 | CLIC3_HUMAN | Chloride intracellular channel protein 3 | 0.59669 | -0.04639 |
| P40306 | PSB10_HUMAN | Proteasome subunit beta type-10 | 0.5972 | -0.07897 |
| Q15424-4 | SAFB1_HUMAN | Isoform 4 of Scaffold attachment factor B1 | 0.5976 | 0.03006 |
| P15311 | EZRI_HUMAN | Ezrin | 0.59828 | 0.03800 |
| P50990 | TCPQ_HUMAN | T-complex protein 1 subunit theta | 0.5986 | 0.07427 |
| P48556 | PSMD8_HUMAN | 26S proteasome non-ATPase regulatory subunit 8 | 0.59879 | -0.03952 |
| P55010 | IF5_HUMAN | Eukaryotic translation initiation factor 5 | 0.59882 | 0.04166 |
| Q969V3 | NCLN_HUMAN | Nicalin | 0.59891 | -0.12072 |
| P63208 | SKP1_HUMAN | S-phase kinase-associated protein 1 | 0.59962 | -0.05238 |
| Q6UXV4 | MIC27_HUMAN | MIC | 0.60012 | -0.05914 |
| O00273 | DFFA_HUMAN | DNA fragmentation factor subunit alpha | 0.60068 | 0.08830 |
| Q9HA64 | KT3K_HUMAN | Ketosamine-3-kinase | 0.60091 | 0.06137 |
| Q7L2H7 | EIF3M_HUMAN | Eukaryotic translation initiation factor 3 subunit M | 0.60132 | 0.04404 |
| P09525 | ANXA4_HUMAN | Annexin A4 | 0.60145 | -0.03015 |
| O43169 | CYB5B_HUMAN | Cytochrome b5 type B | 0.60218 | -0.04638 |
| Q9H2G2 | SLK_HUMAN | STE20-like serine/threonine-protein kinase | 0.6029 | -0.04899 |
| Q14974 | IMB1_HUMAN | Importin subunit beta-1 | 0.60298 | 0.05941 |
| Q71DI3 | H32_HUMAN | Histone H3.2 | 0.60679 | 0.10467 |
| P30626 | SORCN_HUMAN | Sorcin | 0.60736 | -0.03973 |
| P00441 | SODC_HUMAN | Superoxide dismutase [Cu-Zn] | 0.6082 | -0.08640 |
| Q9NT62 | ATG3_HUMAN | Ubiquitin-like-conjugating enzyme ATG3 | 0.60837 | -0.03730 |
| P60174 | TPIS_HUMAN | Triosephosphate isomerase | 0.60884 | 0.01763 |
| P21281 | VATB2_HUMAN | V-type proton ATPase subunit B, brain isoform | 0.60885 | 0.05133 |
| Q92599-2 | SEPT8_HUMAN | Isoform 2 of Septin-8 | 0.60988 | 0.02837 |
| Q13423 | NNTM_HUMAN | NAD(P) transhydrogenase, mitochondrial | 0.61011 | 0.02730 |
| Q9H444 | CHM4B_HUMAN | Charged multivesicular body protein 4b | 0.61132 | 0.03275 |
| P19105 | ML12A_HUMAN | Myosin regulatory light chain 12A | 0.61146 | 0.05032 |
| P47756-2 | CAPZB_HUMAN | Isoform 2 of F-actin-capping protein subunit beta | 0.61164 | 0.03527 |
| P43490 | NAMPT_HUMAN | Nicotinamide phosphoribosyltransferase | 0.61226 | 0.05421 |
| P12268 | IMDH2_HUMAN | Inosine-5'-monophosphate dehydrogenase 2 | 0.61289 | 0.02234 |
| P20810-9 | ICAL_HUMAN | Isoform 9 of Calpastatin | 0.61445 | -0.02322 |
| P43487 | RANG_HUMAN | Ran-specific GTPase-activating protein | 0.61497 | 0.07765 |
| P13674-3 | P4HA1_HUMAN | Isoform 3 of Prolyl 4-hydroxylase subunit alpha-1 | 0.61526 | 0.07288 |
| Q14258 | TRI25_HUMAN | E3 ubiquitin/ISG15 ligase TRIM25 | 0.61812 | -0.03315 |
| O75955 | FLOT1_HUMAN | Flotillin-1 | 0.61947 | 0.03003 |
| P08727 | K1C19_HUMAN | Keratin, type I cytoskeletal 19 | 0.61968 | 0.03147 |
| Q13126 | MTAP_HUMAN | S-methyl-5'-thioadenosine phosphorylase | 0.6206 | 0.08024 |
| O15145 | ARPC3_HUMAN | Actin-related protein 2/3 complex subunit 3 | 0.62114 | 0.05760 |
| P51659 | DHB4_HUMAN | Peroxisomal multifunctional enzyme type 2 | 0.62169 | 0.04525 |
| Q969E2 | SCAM4_HUMAN | Secretory carrier-associated membrane protein 4 | 0.62226 | -0.06132 |
| P05783 | K1C18_HUMAN | Keratin, type I cytoskeletal 18 | 0.62274 | 0.02084 |
| Q9Y3Z3 | SAMH1_HUMAN | Deoxynucleoside triphosphate triphosphohydrolase SAMHD1 | 0.6234 | 0.07257 |
| P07237 | PDIA1_HUMAN | Protein disulfide-isomerase | 0.62421 | 0.01458 |
| Q9Y584 | TIM22_HUMAN | Mitochondrial import inner membrane translocase subunit Tim22 | 0.62543 | -0.10945 |
| P31949 | S10AB_HUMAN | Protein S100-A11 | 0.62809 | -0.07323 |
| P30048 | PRDX3_HUMAN | Thioredoxin-dependent peroxide reductase, mitochondrial | 0.62874 | -0.06025 |
| P11387 | TOP1_HUMAN | DNA topoisomerase 1 | 0.63033 | 0.04471 |
| P55795 | HNRH2_HUMAN | Heterogeneous nuclear ribonucleoprotein H2 | 0.63051 | 0.04009 |
| Q04917 | 1433F_HUMAN | 14-3-3 protein eta | 0.63177 | -0.02555 |
| Q5VT79 | AXA81_HUMAN | Annexin A8-like protein 1 | 0.63286 | -0.05423 |
| Q8TD55 | PKHO2_HUMAN | Pleckstrin homology domain-containing family | 0.6336 | 0.05217 |
| Q9UNS2 | CSN3_HUMAN | COP9 signalosome complex subunit | 0.63628 | -0.07924 |
| P35998 | PRS7_HUMAN | 26S proteasome regulatory subunit 7 | 0.63634 | 0.06474 |
| Q7L5N7 | PCAT2_HUMAN | Lysophosphatidylcholine acyltransferase 2 | 0.63737 | 0.08969 |
| O60829 | PAGE4_HUMAN | P antigen family member 4 | 0.63802 | -0.05540 |
| P53634 | CATC_HUMAN | Dipeptidyl peptidase 1 | 0.63852 | 0.07102 |
| P35579 | MYH9_HUMAN | Myosin-9 | 0.63859 | -0.03151 |
| P35241 | RADI_HUMAN | Radixin | 0.63903 | 0.03417 |
| P29966 | MARCS_HUMAN | Myristoylated alanine-rich C-kinase substrate | 0.64082 | -0.04320 |
| P13645 | K1C10_HUMAN | Keratin, type I cytoskeletal 10 | 0.64178 | 0.04120 |
| P13647 | K2C5_HUMAN | Keratin, type II cytoskeletal 5 | 0.64345 | 0.04784 |
| Q99571 | P2RX4_HUMAN | P2X purinoceptor 4 | 0.64487 | -0.06146 |
| Q8TE68 | ES8L1_HUMAN | Epidermal growth factor receptor kinase substrate 8-like protein 1 | 0.64652 | -0.06275 |
| Q9BTV4 | TMM43_HUMAN | Transmembrane protein 43 | 0.64981 | -0.08787 |
| Q9Y490 | TLN1_HUMAN | Talin-1 | 0.65061 | 0.03640 |
| Q14103 | HNRPD_HUMAN | Heterogeneous nuclear ribonucleoprotein D0 | 0.65177 | 0.09449 |
| Q567U6 | CCD93_HUMAN | Coiled-coil domain-containing protein 93 | 0.65331 | -0.06529 |
| P35908 | K22E_HUMAN | Keratin, type II cytoskeletal 2 epidermal | 0.65596 | 0.04409 |
| P50895 | BCAM_HUMAN | Basal cell adhesion molecule | 0.6575 | -0.04162 |
| P05023 | AT1A1_HUMAN | Sodium/potassium-transporting ATPase subunit alpha-1 | 0.65814 | -0.02099 |
| P15153 | RAC2_HUMAN | Ras-related C3 botulinum toxin substrate 2 | 0.65903 | -0.08912 |
| P52788 | SPSY_HUMAN | Spermine synthase | 0.66052 | -0.04798 |
| Q07960 | RHG01_HUMAN | Rho GTPase-activating protein 1 | 0.66094 | -0.02110 |
| P14866 | HNRPL_HUMAN | Heterogeneous nuclear ribonucleoprotein L | 0.66147 | -0.05413 |
| Q14978 | NOLC1_HUMAN | Nucleolar and coiled-body phosphoprotein 1 | 0.66272 | 0.08816 |
| P08133 | ANXA6_HUMAN | Annexin A6 | 0.66567 | -0.02193 |
| P61106 | RAB14_HUMAN | Ras-related protein Rab-14 | 0.66594 | -0.03660 |
| O75915 | PRAF3_HUMAN | PRA1 family protein 3 | 0.66618 | -0.03033 |
| Q06033 | ITIH3_HUMAN | Inter-alpha-trypsin inhibitor heavy chain H3 | 0.66965 | -0.05301 |
| O75223 | GGCT_HUMAN | Gamma-glutamylcyclotransferase | 0.67031 | 0.03767 |
| Q8TCT9 | HM13_HUMAN | Minor histocompatibility antigen H13 | 0.67401 | 0.08273 |
| Q15286 | RAB35_HUMAN | Ras-related protein Rab-35 | 0.67401 | -0.04262 |
| P31146 | COR1A_HUMAN | Coronin-1A | 0.67556 | -0.06212 |
| P62318 | SMD3_HUMAN | Small nuclear ribonucleoprotein Sm D3 | 0.67656 | 0.02739 |
| P39019 | RS19_HUMAN | 40S ribosomal protein S19 | 0.67777 | 0.02744 |
| P56537 | IF6_HUMAN | Eukaryotic translation initiation factor 6 | 0.67926 | 0.02172 |
| Q16134 | ETFD_HUMAN | Electron transfer flavoprotein-ubiquinone oxidoreductase, mitochondrial | 0.68055 | -0.02375 |
| P23229-9 | ITA6_HUMAN | Isoform 9 of Integrin alpha-6 | 0.68244 | 0.01858 |
| P61158 | ARP3_HUMAN | Actin-related protein 3 | 0.68262 | 0.01880 |
| P19367 | HXK1_HUMAN | Hexokinase-1 | 0.68342 | -0.01477 |
| Q9H5X1 | CIA2A_HUMAN | Cytosolic iron-sulfur assembly component 2A | 0.68449 | 0.04156 |
| P23528 | COF1_HUMAN | Cofilin-1 | 0.68677 | 0.04421 |
| Q15149-4 | PLEC_HUMAN | Isoform 4 of Plectin | 0.68681 | -0.03452 |
| Q9Y6N5 | SQOR_HUMAN | Sulfide:quinone oxidoreductase, mitochondrial | 0.68708 | -0.06152 |
| P14061 | DHB1_HUMAN | Estradiol 17-beta-dehydrogenase 1 | 0.68847 | -0.03731 |
| P55036 | PSMD4_HUMAN | 26S proteasome non-ATPase regulatory subunit 4 | 0.68872 | 0.04204 |
| P02794 | FRIH_HUMAN | Ferritin heavy chain | 0.69045 | 0.06986 |
| P07900 | HS90A_HUMAN | Heat shock protein HSP 90-alpha | 0.69088 | -0.00897 |
| Q9P2E9 | RRBP1_HUMAN | Ribosome-binding protein 1 | 0.69107 | 0.01345 |
| P19823 | ITIH2_HUMAN | Inter-alpha-trypsin inhibitor heavy chain H2 | 0.69202 | -0.08667 |
| Q8NC56 | LEMD2_HUMAN | LEM domain-containing protein 2 | 0.69384 | -0.01675 |
| P21980 | TGM2_HUMAN | Protein-glutamine gamma-glutamyltransferase 2 | 0.6947 | -0.05439 |
| P13473 | LAMP2_HUMAN | Lysosome-associated membrane glycoprotein 2 | 0.69563 | 0.02600 |
| Q13409 | DC1I2_HUMAN | Cytoplasmic dynein 1 intermediate chain 2 | 0.69607 | -0.05481 |
| Q9ULA0 | DNPEP_HUMAN | Aspartyl aminopeptidase | 0.69712 | -0.04040 |
| P20645 | MPRD_HUMAN | Cation-dependent mannose-6-phosphate receptor | 0.69908 | 0.03158 |
| P11142 | HSP7C_HUMAN | Heat shock cognate 71 kDa protein | 0.69964 | 0.02134 |
| P08729 | K2C7_HUMAN | Keratin, type II cytoskeletal 7 | 0.7016 | 0.03147 |
| O43143 | DHX15_HUMAN | Pre-mRNA-splicing factor ATP-dependent RNA helicase DHX15 | 0.70251 | 0.01874 |
| P31040 | SDHA_HUMAN | Succinate dehydrogenase [ubiquinone] flavoprotein subunit, mitochondrial | 0.70516 | -0.02486 |
| Q63HN8 | RN213_HUMAN | E3 ubiquitin-protein ligase RNF213 | 0.70539 | 0.06208 |
| P06454 | PTMA_HUMAN | Prothymosin alpha | 0.70634 | -0.03016 |
| P78417 | GSTO1_HUMAN | Glutathione S-transferase omega-1 | 0.70645 | 0.03063 |
| Q6P9B6 | MEAK7_HUMAN | MTOR- associated protein MEAK7 | 0.70739 | 0.04756 |
| P20340 | RAB6A_HUMAN | Ras-related protein Rab-6A | 0.70871 | 0.02123 |
| Q14669 | TRIPC_HUMAN | E3 ubiquitin-protein ligase TRIP12 | 0.70927 | 0.02537 |
| Q14554 | PDIA5_HUMAN | Protein disulfide-isomerase A5 | 0.70933 | 0.01643 |
| P39687 | AN32A_HUMAN | Acidic leucine-rich nuclear phosphoprotein 32 family member A | 0.71029 | 0.01749 |
| P10253 | LYAG_HUMAN | Lysosomal alpha-glucosidase | 0.71084 | 0.05738 |
| Q9NR31 | SAR1A_HUMAN | GTP-binding protein SAR1a | 0.71198 | 0.02424 |
| O14975 | S27A2_HUMAN | Very long-chain acyl-CoA synthetase | 0.71259 | -0.06761 |
| O43707 | ACTN4_HUMAN | Alpha-actinin-4 | 0.71272 | -0.01950 |
| Q92882 | OSTF1_HUMAN | Osteoclast- stimulating factor 1 | 0.71276 | -0.04808 |
| Q1KMD3 | HNRL2_HUMAN | Heterogeneous nuclear ribonucleoprotein U-like protein 2 | 0.71416 | -0.04360 |
| Q53SF7 | COBL1_HUMAN | Cordon-bleu protein-like 1 | 0.71589 | -0.04803 |
| Q8NBN7 | RDH13_HUMAN | Retinol dehydrogenase 13 | 0.71616 | 0.04085 |
| P00491 | PNPH_HUMAN | Purine nucleoside phosphorylase | 0.71752 | -0.01589 |
| Q12792 | TWF1_HUMAN | Twinfilin-1 | 0.71755 | 0.02952 |
| P06748 | NPM_HUMAN | Nucleophosmin | 0.71892 | 0.04740 |
| Q8IZ83 | A16A1_HUMAN | Aldehyde dehydrogenase family 16 member A1 | 0.72003 | 0.04488 |
| O43684 | BUB3_HUMAN | Mitotic checkpoint protein BUB3 | 0.72034 | -0.02732 |
| P11586 | C1TC_HUMAN | C-1-tetrahydrofolate synthase, cytoplasmic | 0.72044 | 0.02346 |
| P21912 | SDHB_HUMAN | Succinate dehydrogenase [ubiquinone] iron-sulfur subunit, mitochondrial | 0.72252 | -0.03093 |
| Q9UHB6 | LIMA1_HUMAN | LIM domain and actin-binding protein 1 | 0.7243 | 0.05349 |
| P41218 | MNDA_HUMAN | Myeloid cell nuclear differentiation antigen | 0.72443 | -0.05329 |
| Q9Y2B0 | CNPY2_HUMAN | Protein canopy homolog 2 | 0.72469 | -0.02208 |
| P41250 | GARS_HUMAN | Glycine--tRNA ligase | 0.72601 | -0.01330 |
| P53396 | ACLY_HUMAN | ATP-citrate synthase | 0.72776 | -0.02081 |
| Q9BTT0 | AN32E_HUMAN | Acidic leucine-rich nuclear phosphoprotein 32 family member E | 0.72787 | 0.02420 |
| Q15758 | AAAT_HUMAN | Neutral amino acid transporter B(0) | 0.72847 | -0.03558 |
| P16152 | CBR1_HUMAN | Carbonyl reductase [NADPH] 1 | 0.73048 | -0.02847 |
| P30086 | PEBP1_HUMAN | Phosphatidylethanolamine-binding protein 1 | 0.73198 | -0.02811 |
| Q99856 | ARI3A_HUMAN | AT-rich interactive domain-containing protein 3A | 0.73304 | 0.02951 |
| Q9C075 | K1C23_HUMAN | Keratin, type I cytoskeletal 23 | 0.73307 | 0.03770 |
| Q9UNZ2 | NSF1C_HUMAN | NSFL1 cofactor p47 | 0.73499 | 0.03450 |
| P08575 | PTPRC_HUMAN | Receptor-type tyrosine-protein phosphatase C | 0.7369 | 0.07833 |
| P34897 | GLYM_HUMAN | Serine hydroxymethyltransferase, mitochondrial | 0.74007 | -0.02472 |
| P30622 | CLIP1_HUMAN | CAP-Gly domain-containing linker protein 1 | 0.74083 | 0.03023 |
| O14980 | XPO1_HUMAN | Exportin-1 | 0.74088 | 0.03348 |
| Q92616 | GCN1_HUMAN | eIF-2-alpha kinase activator GCN1 | 0.74124 | 0.05773 |
| P38919 | IF4A3_HUMAN | Eukaryotic initiation factor 4A-III | 0.74175 | -0.05127 |
| P62879 | GBB2_HUMAN | Guanine nucleotide-binding protein G(I)/G(S)/G(T) subunit beta-2 | 0.74195 | 0.02940 |
| Q9NZM1 | MYOF_HUMAN | Myoferlin | 0.74266 | -0.01834 |
| P00488 | F13A_HUMAN | Coagulation factor XIII A chain | 0.74361 | 0.06605 |
| P13760 | 2B14_HUMAN | HLA class II histocompatibility antigen, DRB1-4 beta chain | 0.74548 | -0.08936 |
| P61006 | RAB8A_HUMAN | Ras-related protein Rab-8A | 0.74566 | -0.04487 |
| Q92896 | GSLG1_HUMAN | Golgi apparatus protein 1 | 0.74699 | -0.01717 |
| Q9GZU2 | PEG3_HUMAN | Paternally-expressed gene 3 protein | 0.74753 | -0.06462 |
| P23284 | PPIB_HUMAN | Peptidyl-prolyl cis-trans isomerase B | 0.74799 | -0.02244 |
| O60784 | TOM1_HUMAN | Target of Myb protein 1 | 0.74809 | 0.01560 |
| Q04837 | SSBP_HUMAN | Single-stranded DNA-binding protein, mitochondrial | 0.74908 | 0.05772 |
| Q96GT9 | XAGE2_HUMAN | X antigen family member 2 | 0.74922 | 0.04698 |
| Q03518 | TAP1_HUMAN | Antigen peptide transporter 1 | 0.75108 | 0.04876 |
| P31153 | METK2_HUMAN | S-adenosylmethionine synthase isoform type-2 | 0.75118 | -0.02954 |
| O75694 | NU155_HUMAN | Nuclear pore complex protein Nup155 | 0.75144 | 0.02339 |
| Q9NTK5 | OLA1_HUMAN | Obg-like ATPase 1 | 0.75235 | -0.01118 |
| P11766 | ADHX_HUMAN | Alcohol dehydrogenase class-3 | 0.75282 | -0.07635 |
| P01023 | A2MG_HUMAN | Alpha-2-macroglobulin | 0.75351 | -0.05466 |
| P14625 | ENPL_HUMAN | Endoplasmin | 0.75424 | 0.01283 |
| Q16658 | FSCN1_HUMAN | Fascin | 0.7544 | -0.04700 |
| Q9Y696 | CLIC4_HUMAN | Chloride intracellular channel protein 4 | 0.75586 | -0.01420 |
| P07686 | HEXB_HUMAN | Beta-hexosaminidase subunit beta | 0.75673 | 0.01292 |
| P55957 | BID_HUMAN | BH3-interacting domain death agonist | 0.75678 | 0.03762 |
| Q15436 | SC23A_HUMAN | Protein transport protein Sec23A | 0.75739 | -0.04975 |
| P00558 | PGK1_HUMAN | Phosphoglycerate kinase 1 | 0.75898 | 0.01551 |
| Q9UNH7 | SNX6_HUMAN | Sorting nexin-6 | 0.75979 | 0.01472 |
| Q15181 | IPYR_HUMAN | Inorganic pyrophosphatase | 0.76044 | -0.02549 |
| P10599 | THIO_HUMAN | Thioredoxin | 0.76057 | 0.05390 |
| P04899 | GNAI2_HUMAN | Guanine nucleotide-binding protein G(i) subunit alpha-2 | 0.76065 | 0.02617 |
| P13716 | HEM2_HUMAN | Delta-aminolevulinic acid dehydratase | 0.76104 | -0.06129 |
| P13928 | ANXA8_HUMAN | Annexin A8 | 0.76418 | -0.04530 |
| P28072 | PSB6_HUMAN | Proteasome subunit beta type-6 | 0.76429 | 0.02180 |
| Q9HC38-2 | GLOD4_HUMAN | Isoform 2 of Glyoxalase domain-containing protein 4 | 0.76525 | -0.08006 |
| Q9UQ80 | PA2G4_HUMAN | Proliferation-associated protein 2G4 | 0.76675 | -0.05109 |
| O95433 | AHSA1_HUMAN | Activator of 90 kDa heat shock protein ATPase homolog 1 | 0.7671 | 0.00951 |
| P62979 | RS27A_HUMAN | Ubiquitin-40S ribosomal protein S27a | 0.76762 | 0.00934 |
| P13861 | KAP2_HUMAN | cAMP-dependent protein kinase type II-alpha regulatory subunit | 0.7677 | 0.03843 |
| P78527 | PRKDC_HUMAN | DNA-dependent protein kinase catalytic subunit | 0.76835 | -0.05177 |
| Q9H299 | SH3L3_HUMAN | SH3 domain-binding glutamic acid-rich-like protein 3 | 0.77035 | 0.08566 |
| P15170 | ERF3A_HUMAN | Eukaryotic peptide chain release factor GTP-binding subunit ERF3A | 0.77138 | 0.02245 |
| P19971 | TYPH_HUMAN | Thymidine phosphorylase | 0.77288 | 0.04195 |
| P13796 | PLSL_HUMAN | Plastin-2 | 0.77414 | 0.05239 |
| Q16795 | NDUA9_HUMAN | NADH dehydrogenase [ubiquinone] 1 alpha subcomplex subunit 9, mitochondrial | 0.77455 | -0.04410 |
| P62807 | H2B1C_HUMAN | Histone H2B type 1-C/E/F/G/I | 0.77681 | 0.03571 |
| Q9UHX1 | PUF60_HUMAN | Poly(U)-binding-splicing factor PUF60 | 0.77758 | 0.05545 |
| P21399 | ACOC_HUMAN | Cytoplasmic aconitate hydratase | 0.77779 | 0.03295 |
| Q16831 | UPP1_HUMAN | Uridine phosphorylase 1 | 0.77927 | 0.02715 |
| P09382 | LEG1_HUMAN | Galectin-1 | 0.78203 | 0.06713 |
| O75131 | CPNE3_HUMAN | Copine-3 | 0.78216 | 0.04365 |
| P36543 | VATE1_HUMAN | V-type proton ATPase subunit E 1 | 0.7841 | 0.02510 |
| P49720 | PSB3_HUMAN | Proteasome subunit beta type-3 | 0.78419 | -0.01546 |
| P08238 | HS90B_HUMAN | Heat shock protein HSP 90-beta | 0.78571 | -0.00671 |
| Q06210 | GFPT1_HUMAN | Glutamine--fructose-6-phosphate aminotransferase [isomerizing] 1 | 0.78672 | 0.01392 |
| P28070 | PSB4_HUMAN | Proteasome subunit beta type-4 | 0.78711 | -0.01617 |
| P60468 | SC61B_HUMAN | Protein transport protein Sec61 subunit beta | 0.78931 | 0.01179 |
| O95336 | 6PGL_HUMAN | 6-phosphogluconolactonase | 0.79048 | 0.01482 |
| Q15067 | ACOX1_HUMAN | Peroxisomal acyl-coenzyme A oxidase 1 | 0.79385 | 0.02392 |
| Q9UGI8-2 | TES_HUMAN | Isoform 2 of Testin | 0.79386 | 0.01290 |
| P63000 | RAC1_HUMAN | Ras-related C3 botulinum toxin substrate 1 | 0.79494 | 0.01281 |
| Q13155 | AIMP2_HUMAN | Aminoacyl tRNA synthase complex-interacting multifunctional protein 2 | 0.79544 | 0.00586 |
| O75367 | H2AY_HUMAN | Core histone macro-H2A.1 | 0.79594 | 0.03581 |
| Q8N1P7 | CRBG2_HUMAN | Beta/gamma crystallin domain-containing protein 2 | 0.79604 | -0.03875 |
| Q9NTX5 | ECHD1_HUMAN | Ethylmalonyl-CoA decarboxylase | 0.79611 | 0.09499 |
| Q96G03 | PGM2_HUMAN | Phosphoglucomutase-2 | 0.79636 | 0.00963 |
| P13667 | PDIA4_HUMAN | Protein disulfide-isomerase A4 | 0.79668 | -0.01021 |
| P08567 | PLEK_HUMAN | Pleckstrin | 0.79672 | 0.05763 |
| P31939 | PUR9_HUMAN | Bifunctional purine biosynthesis protein PURH | 0.79708 | 0.01260 |
| Q96QR8 | PURB_HUMAN | Transcriptional activator protein Pur-beta | 0.79753 | -0.06630 |
| P63151 | 2ABA_HUMAN | Serine/threonine-protein phosphatase 2A 55 kDa regulatory subunit B alpha isoform | 0.79825 | 0.02273 |
| Q99497 | PARK7_HUMAN | Protein/nucleic acid deglycase DJ-1 | 0.79939 | 0.03749 |
| P27338 | AOFB_HUMAN | Amine oxidase [flavin-containing] B | 0.79992 | -0.07233 |
| Q8TAT6 | NPL4_HUMAN | Nuclear protein localization protein 4 homolog | 0.80014 | 0.00730 |
| P25815 | S100P_HUMAN | Protein S100-P | 0.80118 | 0.03023 |
| P07195 | LDHB_HUMAN | L-lactate dehydrogenase B chain | 0.80278 | -0.02145 |
| Q04695 | K1C17_HUMAN | Keratin, type I cytoskeletal 17 | 0.80505 | 0.02581 |
| P06576 | ATPB_HUMAN | ATP synthase subunit beta, mitochondrial | 0.80566 | -0.00831 |
| Q9Y2Q3 | GSTK1_HUMAN | Glutathione S-transferase kappa 1 | 0.80582 | -0.01322 |
| P05455 | LA_HUMAN | Lupus La protein | 0.80784 | -0.03780 |
| P49588 | SYAC_HUMAN | Alanine--tRNA ligase, cytoplasmic | 0.80808 | -0.05268 |
| P08243 | ASNS_HUMAN | Asparagine synthetase [glutamine-hydrolyzing] | 0.80868 | -0.01920 |
| P31150 | GDIA_HUMAN | Rab GDP dissociation inhibitor alpha | 0.80939 | 0.03128 |
| P80303 | NUCB2_HUMAN | Nucleobindin-2 | 0.81292 | -0.02697 |
| P16401 | H15_HUMAN | Histone H1.5 | 0.81407 | -0.01552 |
| Q6DD88 | ATLA3_HUMAN | Atlastin-3 | 0.815 | 0.01203 |
| Q08722 | CD47_HUMAN | Leukocyte surface antigen CD47 | 0.81525 | 0.03884 |
| P16144 | ITB4_HUMAN | Integrin beta-4 | 0.8159 | 0.02038 |
| Q01518 | CAP1_HUMAN | Adenylyl cyclase-associated protein 1 | 0.81778 | 0.01673 |
| P08134 | RHOC_HUMAN | Rho-related GTP-binding protein RhoC | 0.81897 | 0.00659 |
| Q9P035 | HACD3_HUMAN | Very-long-chain (3R)-3-hydroxyacyl-CoA dehydratase 3 | 0.81913 | 0.02269 |
| Q96CX2 | KCD12_HUMAN | BTB/POZ domain- containing protein KCTD12 | 0.81964 | 0.03219 |
| P50395 | GDIB_HUMAN | Rab GDP dissociation inhibitor beta | 0.82023 | -0.01913 |
| P49023 | PAXI_HUMAN | Paxillin | 0.8206 | -0.01792 |
| P63241 | IF5A1_HUMAN | Eukaryotic translation initiation factor 5A-1 | 0.82114 | 0.04062 |
| Q9Y3A6 | TMED5_HUMAN | Transmembrane emp24 domain-containing protein 5 | 0.82134 | 0.04774 |
| Q99714 | HCD2_HUMAN | 3-hydroxyacyl-CoA dehydrogenase type-2 | 0.82153 | 0.02619 |
| Q5JPE7 | NOMO2_HUMAN | Nodal modulator 2 | 0.82489 | -0.02244 |
| P42167 | LAP2B_HUMAN | Lamina-associated polypeptide 2, isoforms beta/gamma | 0.82503 | -0.00784 |
| P17980 | PRS6A_HUMAN | 26S proteasome regulatory subunit 6A | 0.8256 | -0.02871 |
| P00390 | GSHR_HUMAN | Glutathione reductase, mitochondrial | 0.82707 | 0.00620 |
| P12270 | TPR_HUMAN | Nucleoprotein TPR | 0.8283 | -0.03517 |
| Q04446 | GLGB_HUMAN | 1,4-alpha-glucan-branching enzyme | 0.82863 | 0.04116 |
| P46926 | GNPI1_HUMAN | Glucosamine-6-phosphate isomerase 1 | 0.82886 | 0.01022 |
| P13987 | CD59_HUMAN | CD59 glycoprotein | 0.83032 | -0.01122 |
| P62805 | H4_HUMAN | Histone H4 | 0.83329 | 0.02733 |
| P23634 | AT2B4_HUMAN | Plasma membrane calcium-transporting ATPase 4 | 0.83551 | 0.01635 |
| P46779 | RL28_HUMAN | 60S ribosomal protein L28 | 0.83645 | -0.01914 |
| Q13263 | TIF1B_HUMAN | Transcription intermediary factor 1-beta | 0.83768 | -0.01132 |
| P18669 | PGAM1_HUMAN | Phosphoglycerate mutase 1 | 0.8377 | 0.00772 |
| P12532 | KCRU_HUMAN | Creatine kinase U-type, mitochondrial | 0.83796 | -0.04195 |
| Q32P28-3 | P3H1_HUMAN | Isoform 3 of Prolyl 3-hydroxylase 1 | 0.83994 | -0.01468 |
| A1L0T0 | ILVBL_HUMAN | Acetolactate synthase-like protein | 0.84074 | 0.00865 |
| P06702 | S10A9_HUMAN | Protein S100-A9 | 0.84138 | 0.03563 |
| Q9ULZ3 | ASC_HUMAN | Apoptosis-associated speck-like protein containing a CARD | 0.84186 | -0.03569 |
| Q6PIU2 | NCEH1_HUMAN | Neutral cholesterol ester hydrolase 1 | 0.84194 | 0.01556 |
| P11234 | RALB_HUMAN | Ras-related protein Ral-B | 0.84268 | -0.01350 |
| P32456 | GBP2_HUMAN | Guanylate-binding protein 2 | 0.84443 | 0.01456 |
| P62191 | PRS4_HUMAN | 26S proteasome regulatory subunit 4 | 0.84449 | 0.02146 |
| P21796 | VDAC1_HUMAN | Voltage-dependent anion-selective channel protein 1 | 0.84516 | -0.01001 |
| Q04637-8 | IF4G1_HUMAN | Isoform 8 of Eukaryotic translation initiation factor 4 gamma 1 | 0.84867 | 0.00918 |
| O60716-11 | CTND1_HUMAN | Isoform 2AC of Catenin delta-1 | 0.8495 | -0.01503 |
| P61586 | RHOA_HUMAN | Transforming protein RhoA | 0.84975 | -0.02401 |
| P62820 | RAB1A_HUMAN | Ras-related protein Rab-1A | 0.85012 | 0.00868 |
| Q32MZ4 | LRRF1_HUMAN | Leucine-rich repeat flightless-interacting protein 1 | 0.8519 | 0.01827 |
| Q8TBC4 | UBA3_HUMAN | NEDD8-activating enzyme E1 catalytic subunit | 0.85212 | -0.01642 |
| Q6NZI2 | CAVN1_HUMAN | Caveolae-associated protein 1 | 0.85217 | -0.01470 |
| Q13148 | TADBP_HUMAN | TAR DNA-binding protein 43 | 0.85479 | -0.01687 |
| Q9BQE3 | TBA1C_HUMAN | Tubulin alpha-1C chain | 0.85739 | -0.01949 |
| P08842 | STS_HUMAN | Steryl-sulfatase | 0.8574 | -0.00934 |
| P18206 | VINC_HUMAN | Vinculin | 0.8577 | 0.01598 |
| P53618 | COPB_HUMAN | Coatomer subunit beta | 0.85824 | -0.02092 |
| O14745 | NHRF1_HUMAN | Na(+)/H(+) exchange regulatory cofactor NHE-RF1 | 0.85888 | 0.02396 |
| P12277 | KCRB_HUMAN | Creatine kinase B-type | 0.85917 | 0.03168 |
| P23434 | GCSH_HUMAN | Glycine cleavage system H protein, mitochondrial | 0.86011 | -0.04693 |
| P09211 | GSTP1_HUMAN | Glutathione S-transferase P | 0.86272 | -0.01356 |
| P04406 | G3P_HUMAN | Glyceraldehyde-3-phosphate dehydrogenase | 0.86279 | 0.00947 |
| P54727 | RD23B_HUMAN | UV excision repair protein RAD23 homolog B | 0.86354 | -0.01544 |
| Q9BXJ9 | NAA15_HUMAN | N-alpha-acetyltransferase 15, NatA auxiliary subunit | 0.86669 | -0.01354 |
| P05091 | ALDH2_HUMAN | Aldehyde dehydrogenase, mitochondrial | 0.86687 | -0.03376 |
| P61163 | ACTZ_HUMAN | Alpha-centractin | 0.86769 | 0.01888 |
| P51858 | HDGF_HUMAN | Hepatoma-derived growth factor | 0.8681 | 0.01844 |
| Q16555 | DPYL2_HUMAN | Dihydropyrimidinase-related protein 2 | 0.86829 | 0.01010 |
| P28066 | PSA5_HUMAN | Proteasome subunit alpha type-5 | 0.86829 | -0.01058 |
| P51148 | RAB5C_HUMAN | Ras-related protein Rab-5C | 0.86905 | -0.00819 |
| Q9NS69 | TOM22_HUMAN | Mitochondrial import receptor subunit T | 0.87027 | -0.01494 |
| Q8IYB3 | SRRM1_HUMAN | Serine/arginine repetitive matrix protein 1 | 0.87103 | -0.02021 |
| Q9NZZ3 | CHMP5_HUMAN | Charged multivesicular body protein 5 | 0.87163 | 0.01486 |
| Q15417 | CNN3_HUMAN | Calponin-3 | 0.87404 | 0.01942 |
| P11310-2 | ACADM_HUMAN | Isoform 2 of Medium-chain specific acyl-CoA dehydrogenase, mitochondrial | 0.87438 | 0.00936 |
| O00151 | PDLI1_HUMAN | PDZ and LIM domain protein 1 | 0.87563 | 0.03187 |
| P01920 | DQB1_HUMAN | HLA class II histocompatibility antigen, DQ beta 1 chain | 0.87604 | -0.03066 |
| Q99613 | EIF3C_HUMAN | Eukaryotic translation initiation factor 3 subunit C | 0.88227 | 0.00333 |
| O43493 | TGON2_HUMAN | Trans-Golgi network integral membrane protein 2 | 0.88414 | -0.00646 |
| P67775 | PP2AA_HUMAN | Serine/threonine-protein phosphatase 2A catalytic subunit alpha isoform | 0.8845 | 0.00803 |
| Q96AC1 | FERM2_HUMAN | Fermitin family homolog 2 | 0.88542 | 0.00853 |
| O60271 | JIP4_HUMAN | C-Jun-amino-terminal kinase-interacting protein 4 | 0.88749 | -0.01213 |
| P02792 | FRIL_HUMAN | Ferritin light chain | 0.88845 | 0.02845 |
| Q14694 | UBP10_HUMAN | Ubiquitin carboxyl-terminal hydrolase 10 | 0.88962 | -0.01552 |
| Q86V81 | THOC4_HUMAN | THO complex usbunit 4 | 0.88971 | -0.00886 |
| P83105 | HTRA4_HUMAN | Serine protease HTRA4 | 0.88976 | 0.01597 |
| Q9BWM7 | SFXN3_HUMAN | Sideroflexin-3 | 0.89 | -0.01751 |
| P50213 | IDH3A_HUMAN | Isocitrate dehydrogenase [NAD] subunit alpha, mitochondrial | 0.89103 | 0.01532 |
| P61088 | UBE2N_HUMAN | Ubiquitin-conjugating enzyme E2 N | 0.89113 | -0.01224 |
| P07942 | LAMB1_HUMAN | Laminin subunit beta-1 | 0.89157 | -0.07566 |
| P36871 | PGM1_HUMAN | Phosphoglucomutase-1 | 0.89268 | 0.00611 |
| P98164 | LRP2_HUMAN | Low-density lipoprotein receptor-related protein 2 | 0.89415 | -0.01946 |
| O95831 | AIFM1_HUMAN | Apoptosis-inducing factor 1, mitochondrial | 0.89453 | 0.00773 |
| Q27J81 | INF2_HUMAN | Inverted formin-2 | 0.89465 | 0.04623 |
| Q99829 | CPNE1_HUMAN | Copine-1 | 0.895 | 0.00961 |
| P08754 | GNAI3_HUMAN | Guanine nucleotide-binding protein G(i) subunit alpha | 0.8952 | 0.01547 |
| P68402 | PA1B2_HUMAN | Platelet-activating factor acetylhydrolase IB subunit beta | 0.89619 | 0.01068 |
| Q9NW15 | ANO10_HUMAN | Anoctamin-10 | 0.89689 | -0.02177 |
| P48147 | PPCE_HUMAN | Prolyl endopeptidase | 0.89814 | -0.00995 |
| P04179 | SODM_HUMAN | Superoxide dismutase [Mn], mitochondrial | 0.89908 | -0.01971 |
| Q96TA1 | NIBL1_HUMAN | Niban-like protein 1 | 0.89931 | -0.00827 |
| P42765 | THIM_HUMAN | 3-ketoacyl-CoA thiolase, mitochondrial | 0.89944 | -0.01277 |
| P30419 | NMT1_HUMAN | Glycylpeptide N-tetradecanoyltransferase 1 | 0.90145 | 0.01640 |
| Q9UBE0 | SAE1_HUMAN | SUMO- activating enzyme ubunit 1 | 0.90171 | 0.00532 |
| P53004 | BIEA_HUMAN | Biliverdin reductase A | 0.90274 | -0.02036 |
| Q07666 | KHDR1_HUMAN | KH domain-containing, RNA-binding, signal transduction-associated protein 1 | 0.90377 | 0.00845 |
| P05386 | RLA1_HUMAN | 60S acidic ribosomal protein P1 | 0.90435 | 0.01322 |
| Q99959 | PKP2_HUMAN | Plakophilin-2 | 0.90476 | 0.01580 |
| P35221 | CTNA1_HUMAN | Catenin alpha-1 | 0.90576 | 0.00813 |
| P50570 | DYN2_HUMAN | Dynamin-2 | 0.90734 | 0.00435 |
| P62304 | RUXE_HUMAN | Small nuclear ribonucleoprotein E | 0.90796 | -0.01330 |
| P06730-2 | IF4E_HUMAN | Isoform 2 of Eukaryotic translation initiation factor 4E | 0.909 | 0.00585 |
| P26038 | MOES_HUMAN | Moesin | 0.9095 | 0.01675 |
| P53990 | IST1_HUMAN | IST1 homolog | 0.91008 | 0.00592 |
| P0DP25 | CALM3_HUMAN | Calmodulin-3 | 0.91255 | -0.00686 |
| Q13200 | PSMD2_HUMAN | 26S proteasome non-ATPase regulatory subunit 2 | 0.91457 | 0.00542 |
| P04083 | ANXA1_HUMAN | Annexin A1 | 0.91527 | 0.00671 |
| P41252 | SYIC_HUMAN | Isoleucine--tRNA ligase, cytoplasmic | 0.91547 | 0.00532 |
| O60763 | USO1_HUMAN | General vesicular transport factor p115 | 0.91623 | -0.00965 |
| P23142 | FBLN1_HUMAN | Fibulin-1 | 0.91638 | 0.00491 |
| Q16527 | CSRP2_HUMAN | Cysteine and glycine-rich protein 2 | 0.9165 | -0.00862 |
| Q00839 | HNRPU_HUMAN | Heterogeneous nuclear ribonucleoprotein U | 0.91747 | 0.00625 |
| Q14134 | TRI29_HUMAN | Tripartite motif-containing protein 29 | 0.91883 | 0.02345 |
| P55327-4 | TPD52_HUMAN | Isoform 4 of Tumor protein D52 | 0.92025 | 0.00967 |
| P35580-4 | MYH10_HUMAN | Isoform 4 of Myosin-10 | 0.92137 | -0.02653 |
| O43175 | SERA_HUMAN | D-3-phosphoglycerate dehydrogenase | 0.92282 | 0.00904 |
| P59666 | DEF3_HUMAN | Neutrophil defensin 3 | 0.92307 | -0.02577 |
| P37108 | SRP14_HUMAN | Signal recognition particle 14 kDa protein | 0.92387 | 0.00729 |
| P29692 | EF1D_HUMAN | Elongation factor 1-delta | 0.92402 | -0.01422 |
| Q96QK1 | VPS35_HUMAN | Vacuolar protein sorting-associated protein 35 | 0.92482 | -0.00386 |
| Q9NR45 | SIAS_HUMAN | Sialic acid synthase | 0.92541 | -0.00536 |
| O94776 | MTA2_HUMAN | Metastasis-associated protein MTA2 | 0.92599 | 0.00804 |
| P49189 | AL9A1_HUMAN | 4-trimethylaminobutyraldehyde dehydrogenase | 0.92728 | 0.00411 |
| P35754 | GLRX1_HUMAN | Glutaredoxin-1 | 0.92956 | -0.01902 |
| P46776 | RL27A_HUMAN | 60S ribosomal protein L27a | 0.93137 | -0.01530 |
| Q15293 | RCN1_HUMAN | Reticulocalbin-1 | 0.93174 | -0.00623 |
| Q7Z4W1 | DCXR_HUMAN | L-xylulose reductase | 0.93251 | -0.00649 |
| Q9NZ08 | ERAP1_HUMAN | Endoplasmic reticulum aminopeptidase 1 | 0.93326 | -0.00576 |
| P46782 | RS5_HUMAN | 40S ribosomal protein S5 | 0.9344 | 0.00558 |
| Q15046 | SYK_HUMAN | Lysine--tRNA ligase | 0.93449 | -0.00447 |
| P50995 | ANX11_HUMAN | Annexin A11 | 0.93479 | 0.00453 |
| O43488 | ARK72_HUMAN | Aflatoxin B1 aldehyde reductase member 2 | 0.93523 | 0.00638 |
| Q02218 | ODO1_HUMAN | 2-oxoglutarate dehydrogenase, mitochondrial | 0.93552 | 0.00410 |
| Q4VCS5 | AMOT_HUMAN | Angiomotin | 0.93774 | -0.00939 |
| P80723 | BASP1_HUMAN | Brain acid soluble protein 1 | 0.93788 | 0.00301 |
| P41091 | IF2G_HUMAN | Eukaryotic translation initiation factor 2 subunit 3 | 0.93833 | -0.00385 |
| Q9ULV4 | COR1C_HUMAN | Coronin-1C | 0.93924 | 0.00401 |
| Q15075 | EEA1_HUMAN | Early endosome antigen 1 | 0.93928 | -0.01025 |
| P09601 | HMOX1_HUMAN | Heme oxygenase 1 | 0.93991 | -0.00475 |
| Q93050 | VPP1_HUMAN | V-type proton ATPase 116 kDa subunit a isoform 1 | 0.94087 | -0.00337 |
| P68363 | TBA1B_HUMAN | Tubulin alpha-1B chain | 0.94201 | 0.00549 |
| P06753-2 | TPM3_HUMAN | Isoform 2 of Tropomyosin alpha-3 chain | 0.94562 | 0.00549 |
| Q8WUM4 | PDC6I_HUMAN | Programmed cell death 6-interacting protein | 0.94618 | 0.00232 |
| Q9NPH2 | INO1_HUMAN | Inositol-3-phosphate synthase 1 | 0.94833 | 0.00659 |
| Q9NUV9 | GIMA4_HUMAN | GTPase IMAP family member 4 | 0.94855 | 0.01306 |
| P05114 | HMGN1_HUMAN | Non-histone chromosomal protein HMG-14 | 0.95219 | -0.01858 |
| P14317 | HCLS1_HUMAN | Hematopoietic lineage cell-specific protein | 0.95298 | -0.00583 |
| P48643 | TCPE_HUMAN | T-complex protein 1 subunit epsilon | 0.95305 | 0.00717 |
| P27797 | CALR_HUMAN | Calreticulin | 0.95585 | 0.00178 |
| Q9Y3I0 | RTCB_HUMAN | tRNA-splicing ligase RtcB homolog | 0.9569 | 0.00254 |
| Q14683 | SMC1A_HUMAN | Structural maintenance of chromosomes protein 1A | 0.95768 | -0.00897 |
| Q16851 | UGPA_HUMAN | UTP--glucose-1-phosphate uridylyltransferase | 0.95798 | 0.00315 |
| Q9NNW7-2 | TRXR2_HUMAN | Isoform 2 of Thioredoxin reductase 2, mitochondrial | 0.95804 | 0.00689 |
| P50454 | SERPH_HUMAN | Serpin H1 | 0.95806 | -0.00424 |
| Q12959 | DLG1_HUMAN | Disks large homolog 1 | 0.95869 | 0.00286 |
| Q96M27 | PRRC1_HUMAN | Protein PRRC1 | 0.95912 | 0.00522 |
| Q9P0L0 | VAPA_HUMAN | Vesicle-associated membrane protein-associated protein A | 0.95941 | 0.00397 |
| P05787 | K2C8_HUMAN | Keratin, type II cytoskeletal 8 | 0.96467 | 0.00163 |
| Q9Y6E2 | BZW2_HUMAN | Basic leucine zipper and W2 domain-containing protein 2 | 0.96475 | 0.00521 |
| Q86VP6 | CAND1_HUMAN | Cullin-associated NEDD8-dissociated protein 1 | 0.96627 | 0.00263 |
| Q8WTP9 | XAGE3_HUMAN | X antigen family member 3 | 0.96714 | 0.00700 |
| P30041 | PRDX6_HUMAN | Peroxiredoxin-6 | 0.96746 | 0.00148 |
| Q9BV40 | VAMP8_HUMAN | Vesicle-associated membrane protein 8 | 0.96792 | 0.00236 |
| P61981 | 1433G_HUMAN | 14-3-3 protein gamma | 0.96831 | -0.00296 |
| Q9UBS4 | DJB11_HUMAN | DnaJ homolog subfamily B member 11 | 0.96885 | -0.00421 |
| P63244 | RACK1_HUMAN | Receptor of activated protein C kinase 1 | 0.97271 | -0.00102 |
| P45974 | UBP5_HUMAN | Ubiquitin carboxyl-terminal hydrolase 5 | 0.97281 | 0.00198 |
| P13747 | HLAE_HUMAN | HLA class I histocompatibility antigen, alpha chain E | 0.97451 | 0.00277 |
| Q9UL46 | PSME2_HUMAN | Proteasome activator complex subunit 2 | 0.97485 | 0.00289 |
| O95484 | CLD9_HUMAN | Claudin-9 | 0.97577 | -0.00340 |
| Q9Y4E8 | UBP15_HUMAN | Ubiquitin carboxyl-terminal hydrolase 15 | 0.9761 | 0.00089 |
| P26572 | MGAT1_HUMAN | Alpha-1,3-mannosyl-glycoprotein 2-beta-N-acetylglucosaminyltransferase | 0.97712 | -0.00196 |
| O95359 | TACC2_HUMAN | Transforming acidic coiled-coil-containing protein 2 | 0.97763 | -0.00214 |
| P00167 | CYB5_HUMAN | Cytochrome b5 | 0.97835 | 0.00172 |
| Q6ZT62 | BGIN_HUMAN | Bargin | 0.97902 | 0.00215 |
| Q86UE4 | LYRIC_HUMAN | Protein LYRIC | 0.9805 | -0.00429 |
| P38646 | GRP75_HUMAN | Stress-70 protein, mitochondrial | 0.98305 | -0.00170 |
| O95379 | TFIP8_HUMAN | Tumor necrosis factor alpha-induced protein 8 | 0.98333 | 0.00092 |
| P37802 | TAGL2_HUMAN | Transgelin-2 | 0.98357 | 0.00159 |
| P00533 | EGFR_HUMAN | Epidermal growth factor receptor | 0.98442 | -0.00072 |
| P62256 | UBE2H_HUMAN | Ubiquitin-conjugating enzyme E2 H | 0.9857 | -0.00125 |
| Q9NSE4 | SYIM_HUMAN | Isoleucine--tRNA ligase, mitochondrial | 0.98808 | -0.00165 |
| P35222 | CTNB1_HUMAN | Catenin beta-1 | 0.98957 | 0.00100 |
| P51153 | RAB13_HUMAN | Ras-related protein Rab-13 | 0.99065 | -0.00143 |
| Q07021 | C1QBP_HUMAN | Complement component 1 Q subcomponent-binding protein, mitochondrial | 0.99151 | 0.00048 |
| Q15393 | SF3B3_HUMAN | Splicing factor 3B subunit 3 | 0.99281 | 0.00062 |
| P23588 | IF4B_HUMAN | Eukaryotic translation initiation factor 4B | 0.99367 | 0.00290 |
| Q15185 | TEBP_HUMAN | Prostaglandin E synthase 3 | 0.99449 | 0.00036 |
| Q99460 | PSMD1_HUMAN | 26S proteasome non-ATPase regulatory subunit 1 | 0.99471 | -0.00040 |
| P05109 | S10A8_HUMAN | Protein S100-A8 | 0.99537 | 0.00095 |
| P61019 | RAB2A_HUMAN | Ras-related protein Rab-2A | 0.99685 | 0.00057 |
| P02533 | K1C14_HUMAN | Keratin, type I cytoskeletal 14 | 0.99686 | 0.00023 |
